# Developing and Validating Species Distribution Models for Wetland Plants Across Europe

**DOI:** 10.1002/ece3.71157

**Published:** 2025-04-23

**Authors:** Ojaswi Sumbh, Marjon Hellegers, Valerio Barbarossa, Renata Ćušterevska, Borja Jiménez‐Alfaro, Łukasz Kozub, Francesca Napoleone, Zvjezdana Stančić, Aafke M. Schipper

**Affiliations:** ^1^ PBL Netherlands Environmental Assessment Agency The Hague the Netherlands; ^2^ Radboud Institute for Biological and Environmental Sciences Radboud University Nijmegen the Netherlands; ^3^ Institute of Environmental Sciences Leiden University Leiden the Netherlands; ^4^ Faculty of Natural Sciences and Mathematics, Institute of Biology University of Ss. Cyril and Methodius Skopje Republic of Macedonia; ^5^ Joint Biodiversity Research Institute University of Oviedo Oviedo Asturias Spain; ^6^ Department of Ecology and Environmental Protection, Faculty of Biology, Institute of Environmental Biology University of Warsaw Warsaw Poland; ^7^ Department of Environmental Biology Sapienza University of Rome Rome Italy; ^8^ Faculty of Geotechnical Engineering University of Zagreb Varaždin Croatia

**Keywords:** biodiversity, BioScore, ecological indicator values, habitat suitability modelling, niche optima, species distribution modelling, wetland species

## Abstract

Drainage, agricultural conversion, and climate change threaten wetlands and their unique biodiversity. Species distribution models (SDMs) can help to identify effective conservation measures. However, existing SDMs for wetland plants are often geographically limited, miss variables representing hydrological conditions, and neglect moss species, essential to many wetlands. Here, we developed and validated SDMs for 265 vascular plant and moss species characteristic of European wetlands, using environmental variables representing climate, soil, hydrology, and anthropogenic pressures. We validated the spatial predictions of the SDMs through cross‐validation and against independent data from the Global Biodiversity Information Facility (GBIF). Further, we validated the niche optima of the species, as obtained from the modelled species response curves, with empirical niche optima. The spatial validation revealed good predictive power of the SDMs, especially for diagnostic mosses, for which we obtained median cross‐validated values of the area under the curve (AUC) and true skill statistic (TSS) of 0.93 and 0.73, respectively, and a median true positive rate (TPR) based on GBIF records of 0.77. SDMs of diagnostic vascular plants performed well, too, with median AUC, TSS, and TPR of 0.91, 0.69, and 0.67, respectively. SDMs of non‐diagnostic plants had the lowest performance, with median AUC, TSS, and TPR values of 0.84, 0.53, and 0.62, respectively. Correlations between modelled and empirical niche optima were typically in the expected direction. Climate variables, particularly the mean temperature of the coldest month, were the strongest predictors of species occurrence. At the same time, groundwater table depth was a significant predictor for diagnostic vascular plants but not for mosses. We concluded that our SDMs are suitable for predicting broad‐scale patterns of wetland plant species distributions as governed by climatic conditions. Alternative or additional variables or a different modelling approach might be needed to represent better the local heterogeneity in the hydrological conditions of wetlands.

## Graphical abstract

We fitted species distribution models (SDMs) for 265 wetland plant species in Europe. We validated predicted occurrences using independent data. The SDMs showed a high predictive performance, such that our SDMs can be used to predict broad‐scale distributions of wetland plants.

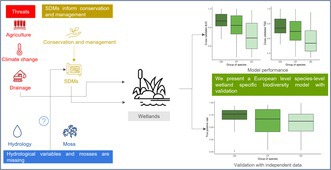

## 1 Introduction

Wetlands are biodiversity hotspots that provide essential ecosystem services, such as flood protection, carbon sequestration, and storage (Janse et al. 2019; Zedler and Kercher 2005). Approximately 5%–8% of the global land surface is categorized as wetlands, which comprise 20%–30% of the world's carbon pool (Salimi et al. 2021). However, wetlands are disproportionately threatened due to increasing demands for resources. Many wetlands have been drained and converted into arable land to meet the needs of the world's growing population (Fluet‐Chouinard et al. 2023). It is estimated that 3.4 million km^2^ of inland wetlands have been lost since 1700, corresponding with 21% of the world's wetlands (Fluet‐Chouinard et al. 2023). Further losses and degradation are expected as a result of further conversion to agricultural land, direct human impacts such as drainage, and changes in hydrological conditions due to climate change (Janse et al. 2019; Salimi et al. 2021). Climate change poses a significant threat to wetland ecosystems (Erwin 2009; Salimi et al. 2021). Climate change leads to higher temperatures and, thus, higher evapotranspiration, rainfall intensity, and frequency changes, and more frequent extreme climatic events such as droughts, flooding, and storms. These changes can modify wetland hydrology and biogeochemical processes so that vital ecosystem services may degrade or even transform into disservices. For instance, warmer conditions can enhance microbial activity, leading to increased emissions of nitrous oxide due to accelerated nitrification and denitrification processes (Huang et al. 2013; Salimi et al. 2021). Policymakers, scientists, and conservationists recognize the urgency to halt the loss of wetlands and their unique biodiversity (Janse et al. 2019), as reflected by various (inter)national agreements and targets related to biodiversity and sustainable development (Seifollahi‐Aghmiuni et al. 2019).

Spatially explicit modelling tools are key for evaluating and predicting the impacts of climate change, land use change, and other human impacts on biodiversity and identifying potential conservation and restoration measures (Backus et al. 2023; Rodrigues et al. 2015). Thanks to the increasing availability of data and computing power, species‐level modelling is increasingly being used to assess the impacts of human interventions on biodiversity (Araújo and New 2007). Species distribution models (SDMs) are extensively used to evaluate, for example, potential range shifts or declines in response to projected climate change (e.g., Kermavnar et al. 2023; Porfirio et al. 2014). However, SDMs representative of the unique biodiversity of wetlands are still in their infancy, and existing SDMs of wetland species typically have a limited geographic extent and miss out on moss species (Cao et al. 2020; Dang et al. 2021; Janse et al. 2019; Lou et al. 2018; Zhong et al. 2021). The latter is a key gap given the current lack of knowledge about the distributions of mosses and their key role in shaping wetland ecosystems, especially peatlands (Ferretto et al. 2023; Ma et al. 2022; Poncet et al. 2015). Further, wetlands are characterised by distinct hydrological conditions different from their surroundings regarding water levels and contributions of different water sources (surface water, groundwater, precipitation). These hydrological conditions play a key role in shaping plant distributions, yet hydrological variables are frequently missing from wetlands SDMs (Araya et al. 2010; Gardner et al. 2019). With climate change, profound impacts on wetlands are anticipated due to impacts on hydrological regimes (Zhong et al. 2021). Hence, including variables representing hydroclimatic conditions in wetland SDMs is essential. For example, Cao et al. (2020) found that precipitation‐related environmental factors mainly determined the predicted shifts in distribution ranges of six endangered wetland plant species in China. Similarly, Dang et al. (2021) showed that the habitat suitability of three wetland‐characteristic tree species in the Mekong Delta was governed by seasonal variation in precipitation, temperature, and sea level rise. These studies highlight the need to develop and improve SDMs of wetland species with environmental variables representative of wetlands.

In this study, we develop and validate SDMs for European wetland plants. We created SDMs for 265 vascular plant and moss species characteristic of European wetlands, using environmental variables representative of climate, soil, hydrology, and anthropogenic pressures. Specifically, we included three hydrological variables indicative of water availability and water level fluctuations. We evaluated the model fit based on cross‐validation and quantified the importance of each variable. We then validated the models' ability to accurately predict species occurrence by comparing the SDM predictions with independent occurrence data from the Global Biodiversity Information Facility (GBIF) database. In addition, we compared the niche optima obtained from the modelled response curves of the SDMs with empirical niche optima, thus testing the ecological realism of the modelled species' responses to the environmental conditions (Hellegers et al. 2020). For this purpose, we obtained ecological indicator values from the ecological indicator value in Europe (EIVE1.0) 1.0 database (Dengler et al. 2023). We performed the analysis for three distinct groups: moss species diagnostic of wetland vegetation, vascular plant species diagnostic of wetland vegetation, and non‐diagnostic plants (including mosses and vascular plants). Diagnostic species are more strongly associated with a particular habitat, absent or rare in other habitats, thus serving as indicators for distinguishing a habitat from others (Chytrý et al. 2020; Whittaker 1962). Given that our SDMs include wetland‐specific environmental variables, we expect the models of diagnostic species to perform better than those of non‐diagnostic species.

## 2 Methodology

### 2.1 Vegetation Plot and Species Selection

**FIGURE A1:**
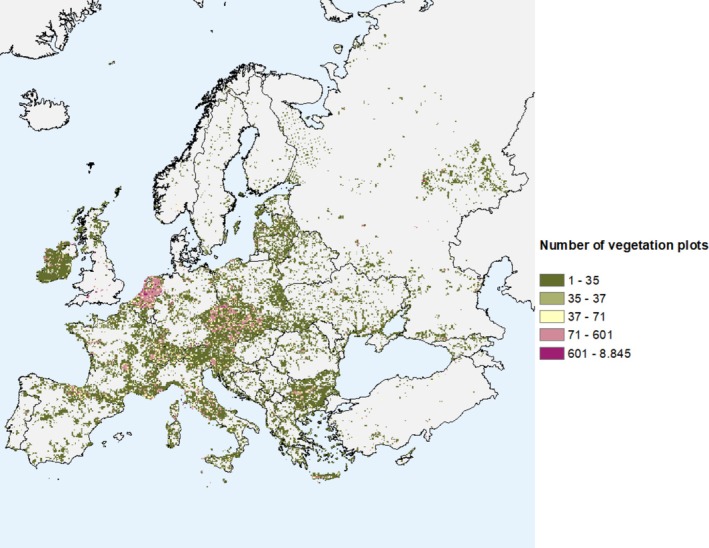
Map of the 533,254 vegetation plots used in the analyses, as obtained from the European vegetation archive.

For this study, we obtained a total of 1,652,563 vegetation plots from the European Vegetation Archive (EVA) (Chytrý et al. 2016). Following Hellegers et al. (2020), we excluded plots recorded before 1990 and after 2018, plots with a known spatial uncertainty larger than 1 km, and plots with missing values for one or more environmental variables (see next section). We also excluded plots that were classified as marine habitat types (MA), inland waters (P), and vegetated man‐made habitat types (V) according to the EUNIS (European Nature Information System) habitat type classification (Chytrý et al. 2020). Thus, we retained 533,254 vegetation plots, which equates to about 32% of the initial EVA vegetation plots (Figure A1).

Next, we selected vascular plant and moss species characteristic of wetlands based on the EUNIS habitat type classification (Chytrý et al. 2020). We selected species of the following wetlands habitat types: Q1—raised and blanket bogs, Q2—valley mires, poor fens, and transition mires, Q3—palsa and polygon mires, Q4—base‐rich fens and calcareous spring mires, Q5—helophyte beds, and Q6—periodically exposed shores (Chytrý et al. 2021). Raised and blanket bogs (Q1) rely mainly on rainfall for moisture and nutrients, forming highly acidic peat in cool climates with high rainfall. Valley mires (Q2) have peat‐forming vegetation that depends on water draining from the surrounding landscape. Poor fens are fed by acidic, nutrient‐poor groundwater, while transition mires support peat‐forming vegetation in areas with acidic groundwater or acidic pool and lake water, often forming floating vegetation rafts. Palsa and polygon mires (Q3) develop when thick peat is subject to sporadic permafrost, with low precipitation and an annual mean temperature below −1°C. Base‐rich fens and calcareous spring mires (Q4) are found in river valleys, alluvial plains, or hillsides. They depend on calcareous or general cation‐rich groundwater, with the water level at or near the surface. Peat forms here due to a permanently high water table. Helophyte beds (Q5) are related to nutrient‐rich waters of riverine or lake origin and can be both peat‐forming or related to non‐peaty substrates. Characteristic conditions are shallow to moderately deep mesotrophic to eutrophic fresh or slightly brackish waters along the banks of rivers and lakes. Periodically exposed shores (Q6) include riverbanks, sediment islets, drying oxbows, shallow water bodies like lakes and fishponds, and ditches. These areas share nutrient‐rich muddy or sandy‐muddy soils from natural sedimentation or human input. Among the listed habitat types, we excluded species of saline habitats (Q54; Q63). Chytrý et al. (2021) classify the characteristic species of EUNIS habitat types as diagnostic, dominant, and constant species. Our selection resulted in 364 plant species: 81 diagnostic moss species, 223 diagnostic vascular plant species, and 60 non‐diagnostic species, that is, dominant or constant vascular plants and mosses.

### 2.2 Environmental Variables

As a starting point for selecting environmental variables for the SDMs for wetland plant species across Europe, we used the climate, soil, and atmospheric nitrogen deposition variables from Hellegers et al. (2020), who used these variables to establish SDMs of terrestrial vascular plants across Europe. We supplemented this set with additional environmental variables that we expected to be relevant for wetland plants (Table 1). Temperature and precipitation are the most critical factors driving the large‐scale distributions of species (Gutiérrez‐Hernández and García 2021). Following Hellegers et al. (2020), we included four climatic variables that pose physiological limitations on large‐scale plant species distributions, namely total annual precipitation, mean temperature of the coldest month, annual growing degree days, and a water balance variable (Araújo et al. 2011; Lindborg et al. 2021; Wang et al. 2020). We calculated the annual growing degree days as the annual sum of daily temperature values above 5°C. We derived the daily temperature values from linear interpolation of mean monthly temperature values. We calculated water balance as the sum of the monthly differences in precipitation and potential evapotranspiration. We calculated the potential evapotranspiration (PET) per month as (Lugo et al. 1999):

(1) $${\mathrm{PET}}_{m}=0\;\text{if}\;T_{m}\leq 0^{{}^{\circ}}C$$

(2) $${\mathrm{PET}}_{m}=58.93\times T_{m}\;\text{if}\;0^{{}^{\circ}}C<T_{m}<30^{{}^{\circ}}C$$

(3) $${\mathrm{PET}}_{m}=58.93\times 30\;\text{if}\;T_{m}\geq 30^{{}^{\circ}}C$$

PET_m_ is the monthly potential evapotranspiration (mm), and *T*_m_ is the monthly mean temperature (°C). We quantified all climate variables using mean monthly temperature and monthly precipitation from the CHELSA dataset version 2.1, as CHELSA data is adjusted for the effects of elevation and aspect, and we averaged the values over the period 1990–2018 to match the time frame of the vegetation plots (Karger et al. 2017, 2021). Following Hellegers et al. (2020), we also included various soil properties that are relevant for plant growth via water and nutrient retention capacity (Figueiredo et al. 2018; Trettin et al. 2020), collected from the SoilGrids dataset (Hengl et al. 2017). The physical and chemical properties of soil strongly affect the hydrology and hydrochemistry in wetlands and thus influence wetland formation and functioning (Kolka and Thompson 2006). We aimed to select soil variables directly influencing hydrological conductivity, water storage, and availability, which depend on soil texture, soil structure, bulk density, porosity, and pore size distribution (Kolka and Thompson 2006). To represent these physical properties, we selected the variables clay content, volume of coarse fragments, silt content, organic carbon content, sand content, and bulk density. Chemical soil properties important for plant growth include soil acidity and buffer capacity, which influence the solubility of various elements in the soil, particularly plant nutrients (Kolka and Thompson 2006). We, therefore, also included the variables soil pH and cation exchange capacity. We selected the values from the top 5 cm of the soil.

**TABLE 1:** Environmental variables selected for the SDMs for wetland‐characteristic vascular plant and moss species.

Environmental variable	Short name	Unit	Source	Native spatial resolution	Reference
Climate					
Mean temperature of the coldest month	MinTemp	°C	Chelsa	30 arcsec	Karger et al. (2017)
Total annual precipitation	Precipitation	mm	Chelsa	30 arcsec	
Annual growing degree days	TempSum	°C	Chelsa	30 arcsec	
Water balance	WB	mm	Chelsa	30 arcsec	
Soil					
Clay content	Clay	weight %	Soil grids	1 km	Hengl et al. (2017)
Soil pH × 10 in H_2_O	pH	pH	Soil grids	1 km	
Volume of coarse fragments	Coarse	volumetric %	Soil grids	1 km	
Silt content	Silt	weight %	Soil grids	1 km	
Organic carbon content	Carbon	g kg^−1^	Soil grids	1 km	
Cation exchange capacity of soil	CEC	cmolc kg^−1^	Soil grids	1 km	
Sand content	Sand	weight %	Soil grids	1 km	
Bulk density	Bulk	kg m^−3^	Soil grids	1 km	
Hydrology					
Groundwater table depth	GWTD	m	Fan et al. (2013)	30 arcsec	Fan et al. (2013)
Water and Wetness Probability Index (WWPI)	WWPI	dimensionless	Copernicus land monitoring service	10 m	European Environment Agency (2020)
Topographic wetness index	TWI	dimensionless	Marthews et al. (2015)	1 km	Marthews et al. (2015)
Others					
Nitrogen deposition	Ndp	mg m^−2^	EMEP	0.1°	Fagerli et al. (2019)
Anthropogenic land cover in upstream catchment	ALC	km^2^	this study	1 km	Coordination Centre for Effects (Gebhardt 2023)
Saltwater affected area	Salt	Yes/No	this study	1 km	Not applicable; see method description

Because excess water is an important prerequisite for the occurrence of wetland ecosystems and their characteristic vegetation, we included groundwater table depth (GWTD), topographic wetness index (TWI) and water, and wetness probability index (WWPI) (Choi et al. 2019; Fan et al. 2013; Guntenspergen et al. 1989; Raulings et al. 2010). These variables represent the groundwater levels, propensity to moisture accumulation, and inundation probability, respectively. We obtained the GWTD from Fan et al. (2013) and retrieved the TWI from Marthews et al. (2015). The TWI is based on the local downstream slope of a grid cell and its upstream catchment area. It is an indicator of the tendency of a site to accumulate water, given its topographic position. We obtained the WWPI from the Copernicus land monitoring service over the years 2009–2015 (European Environment Agency 2020). This index specifies the number of times that a grid cell is inundated or wet relative to the total number of valid observations, as follows:

(4) $$\text{WWPI}=\left(n_{\text{water}}+0.75\times n_{\mathrm{wet}}\right)/n_{\text{total}}\times 100$$

where *n*_water_ represents the number of water occurrences, *n*_wet_ represents the number of wet occurrences, and *n*_total_ represents the total number of water, wet, and dry occurrences.

Finally, we included the potential influence of salt, nitrogen deposition, and anthropogenic land cover in the upstream catchment; the last two variables indicate anthropogenic pressure on wetland plants. We calculated the potential influence of salt spray, salt‐water inundation, or salt‐water intrusion as a binary variable (0–1) reflecting whether a location is within 3 km of the nearest coastline (Du and Hesp 2020). We obtained nitrogen deposition in the year 2013 from (Fagerli et al. 2019) as an indicator of eutrophication because eutrophication is one of the threats to the wetland ecosystem and can significantly impact wetland vegetation (Borgström et al. 2024; Gustafson and Wang 2002; Yousaf et al. 2021). Lastly, we included the total area of anthropogenic land cover in the upstream catchment of each cell as a proxy of various human pressures affecting wetlands and their vegetation, including the inflow of nutrients and pollutants. To this end, we retrieved an EUNIS level 3 land cover map from the Coordination Centre for Effects (Gebhardt 2023). We derived an anthropogenic land cover map from this map by clustering all grid cells allocated to habitat types of class V1, that is, arable land and market gardens, and class J, that is, constructed, industrial, and other artificial habitats. Then, we resampled this layer to 30 arcsec to match the spatial extent of the hydrography layer from HydroSHEDS (Lehner et al. 2008). We used the anthropogenic land cover map and the hydrography layer to quantify the total area of anthropogenic land cover in the upstream catchment area of each grid cell using the AreaD8 function of the TauDEM toolbox from ArcGIS (version 10.8.1). We reprojected all the environmental variable layers to the ETRS89 Lambert azimuthal equal‐area coordinate system and resampled them to a 1 km resolution.

### 2.3 Fitting Species Distribution Models

We established an SDM for each selected species following the methodology described by Hellegers et al. (2020). Before fitting the SDMs, we performed a variance inflation factor (VIF) analysis to detect collinearity between the environmental variables, using the VIF function from the ‘used’ package (Naimi et al. 2014). Using a VIF threshold of 10 (Chatterjee and Hadi 2013), we excluded sand content, bulk density, and water balance, thus keeping 15 environmental variables for model fitting. We established the SDMs in R version 2024.04‐2+764 (R Core Team 2018) according to an ensemble modelling approach based on a generalised linear model (GLM), a generalised additive model (GAM) and boosted regression trees (BRT) using the ‘biomod2’ package (ver. 3.5.1) with the default settings (Thuiller et al. 2024). We adopted a random sampling approach to reduce spatial bias and pseudo‐replication, selecting only one vegetation plot per 1 km grid cell where the species was present. We selected species with a minimum of 94 presence points, ensuring five presences per environmental variable for model fitting, after setting aside 20% of the observations for cross‐validation, leaving 274 of the 364 species (Araújo et al. 2011; Hellegers et al. 2020). We selected absence records for each species by randomly sampling one plot per 1 km grid cell where the species was not recorded. Because of the large number of vegetation plots, we used a further subset of the absence records to reduce the computation time. For GAM and GLM, we selected a minimum of 10,000 absences, while for BRT, we selected a minimum of 1000 absences (Barbet‐Massin et al. 2012). If the count of presence records exceeded either 1000 or 10,000, we selected a number of absence records matching the number of presence records (Barbet‐Massin et al. 2012; Hellegers et al. 2020). We calibrated the models using a single random sample comprising 80% of the data and used the remaining 20% for evaluation. We established an ensemble model for each species using the three modelling techniques, each weighted with their cross‐validated true skill statistic (TSS) value (Hellegers et al. 2020). We quantified the performance of the ensemble model based on the evaluation data using the TSS and area under the receiver operating characteristic curve (AUC) (Allouche et al. 2006). Next, we selected species with an ensemble model with a cross‐validated TSS value ≥ 0.3 and a cross‐validated AUC ≥ 0.7 to remove species with a poorly performing model (Araújo et al. 2011), removing six species. Thereafter, we fitted the SDMs with 100% of the data. We discarded three species for which not all models converged. Thus, we obtained SDMs for 265 wetland plant species, of which 48 diagnostic moss species, 159 diagnostic vascular plant species, and 58 non‐diagnostic plant species (Supporting Information S1; Table A1). For each of the 265 species, we calculated the variable importance of each environmental variable as a weighted average of the variable importance values for individual modelling techniques, obtained with the ‘get_variables_importance’ tool from the ‘biomod2’ package (ver. 3.3–7) (Thuiller et al. 2024), using the cross‐validated TSS values for each of the three models as weights.

### 2.4 Validation of the SDMs

We validated the SDMs using independent data using two distinct validation methods. First, we compared the predicted distribution of each species with independent occurrence data from the GBIF, which contains occurrence data for a wide range of species worldwide (GBIF 2023). To that end, we first transformed the probabilities of occurrence (PoO) of each species as predicted by the ensemble model to a binary distribution map (present or absent) using a PoO threshold that maximised the TSS value (Araújo and Guisan 2006; Liu et al. 2005). We then compared the binarised output with GBIF occurrence records using the true positive rate (TPR), that is, the proportion of GBIF records corresponding with a predicted presence, as a performance measure. We retrieved species' presence records from 1990 to 2021 from GBIF using the ‘rgbif’ package in R (Chamberlain and Boettiger 2017). We could not retrieve GBIF records for three species because the taxonomic backbone did not match any records found in the GBIF database, leaving us with 262 species for validation. From GBIF, we excluded records with a known geospatial issue, a location uncertainty of more than 1 km, and records outside the study area. Further, we selected a random single GBIF presence value per 1 km grid cell to avoid pseudo‐replication. After these filters, there were 14 species without any occurrence records. This left 248 species for validation, of which 44 were diagnostic mosses, 148 diagnostic vascular plants, and 56 non‐diagnostic plants. Across all the species, we obtained a median of 14,966 records, with a minimum of four records for *Cirsium appendiculatum* and a maximum of 451,869 records for *Festuca rubra* .

Second, we tested the ecological realism of the modelled species responses to the environmental variables by comparing niche optima retrieved from the SDMs with independent empirical data on niche optima, using the method developed by Hellegers et al. (2020). For the empirical niche optima, we used ecological indicator values (EIVs), which indicate the preferred niche conditions of plant species for various environmental variables based on field observations of species co‐occurrence patterns, in situ measurements of environmental variables, and occasional experiments (Ellenberg 1974). EIVs are widely employed in vegetation science because they enable the assessment of environmental variables without direct measurements (Bartelheimer and Poschlod 2016). The comparison with EIVs was limited to vascular plant species due to a lack of data for moss species at the European scale. We collected EIVs for 208 vascular plant species, of which 158 were diagnostic and 50 non‐diagnostic, for soil moisture, soil nitrogen, soil acidity (pH), light and temperature, from ecological indicator values for Europe (EIVE1.0) 1.0, which represents the most extensive ecological indicator value database available for European vascular plants to date (Dengler et al. 2023). EIVE1.0 is a comprehensive database consisting of 31 EIV systems dating from 1956 to 2022, where in the case of multiple EIV systems available for a region, the latest and most comprehensive one was used (Dengler et al. 2023). Dengler et al. (2023) validated the EIVs against bioclimatic variables from Chelsa version 2.1, where they found a good correlation between the temperature variables in CHELSA and the temperature variable from EIVE1.0, indicating that the values are comparable. We extracted species‐specific modelled niche optima from the SDMs, from here on referred to as modelled indicator values (MIVs), as the values of the environmental variables corresponding to the highest PoO of the species (Hellegers et al. 2020). We extracted MIVs for each species for variables with a variable importance of at least 0.05 (Hellegers et al. 2020) (Table A2). To extract MIVs, we implemented the evaluation strip method proposed by Elith et al. (2005), using the ‘response.plot2’ tool from the ‘biomod2’ package (ver. 3.3–7) to obtain response curves (Thuiller et al. 2024). We obtained a response curve for each modelling technique by varying the environmental variable of interest across its range while keeping the other variables at their mean values across the plots of observed species. Next, we calculated species‐specific ensemble response curves for each variable by averaging the response curves across the modelling techniques, with each curve weighted by the cross‐validated TSS value of the corresponding model (Hellegers et al. 2020). From the ensemble response curves, we retrieved the MIV for each environmental variable and each species as the variable value corresponding to the highest PoO. We computed their median in cases where multiple values were linked to the maximum occurrence probability. Finally, we performed a Spearman's rank correlation analysis between each pair of MIV‐EIV across the species, using the ‘corr.test’ function from the ‘psych’ package in R (v.2.3.9) (Revelle 2022; Wu et al. 2024).

## 3 Results

### 3.1 Cross‐Validation and Variable Importance

**FIGURE 1:**
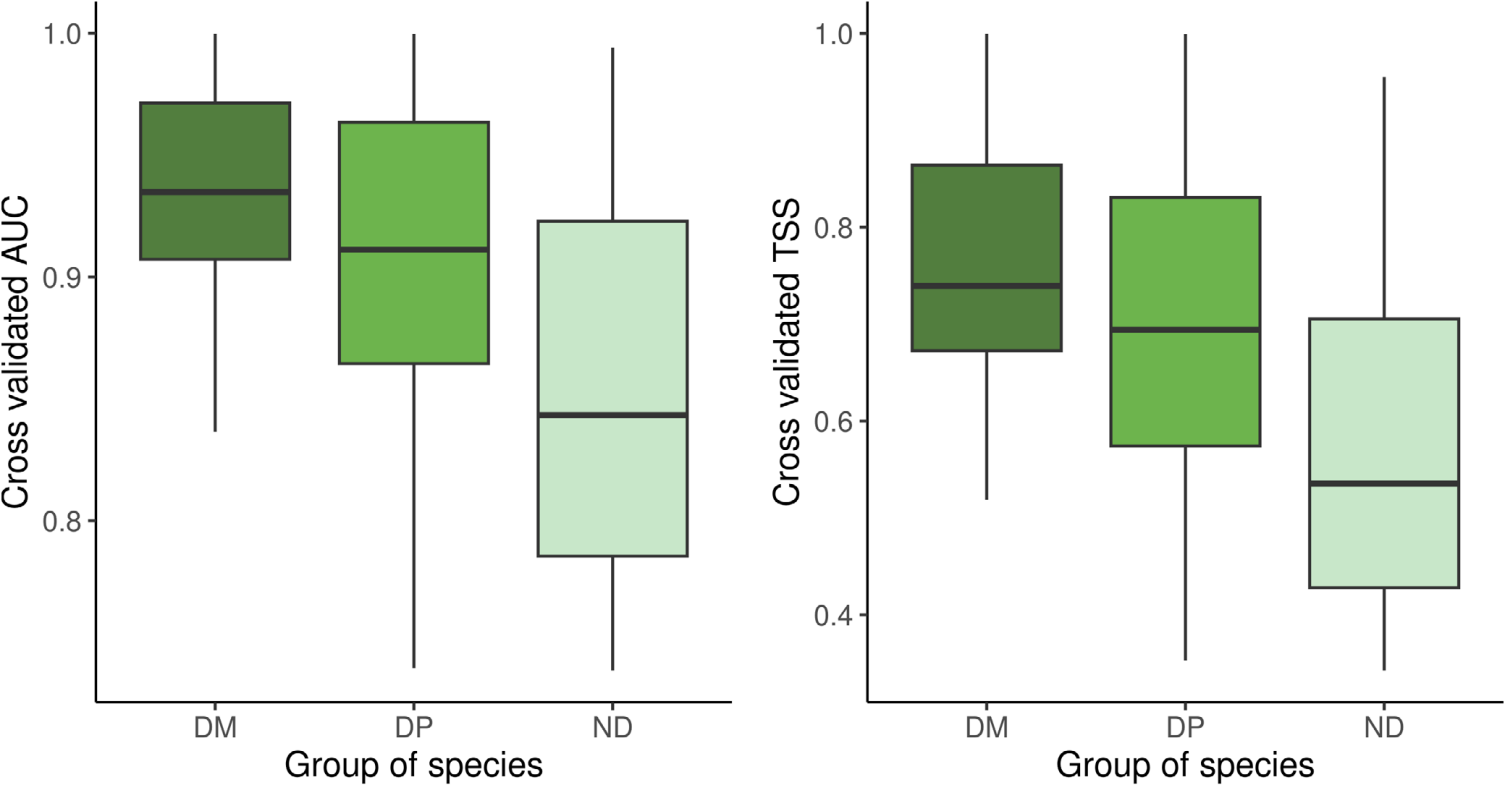
Boxplots of the cross‐validated AUC (area under the receiver operating characteristic curve) and TSS (true skill statistic) of the ensemble models fitted for diagnostic mosses (DM; *n* = 48), diagnostic vascular plants (DP; *n* = 159), and non‐diagnostic species (ND; *n* = 58). The boxes show the medians, the 25 and 75 percentiles, and the whiskers represent 1.5 times the interquartile distance.

**FIGURE 2:**
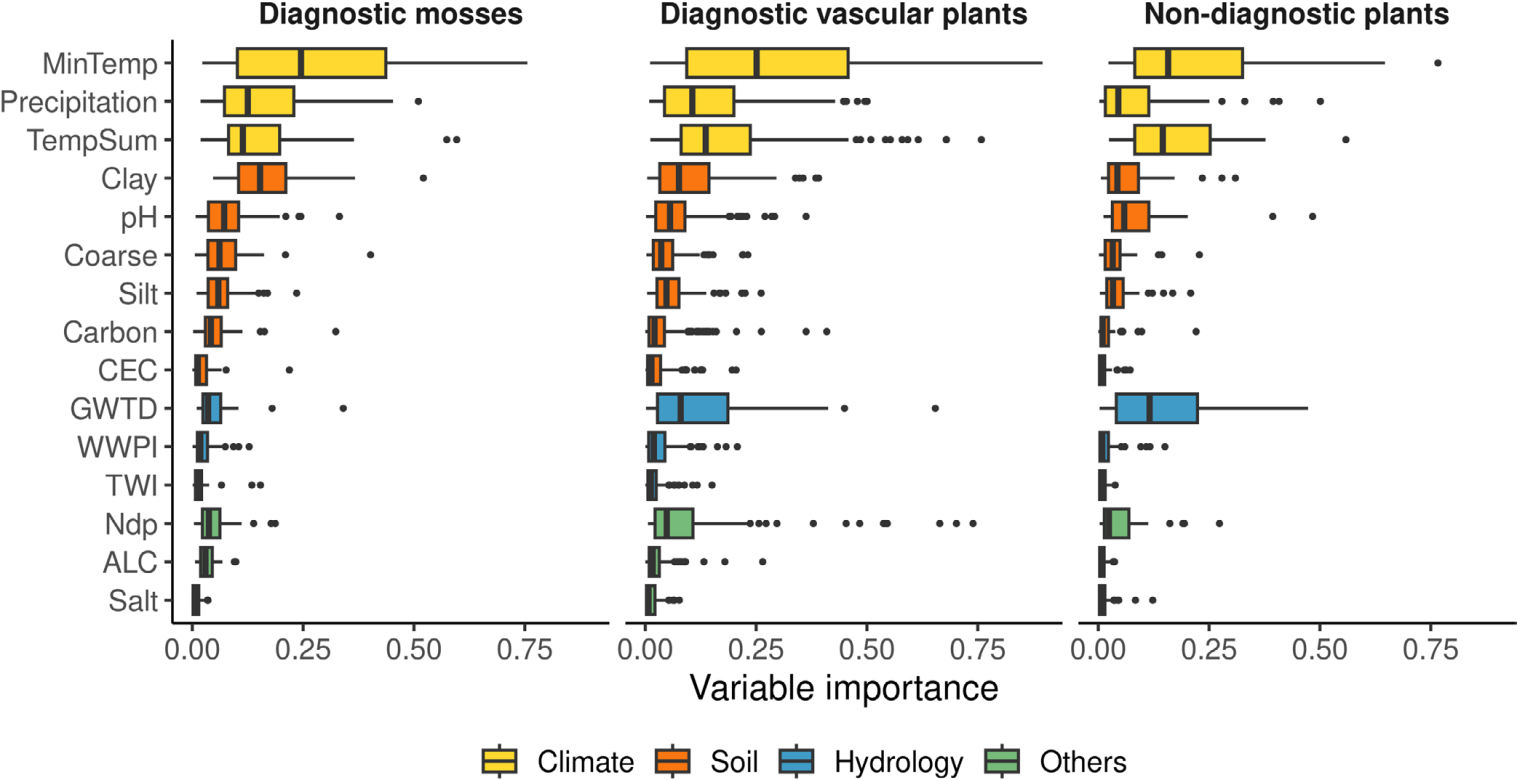
Variable importance of all variables used to fit the SDMs for the three species groups (diagnostic moss species, diagnostic vascular plant species, and non‐diagnostic species). The boxes show the medians, the 25 and 75 percentiles, and the whiskers represent 1.5 times the interquartile distance. The explanations of the variable abbreviations can be found in Table 1.

Based on the cross‐validation, the overall model performance was moderate to high; that is, for 97% of the modelled species, we obtained an ensemble model with AUC > 0.7 and TSS > 0.4 (Figure 1). We found the highest model performance for the diagnostic mosses, with median AUC and TSS values of 0.93 and 0.73 and ranges of 0.84–0.99 and 0.51–0.99, respectively (Figure 1; Table A3). The model performance for the diagnostic vascular plants was slightly lower (median AUC and TSS values of 0.91 and 0.69, respectively), while it was the lowest for the non‐diagnostic species (median AUC and TSS values of 0.84 and 0.53, respectively). For all three species groups and based on the median variable importance values, the most important environmental variable was the mean temperature of the coldest month (Figure 2; Table A2). The second most important variable for the diagnostic mosses was clay content, followed by annual growing degree days and precipitation, while GWTD was less important. For diagnostic vascular plants, annual growing degree days were the second most important variable, followed by precipitation, clay content, GWTD, and atmospheric nitrogen deposition. For the non‐diagnostic plants, annual growing degree days were the second most important variable, followed by GTWD.

### 3.2 Validation Against Independent Data

**FIGURE 3:**
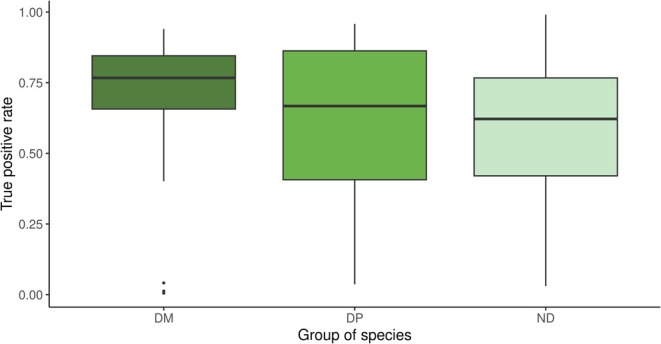
Proportion of GBIF observations correctly predicted by the SDMs (true positive rate; TPR) for diagnostic moss species (DM; *n* = 48), diagnostic vascular plant species (DP; *n* = 159), and non‐diagnostic species (ND; *n* = 58). The boxes show the medians, the 25 and 75 percentiles, and the whiskers represent 1.5 times the interquartile distance.

**FIGURE A2:**
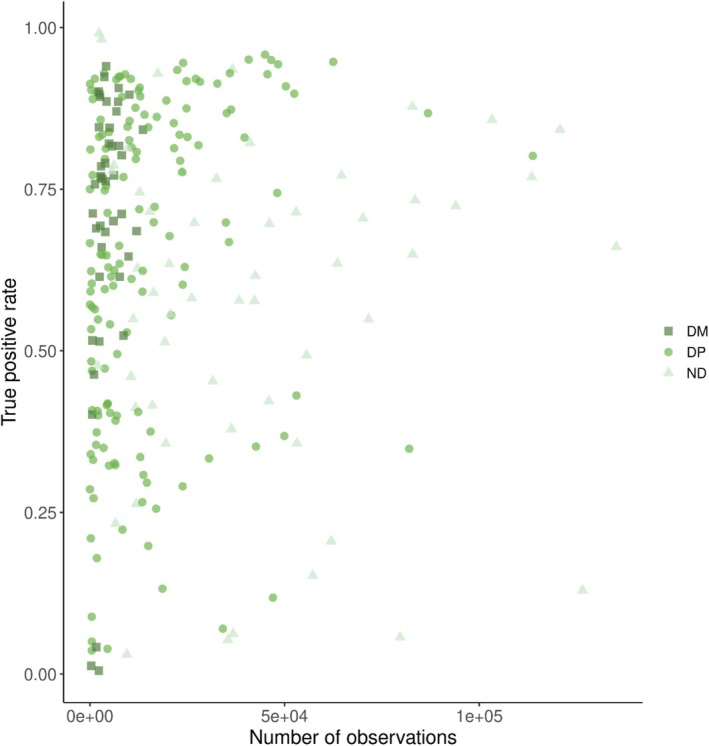
True positive rate in relation to the number of observations per species as obtained from GBIF (Global Biodiversity Information Facility) for three species groups: DM, diagnostic mosses; DV, diagnostic vascular plants; ND, non‐diagnostic plants.

The comparison of the SDM outputs with independent occurrence data revealed that models fitted for diagnostic mosses aligned most closely with observed presences, yielding a median TPR of 0.77; that is, 77% of the presences obtained from GBIF are correctly predicted. We found a TPR > 0.5 for 81% of the diagnostic mosses (Figure 3). For diagnostic vascular plants and non‐diagnostic plants, the median TPR was 0.67 and 0.62, respectively. Overall, 63% of the diagnostic vascular plants and 64% of the non‐diagnostic plants had SDMs with a TPR > 0.5. We found no relation between the TPR and the number of GBIF presences (Figure A2).

**FIGURE 4:**
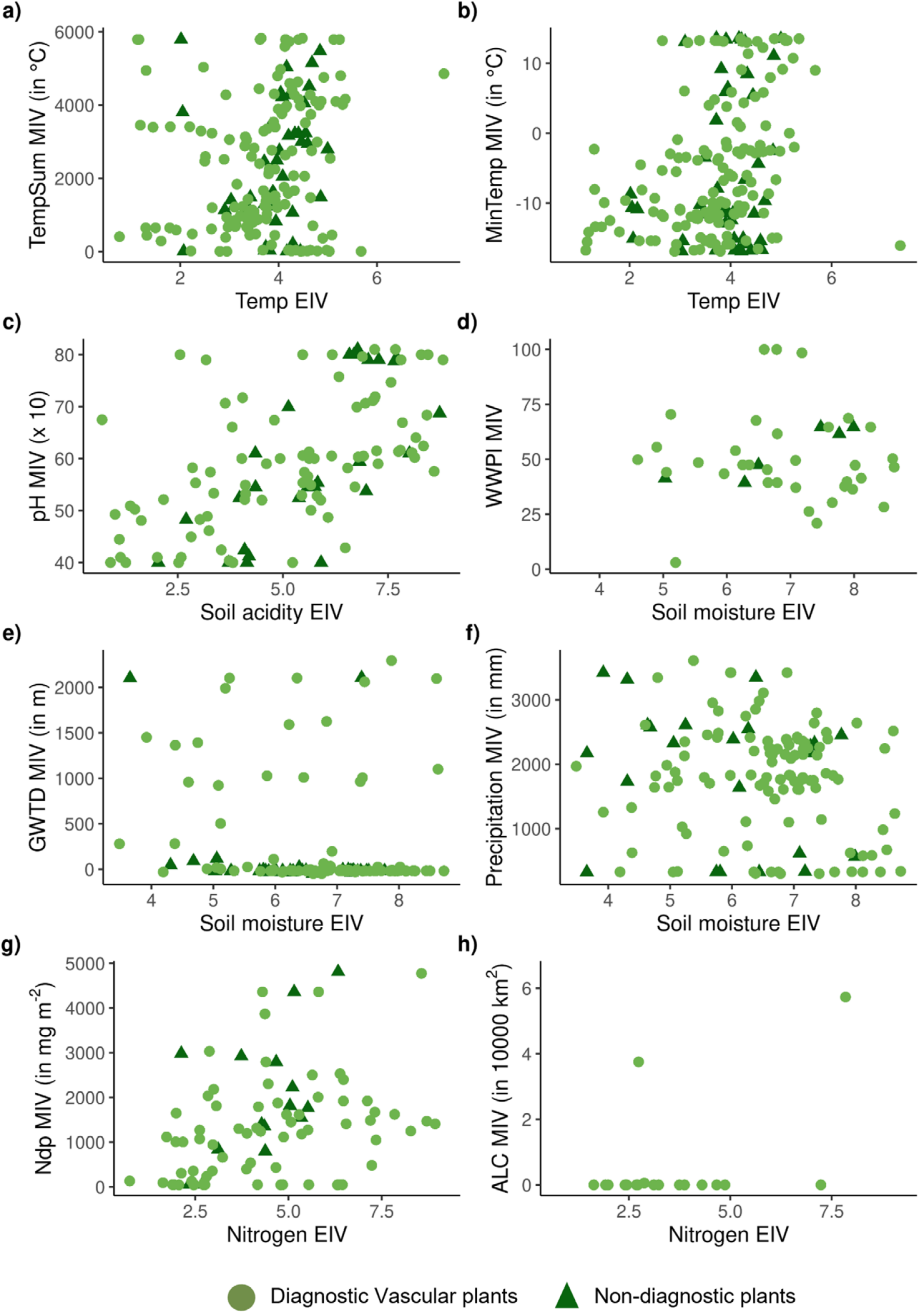
Modelled indicator values (MIV) in relation to ecological indicator values (EIV) for diagnostic and non‐diagnostic vascular plants. Corresponding correlation coefficients (Spearman's rho), *p*‐values, and number of species included per pairwise comparison can be found in Table 2. The explanation of the variable abbreviations can be found in Table 1.

We found weak primarily (0.1 ≤ rho ≤ 0.3) to moderate (0.3 ≤ rho ≤ 0.7) relationships between MIVs and EIVs of vascular plants (Figure 4; Table 2). We found the most substantial MIV‐EIV relationship for diagnostic vascular plants' soil pH–soil acidity pair. This was followed by the relationship between the MIV‐EIV pairs of mean temperature of the coldest month and temperature and atmospheric nitrogen deposition and nitrogen (Table 2). For non‐diagnostic vascular plants, we found the most substantial relationship for the MIV‐EIV pair of soil pH and soil acidity, followed by water and wetness probability index and soil moisture. The MIV‐EIV pair of nitrogen deposition and nitrogen had a moderate rho value (0.45), but it was not a significant relationship (*p* = 0.09).

**TABLE 2:** Spearman's rank correlations (rho) between ecological indicator values (EIVs) and modelled indicator values (MIVs) for diagnostic and non‐diagnostic vascular plants. *n* represents the number of species included in the analysis. Correlations of rho ≥ 0.3 with *p* < 0.05 are depicted in bold.

EIV	MIV	Species group					
		Diagnostic vascular plants			Non‐diagnostic vascular plants		
		rho	*p*	*n*	rho	*p*	*n*
Soil moisture	Precipitation	−0.10	0.29	109	−0.19	0.39	23
	Groundwater table depth	−0.21	0.03	101	−0.27	0.10	38
	Water and wetness probability index	−0.20	0.25	34	**0.84**	**0.03**	6
Nitrogen	Nitrogen deposition	**0.36**	**0.00**	75	0.45	0.09	15
	Anthropogenic land cover in upstream catchment area	0.21	0.39	20	NA	NA	NA
Soil acidity	pH	**0.61**	**< 0.001**	86	**0.71**	**< 0.001**	26
Temperature	Mean temperature of the coldest month	**0.42**	**< 0.001**	140	0.06	0.68	47
	Annual growing degree days	0.19	0.02	144	**0.33**	**0.02**	44

## 4 Discussion

In this study, we developed and validated SDMs for 265 vascular plant and moss species characteristic of wetland vegetation across Europe. These models were based on the framework established by Hellegers et al. (2020). We go beyond existing studies that have fitted SDMs for wetland species by including moss species and variables representative of the unique hydrological conditions of wetlands. The performance of the SDMs was generally moderate to high with cross‐validated AUC > 0.7 and cross‐validated TSS > 0.4 for 48 out of 48 diagnostic mosses, 158 out of 159 diagnostic vascular plants, and 52 out of 58 non‐diagnostic plants (Table A3). The SDMs for diagnostic mosses exhibited the highest performance both in cross‐validation and based on a comparison with independent observations from GBIF, followed by the diagnostic vascular plants and the non‐diagnostic species. This difference in performance may reflect that SDMs, in general, are likely to have more discriminatory power for specialist species than for generalists, as the distributions of the former are more tightly bound to specific environmental conditions (Morelli et al. 2024). Mosses, for example, are especially prevalent in relatively cold and wet environments because, unlike vascular plants, they are unable to regulate their internal water content (Mohanasundaram and Pandey 2022). Consequently, temperature and precipitation are important determinants of the occurrence of moss species at a European scale (Figure 2); hence, SDMs, including these environmental variables, can be expected to perform well.

Despite the crucial role of moisture for moss species (Choi et al. 2019; Riihimäki et al. 2021), we found that none of the hydrological variables, namely, groundwater table depth (GWTD), inundation probability (WWPI), and moisture accumulation (TWI), were important predictors of occurrence in the SDMs of diagnostic moss species (Figure 2). Most mosses absorb water and nutrients directly through their surfaces and depend highly on moisture from their immediate environment (Hodgetts et al. 2019). Despite this dependency, mosses have developed adaptations that enable them to endure complete desiccation (Zhou et al. 2021). They can suspend physiological activities during droughts, enabling them to survive without water for extended periods. When rehydration occurs, mosses can quickly reactivate their metabolic processes and resume growth (Hodgetts et al. 2019; Zhou et al. 2021). While groundwater can provide essential moisture when water tables are high, a drop in the groundwater table leaves precipitation as the primary water source (Liu et al. 2005; Utstøl‐Klein et al. 2015; Zhong et al. 2020; Zhou et al. 2021). Recent studies have demonstrated that even minimal precipitation (< 1 mm) can significantly increase water content and boost the productivity of moss species like *Sphagnum*, underscoring the importance of precipitation for moss survival (Bengtsson et al. 2021; Nijp et al. 2014; Thompson and Waddington 2008).

Compared to mosses, groundwater table depth was a more important variable for both diagnostic vascular plants and non‐diagnostic wetland plants (Figure 2) likely due to their ability to extend roots below the water table (Carlson Mazur et al. 2020). However, we found the other two hydrological variables of limited importance for vascular plants. Although inundation probability is generally a good indicator of potential wetland areas (Ludwig et al. 2019), WWPI was not a significant predictor in the SDMs for wetland vascular plants in our analyses. Further, we observed a strong relationship between modelled and empirical niche optima for soil moisture only for the relatively small subset of non‐diagnostic species. The rho‐values from the correlation analyses between all three hydrological variables and soil moisture were low for other species. Climatic variables emerged as the most important predictors for the European distributions of wetland vascular plants and mosses. This aligns with findings from other studies on wetland plant distributions (Cerrejón et al. 2020; Dang et al. 2021; Samal et al. 2022). Furthermore, our results highlight the importance of soil pH, for which modelled niche optima correlated strongly with ecological indicator values for soil acidity. The important role of soil properties, such as pH, in determining wetland species distributions is also confirmed by previous research (Clough 2014; Dang et al. 2021; Hossain and Nuruddin 2016).

In our study, we found nitrogen deposition to be a relatively important factor for predicting the occurrence of vascular plants. Nitrogen deposition can alter plant competition dynamics, particularly by enhancing the ability of certain species to compete for light and by disrupting physiological processes through soil acidification (Midolo et al. 2019). It can have direct toxic impacts on plants and increase their vulnerability to secondary stress and disturbance (Yuan et al. 2020). Despite the importance of nitrogen, we found only relatively weak relationships between the modelled and empirical niche optima for nitrogen deposition and nitrogen. This suggests that other variables, such as soil characteristics, properties of the surrounding land use, and management practices, also influence the soil nitrogen content (Kooijman et al. 1998; Nissinen and Hari 1998). Moreover, nitrogen availability in wetlands, especially peatlands, is reduced due to limited decomposition rates.

Anthropogenic land cover in the upstream catchment area (ALC) did not emerge as a significant predictor for the occurrence of wetland species in our study despite studies showing the impact of neighbouring land use on wetland conditions and species richness (Houlahan et al. 2006; Im et al. 2020; Stapanian et al. 2018; Vörösmarty et al. 2010). Our findings suggest that the ALC variable used here may not adequately capture the flow of nutrients and pollutants from upstream land to wetlands, highlighting an important area for further detailed field research. Moreover, the resolution of the ALC variable may have been too coarse to reflect finer‐scale land use impacts (Houlahan et al. 2006). Including more refined land use variables in SDMs, specifically variables representing land use and intensity in areas directly adjacent to wetlands, may offer deeper insights into how surrounding land use influences wetland plant species distributions.

Despite the reasonable to excellent performance of our SDMs, we acknowledge that our study has several limitations. Although hydrology plays a crucial role in wetland ecosystems, the selected hydrological variables had a relatively modest impact on the predictions. The relatively low predictive power of the hydrological variables in our SDMs might be attributed to the fine‐scale spatial and temporal heterogeneity of hydrological conditions in wetlands, which can be challenging to capture in large‐scale SDMs (Hellegers et al. 2020). For example, the global groundwater table depth map from Fan et al. (2013) does not incorporate groundwater dynamics or fine‐grain spatial variability in surface elevation, making it challenging to capture fine‐grain heterogeneity in groundwater depth. Using region‐specific and more detailed hydrological databases with a better representation of fine‐scale spatial and temporal hydrological conditions may improve predictive power. Further, our assessment of the ecological relevance of the modelled responses yielded promising results for some environmental variables but not for others. While some pairs of modelled indicator values (MIV) and ecological indicator values (EIV) demonstrated strong relationships—such as the correlation between EIVE1.0 temperature values and mean temperature of the coldest month from CHELSA (version 2.1), similar to findings from Dengler et al. (2023)—others exhibited weaker relations (e.g., atmospheric nitrogen deposition and soil nitrogen EIV). We acknowledge that comparing the modelled and empirical indicator values is associated with uncertainties. EIVs are ordinal scores that describe a plant species' environmental preferences concerning specific conditions. However, these values do not necessarily reflect a species' physiological optimum for the variable of concern but rather its realised niche, which persists under competition (Bartelheimer and Poschlod 2016; Kermavnar et al. 2023). Moreover, the EIV values obtained from the EIVE1.0 database originate from various sources, regions, and times. This may result in heterogeneity in the EIV data and a potential mismatch with the environmental data used in our SDMs. Further, there might be intrinsic differences between the variables represented by the EIVs and those underlying our MIVs. For example, Schaffers and Sýkora (2000) found that EIVs of soil moisture correlate most strongly with the lowest moisture content during summer. Hence, EIVs may have stronger associations with some specific environmental conditions than others, meaning EIVs may represent specific conditions in the environment that the MIVs might not capture. Finally, our method of calculating the MIV focuses solely on the specific environmental conditions corresponding to the modelled niche optima of species without accounting for other niche‐related factors, such as niche breadth or the potential of multiple optima per species. Future studies could refine our validation approach by incorporating additional relevant aspects, including niche width and multiple favourable conditions.

Our study demonstrates that the SDMs fitted here successfully capture the large‐scale potential distributions of wetland vascular plants and mosses, with climatic variables emerging as key predictors. This underscores the potential of our models to assess how wetland biodiversity might respond to future climate change, offering valuable insights for informing large‐scale climate change mitigation strategies. While the selected hydrological and anthropogenic variables showed weaker predictive power, our models performed well in predicting the potential distribution of species diagnostic of wetlands, as validated by independent datasets. The results of the variable importance analysis and the relatively weak relationships in the MIV–EIV analysis suggest opportunities for refinement. Importantly, our study highlights the need to explore alternative variables that better reflect local conditions, particularly those related to hydrology and anthropogenic pressures or to adopt a different model structure. A hierarchical approach, where coarse‐grain and fine‐grain environmental predictors are used in subsequent modelling steps, might be better able to capture fine‐grain heterogeneity in hydrology and land cover (Mateo et al. 2019). These improvements will further enhance the accuracy of wetland SDMs, ultimately contributing to more robust tools for wetland conservation and management.

## 5 Conclusions

Wetlands and their biodiversity face significant threats from drainage, agricultural conversion, and climate change, necessitating effective conservation measures. We developed and validated SDMs for wetland vegetation across Europe, including vascular plants and moss species, which are often overlooked in existing wetland models. The SDMs performed well in predicting large‐scale distribution patterns, particularly for diagnostic mosses, with strong cross‐validation results and alignment with GBIF occurrence data. Climate variables, especially the mean temperature of the coldest month, emerged as the most important predictors of species occurrence. The weak correlation between the modelled niche optima of the hydrological variables and the empirical niche optima of soil moisture indicates a need for an improved representation of local hydrological conditions in the models. Addressing the gaps can further enhance the robustness and utility of wetland SDMs for guiding wetland conservation and restoration efforts, for example, in the context of international conventions such as the Ramsar Convention and the Convention on Biological Diversity (CBD).

## Author Contributions

**Ojaswi Sumbh:** conceptualization (lead), data curation (lead), formal analysis (lead), investigation (lead), methodology (lead), project administration (supporting), software (supporting), validation (lead), visualization (lead), writing – original draft (lead), writing – review and editing (lead). **Marjon Hellegers:** conceptualization (lead), data curation (lead), formal analysis (lead), funding acquisition (equal), investigation (equal), methodology (equal), project administration (equal), software (lead), supervision (equal), writing – review and editing (equal). **Valerio Barbarossa:** data curation (supporting), writing – review and editing (supporting). **Renata Ćušterevska:** data curation (supporting), writing – review and editing (supporting). **Borja Jiménez‐Alfaro:** data curation (supporting), writing – review and editing (supporting). **Łukasz Kozub:** data curation (supporting), writing – review and editing (supporting). **Francesca Napoleone:** data curation (supporting), writing – review and editing (supporting). **Zvjezdana Stančić:** data curation (supporting), writing – review and editing (supporting). **Aafke M. Schipper:** conceptualization (lead), data curation (equal), formal analysis (equal), funding acquisition (lead), investigation (equal), methodology (equal), project administration (lead), supervision (lead), validation (supporting), visualization (supporting), writing – review and editing (equal).

## Conflicts of Interest

The authors declare no conflicts of interest.

## Supporting information


Data S1.

## Data Availability

The species observations in the vegetation plots used for this analysis are available via the European Vegetation Archive (https://euroveg.org/eva‐database). The climate, soil, and topographic variables from CHELSA (Karger et al. 2017), SoilGrids (Hengl et al. 2017), and Marthews et al. (2015), respectively, are publicly available. Data on land use and land change is publicly available via the German Environmental Agency (https://www.umweltbundesamt.de/en/publikationen/creation‐of‐a‐harmonized‐land‐cover‐map‐as‐an).

## Appendix A

**TABLE A1:** List of wetland species for which species distribution models were fitted in this study.

**Diagnostic mosses**		
*Aneura pinguis*	*Odontoschisma sphagni*	*Sphagnum fuscum*
*Aulacomnium palustre*	*Paludella squarrosa*	*Sphagnum magellanicum*
*Breutelia chrysocoma*	*Palustriella decipiens*	*Sphagnum papillosum*
*Bryum pseudotriquetrum*	*Philonotis calcarea*	*Sphagnum platyphyllum*
*Calliergon giganteum*	*Philonotis fontana*	*Sphagnum rubellum*
*Calypogeia muelleriana*	*Philonotis seriata*	*Sphagnum russowii*
*Campylium stellatum*	*Polytrichum commune*	*Sphagnum subnitens*
*Campylopus flexuosus*	*Polytrichum strictum*	*Sphagnum subsecundum*
*Cephalozia bicuspidata*	*Pseudocalliergon trifarium*	*Sphagnum tenellum*
*Cephalozia connivens*	*Racomitrium lanuginosum*	*Sphagnum teres*
*Dicranum elongatum*	*Scapania irrigua*	*Sphagnum warnstorfii*
*Diplophyllum albicans*	*Scapania undulata*	*Sphenolobus minutus*
*Fissidens adianthoides*	*Scorpidium scorpioides*	*Straminergon stramineum*
*Hamatocaulis vernicosus*	*Sphagnum compactum*	*Tomentypnum nitens*
*Mylia anomala*	*Sphagnum contortum*	*Warnstorfia exannulata*
*Mylia taylorii*	*Sphagnum cuspidatum*	*Warnstorfia fluitans*
**Diagnostic vascular plants**		
*Acorus calamus*	*Eleocharis ovata*	*Phalaroides arundinacea*
*Agrostis canina*	*Eleocharis palustris*	*Pinguicula alpina*
*Alchemilla glabra*	*Eleocharis quinqueflora*	*Pinguicula balcanica*
*Allium schoenoprasum*	*Epipactis palustris*	*Pinguicula vulgaris*
*Alopecurus aequalis*	*Equisetum fluviatile*	*Plantago maritima*
*Andromeda polifolia*	*Equisetum palustre*	*Polygala amarella*
*Arctostaphylos alpinus*	*Equisetum variegatum*	*Polygala serpyllifolia*
*Bartsia alpina*	*Erica cinerea*	*Polygonum aviculare*
*Bellis annua*	*Erica tetralix*	*Potamogeton polygonifolius*
*Berula erecta*	*Eriophorum angustifolium*	*Potentilla erecta*
*Bistorta vivipara*	*Eriophorum latifolium*	*Potentilla supina*
*Blysmus compressus*	*Eriophorum vaginatum*	*Primula farinosa*
*Bruckenthalia spiculifolia*	*Festuca rubra*	*Radiola linoides*
*Callitriche palustris*	*Galium uliginosum*	*Ranunculus sceleratus*
*Calluna vulgaris*	*Gentiana pyrenaica*	*Rhododendron tomentosum*
*Carex acuta*	*Gentianella bulgarica*	*Rhynchospora alba*
*Carex bohemica*	*Geum coccineum*	*Rorippa amphibia*
*Carex canescens*	*Glyceria declinata*	*Rorippa palustris*
*Carex capillaris*	*Glyceria maxima*	*Rubus chamaemorus*
*Carex chordorrhiza*	*Gnaphalium uliginosum*	*Rumex hydrolapathum*
*Carex davalliana*	*Illecebrum verticillatum*	*Rumex maritimus*
*Carex diandra*	*Isolepis cernua*	*Sagittaria sagittifolia*
*Carex dioica*	*Isolepis setacea*	*Salix reticulata*
*Carex distans*	*Juncus alpinoarticulatus*	*Saxifraga aizoides*
*Carex disticha*	*Juncus bulbosus*	*Saxifraga stellaris*
*Carex echinata*	*Juncus capitatus*	*Scheuchzeria palustris*
*Carex flava*	*Juncus filiformis*	*Schoenoplectus lacustris*
*Carex frigida*	*Juncus subnodulosus*	*Schoenoplectus lacustris glaucus*
*Carex hostiana*	*Lemna minor*	*Schoenus ferrugineus*
*Carex lasiocarpa*	*Ligusticum mutellina*	*Schoenus nigricans*
*Carex lepidocarpa*	*Limosella aquatica*	*Selaginella selaginoides*
*Carex limosa*	*Lipandra polysperma*	*Sesleria comosa*
*Carex nigra*	*Lythrum hyssopifolia*	*Sparganium emersum*
*Carex panicea*	*Lythrum portula*	*Spergularia rubra*
*Carex pauciflora*	*Lythrum salicaria*	*Succisa pratensis*
*Carex riparia*	*Mentha pulegium*	*Taraxacum Weber*
*Carex rostrata*	*Menyanthes trifoliata*	*Taraxacum apenninum*
*Carex vesicaria*	*Myosotis scorpioides*	*Thalictrum alpinum*
*Cirsium appendiculatum*	*Myosoton aquaticum*	*Tofieldia calyculata*
*Cladium mariscus*	*Nardus stricta*	*Trichophorum alpinum*
*Coleanthus subtilis*	*Narthecium ossifragum*	*Trichophorum cespitosum*
*Comarum palustre*	*Oenanthe aquatica*	*Triglochin maritima*
*Corrigiola litoralis*	*Oxybasis glauca*	*Triglochin palustris*
*Cyperus fuscus*	*Oxybasis rubra*	*Typha latifolia*
*Cyperus michelianus*	*Parnassia palustris*	*Utricularia intermedia*
*Dactylorhiza cordigera*	*Pedicularis oederi*	*Utricularia minor*
*Dactylorhiza incarnata*	*Pedicularis palustris*	*Vaccinium microcarpum*
*Dactylorhiza majalis*	*Pedicularis sylvatica*	*Vaccinium oxycoccos*
*Drosera longifolia*	*Persicaria amphibia*	*Vaccinium uliginosum*
*Drosera rotundifolia*	*Persicaria dubia*	*Valeriana dioica*
*Elatine hydropiper*	*Persicaria hydropiper*	*Veratrum lobelianum*
*Elatine triandra*	*Persicaria lapathifolia*	*Viola palustris*
*Eleocharis acicularis*	*Peucedanum palustre*	*Willemetia stipitata*
**Non‐diagnostic plants**		
*Achillea millefolium*	*Chenopodium album*	*Lysimachia vulgaris*
*Agrostis stolonifera*	*Cirsium palustre*	*Mentha aquatica*
*Angelica sylvestris*	*Crepis paludosa*	*Molinia caerulea*
*Anthoxanthum odoratum*	*Ctenidium molluscum*	*Myrica gale*
*Atriplex prostrata*	*Dicranum scoparium*	*Persicaria maculosa*
*Bellidiastrum michelii*	*Epilobium palustre*	*Phragmites australis*
*Betula pubescens*	*Filipendula ulmaria*	*Pinus sylvestris*
*Briza media*	*Frangula alnus*	*Plagiomnium affine*
*Calamagrostis canescens*	*Galium palustre*	*Pleurozium schreberi*
*Calliergonella cuspidata*	*Glaux maritima*	*Poa alpina*
*Caltha palustris*	*Iris pseudacorus*	*Ptilidium ciliare*
*Calystegia sepium*	*Juncus bufonius*	*Ranunculus flammula*
*Cardamine pratensis*	*Juncus effusus*	*Ranunculus repens*
*Carex L*	*Juncus squarrosus*	*Rorippa sylvestris*
*Carex acutiformis*	*Leontodon hispidus*	*Salix repens*
*Carex elata*	*Leucobryum glaucum*	*Sesleria caerulea*
*Carex flacca*	*Linum catharticum*	*Solanum dulcamara*
*Carex paniculata*	*Lotus tenuis*	*Typha angustifolia*
*Carex sempervirens*	*Lycopus europaeus*	*Vaccinium myrtillus*
*Carex vaginata*		

**TABLE A2:** Number of modelled species per environmental variable with an importance value of > 0.05. The explanations of abbreviations for the variables can be found in Table 1. DM, diagnostic mosses; DV, diagnostic vascular plants; ND, non‐diagnostic plants.

Variable	Species group		
	DM	DP	ND
Precipitation	48	159	53
MinTemp	48	159	58
TempSum	48	159	58
Clay	48	158	58
Silt	48	158	57
CEC	42	121	38
pH	48	156	58
Carbon	46	137	45
Coarse	48	152	55
Salt	25	96	35
Ndp	47	159	54
TWI	44	126	38
GWTD	48	154	57
WWPI	44	128	39
ALC	48	139	37
Total number of species modelled	48	159	58

**TABLE A3:** Distributions of area under the receiver operating characteristic curve (AUC), true skill statistic (TSS), and true positive rate based on comparison with GBIF data (TPR) of the fitted SDMs for 265 species across three species groups. Numbers of species per AUC or TSS range are also provided.

	Species group		
	Diagnostic mosses	Diagnostic vascular plants	Non‐diagnostic species
AUC			
Minimum	0.84	0.74	0.73
25th percentile	0.90	0.86	0.78
Median	0.93	0.91	0.84
75th percentile	0.97	0.96	0.92
Maximum	0.99	0.99	0.99
Number of species			
< 0.3	0	0	0
0.3–0.7	0	0	0
> 0.7	48	159	58
TSS			
Minimum	0.51	0.35	0.34
25th percentile	0.67	0.57	0.42
Median	0.73	0.69	0.53
75th percentile	0.86	0.83	0.70
Maximum	0.99	0.99	0.95
Number of species			
< 0.4	0	1	6
0.4–0.6	6	49	32
> 0.6	42	109	20
TPR			
Minimum	0.005	0.03	0.03
25th percentile	0.65	0.40	0.42
Median	0.76	0.66	0.62
75th percentile	0.84	0.82	0.76
Maximum	0.94	0.95	0.99
