# Supplementary material for: Developing and Validating Species Distribution Models for Wetland Plants Across Europe
Source: Ecol Evol. 2025 Apr 23;15(4):e71157. doi: 10.1002/ece3.71157 (PMC12015742; doi:10.1002/ece3.71157)
Supplement: Supplementary file 1 — Data S1. [file ECE3-15-e71157-s001.pdf]

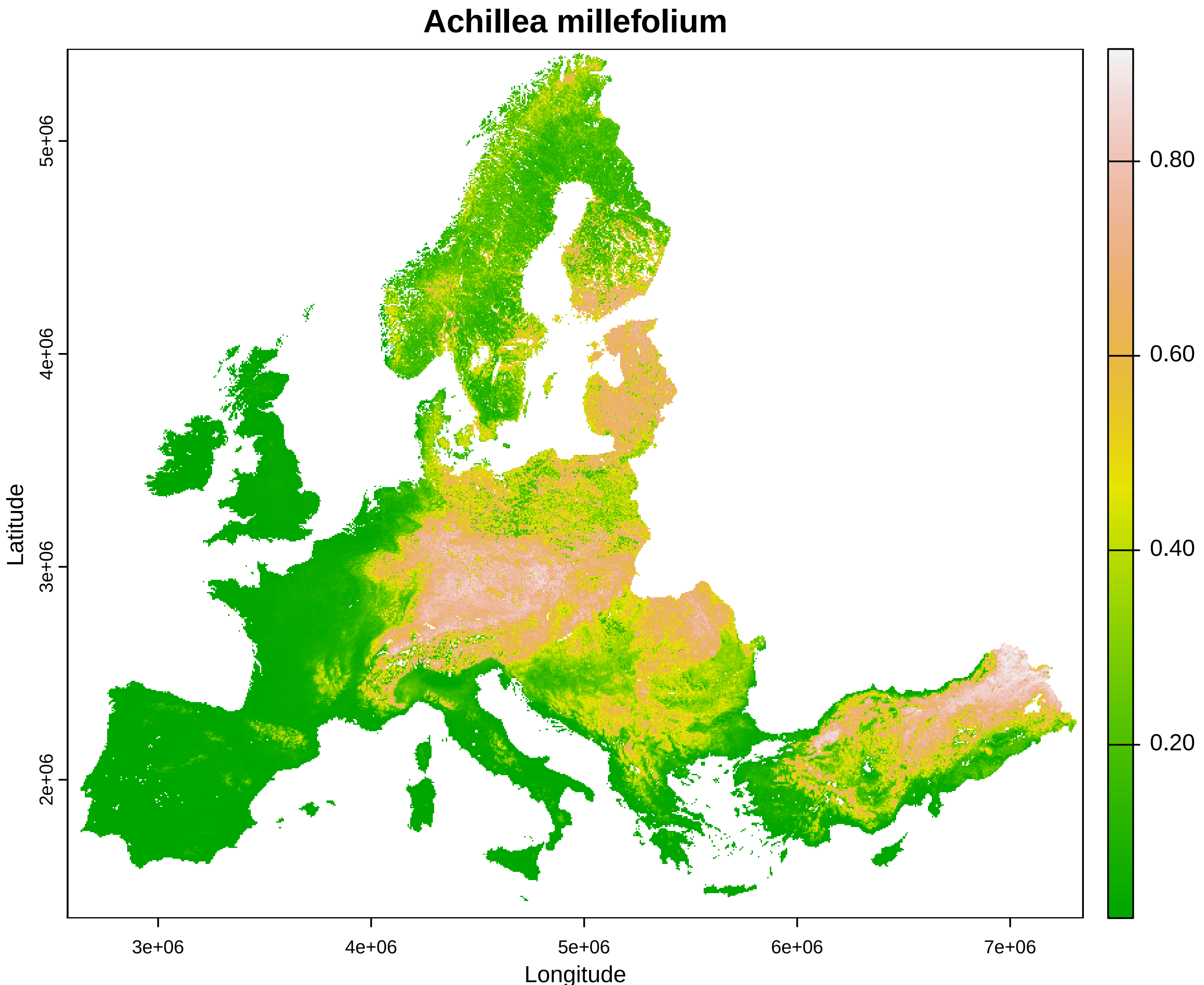
Achillea millefolium
Latitude
5e+06
4e+06
3e+06
2e+06
Longitude
3e+06
4e+06
5e+06
6e+06
7e+06
0.80
0.60
0.40
0.20

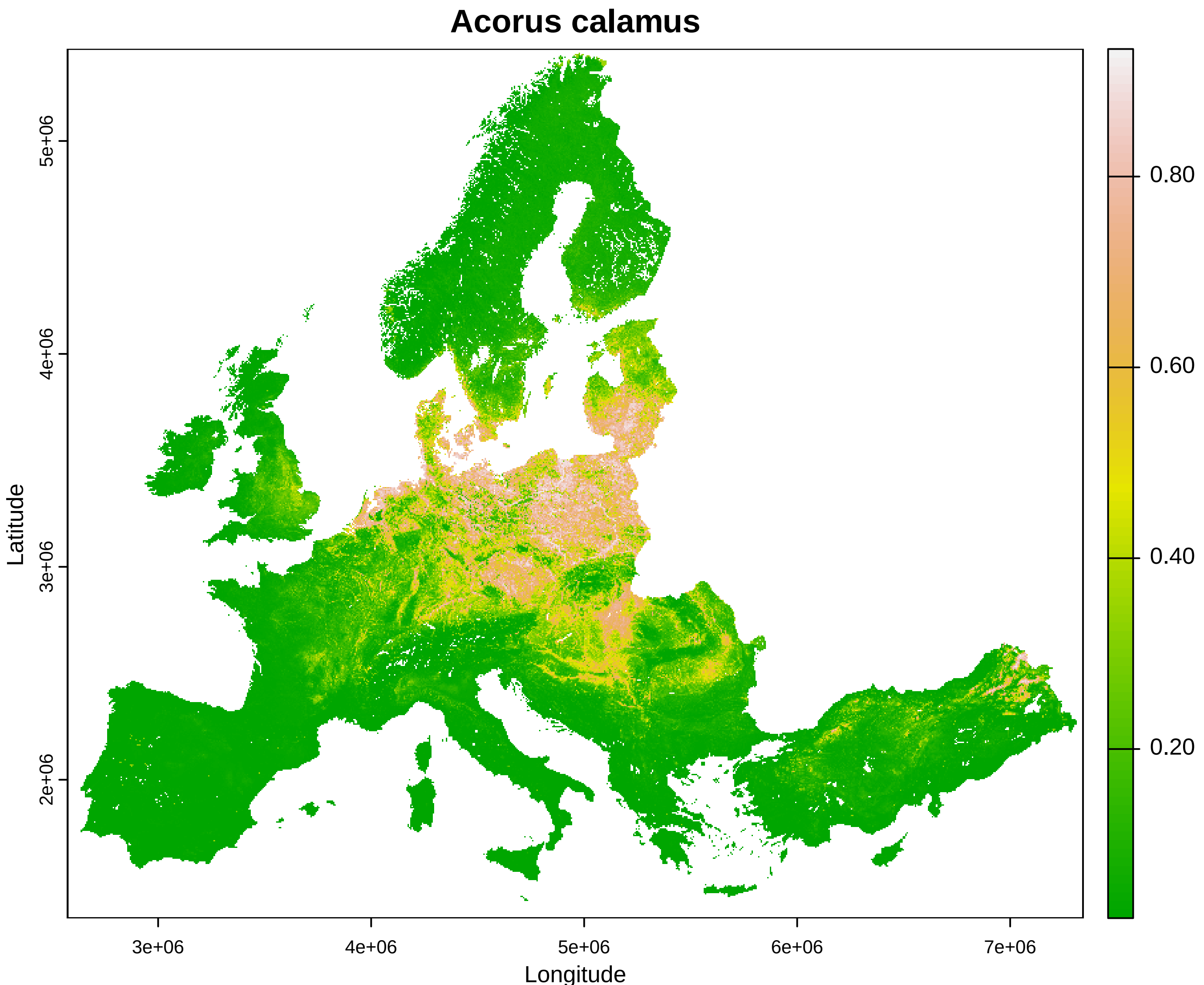
Acorus calamus
Latitude
5e+06
4e+06
3e+06
2e+06
3e+06
4e+06
5e+06
6e+06
7e+06
Longitude
0.80
0.60
0.40
0.20

Agrostis canina

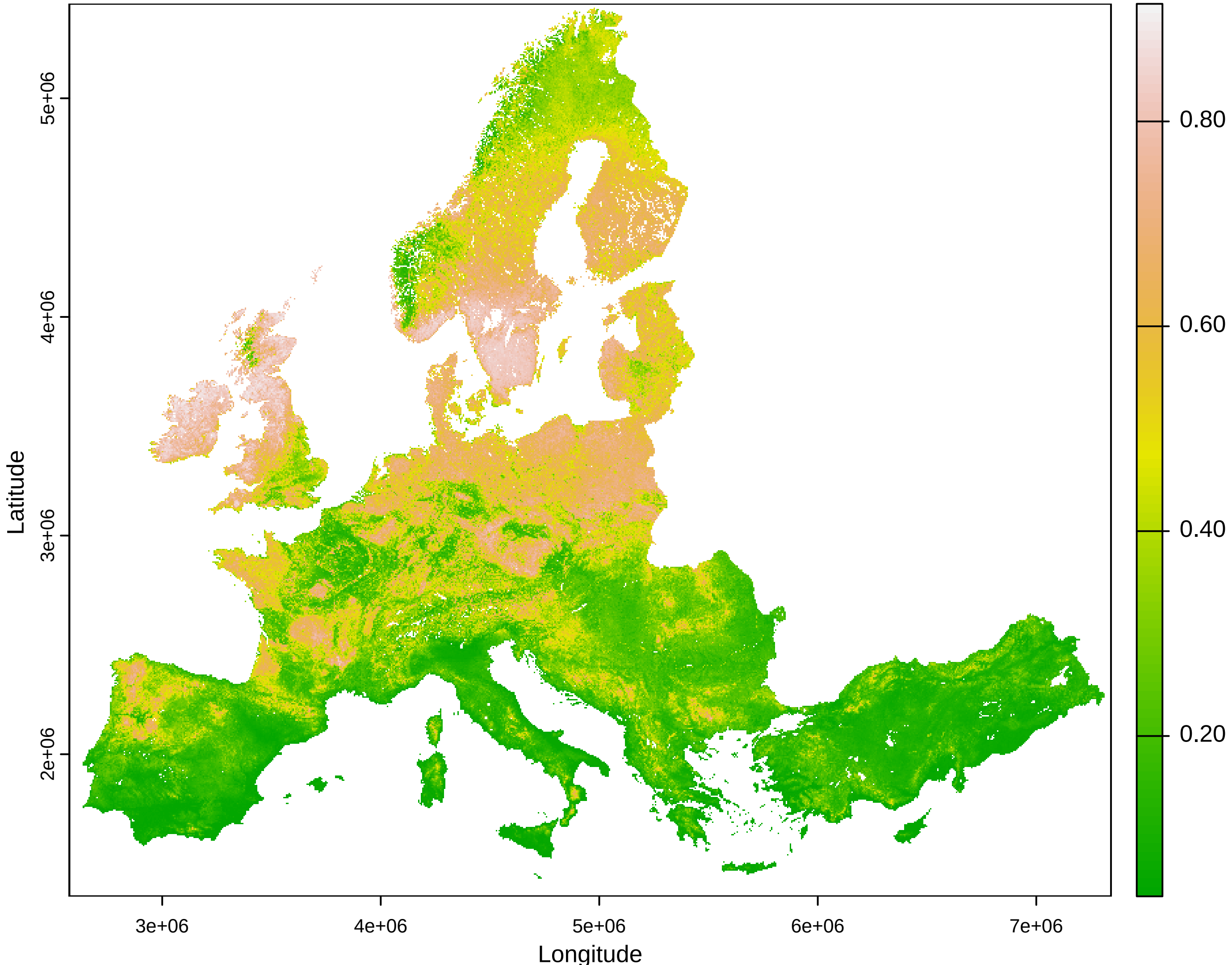

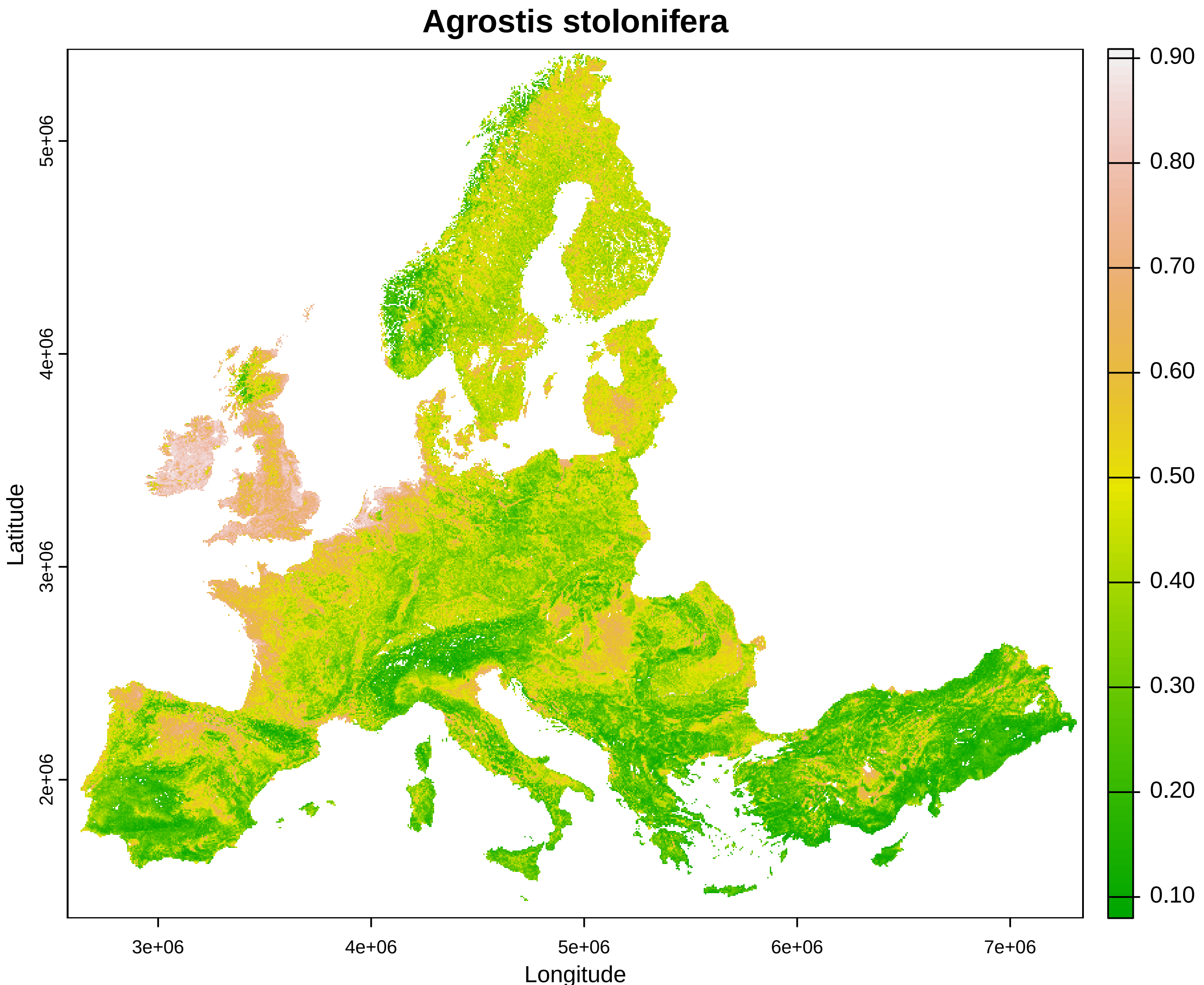

Agrostis stolonifera
Latitude
Longitude
5e+06
4e+06
3e+06
2e+06
3e+06
4e+06
5e+06
6e+06
7e+06
0.90
0.80
0.70
0.60
0.50
0.40
0.30
0.20
0.10

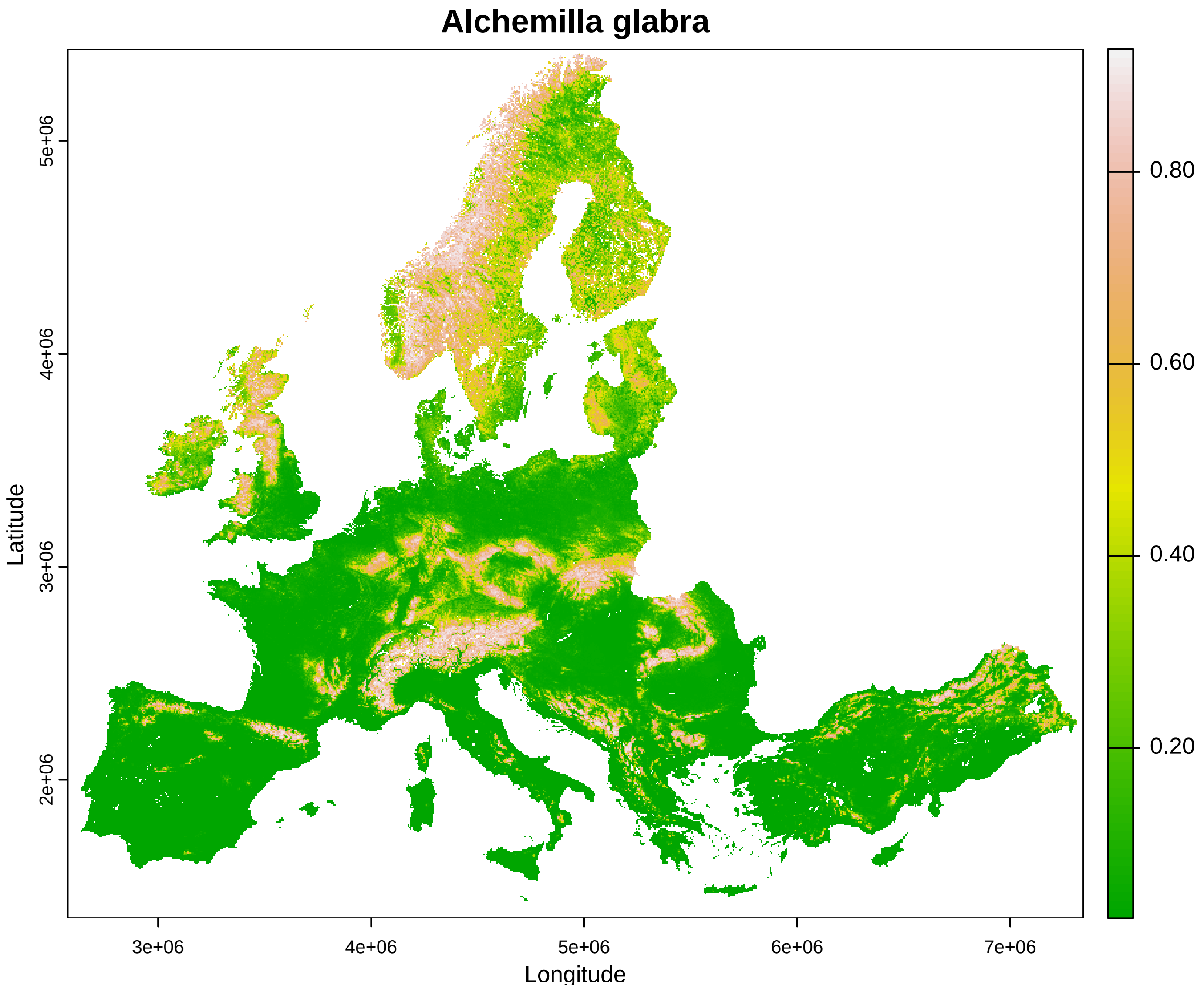
Alchemilla glabra
Latitude
5e+06
4e+06
3e+06
2e+06
Longitude
3e+06
4e+06
5e+06
6e+06
7e+06
0.80
0.60
0.40
0.20

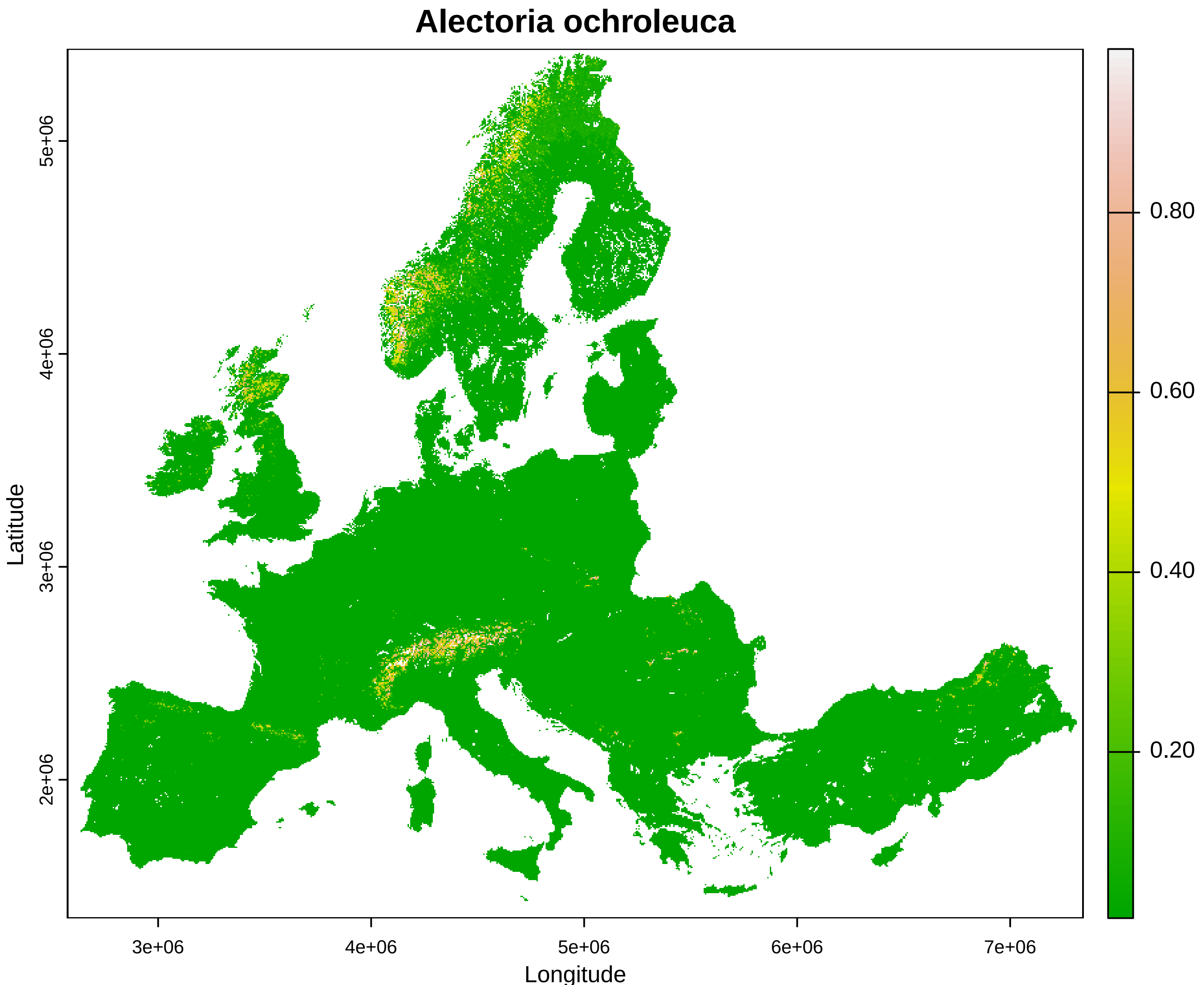
Alectoria ochroleuca
Latitude
Longitude
5e+06
4e+06
3e+06
2e+06
3e+06
4e+06
5e+06
6e+06
7e+06
0.80
0.60
0.40
0.20

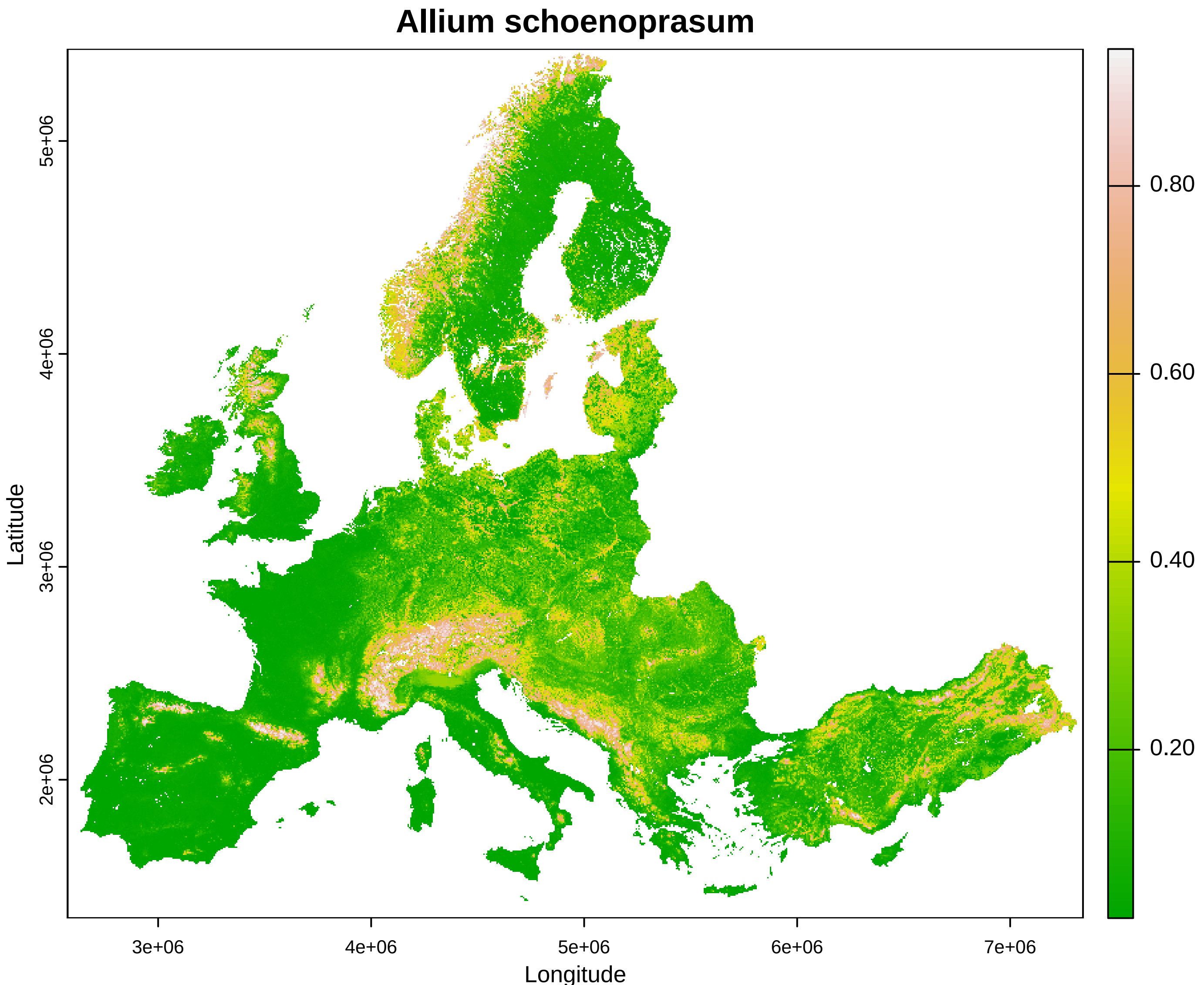

Allium schoenoprasum
Latitude
5e+06
4e+06
3e+06
2e+06
3e+06
4e+06
5e+06
6e+06
7e+06
Longitude
0.80
0.60
0.40
0.20

Alopecurus aequalis

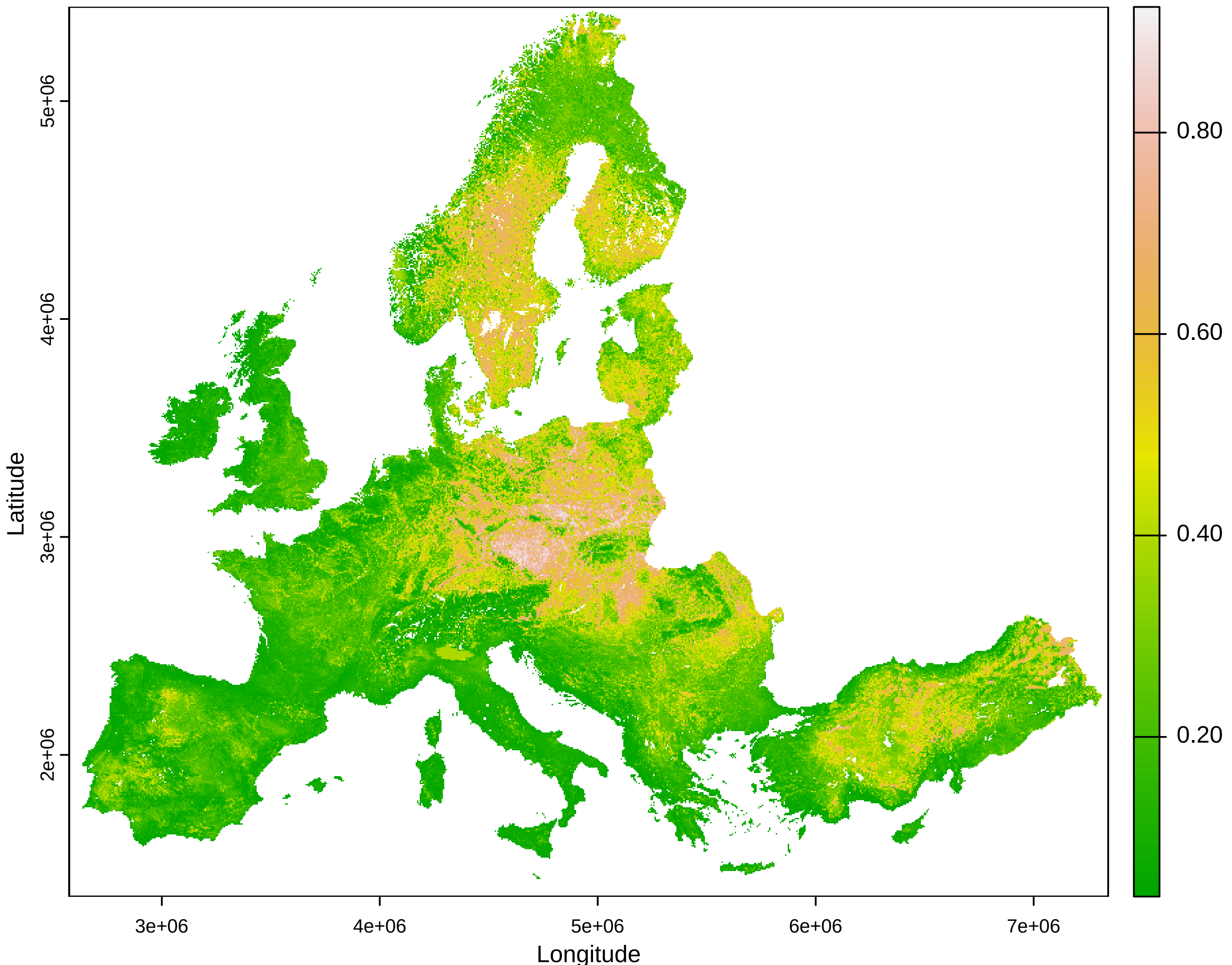

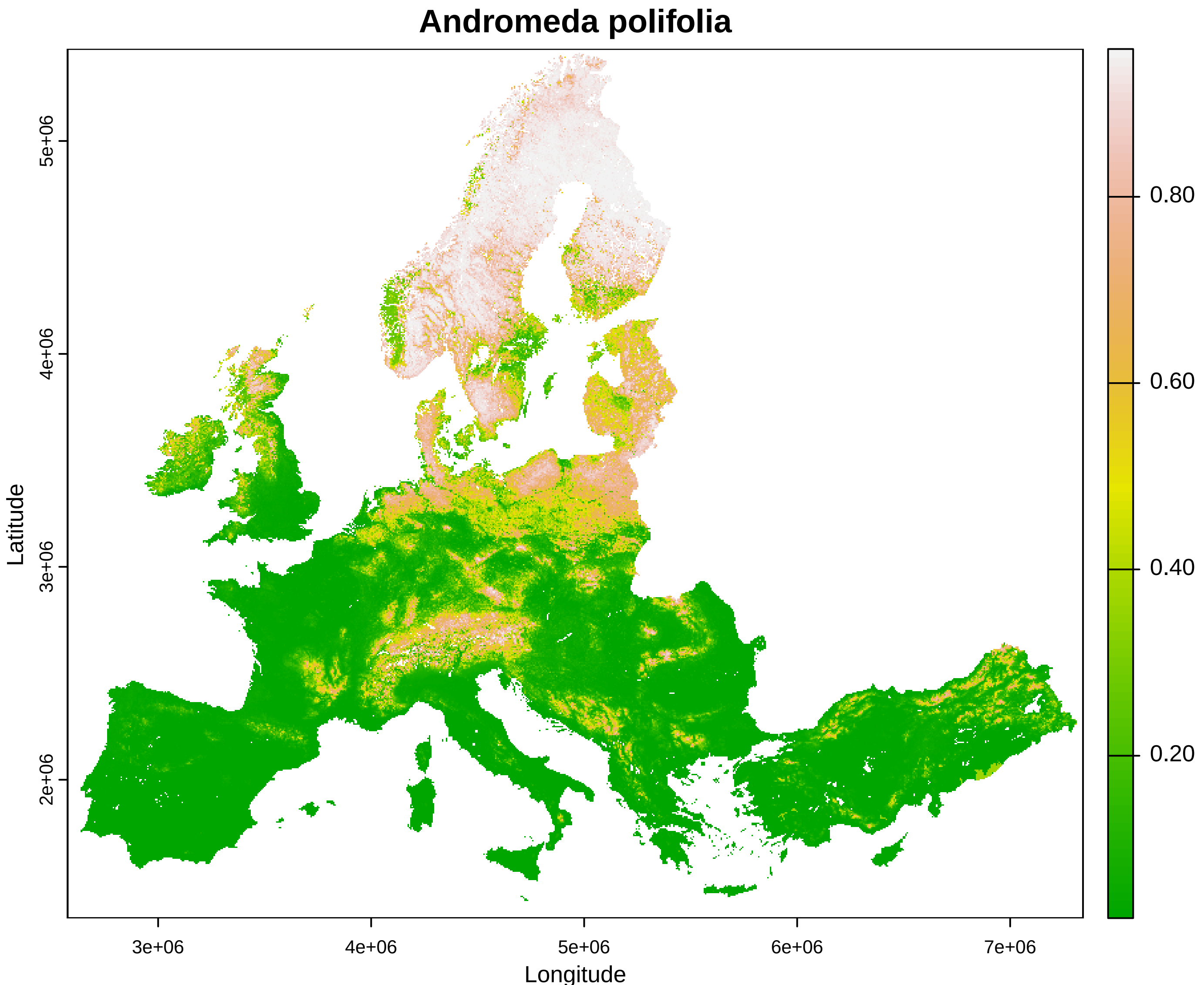
Andromeda polifolia
Latitude
5e+06
4e+06
3e+06
2e+06
Longitude
3e+06
4e+06
5e+06
6e+06
7e+06
0.80
0.60
0.40
0.20

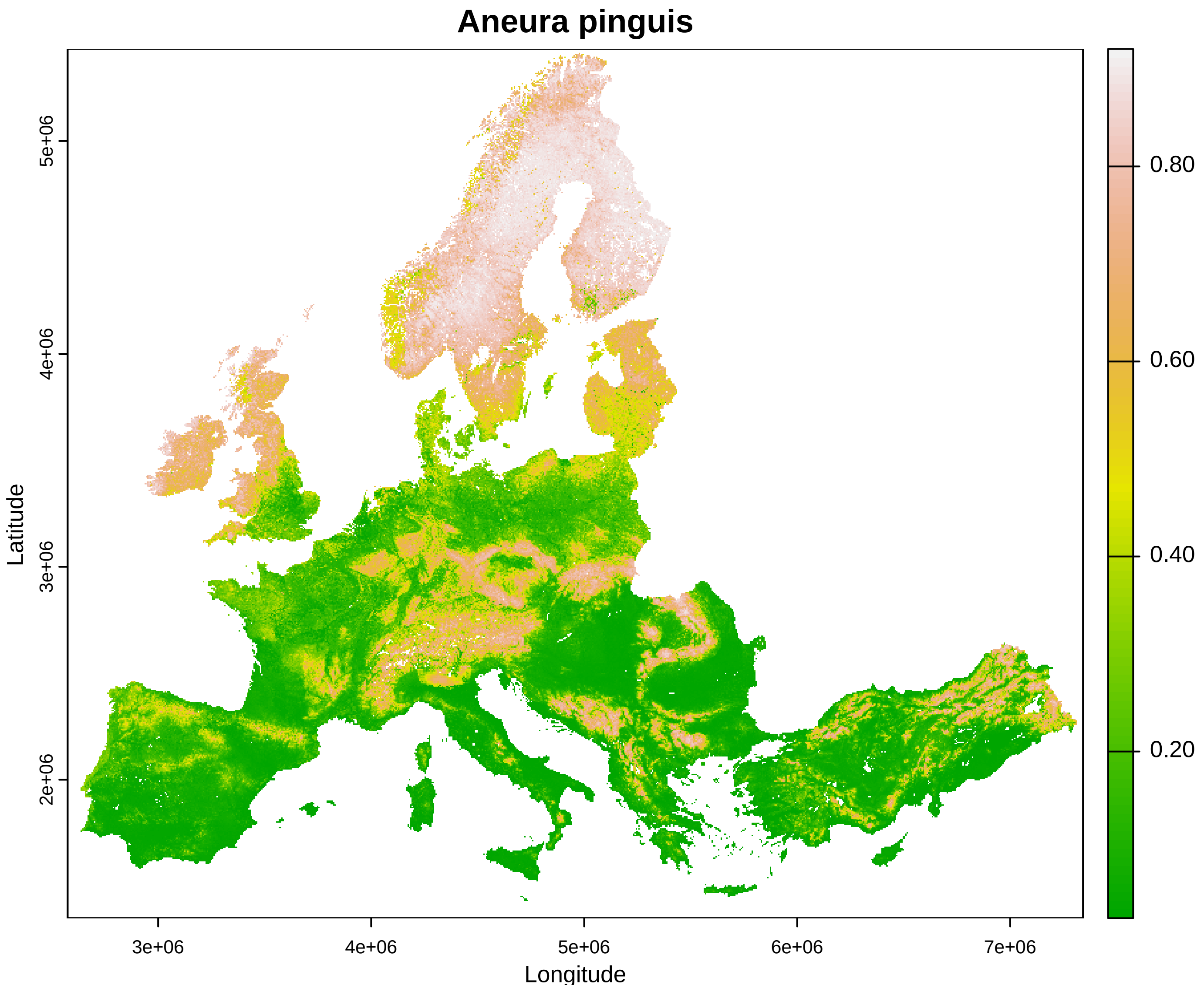

Aneura pinguis
Latitude
Longitude
5e+06
4e+06
3e+06
2e+06
3e+06
4e+06
5e+06
6e+06
7e+06
0.80
0.60
0.40
0.20

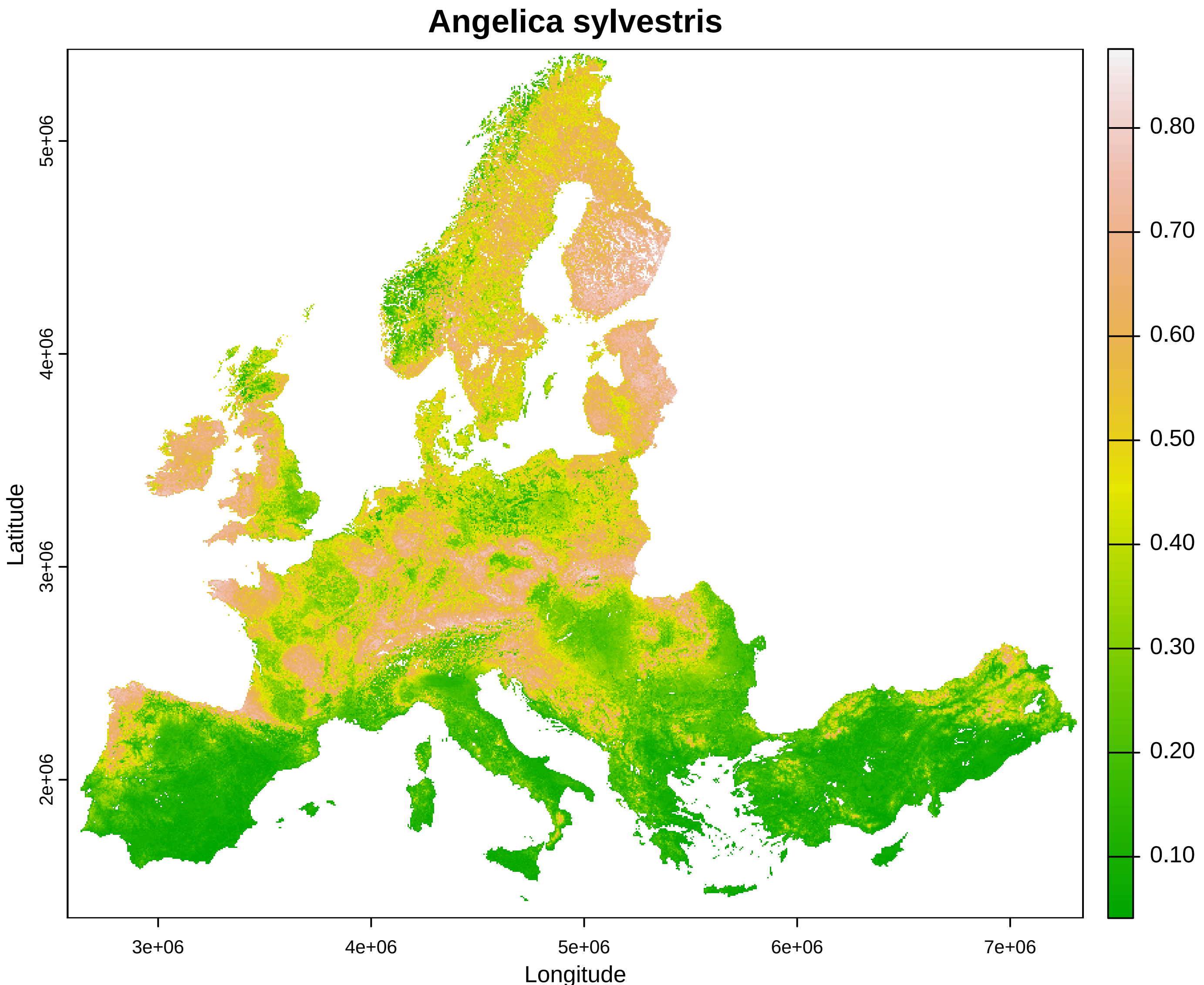

Angelica sylvestris
Latitude
5e+06
4e+06
3e+06
2e+06
Longitude
3e+06
4e+06
5e+06
6e+06
7e+06
0.80
0.70
0.60
0.50
0.40
0.30
0.20
0.10

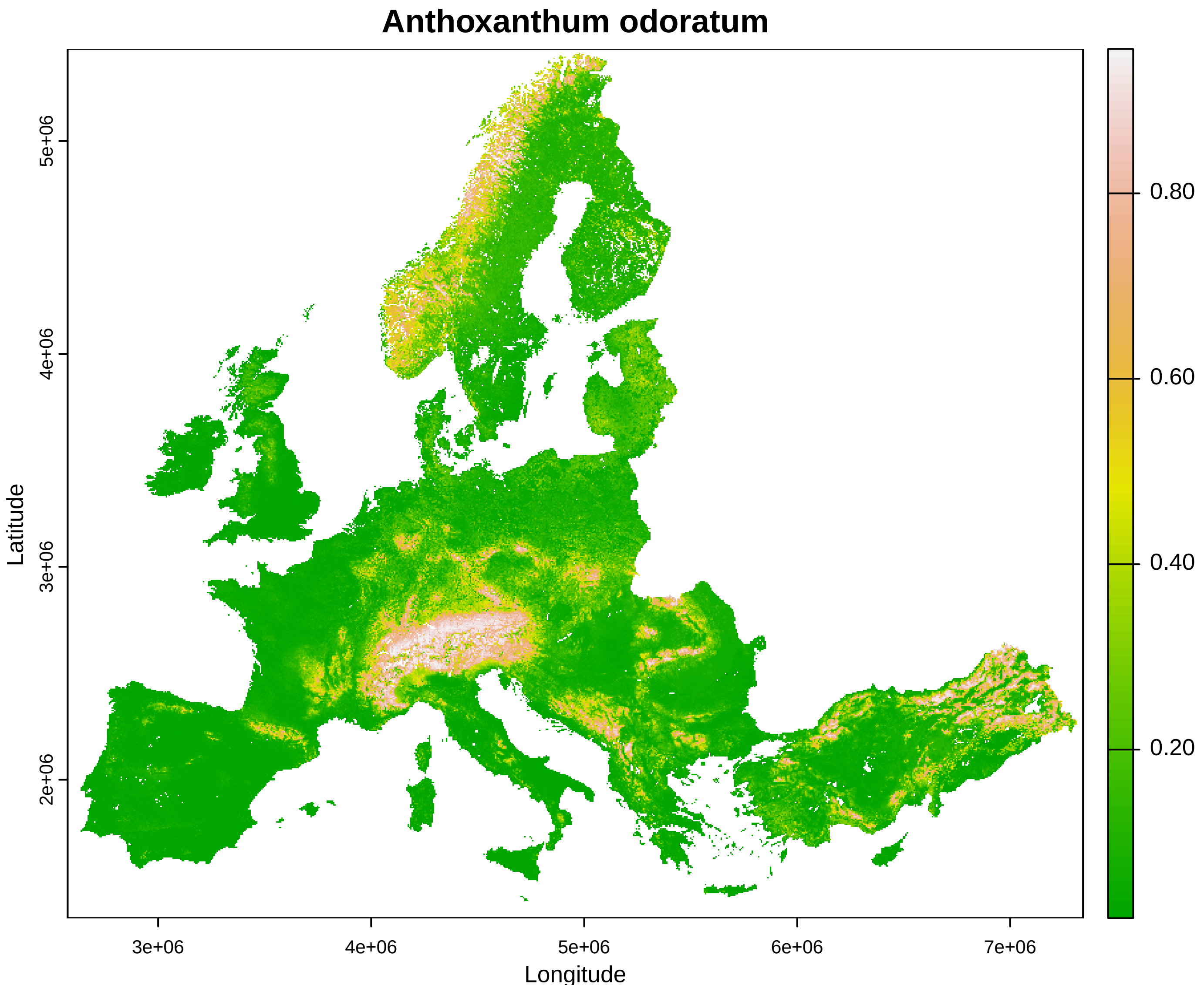
Anthoxanthum odoratum
Latitude
5e+06
4e+06
3e+06
2e+06
Longitude
3e+06
4e+06
5e+06
6e+06
7e+06
0.80
0.60
0.40
0.20

Arctostaphylos alpinus

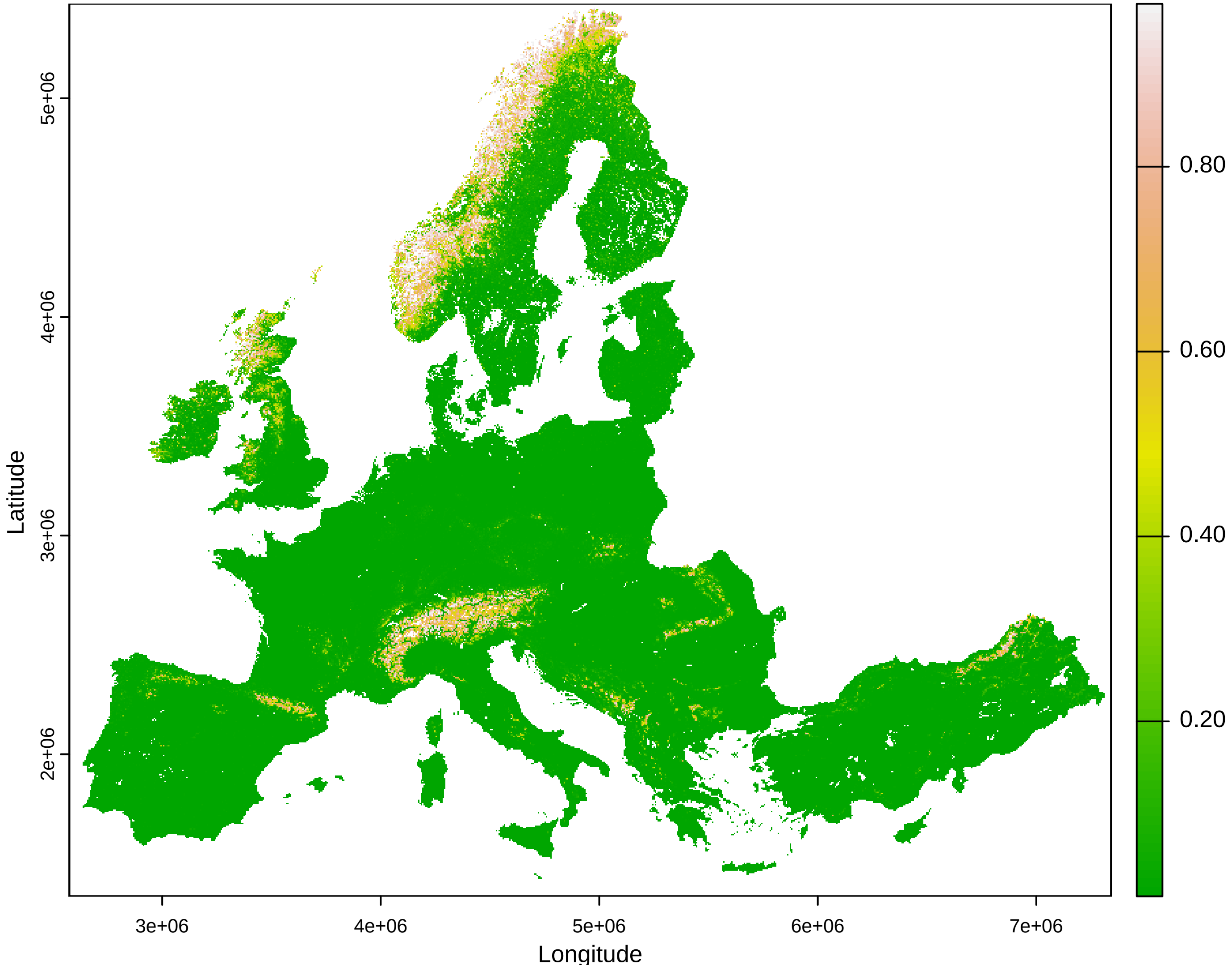

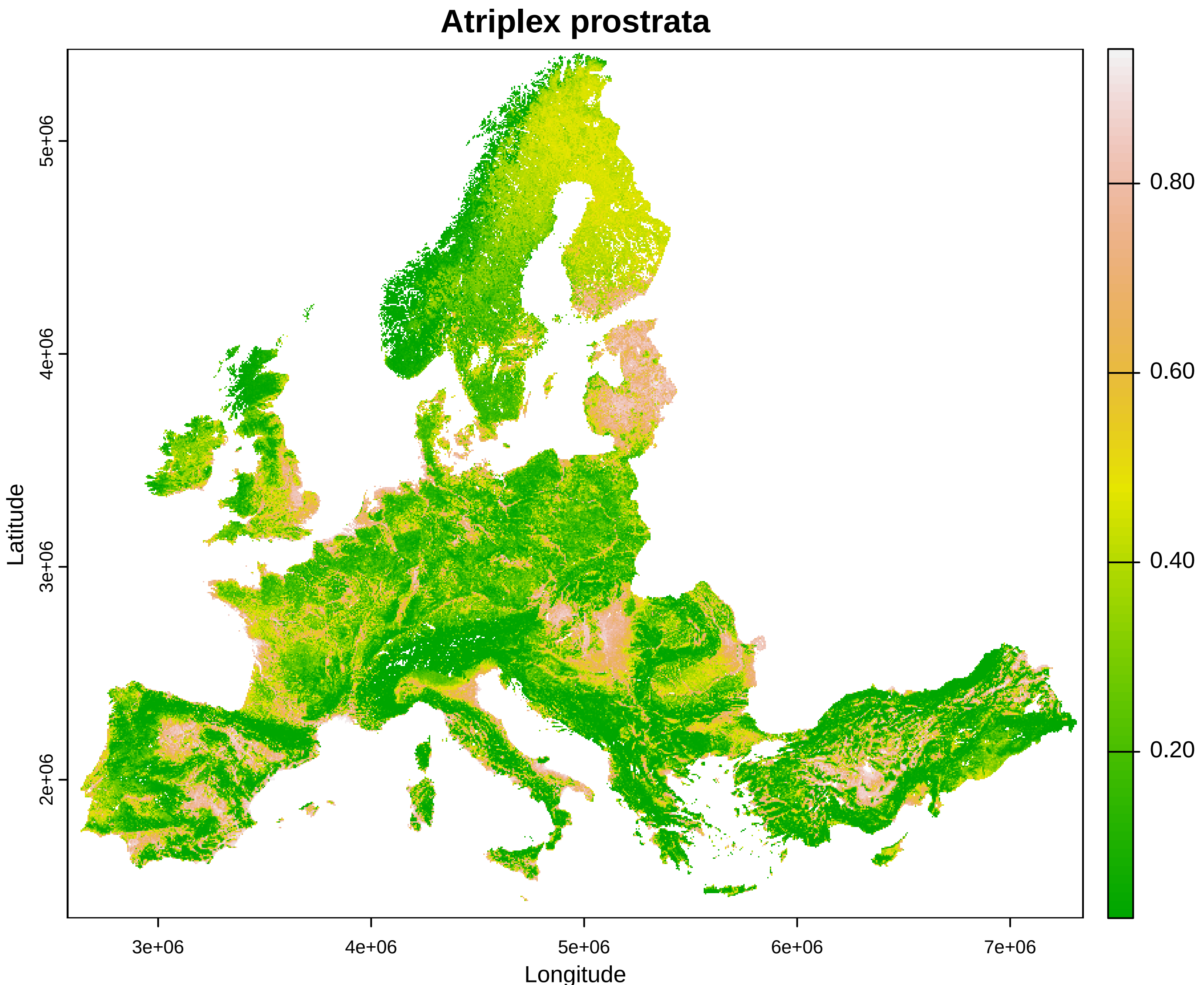

Atriplex prostrata
Latitude
Longitude
5e+06
4e+06
3e+06
2e+06
3e+06
4e+06
5e+06
6e+06
7e+06
0.80
0.60
0.40
0.20

Aulacomnium palustre

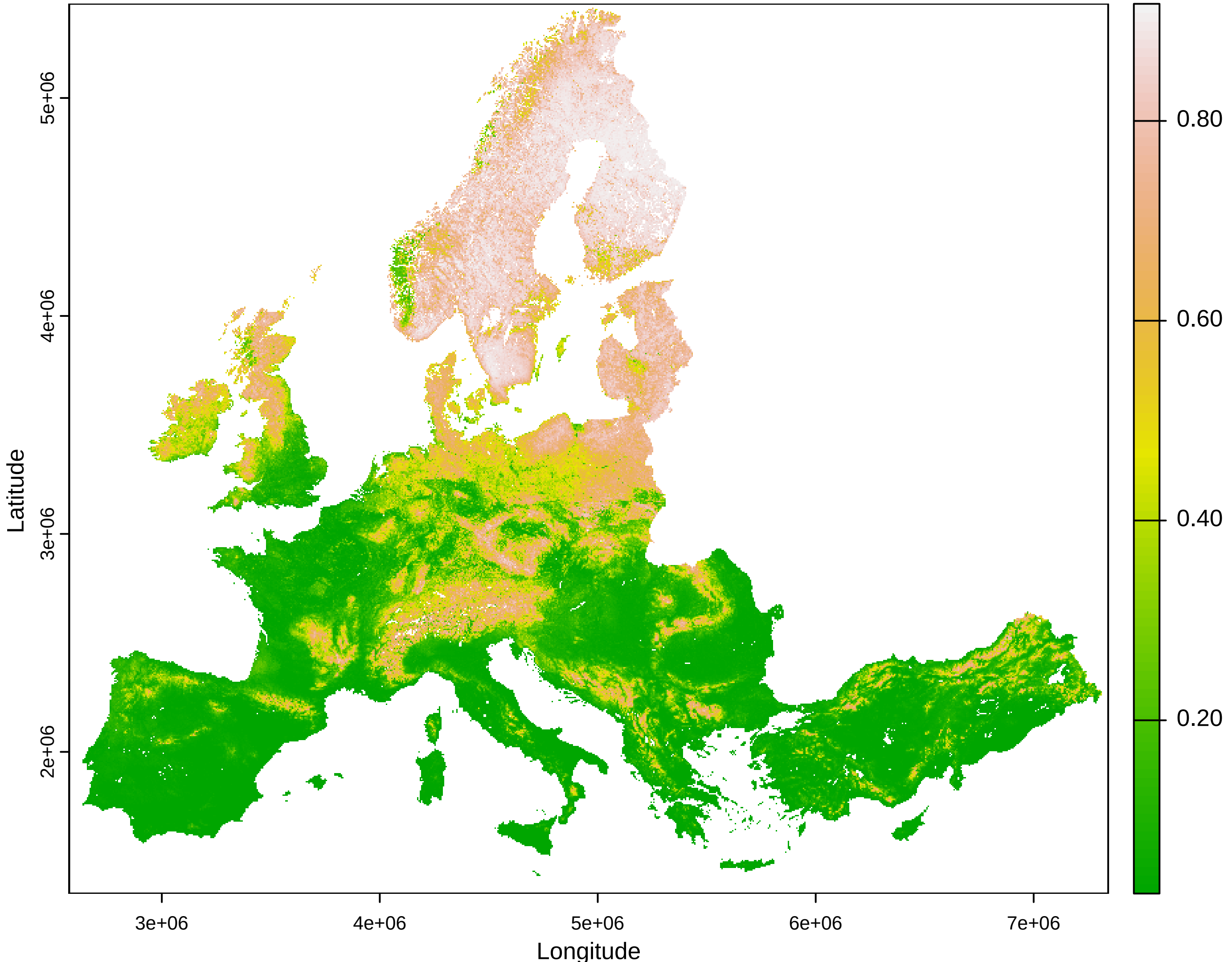

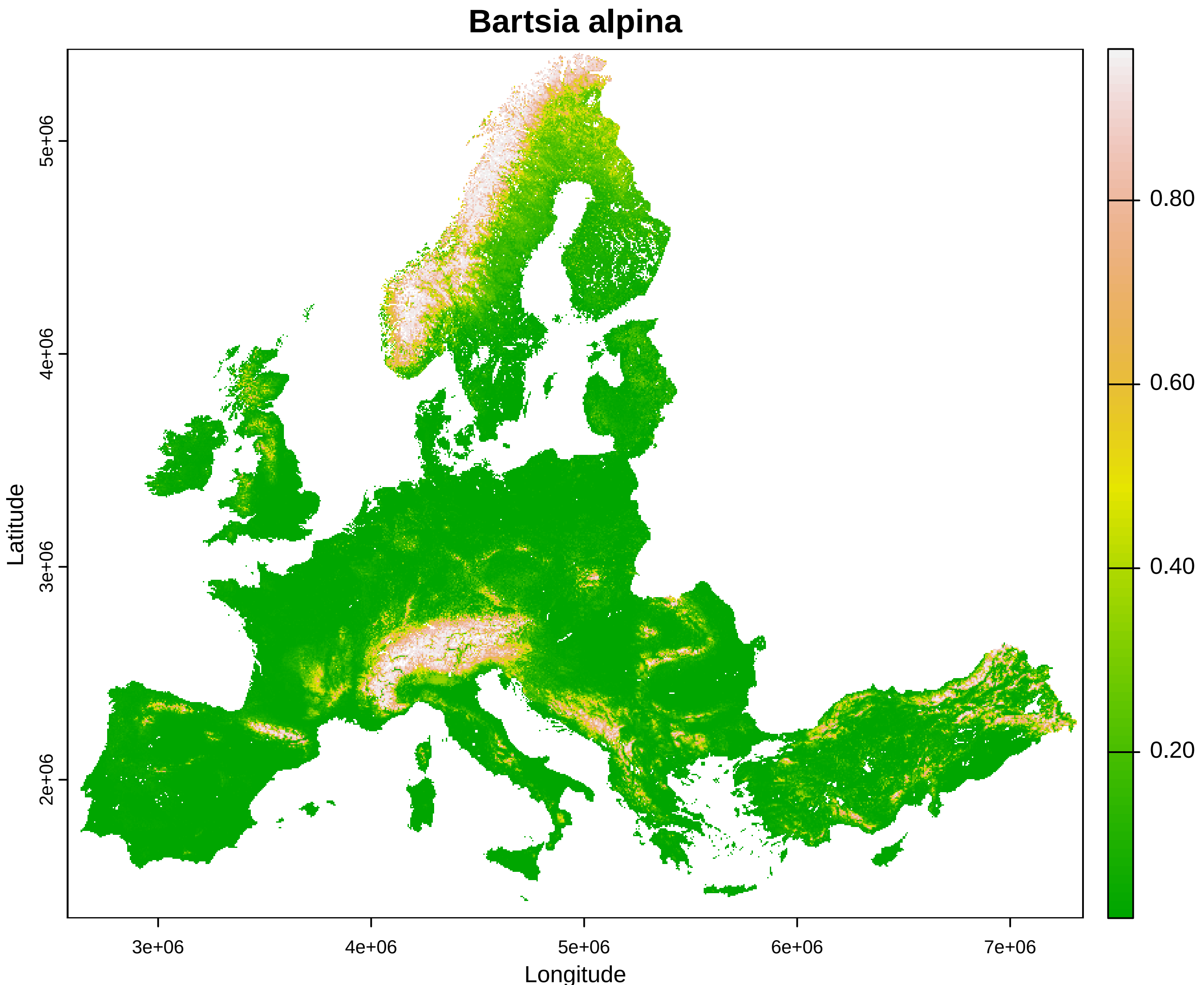
Bartsia alpina
Latitude
Longitude
5e+06
4e+06
3e+06
2e+06
3e+06
4e+06
5e+06
6e+06
7e+06
0.80
0.60
0.40
0.20

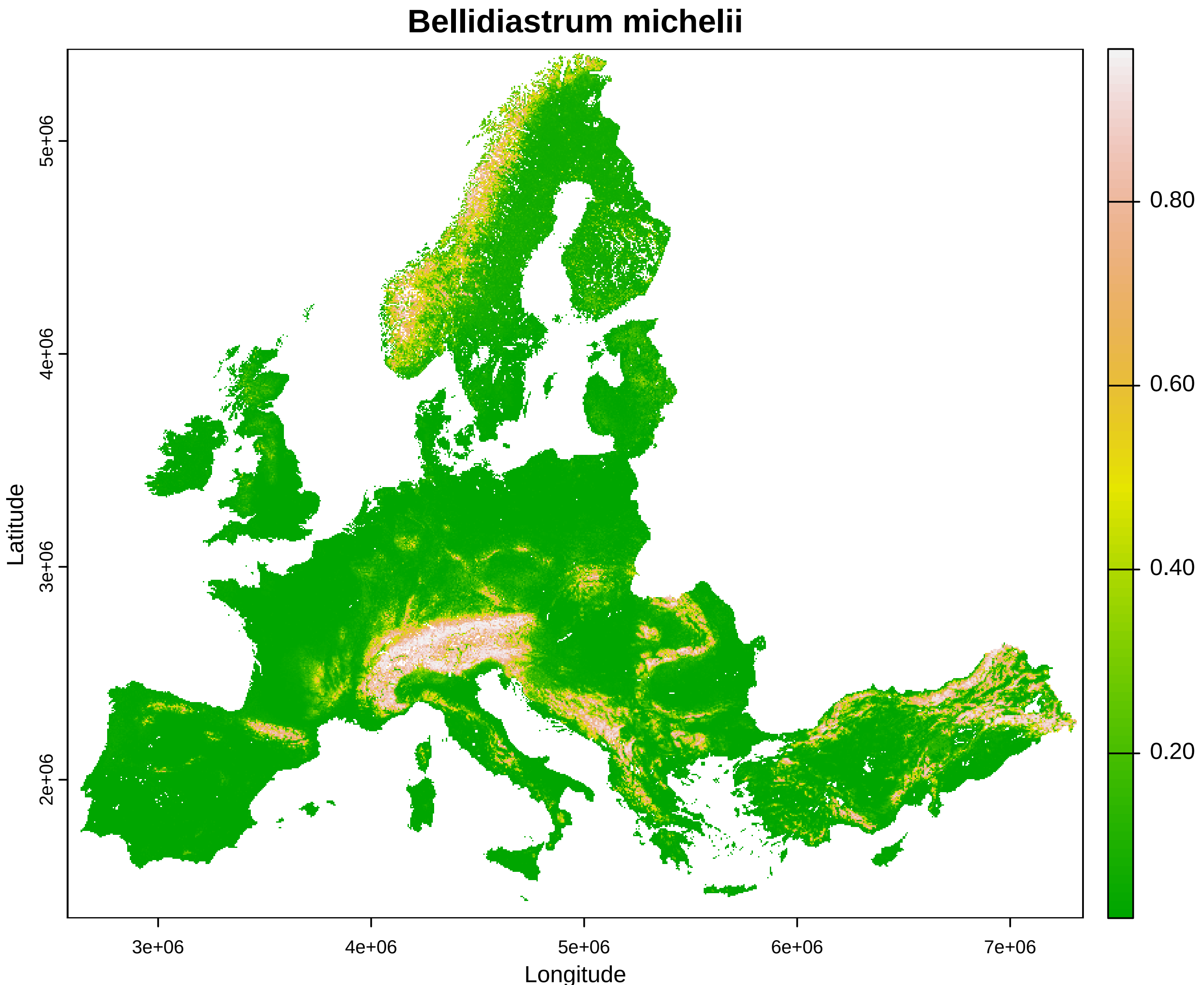

Bellidiastrum michelii
Latitude
Longitude
5e+06
4e+06
3e+06
2e+06
3e+06
4e+06
5e+06
6e+06
7e+06
0.80
0.60
0.40
0.20

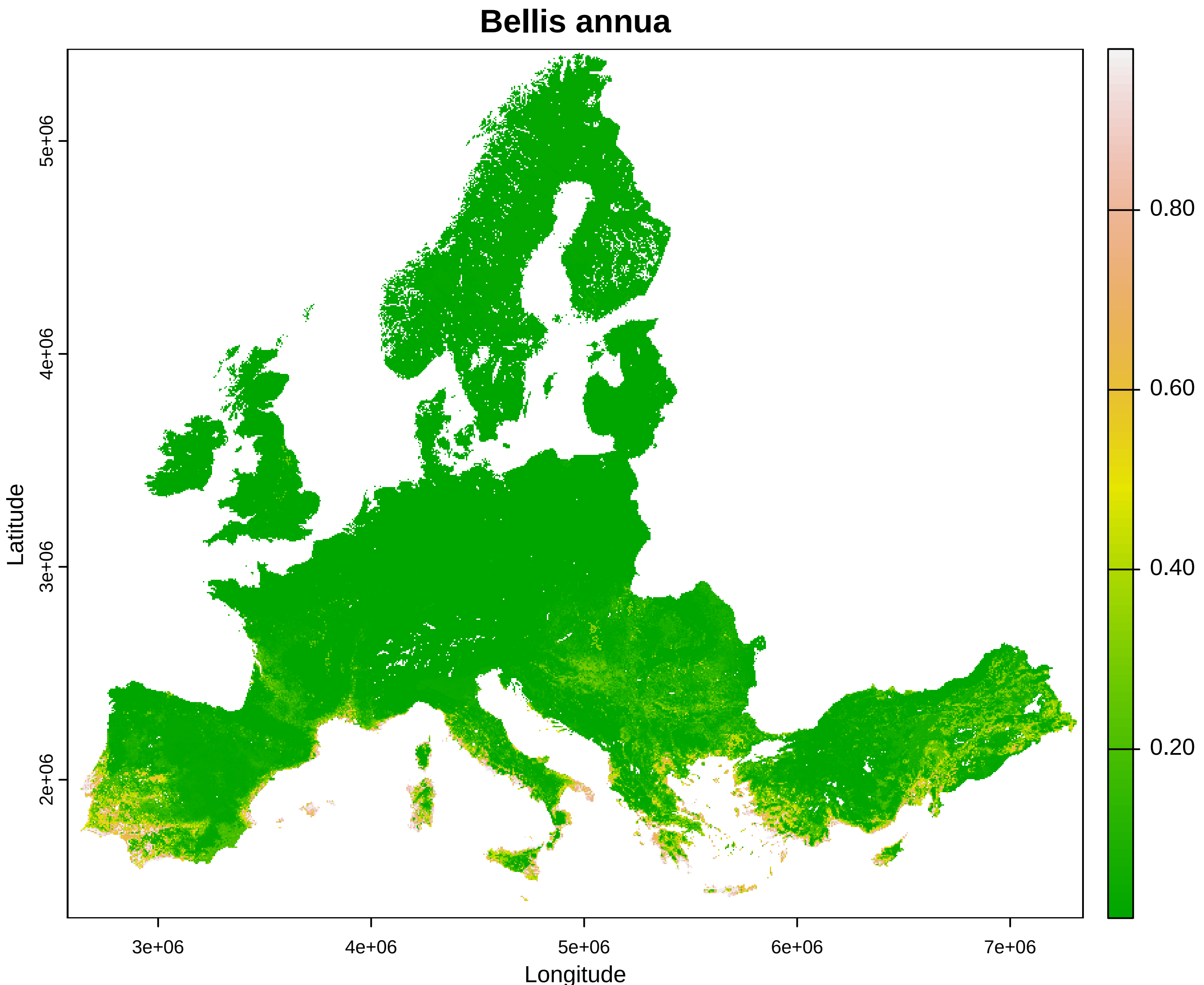
Bellis annua
Latitude
Longitude
5e+06
4e+06
3e+06
2e+06
3e+06
4e+06
5e+06
6e+06
7e+06
0.80
0.60
0.40
0.20

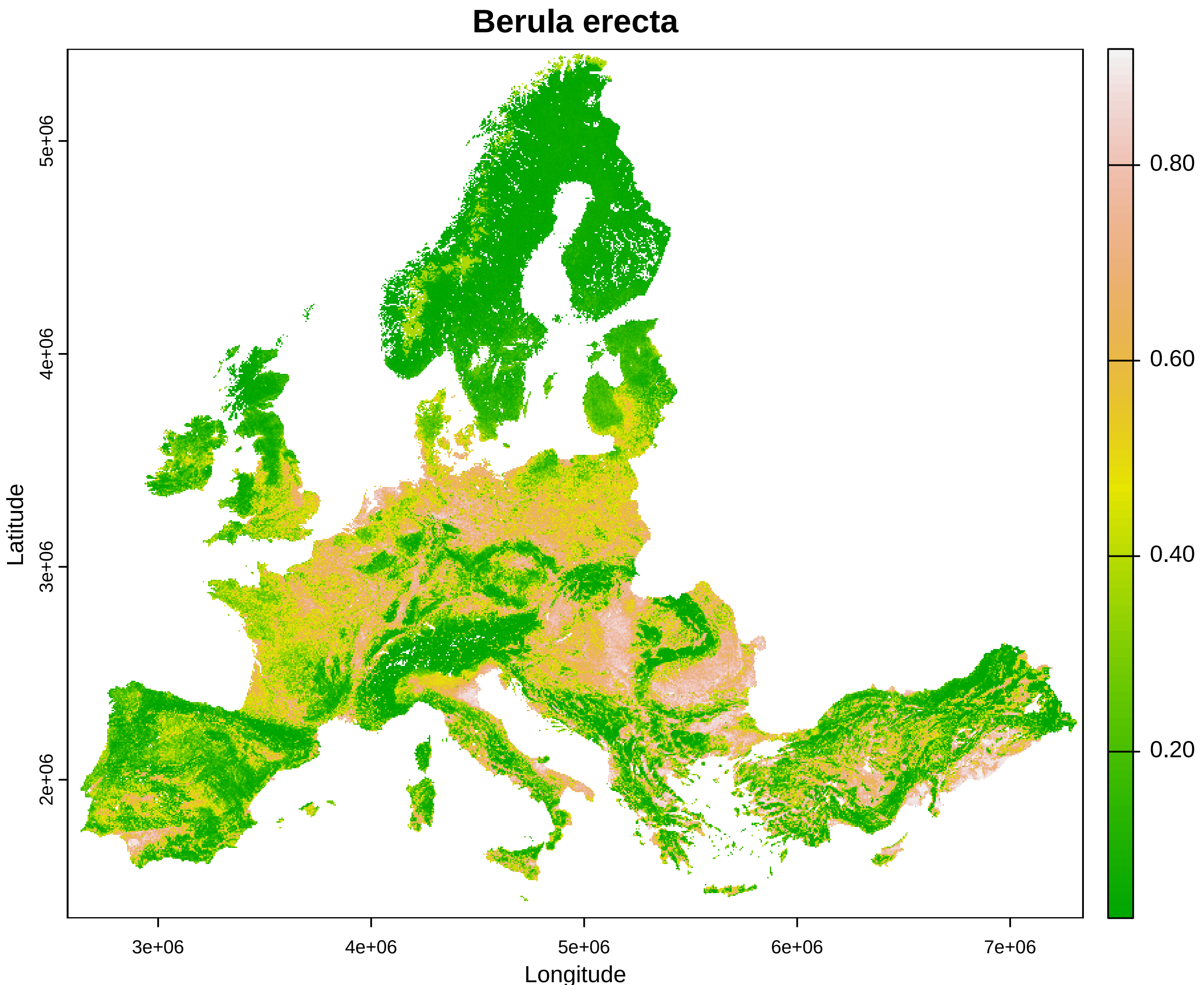

Berula erecta
Latitude
Longitude
5e+06
4e+06
3e+06
2e+06
3e+06
4e+06
5e+06
6e+06
7e+06
0.80
0.60
0.40
0.20

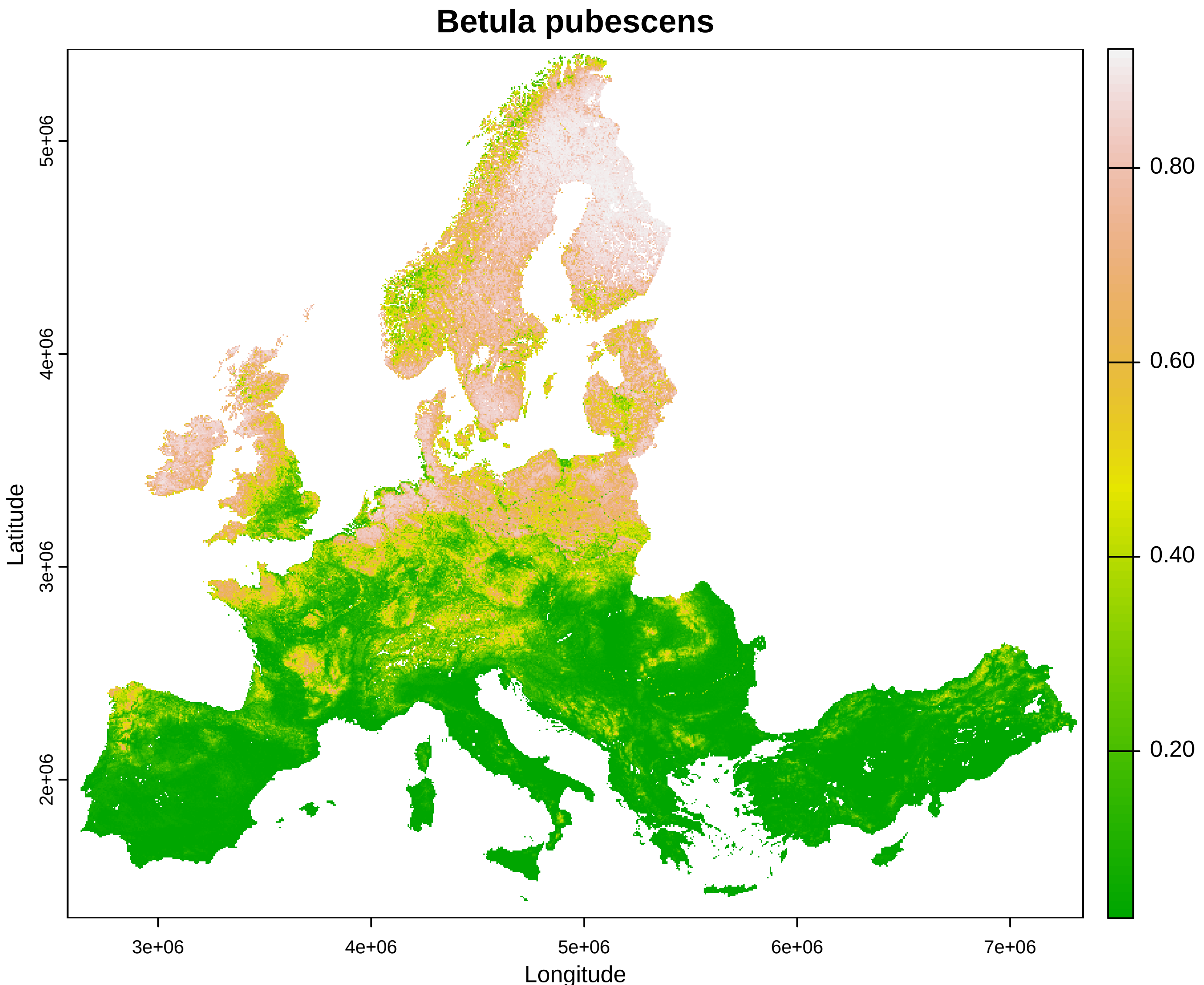
Betula pubescens
Latitude
Longitude
5e+06
4e+06
3e+06
2e+06
3e+06
4e+06
5e+06
6e+06
7e+06
0.80
0.60
0.40
0.20

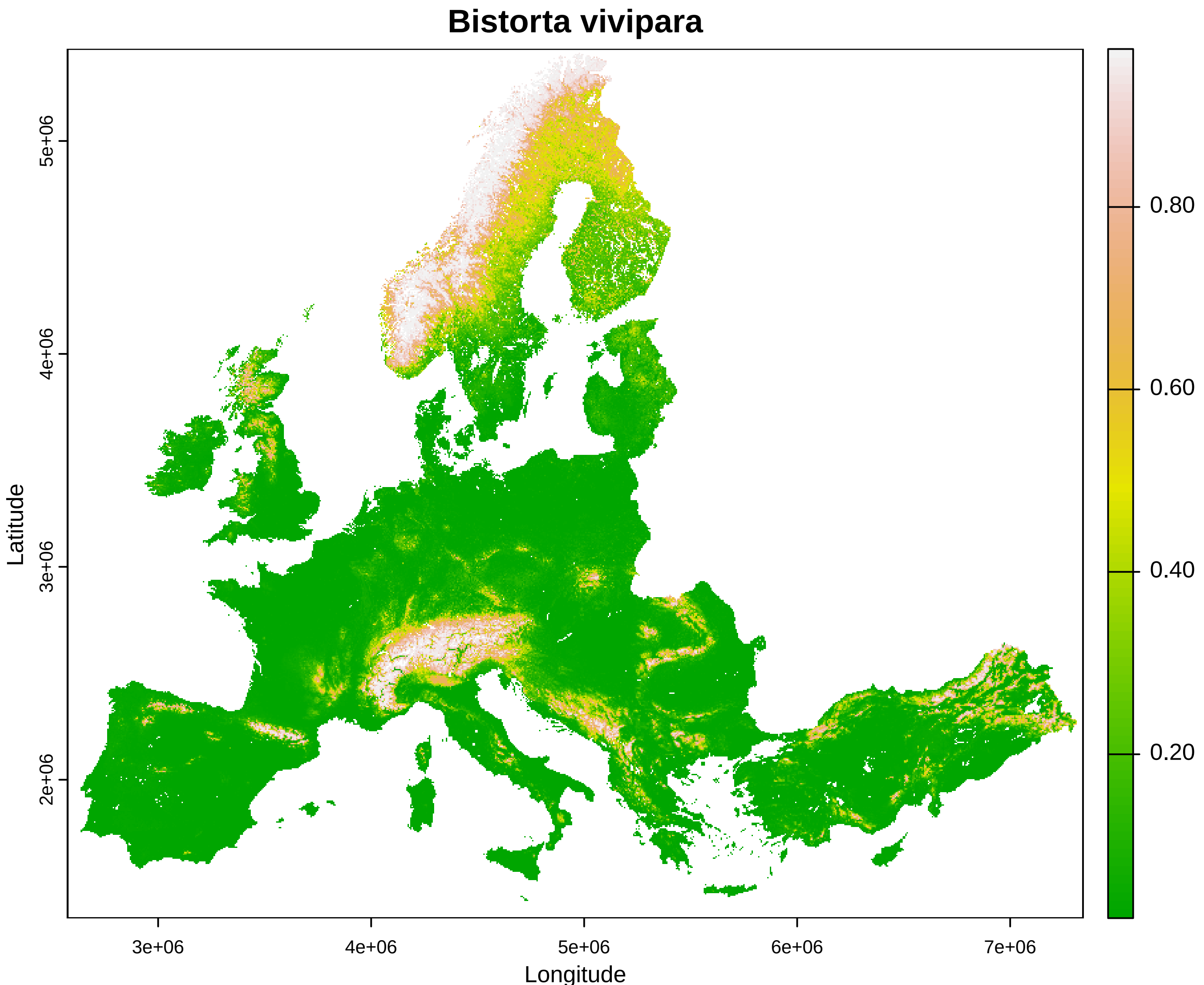
Bistorta vivipara
5e+06
4e+06
3e+06
2e+06
Latitude
3e+06
4e+06
5e+06
6e+06
7e+06
Longitude
0.80
0.60
0.40
0.20

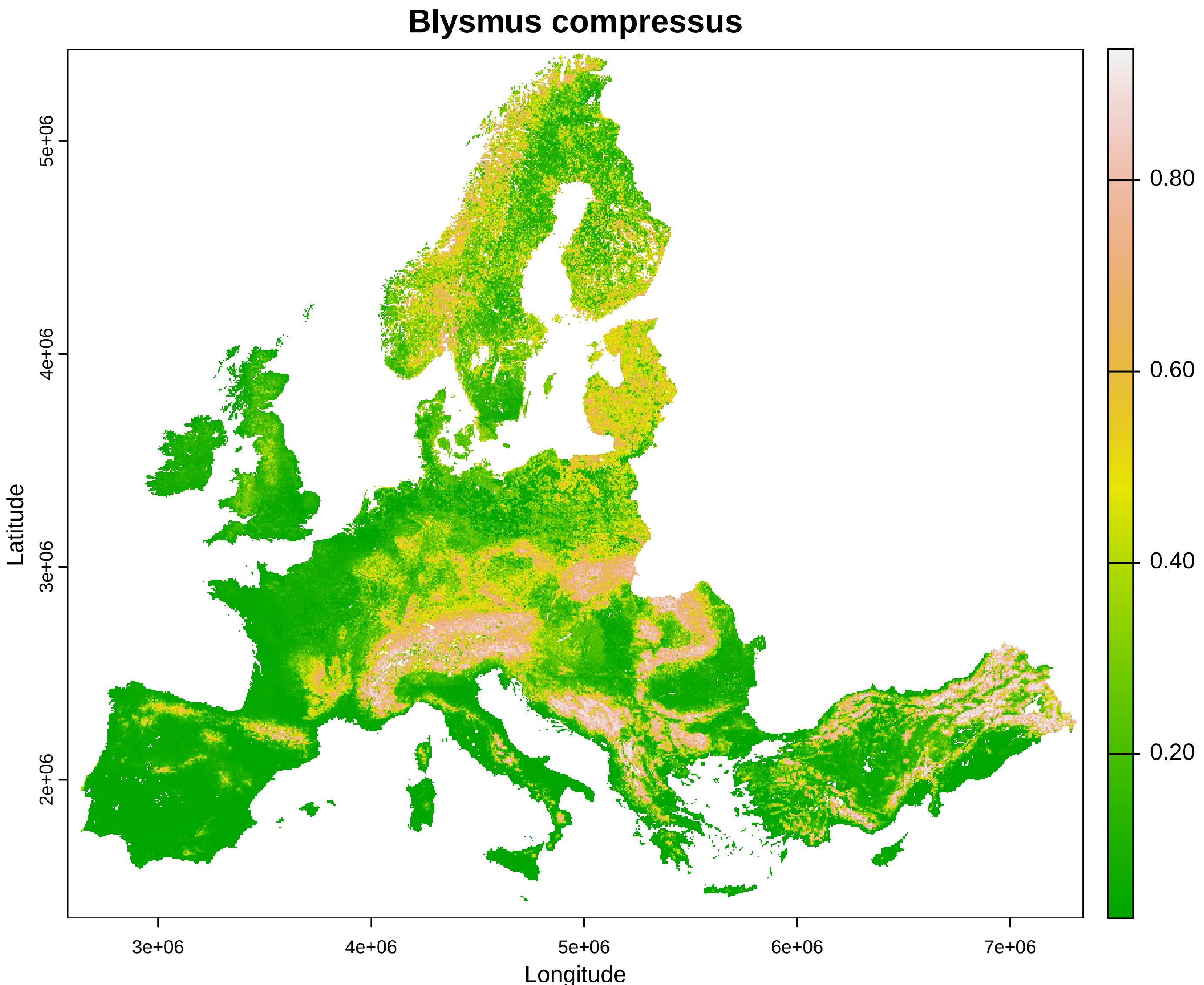

Blysmus compressus
Latitude
5e+06
4e+06
3e+06
2e+06
3e+06
4e+06
5e+06
6e+06
7e+06
Longitude
0.80
0.60
0.40
0.20

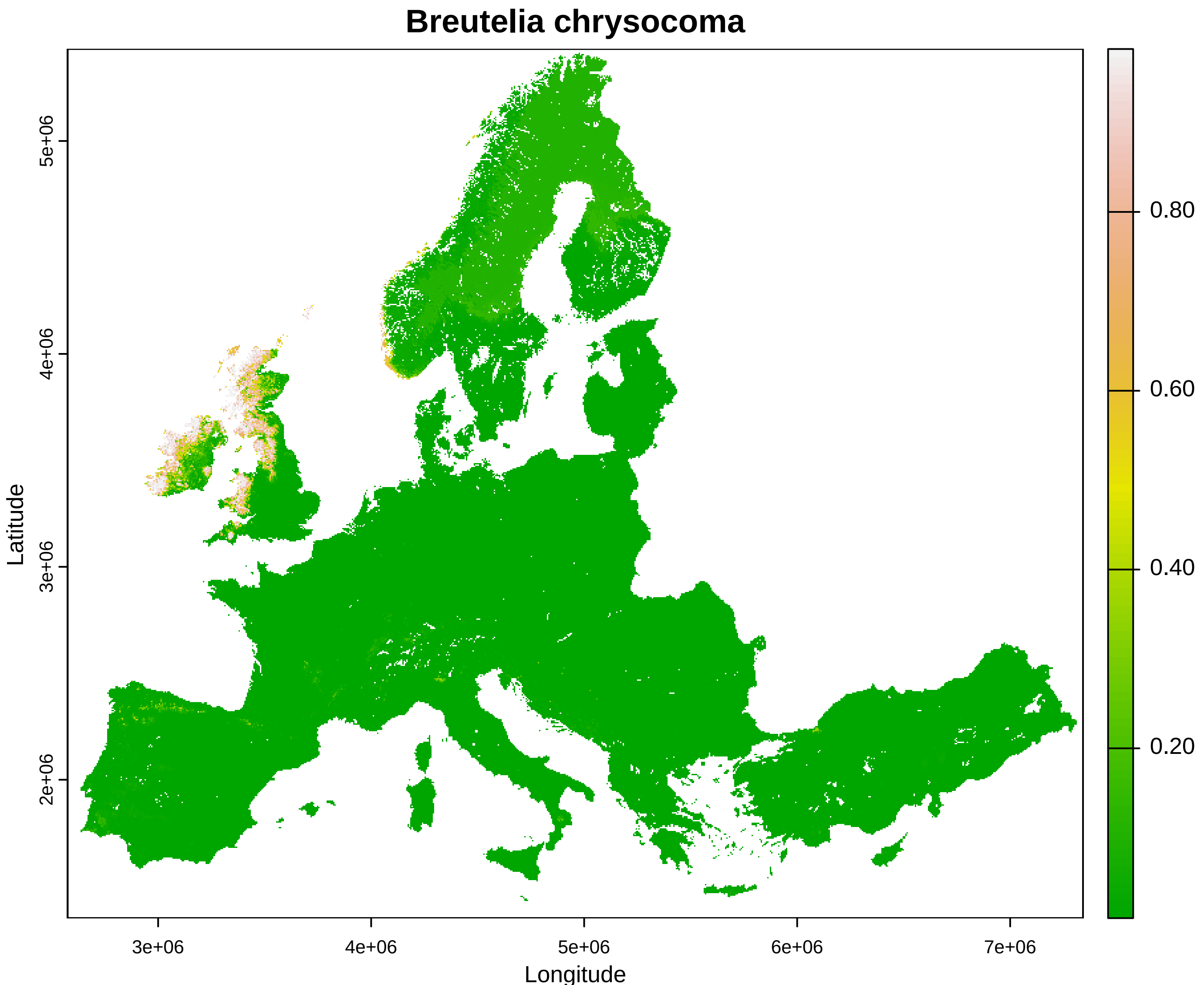

Breutelia chrysocoma
Latitude
Longitude
5e+06
4e+06
3e+06
2e+06
3e+06
4e+06
5e+06
6e+06
7e+06
0.80
0.60
0.40
0.20

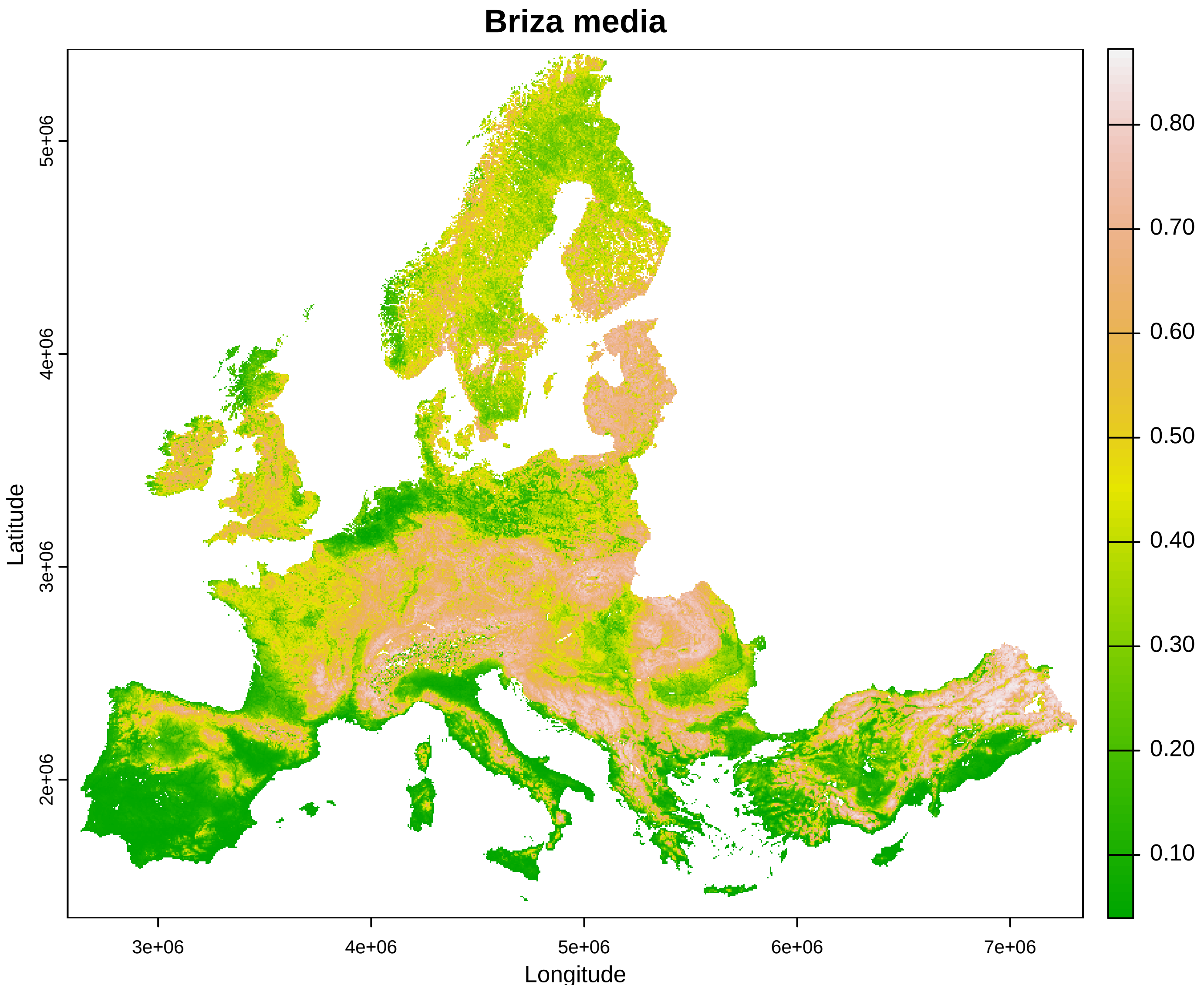
Briza media
Latitude
Longitude
5e+06
4e+06
3e+06
2e+06
3e+06
4e+06
5e+06
6e+06
7e+06
0.80
0.70
0.60
0.50
0.40
0.30
0.20
0.10

Bruckenthalia spiculifolia

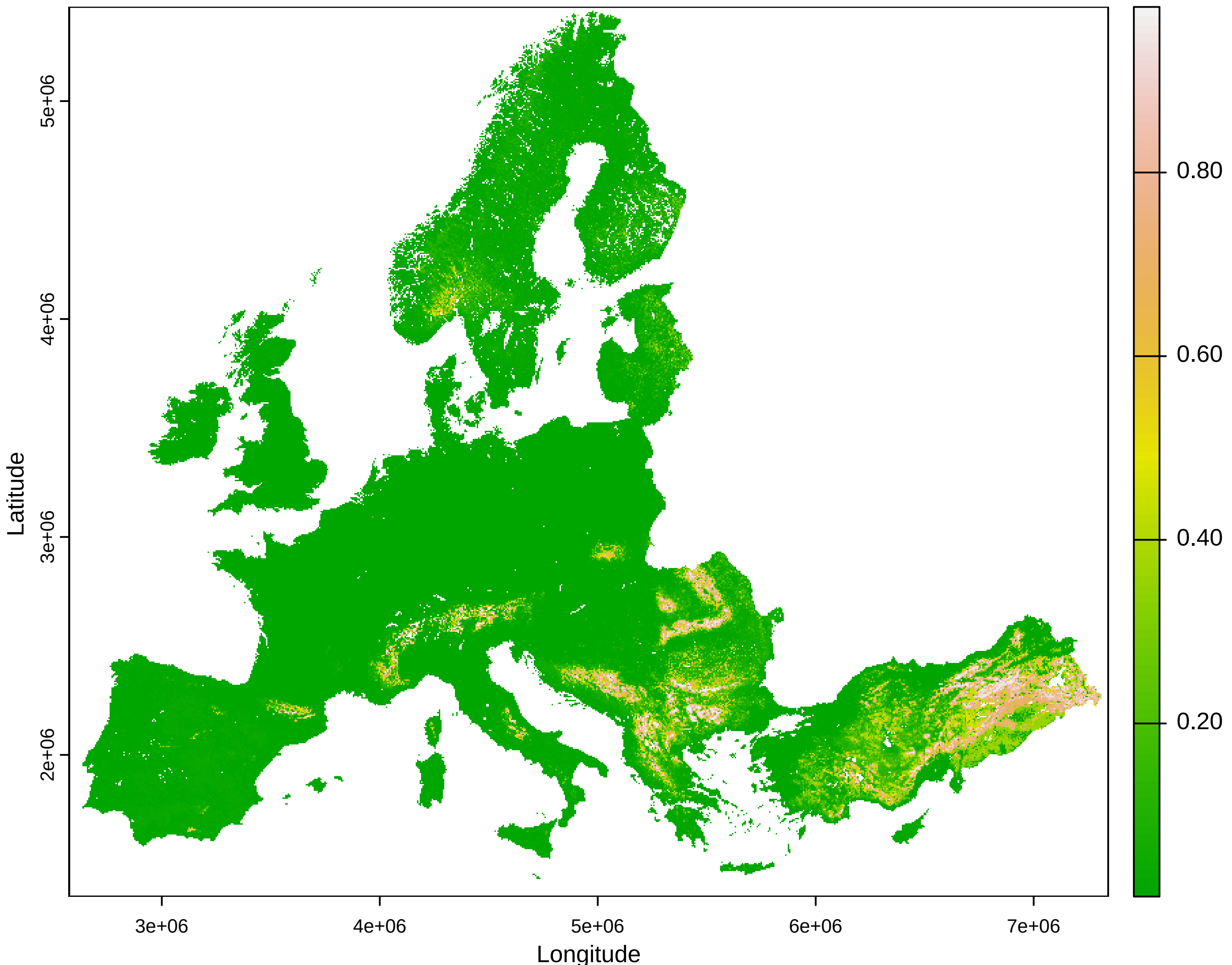

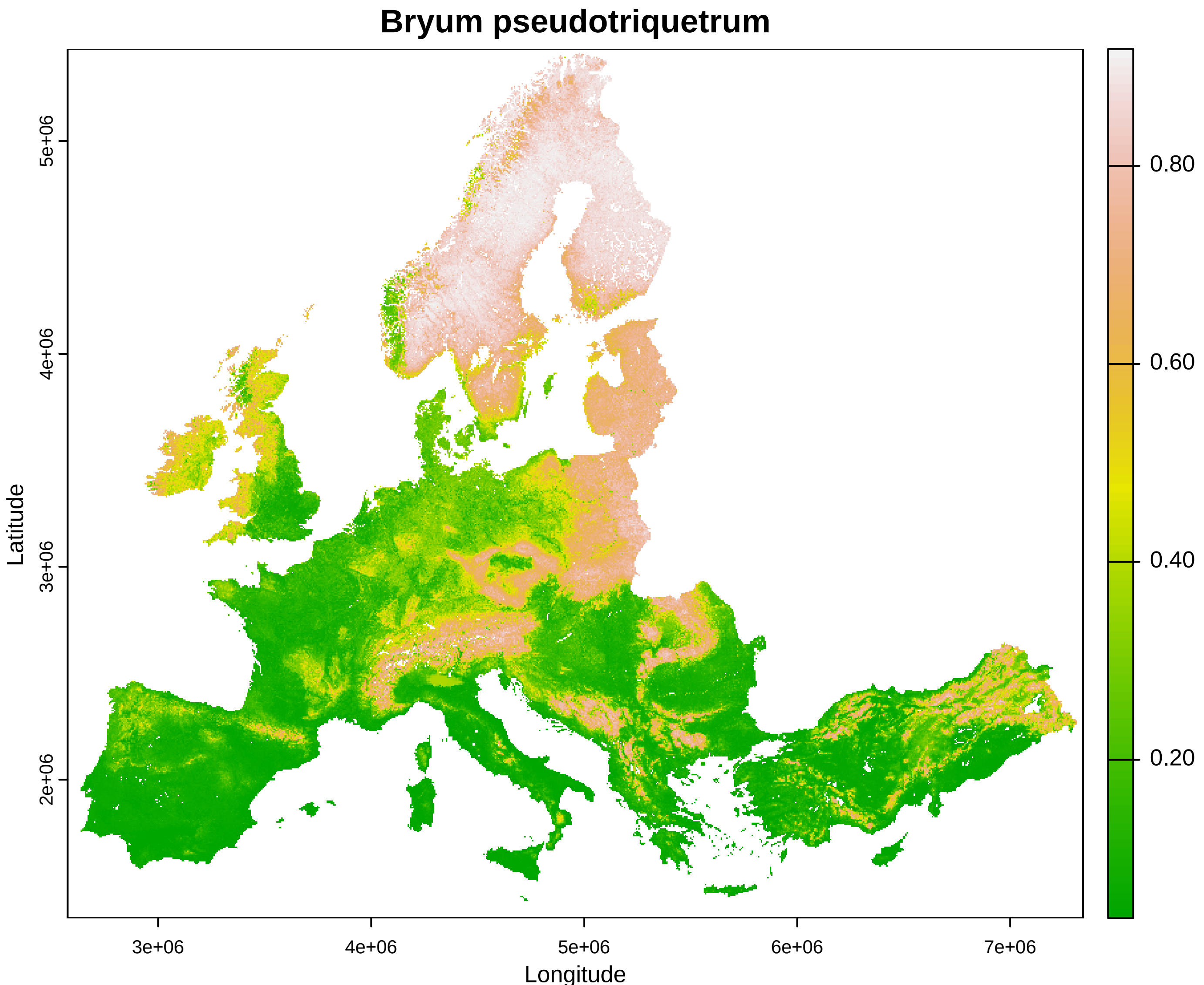

Bryum pseudotriquetrum
Latitude
5e+06
4e+06
3e+06
2e+06
3e+06
4e+06
5e+06
6e+06
7e+06
Longitude
0.80
0.60
0.40
0.20

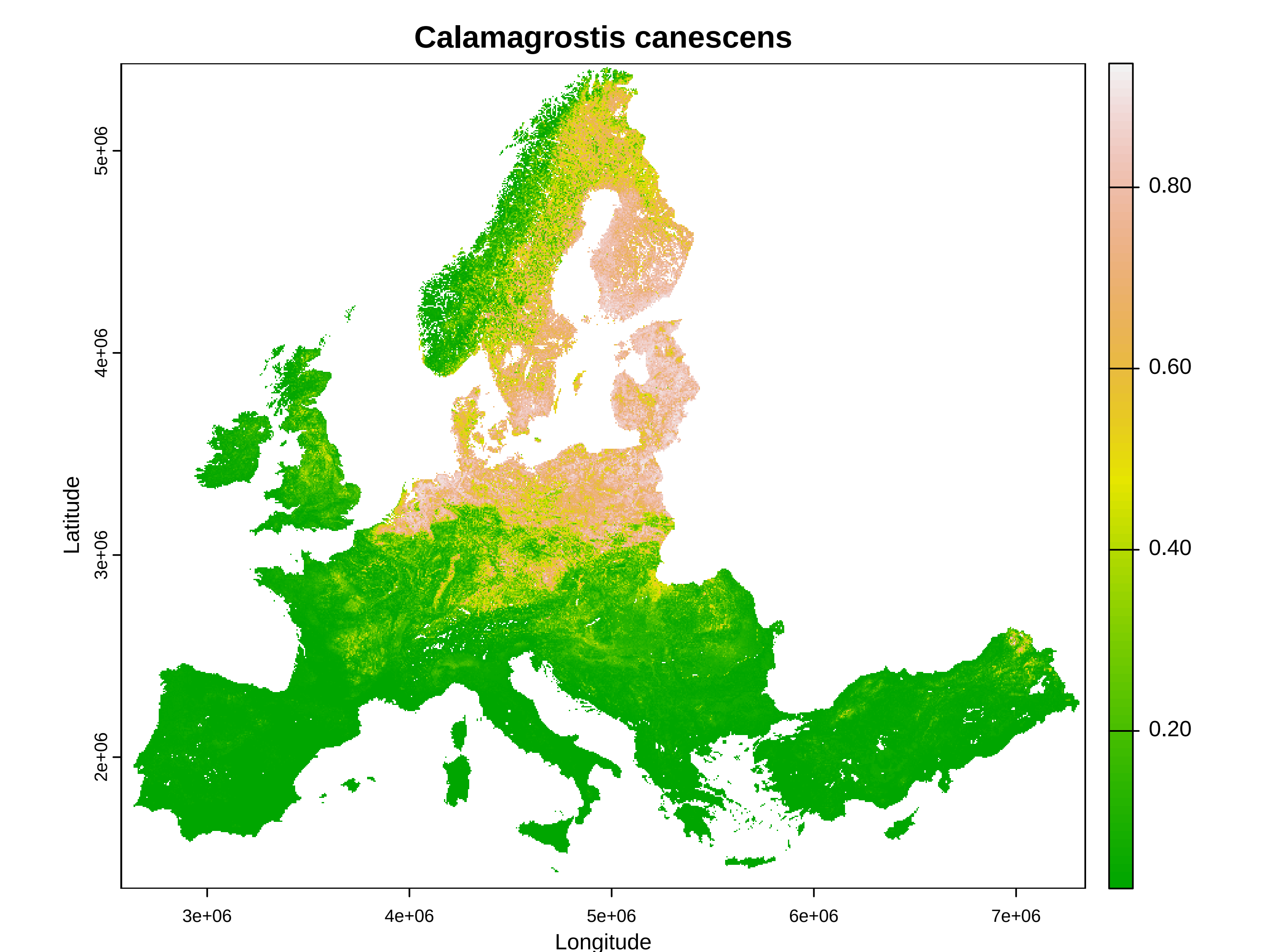

Calamagrostis canescens
Latitude
Longitude
5e+06
4e+06
3e+06
2e+06
3e+06
4e+06
5e+06
6e+06
7e+06
0.80
0.60
0.40
0.20

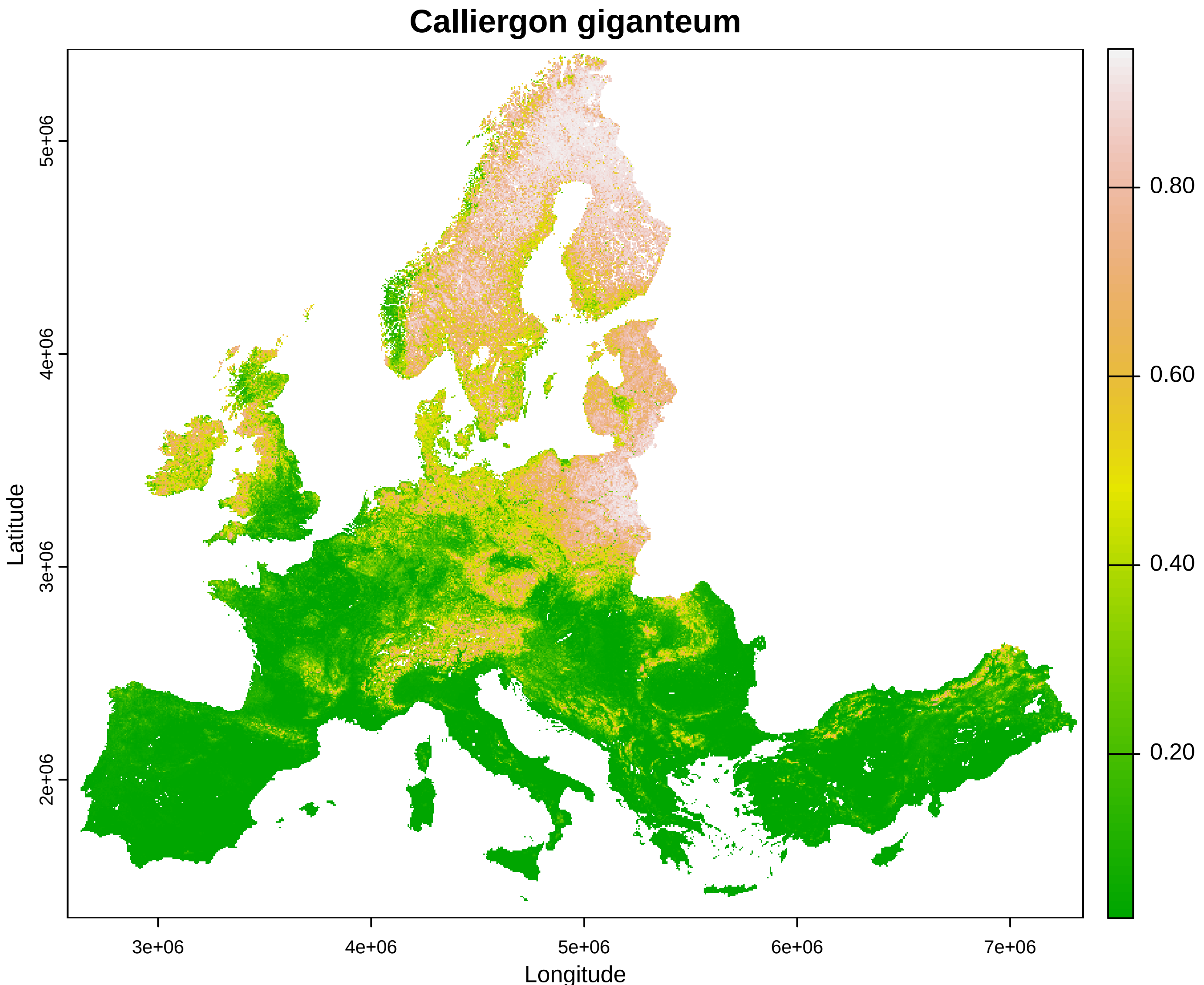
Calliergon giganteum
Latitude
Longitude
5e+06
4e+06
3e+06
2e+06
3e+06
4e+06
5e+06
6e+06
7e+06
0.80
0.60
0.40
0.20

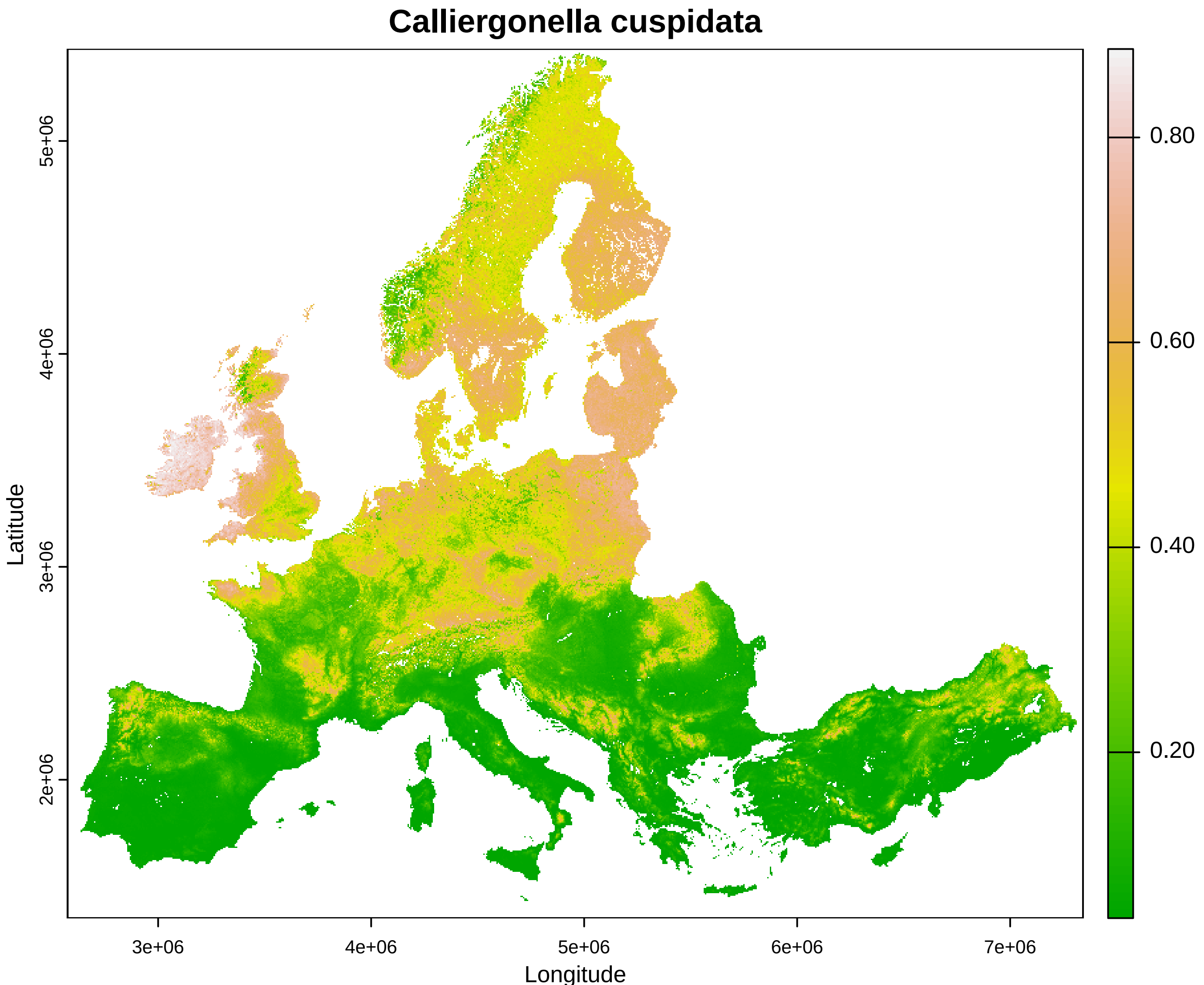
Calliergonella cuspidata
Latitude
5e+06
4e+06
3e+06
2e+06
Longitude
3e+06
4e+06
5e+06
6e+06
7e+06
0.80
0.60
0.40
0.20

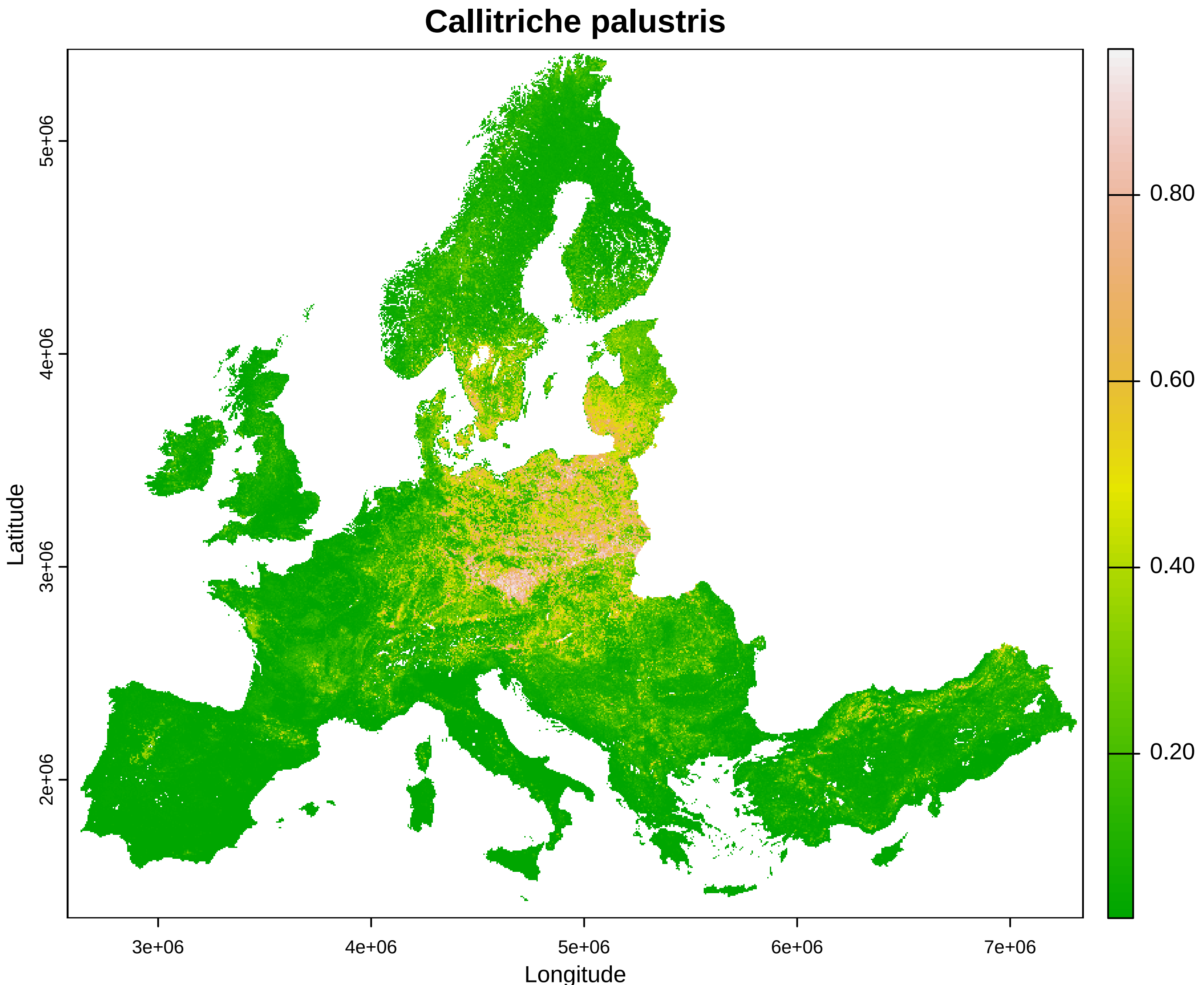
Callitriche palustris
Latitude
5e+06
4e+06
3e+06
2e+06
Longitude
3e+06
4e+06
5e+06
6e+06
7e+06
0.80
0.60
0.40
0.20

Calluna vulgaris

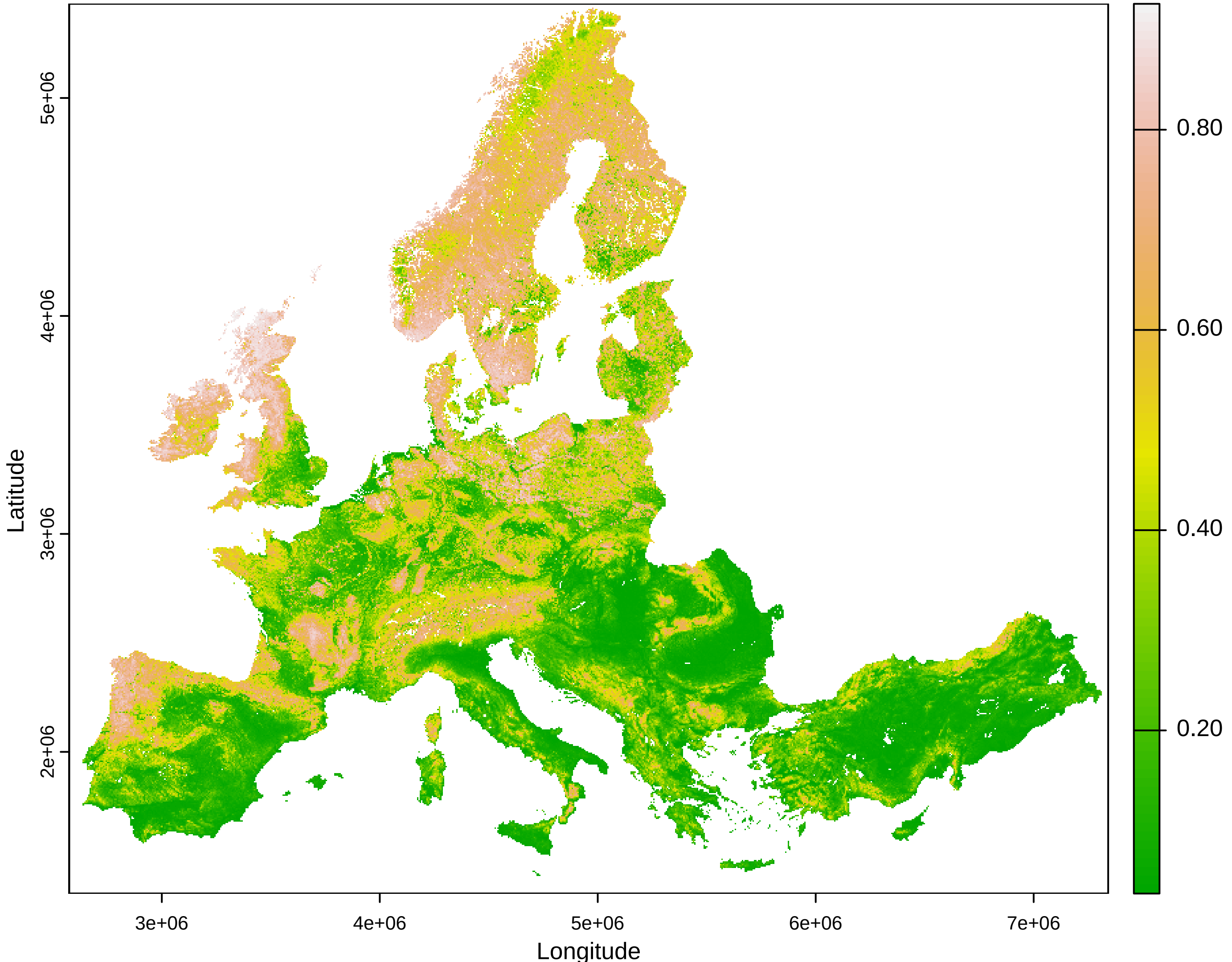

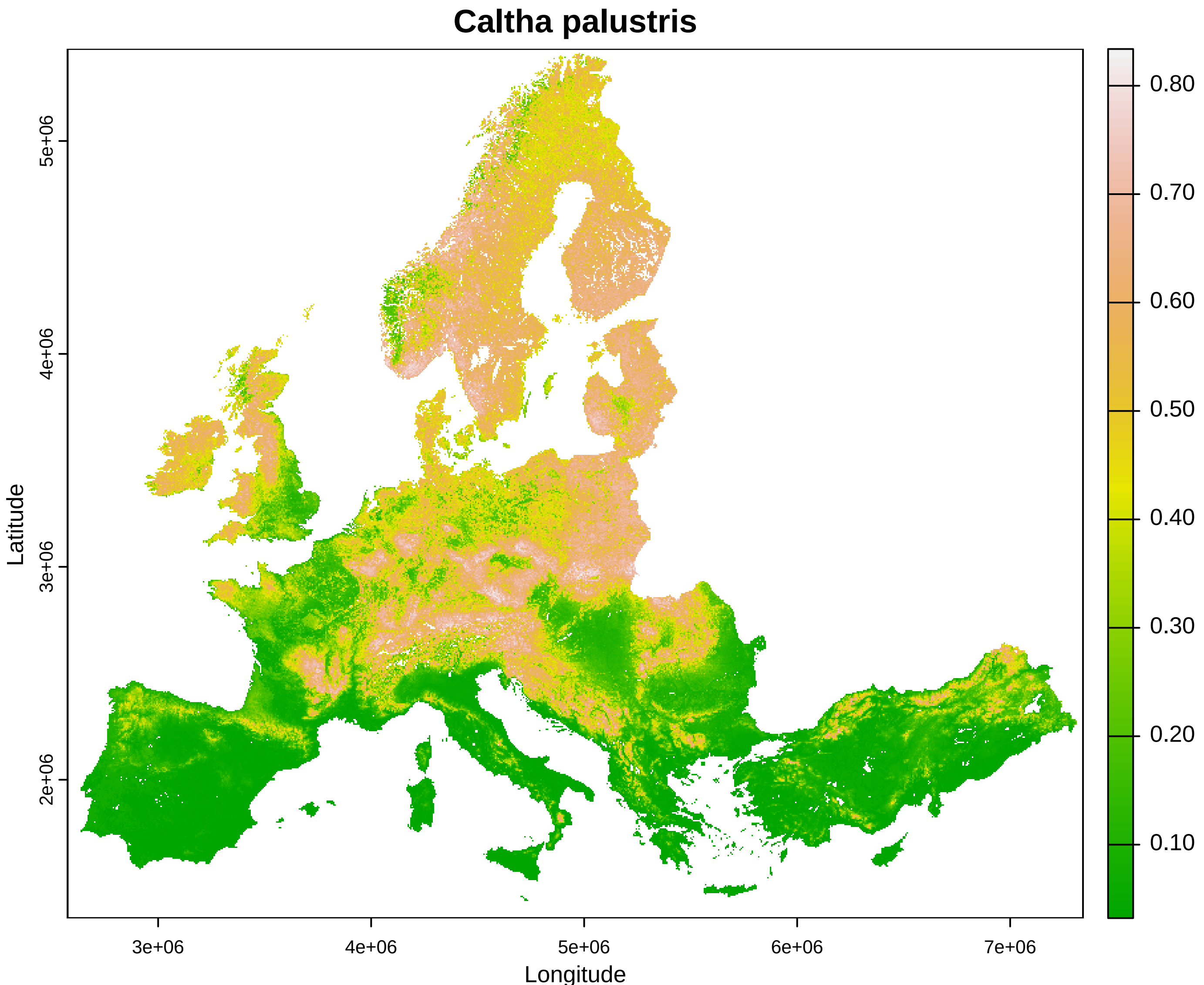
Caltha palustris
Latitude
5e+06
4e+06
3e+06
2e+06
3e+06
4e+06
5e+06
6e+06
7e+06
Longitude
0.80
0.70
0.60
0.50
0.40
0.30
0.20
0.10

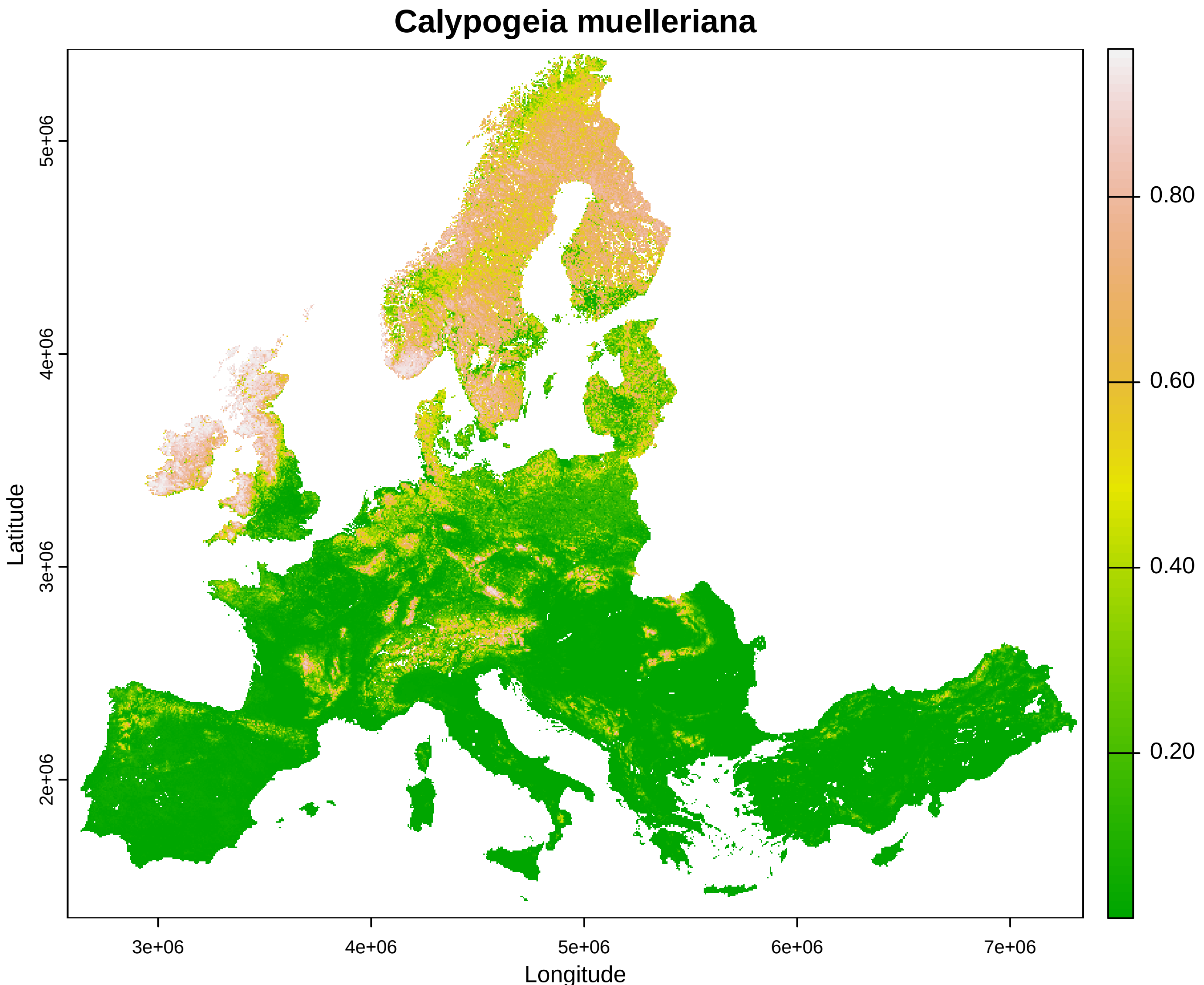

Calypogeia muelleriana
Latitude
Longitude
5e+06
4e+06
3e+06
2e+06
3e+06
4e+06
5e+06
6e+06
7e+06
0.80
0.60
0.40
0.20

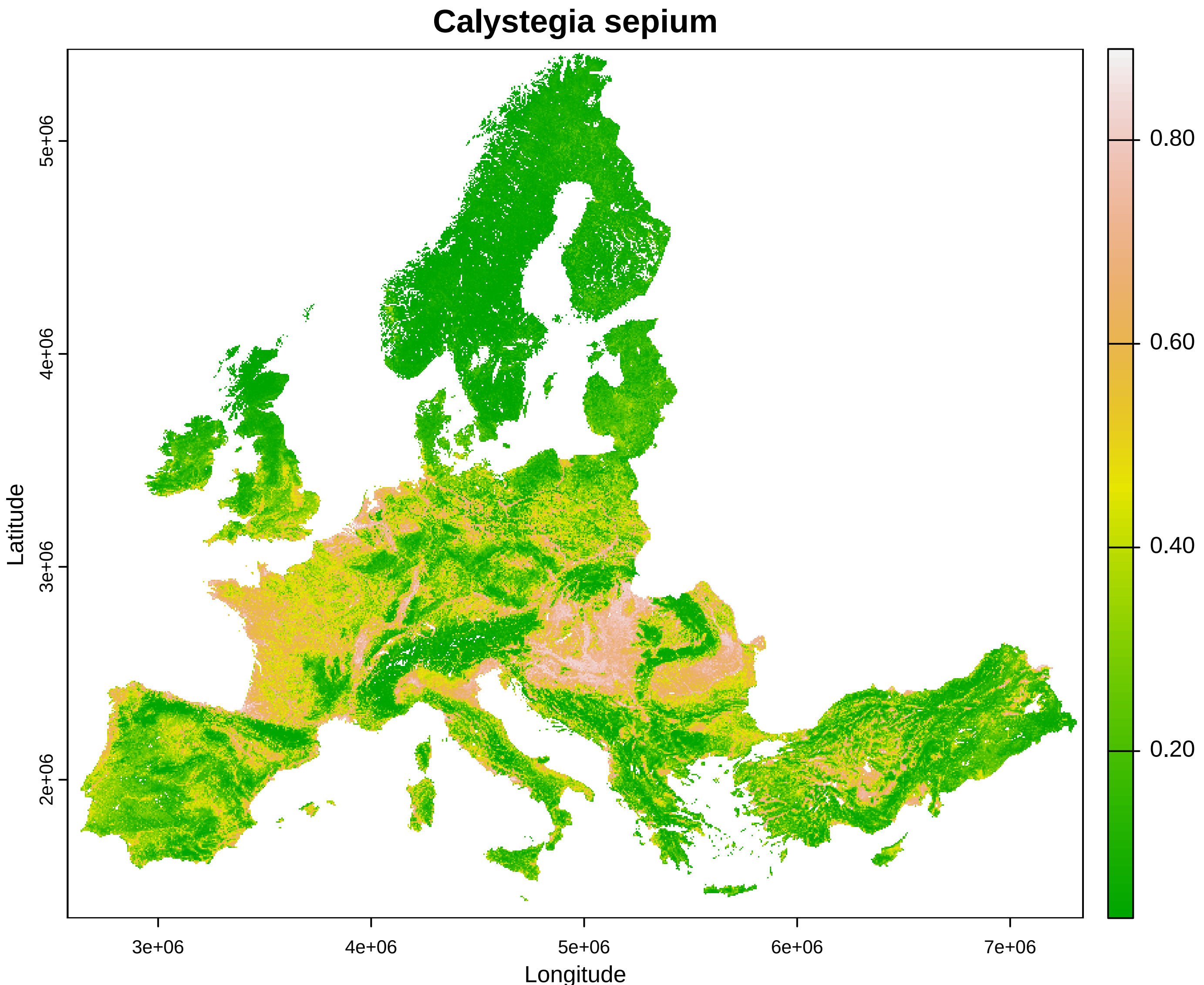

Calystegia sepium
Latitude
5e+06
4e+06
3e+06
2e+06
3e+06
4e+06
5e+06
6e+06
7e+06
Longitude
0.80
0.60
0.40
0.20

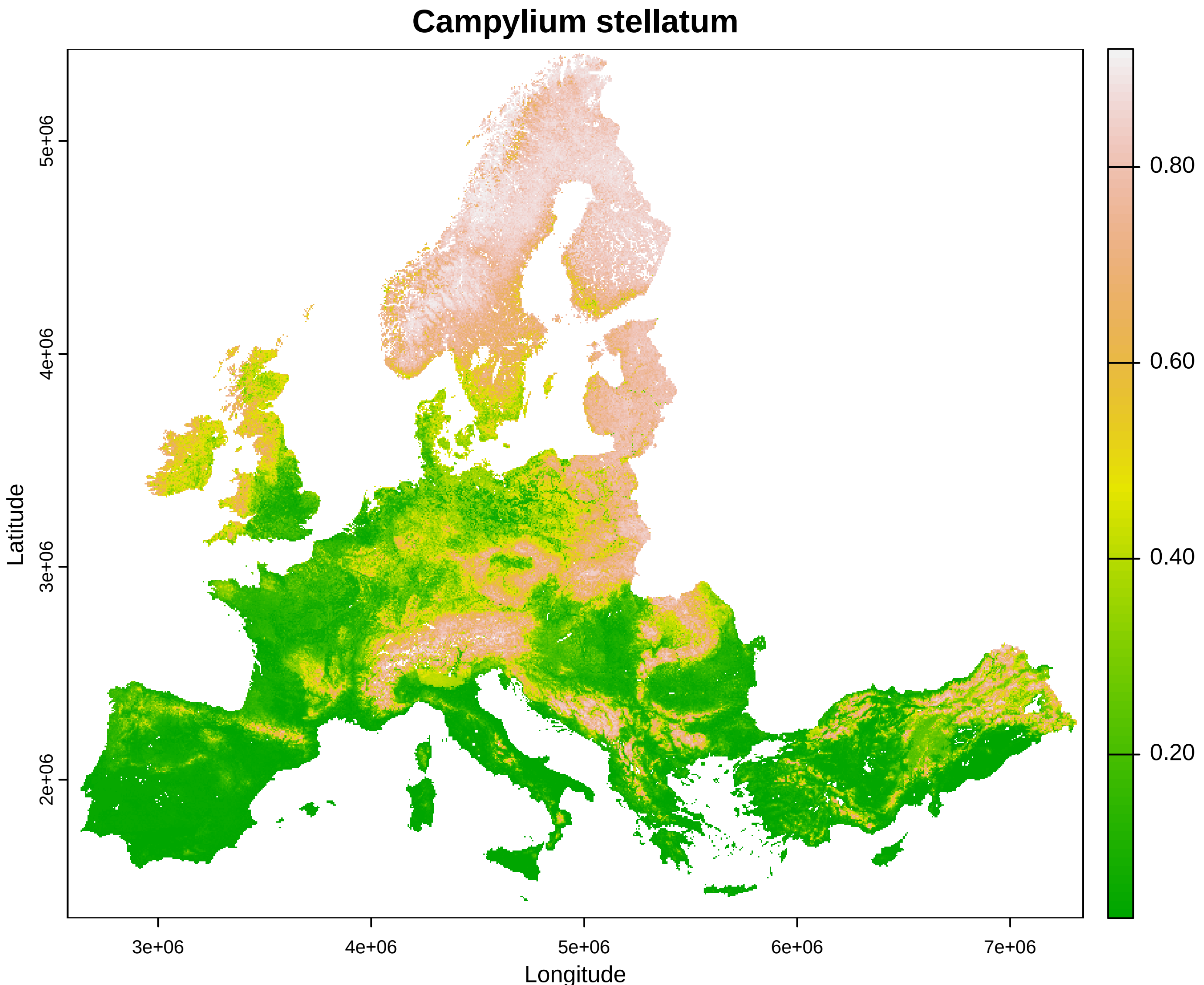
Campylium stellatum
Latitude
Longitude
5e+06
4e+06
3e+06
2e+06
3e+06
4e+06
5e+06
6e+06
7e+06
0.80
0.60
0.40
0.20

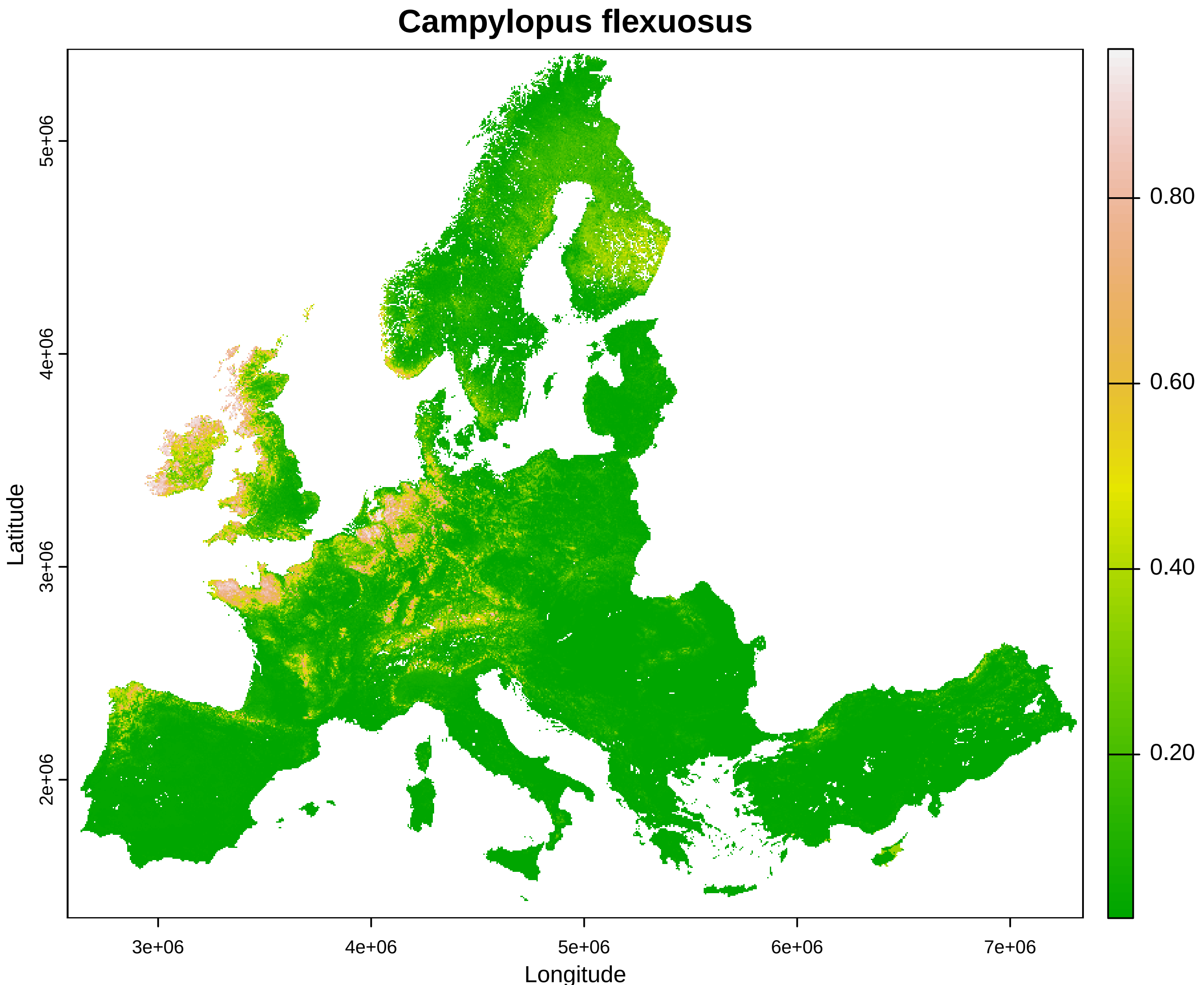

Campylopus flexuosus
Latitude
Longitude
5e+06
4e+06
3e+06
2e+06
3e+06
4e+06
5e+06
6e+06
7e+06
0.80
0.60
0.40
0.20

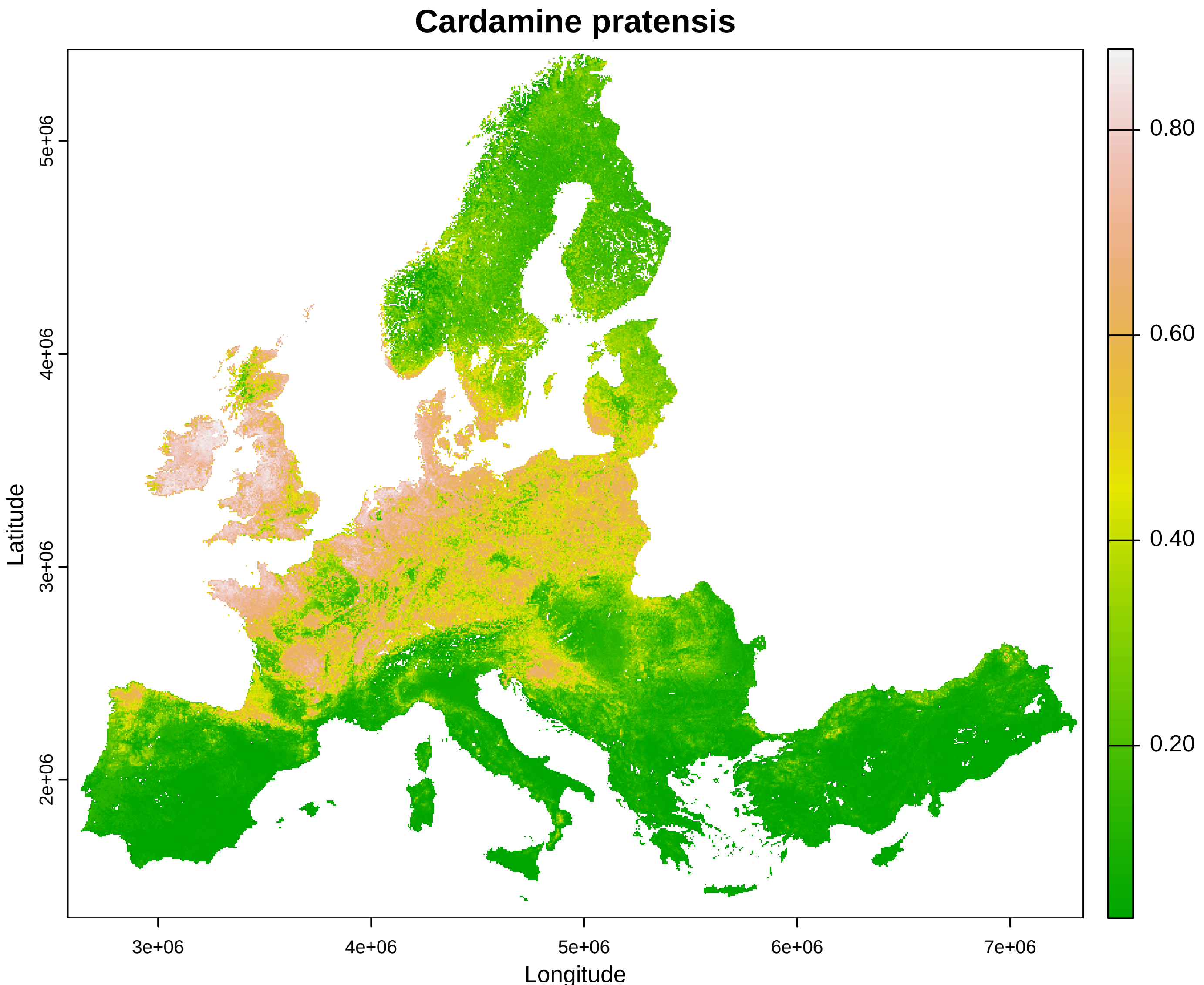

Cardamine pratensis
Latitude
Longitude
5e+06
4e+06
3e+06
2e+06
3e+06
4e+06
5e+06
6e+06
7e+06
0.80
0.60
0.40
0.20

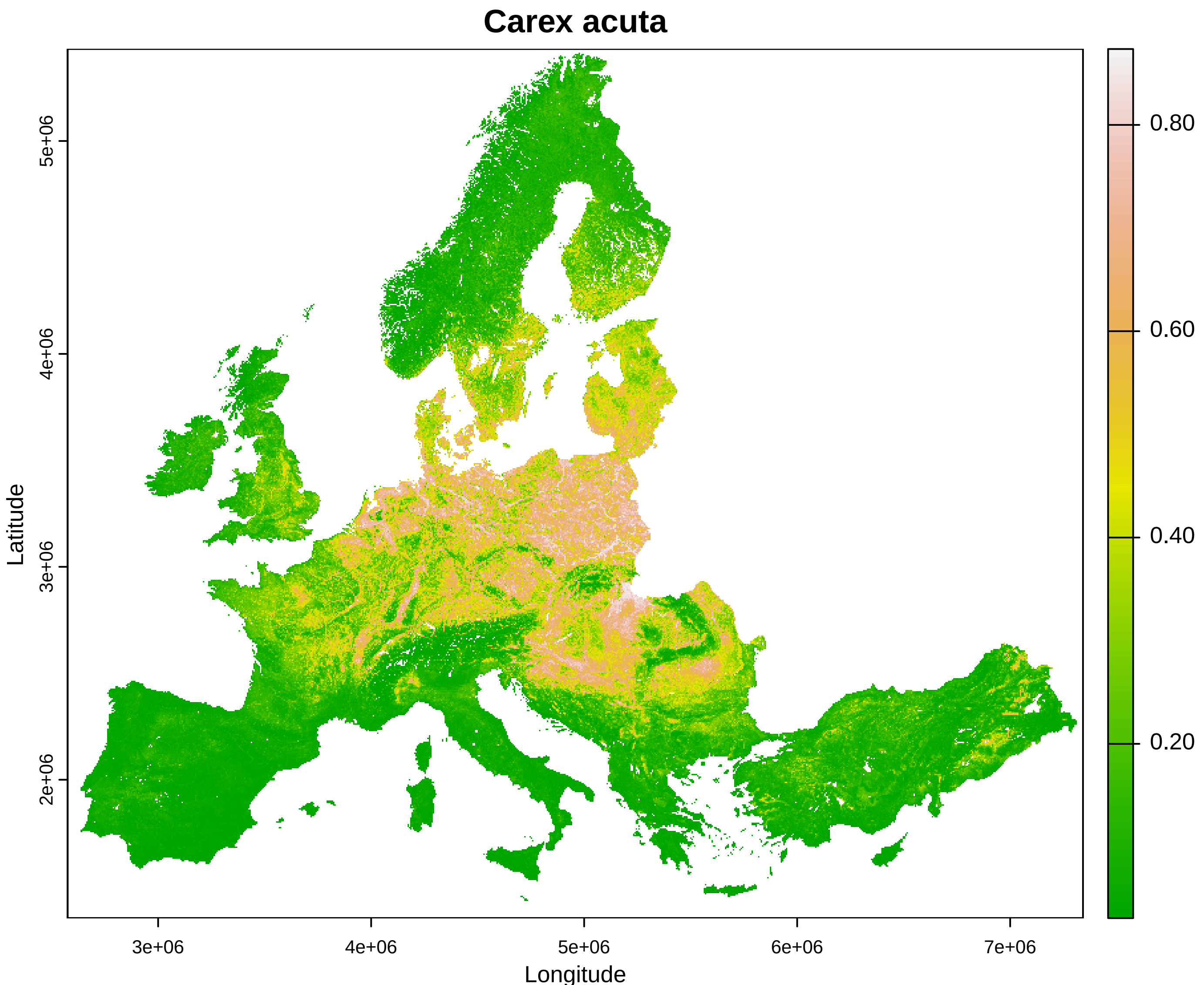
Carex acuta
Latitude
Longitude
5e+06
4e+06
3e+06
2e+06
3e+06
4e+06
5e+06
6e+06
7e+06
0.80
0.60
0.40
0.20

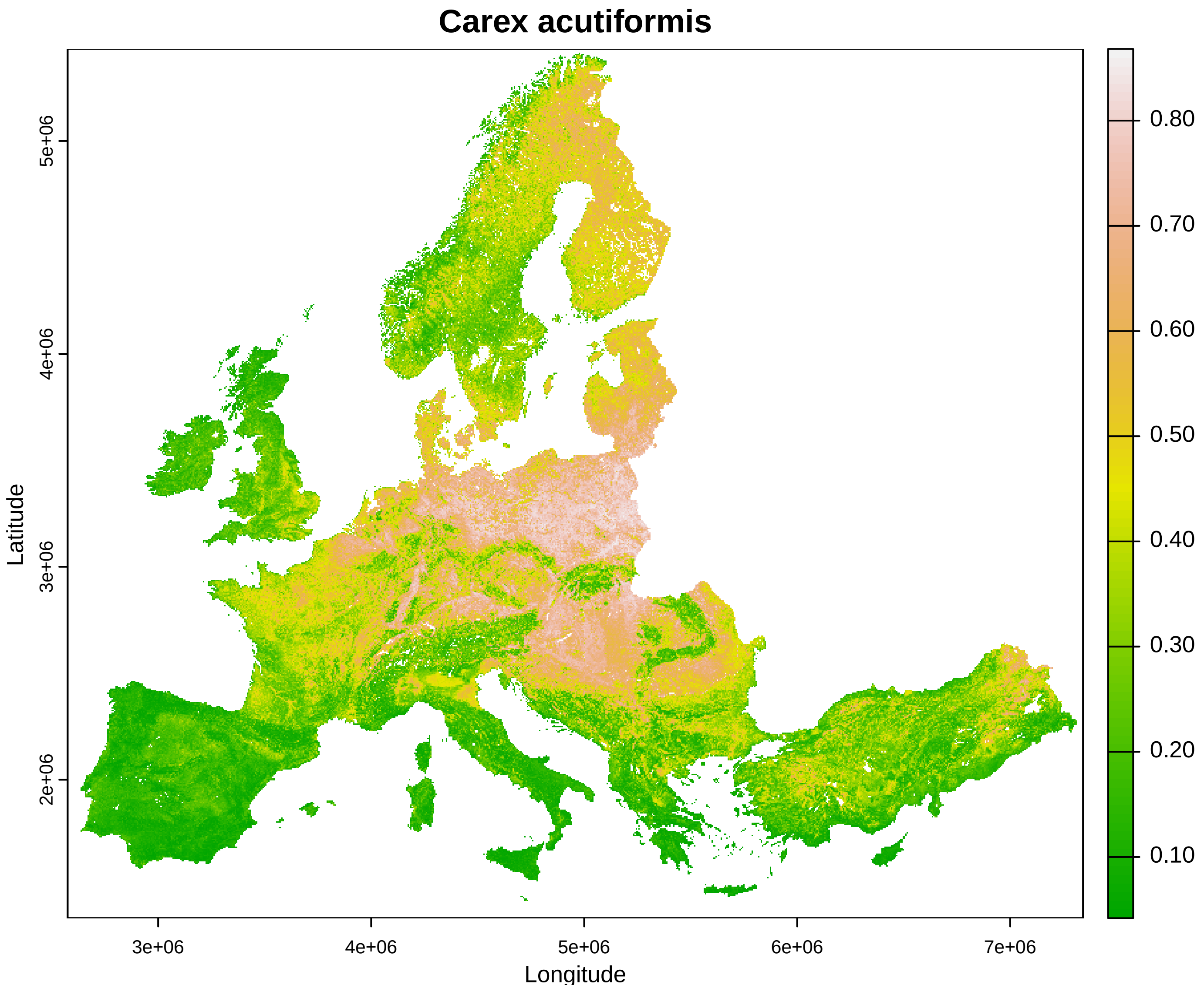

Carex acutiformis
Latitude
5e+06
4e+06
3e+06
2e+06
Longitude
3e+06
4e+06
5e+06
6e+06
7e+06
0.80
0.70
0.60
0.50
0.40
0.30
0.20
0.10

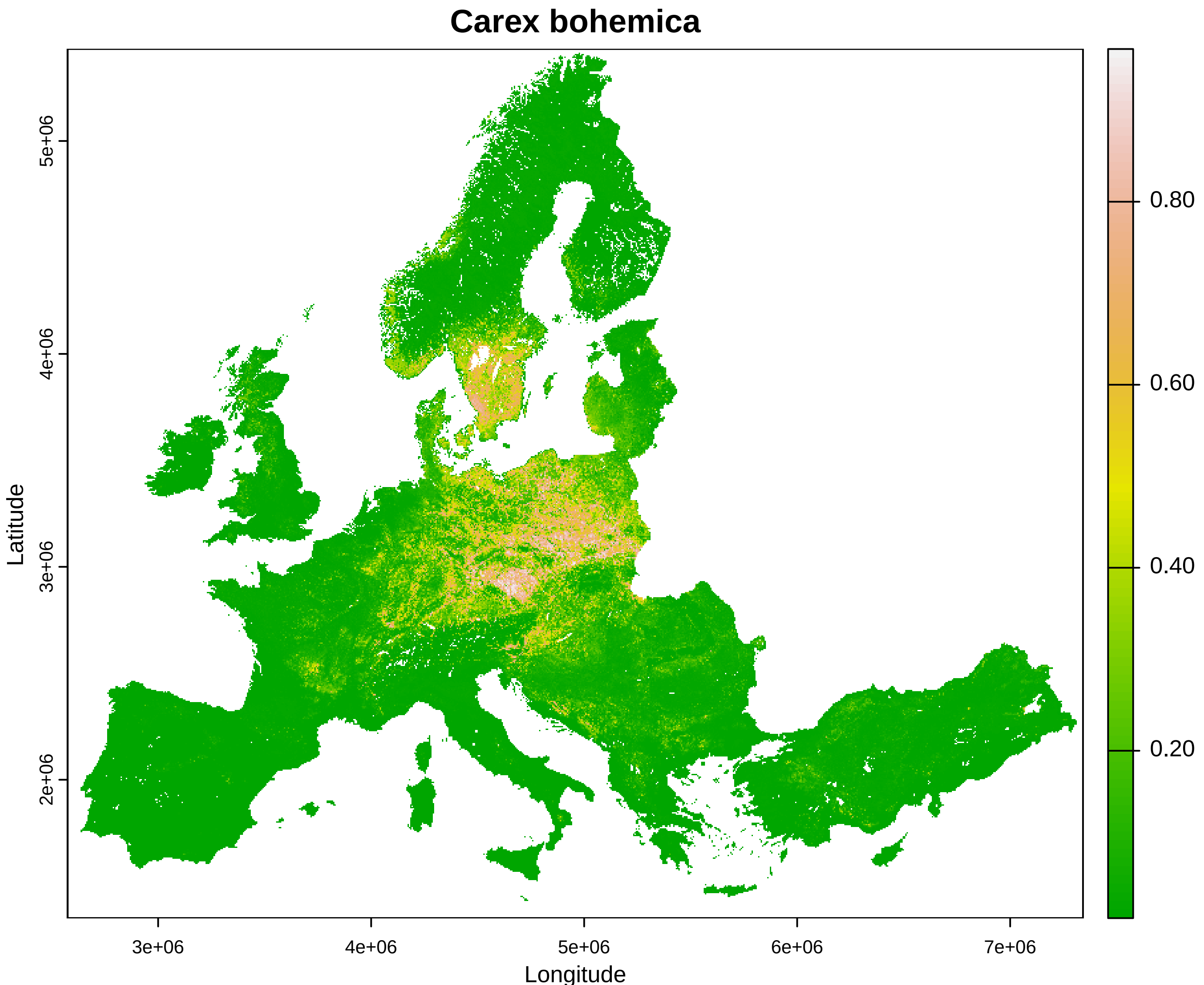
Carex bohemica
Latitude
Longitude
5e+06
4e+06
3e+06
2e+06
3e+06
4e+06
5e+06
6e+06
7e+06
0.80
0.60
0.40
0.20

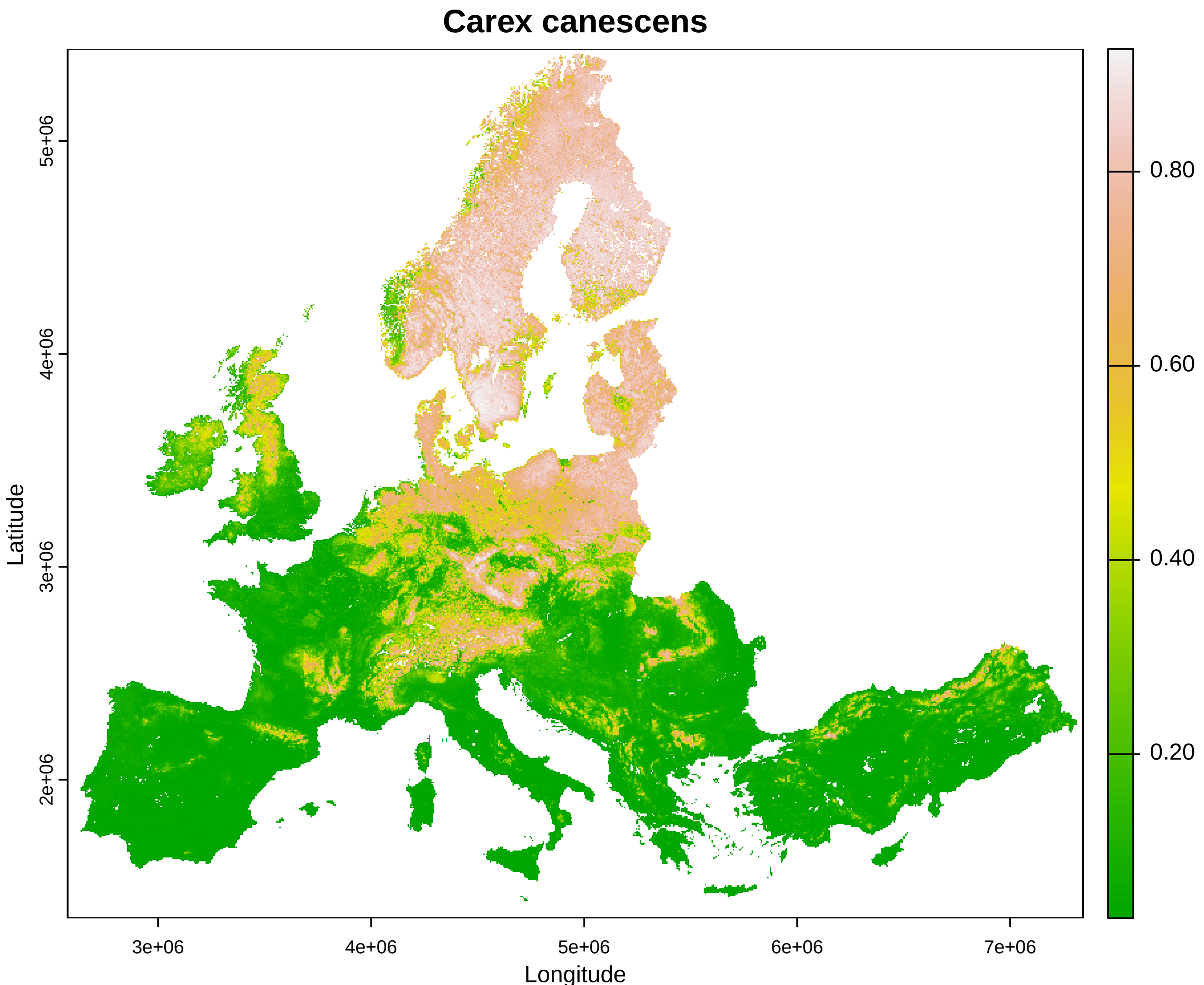
Carex canescens
Latitude
Longitude
5e+06
4e+06
3e+06
2e+06
3e+06
4e+06
5e+06
6e+06
7e+06
0.80
0.60
0.40
0.20

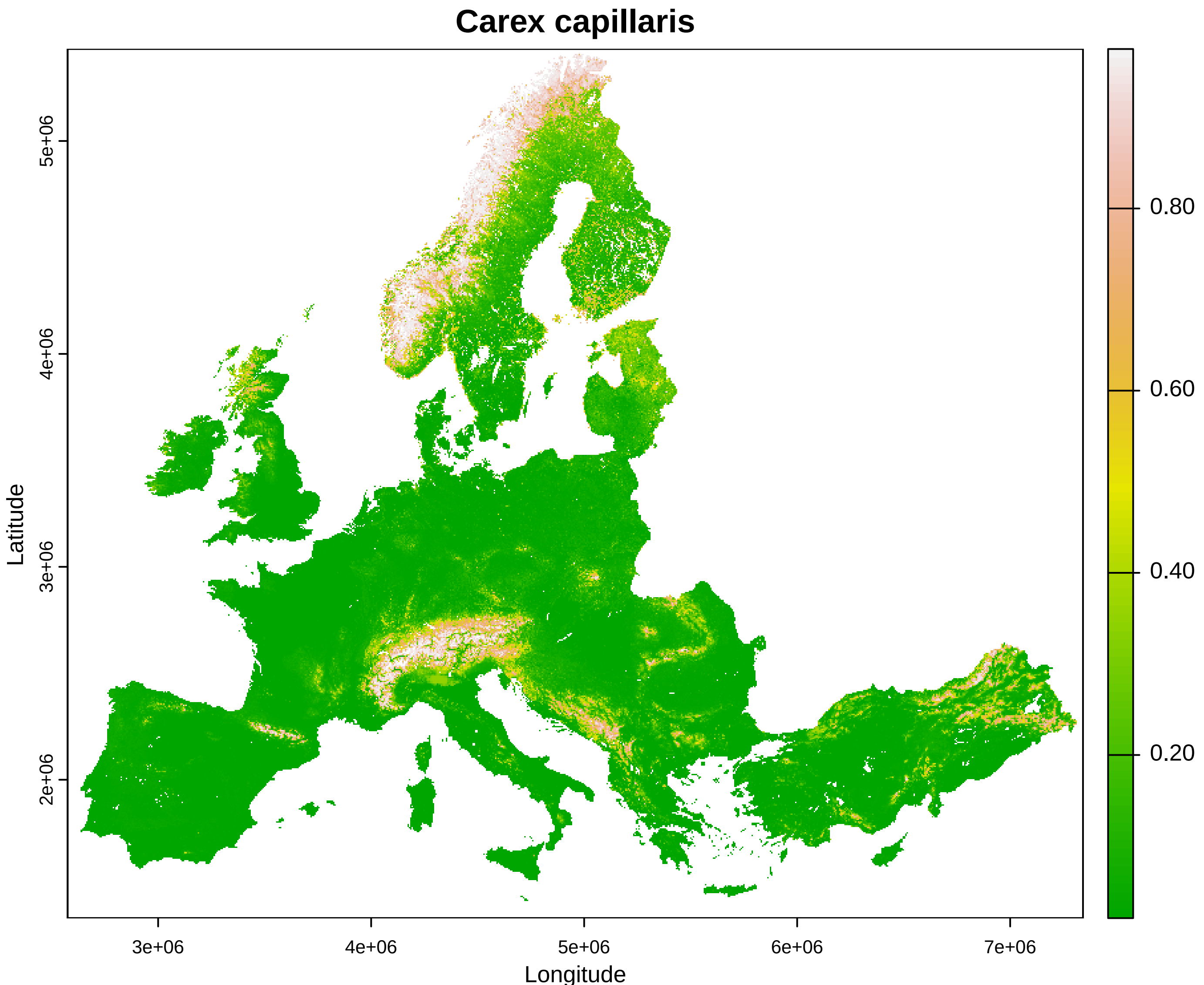
Carex capillaris
Latitude
Longitude
5e+06
4e+06
3e+06
2e+06
3e+06
4e+06
5e+06
6e+06
7e+06
0.80
0.60
0.40
0.20

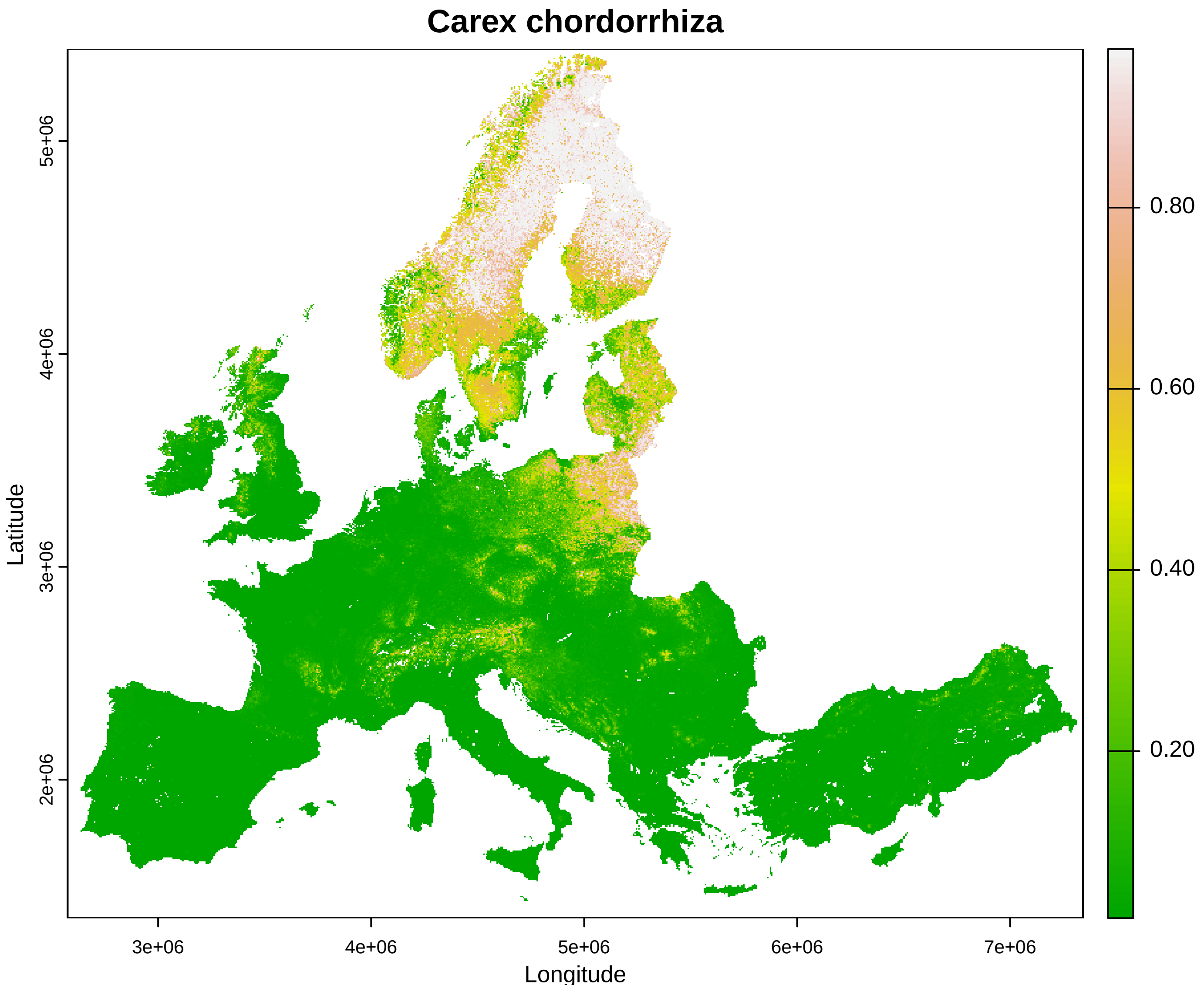

Carex chordorrhiza
Latitude
Longitude
5e+06
4e+06
3e+06
2e+06
3e+06
4e+06
5e+06
6e+06
7e+06
0.80
0.60
0.40
0.20

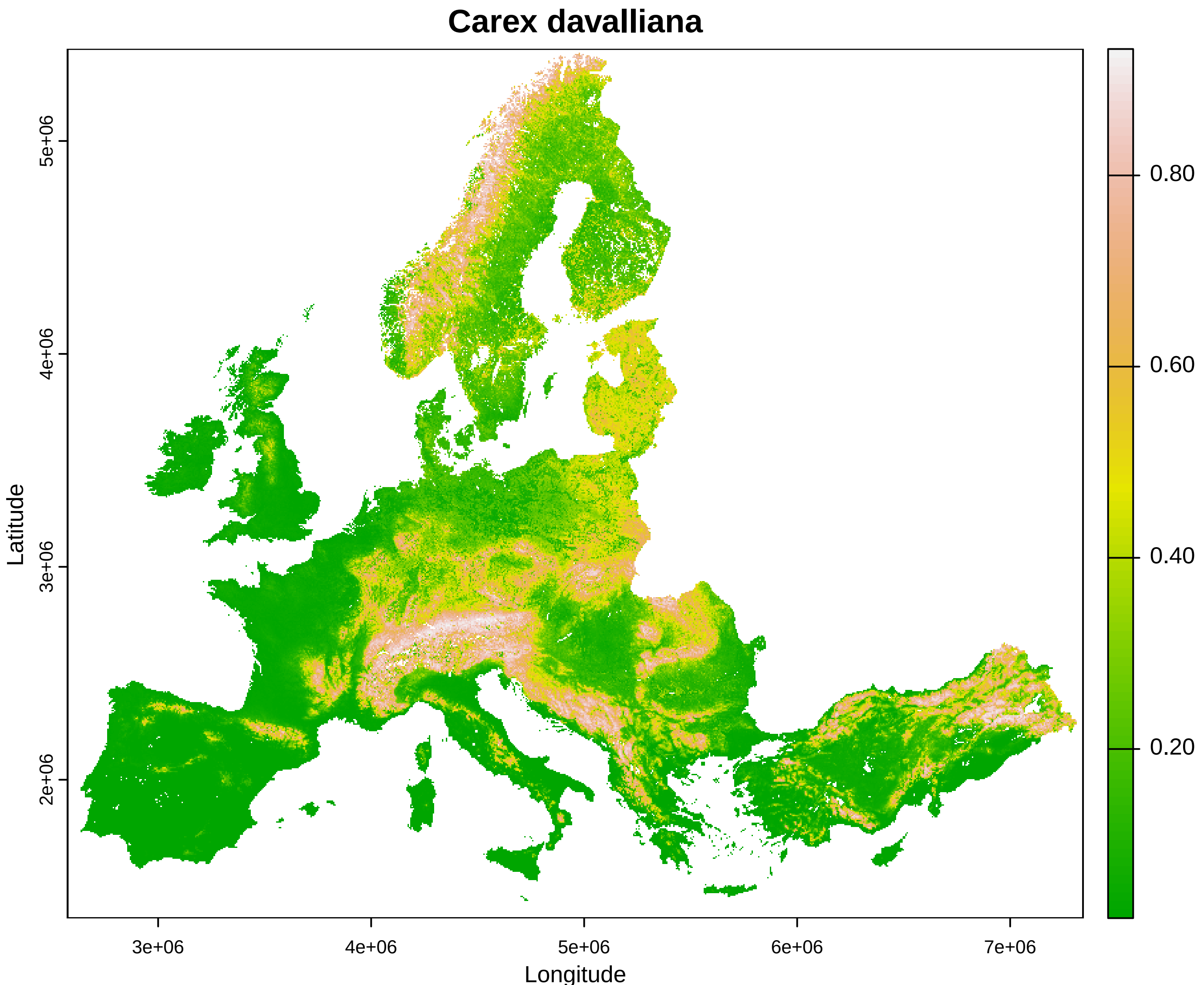
Carex davalliana
Latitude
Longitude
5e+06
4e+06
3e+06
2e+06
3e+06
4e+06
5e+06
6e+06
7e+06
0.80
0.60
0.40
0.20

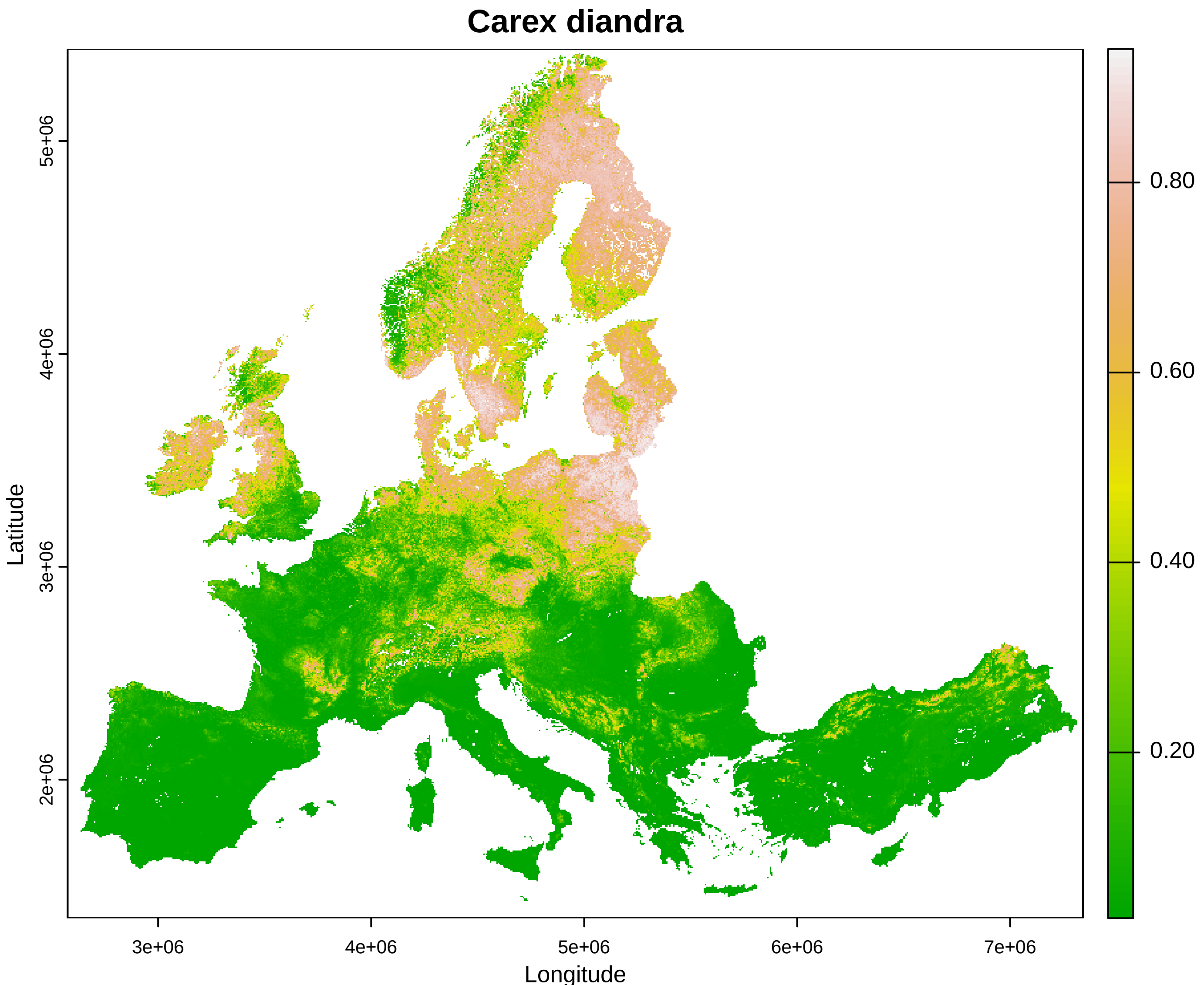

Carex diandra
Latitude
Longitude
5e+06
4e+06
3e+06
2e+06
3e+06
4e+06
5e+06
6e+06
7e+06
0.80
0.60
0.40
0.20

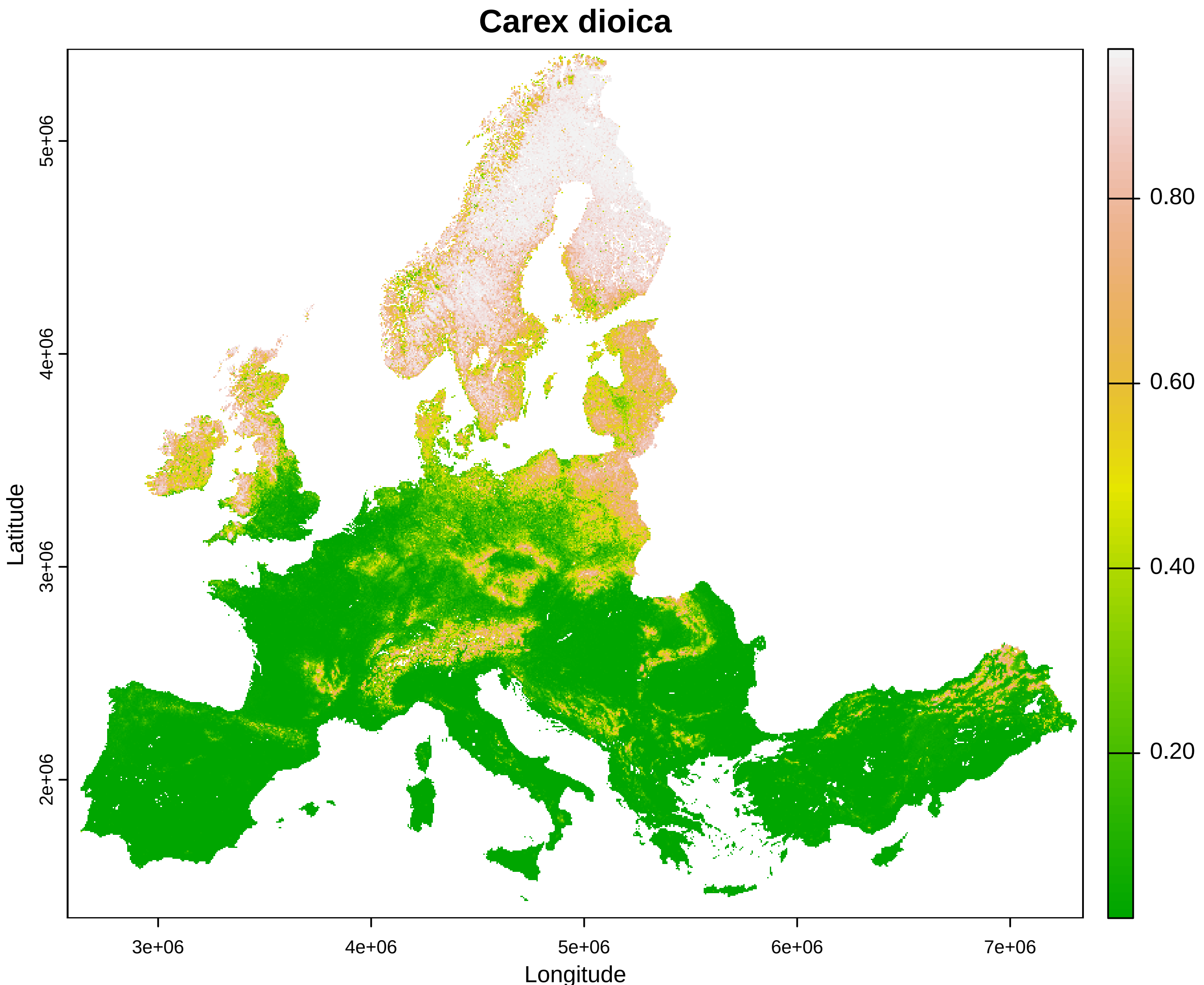

Carex dioica
Latitude
Longitude
5e+06
4e+06
3e+06
2e+06
3e+06
4e+06
5e+06
6e+06
7e+06
0.80
0.60
0.40
0.20

Carex distans

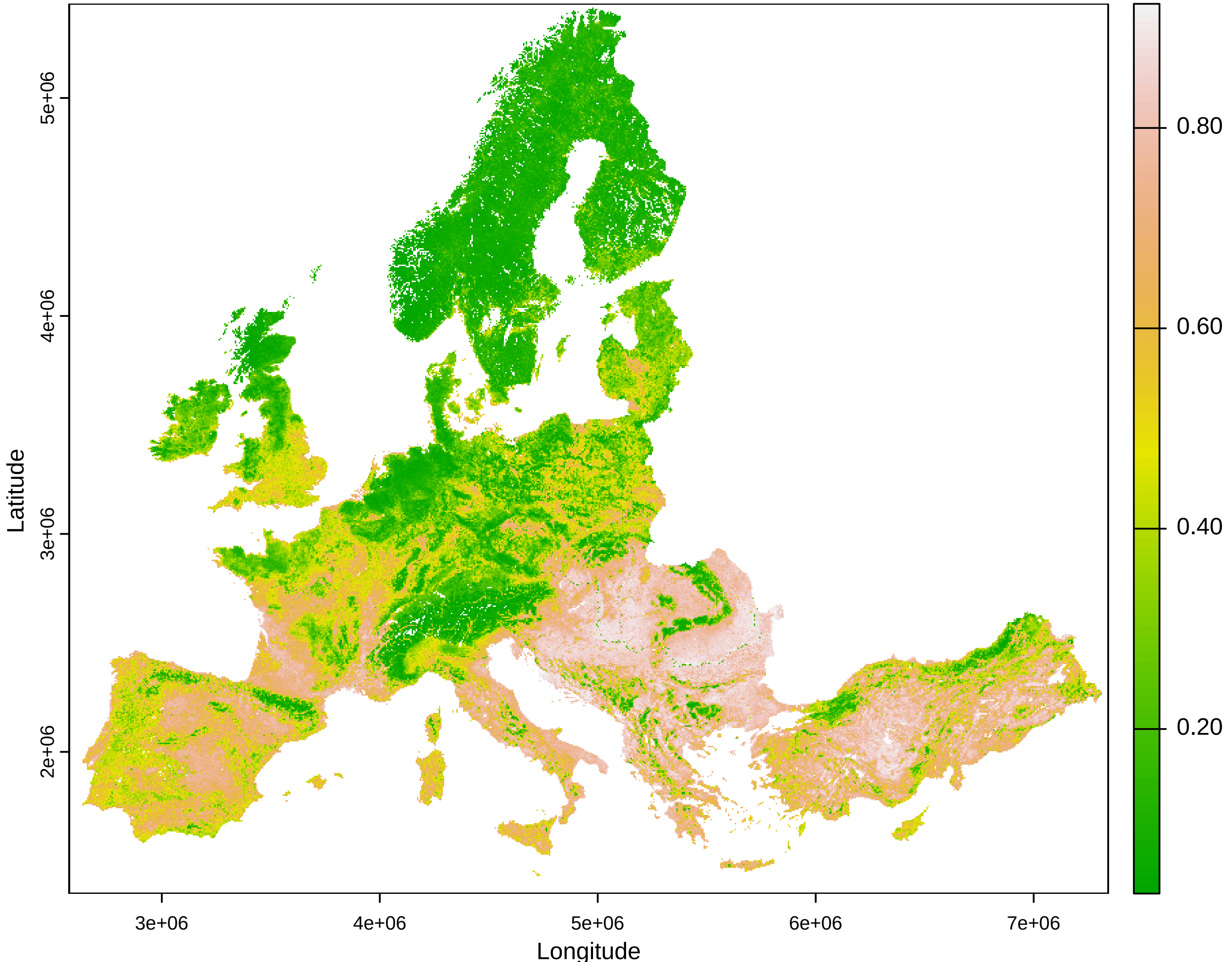

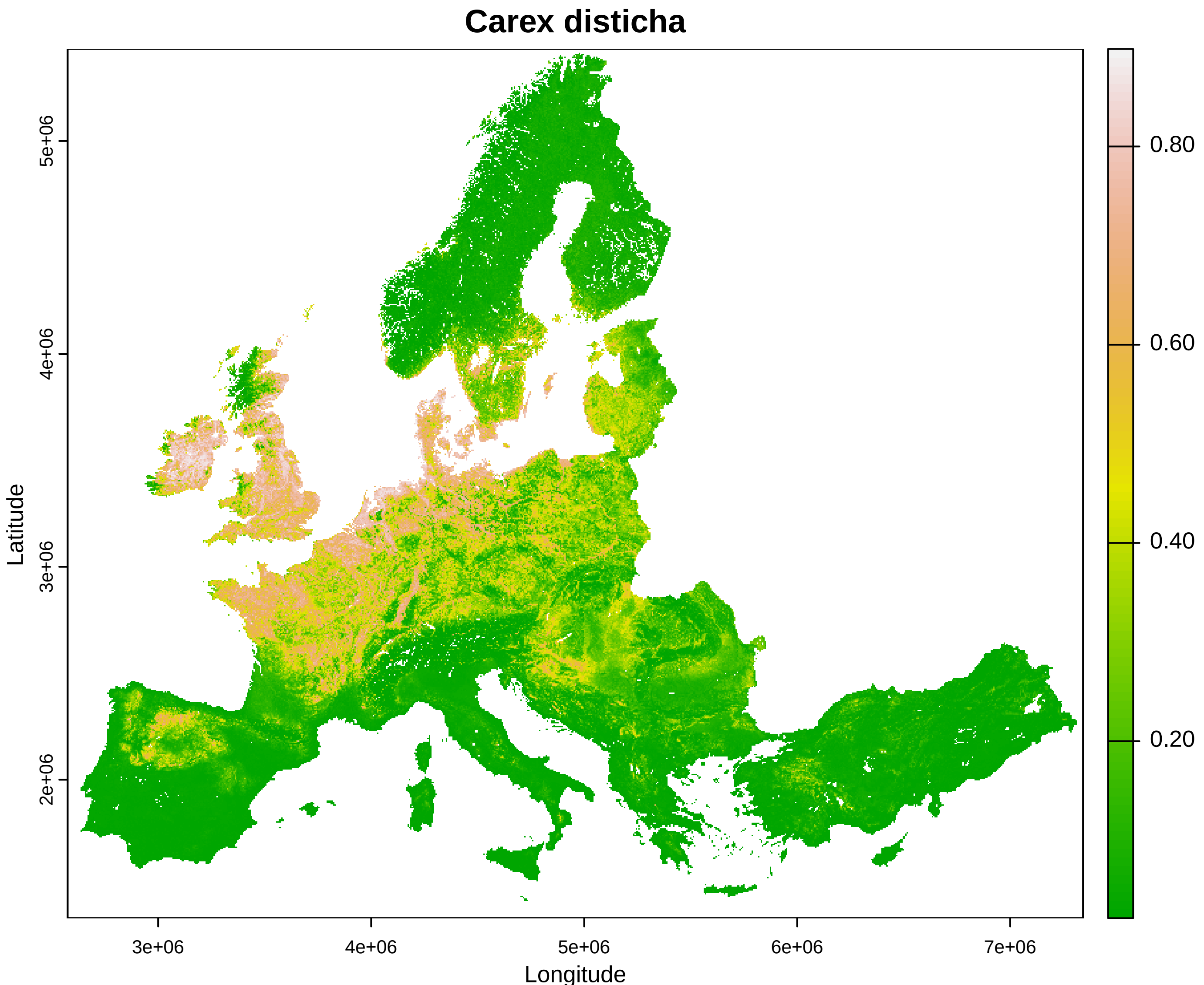

Carex disticha
Latitude
Longitude
5e+06
4e+06
3e+06
2e+06
3e+06
4e+06
5e+06
6e+06
7e+06
0.80
0.60
0.40
0.20

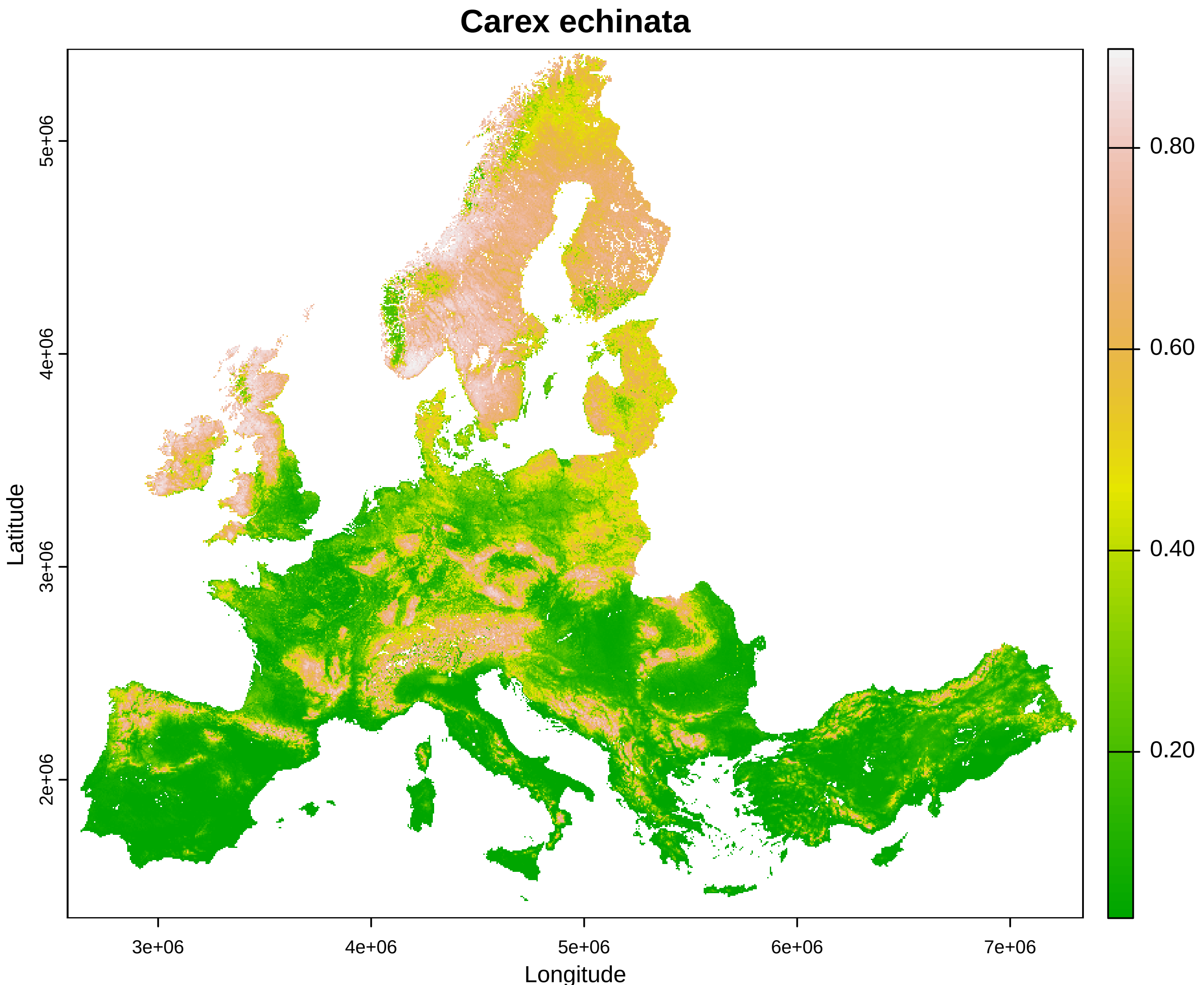
Carex echinata
Latitude
Longitude
5e+06
4e+06
3e+06
2e+06
3e+06
4e+06
5e+06
6e+06
7e+06
0.80
0.60
0.40
0.20

Carex elata

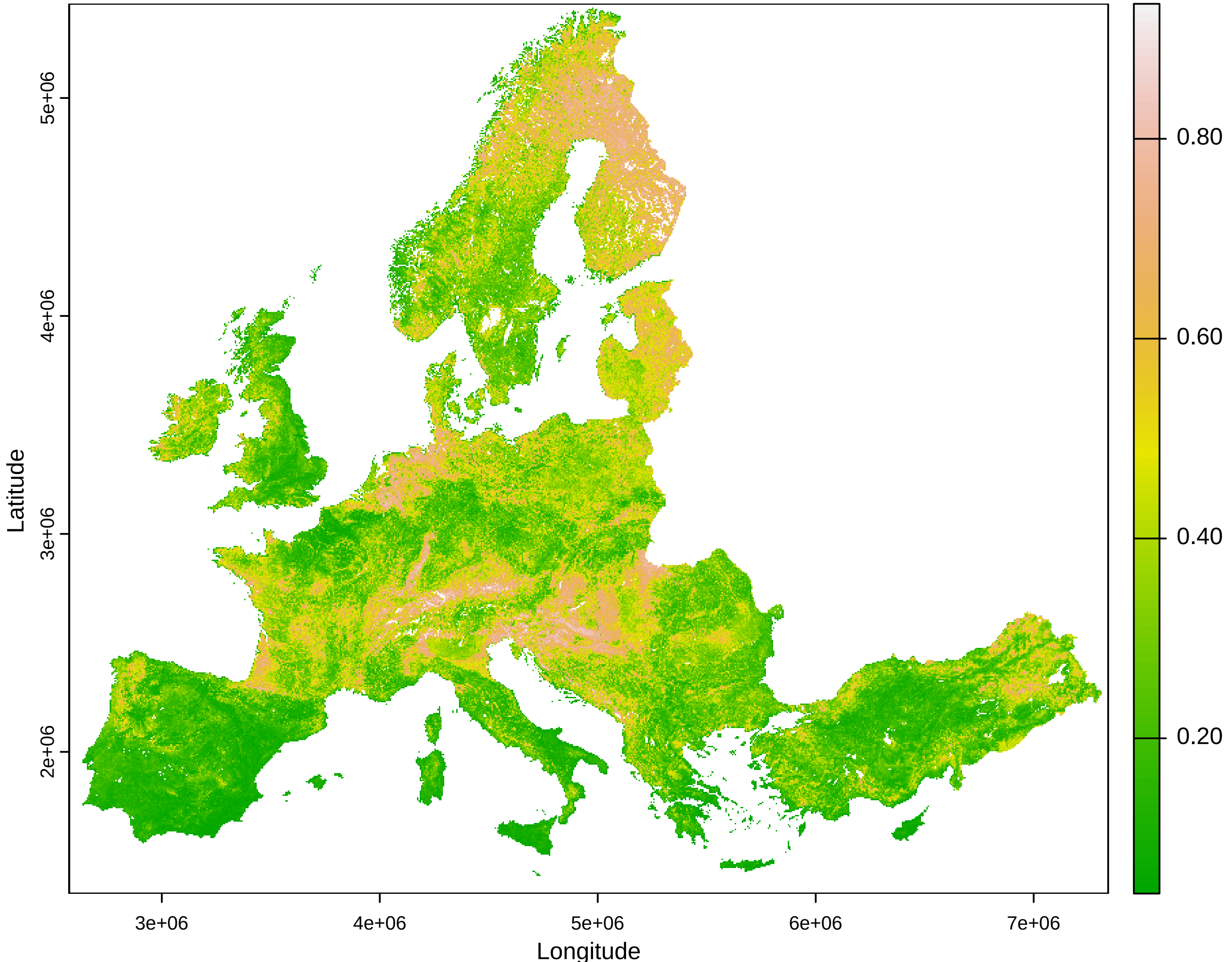

Latitude
5e+06
4e+06
3e+06
2e+06
3e+06
4e+06
5e+06
6e+06
7e+06
Longitude
0.80
0.60
0.40
0.20

Carex flacca

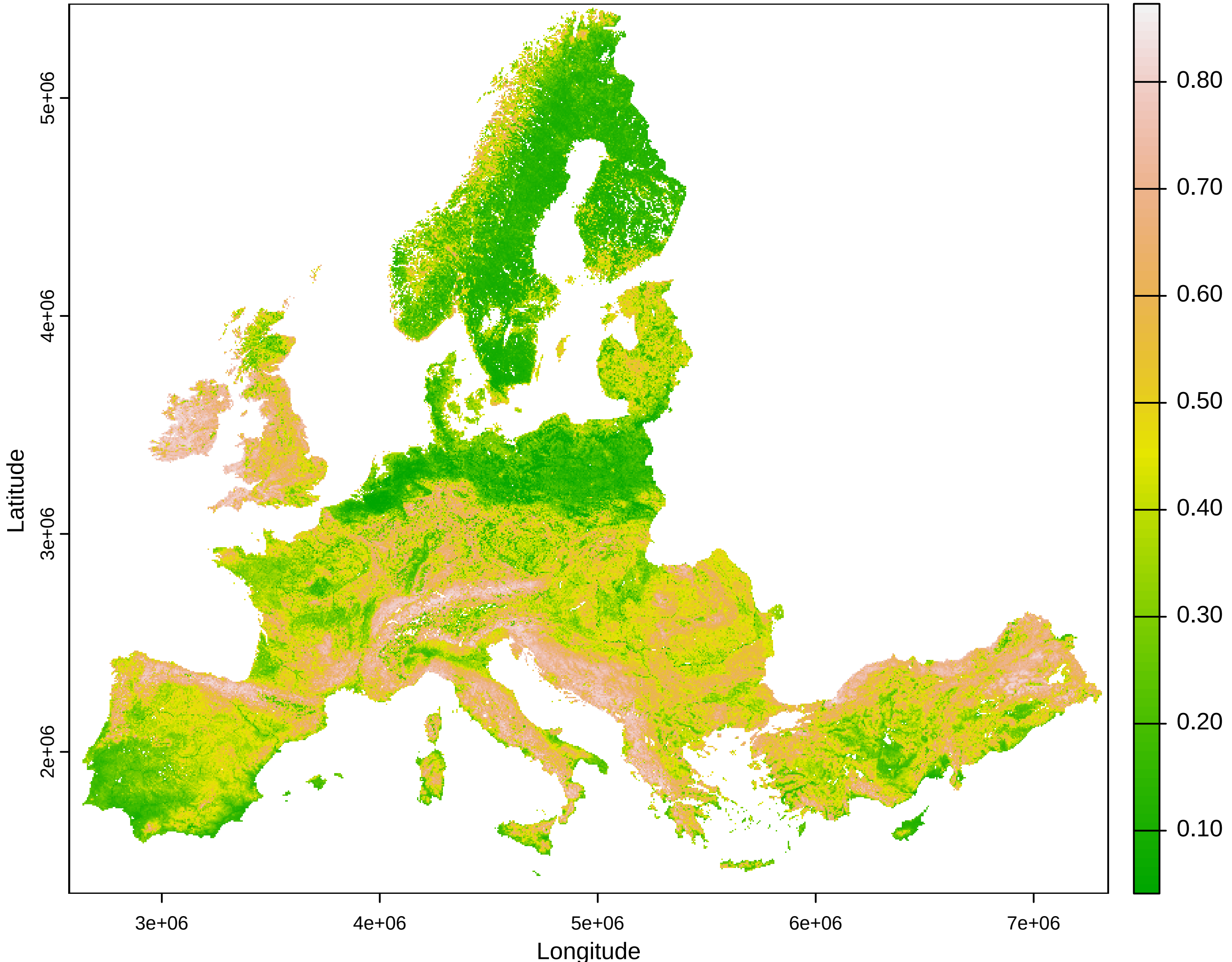

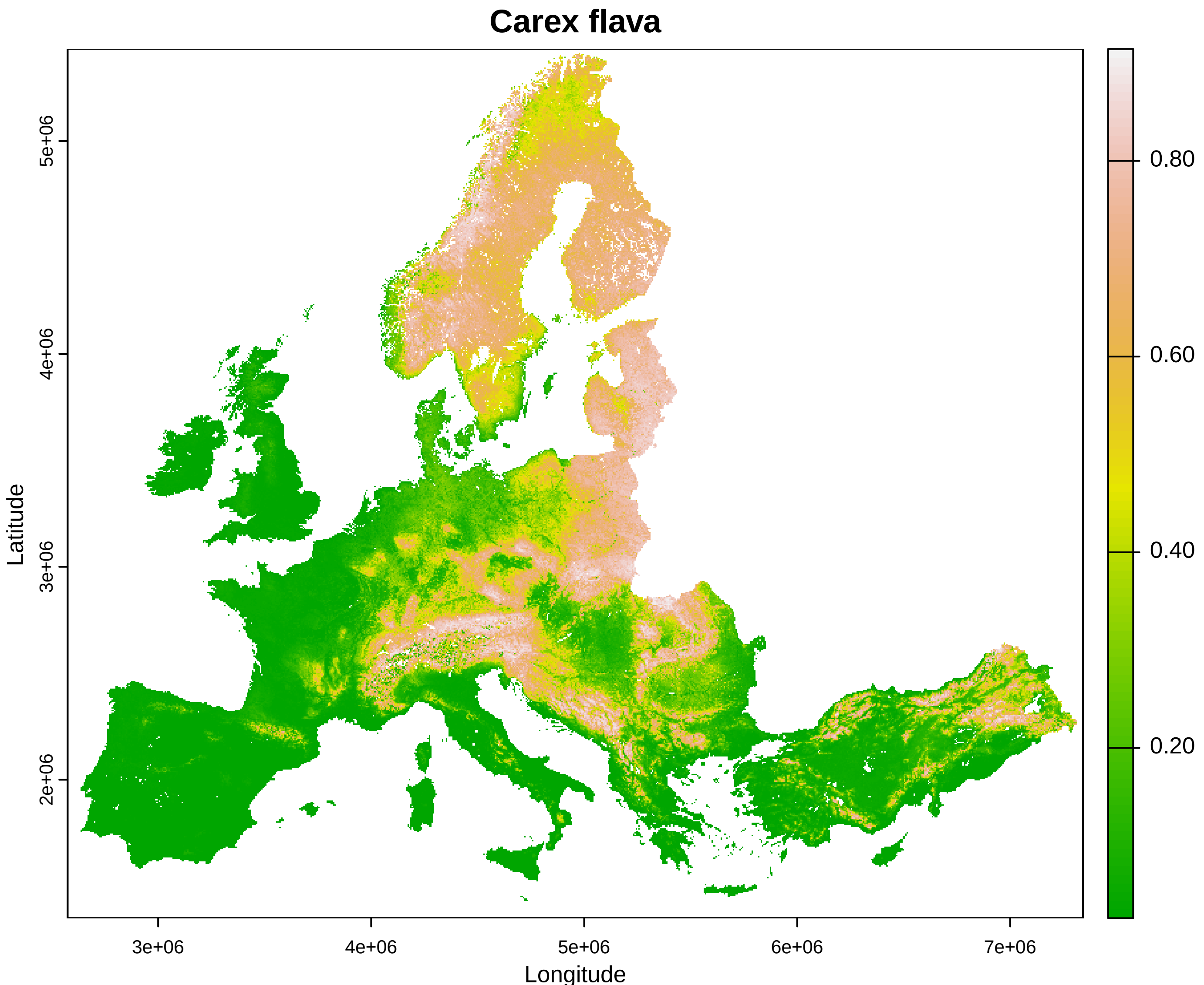
Carex flava
Latitude
Longitude
5e+06
4e+06
3e+06
2e+06
3e+06
4e+06
5e+06
6e+06
7e+06
0.80
0.60
0.40
0.20

Carex frigida

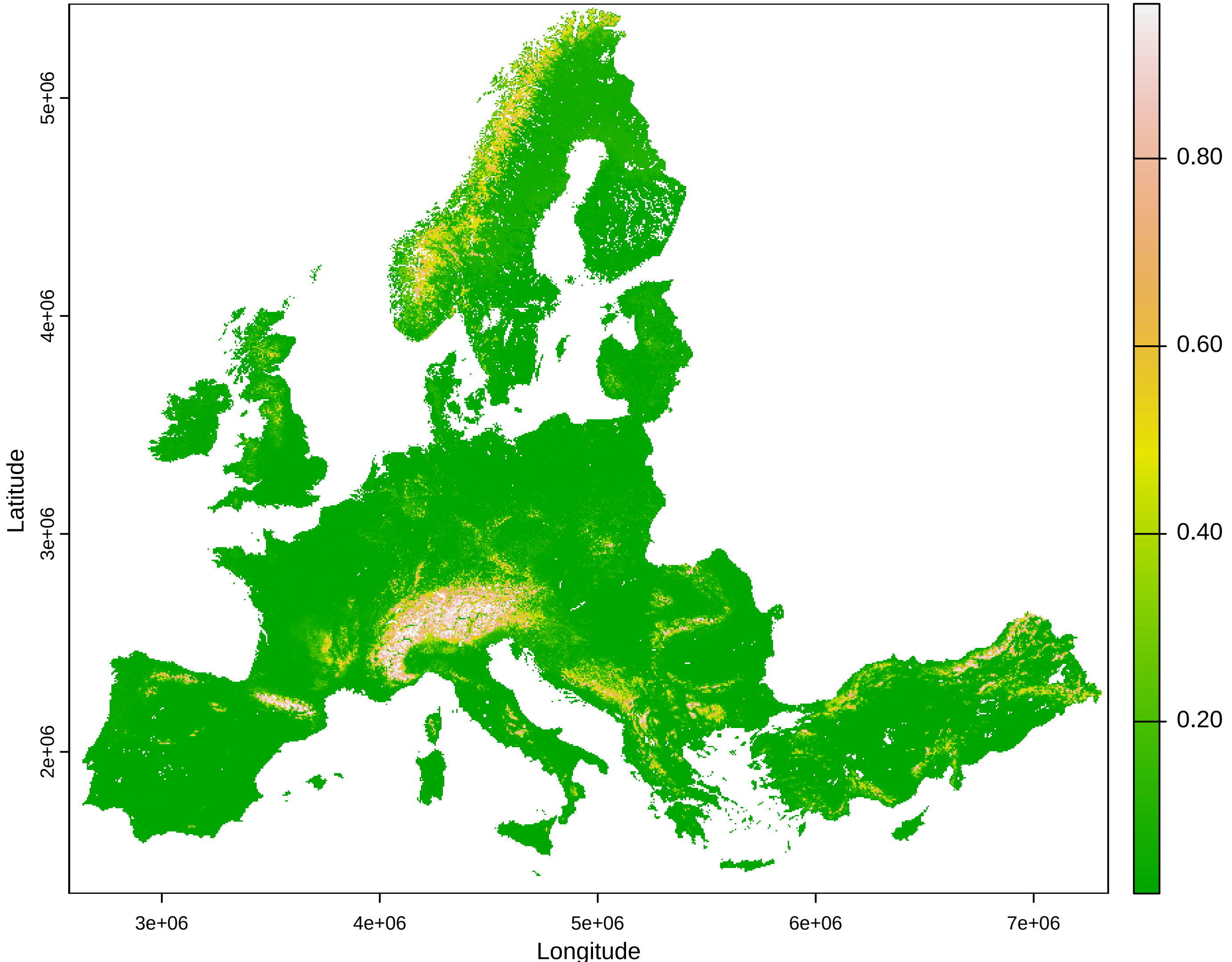

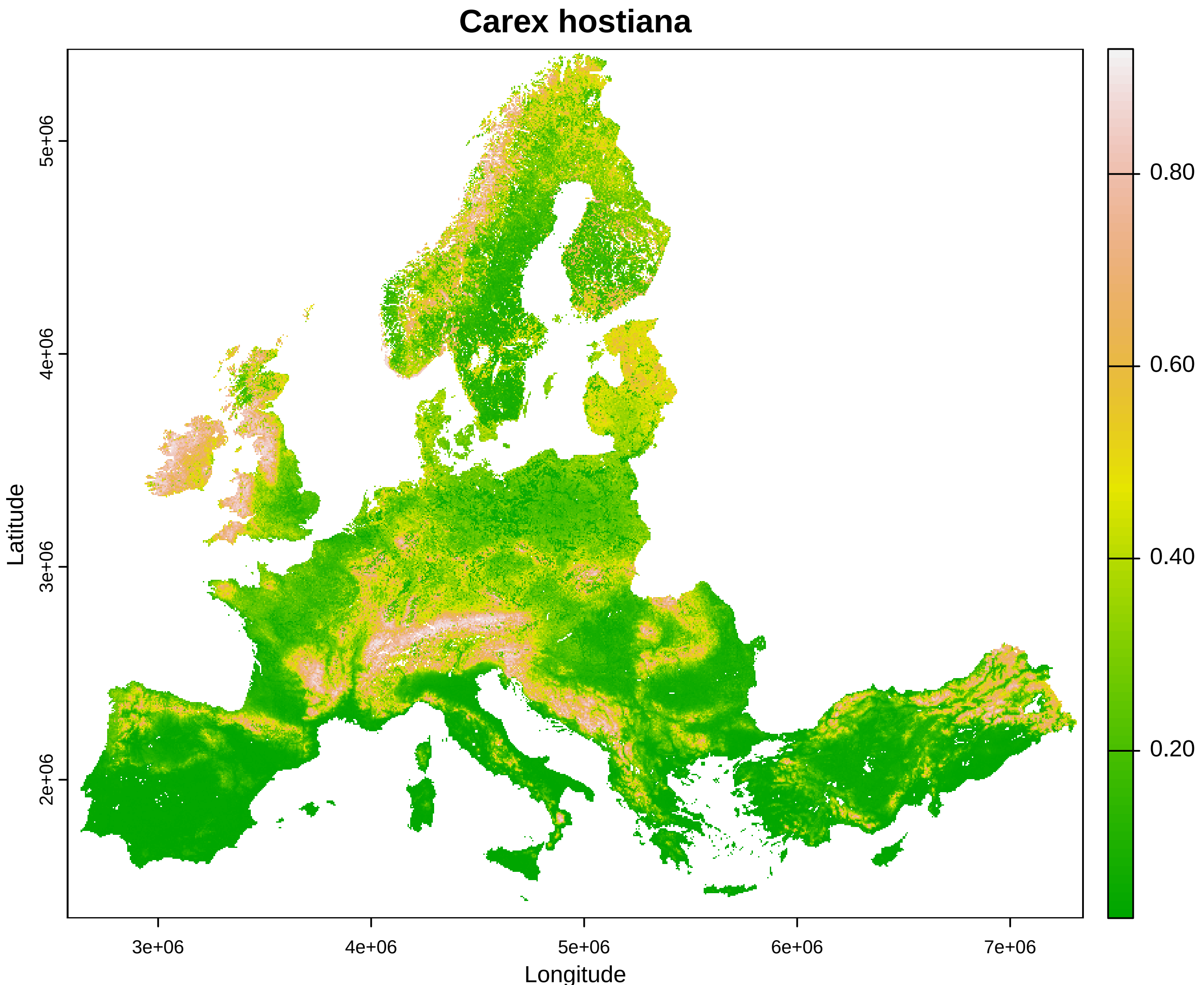
Carex hostiana
Latitude
5e+06
4e+06
3e+06
2e+06
3e+06
4e+06
5e+06
6e+06
7e+06
Longitude
0.80
0.60
0.40
0.20

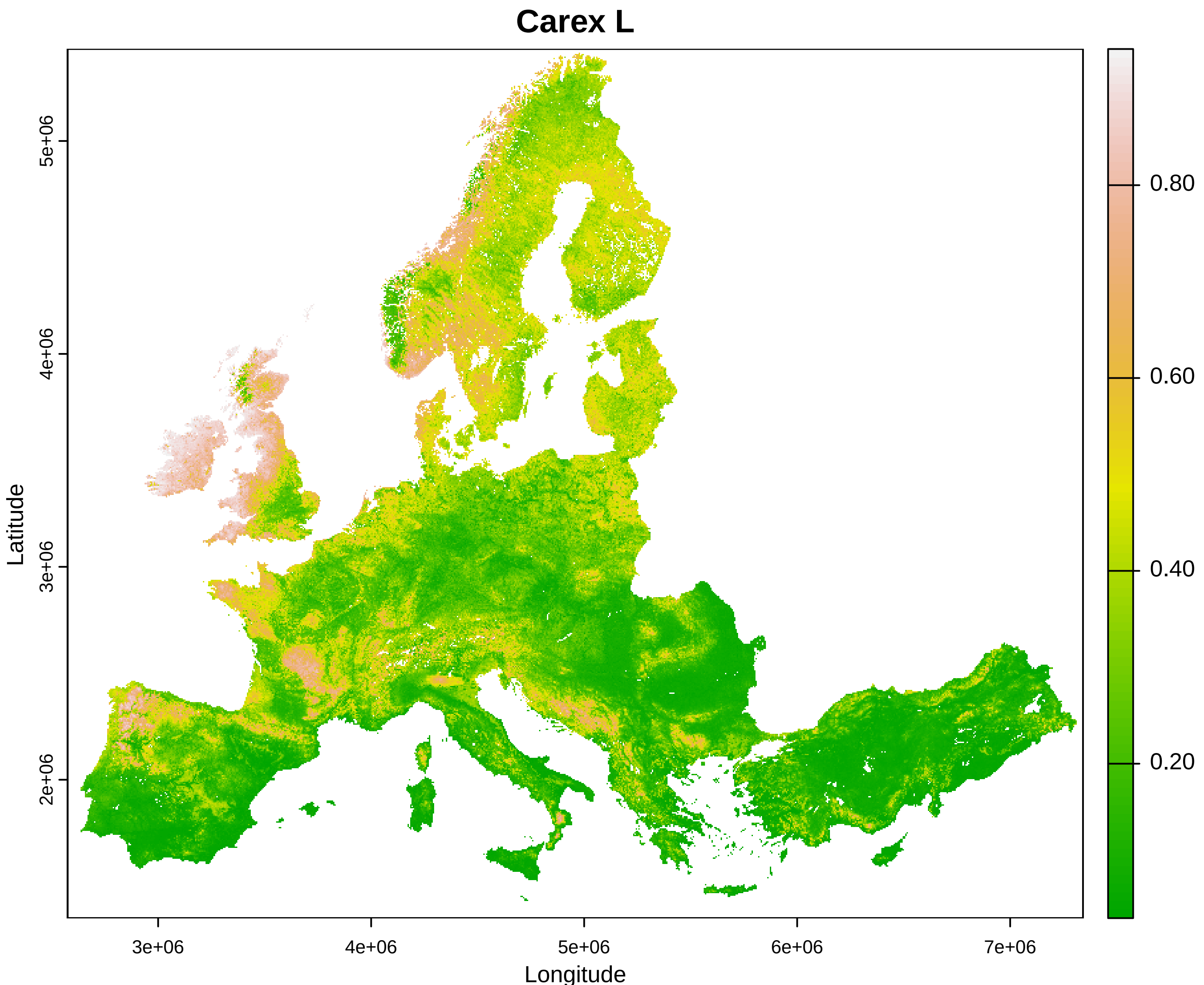
Carex L
Latitude
5e+06
4e+06
3e+06
2e+06
Longitude
3e+06
4e+06
5e+06
6e+06
7e+06
0.80
0.60
0.40
0.20

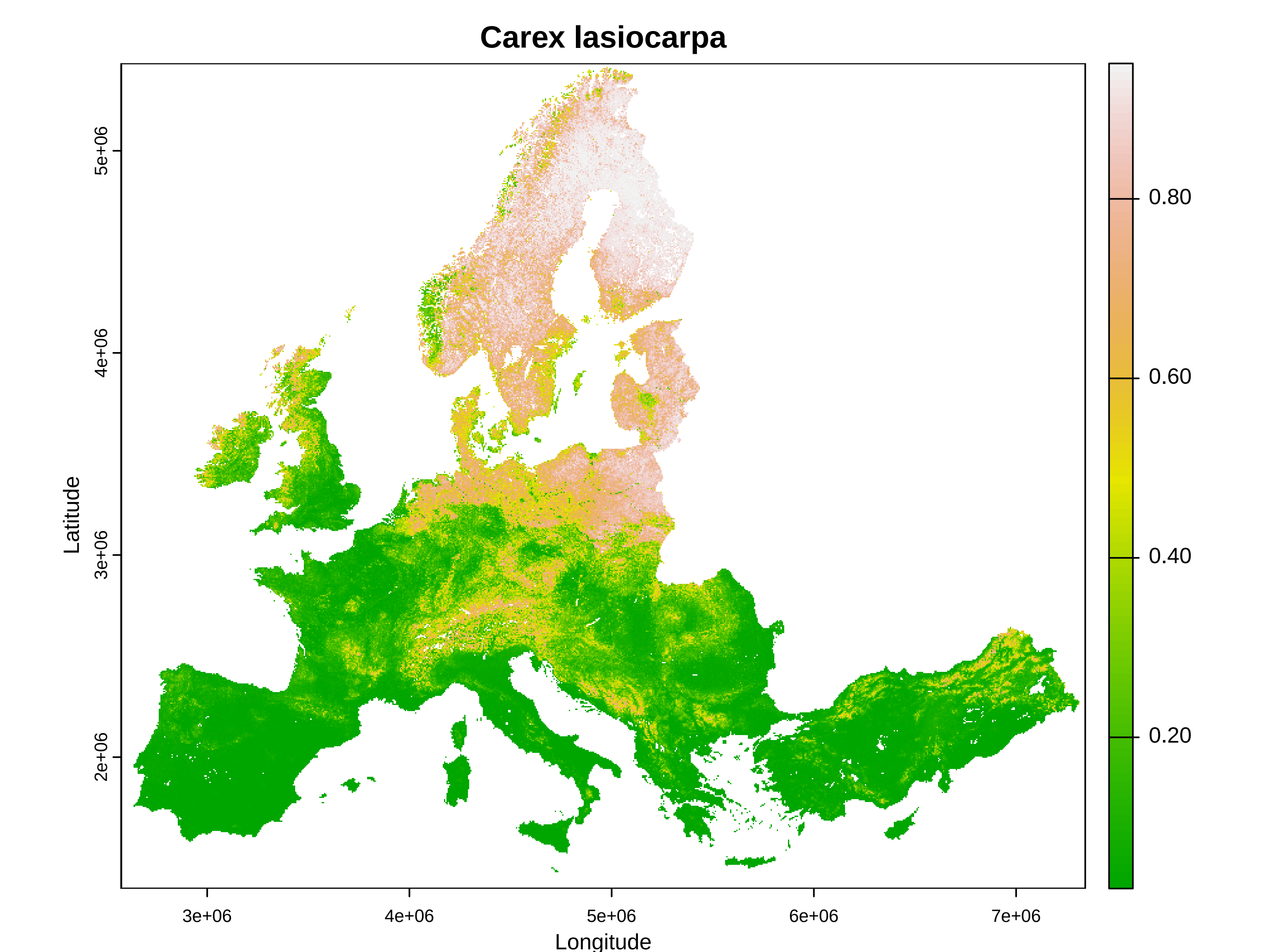
Carex lasiocarpa
Latitude
Longitude
5e+06
4e+06
3e+06
2e+06
3e+06
4e+06
5e+06
6e+06
7e+06
0.80
0.60
0.40
0.20

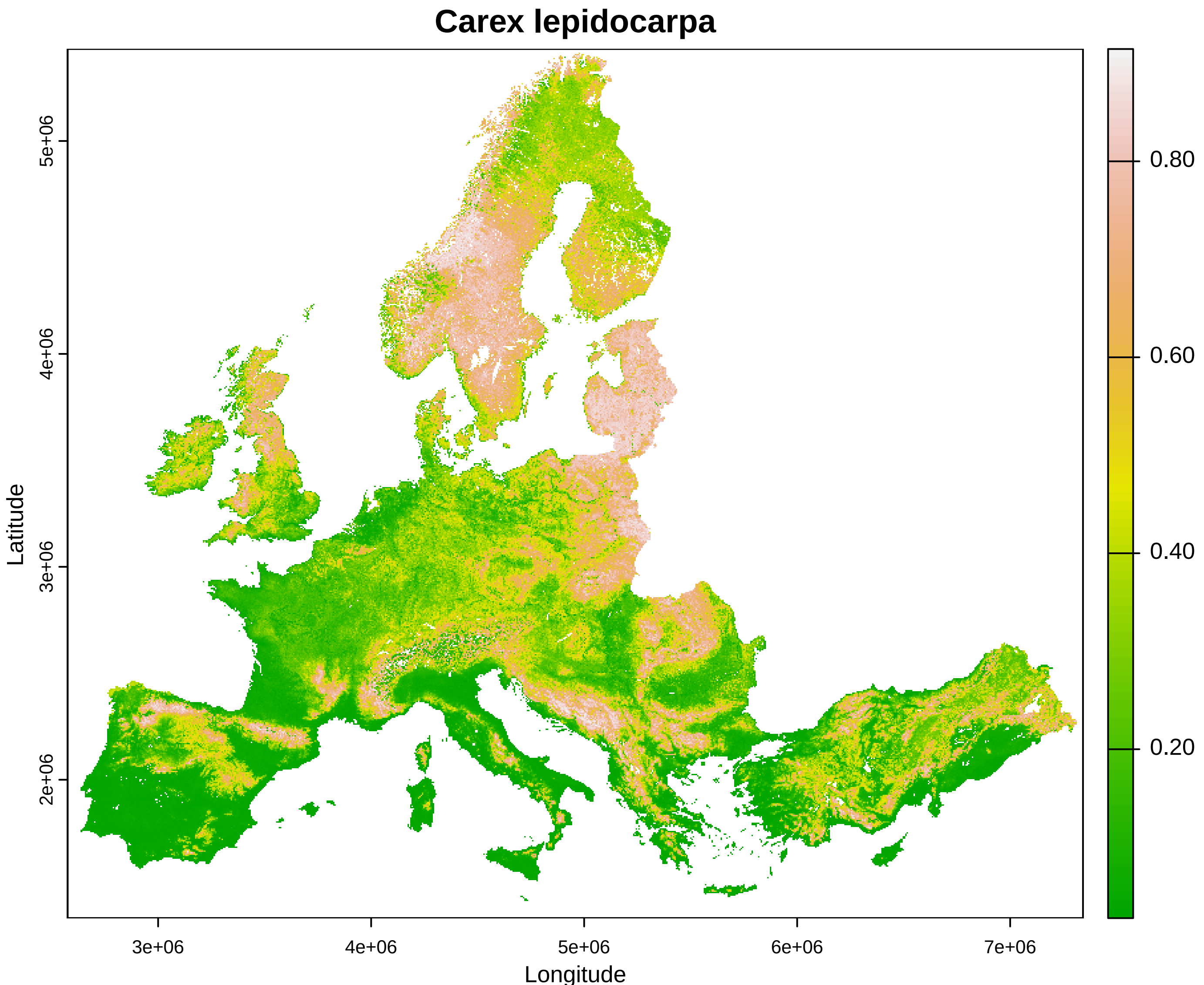
Carex lepidocarpa
Latitude
Longitude
5e+06
4e+06
3e+06
2e+06
3e+06
4e+06
5e+06
6e+06
7e+06
0.80
0.60
0.40
0.20

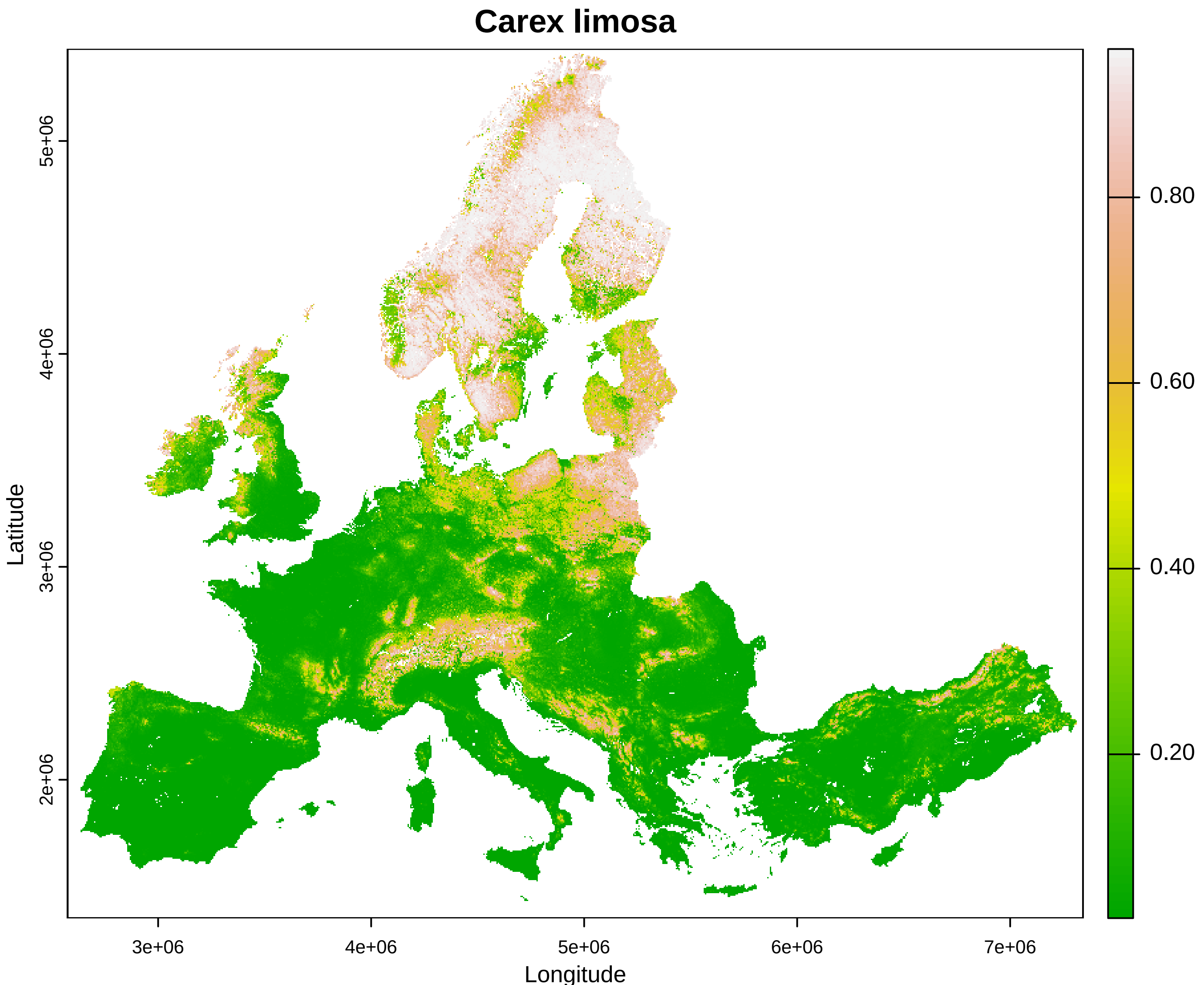
Carex limosa
Latitude
Longitude
5e+06
4e+06
3e+06
2e+06
3e+06
4e+06
5e+06
6e+06
7e+06
0.80
0.60
0.40
0.20

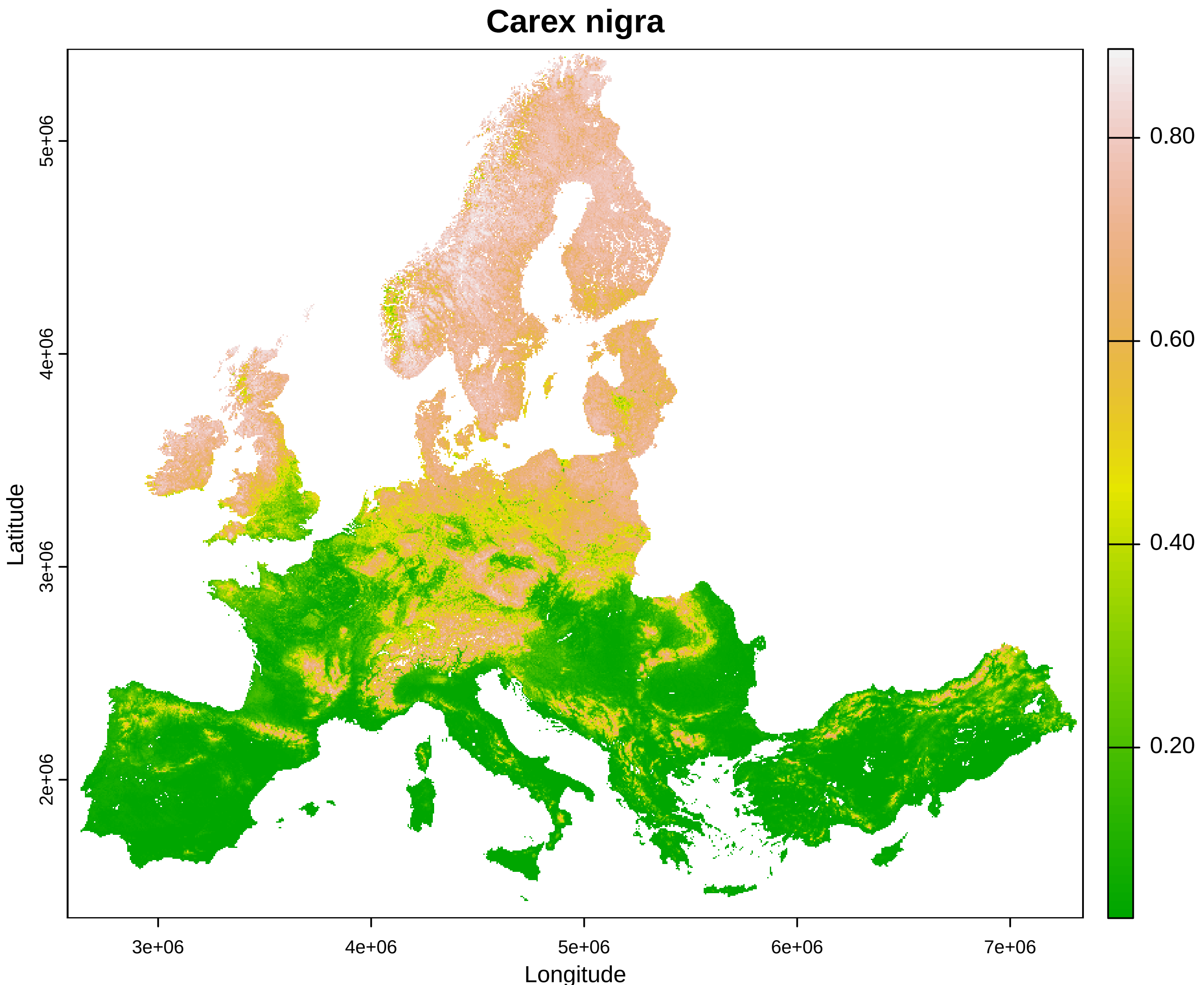

Carex nigra
Latitude
Longitude
5e+06
4e+06
3e+06
2e+06
3e+06
4e+06
5e+06
6e+06
7e+06
0.80
0.60
0.40
0.20

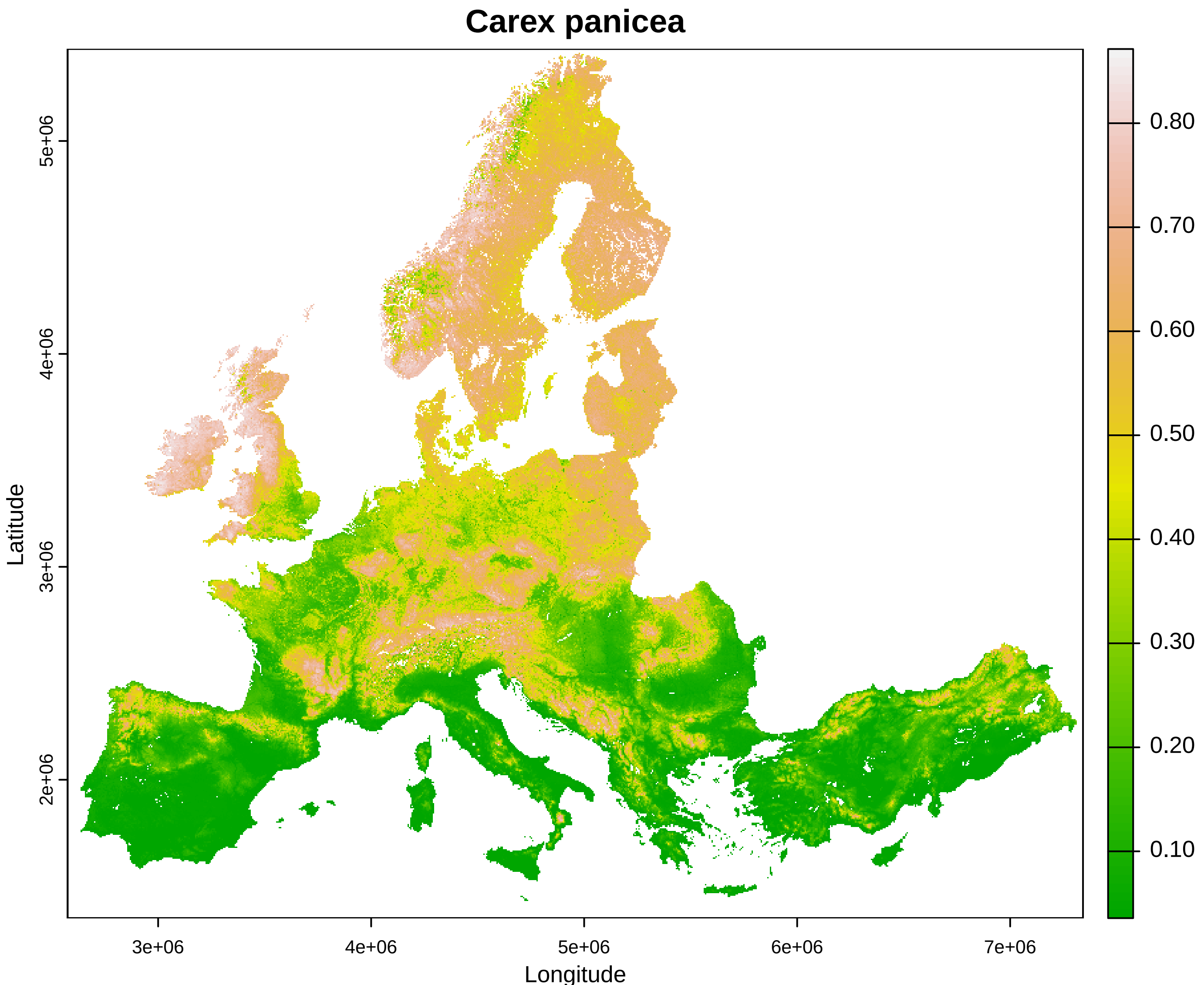
Carex panicea
Latitude
Longitude
5e+06
4e+06
3e+06
2e+06
3e+06
4e+06
5e+06
6e+06
7e+06
0.80
0.70
0.60
0.50
0.40
0.30
0.20
0.10

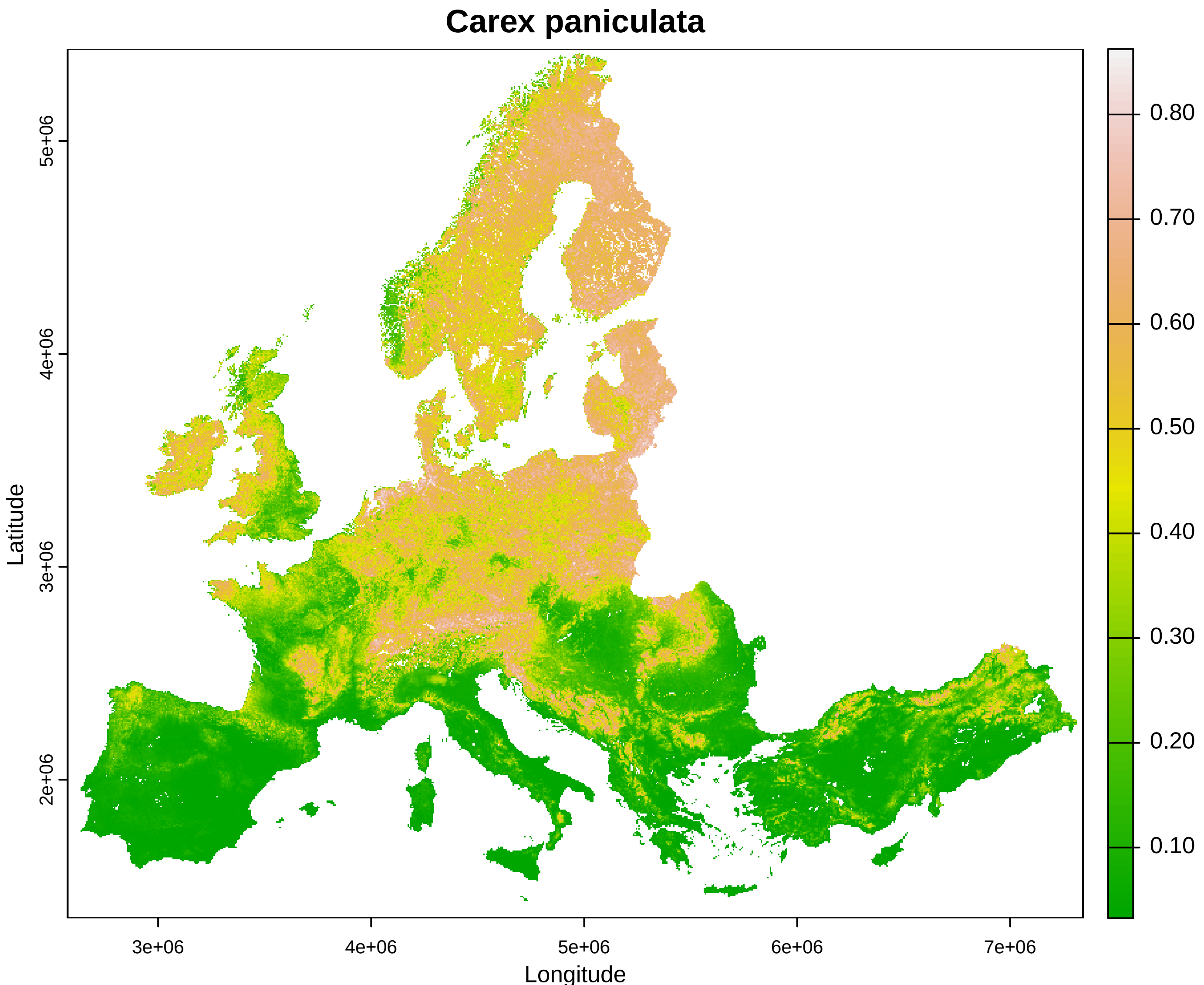
Carex paniculata
Latitude
5e+06
4e+06
3e+06
2e+06
Longitude
3e+06
4e+06
5e+06
6e+06
7e+06
0.80
0.70
0.60
0.50
0.40
0.30
0.20
0.10

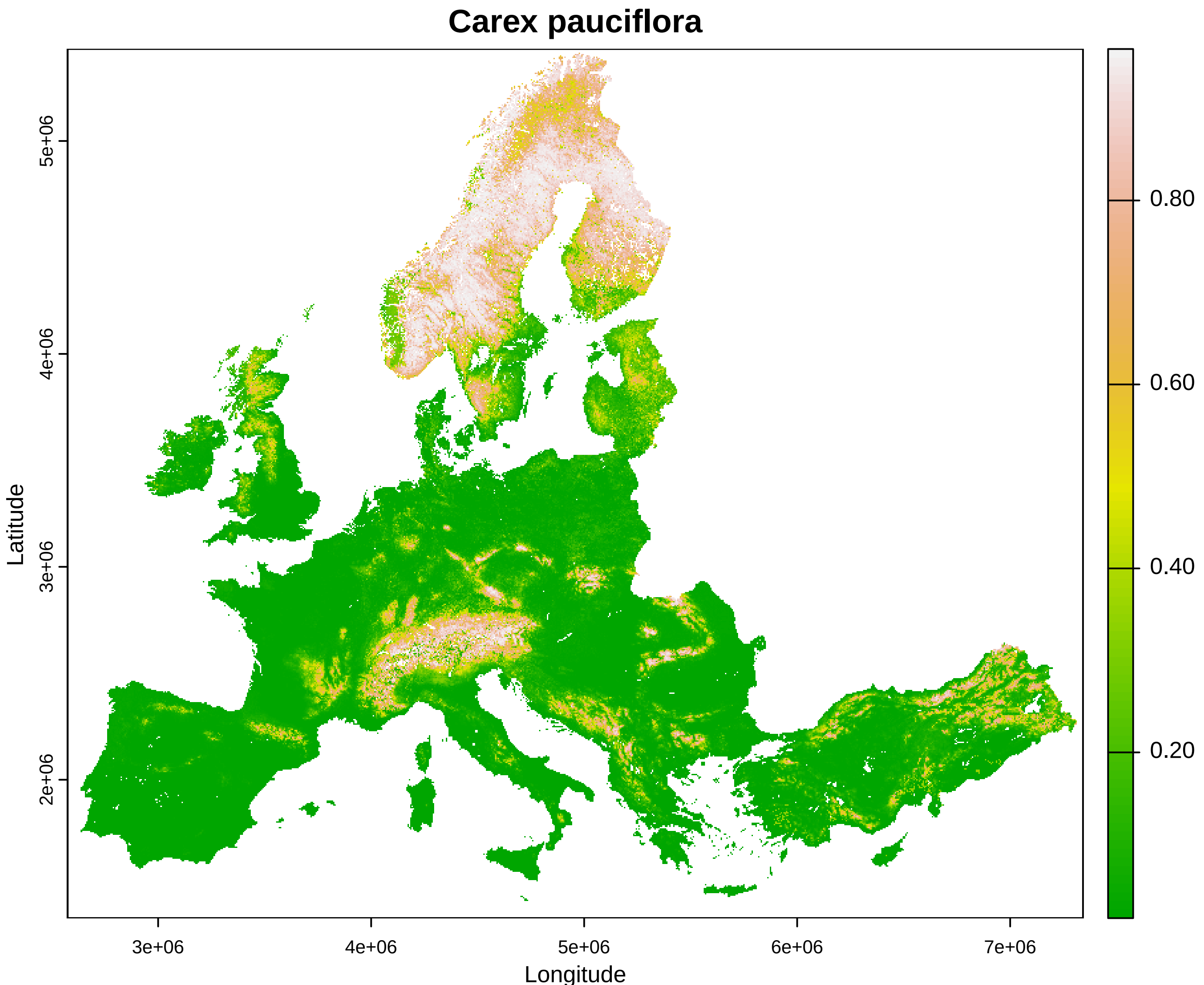

Carex pauciflora
Latitude
Longitude
5e+06
4e+06
3e+06
2e+06
3e+06
4e+06
5e+06
6e+06
7e+06
0.80
0.60
0.40
0.20

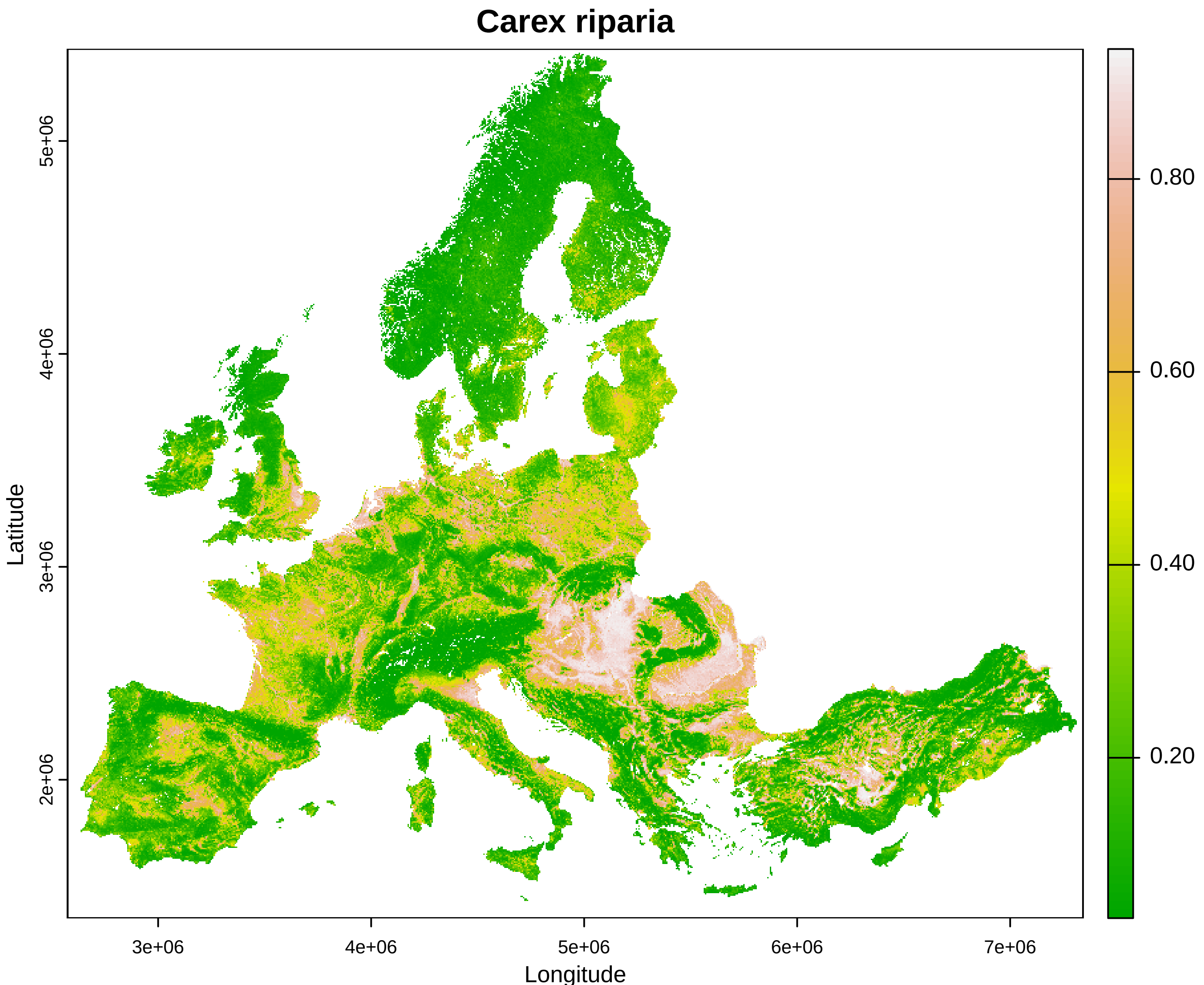
Carex riparia
Latitude
Longitude
5e+06
4e+06
3e+06
2e+06
3e+06
4e+06
5e+06
6e+06
7e+06
0.80
0.60
0.40
0.20

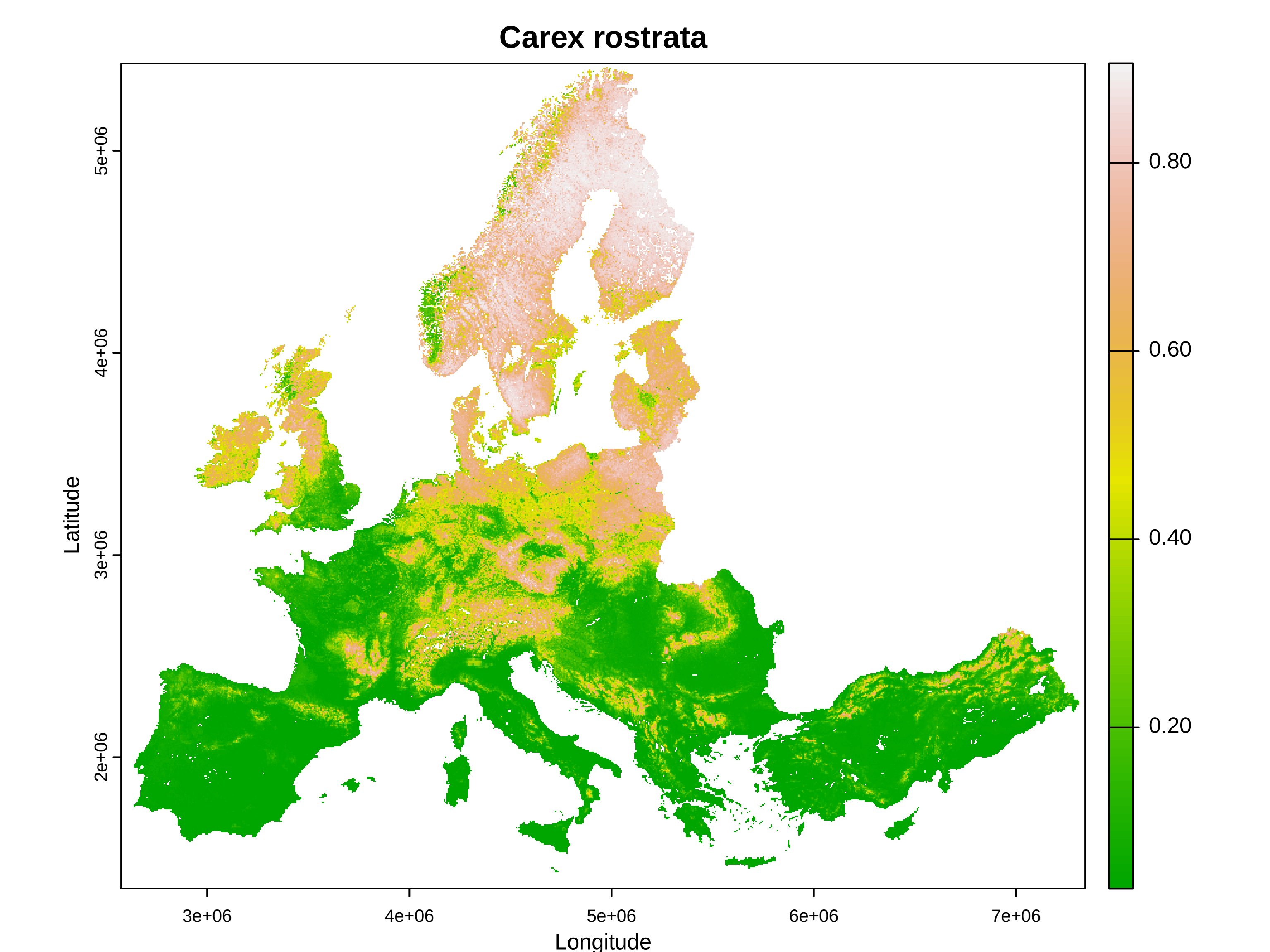

Carex rostrata
Latitude
5e+06
4e+06
3e+06
2e+06
3e+06
4e+06
5e+06
6e+06
7e+06
Longitude
0.80
0.60
0.40
0.20

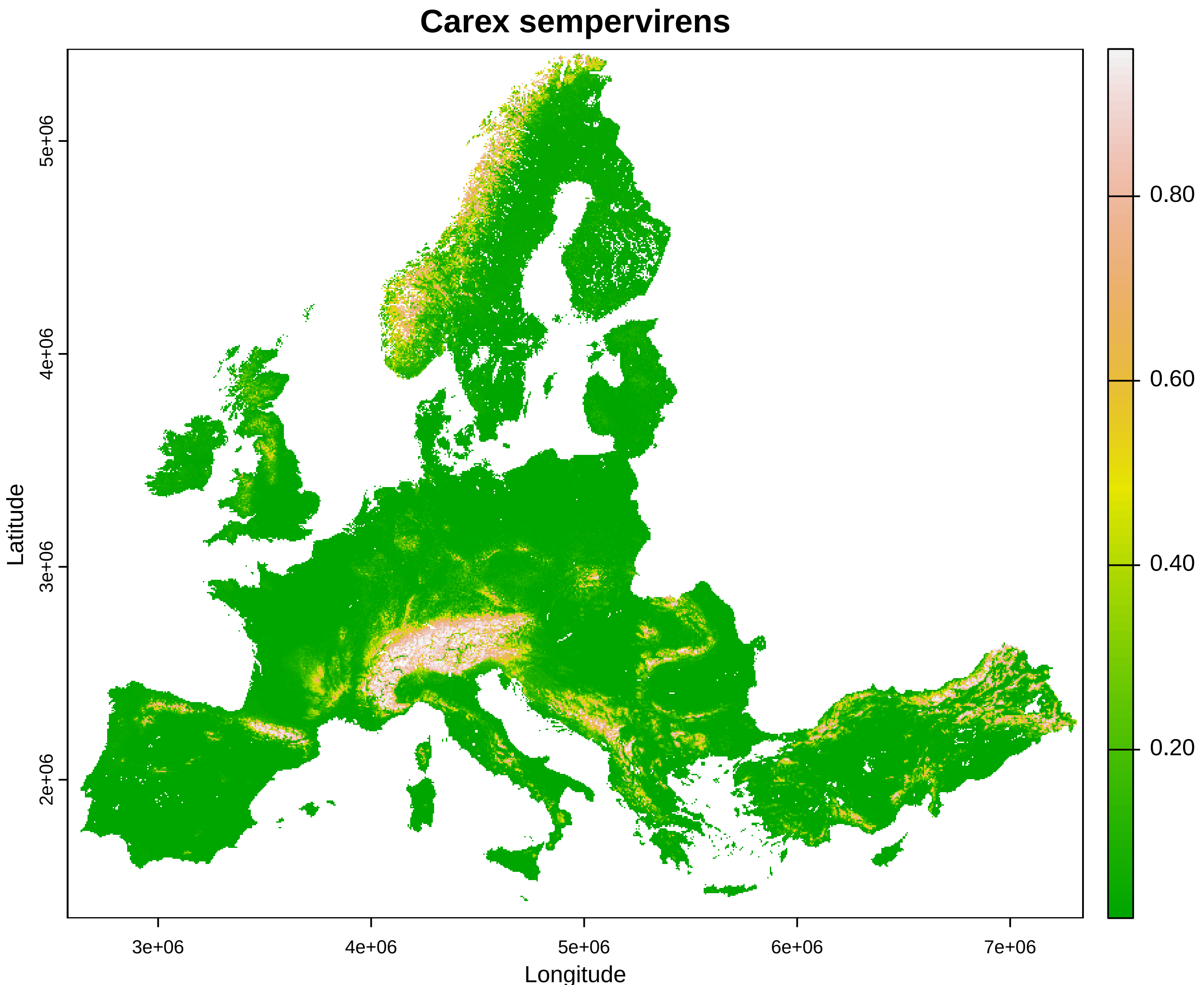
Carex sempervirens
Latitude
Longitude
5e+06
4e+06
3e+06
2e+06
3e+06
4e+06
5e+06
6e+06
7e+06
0.80
0.60
0.40
0.20

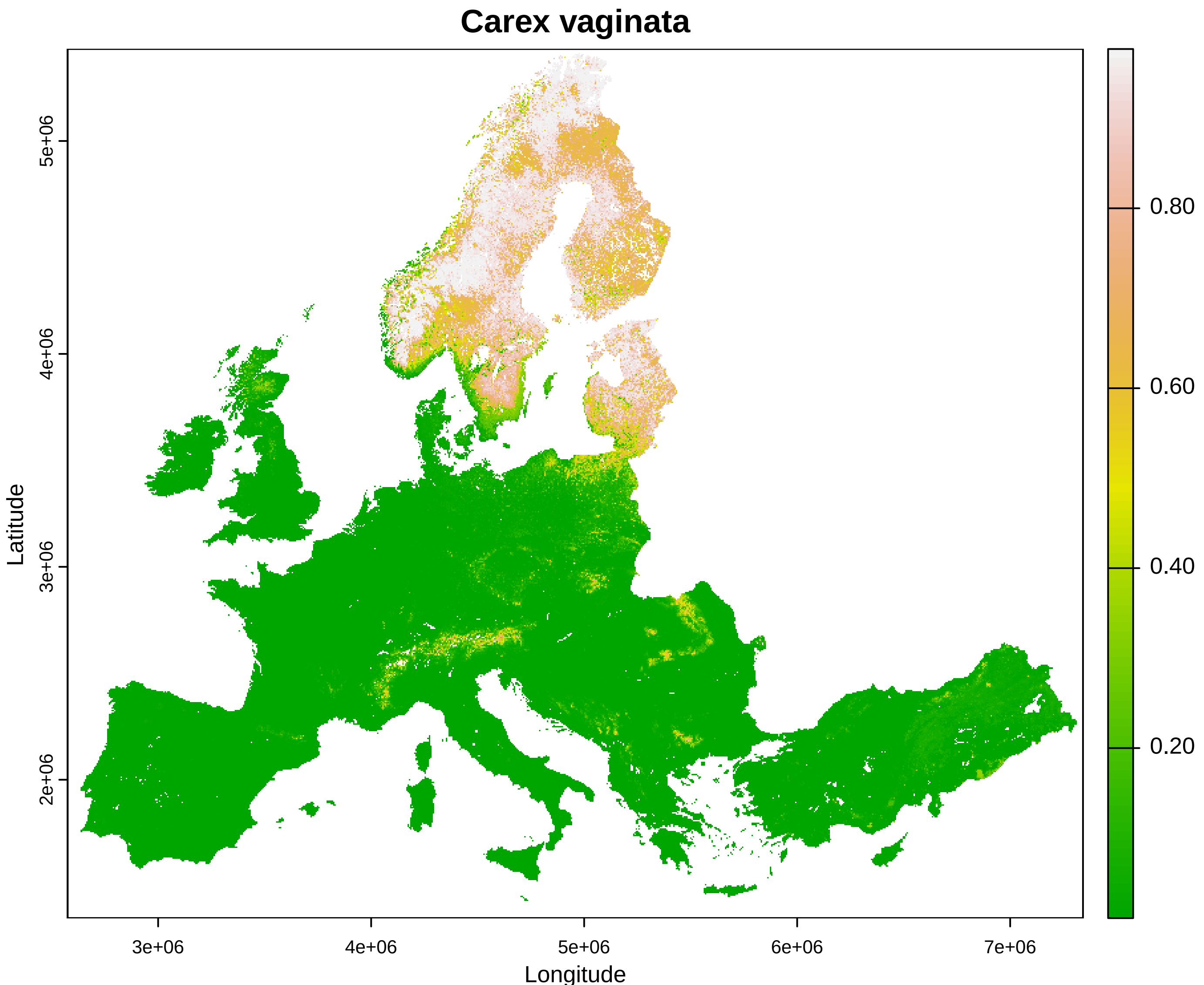
Carex vaginata
Latitude
5e+06
4e+06
3e+06
2e+06
Longitude
3e+06
4e+06
5e+06
6e+06
7e+06
0.80
0.60
0.40
0.20

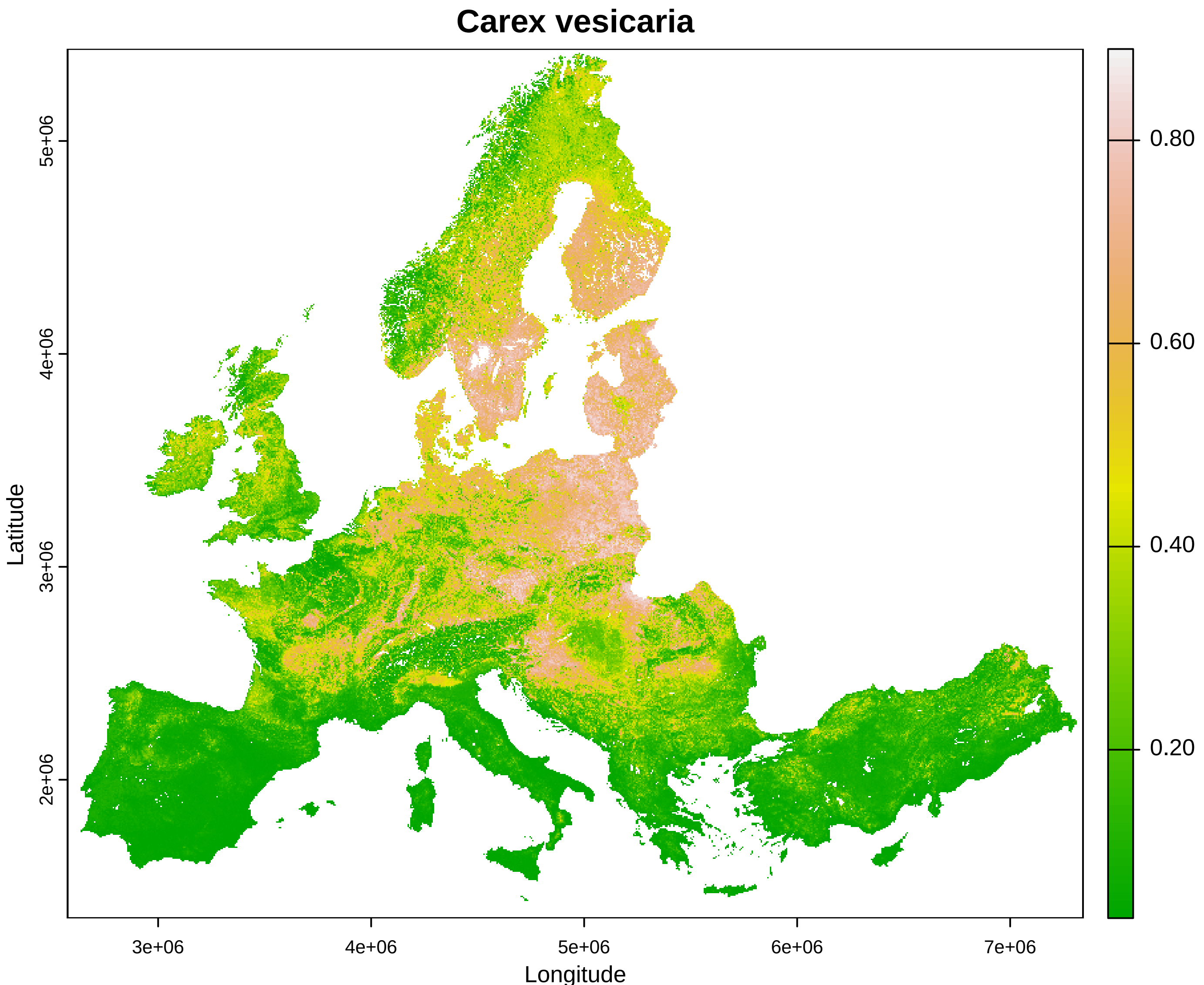
Carex vesicaria
Latitude
Longitude
5e+06
4e+06
3e+06
2e+06
3e+06
4e+06
5e+06
6e+06
7e+06
0.80
0.60
0.40
0.20

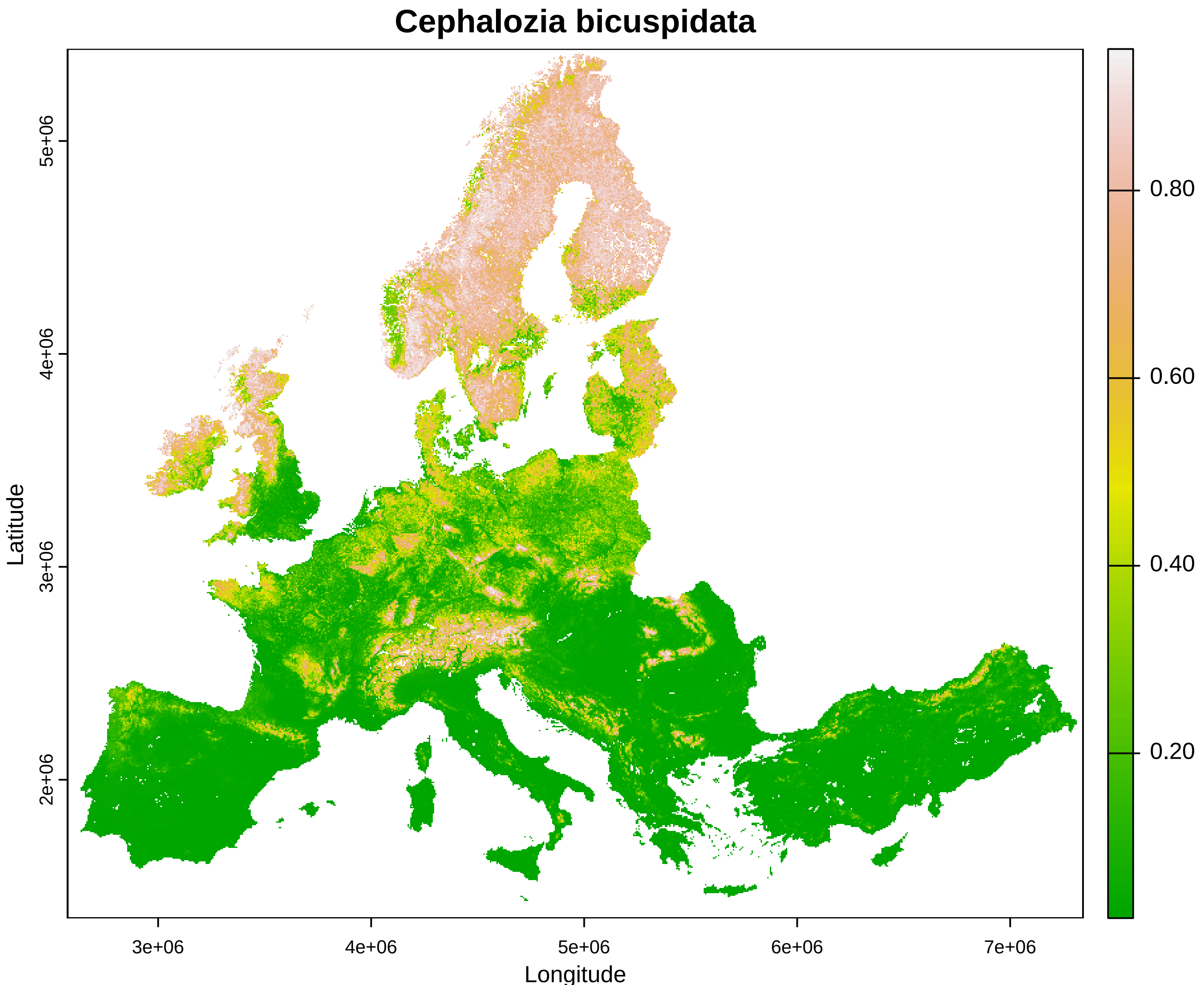

Cephalozia bicuspidata
Latitude
Longitude
5e+06
4e+06
3e+06
2e+06
3e+06
4e+06
5e+06
6e+06
7e+06
0.80
0.60
0.40
0.20

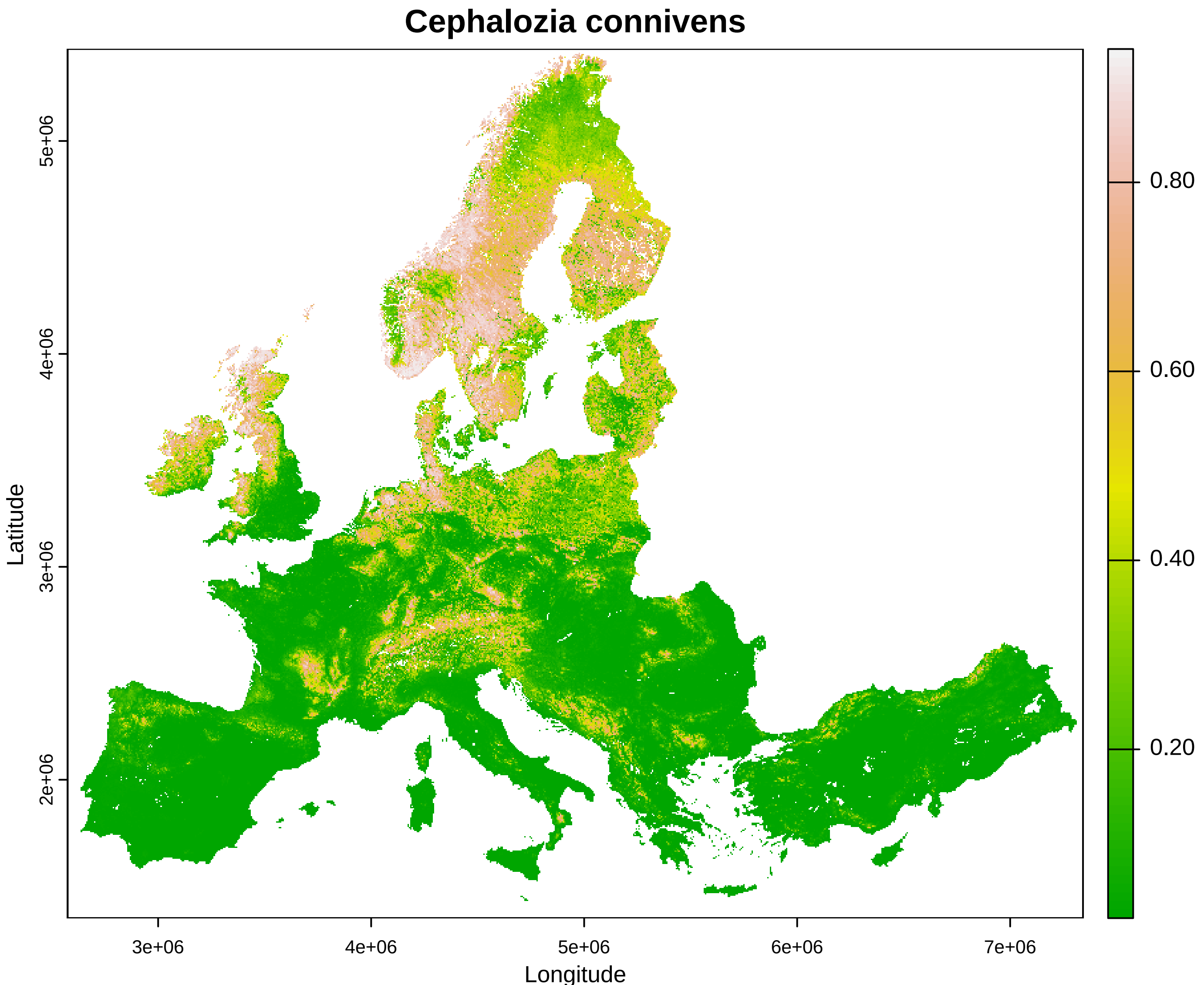
Cephalozia connivens
Latitude
Longitude
5e+06
4e+06
3e+06
2e+06
3e+06
4e+06
5e+06
6e+06
7e+06
0.80
0.60
0.40
0.20

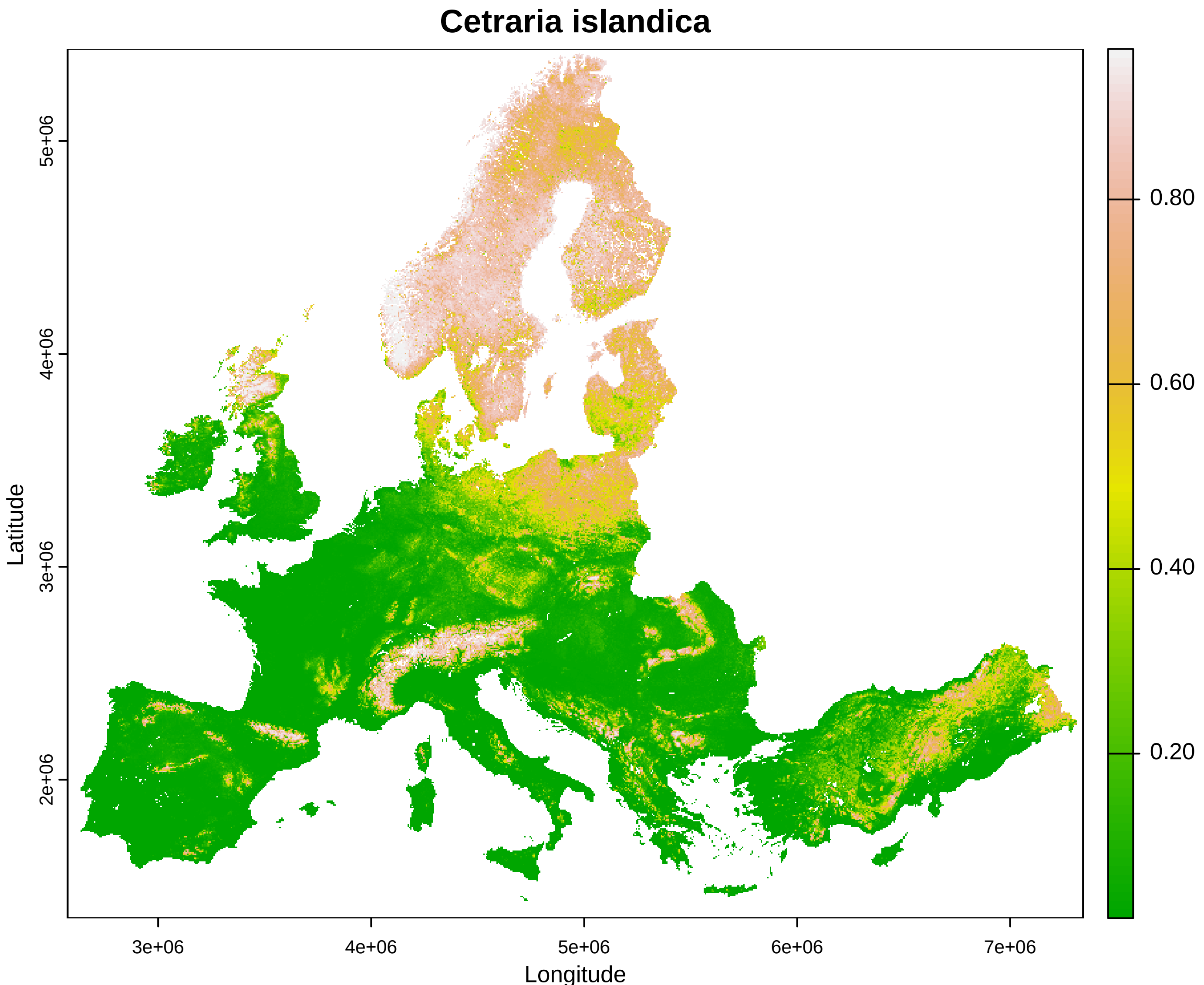

Cetraria islandica
Latitude
5e+06
4e+06
3e+06
2e+06
Longitude
3e+06
4e+06
5e+06
6e+06
7e+06
0.80
0.60
0.40
0.20

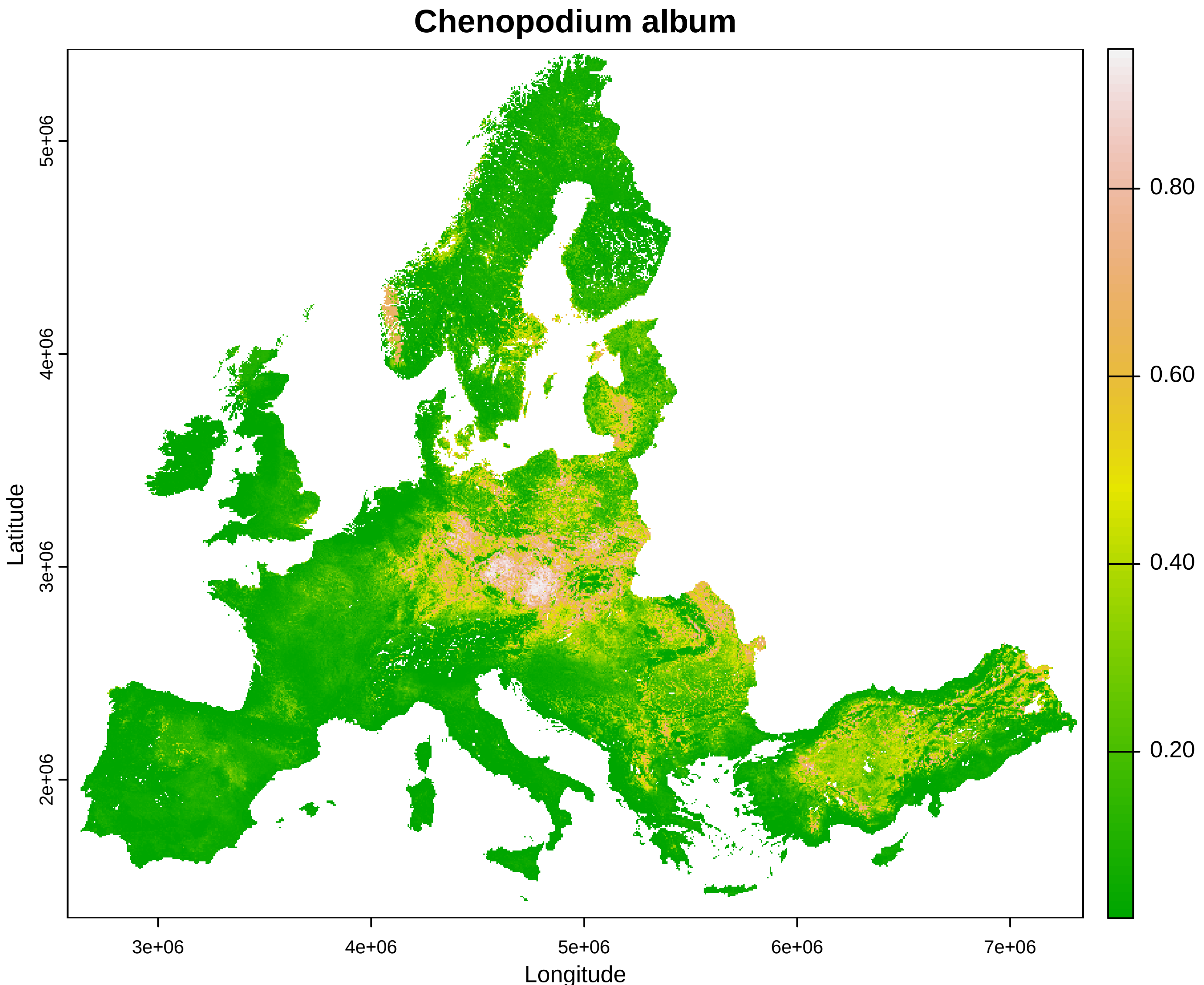

Chenopodium album
Latitude
Longitude
5e+06
4e+06
3e+06
2e+06
3e+06
4e+06
5e+06
6e+06
7e+06
0.80
0.60
0.40
0.20

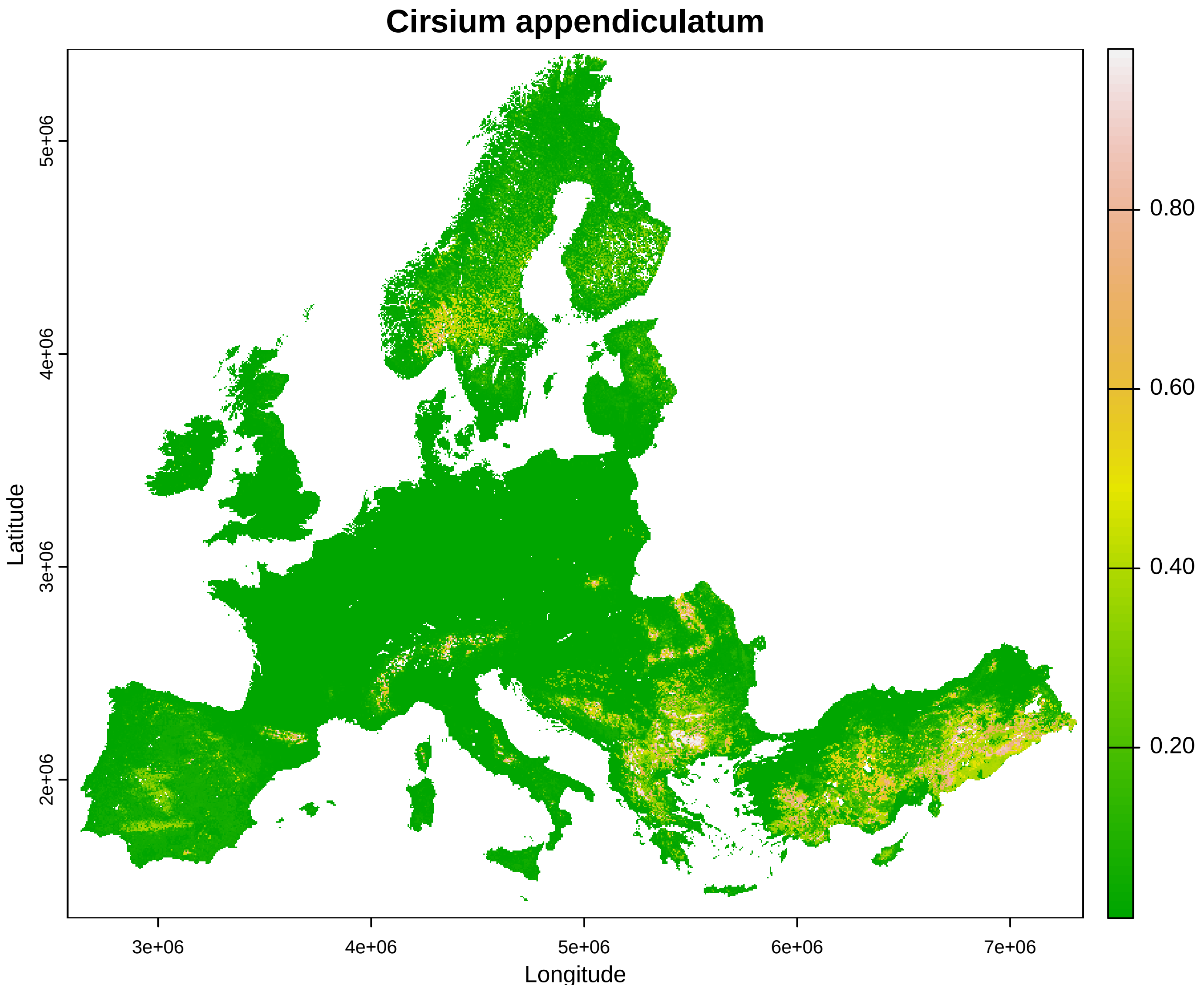
Cirsium appendiculatum
Latitude
Longitude
5e+06
4e+06
3e+06
2e+06
3e+06
4e+06
5e+06
6e+06
7e+06
0.80
0.60
0.40
0.20

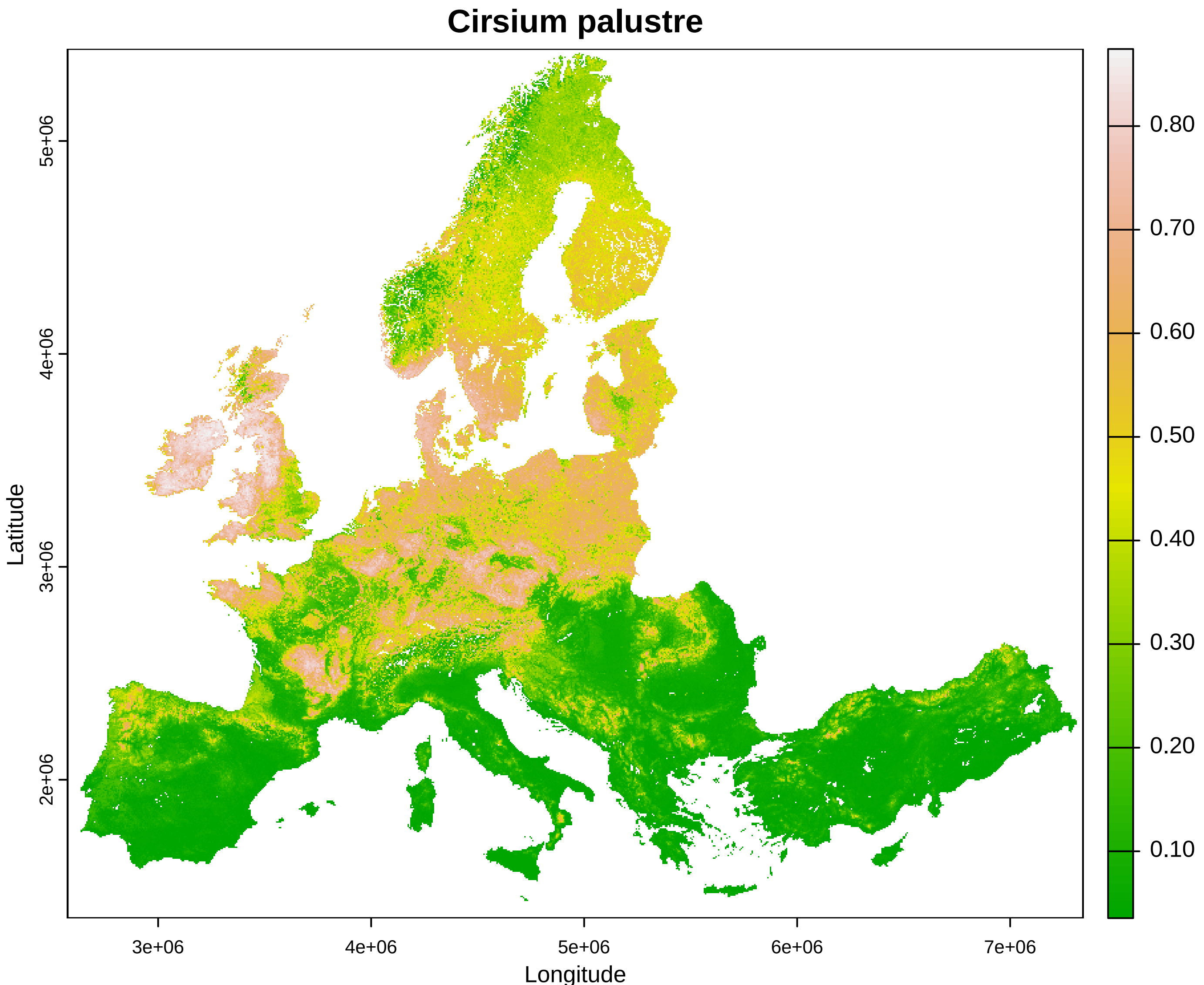

Cirsium palustre
Latitude
Longitude
5e+06
4e+06
3e+06
2e+06
3e+06
4e+06
5e+06
6e+06
7e+06
0.80
0.70
0.60
0.50
0.40
0.30
0.20
0.10

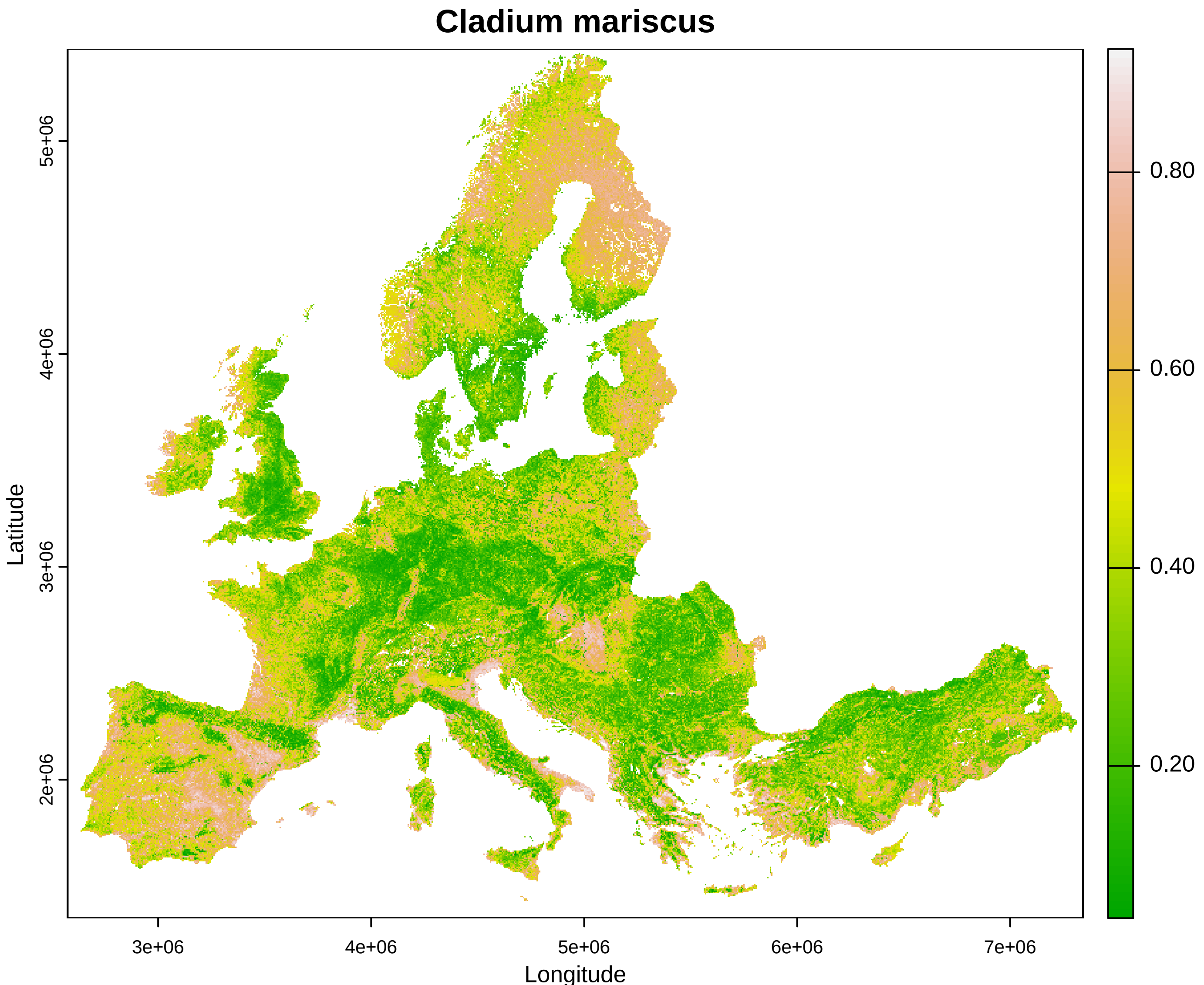
Cladium mariscus
Latitude
5e+06
4e+06
3e+06
2e+06
3e+06
4e+06
5e+06
6e+06
7e+06
Longitude
0.80
0.60
0.40
0.20

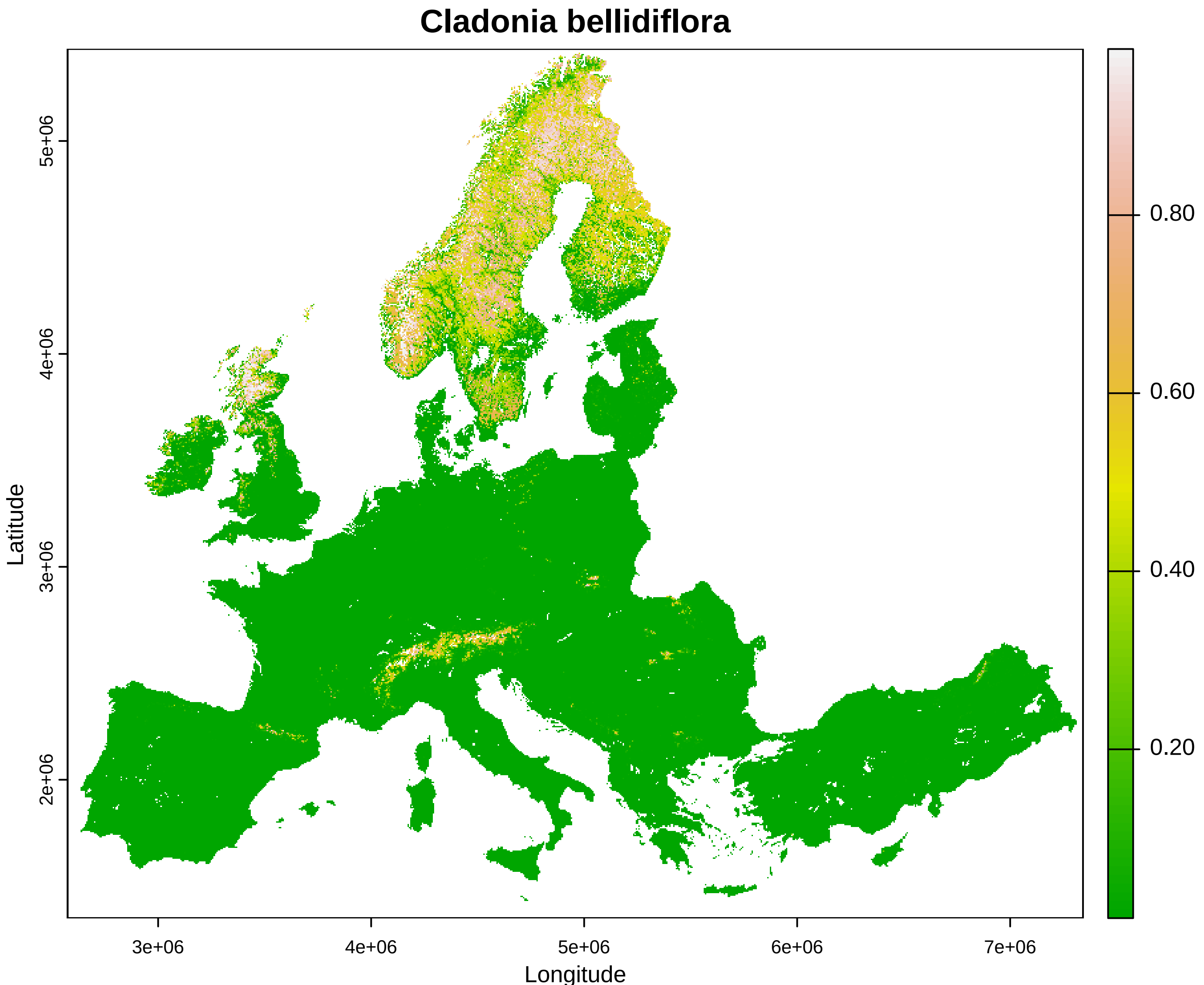

Cladonia bellidiflora
Latitude
Longitude
5e+06
4e+06
3e+06
2e+06
3e+06
4e+06
5e+06
6e+06
7e+06
0.80
0.60
0.40
0.20

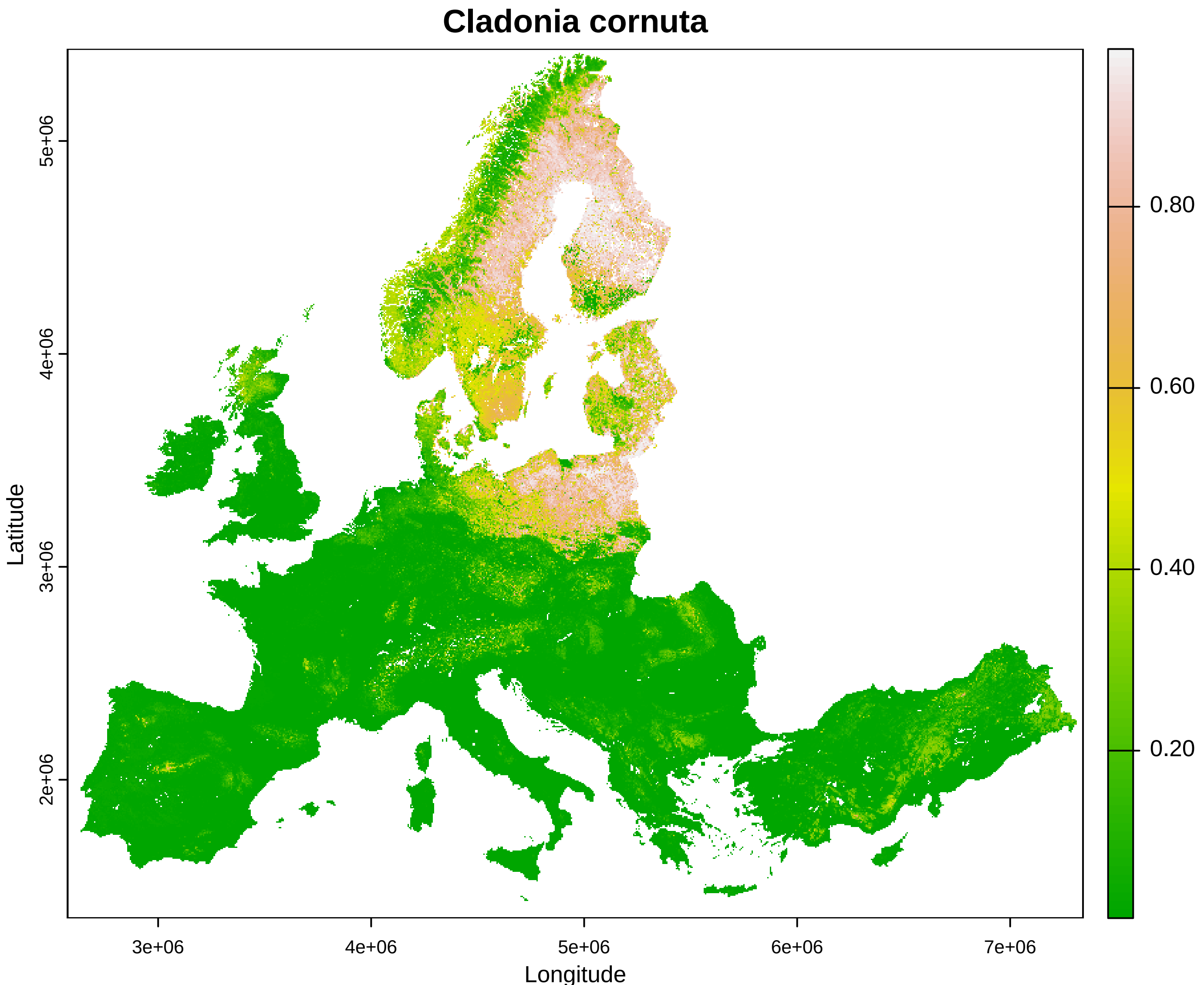
Cladonia cornuta
Latitude
5e+06
4e+06
3e+06
2e+06
3e+06
4e+06
5e+06
6e+06
7e+06
Longitude
0.80
0.60
0.40
0.20

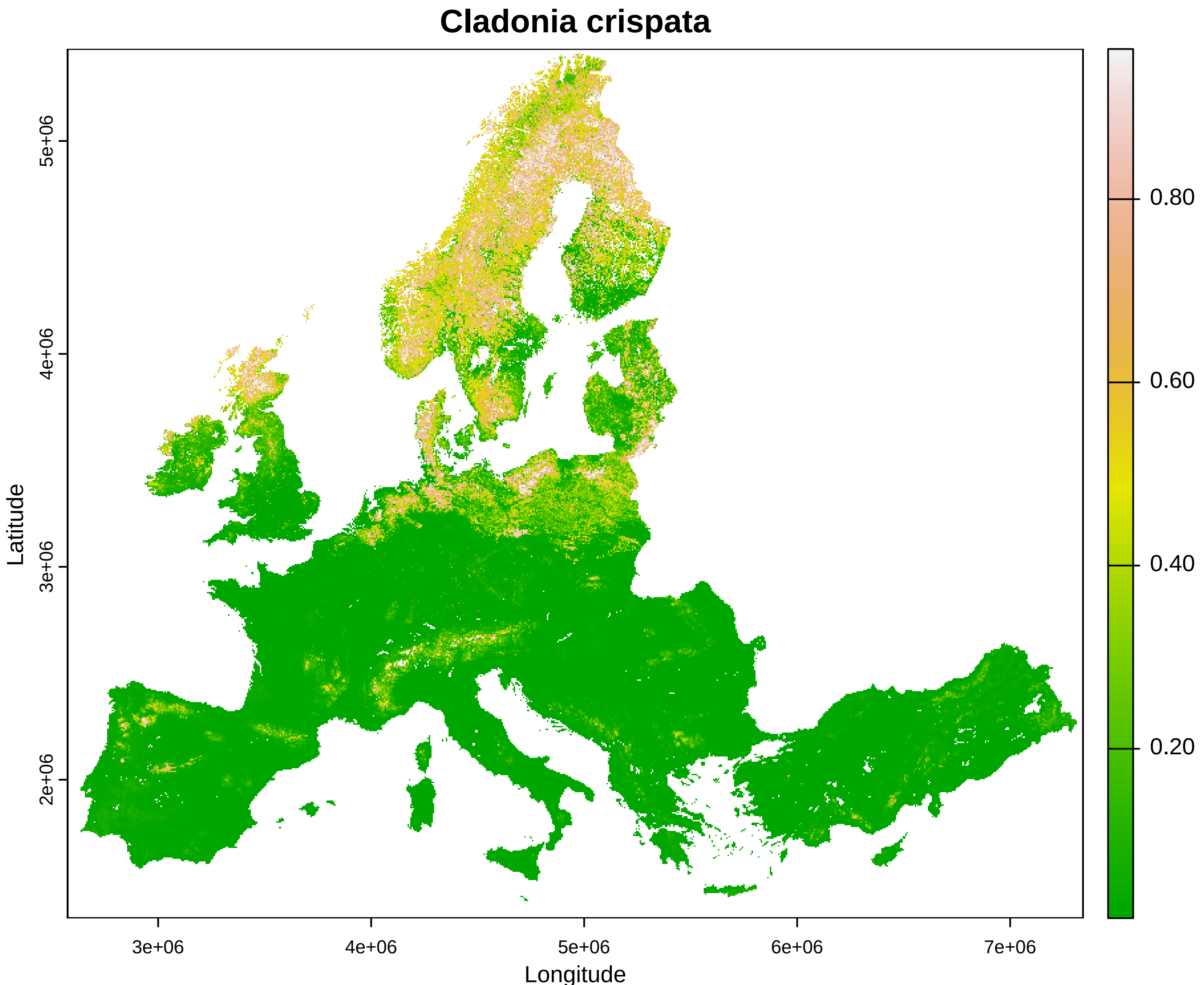

Cladonia crispata
Latitude
Longitude
5e+06
4e+06
3e+06
2e+06
3e+06
4e+06
5e+06
6e+06
7e+06
0.80
0.60
0.40
0.20

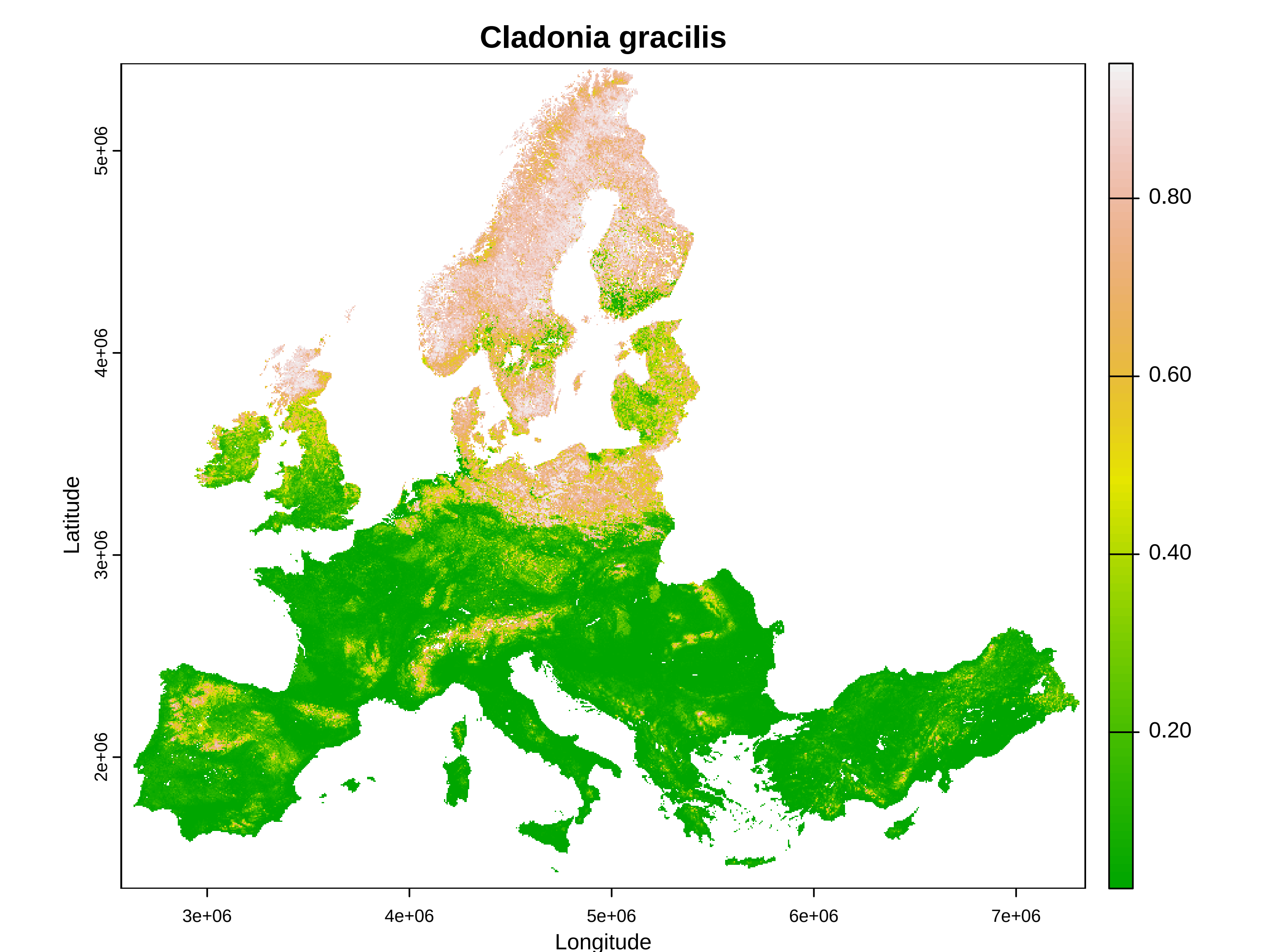

Cladonia gracilis
Latitude
Longitude
5e+06
4e+06
3e+06
2e+06
3e+06
4e+06
5e+06
6e+06
7e+06
0.80
0.60
0.40
0.20

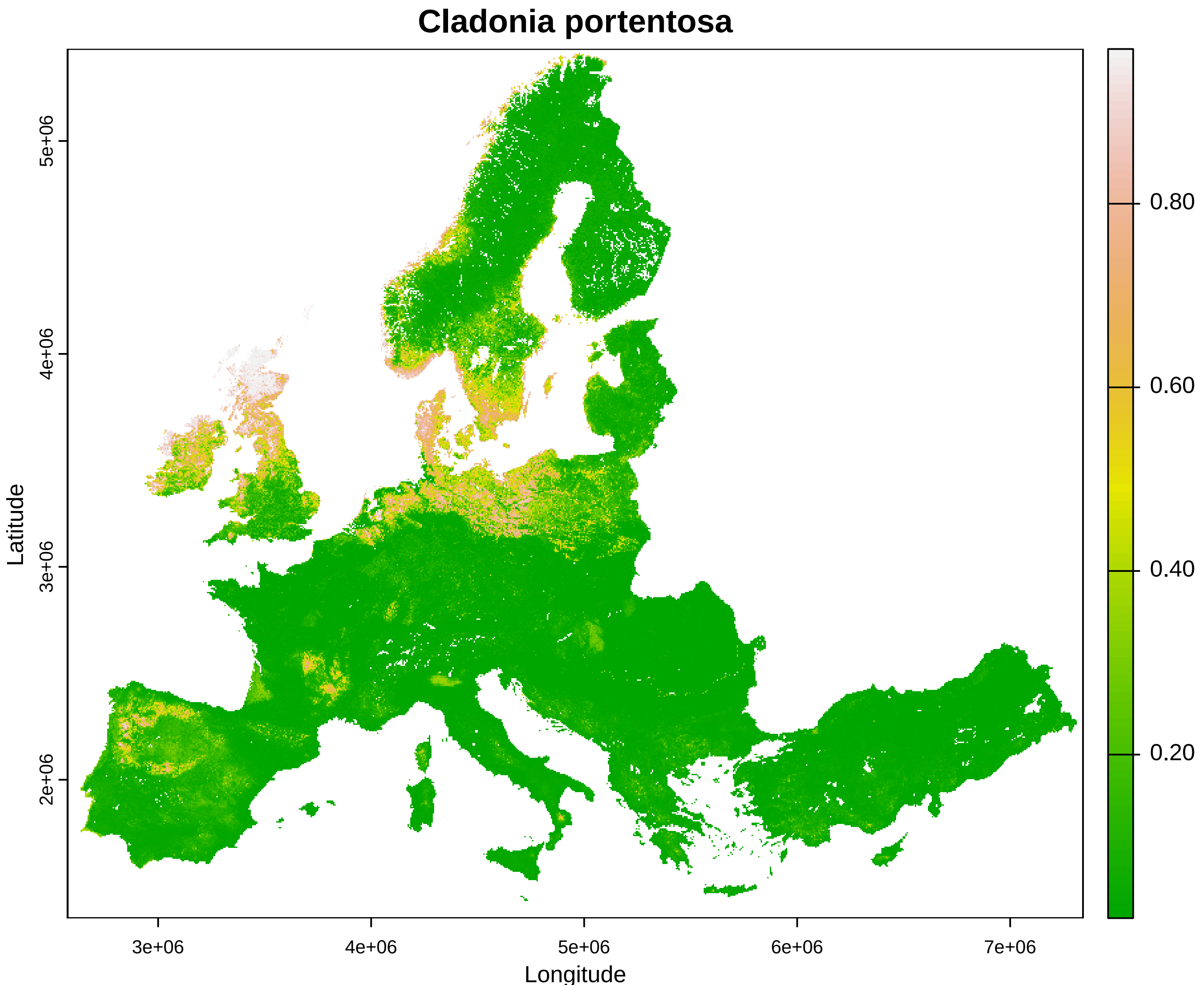
Cladonia portentosa
Latitude
Longitude
5e+06
4e+06
3e+06
2e+06
3e+06
4e+06
5e+06
6e+06
7e+06
0.80
0.60
0.40
0.20

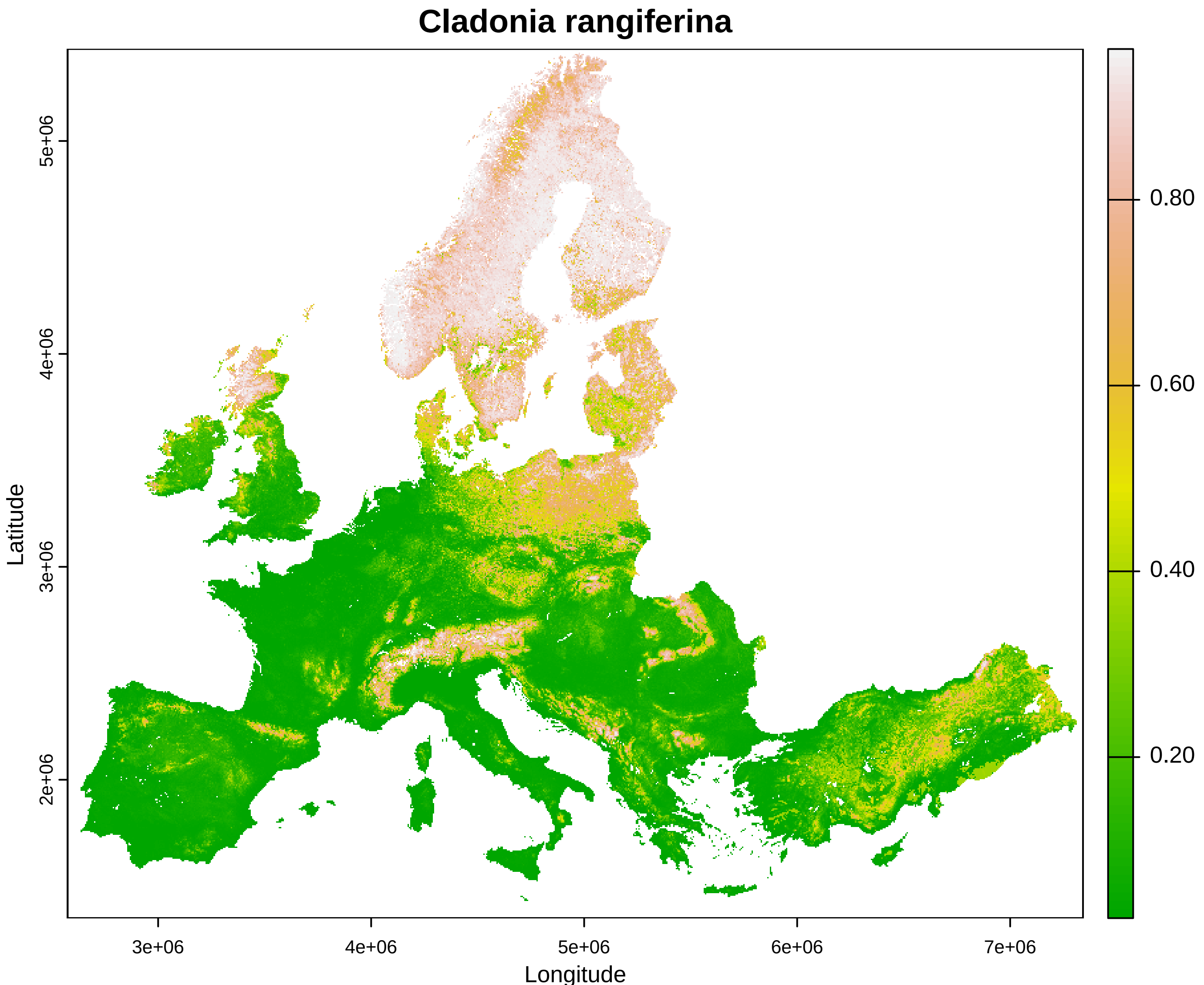
Cladonia rangiferina
Latitude
5e+06
4e+06
3e+06
2e+06
Longitude
3e+06
4e+06
5e+06
6e+06
7e+06
0.80
0.60
0.40
0.20

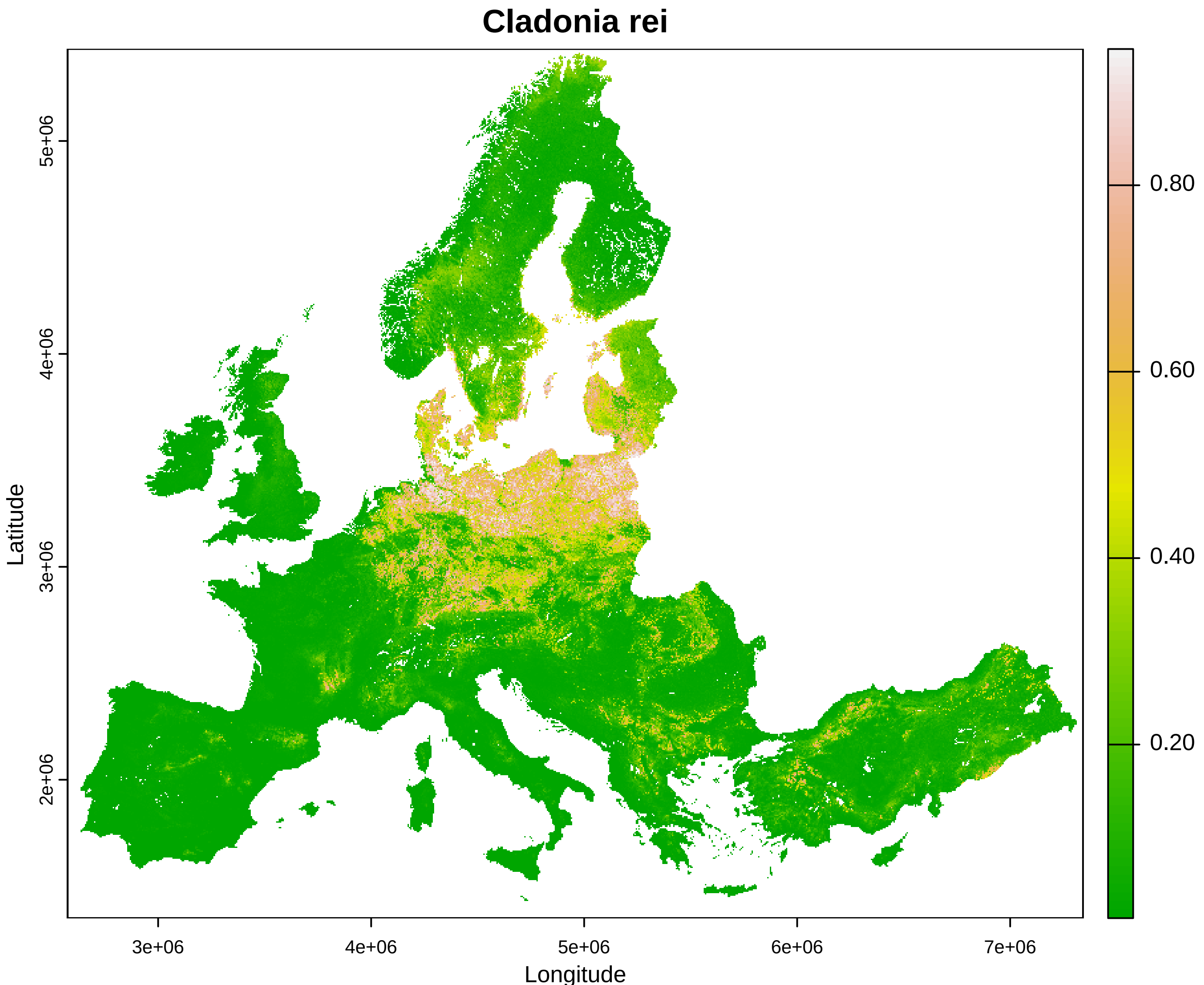
Cladonia rei
Latitude
Longitude
5e+06
4e+06
3e+06
2e+06
3e+06
4e+06
5e+06
6e+06
7e+06
0.80
0.60
0.40
0.20

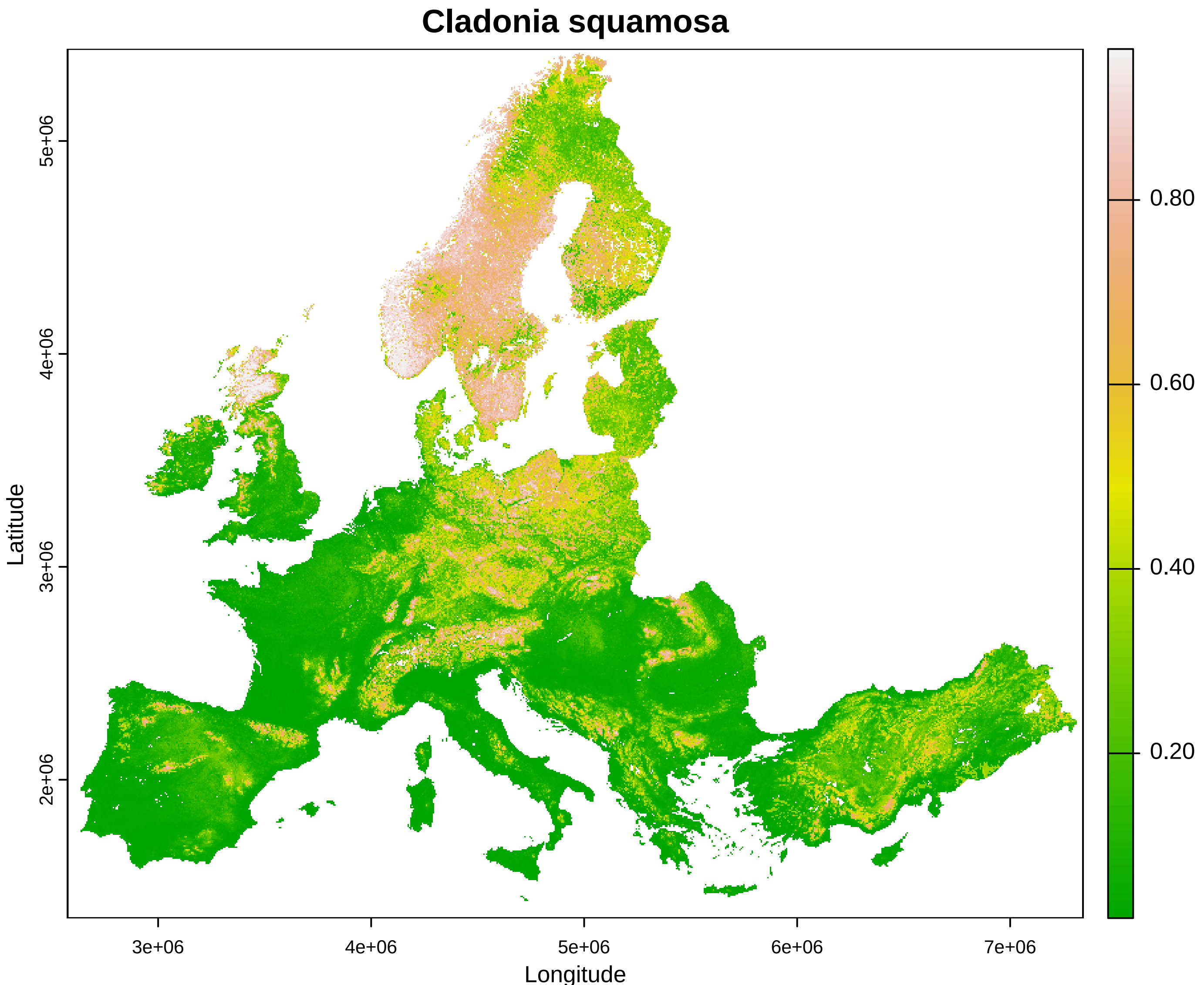
Cladonia squamosa
Latitude
Longitude
5e+06
4e+06
3e+06
2e+06
3e+06
4e+06
5e+06
6e+06
7e+06
0.80
0.60
0.40
0.20

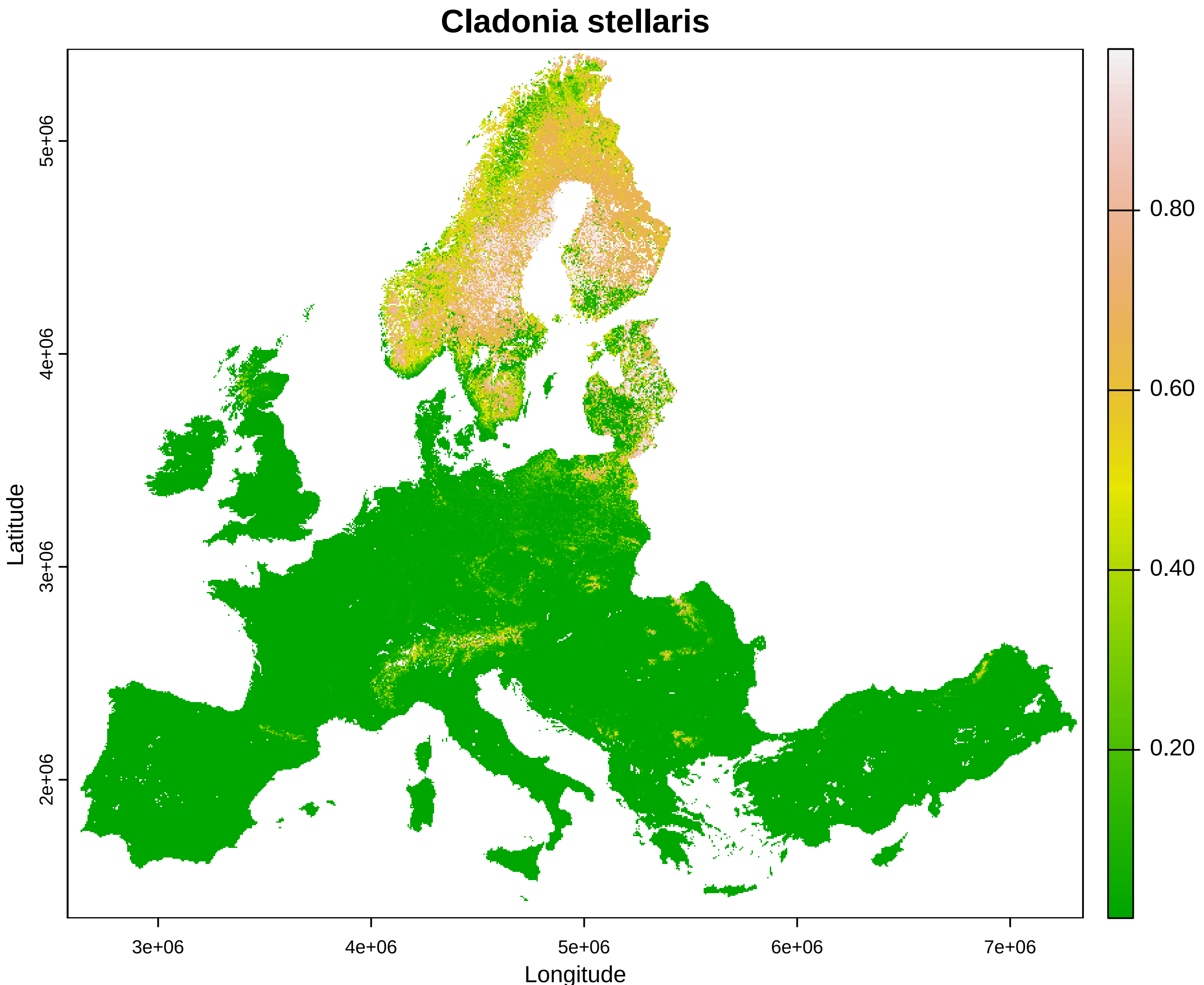
Cladonia stellaris
Latitude
Longitude
5e+06
4e+06
3e+06
2e+06
3e+06
4e+06
5e+06
6e+06
7e+06
0.80
0.60
0.40
0.20

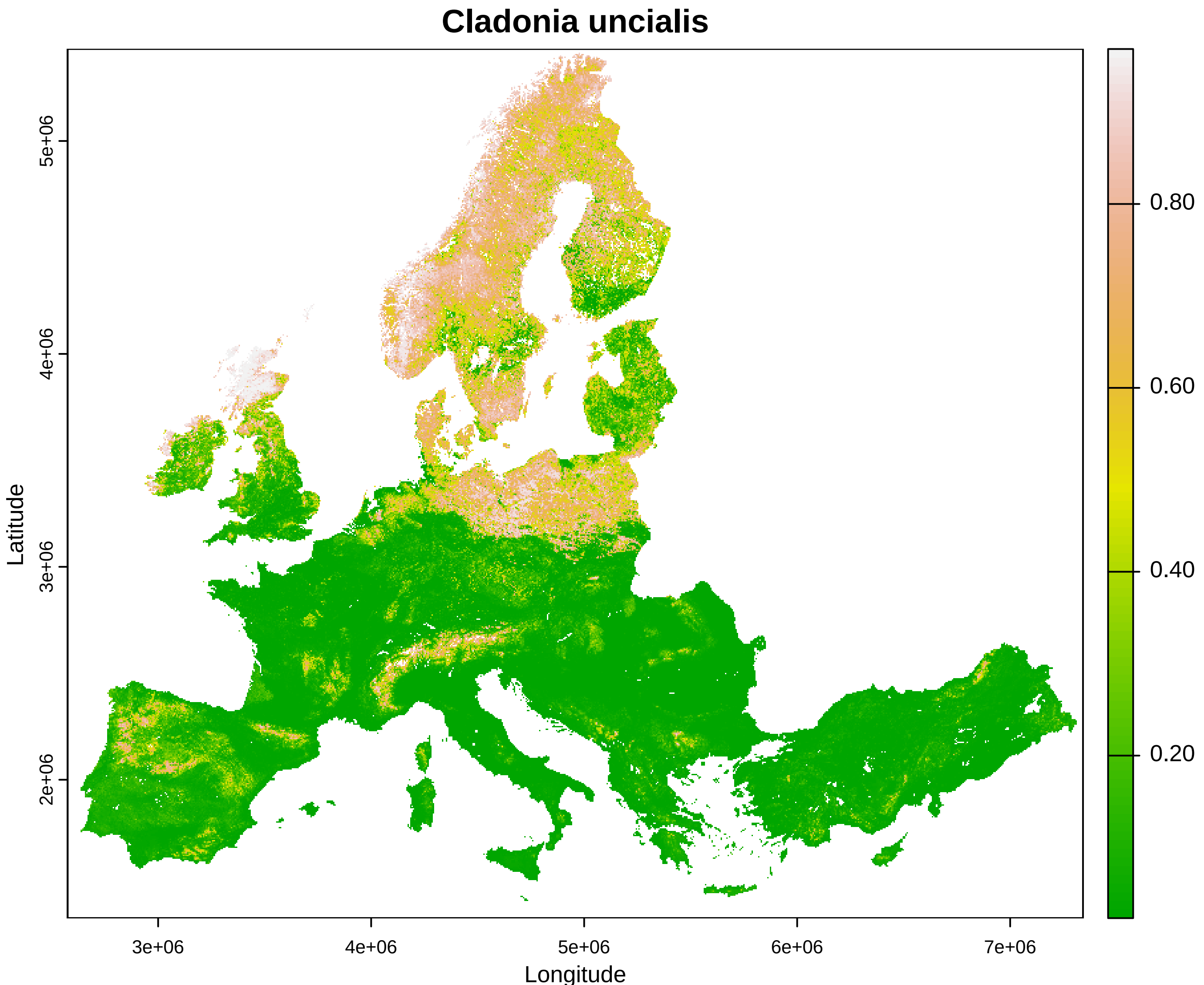

Cladonia uncialis
Latitude
5e+06
4e+06
3e+06
2e+06
3e+06
4e+06
5e+06
6e+06
7e+06
Longitude
0.80
0.60
0.40
0.20

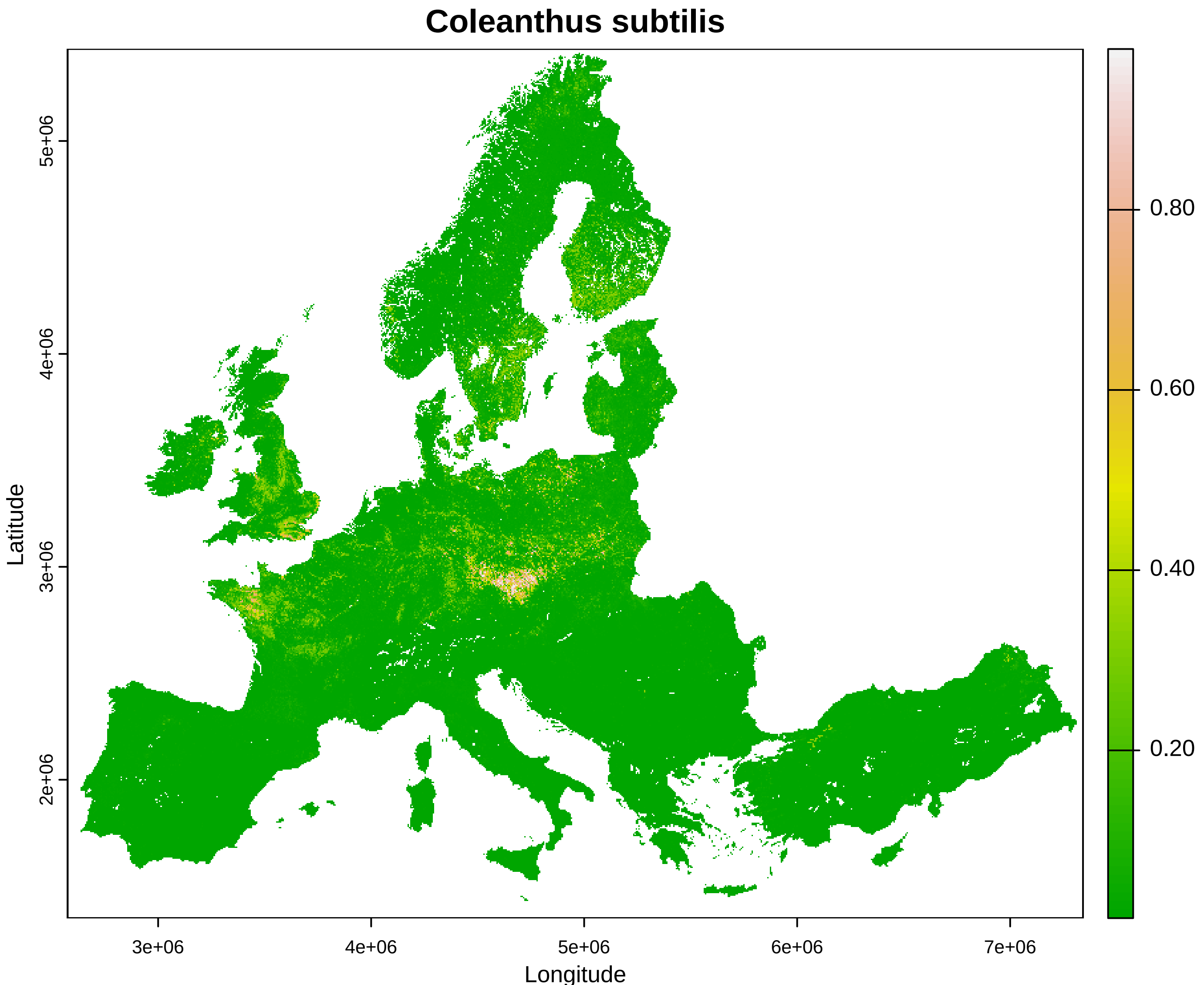
Coleanthus subtilis
Latitude
Longitude
5e+06
4e+06
3e+06
2e+06
3e+06
4e+06
5e+06
6e+06
7e+06
0.80
0.60
0.40
0.20

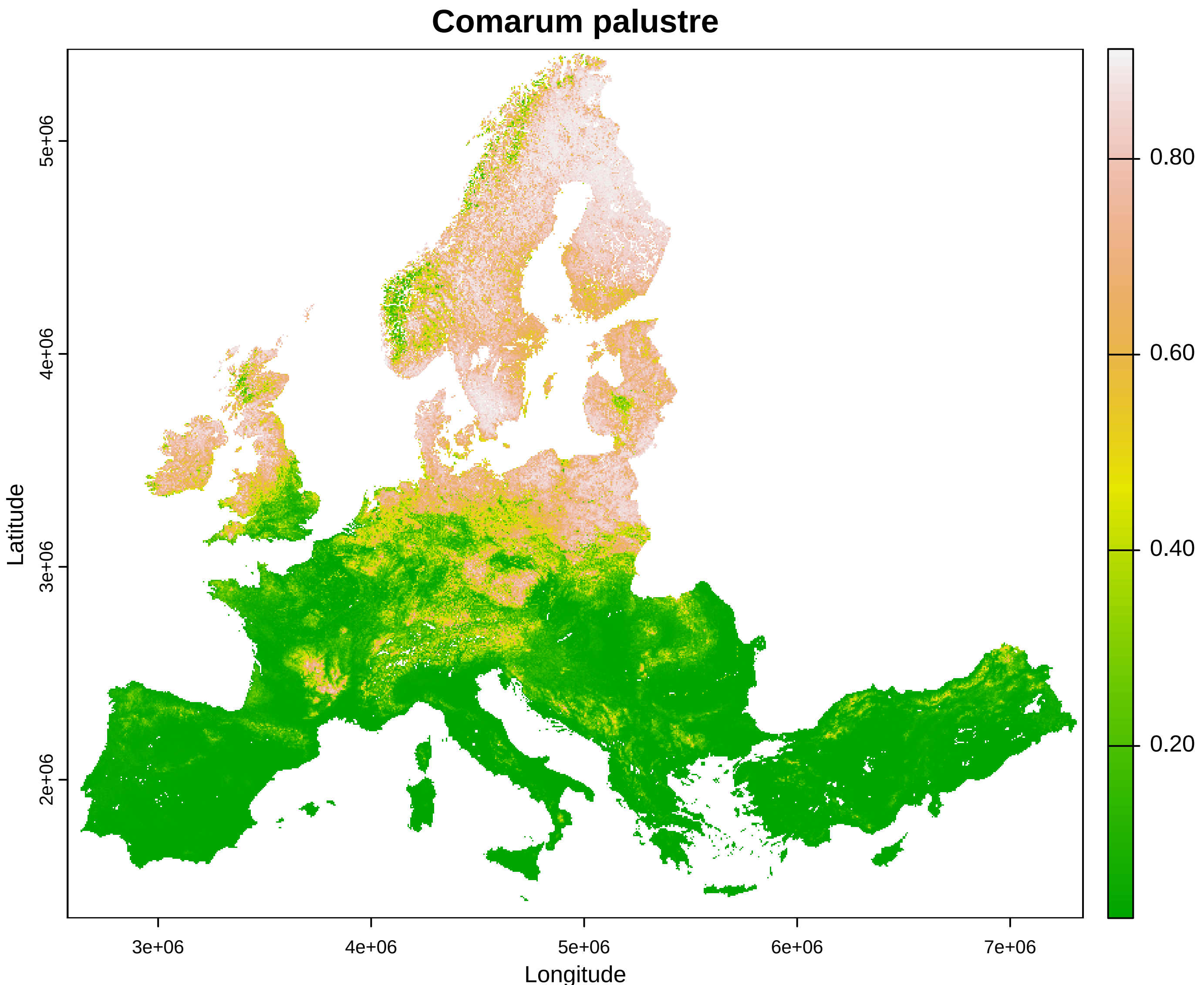
Comarum palustre
Latitude
5e+06
4e+06
3e+06
2e+06
3e+06
4e+06
5e+06
6e+06
7e+06
Longitude
0.80
0.60
0.40
0.20

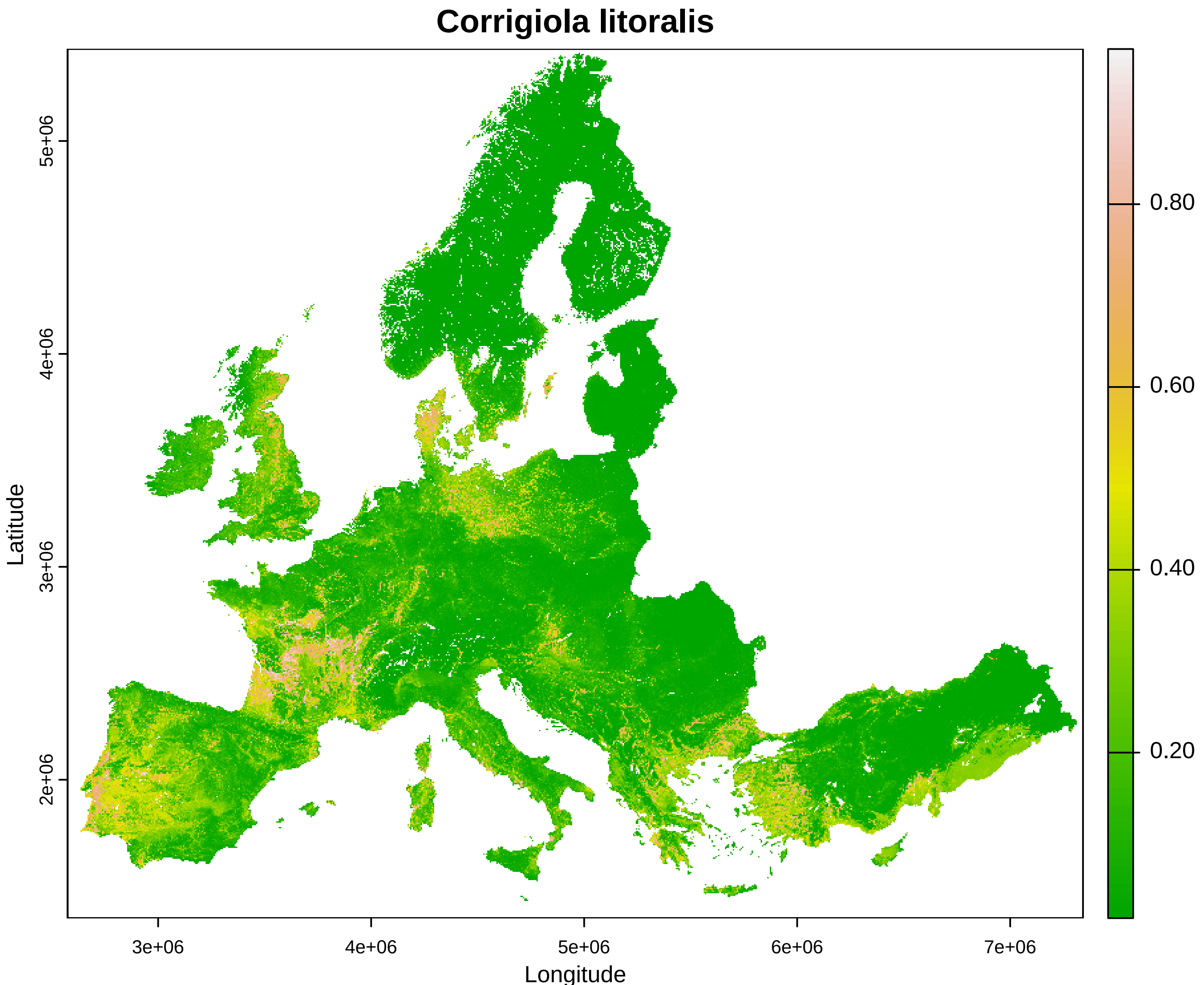
Corrigiola litoralis
Latitude
5e+06
4e+06
3e+06
2e+06
Longitude
3e+06
4e+06
5e+06
6e+06
7e+06
0.80
0.60
0.40
0.20

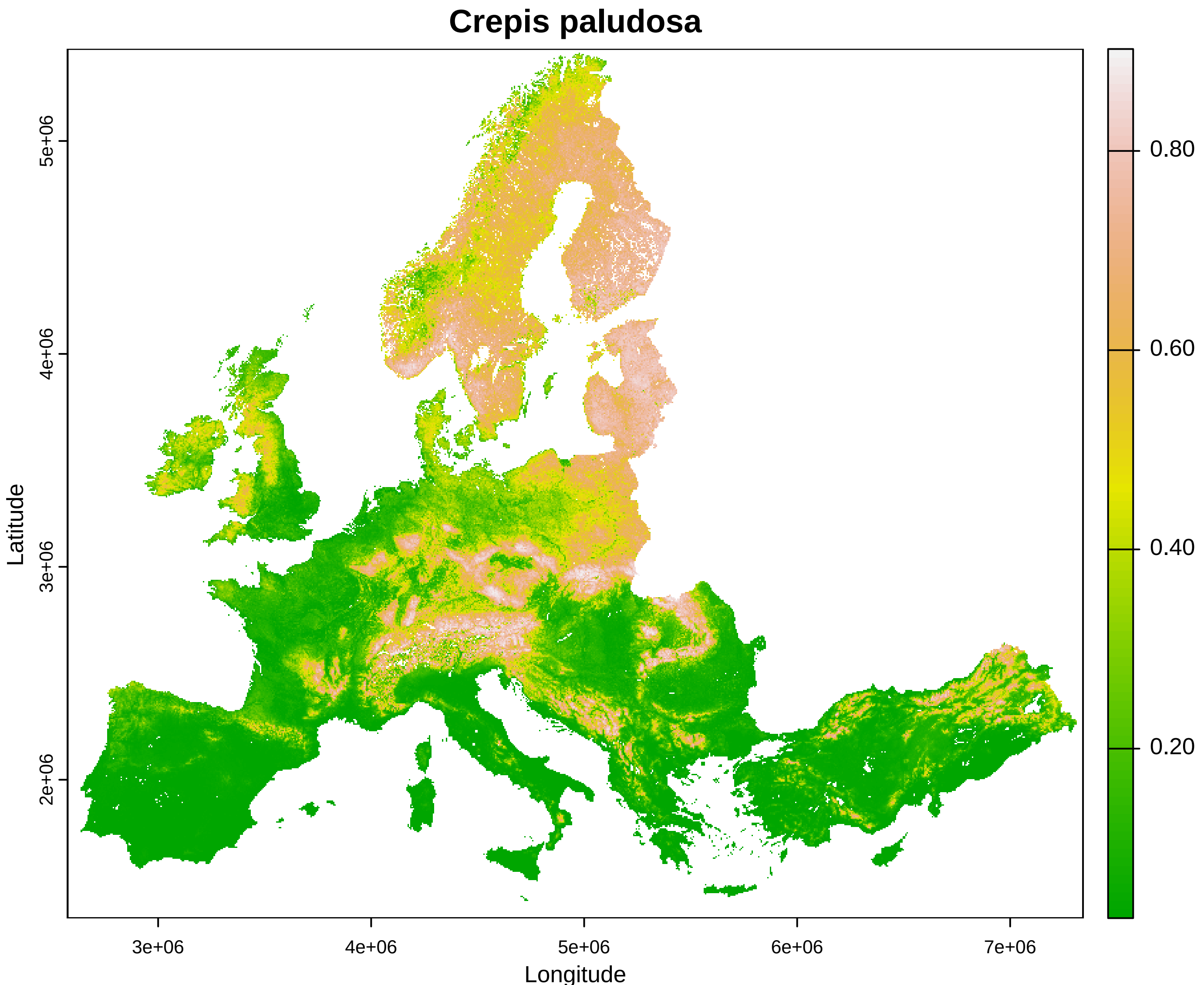

Crepis paludosa
Latitude
Longitude
5e+06
4e+06
3e+06
2e+06
3e+06
4e+06
5e+06
6e+06
7e+06
0.80
0.60
0.40
0.20

Ctenidium molluscum

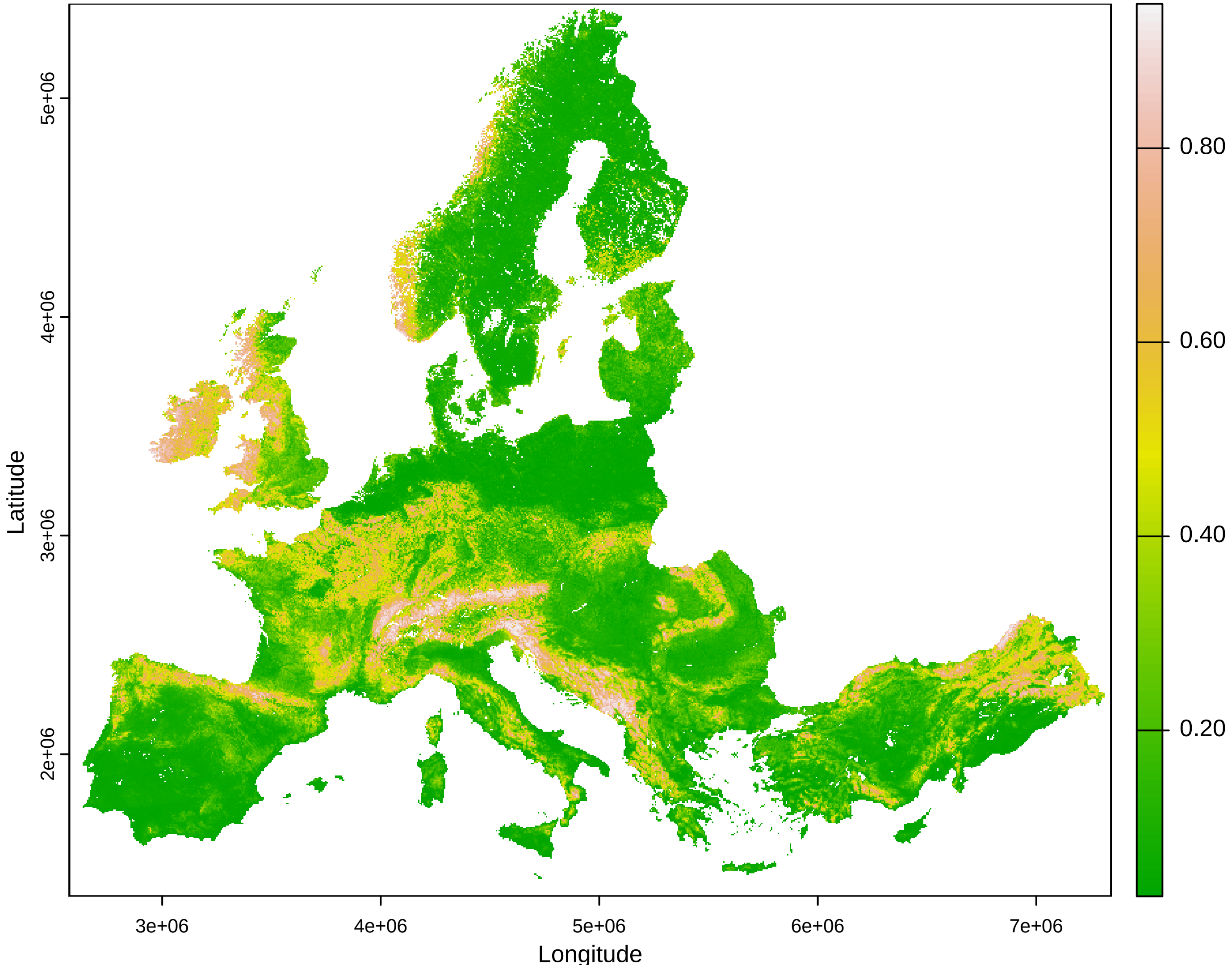

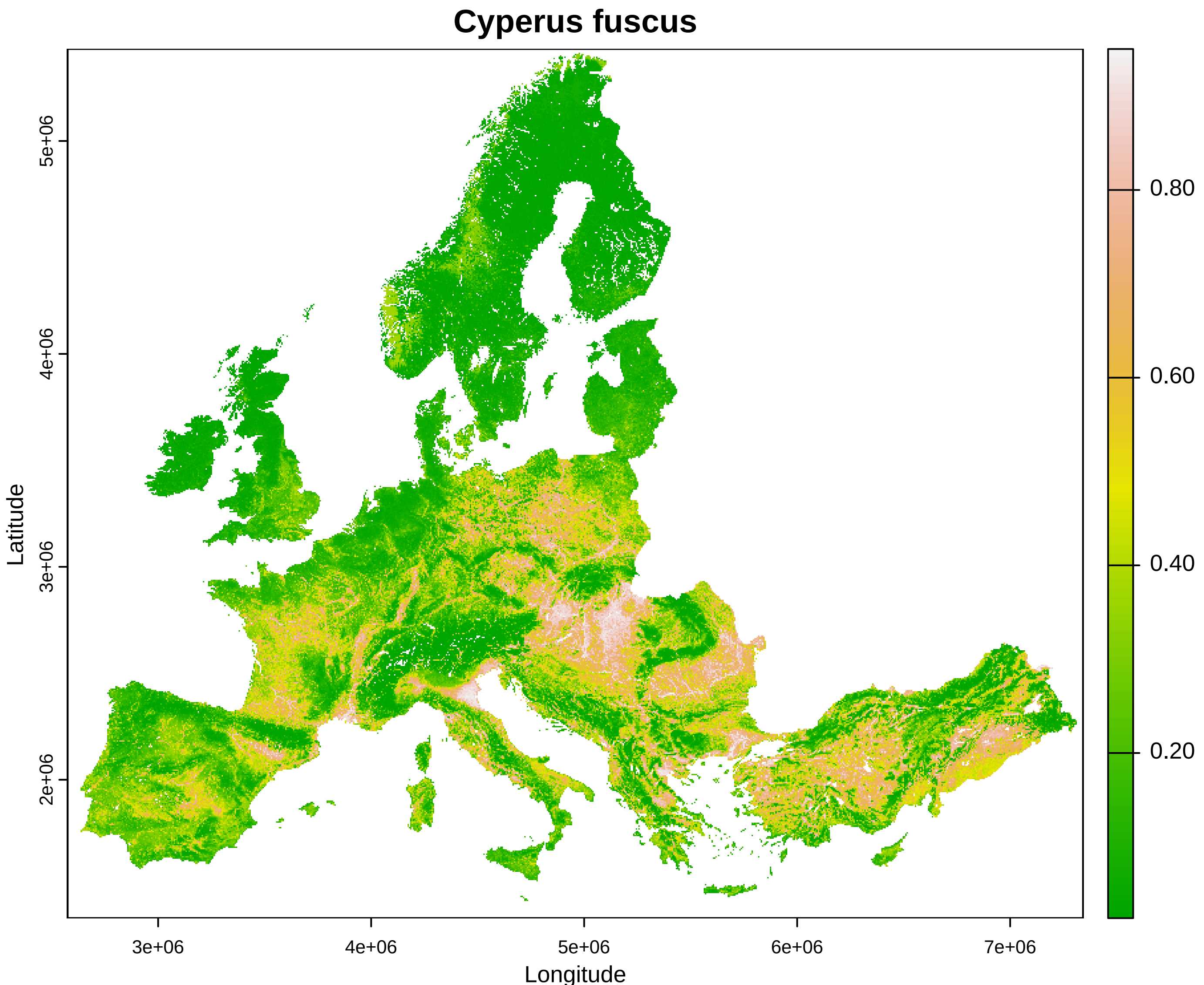

Cyperus fuscus
Latitude
Longitude
5e+06
4e+06
3e+06
2e+06
3e+06
4e+06
5e+06
6e+06
7e+06
0.80
0.60
0.40
0.20

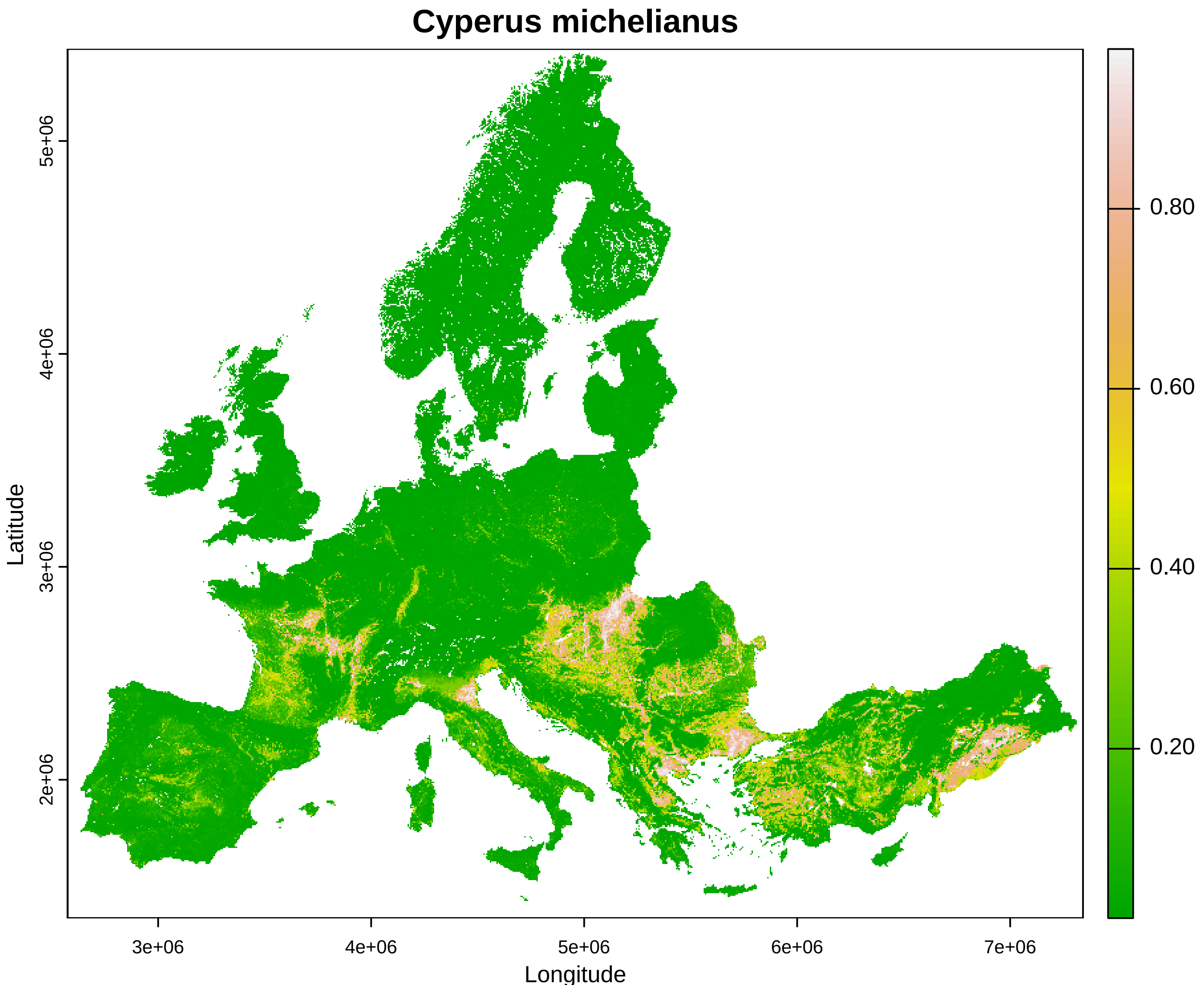

Cyperus michelianus
Latitude
5e+06
4e+06
3e+06
2e+06
Longitude
3e+06
4e+06
5e+06
6e+06
7e+06
0.80
0.60
0.40
0.20

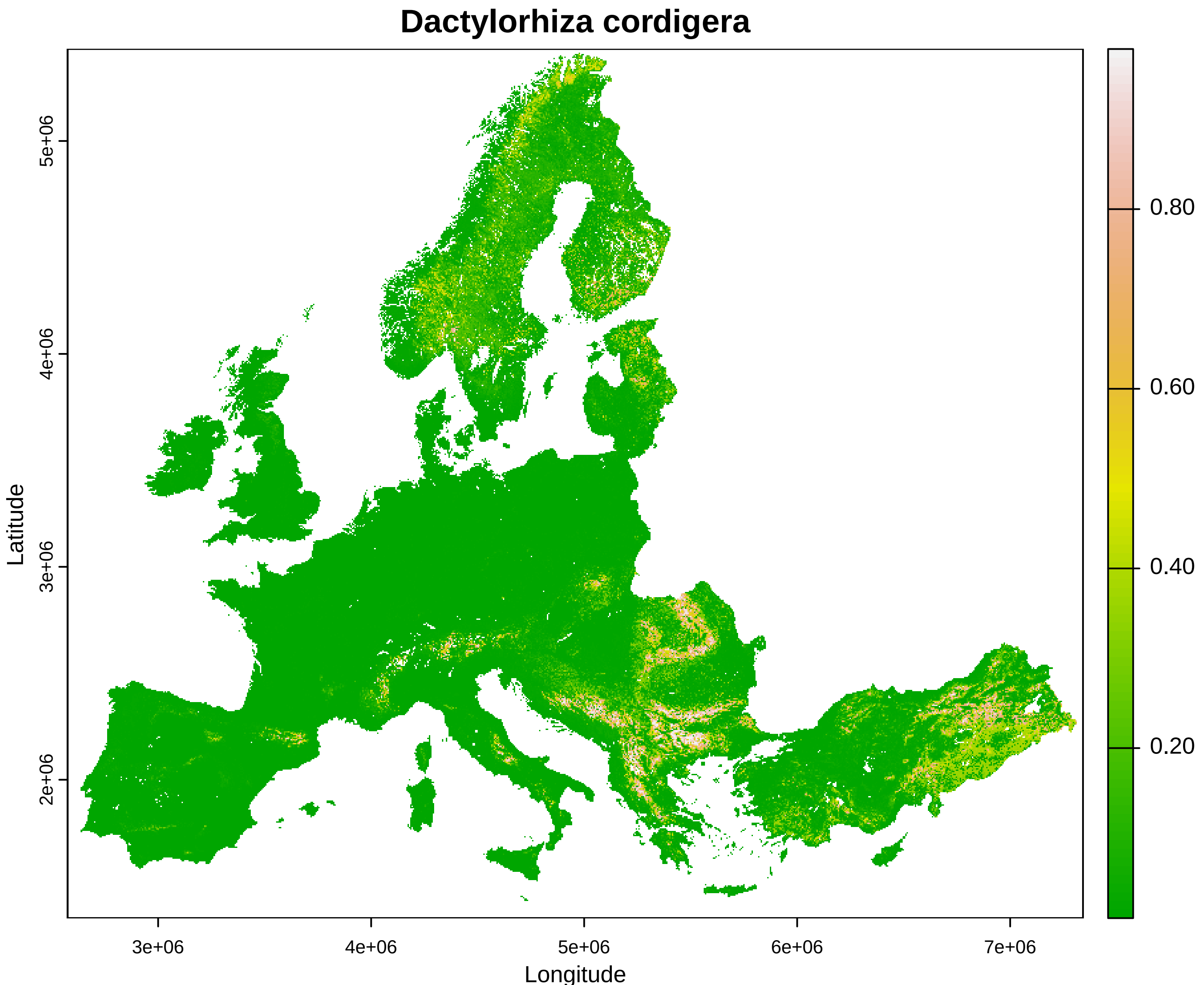
Dactylorhiza cordigera
Latitude
Longitude
5e+06
4e+06
3e+06
2e+06
3e+06
4e+06
5e+06
6e+06
7e+06
0.80
0.60
0.40
0.20

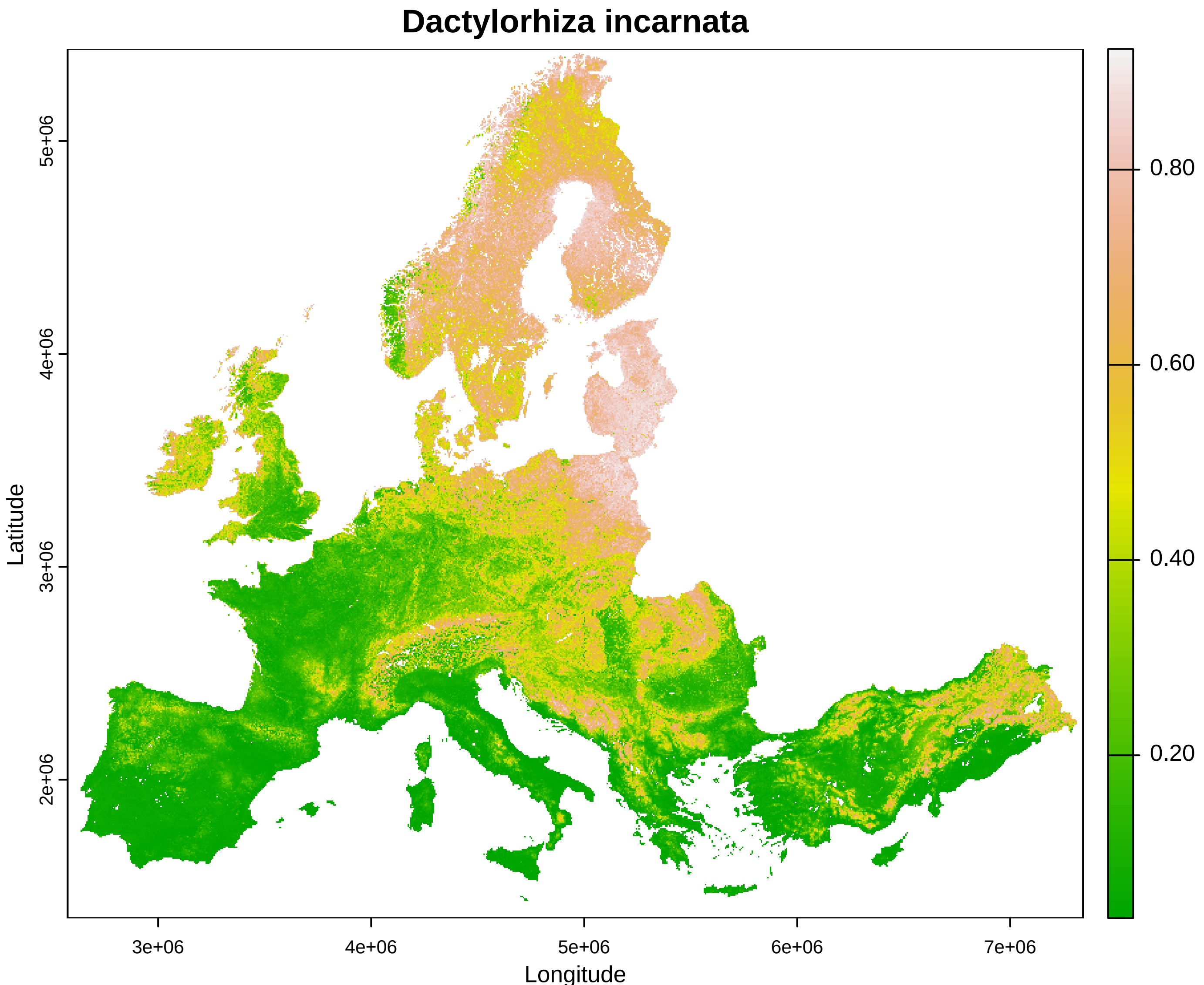
Dactylorhiza incarnata
Latitude
Longitude
5e+06
4e+06
3e+06
2e+06
3e+06
4e+06
5e+06
6e+06
7e+06
0.80
0.60
0.40
0.20

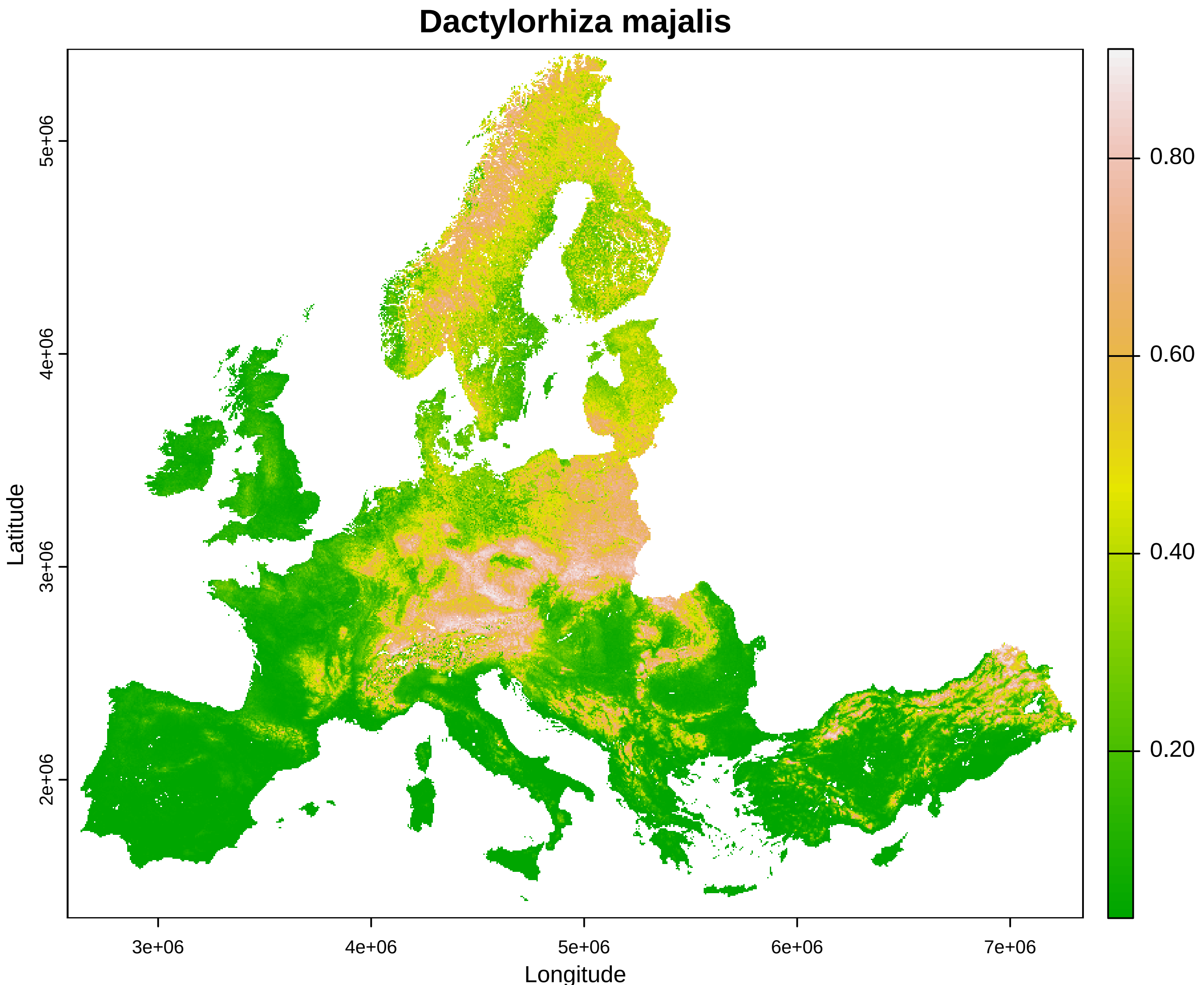
Dactylorhiza majalis
Latitude
5e+06
4e+06
3e+06
2e+06
Longitude
3e+06
4e+06
5e+06
6e+06
7e+06
0.80
0.60
0.40
0.20

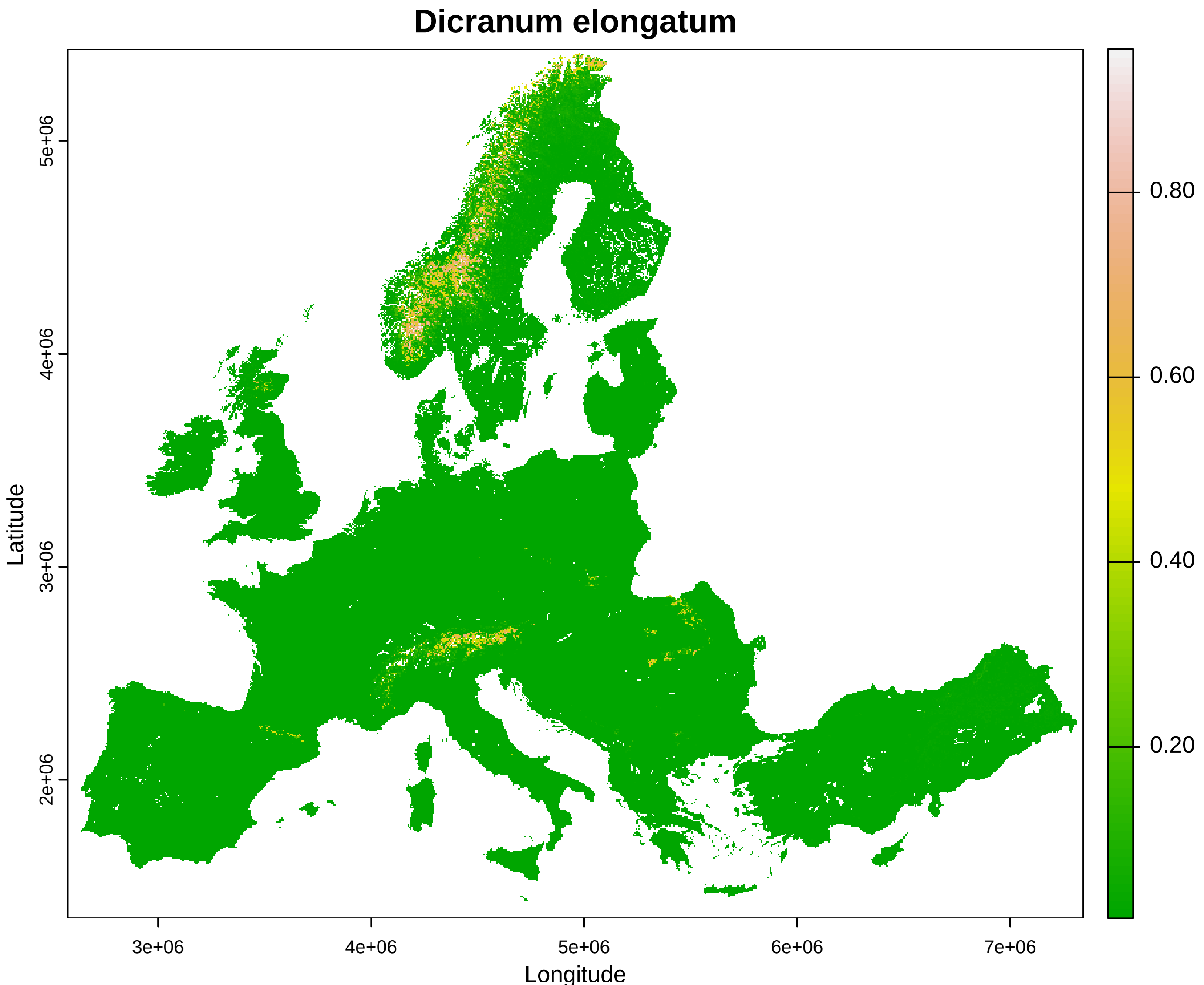
Dicranum elongatum
Latitude
Longitude
5e+06
4e+06
3e+06
2e+06
3e+06
4e+06
5e+06
6e+06
7e+06
0.80
0.60
0.40
0.20

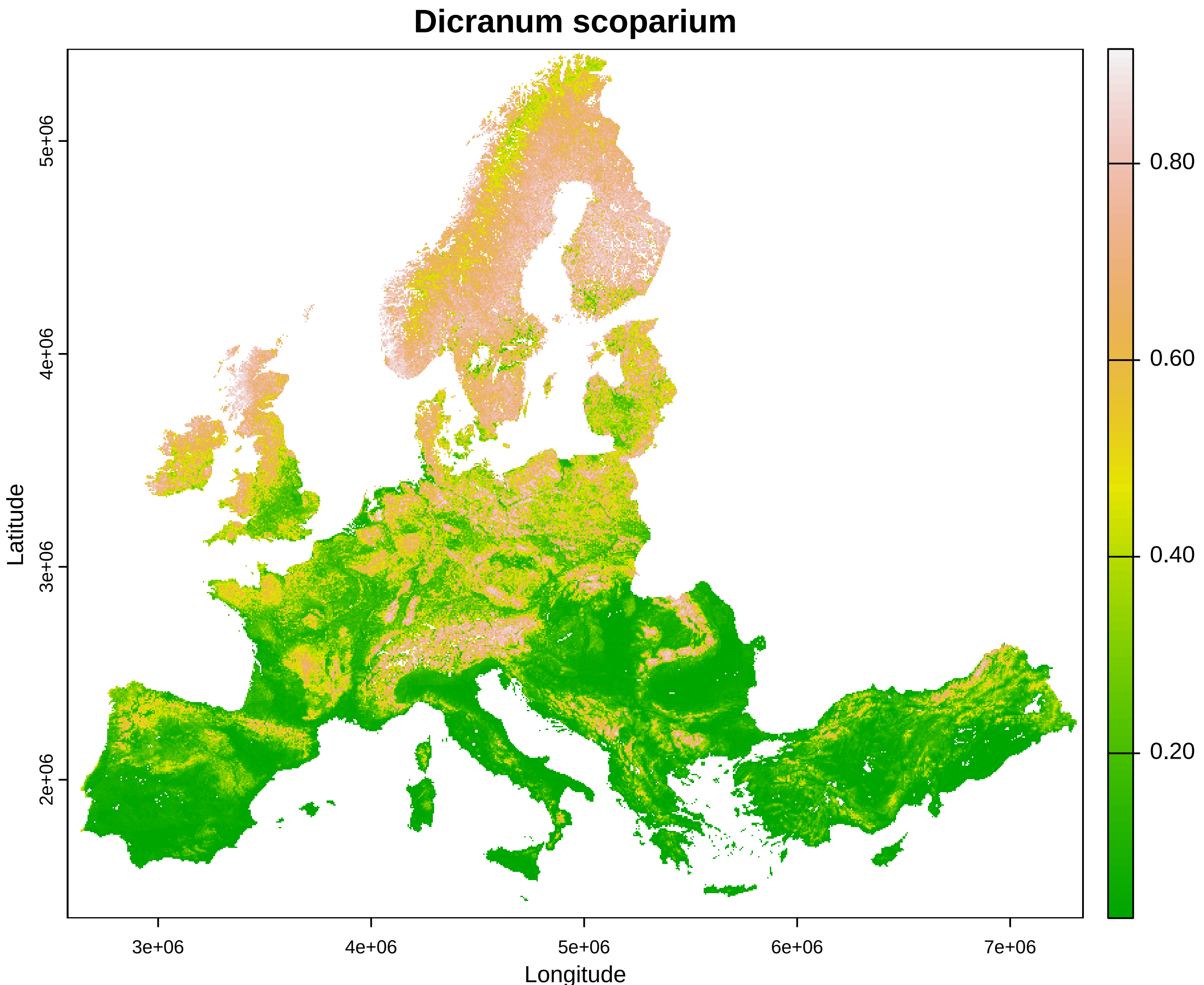
Dicranum scoparium
Latitude
Longitude
5e+06
4e+06
3e+06
2e+06
3e+06
4e+06
5e+06
6e+06
7e+06
0.80
0.60
0.40
0.20

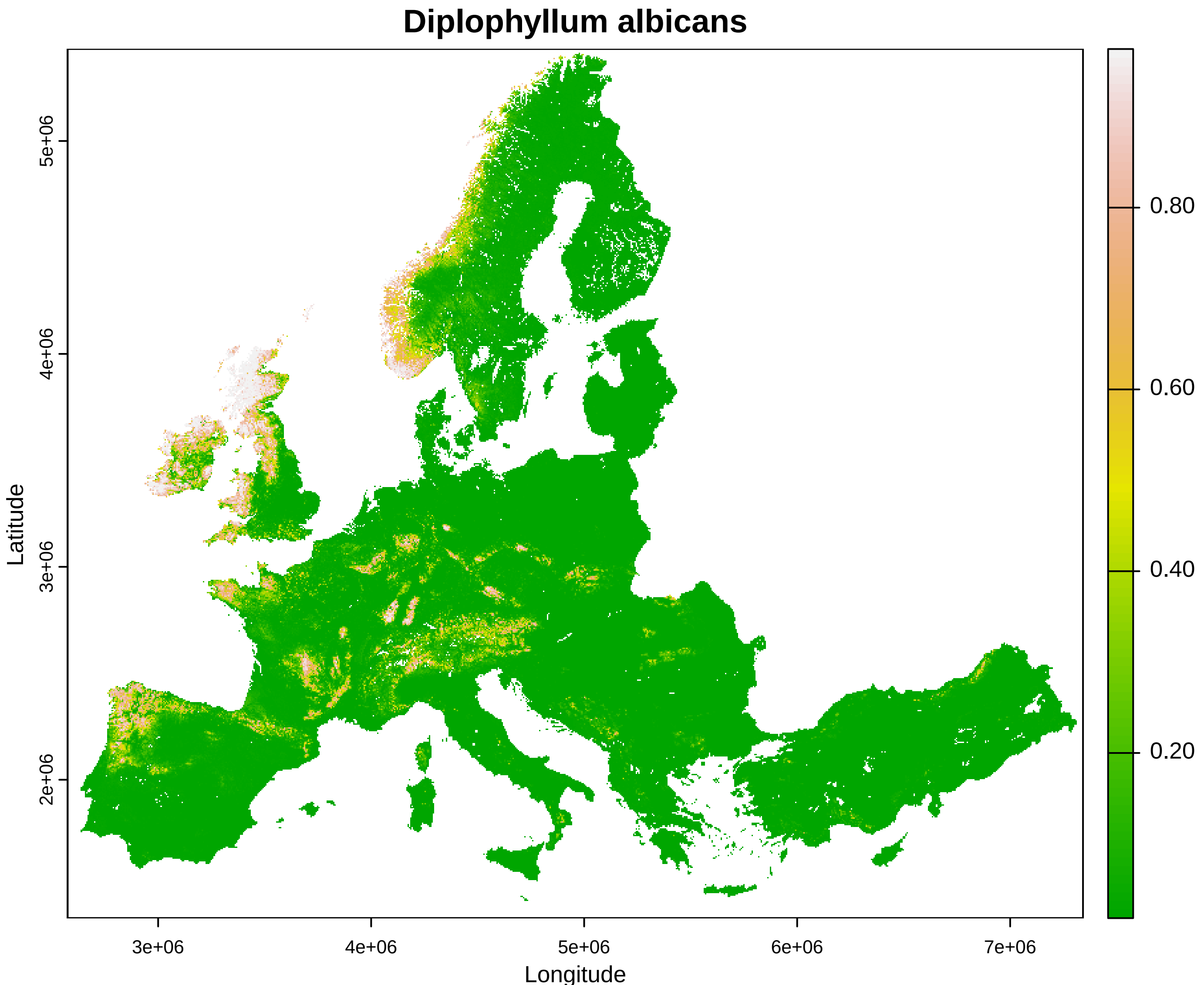

Diplophyllum albicans
Latitude
5e+06
4e+06
3e+06
2e+06
3e+06
4e+06
5e+06
6e+06
7e+06
Longitude
0.80
0.60
0.40
0.20

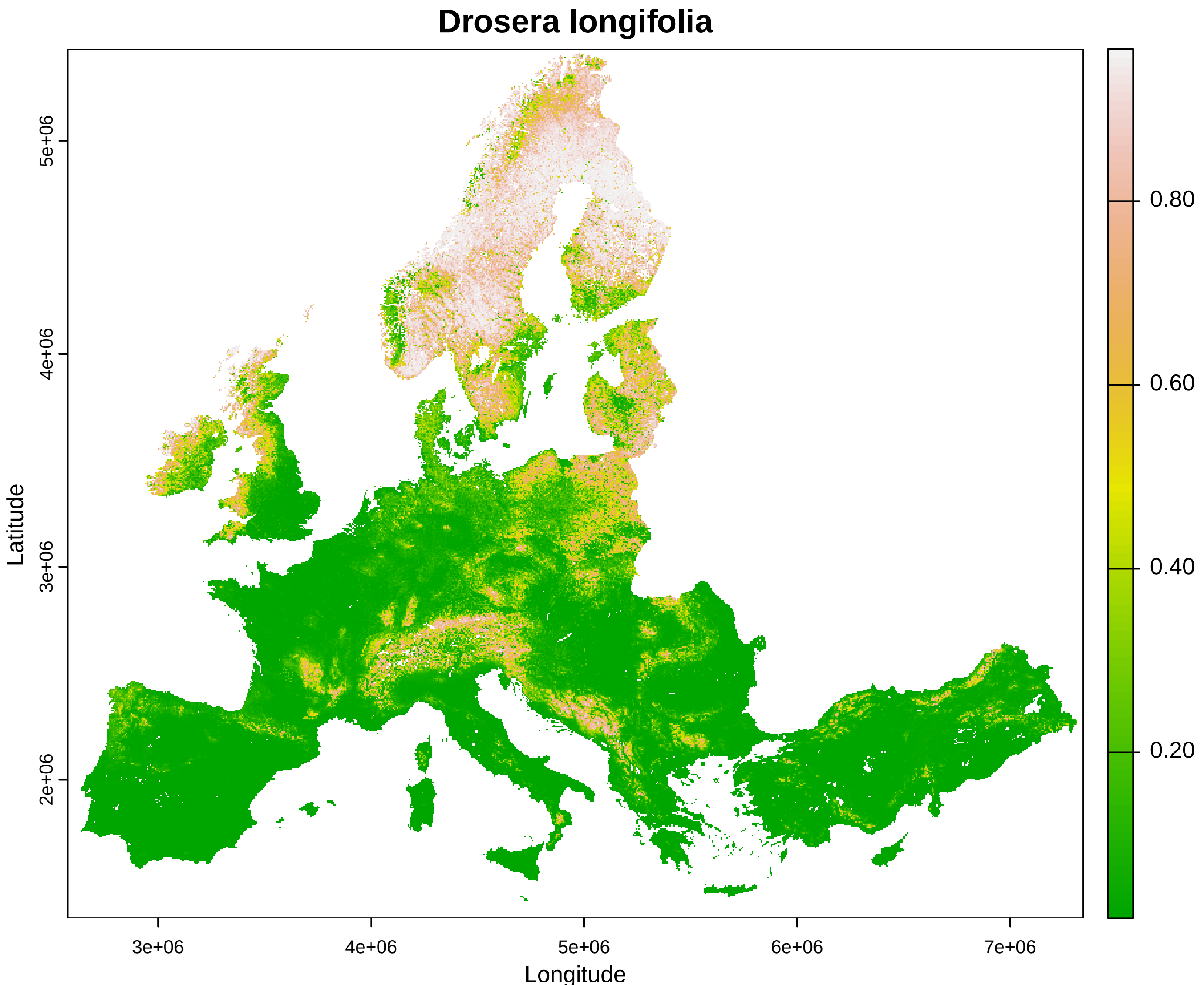
Drosera longifolia
Latitude
Longitude
5e+06
4e+06
3e+06
2e+06
3e+06
4e+06
5e+06
6e+06
7e+06
0.80
0.60
0.40
0.20

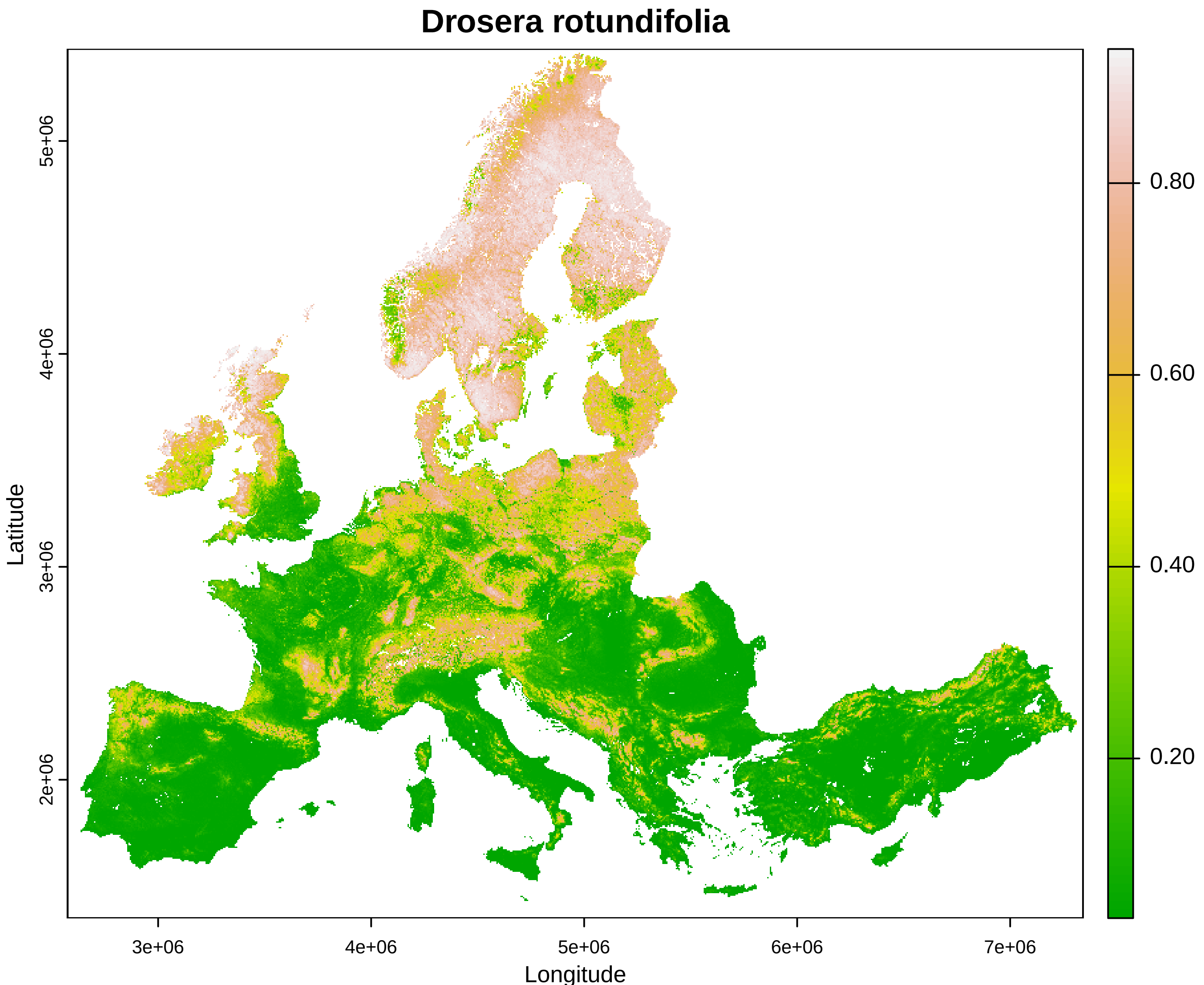

Drosera rotundifolia
5e+06
4e+06
3e+06
2e+06
Latitude
3e+06
4e+06
5e+06
6e+06
7e+06
Longitude
0.80
0.60
0.40
0.20

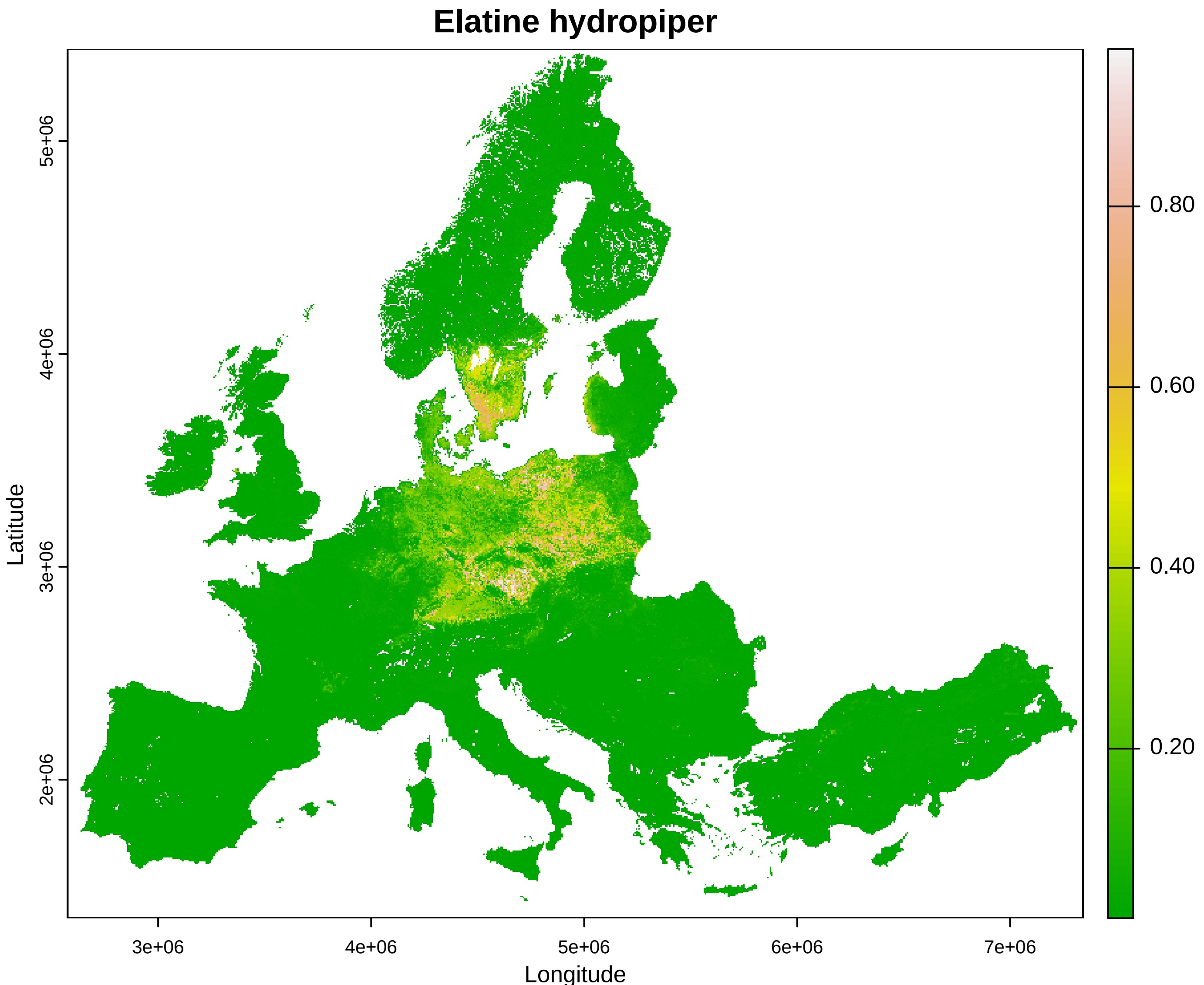
Elatine hydropiper
Latitude
5e+06
4e+06
3e+06
2e+06
3e+06
4e+06
5e+06
6e+06
7e+06
Longitude
0.80
0.60
0.40
0.20

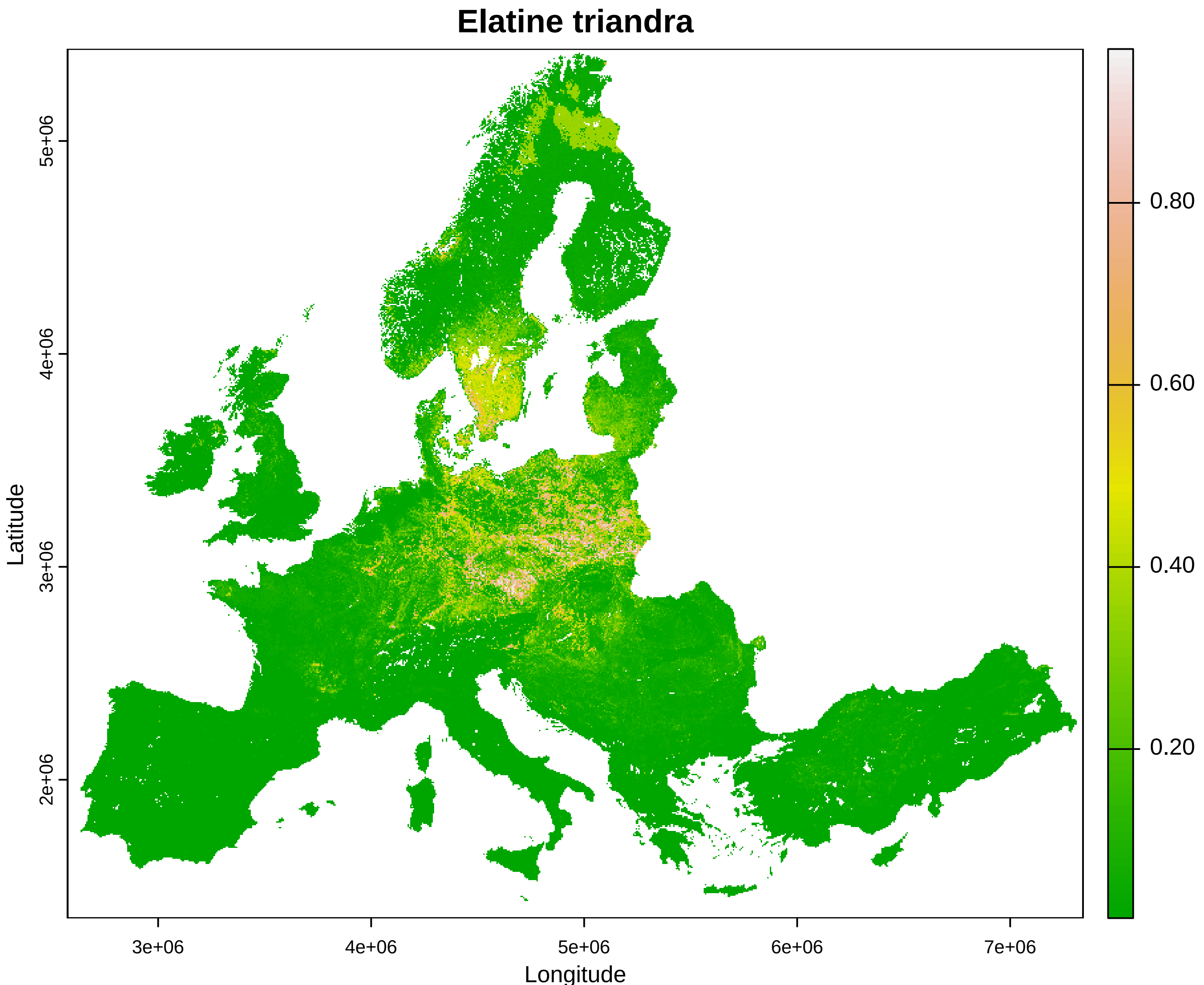
Elatine triandra
Latitude
Longitude
5e+06
4e+06
3e+06
2e+06
3e+06
4e+06
5e+06
6e+06
7e+06
0.80
0.60
0.40
0.20

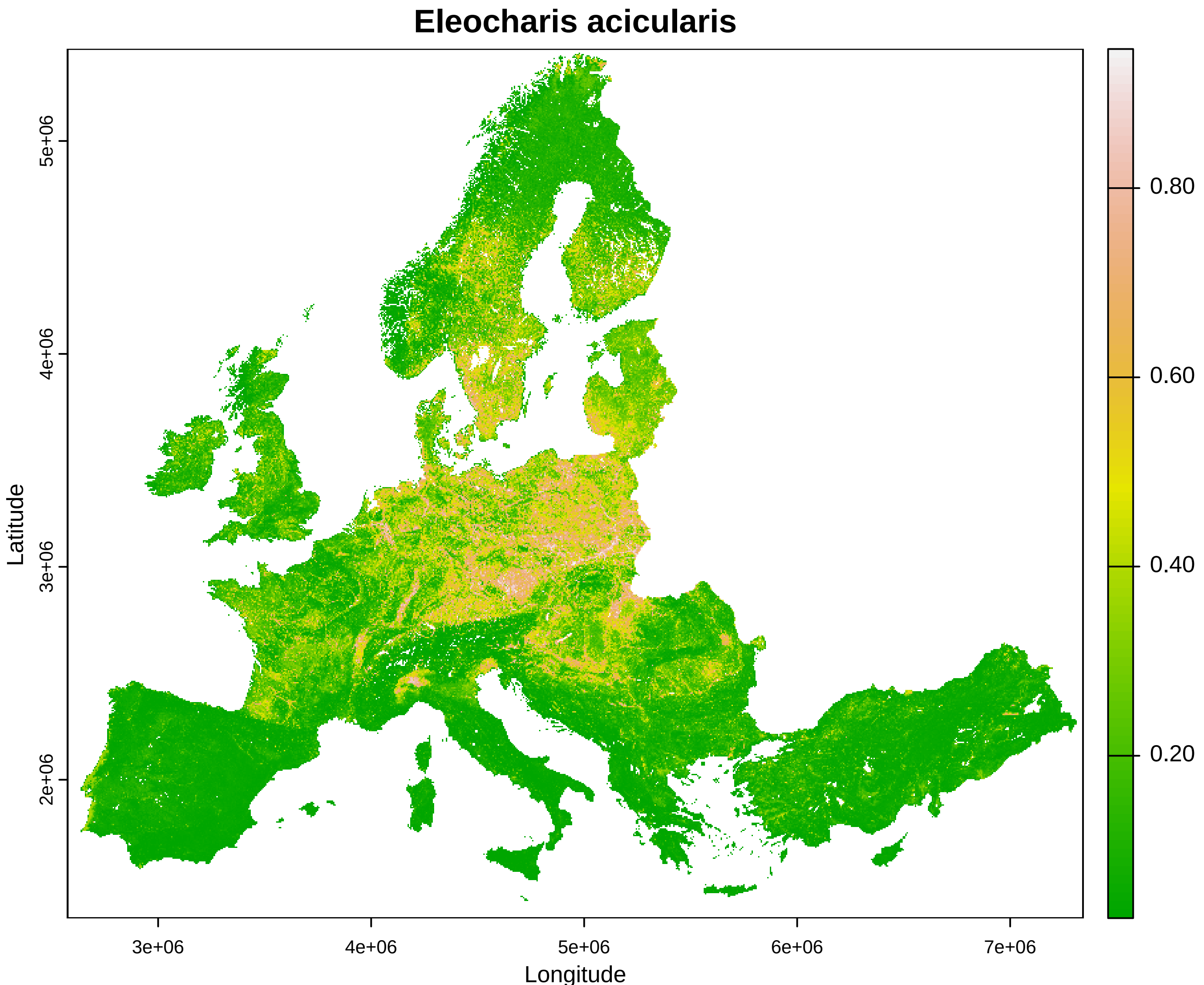

Eleocharis acicularis
Latitude
5e+06
4e+06
3e+06
2e+06
Longitude
3e+06
4e+06
5e+06
6e+06
7e+06
0.80
0.60
0.40
0.20

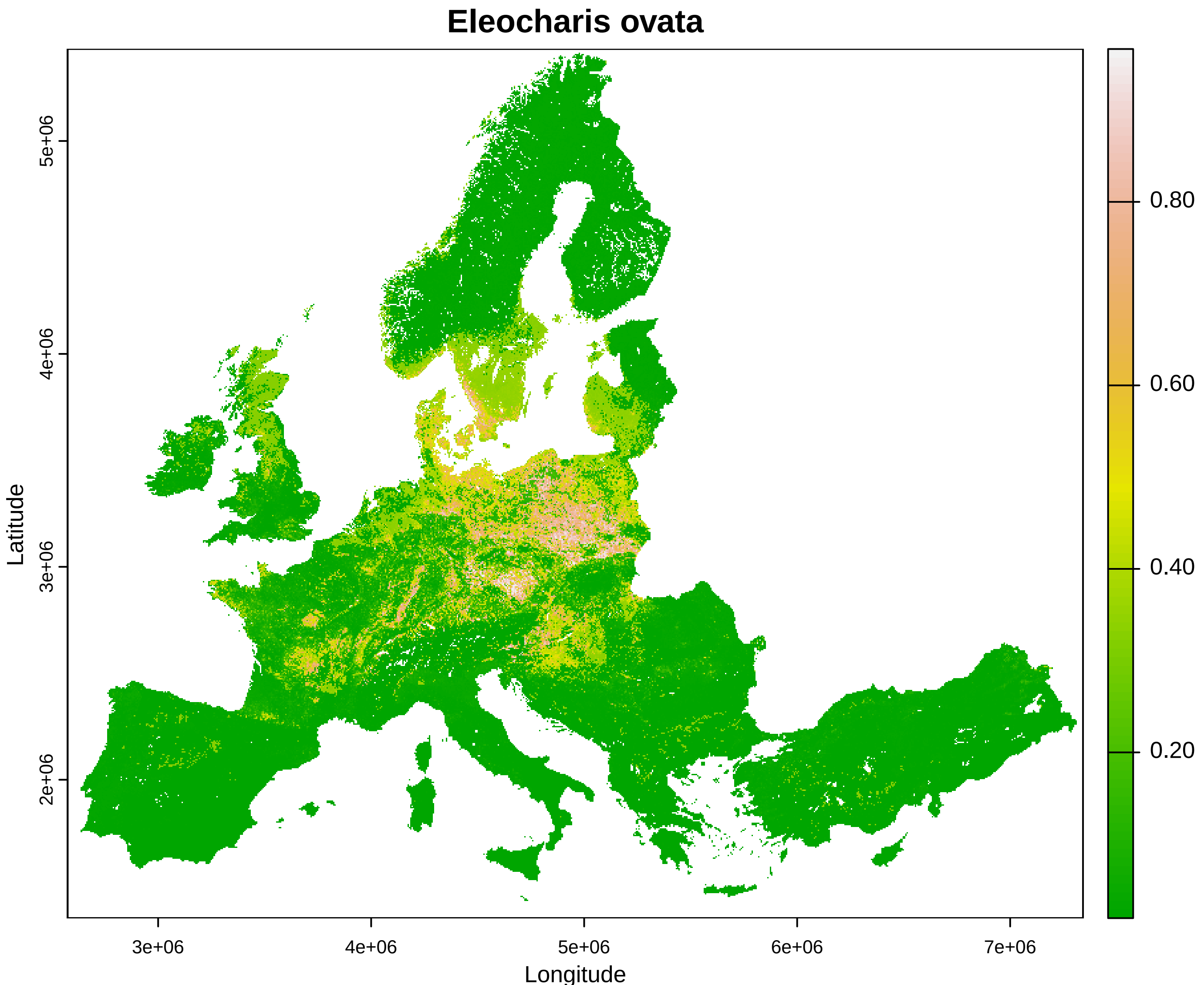
Eleocharis ovata
Latitude
Longitude
5e+06
4e+06
3e+06
2e+06
3e+06
4e+06
5e+06
6e+06
7e+06
0.80
0.60
0.40
0.20

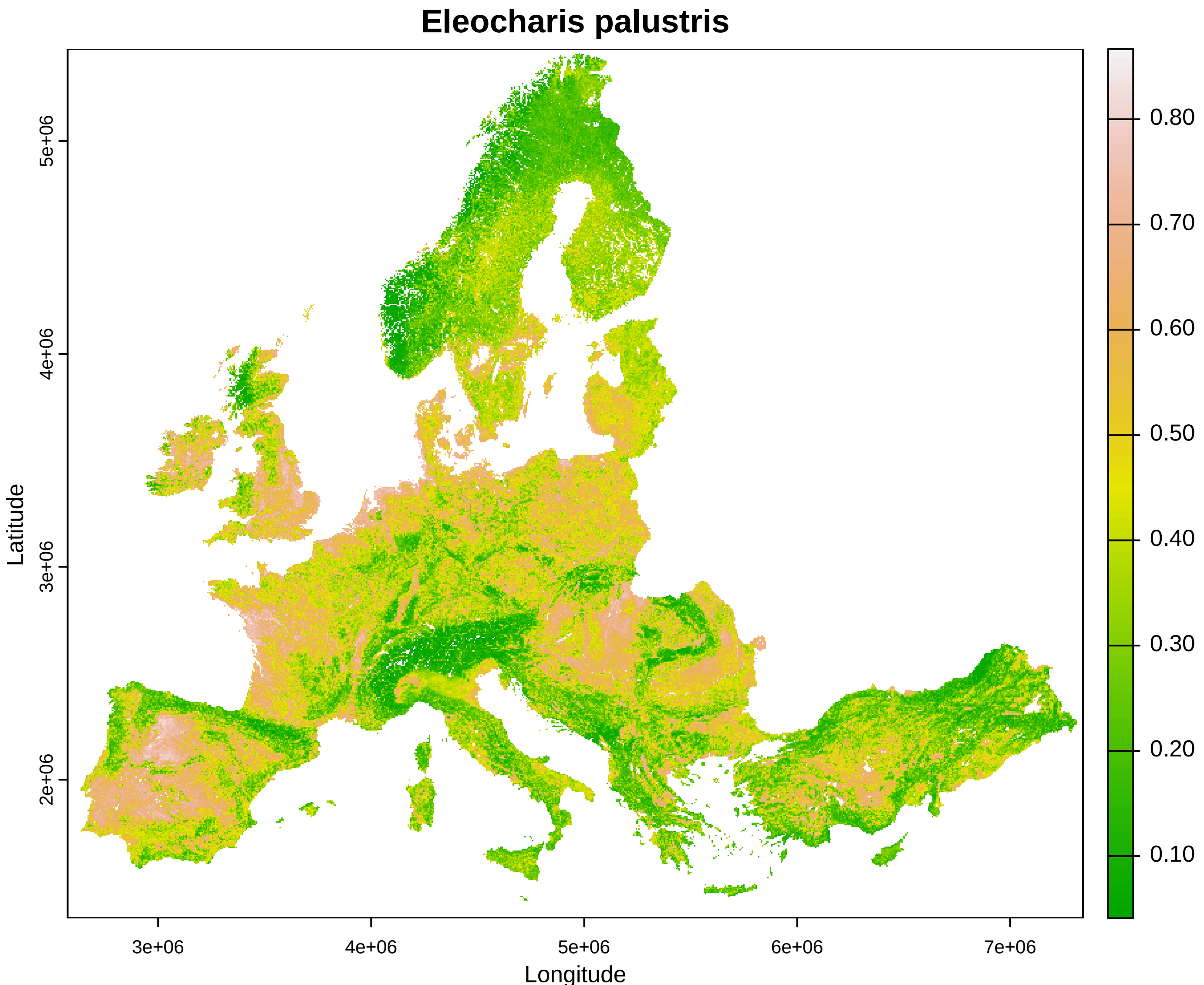

Eleocharis palustris
Latitude
Longitude
5e+06
4e+06
3e+06
2e+06
3e+06
4e+06
5e+06
6e+06
7e+06
0.80
0.70
0.60
0.50
0.40
0.30
0.20
0.10

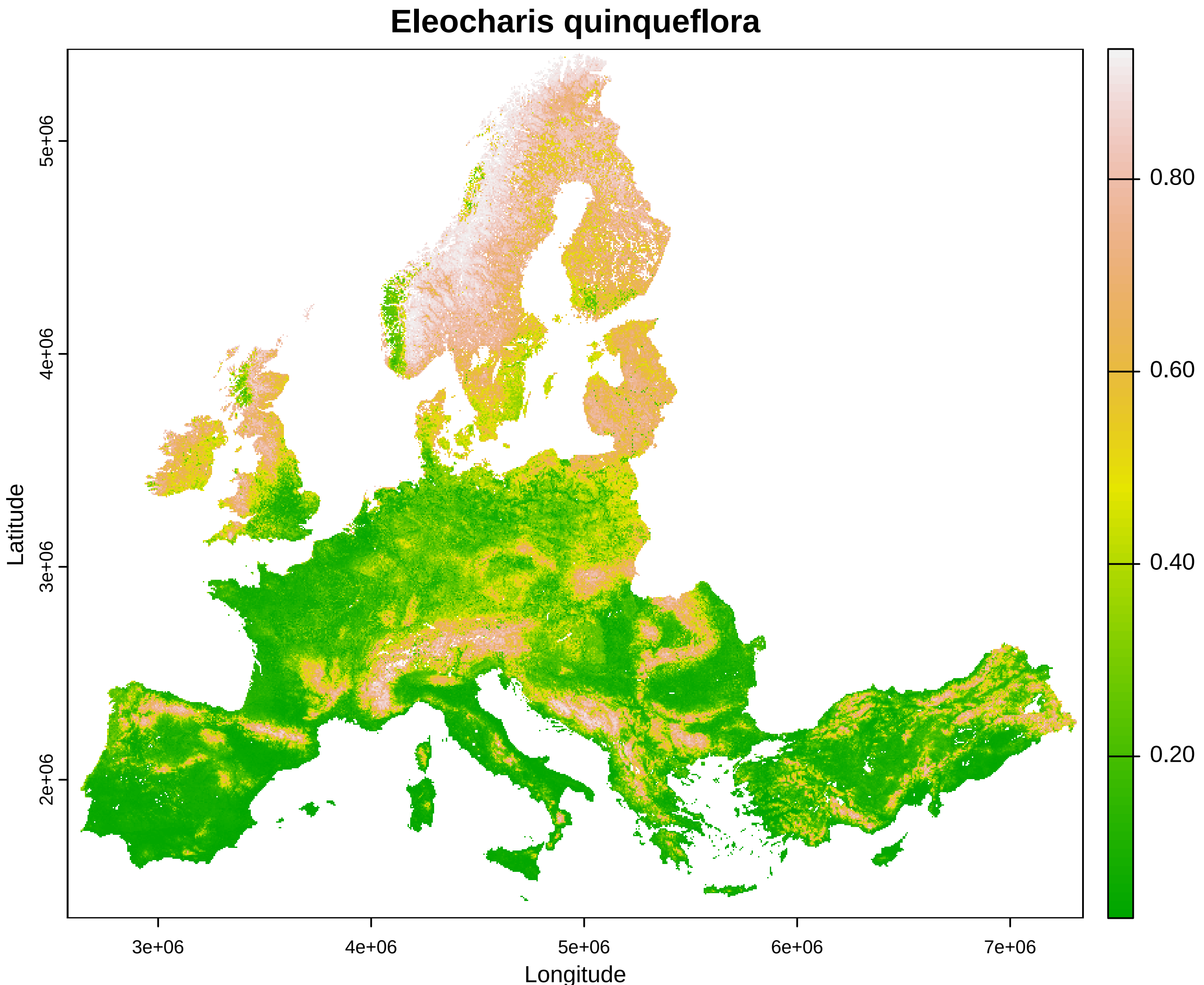

Eleocharis quinqueflora
Latitude
5e+06
4e+06
3e+06
2e+06
3e+06
4e+06
5e+06
6e+06
7e+06
Longitude
0.80
0.60
0.40
0.20

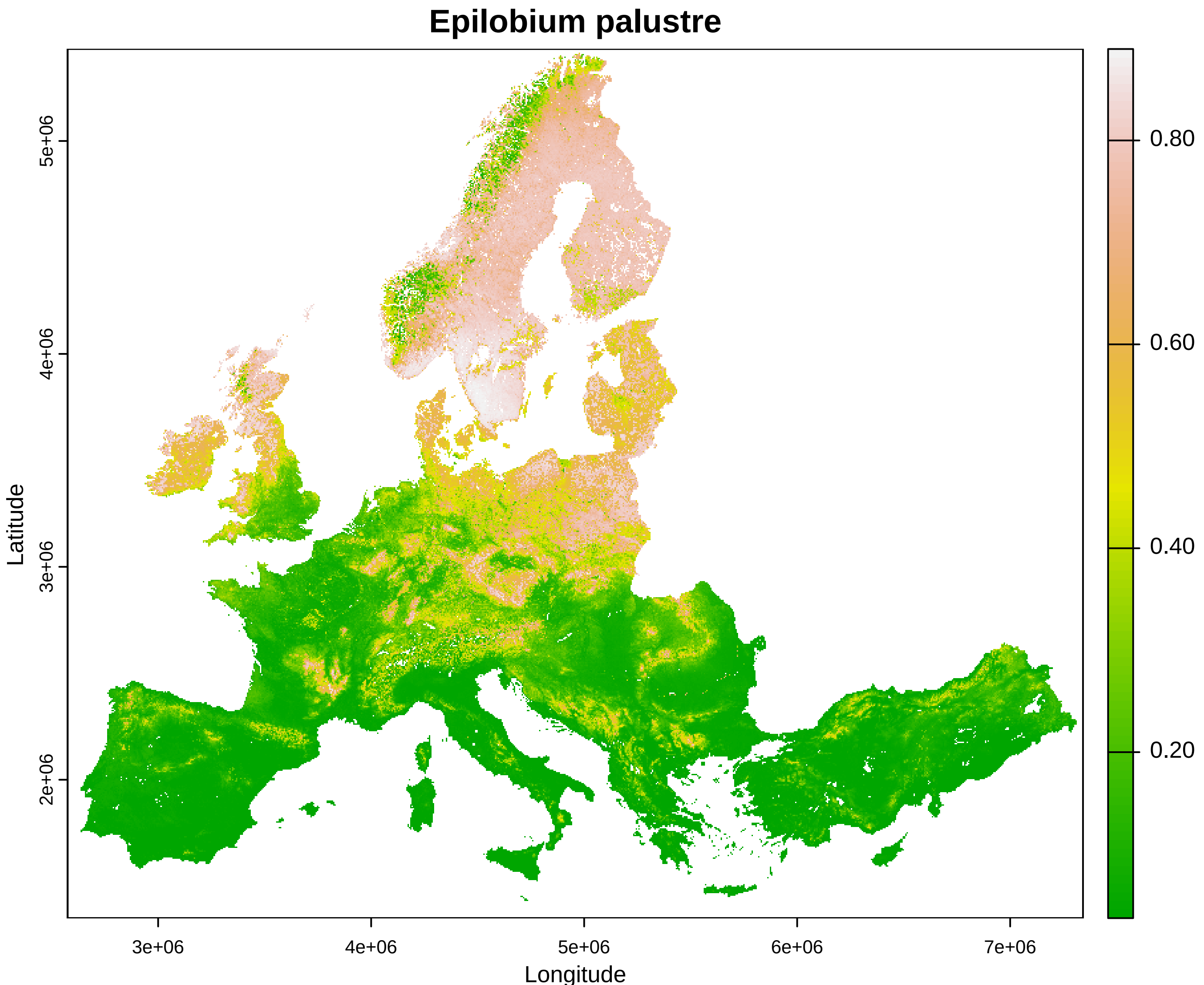

Epilobium palustre
Latitude
Longitude
5e+06
4e+06
3e+06
2e+06
3e+06
4e+06
5e+06
6e+06
7e+06
0.80
0.60
0.40
0.20

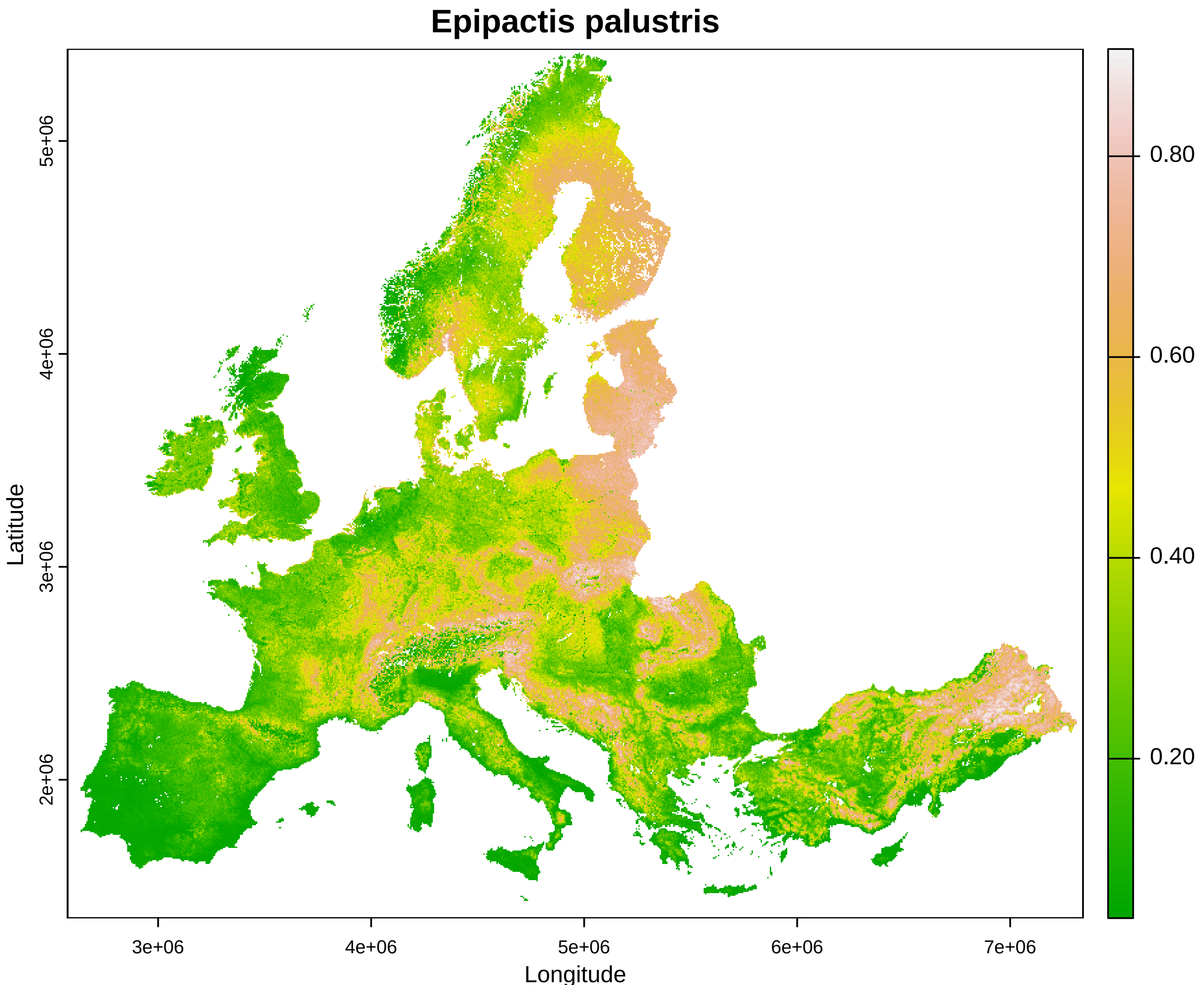
Epipactis palustris
Latitude
Longitude
5e+06
4e+06
3e+06
2e+06
3e+06
4e+06
5e+06
6e+06
7e+06
0.80
0.60
0.40
0.20

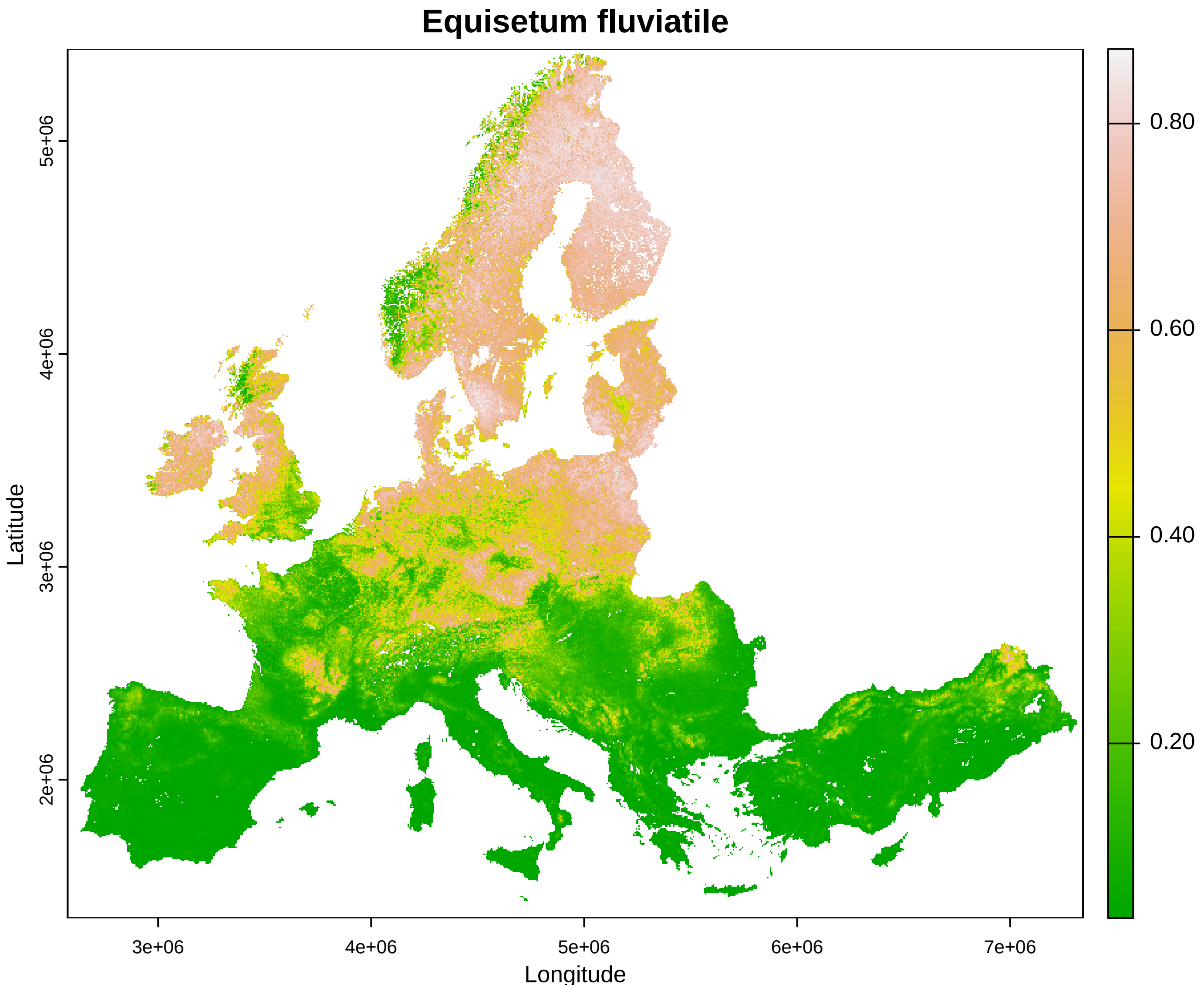

Equisetum fluviatile
Latitude
Longitude
5e+06
4e+06
3e+06
2e+06
3e+06
4e+06
5e+06
6e+06
7e+06
0.80
0.60
0.40
0.20

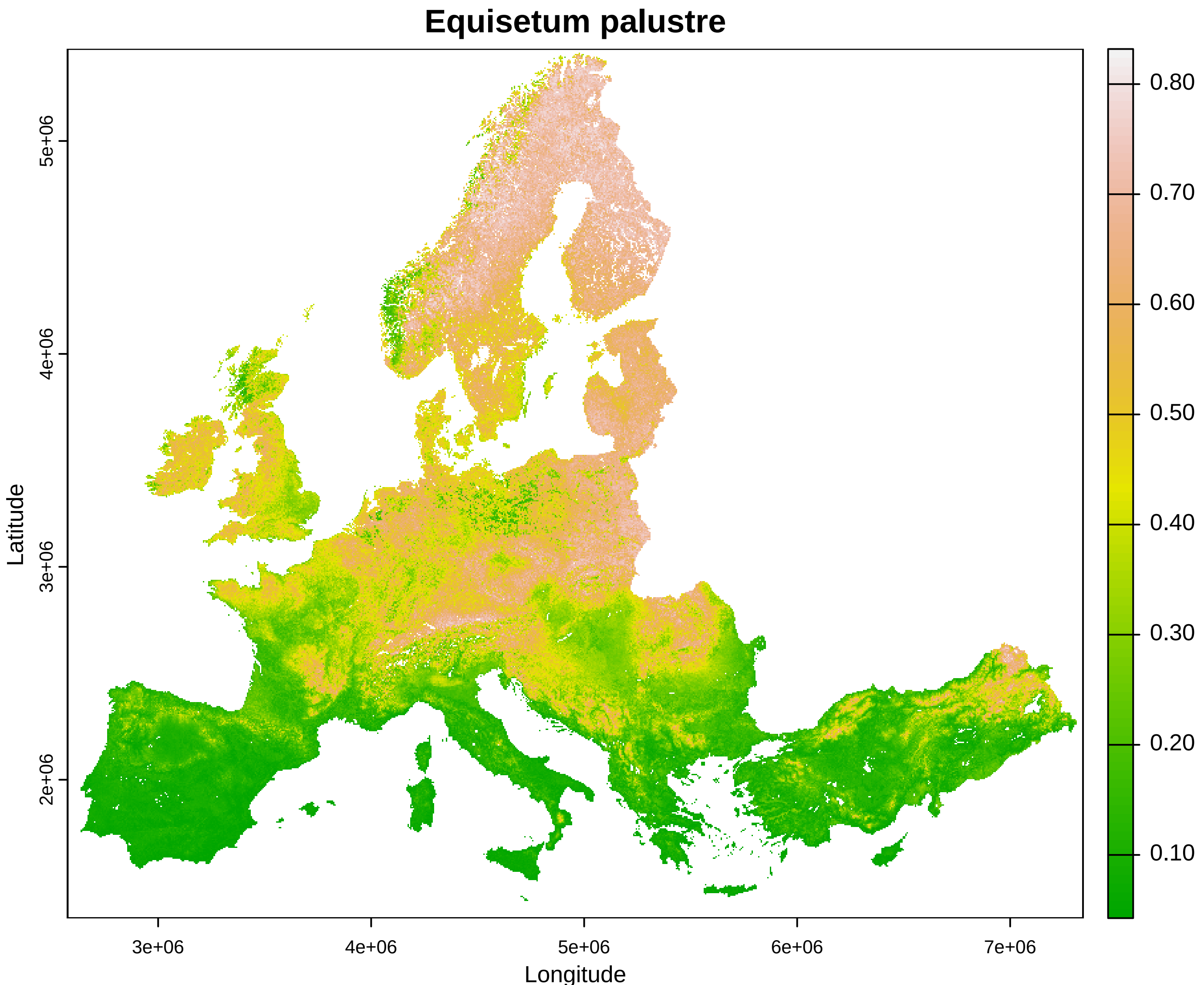

Equisetum palustre
Latitude
Longitude
5e+06
4e+06
3e+06
2e+06
3e+06
4e+06
5e+06
6e+06
7e+06
0.80
0.70
0.60
0.50
0.40
0.30
0.20
0.10

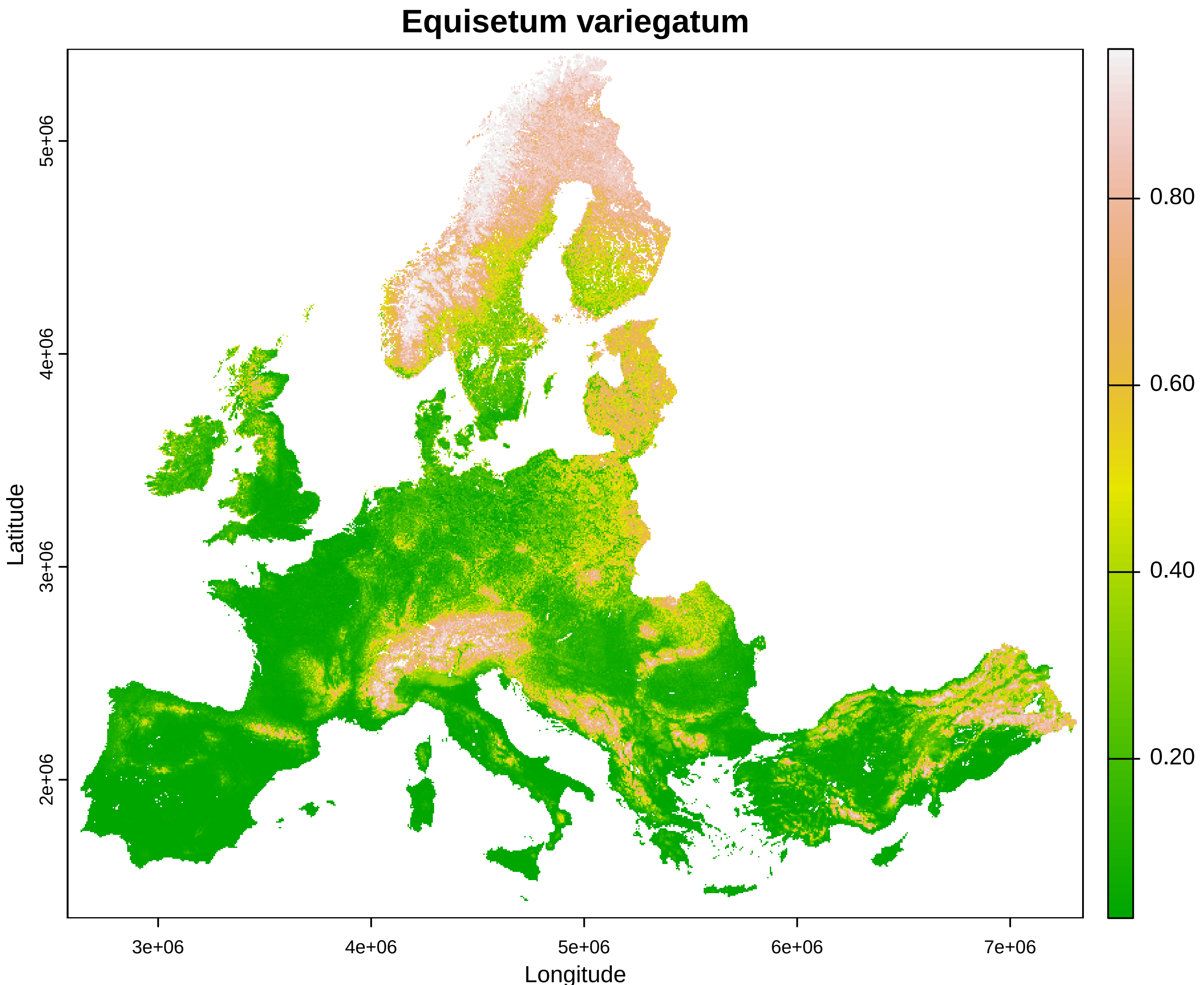

Equisetum variegatum
Latitude
Longitude
5e+06
4e+06
3e+06
2e+06
3e+06
4e+06
5e+06
6e+06
7e+06
0.80
0.60
0.40
0.20

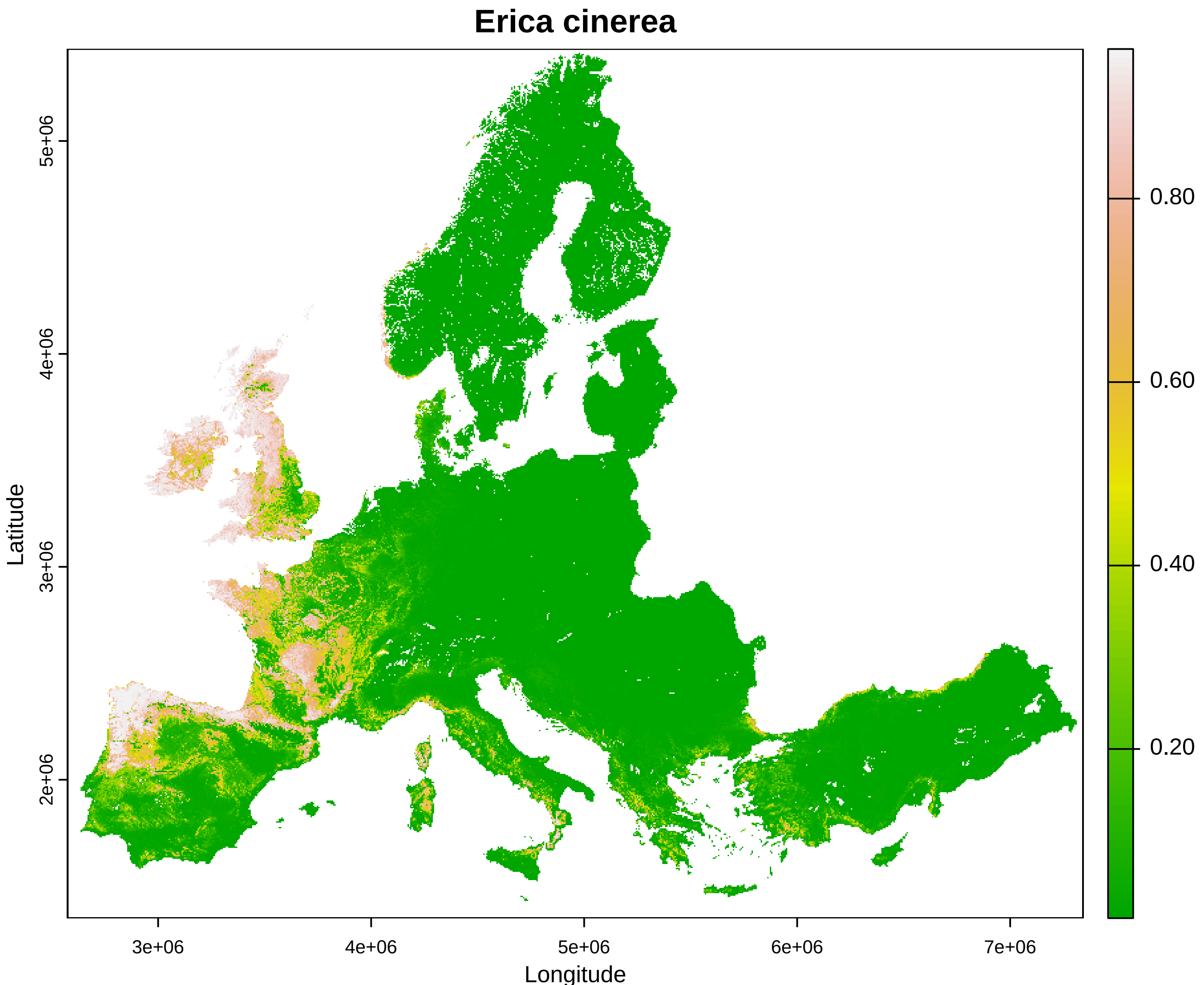

Erica cinerea
Latitude
Longitude
5e+06
4e+06
3e+06
2e+06
3e+06
4e+06
5e+06
6e+06
7e+06
0.80
0.60
0.40
0.20

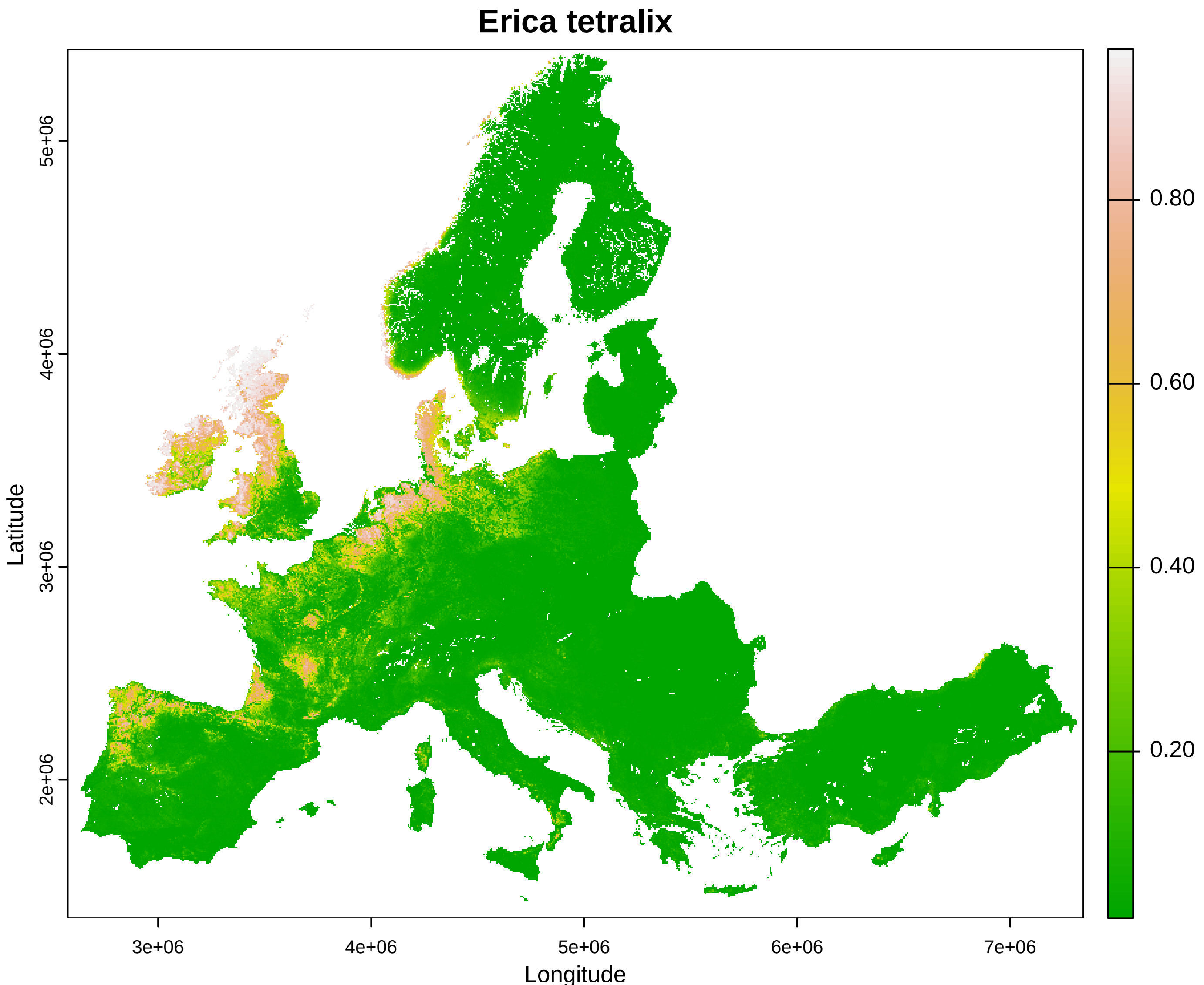
Erica tetralix
Latitude
5e+06
4e+06
3e+06
2e+06
Longitude
3e+06
4e+06
5e+06
6e+06
7e+06
0.80
0.60
0.40
0.20

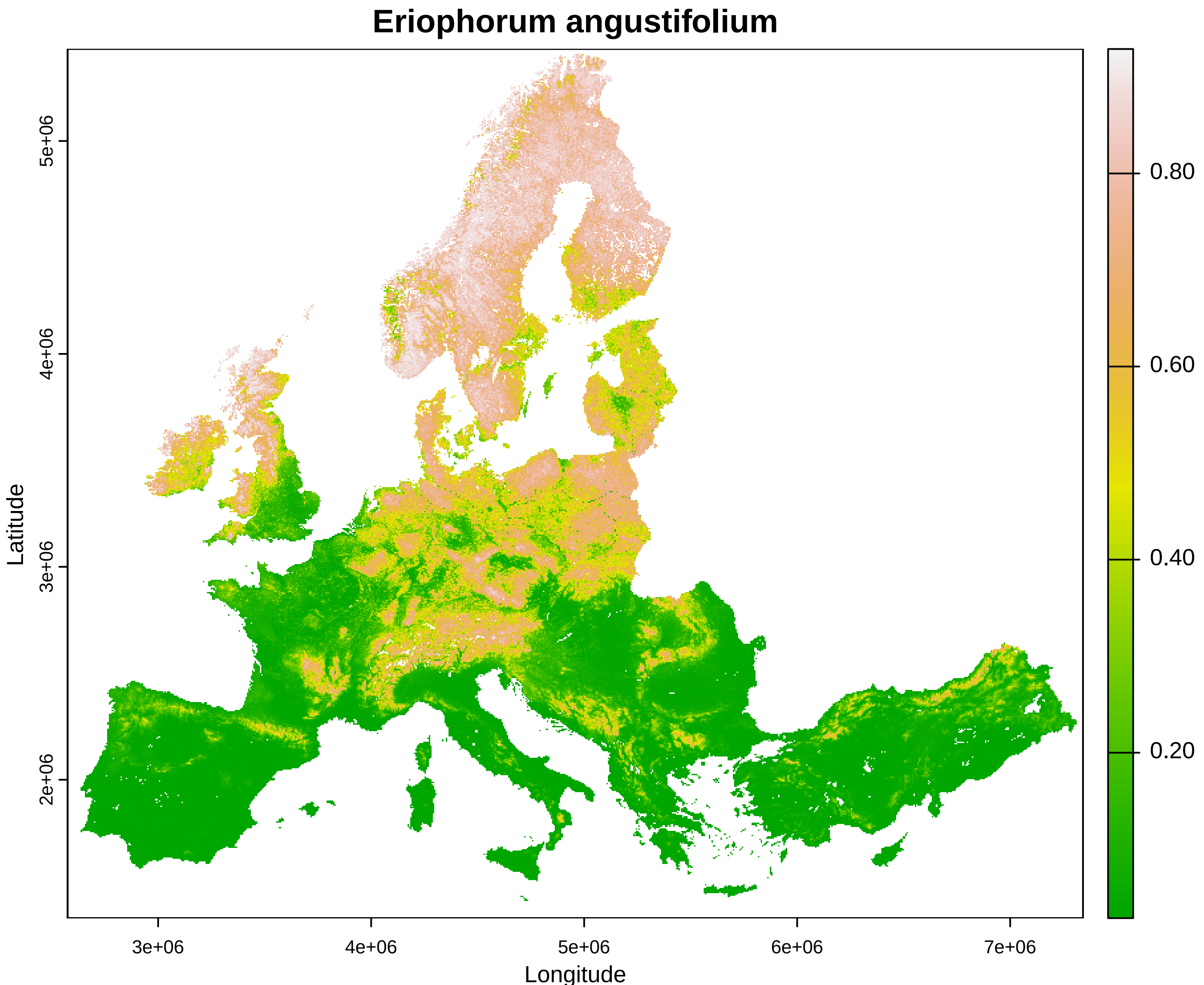
Eriophorum angustifolium
Latitude
Longitude
5e+06
4e+06
3e+06
2e+06
3e+06
4e+06
5e+06
6e+06
7e+06
0.80
0.60
0.40
0.20

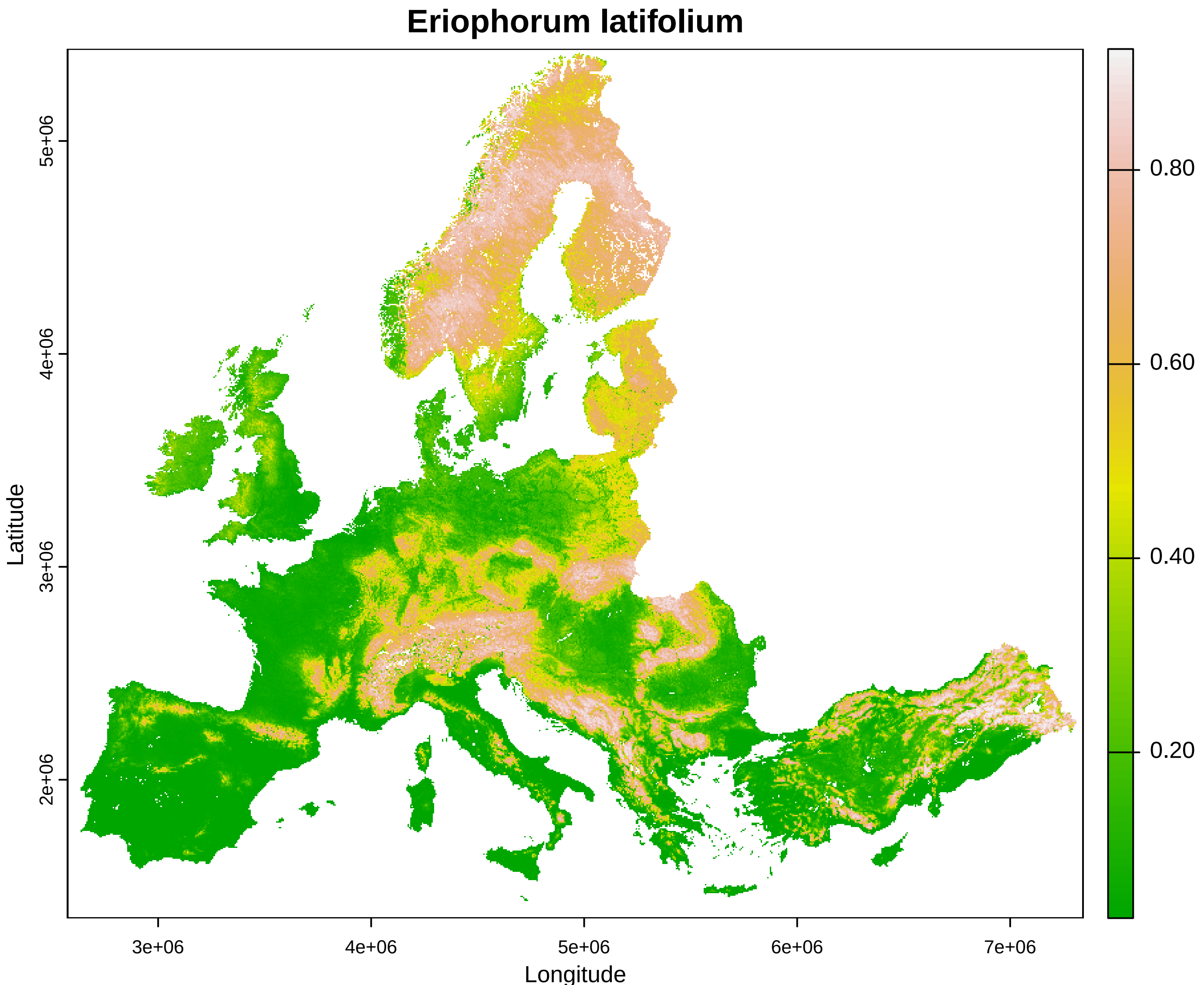

Eriophorum latifolium
Latitude
Longitude
5e+06
4e+06
3e+06
2e+06
3e+06
4e+06
5e+06
6e+06
7e+06
0.80
0.60
0.40
0.20

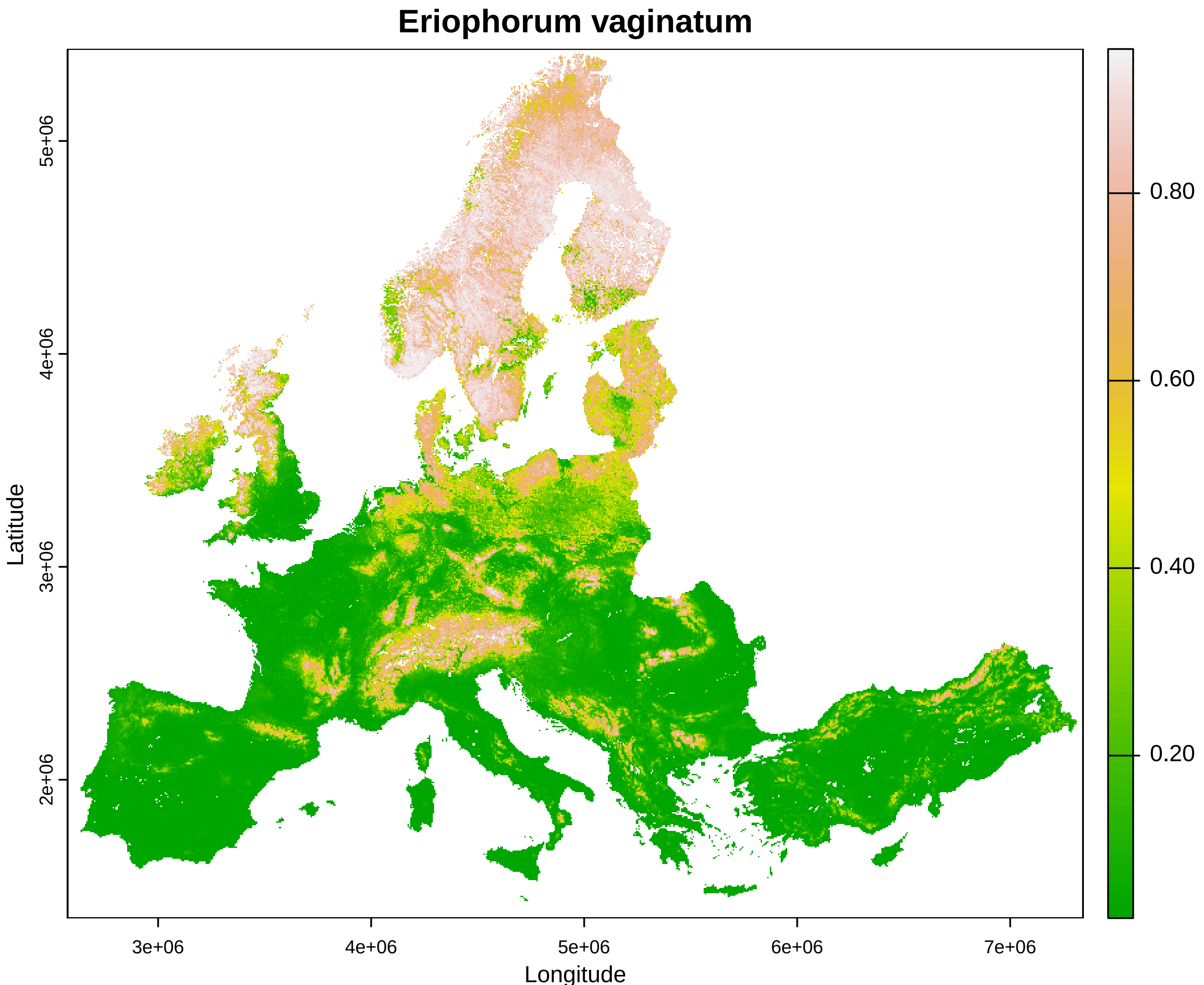

Eriophorum vaginatum
Latitude
Longitude
5e+06
4e+06
3e+06
2e+06
3e+06
4e+06
5e+06
6e+06
7e+06
0.80
0.60
0.40
0.20

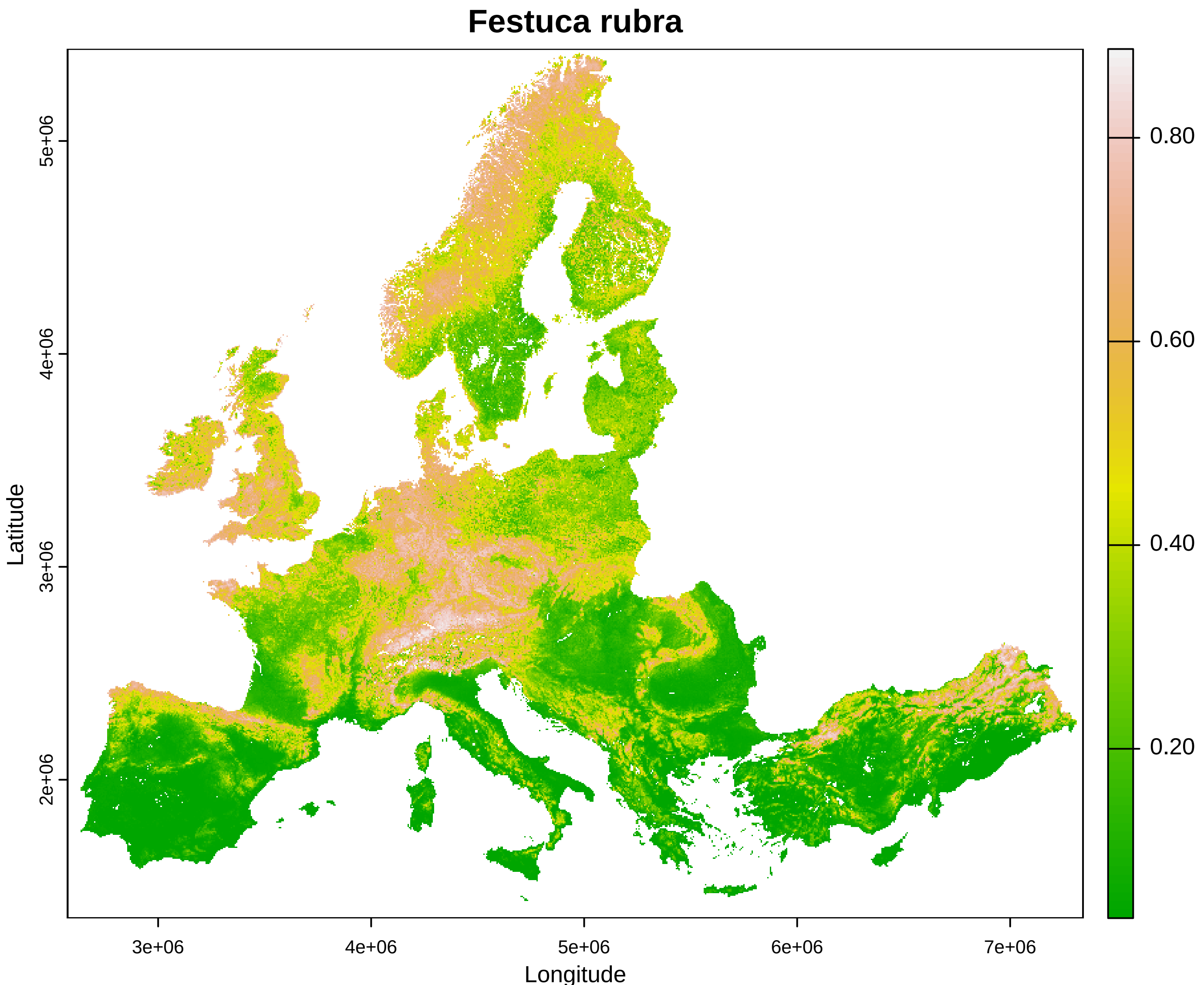

Festuca rubra
Latitude
5e+06
4e+06
3e+06
2e+06
Longitude
3e+06
4e+06
5e+06
6e+06
7e+06
0.80
0.60
0.40
0.20

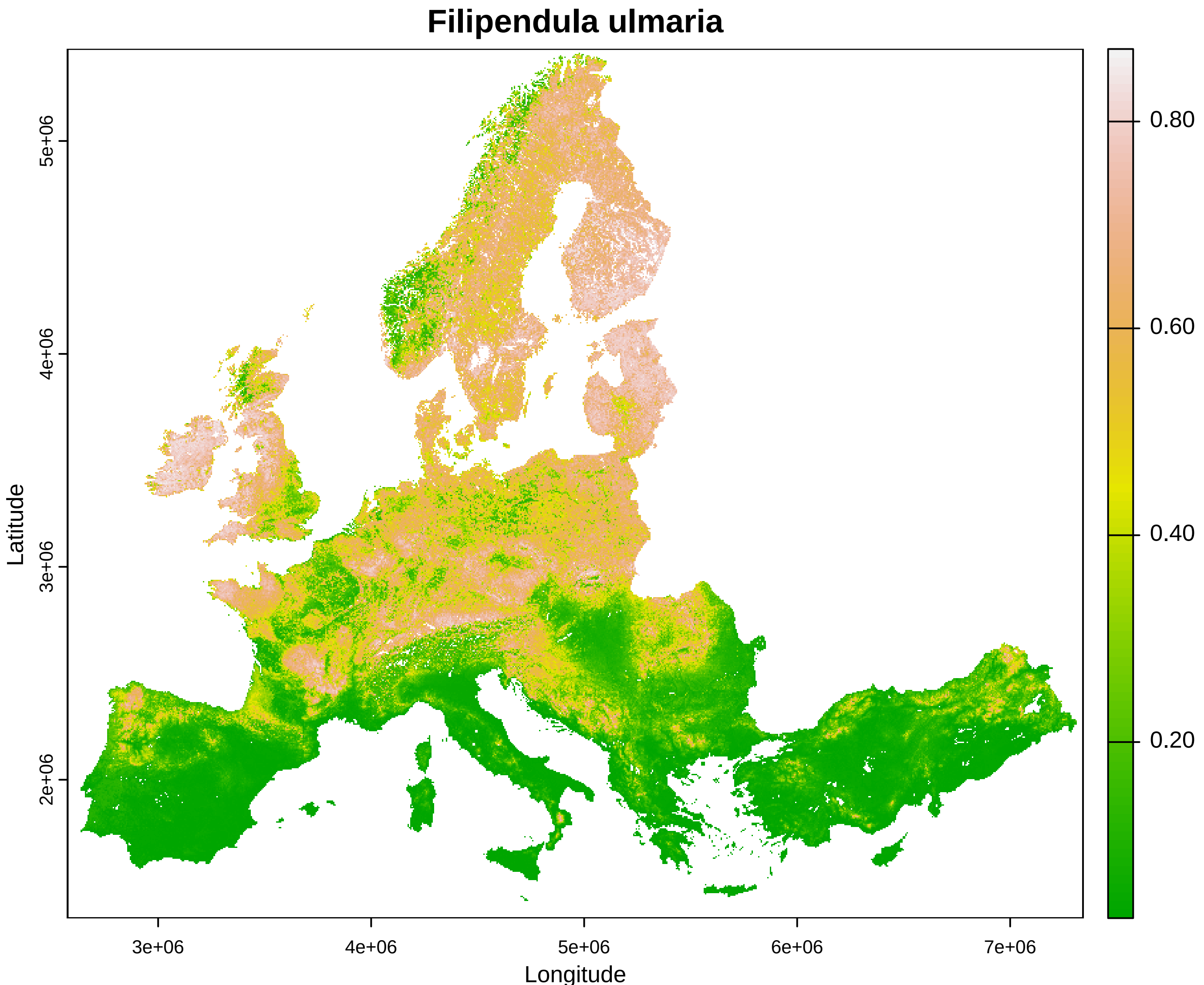
Filipendula ulmaria
Latitude
5e+06
4e+06
3e+06
2e+06
Longitude
3e+06
4e+06
5e+06
6e+06
7e+06
0.80
0.60
0.40
0.20

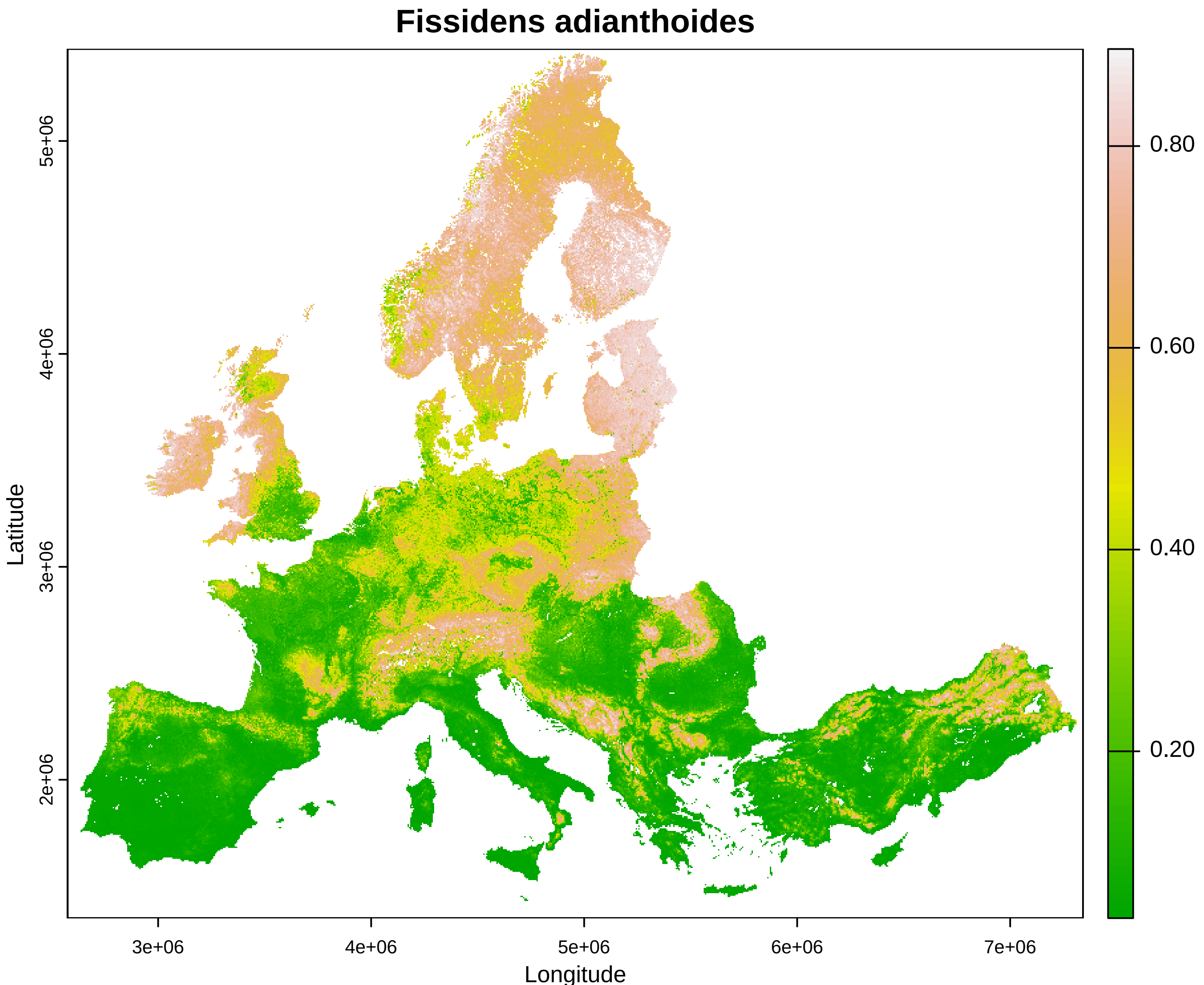

Fissidens adianthoides
Latitude
5e+06
4e+06
3e+06
2e+06
3e+06
4e+06
5e+06
6e+06
7e+06
Longitude
0.80
0.60
0.40
0.20

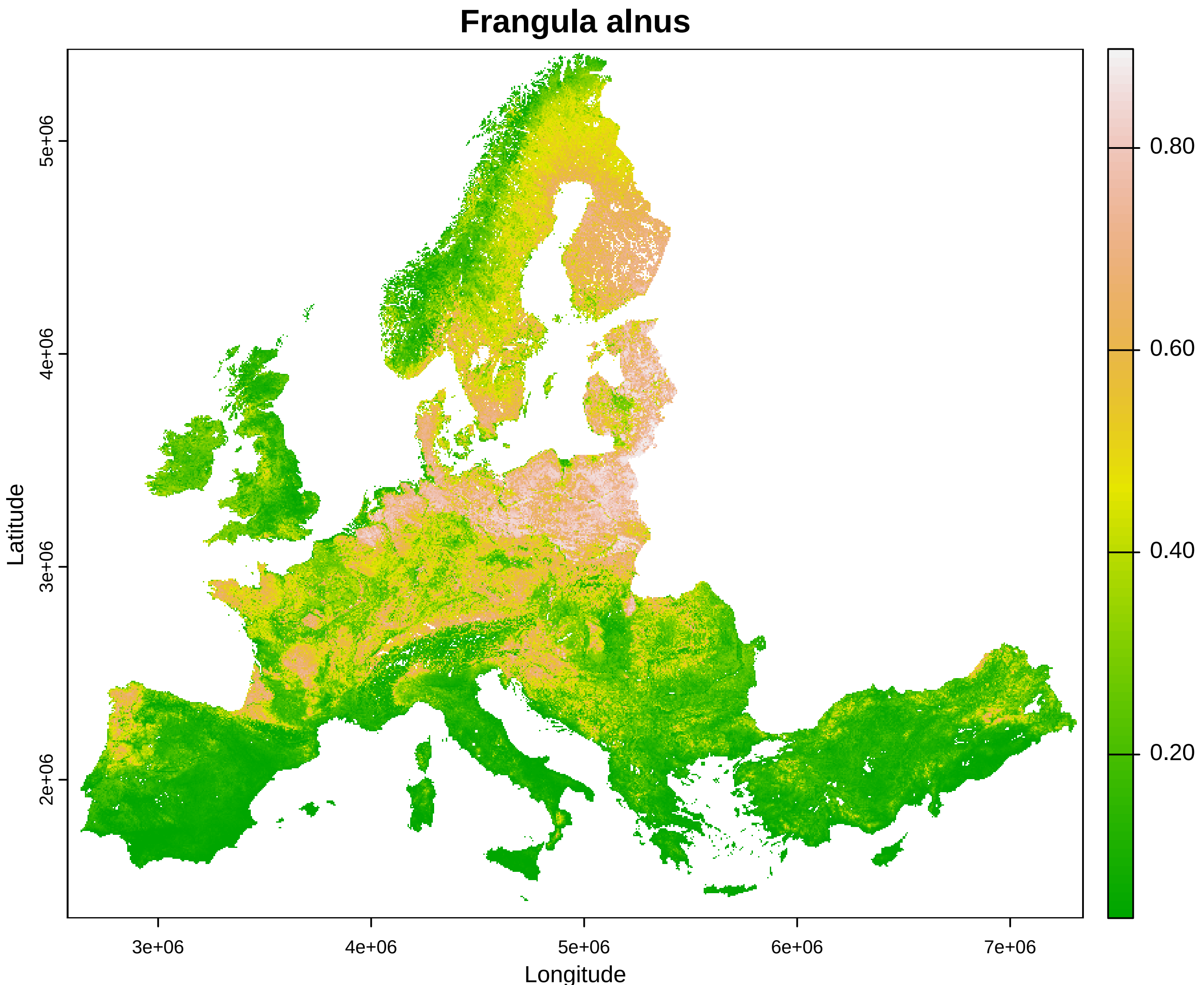
Frangula alnus
Latitude
Longitude
5e+06
4e+06
3e+06
2e+06
3e+06
4e+06
5e+06
6e+06
7e+06
0.80
0.60
0.40
0.20

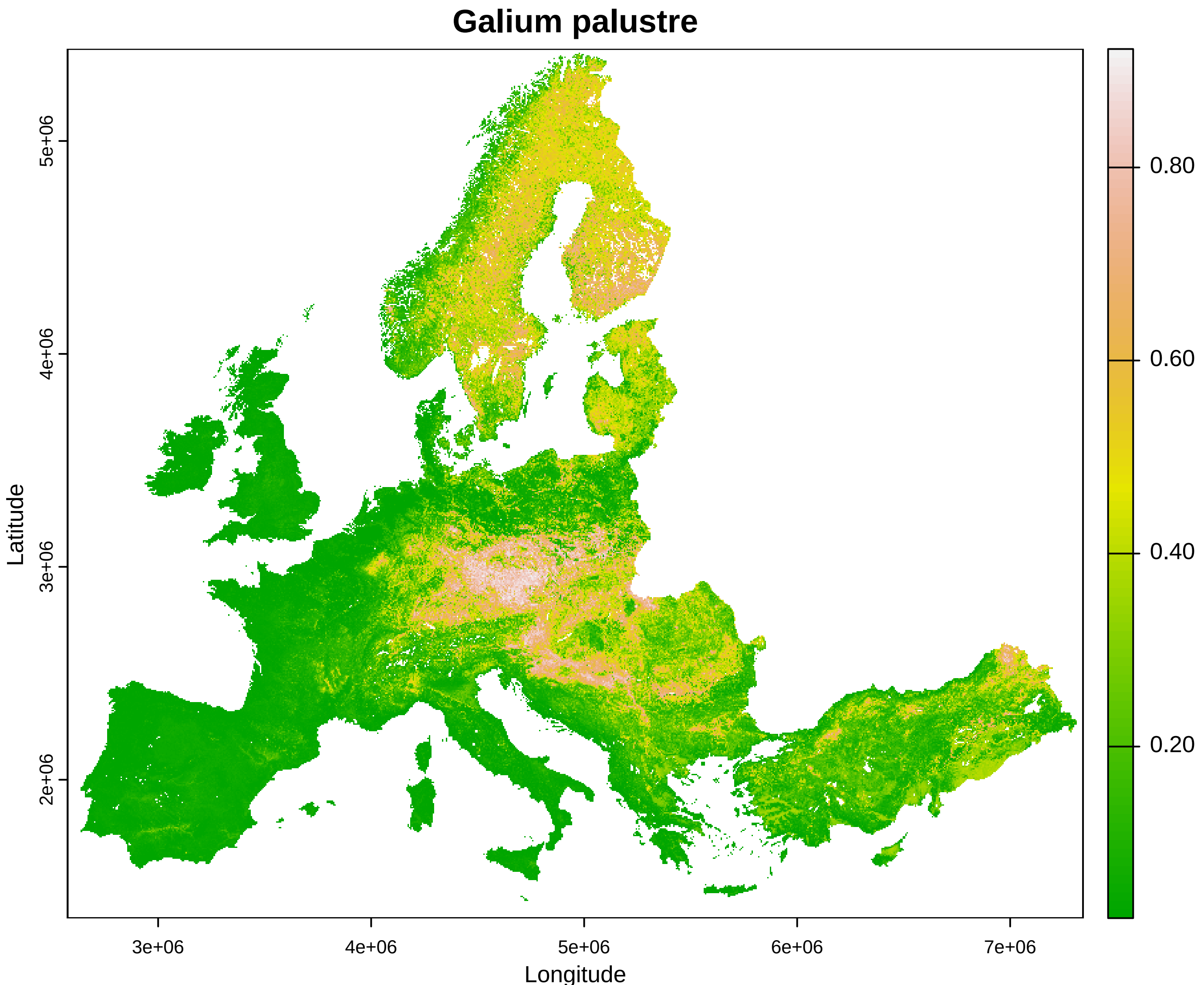
Galium palustre
Latitude
5e+06
4e+06
3e+06
2e+06
3e+06
4e+06
5e+06
6e+06
7e+06
Longitude
0.80
0.60
0.40
0.20

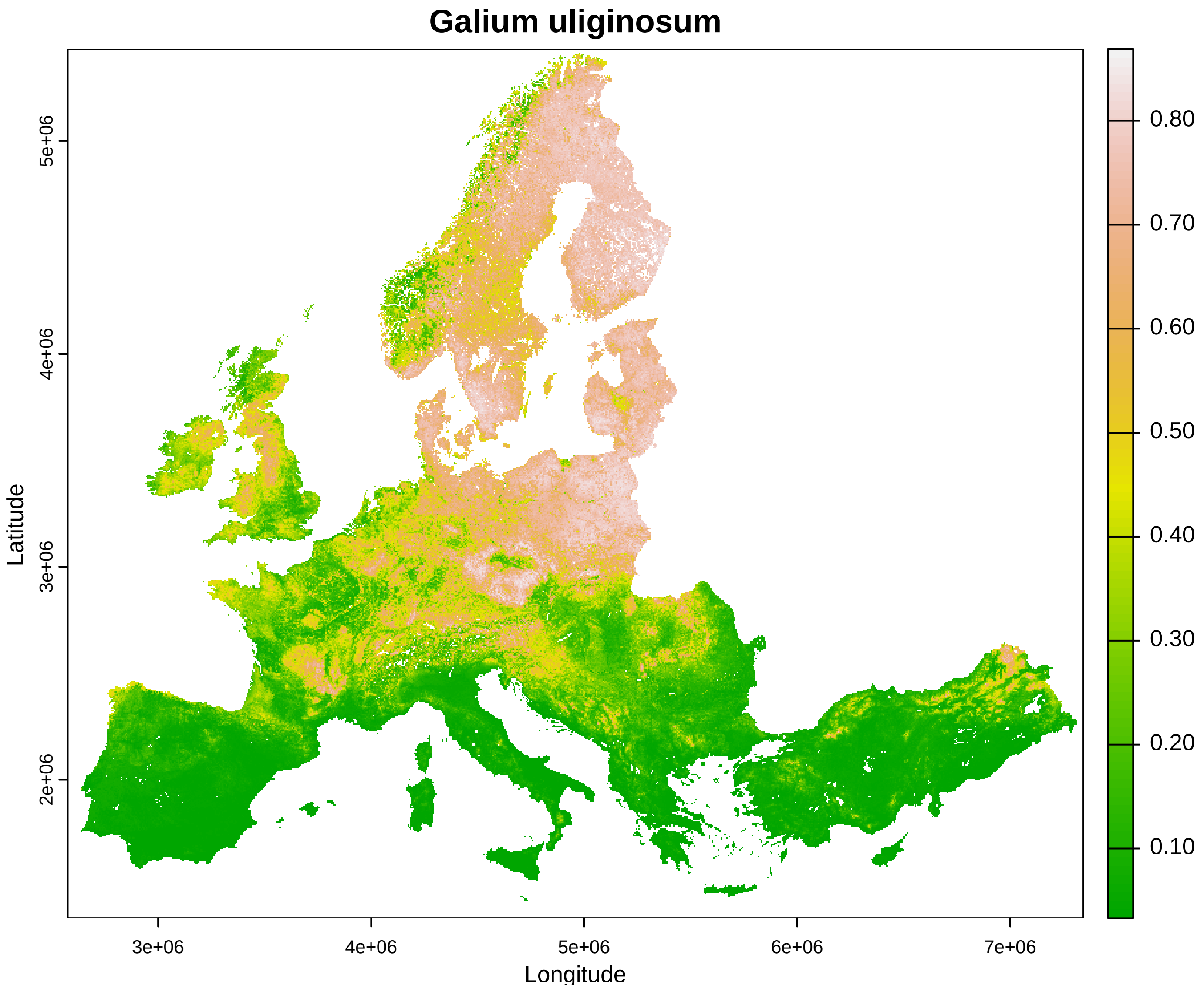
Galium uliginosum
Latitude
5e+06
4e+06
3e+06
2e+06
3e+06
4e+06
5e+06
6e+06
7e+06
Longitude
0.80
0.70
0.60
0.50
0.40
0.30
0.20
0.10

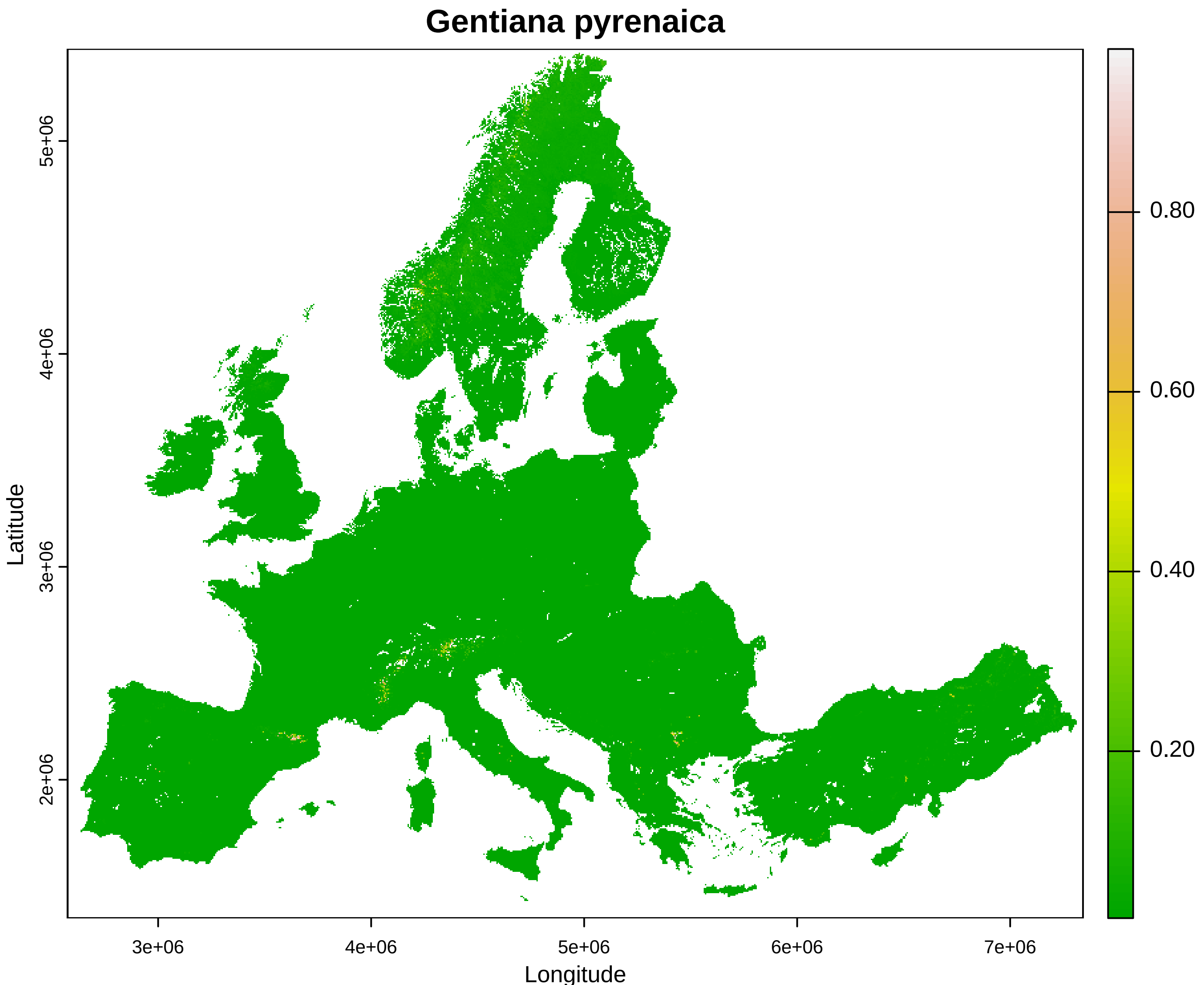
Gentiana pyrenaica
Latitude
Longitude
5e+06
4e+06
3e+06
2e+06
3e+06
4e+06
5e+06
6e+06
7e+06
0.80
0.60
0.40
0.20

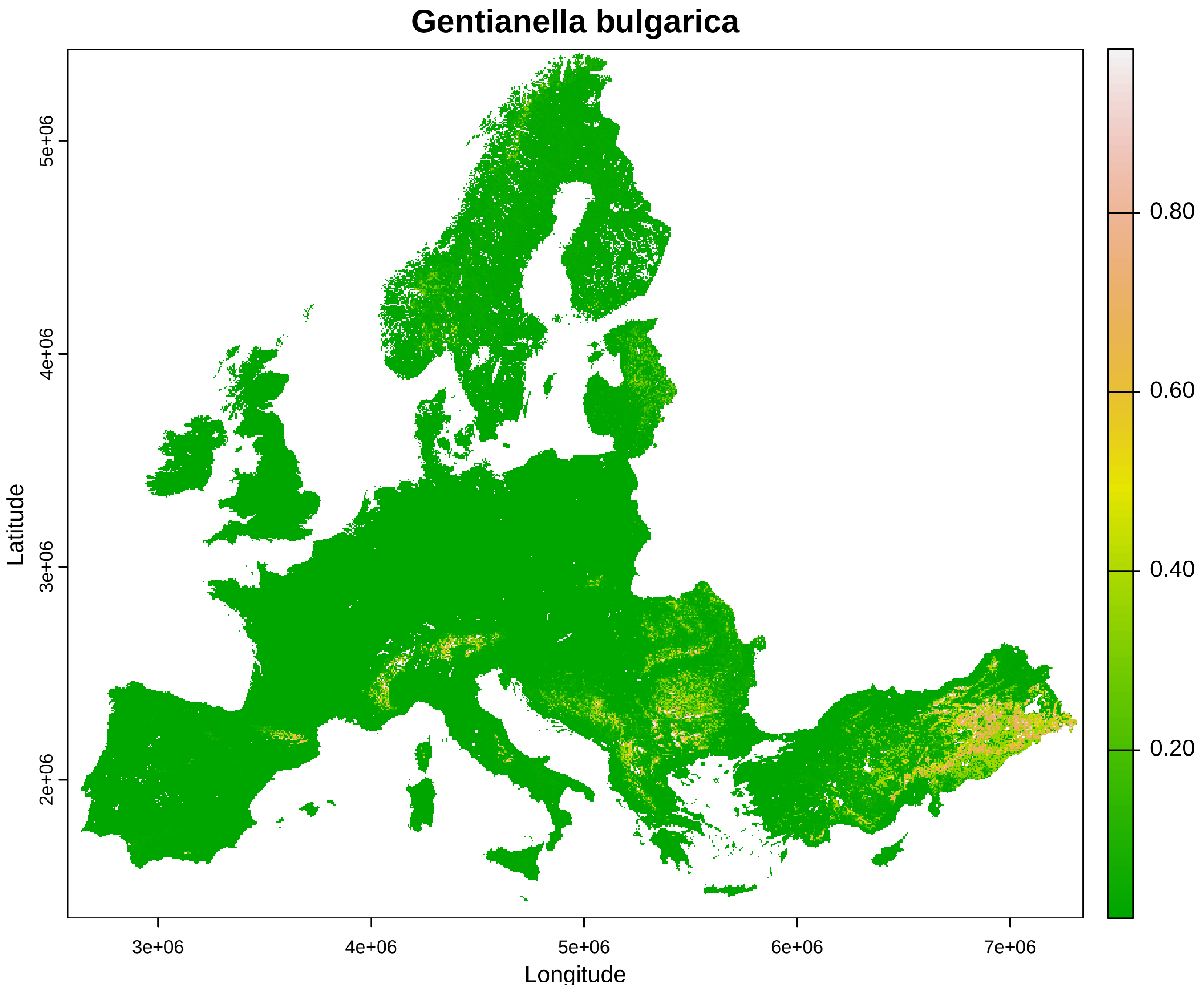
Gentianella bulgarica
Latitude
Longitude
5e+06
4e+06
3e+06
2e+06
3e+06
4e+06
5e+06
6e+06
7e+06
0.80
0.60
0.40
0.20

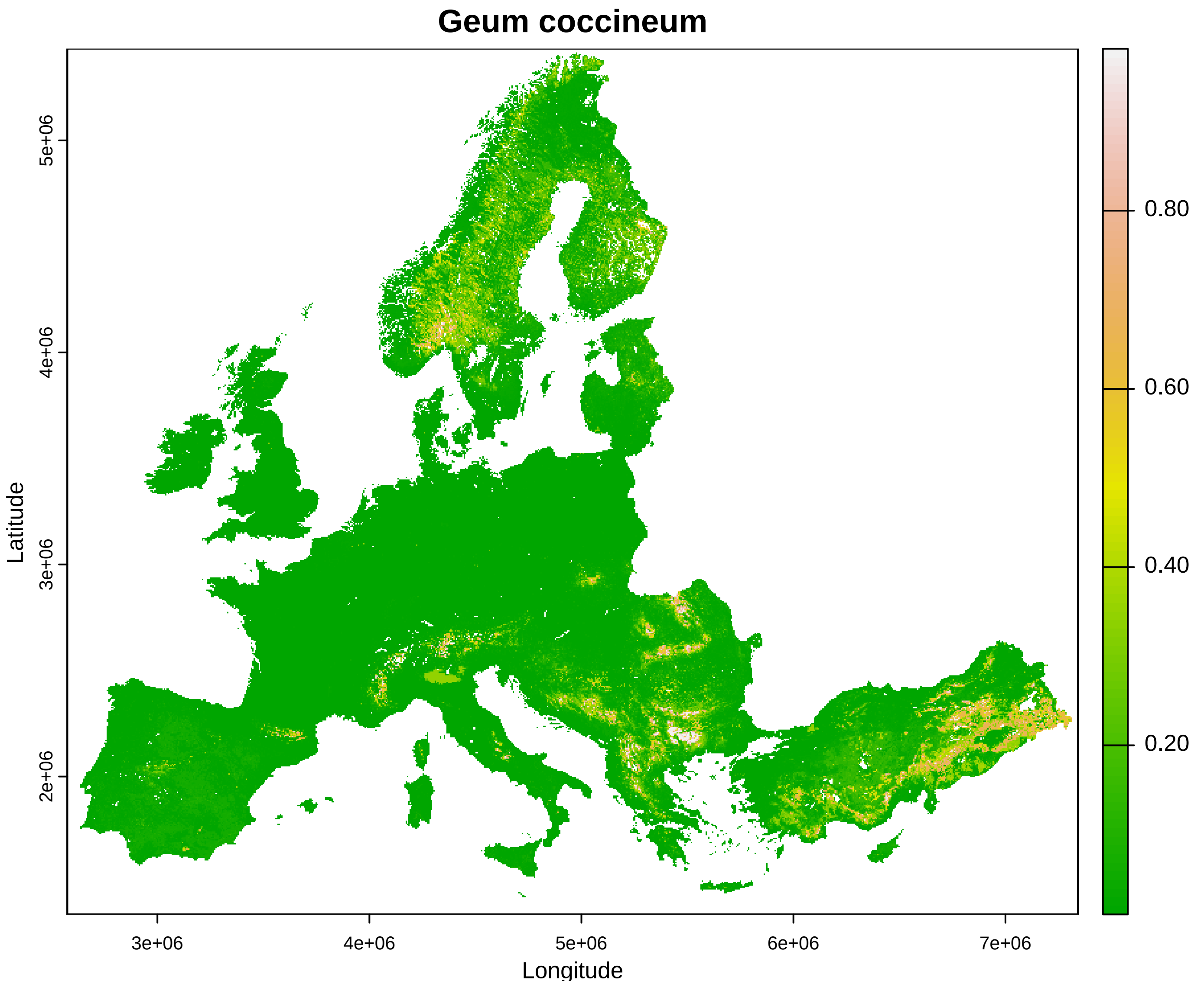
Geum coccineum
Latitude
Longitude
5e+06
4e+06
3e+06
2e+06
3e+06
4e+06
5e+06
6e+06
7e+06
0.80
0.60
0.40
0.20

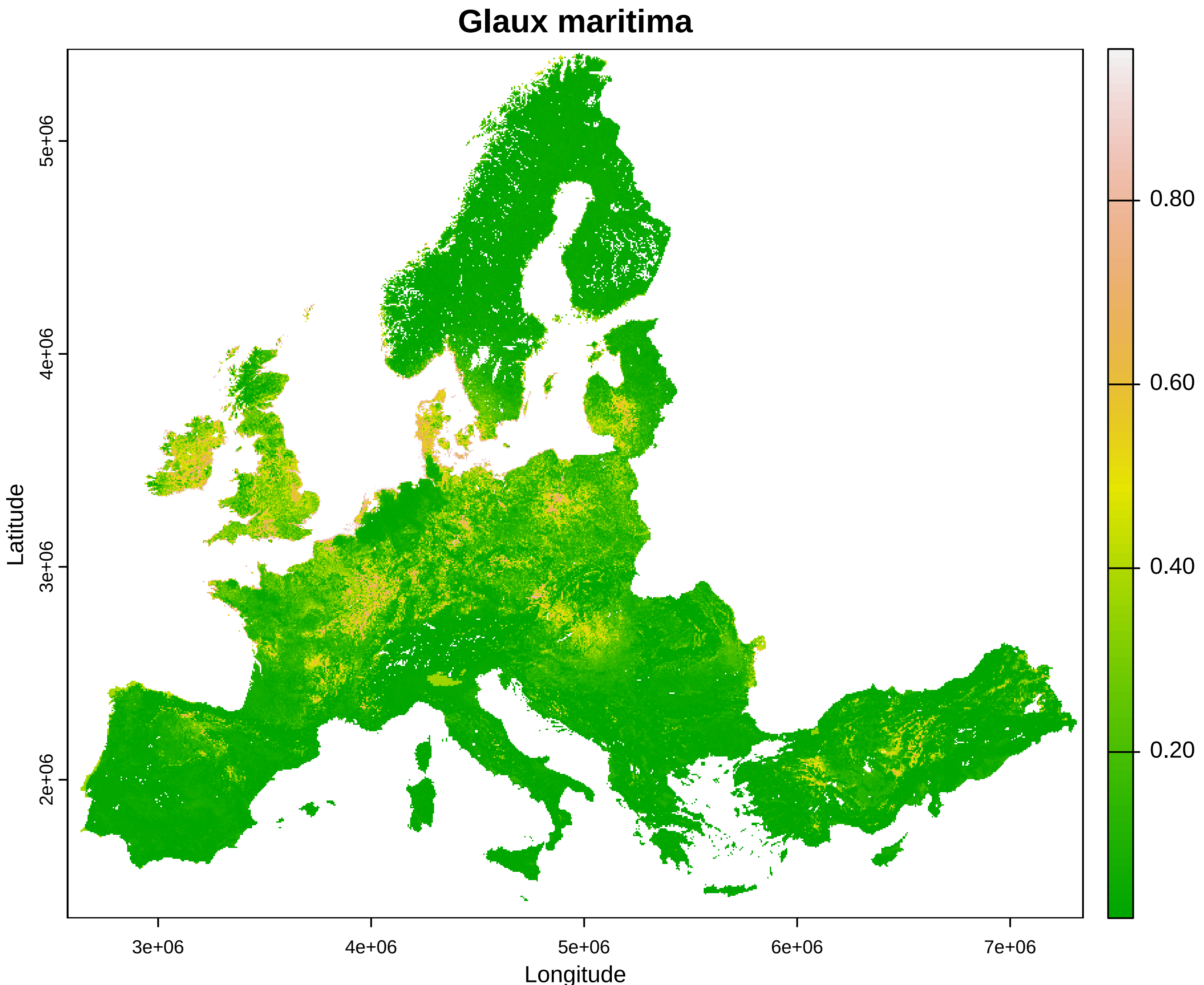

Glaux maritima
Latitude
Longitude
5e+06
4e+06
3e+06
2e+06
3e+06
4e+06
5e+06
6e+06
7e+06
0.80
0.60
0.40
0.20

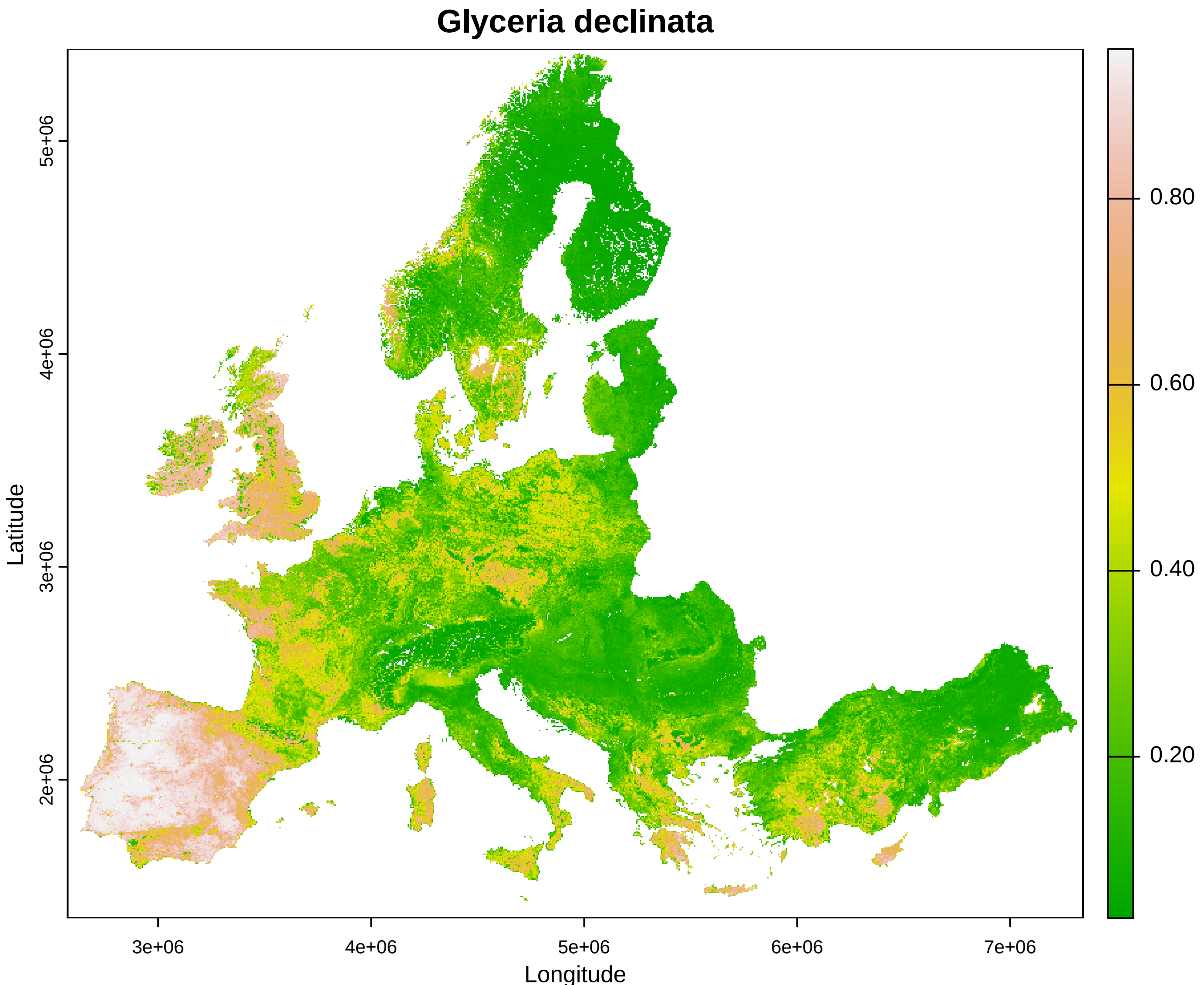
Glyceria declinata
Latitude
5e+06
4e+06
3e+06
2e+06
Longitude
3e+06
4e+06
5e+06
6e+06
7e+06
0.80
0.60
0.40
0.20

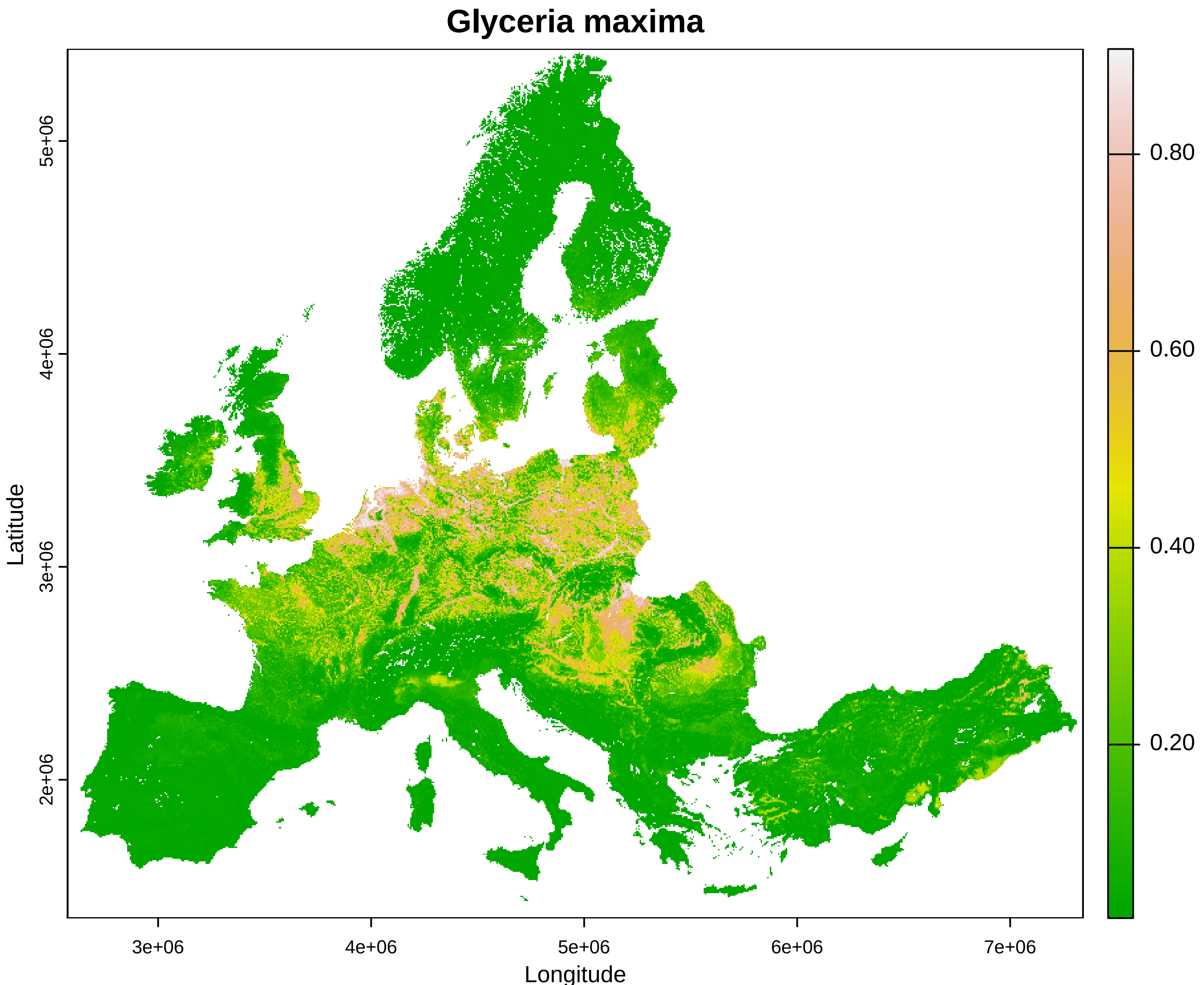

Glyceria maxima
Latitude
Longitude
5e+06
4e+06
3e+06
2e+06
3e+06
4e+06
5e+06
6e+06
7e+06
0.80
0.60
0.40
0.20

Gnaphalium uliginosum

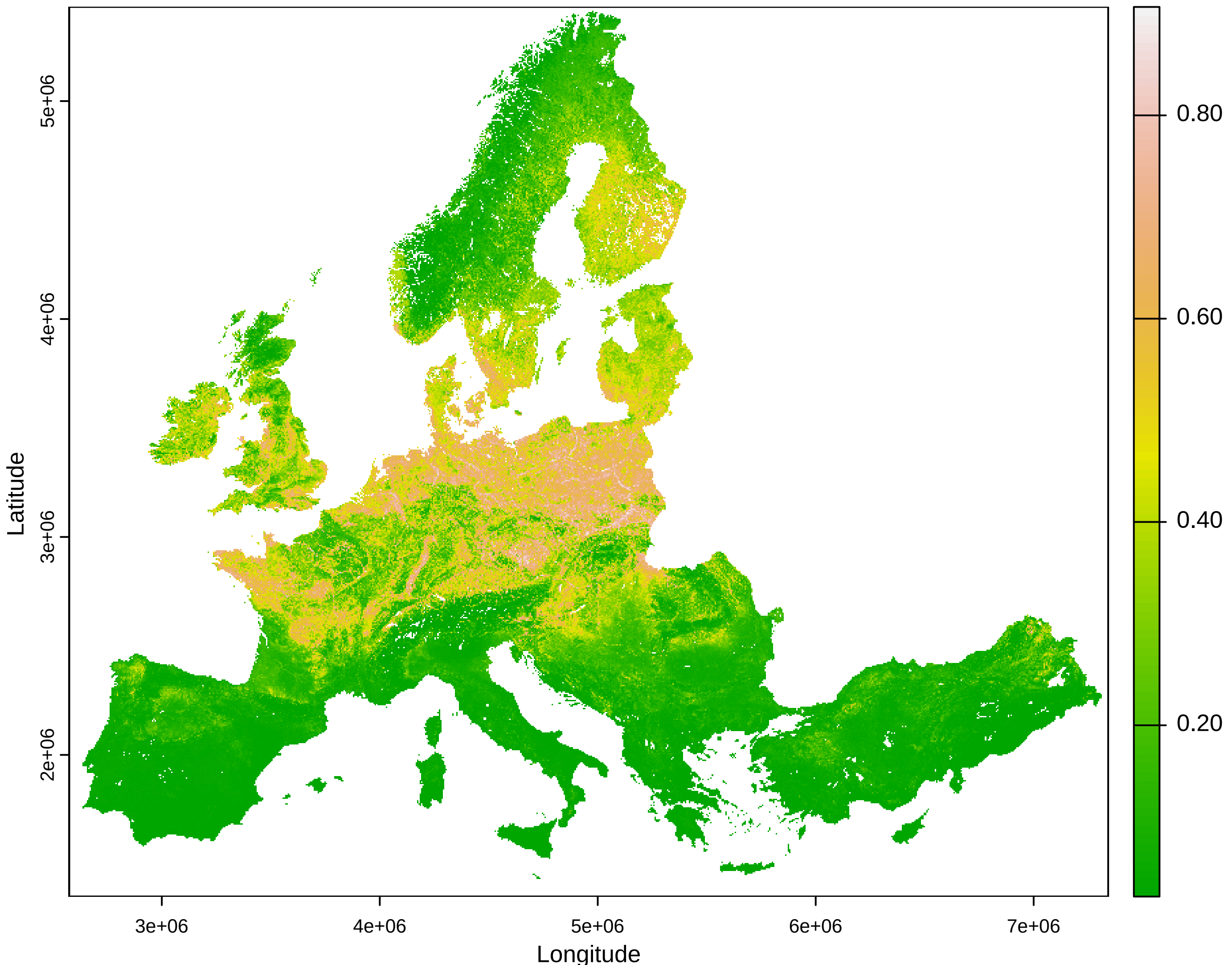

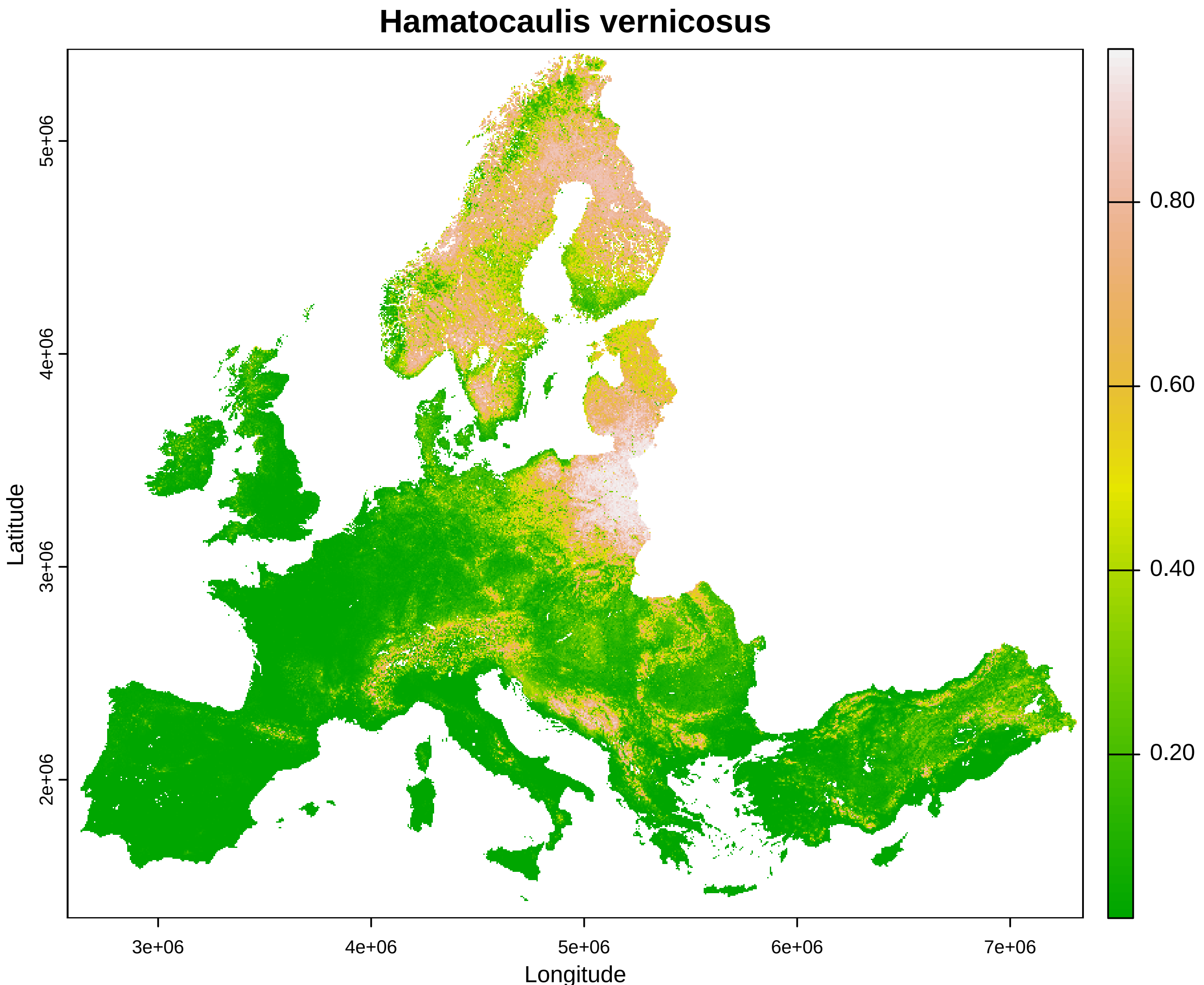
Hamatocaulis vernicosus
Latitude
5e+06
4e+06
3e+06
2e+06
3e+06
4e+06
5e+06
6e+06
7e+06
Longitude
0.80
0.60
0.40
0.20

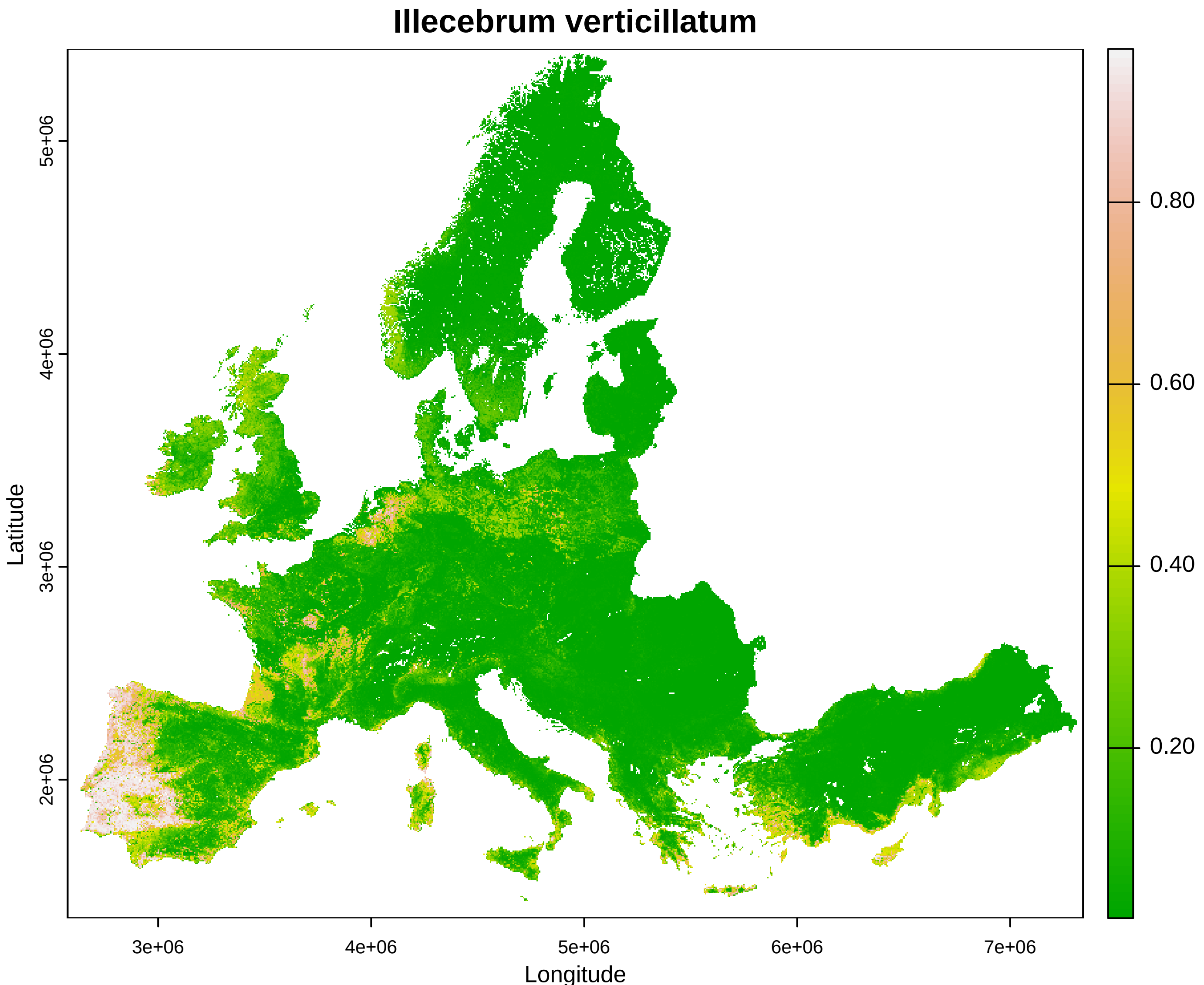
Illecebrum verticillatum
Latitude
Longitude
5e+06
4e+06
3e+06
2e+06
3e+06
4e+06
5e+06
6e+06
7e+06
0.80
0.60
0.40
0.20

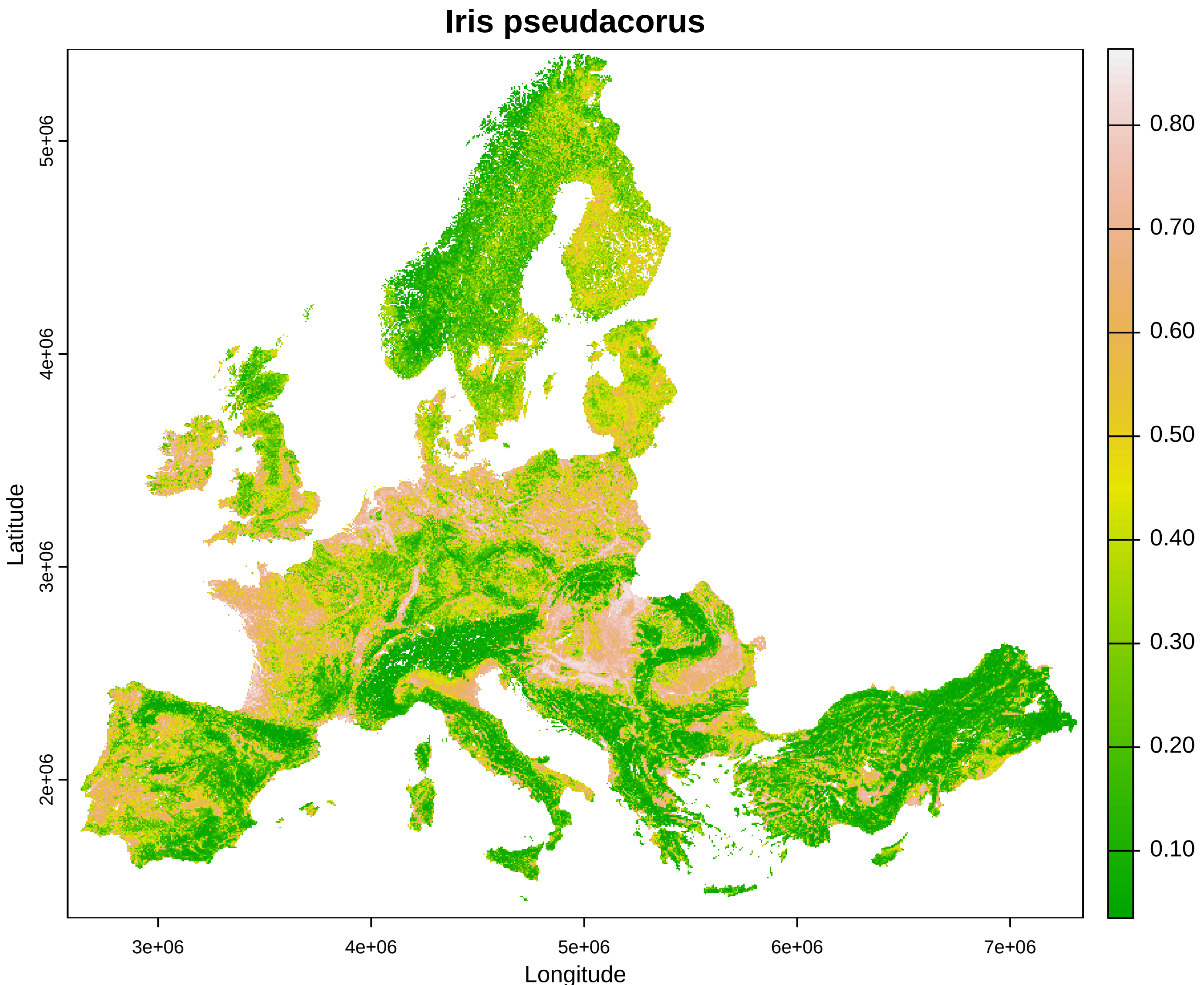
Iris pseudacorus
Latitude
5e+06
4e+06
3e+06
2e+06
Longitude
3e+06
4e+06
5e+06
6e+06
7e+06
0.80
0.70
0.60
0.50
0.40
0.30
0.20
0.10

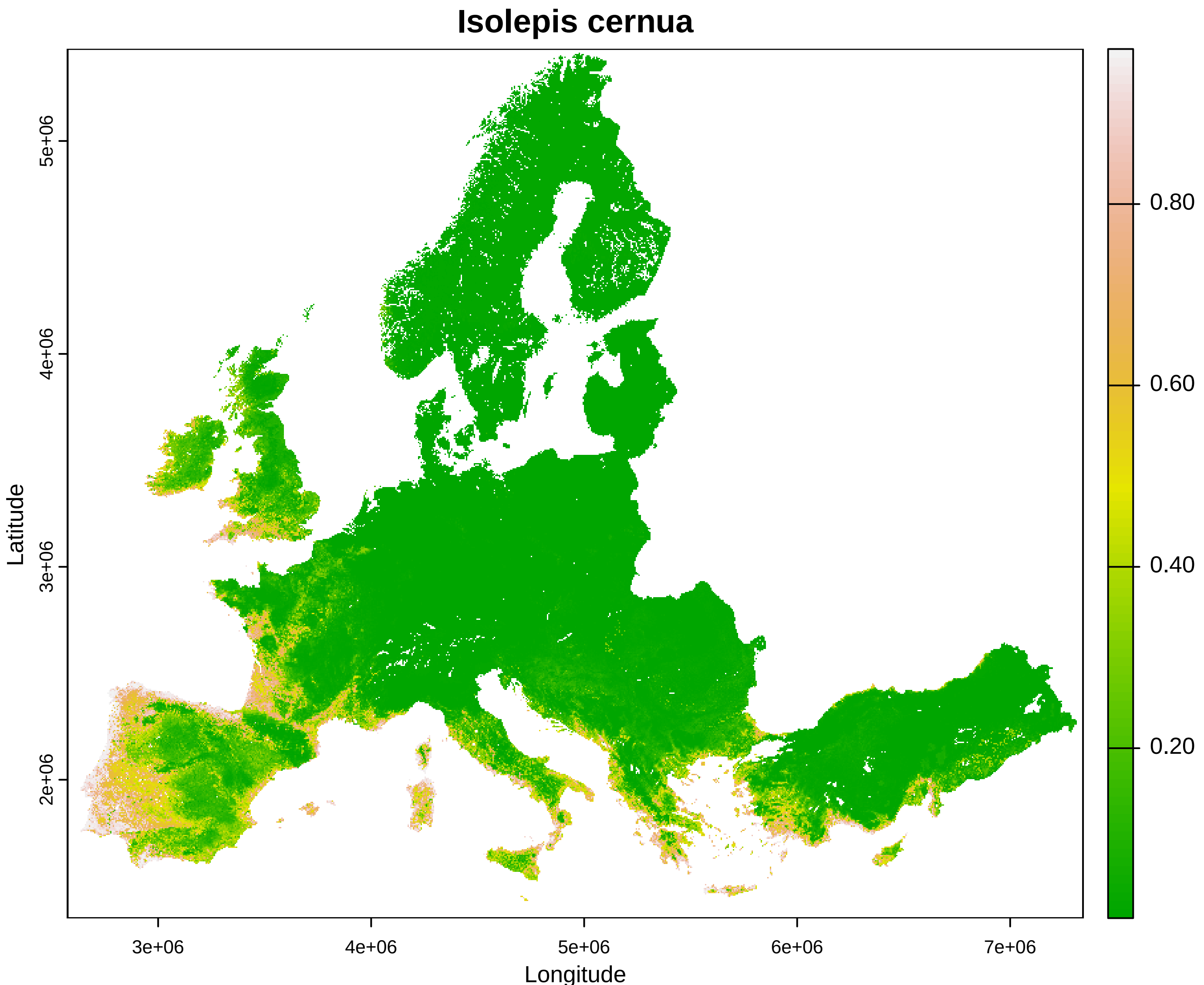
Isolepis cernua
Latitude
5e+06
4e+06
3e+06
2e+06
Longitude
3e+06
4e+06
5e+06
6e+06
7e+06
0.80
0.60
0.40
0.20

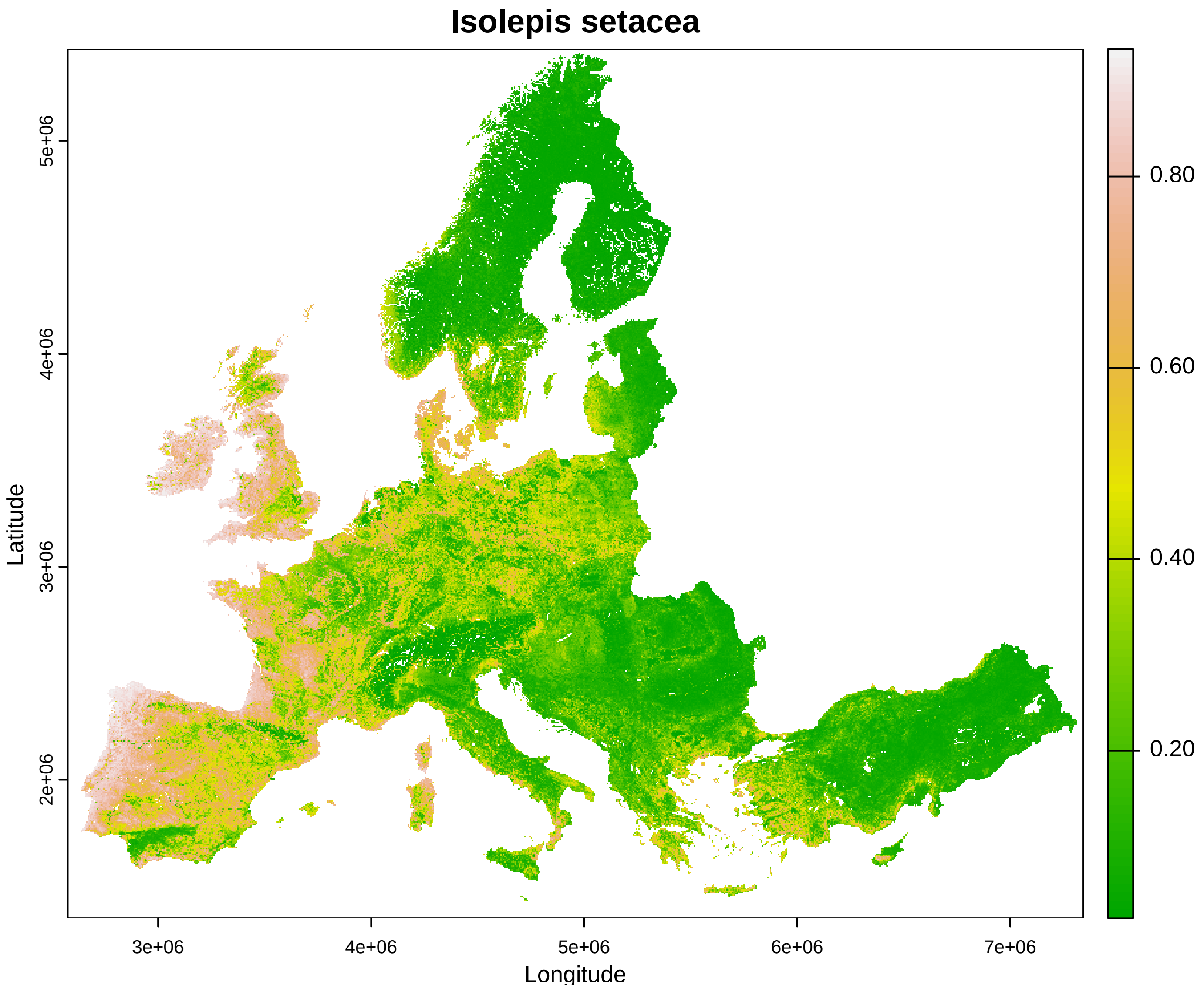

Isolepis setacea
Latitude
Longitude
5e+06
4e+06
3e+06
2e+06
3e+06
4e+06
5e+06
6e+06
7e+06
0.80
0.60
0.40
0.20

Juncus alpinoarticulatus

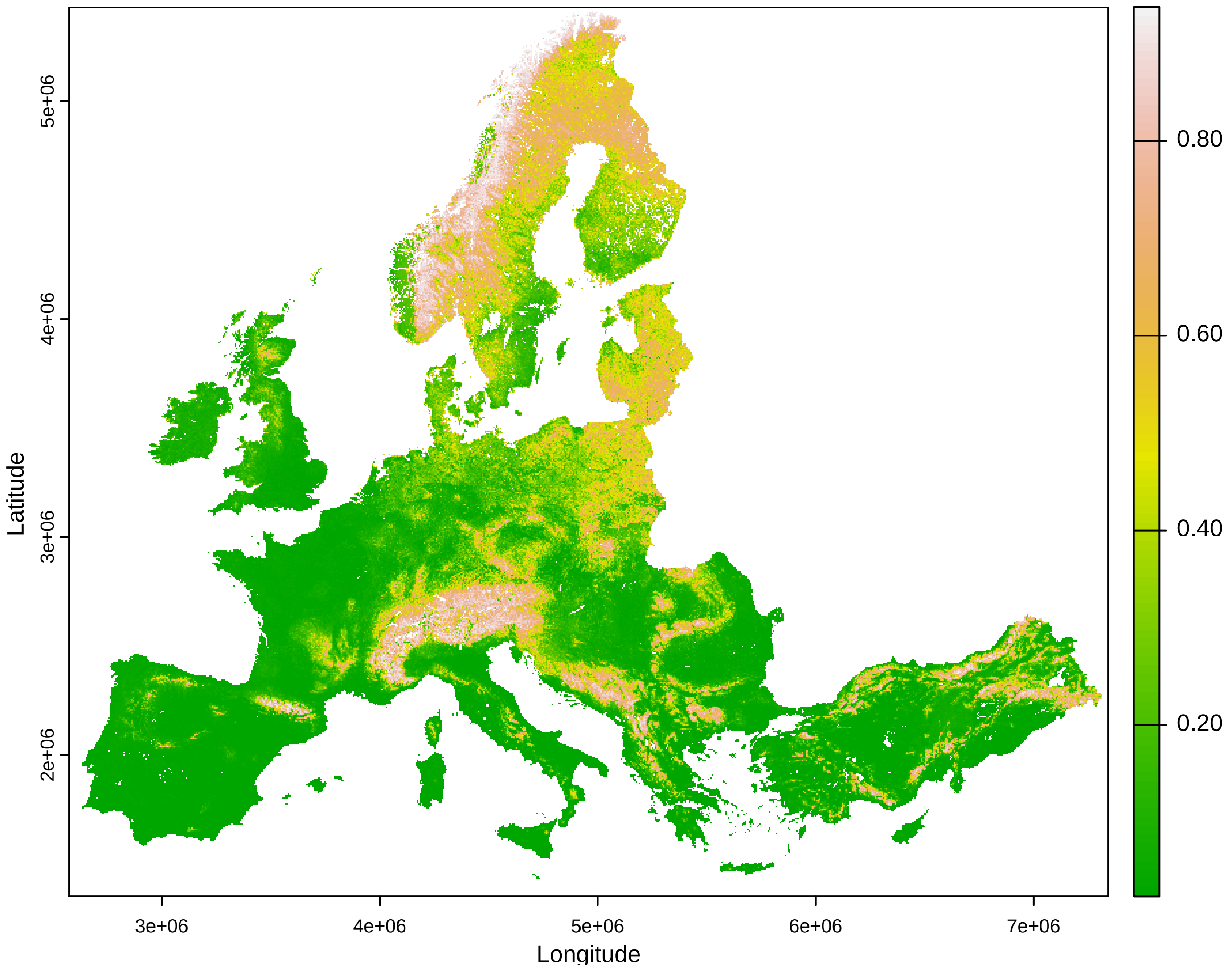

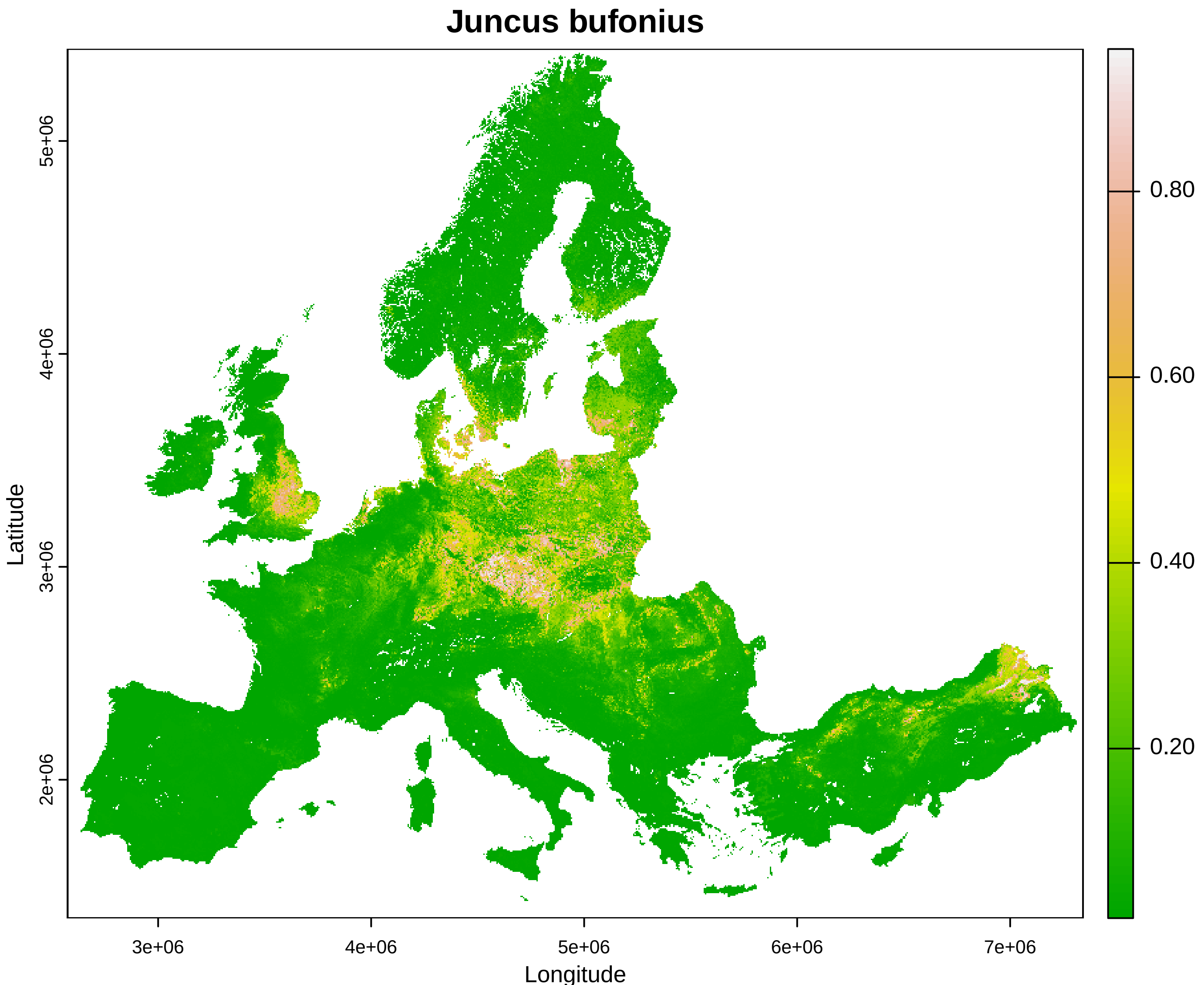
Juncus bufonius
Latitude
Longitude
5e+06
4e+06
3e+06
2e+06
3e+06
4e+06
5e+06
6e+06
7e+06
0.80
0.60
0.40
0.20

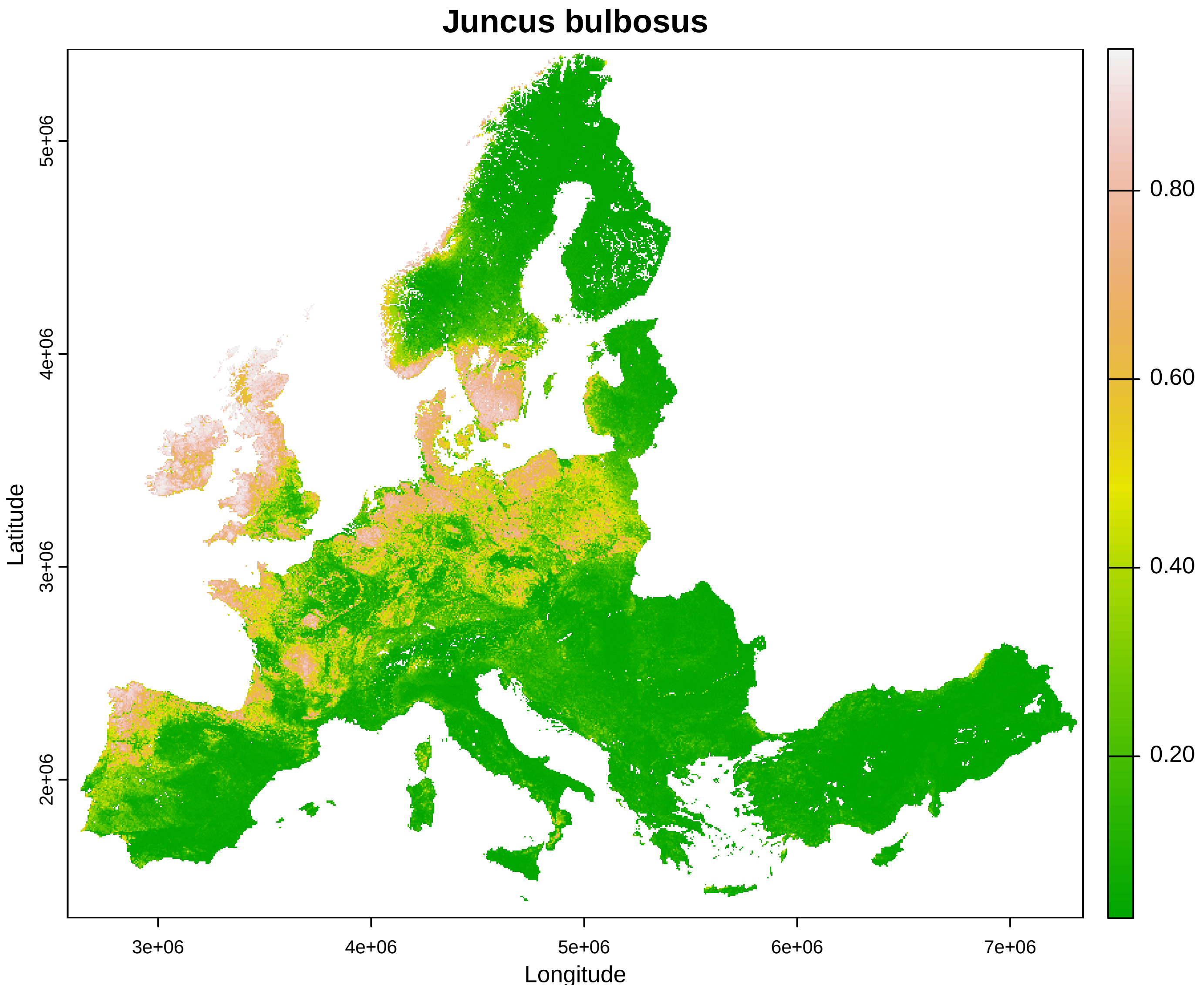
Juncus bulbosus
Latitude
5e+06
4e+06
3e+06
2e+06
Longitude
3e+06
4e+06
5e+06
6e+06
7e+06
0.80
0.60
0.40
0.20

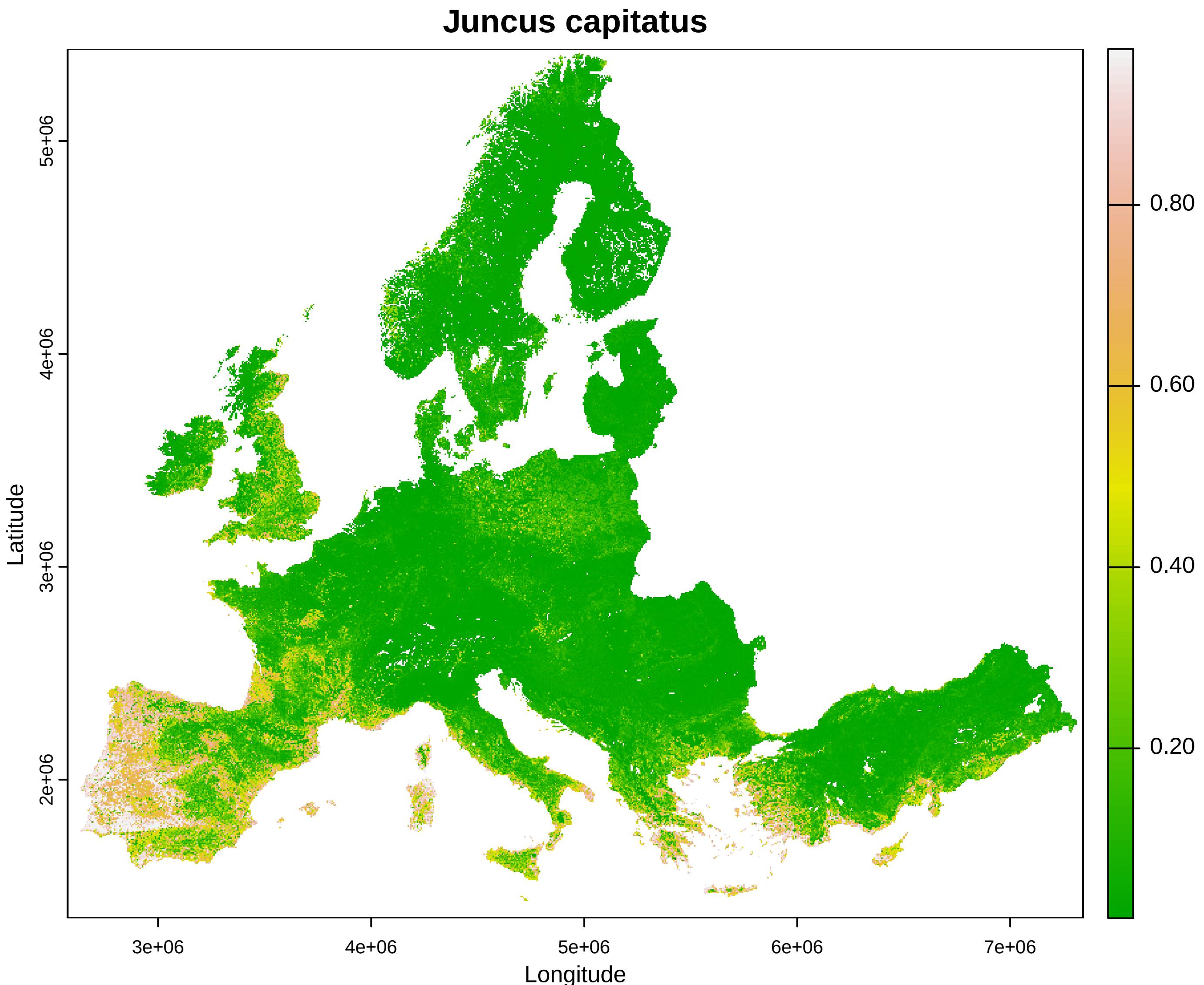
Juncus capitatus
Latitude
Longitude
5e+06
4e+06
3e+06
2e+06
3e+06
4e+06
5e+06
6e+06
7e+06
0.80
0.60
0.40
0.20

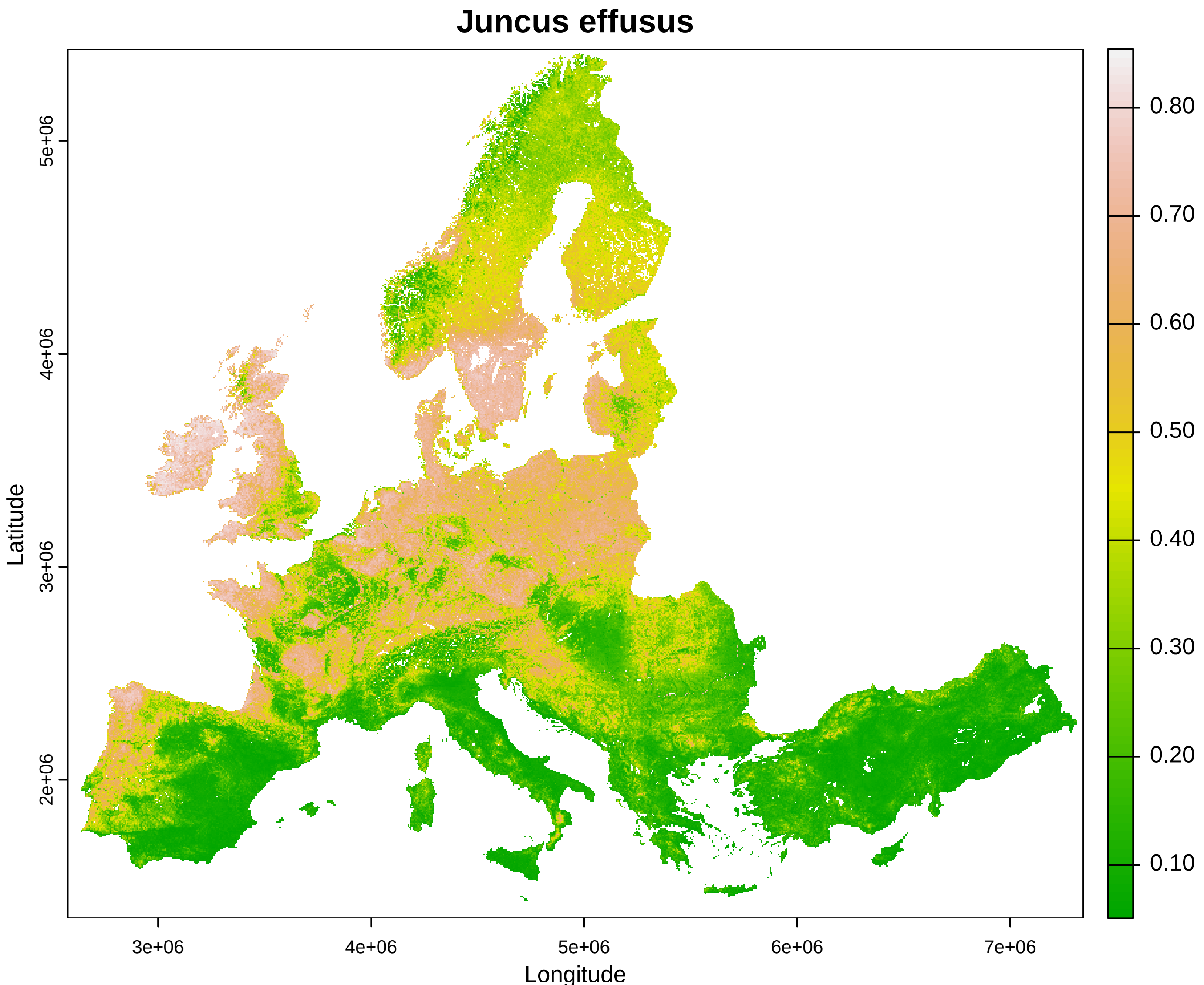
Juncus effusus
Latitude
Longitude
5e+06
4e+06
3e+06
2e+06
3e+06
4e+06
5e+06
6e+06
7e+06
0.80
0.70
0.60
0.50
0.40
0.30
0.20
0.10

Juncus filiformis

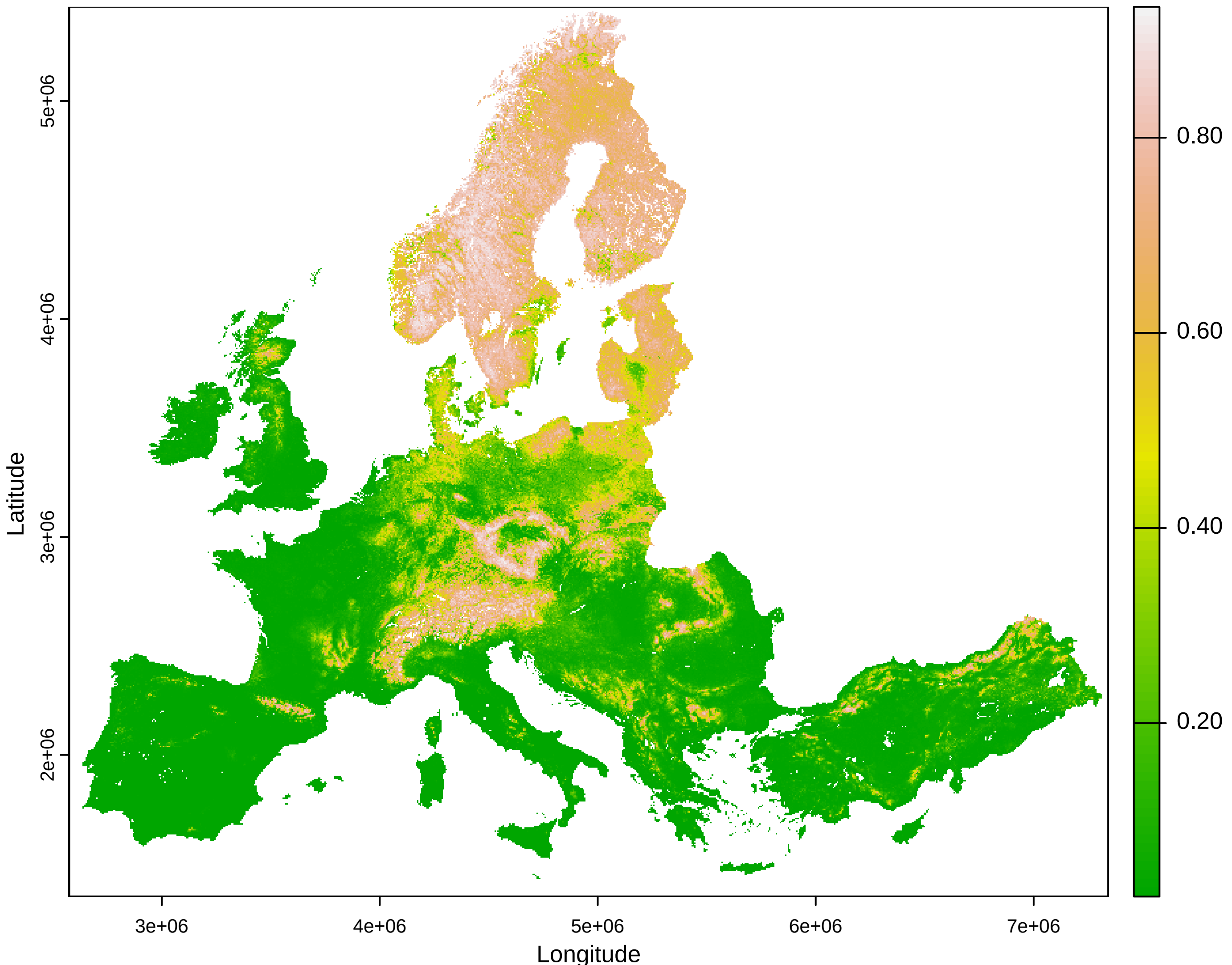

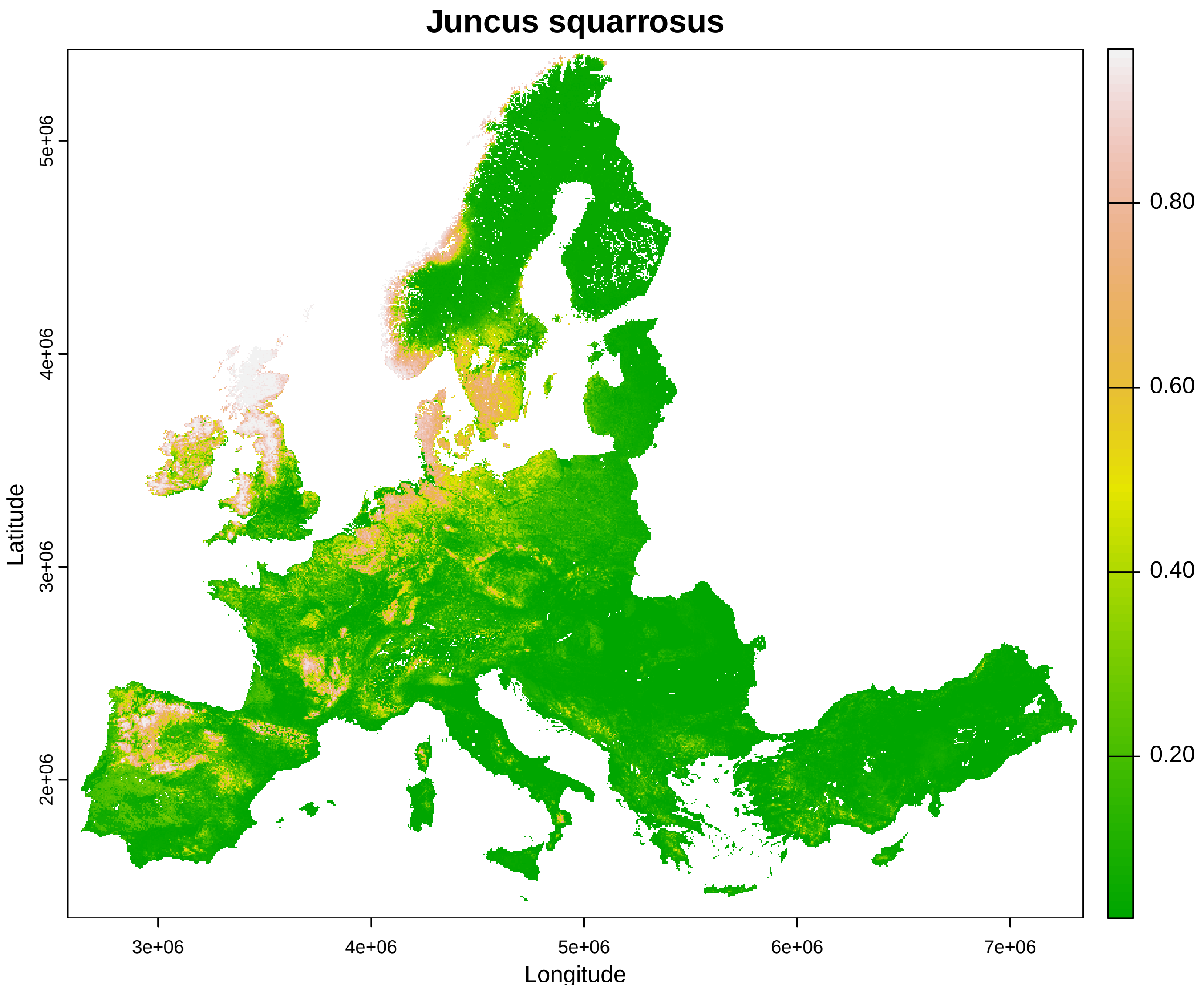

Juncus squarrosus
Latitude
5e+06
4e+06
3e+06
2e+06
3e+06
4e+06
5e+06
6e+06
7e+06
Longitude
0.80
0.60
0.40
0.20

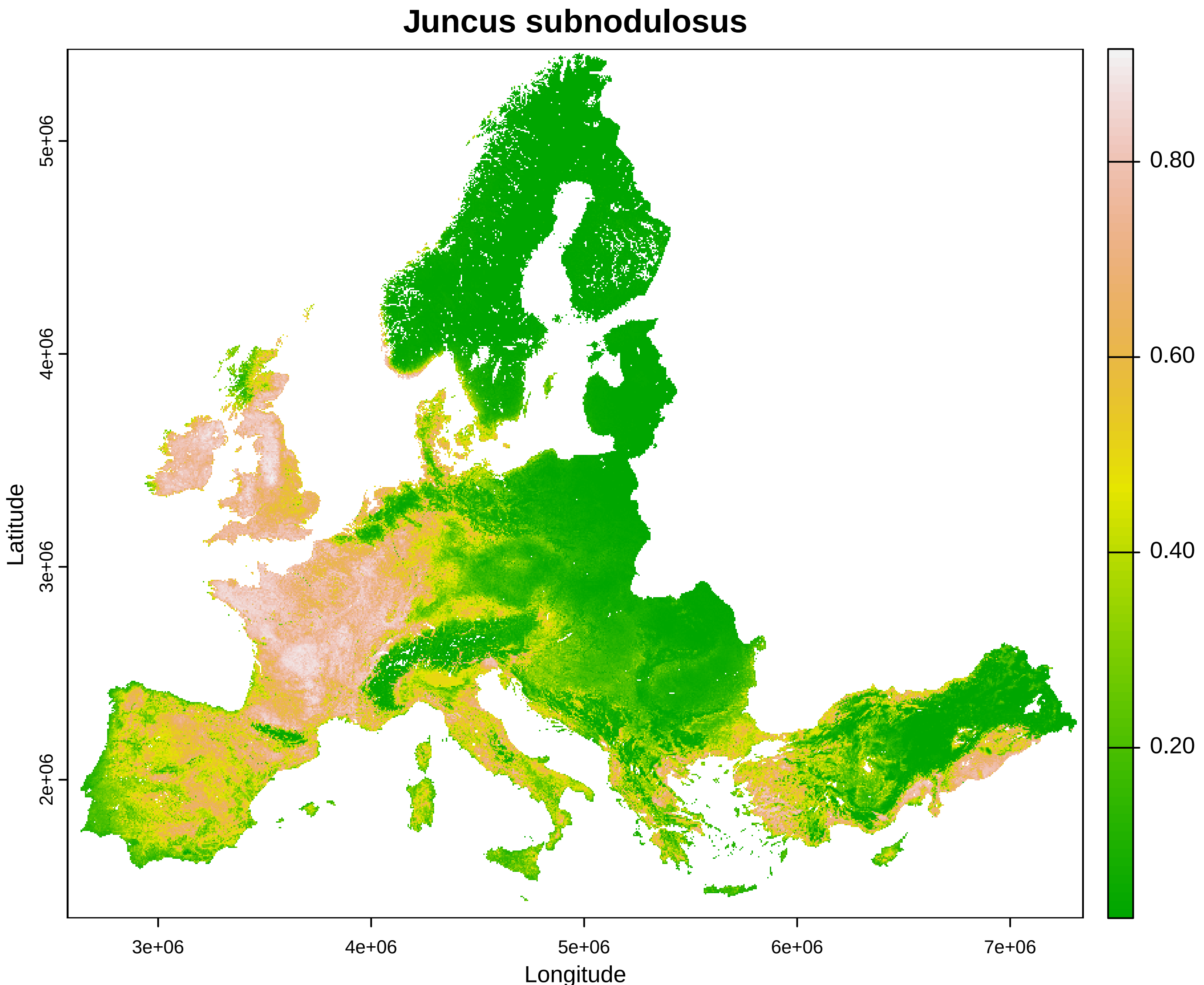

Juncus subnodulosus
Latitude
Longitude
5e+06
4e+06
3e+06
2e+06
3e+06
4e+06
5e+06
6e+06
7e+06
0.80
0.60
0.40
0.20

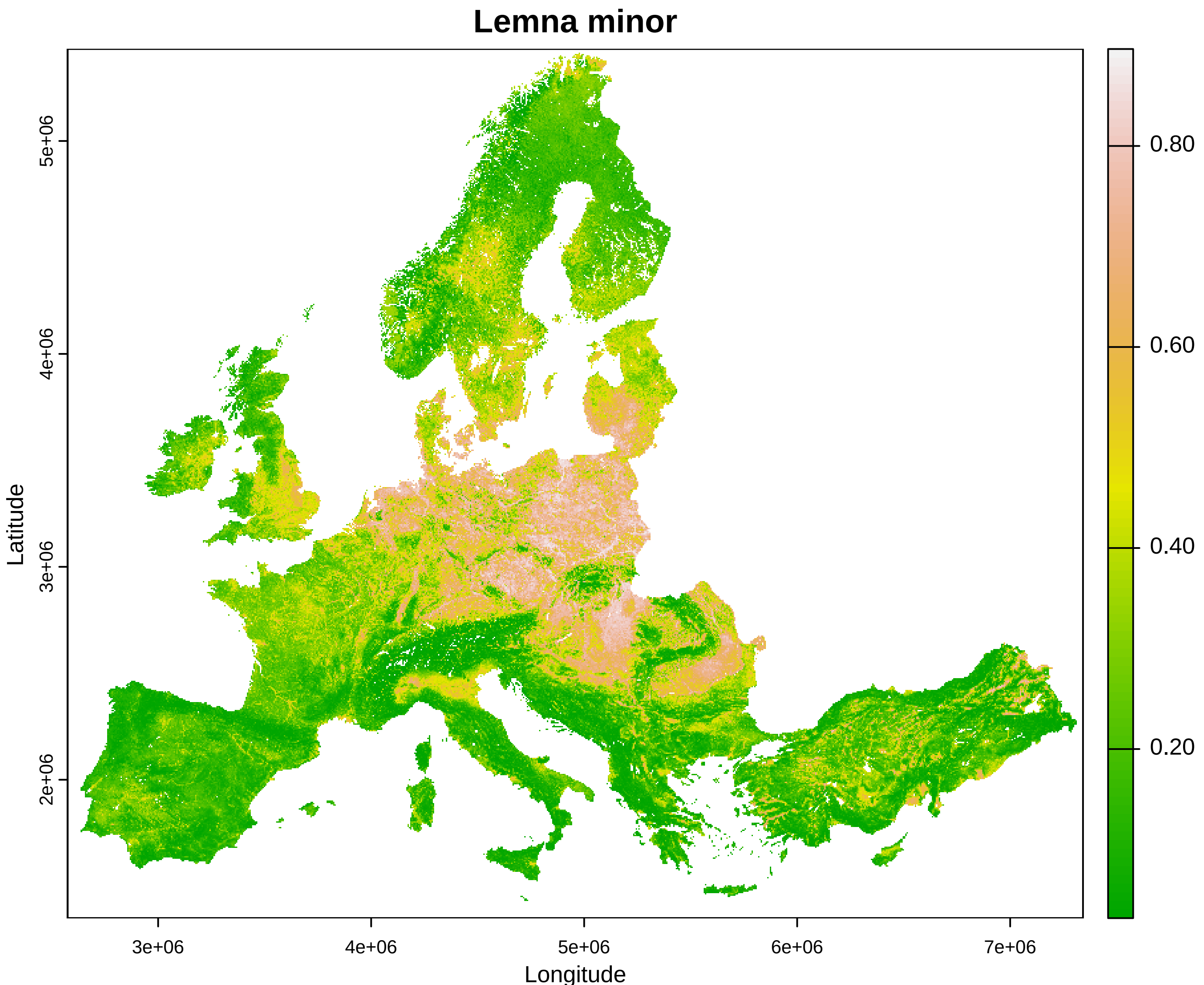

Lemna minor
Latitude
Longitude
5e+06
4e+06
3e+06
2e+06
3e+06
4e+06
5e+06
6e+06
7e+06
0.80
0.60
0.40
0.20

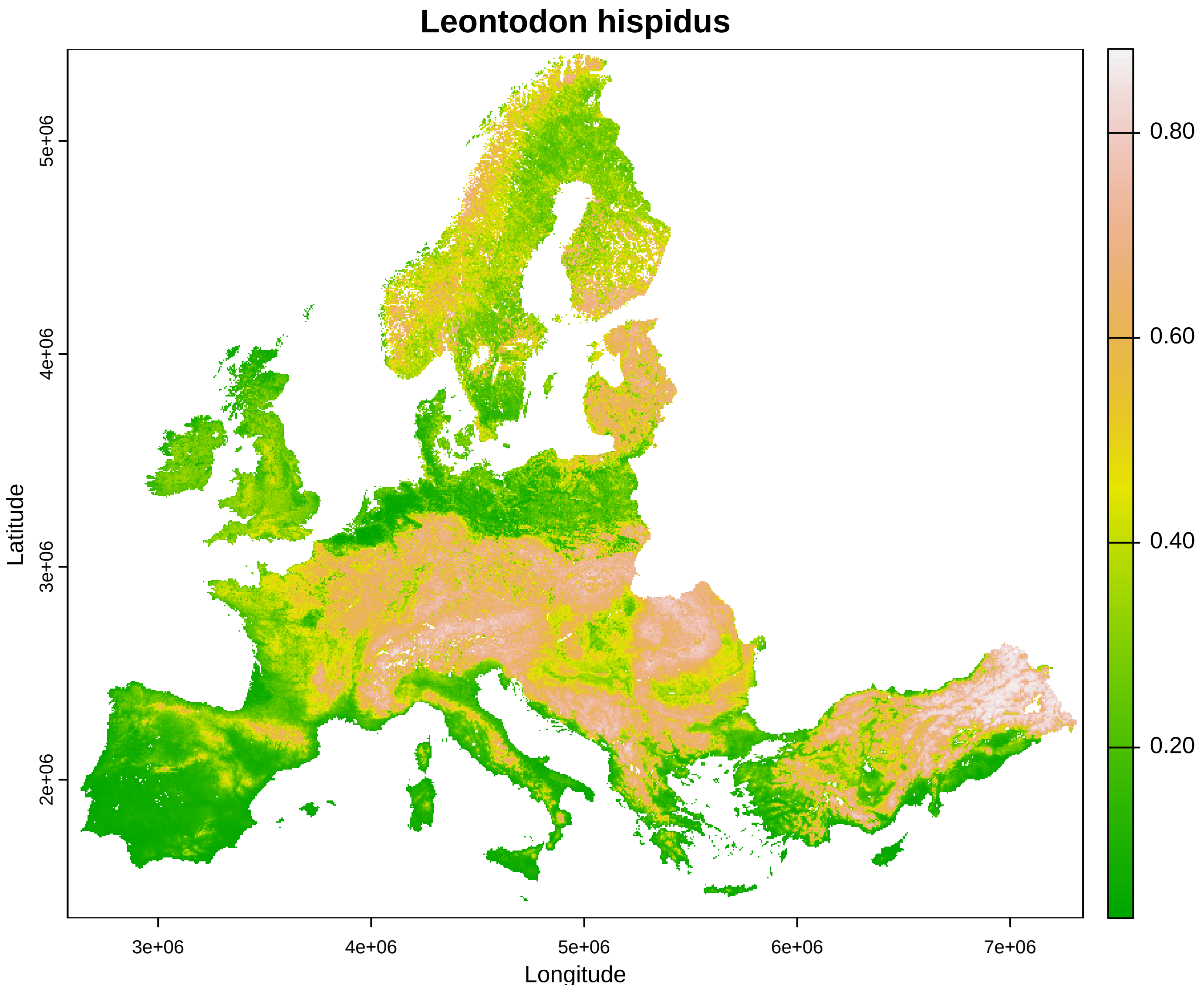
Leontodon hispidus
Latitude
Longitude
5e+06
4e+06
3e+06
2e+06
3e+06
4e+06
5e+06
6e+06
7e+06
0.80
0.60
0.40
0.20

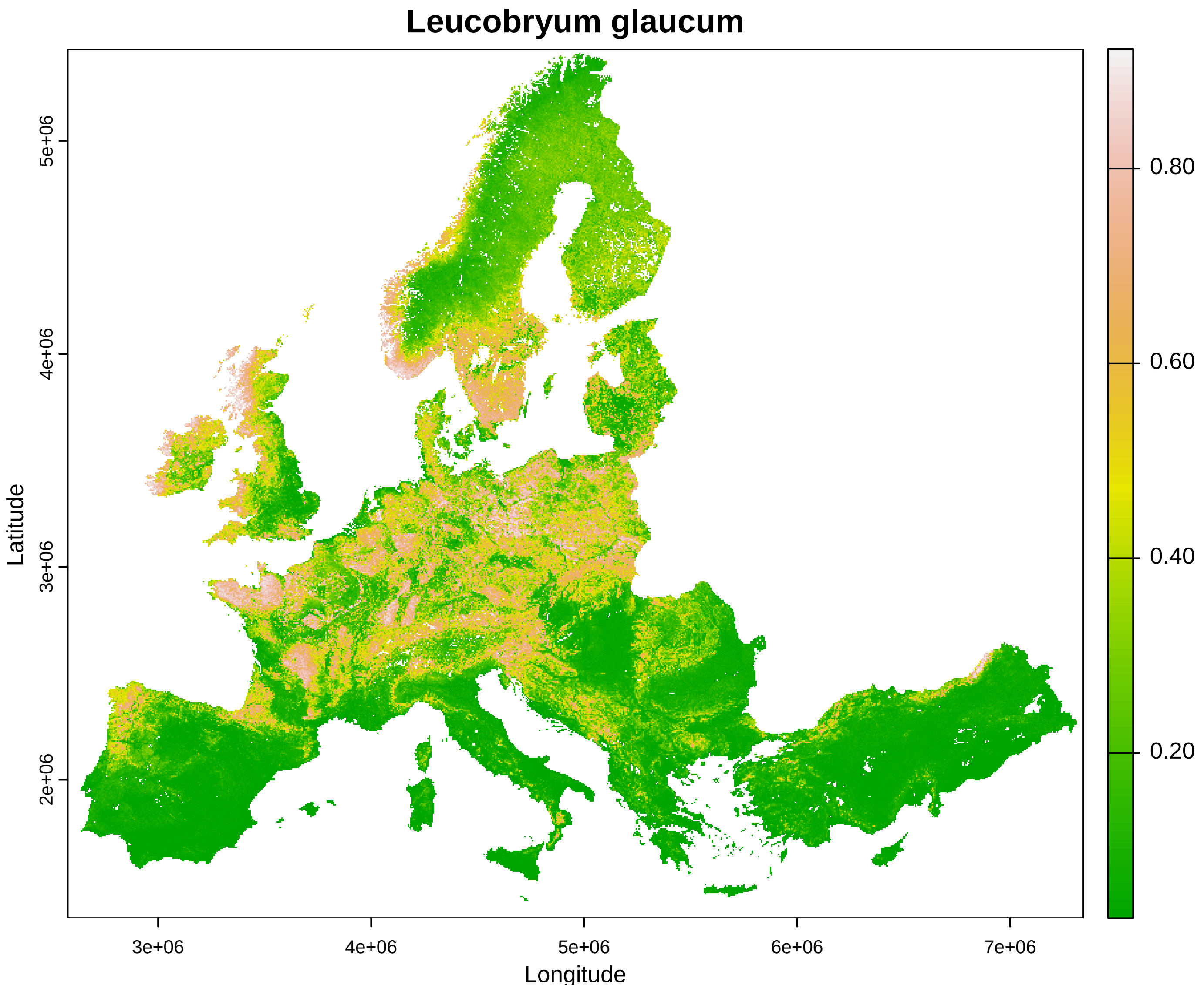

Leucobryum glaucum
Latitude
Longitude
5e+06
4e+06
3e+06
2e+06
3e+06
4e+06
5e+06
6e+06
7e+06
0.80
0.60
0.40
0.20

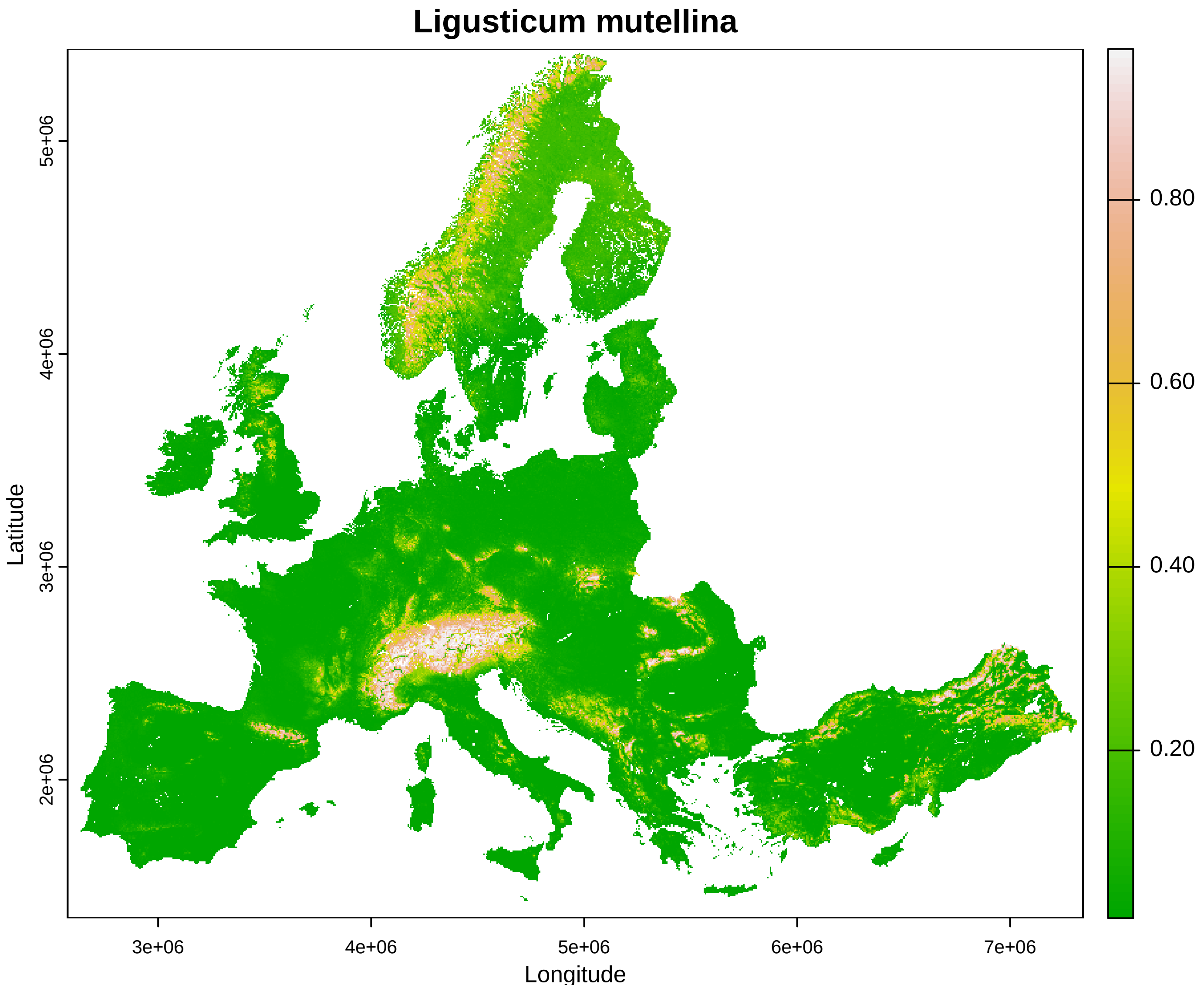
Ligusticum mutellina
Latitude
Longitude
5e+06
4e+06
3e+06
2e+06
3e+06
4e+06
5e+06
6e+06
7e+06
0.80
0.60
0.40
0.20

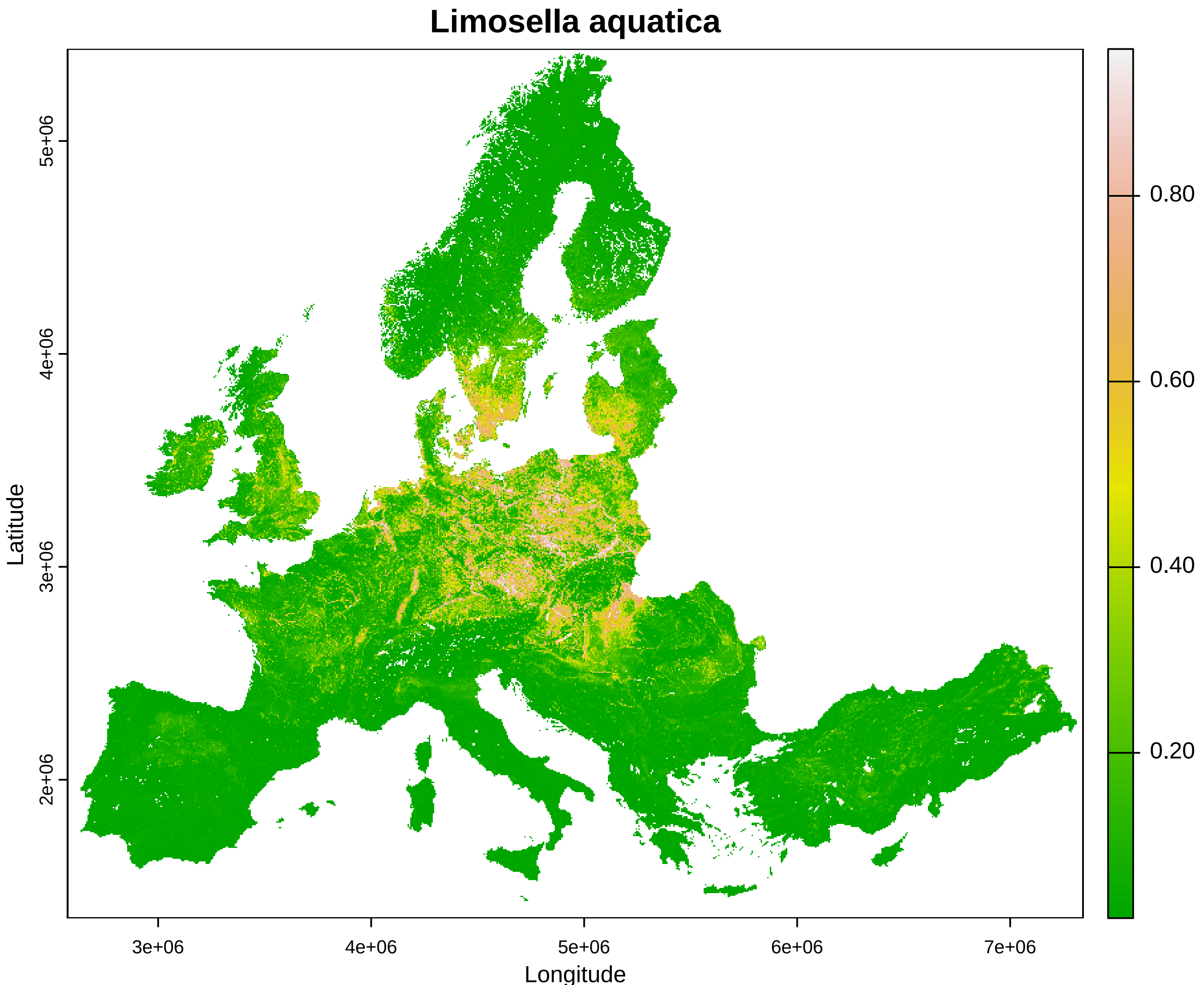
Limosella aquatica
Latitude
Longitude
5e+06
4e+06
3e+06
2e+06
3e+06
4e+06
5e+06
6e+06
7e+06
0.80
0.60
0.40
0.20

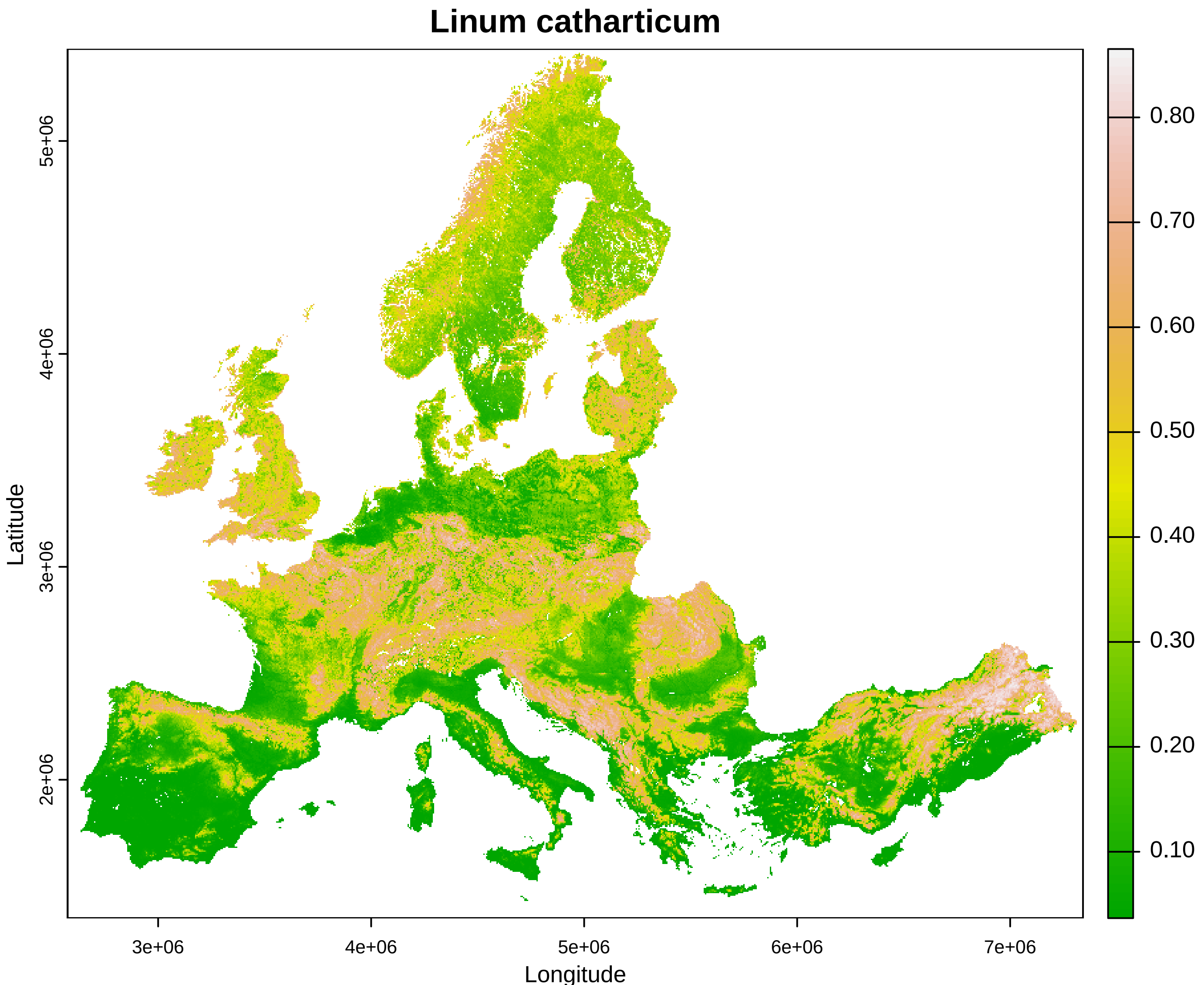
Linum catharticum
Latitude
5e+06
4e+06
3e+06
2e+06
3e+06
4e+06
5e+06
6e+06
7e+06
Longitude
0.80
0.70
0.60
0.50
0.40
0.30
0.20
0.10

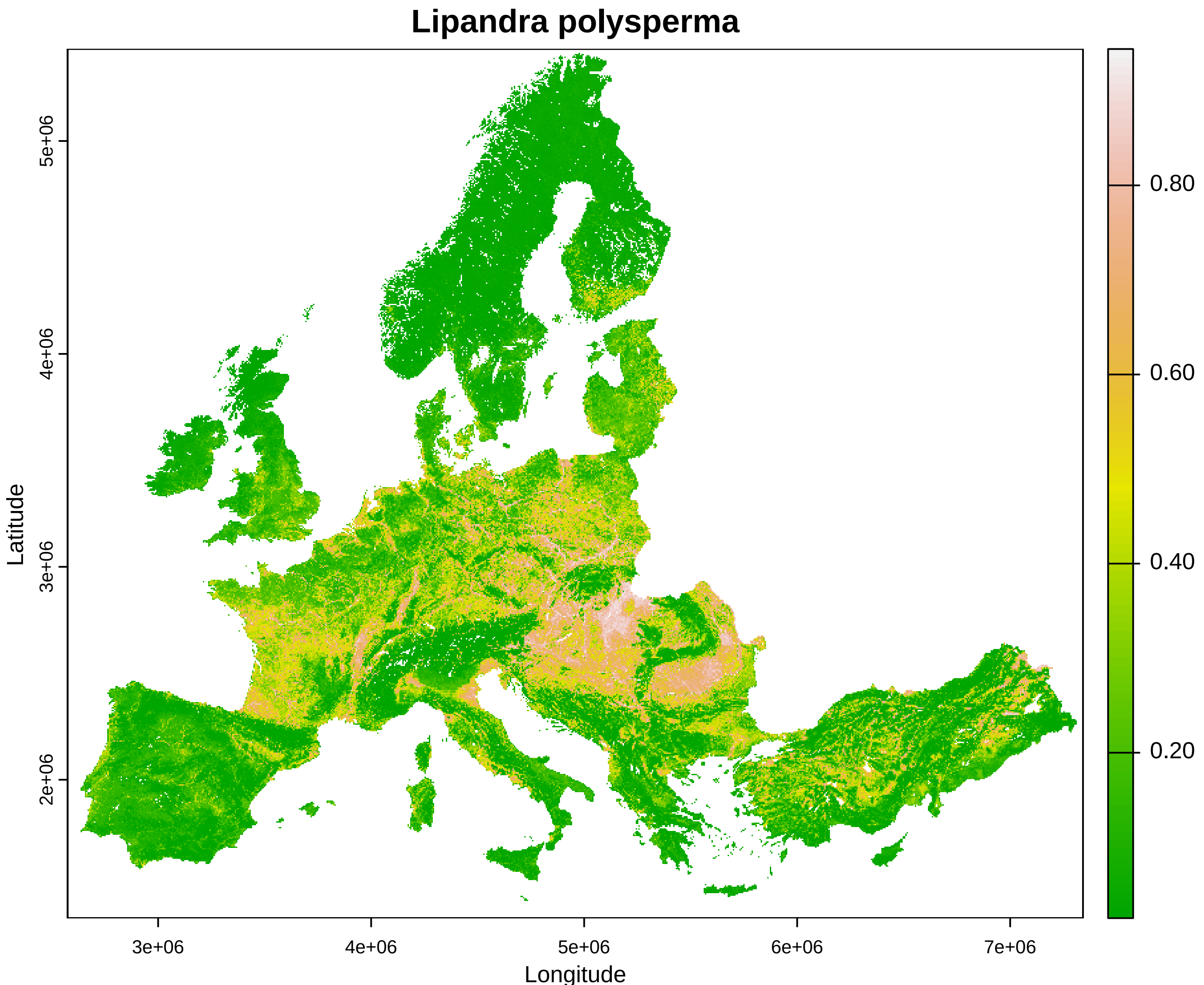

Lipandra polysperma
Latitude
Longitude
5e+06
4e+06
3e+06
2e+06
3e+06
4e+06
5e+06
6e+06
7e+06
0.80
0.60
0.40
0.20

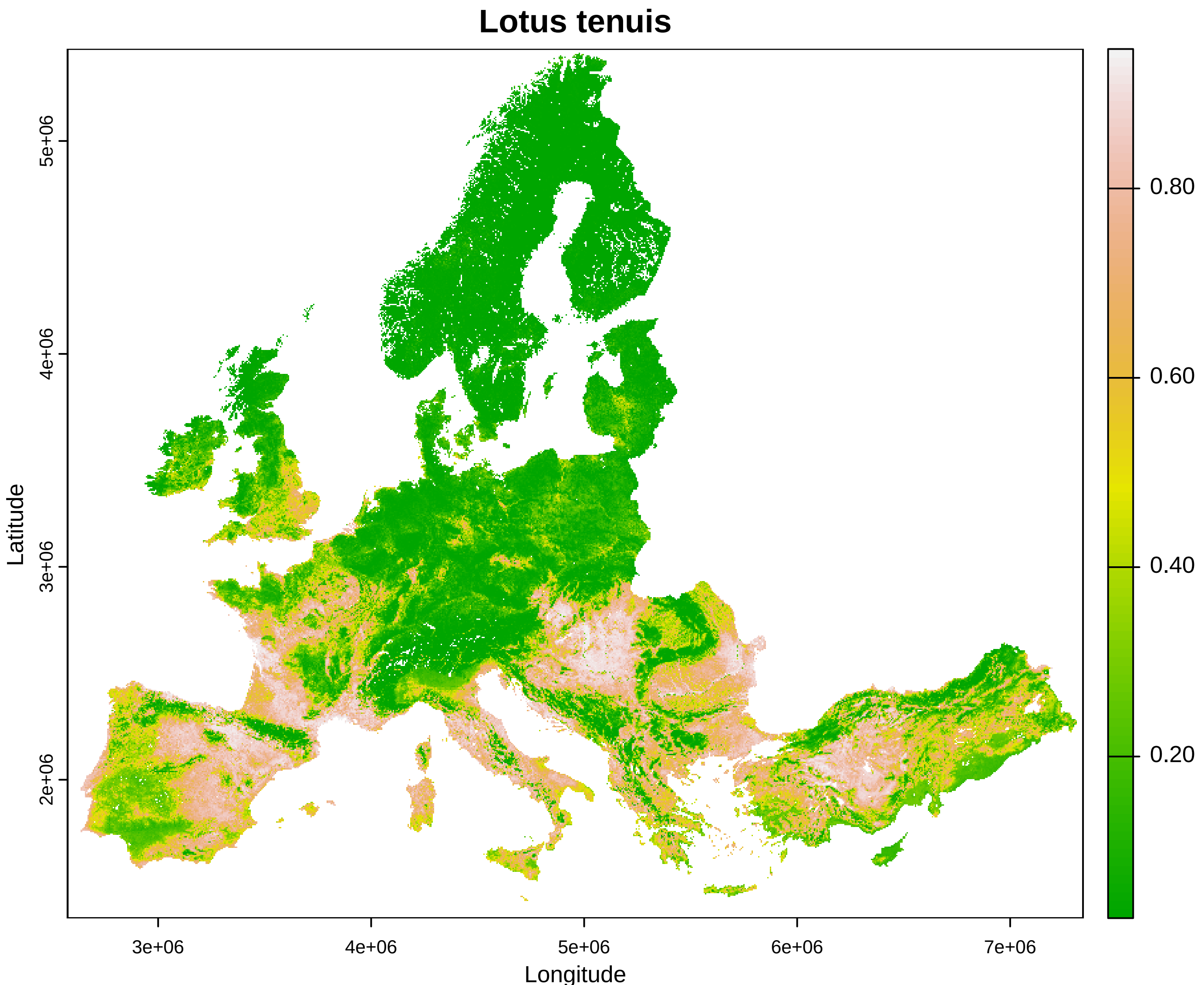

Lotus tenuis
Latitude
Longitude
5e+06
4e+06
3e+06
2e+06
3e+06
4e+06
5e+06
6e+06
7e+06
0.80
0.60
0.40
0.20

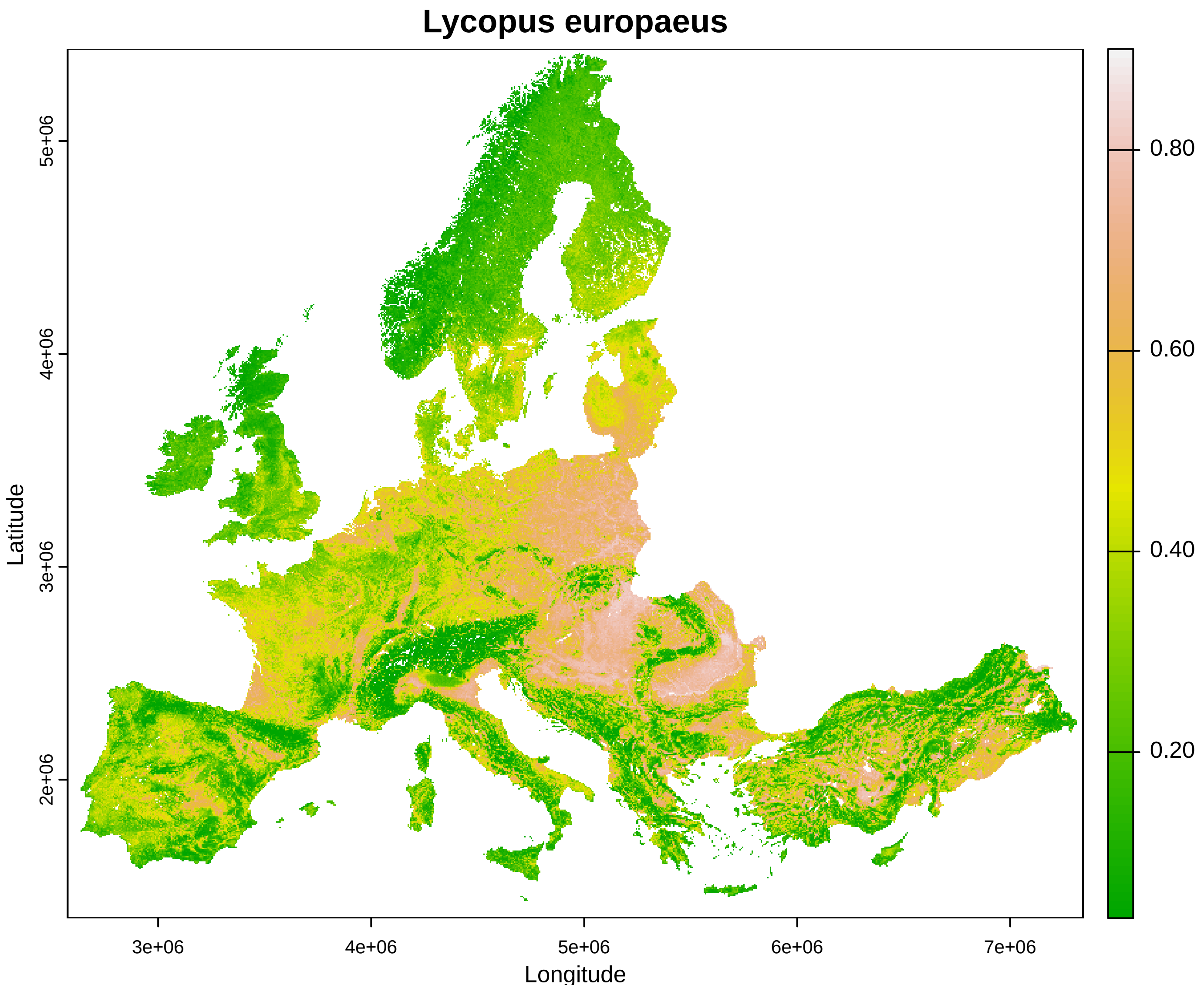

Lycopus europaeus
Latitude
Longitude
5e+06
4e+06
3e+06
2e+06
3e+06
4e+06
5e+06
6e+06
7e+06
0.80
0.60
0.40
0.20

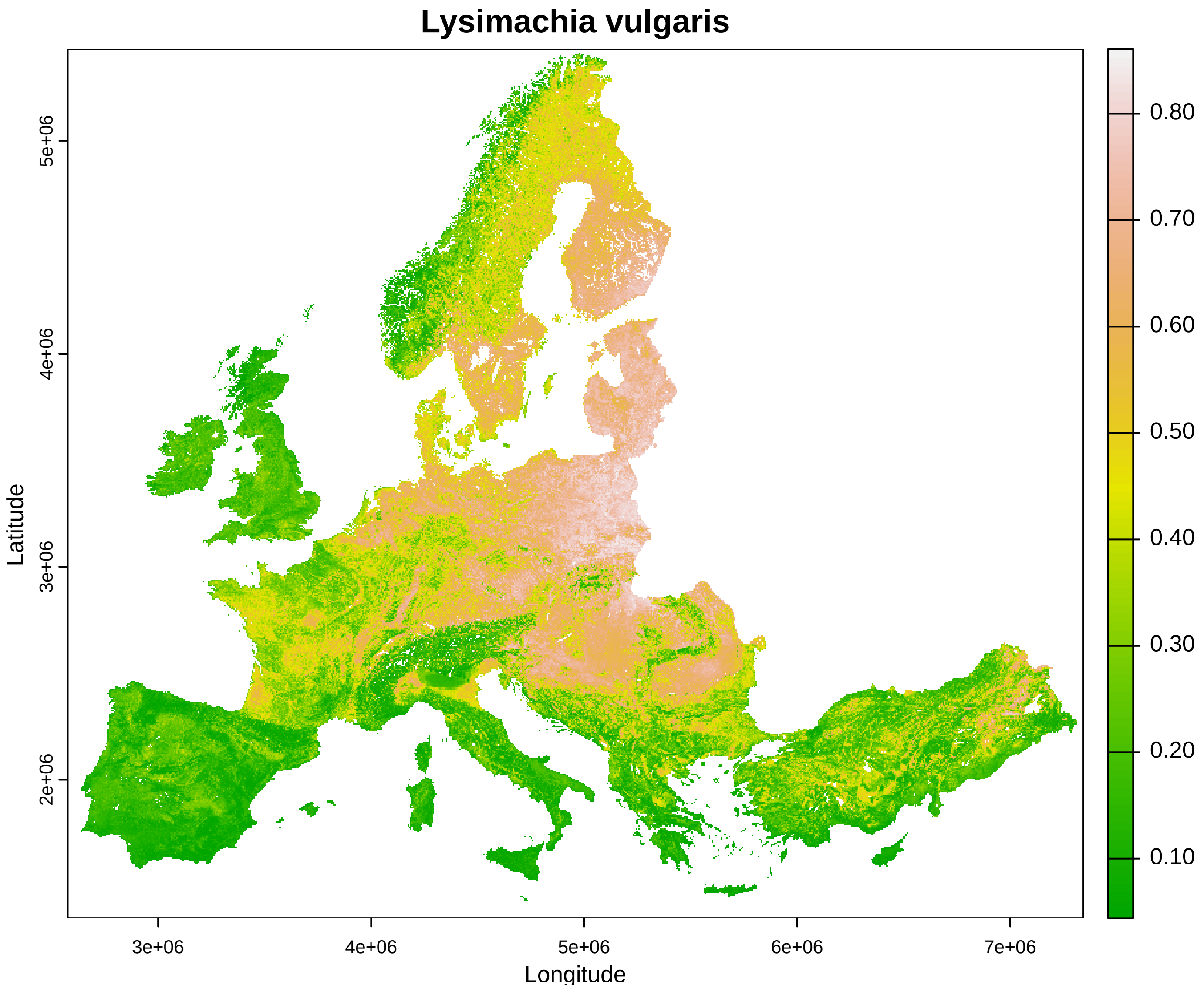

Lysimachia vulgaris
Latitude
Longitude
5e+06
4e+06
3e+06
2e+06
3e+06
4e+06
5e+06
6e+06
7e+06
0.80
0.70
0.60
0.50
0.40
0.30
0.20
0.10

Lythrum hyssopifolia

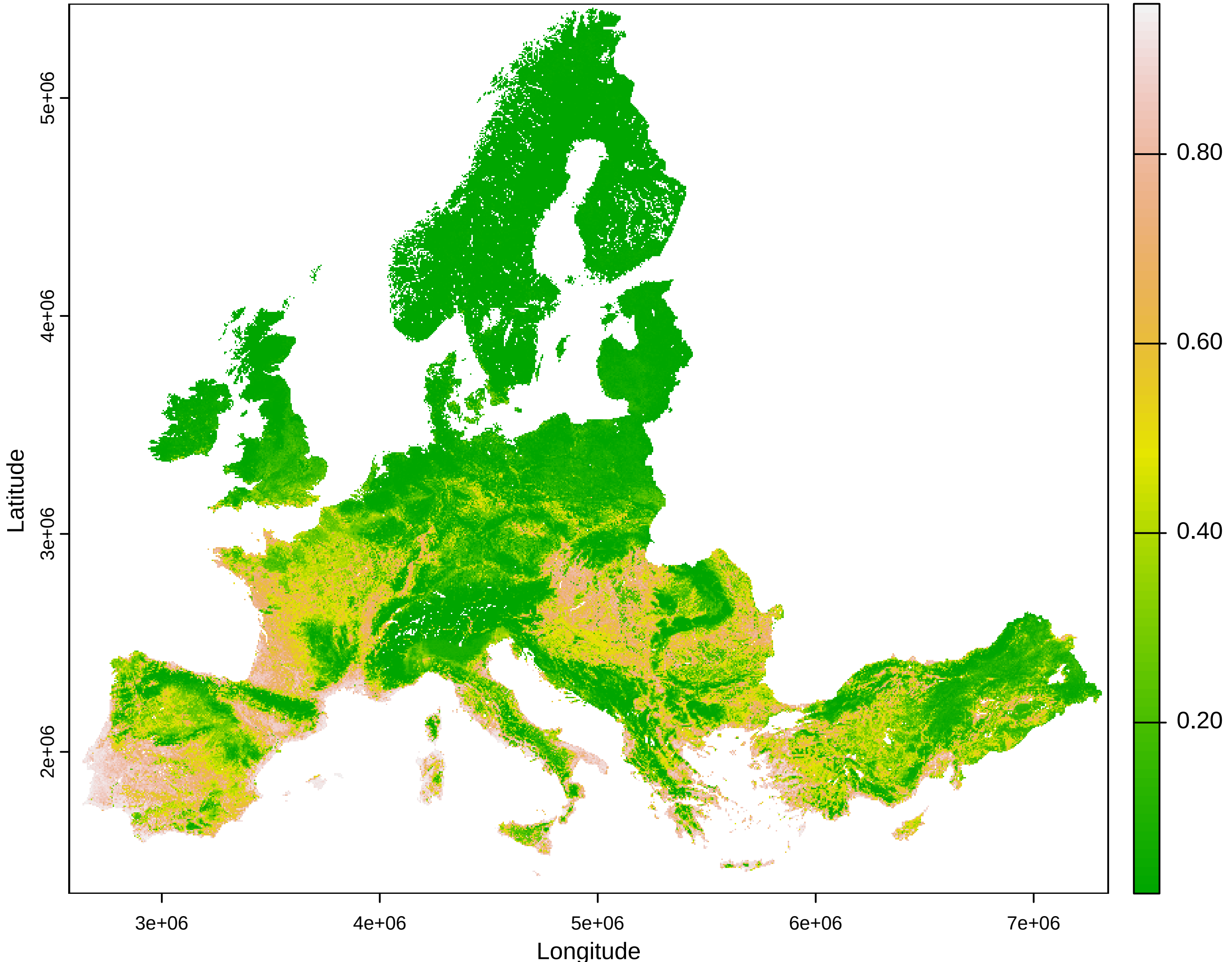

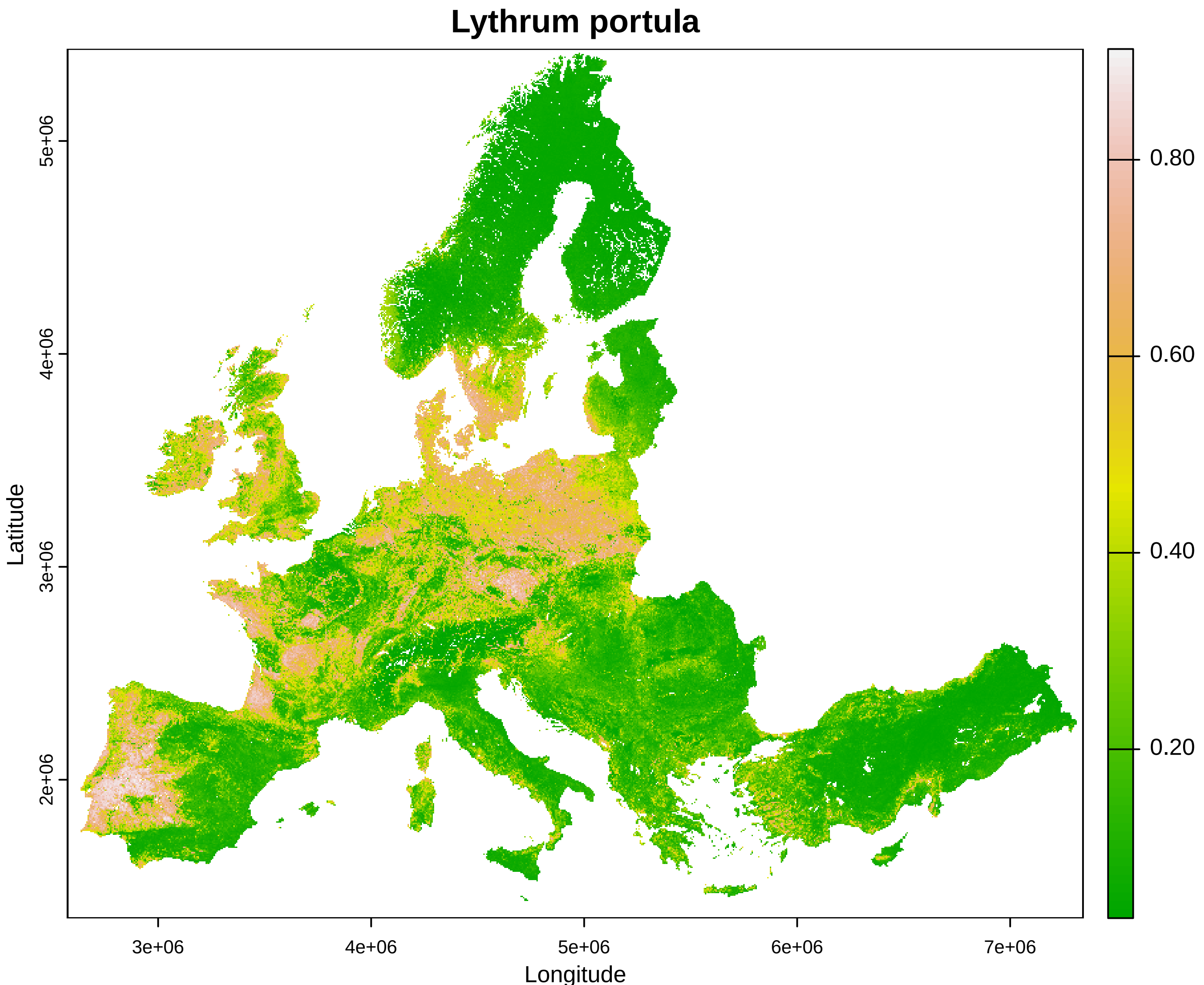

Lythrum portula
Latitude
Longitude
5e+06
4e+06
3e+06
2e+06
3e+06
4e+06
5e+06
6e+06
7e+06
0.80
0.60
0.40
0.20

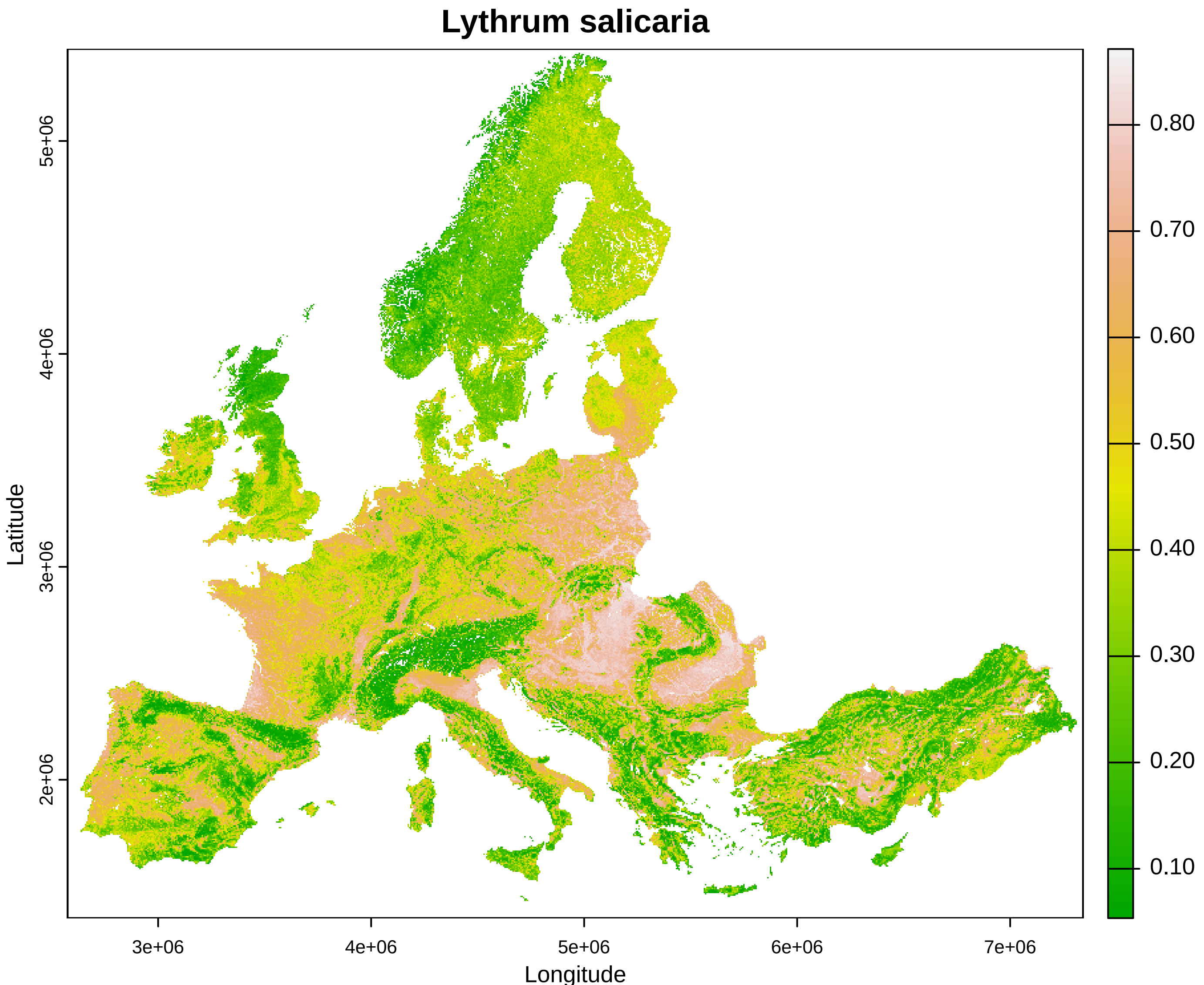
Lythrum salicaria
Latitude
5e+06
4e+06
3e+06
2e+06
3e+06
4e+06
5e+06
6e+06
7e+06
Longitude
0.80
0.70
0.60
0.50
0.40
0.30
0.20
0.10

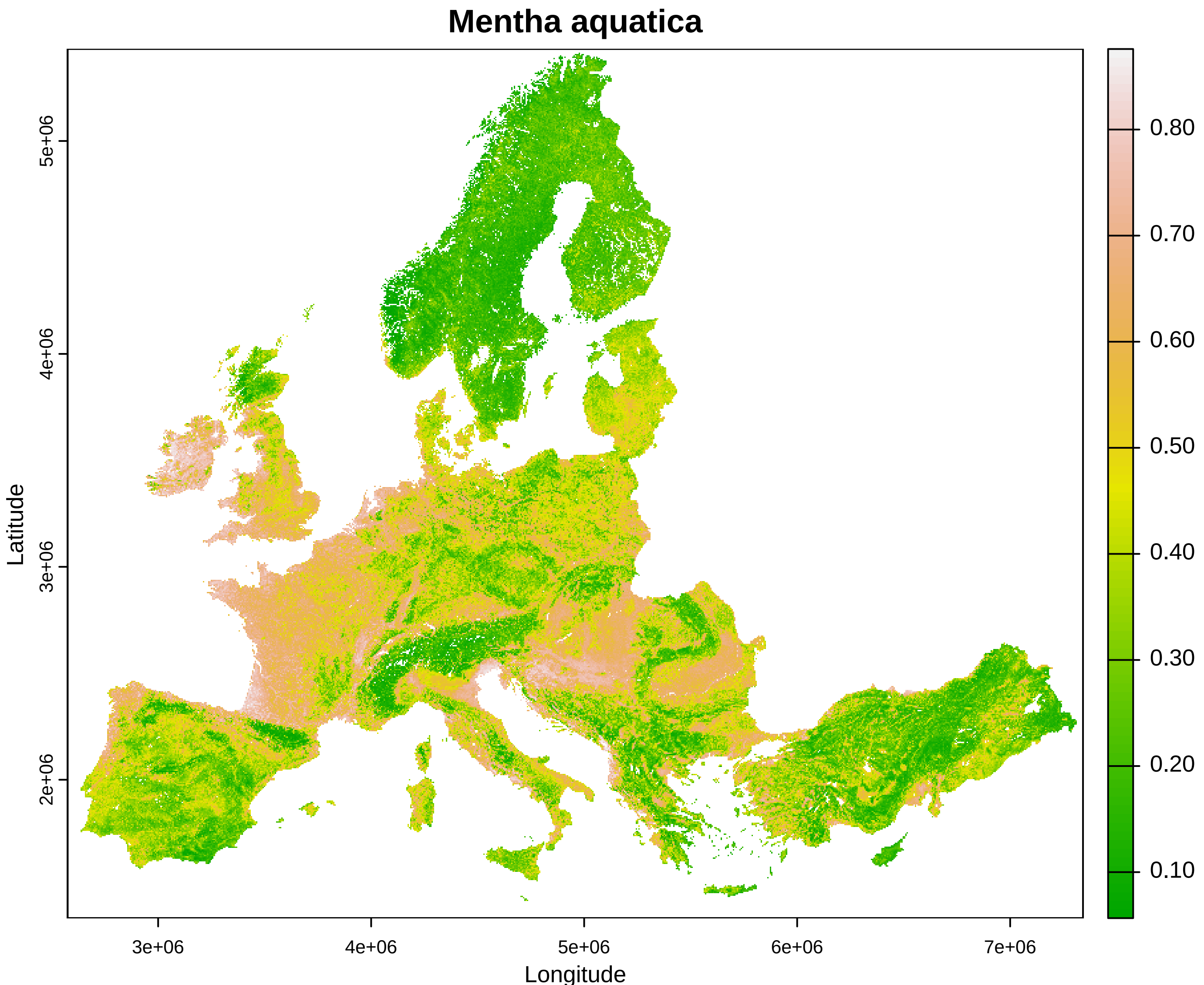

Mentha aquatica
Latitude
Longitude
5e+06
4e+06
3e+06
2e+06
3e+06
4e+06
5e+06
6e+06
7e+06
0.80
0.70
0.60
0.50
0.40
0.30
0.20
0.10

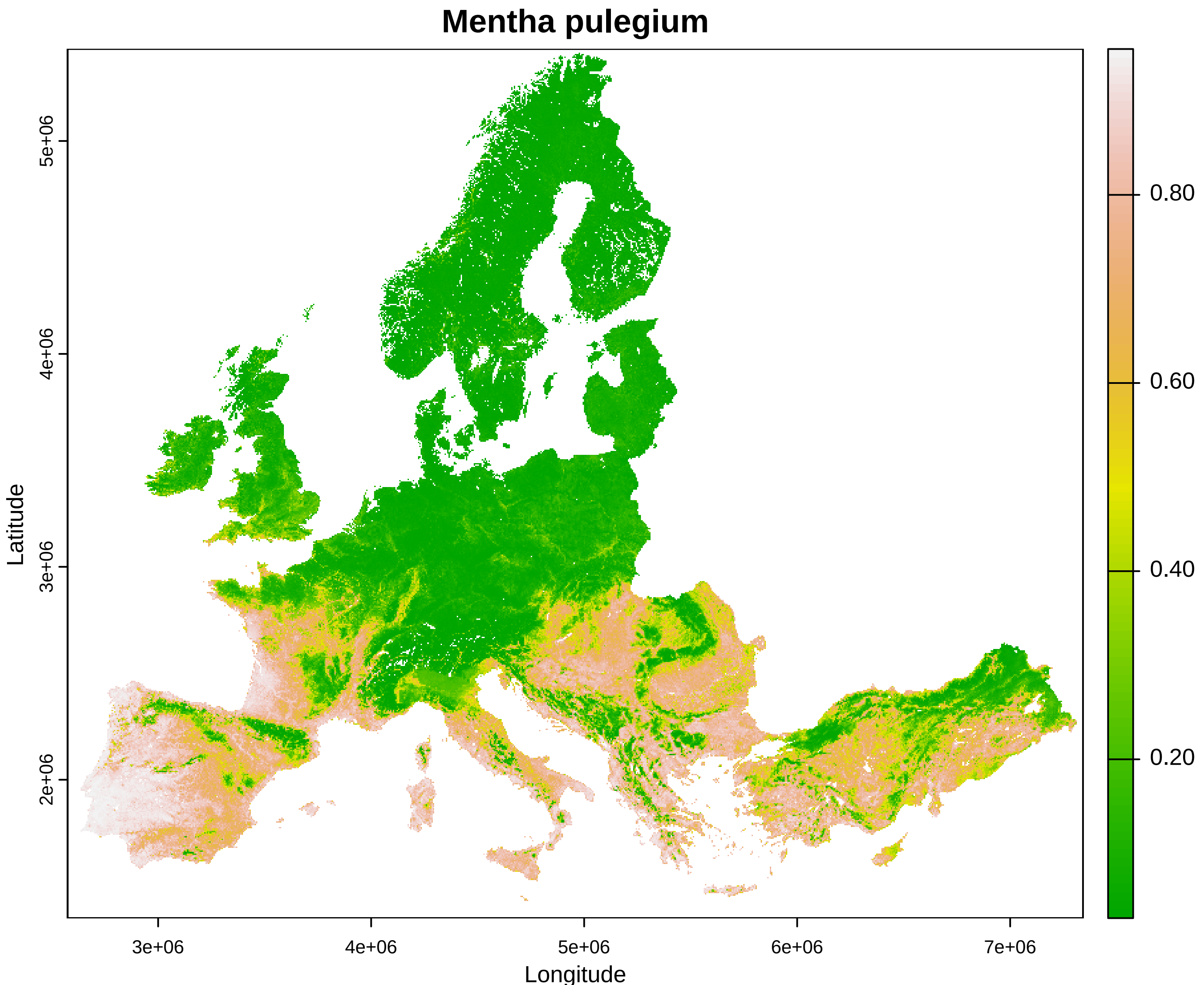
Mentha pulegium
Latitude
Longitude
5e+06
4e+06
3e+06
2e+06
3e+06
4e+06
5e+06
6e+06
7e+06
0.80
0.60
0.40
0.20

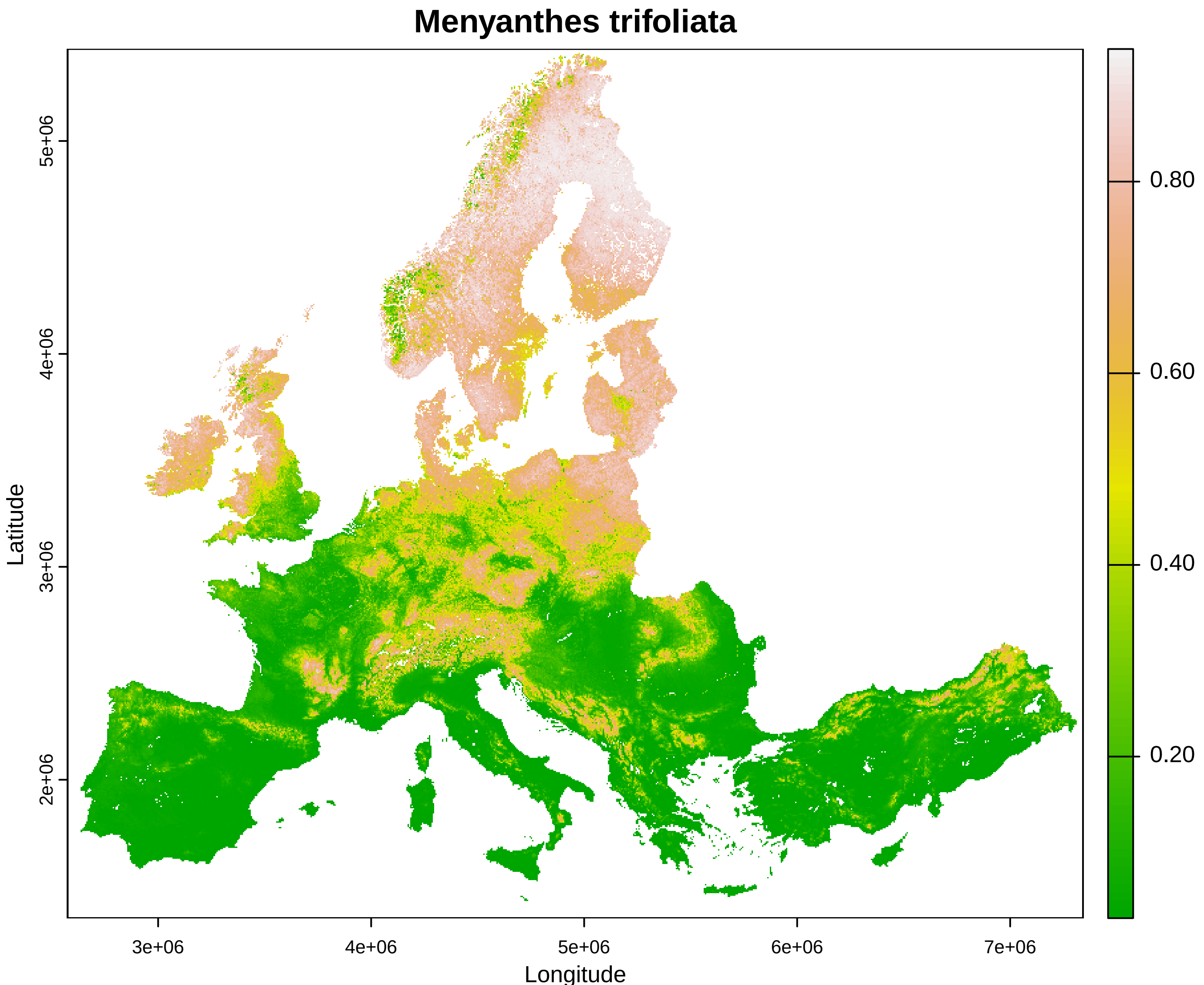
Menyanthes trifoliata
Latitude
Longitude
5e+06
4e+06
3e+06
2e+06
3e+06
4e+06
5e+06
6e+06
7e+06
0.80
0.60
0.40
0.20

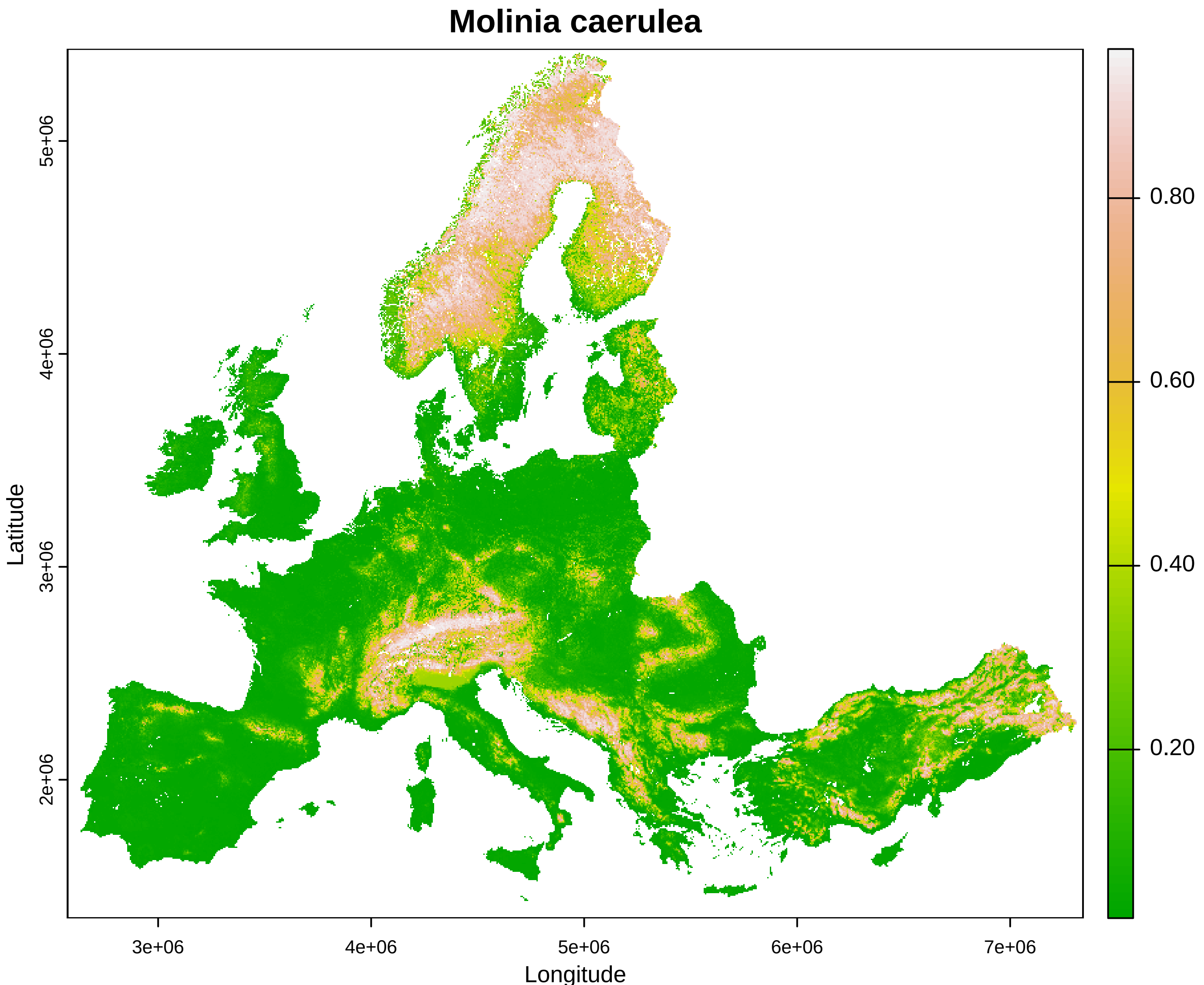
Molinia caerulea
Latitude
Longitude
5e+06
4e+06
3e+06
2e+06
3e+06
4e+06
5e+06
6e+06
7e+06
0.80
0.60
0.40
0.20

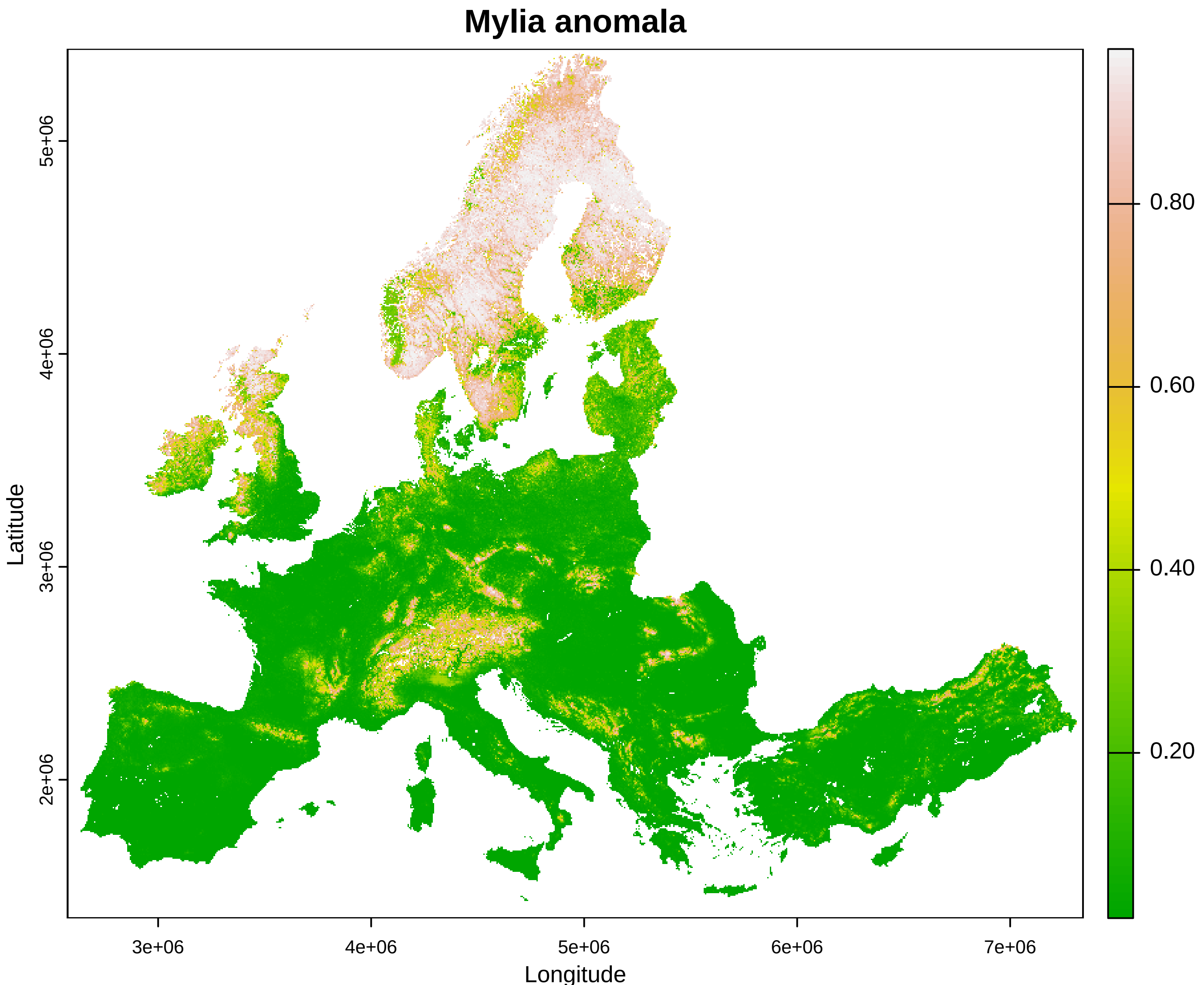
Mylia anomala
Latitude
5e+06
4e+06
3e+06
2e+06
3e+06
4e+06
5e+06
6e+06
7e+06
Longitude
0.80
0.60
0.40
0.20

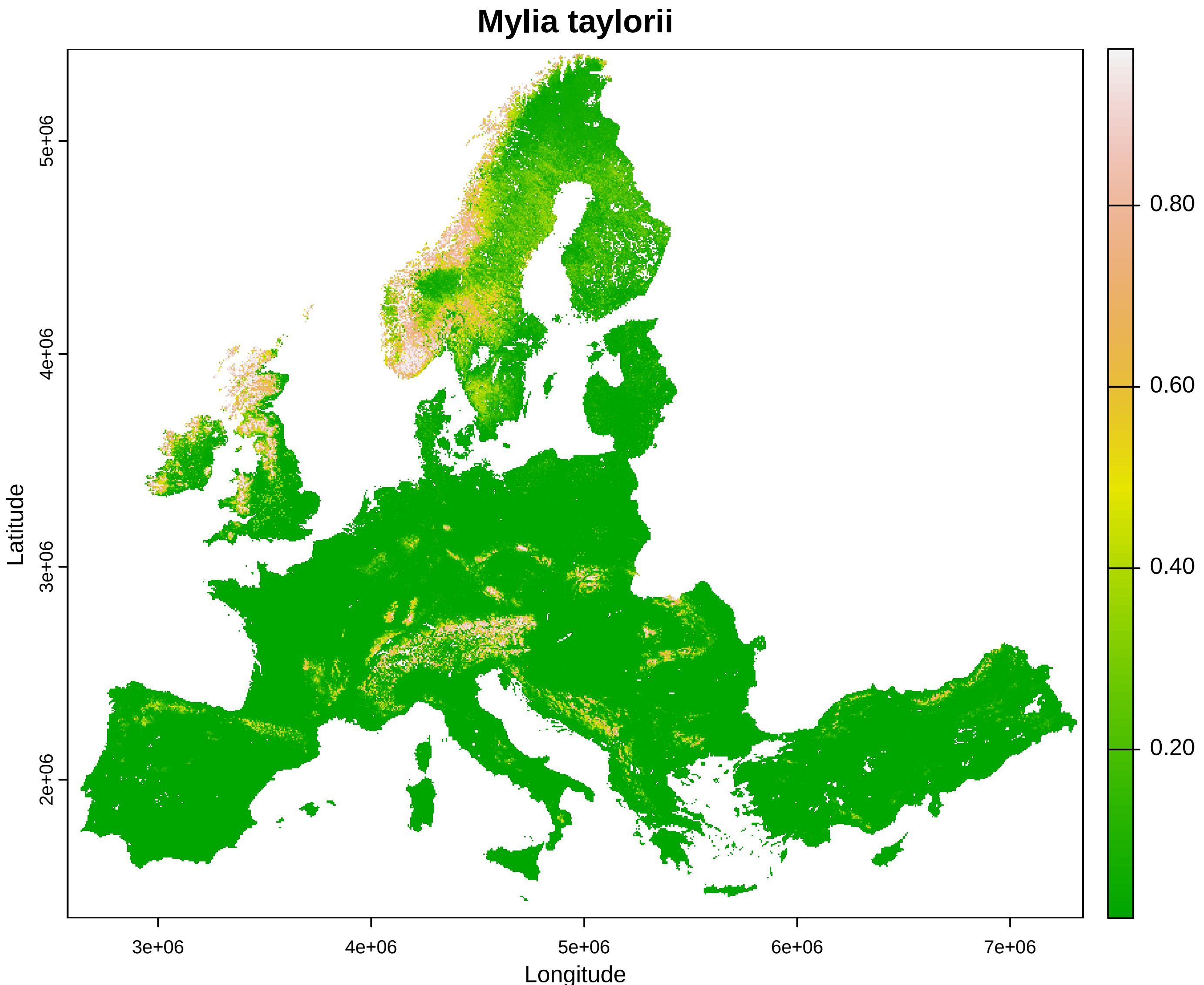

Mylia taylorii
Latitude
5e+06
4e+06
3e+06
2e+06
3e+06
4e+06
5e+06
6e+06
7e+06
Longitude
0.80
0.60
0.40
0.20

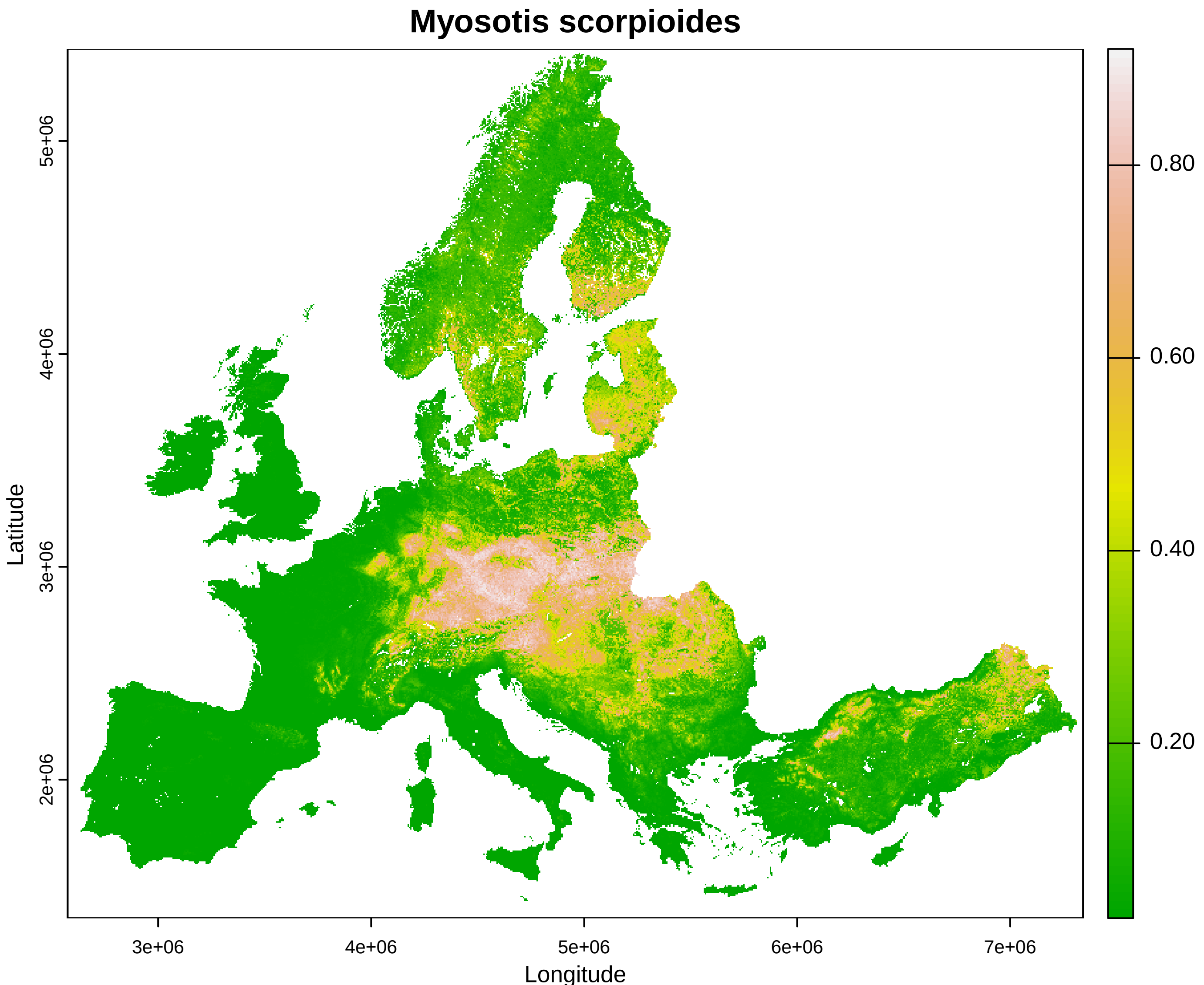

Myosotis scorpioides
Latitude
Longitude
5e+06
4e+06
3e+06
2e+06
3e+06
4e+06
5e+06
6e+06
7e+06
0.80
0.60
0.40
0.20

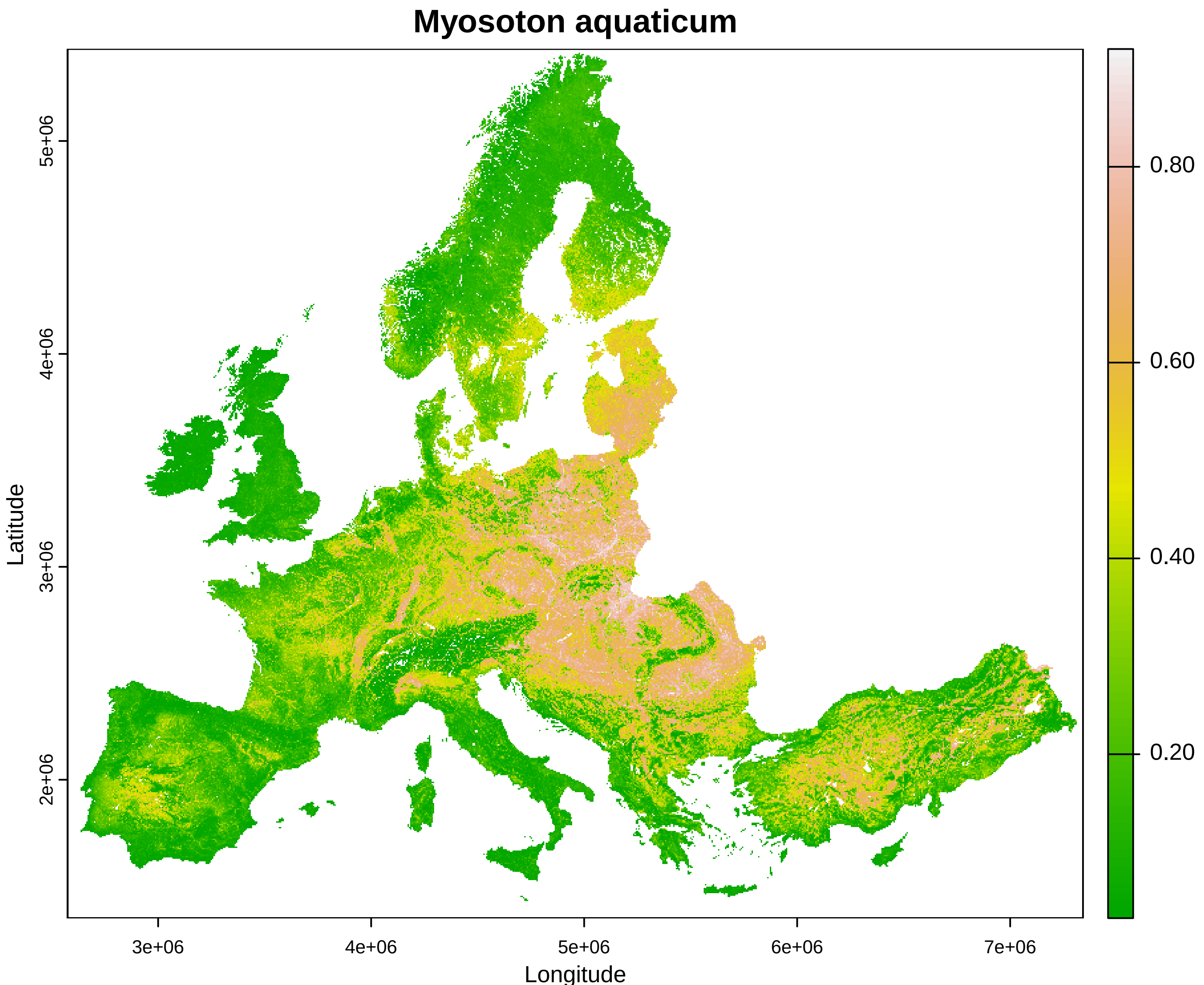

Myosoton aquaticum
Latitude
Longitude
5e+06
4e+06
3e+06
2e+06
3e+06
4e+06
5e+06
6e+06
7e+06
0.80
0.60
0.40
0.20

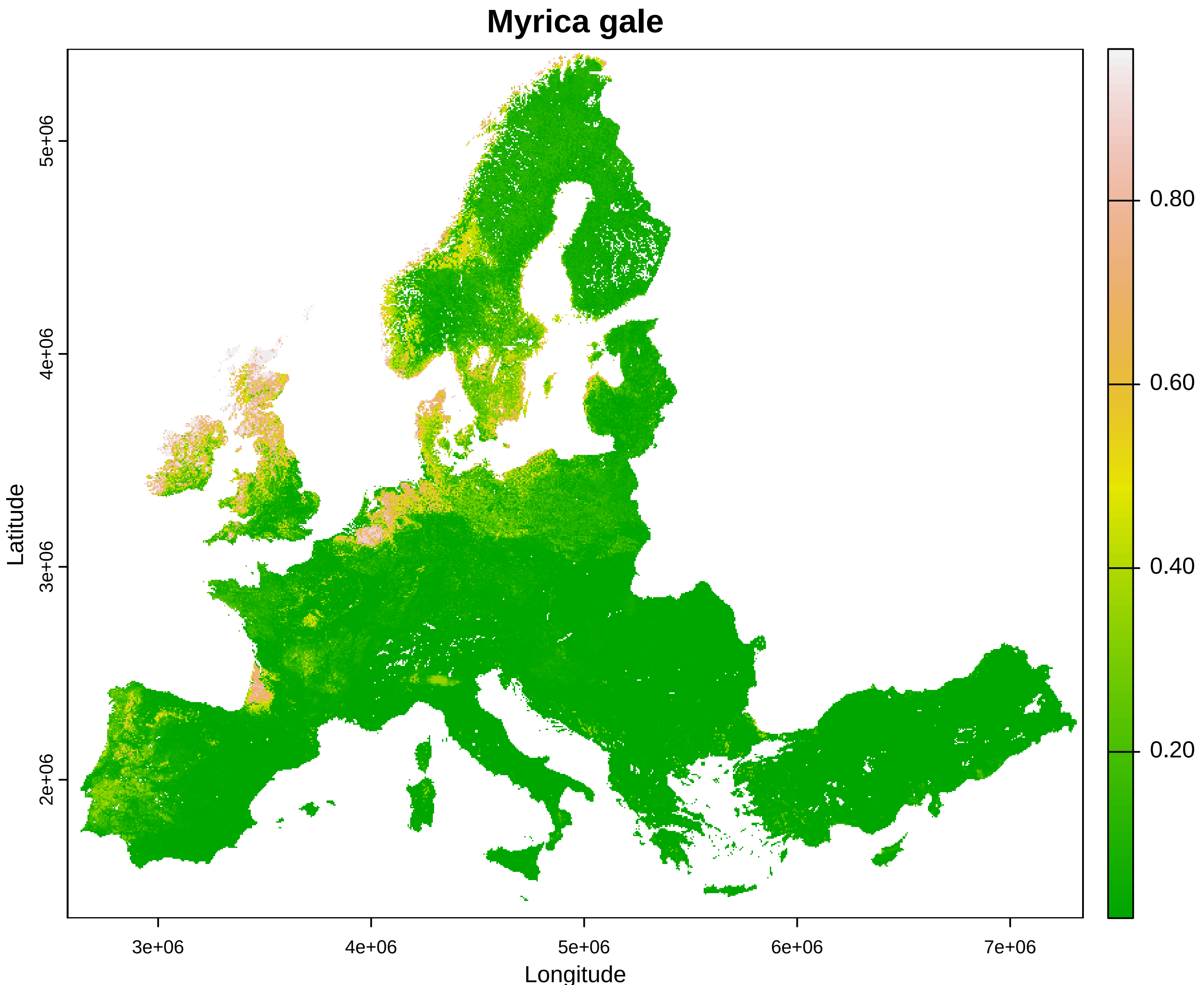

Myrica gale
Latitude
Longitude
5e+06
4e+06
3e+06
2e+06
3e+06
4e+06
5e+06
6e+06
7e+06
0.80
0.60
0.40
0.20

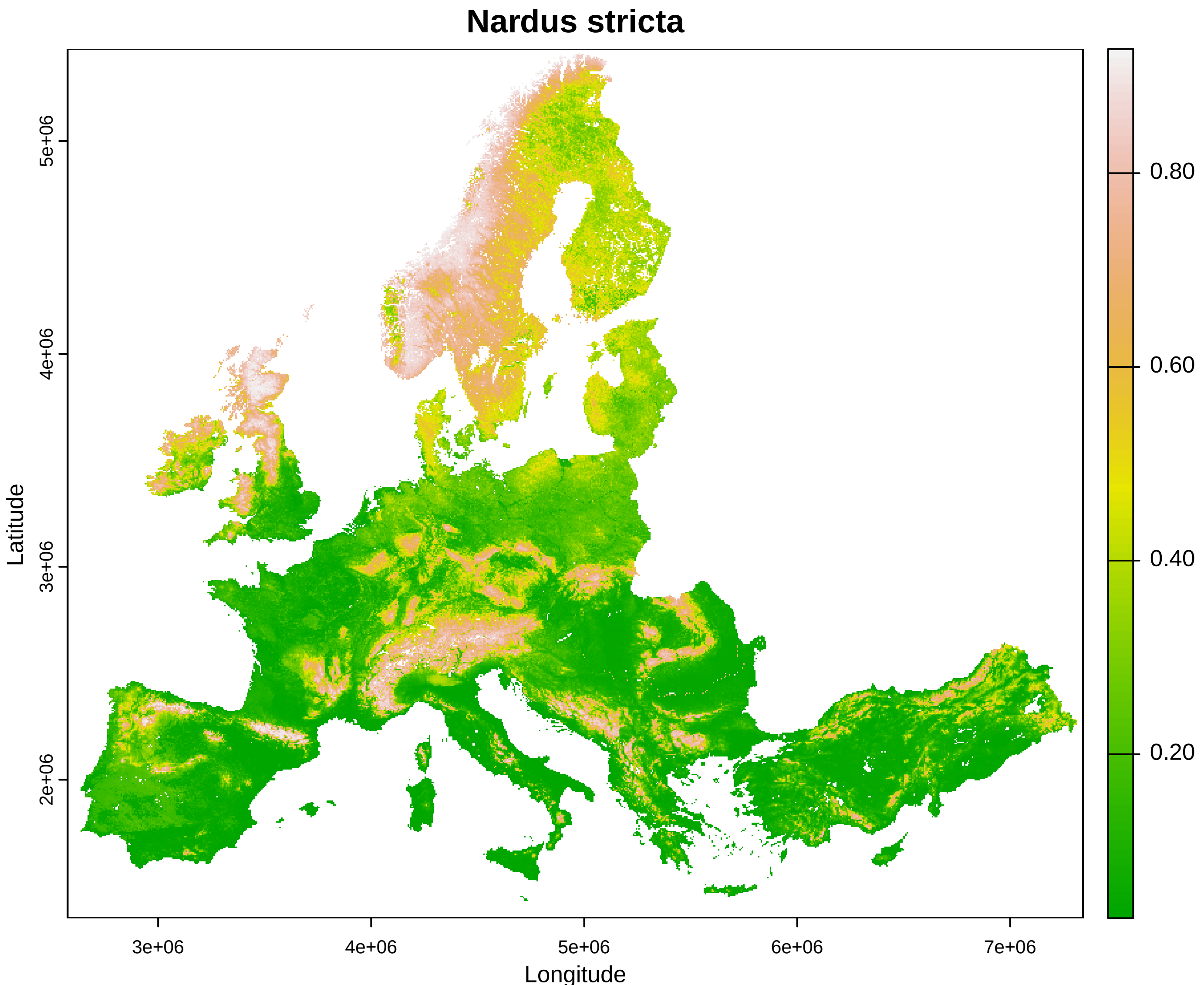

Nardus stricta
Latitude
Longitude
5e+06
4e+06
3e+06
2e+06
3e+06
4e+06
5e+06
6e+06
7e+06
0.80
0.60
0.40
0.20

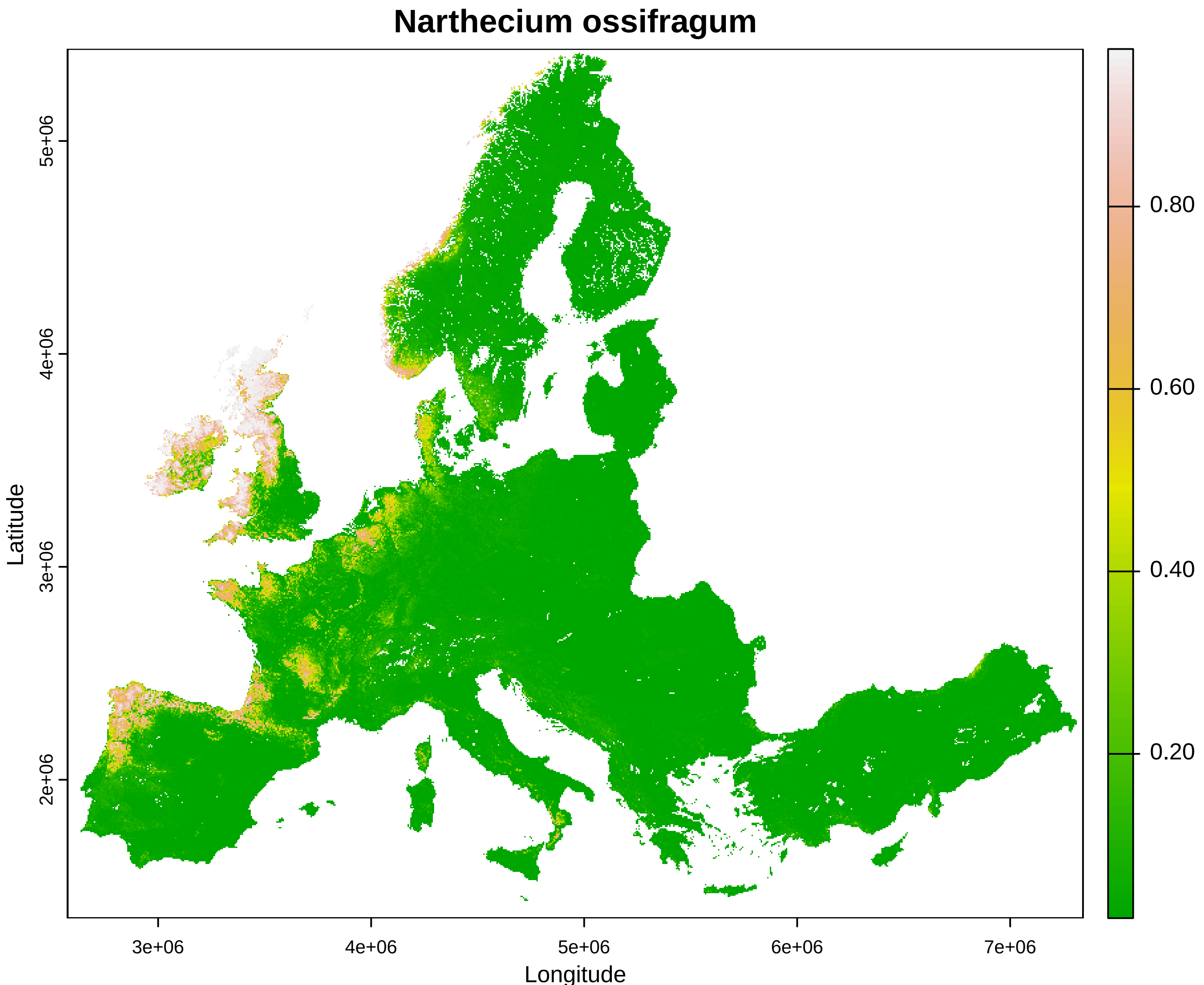
Narthecium ossifragum
Latitude
Longitude
5e+06
4e+06
3e+06
2e+06
3e+06
4e+06
5e+06
6e+06
7e+06
0.80
0.60
0.40
0.20

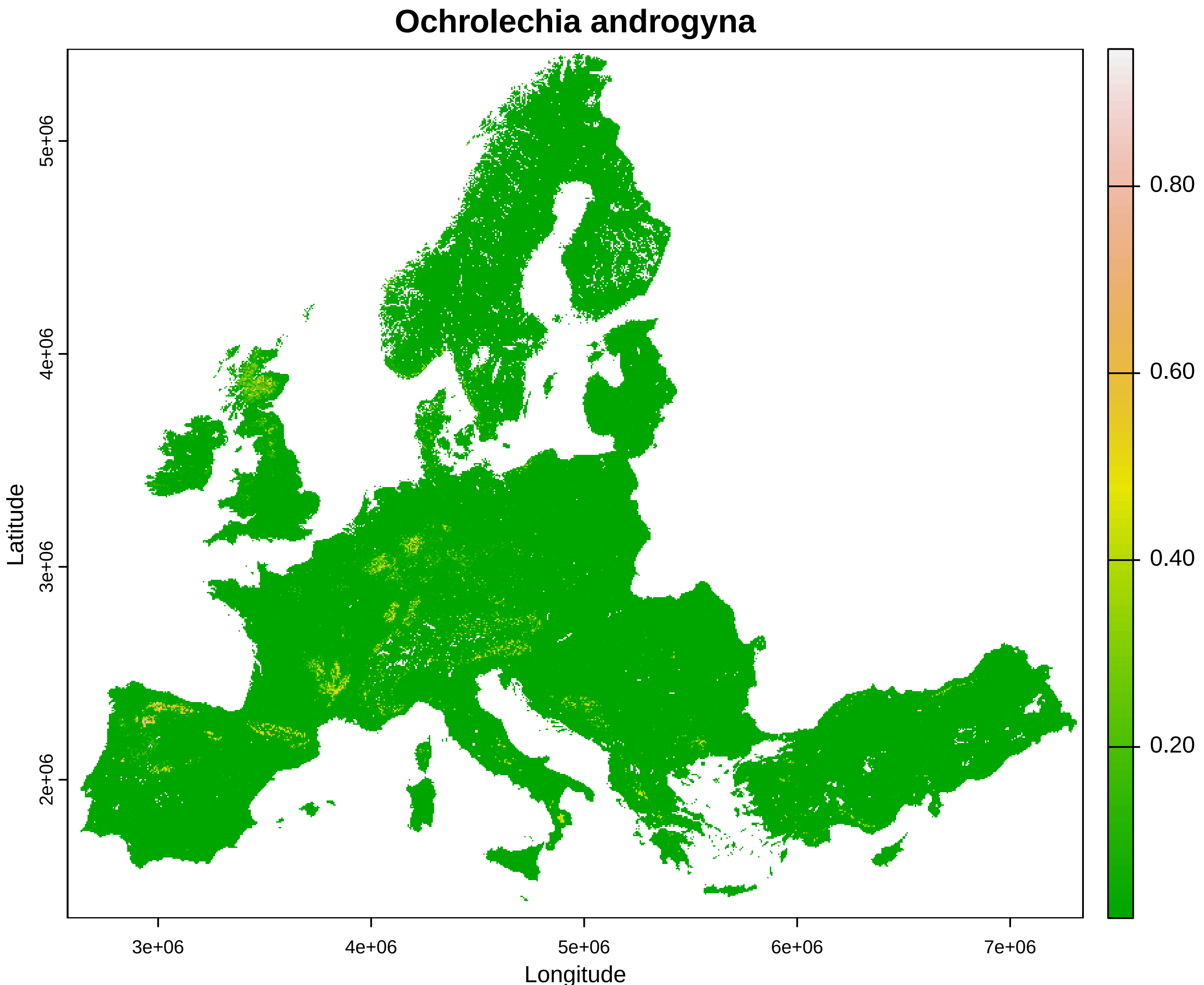

Ochrolechia androgyna
Latitude
Longitude
5e+06
4e+06
3e+06
2e+06
3e+06
4e+06
5e+06
6e+06
7e+06
0.80
0.60
0.40
0.20

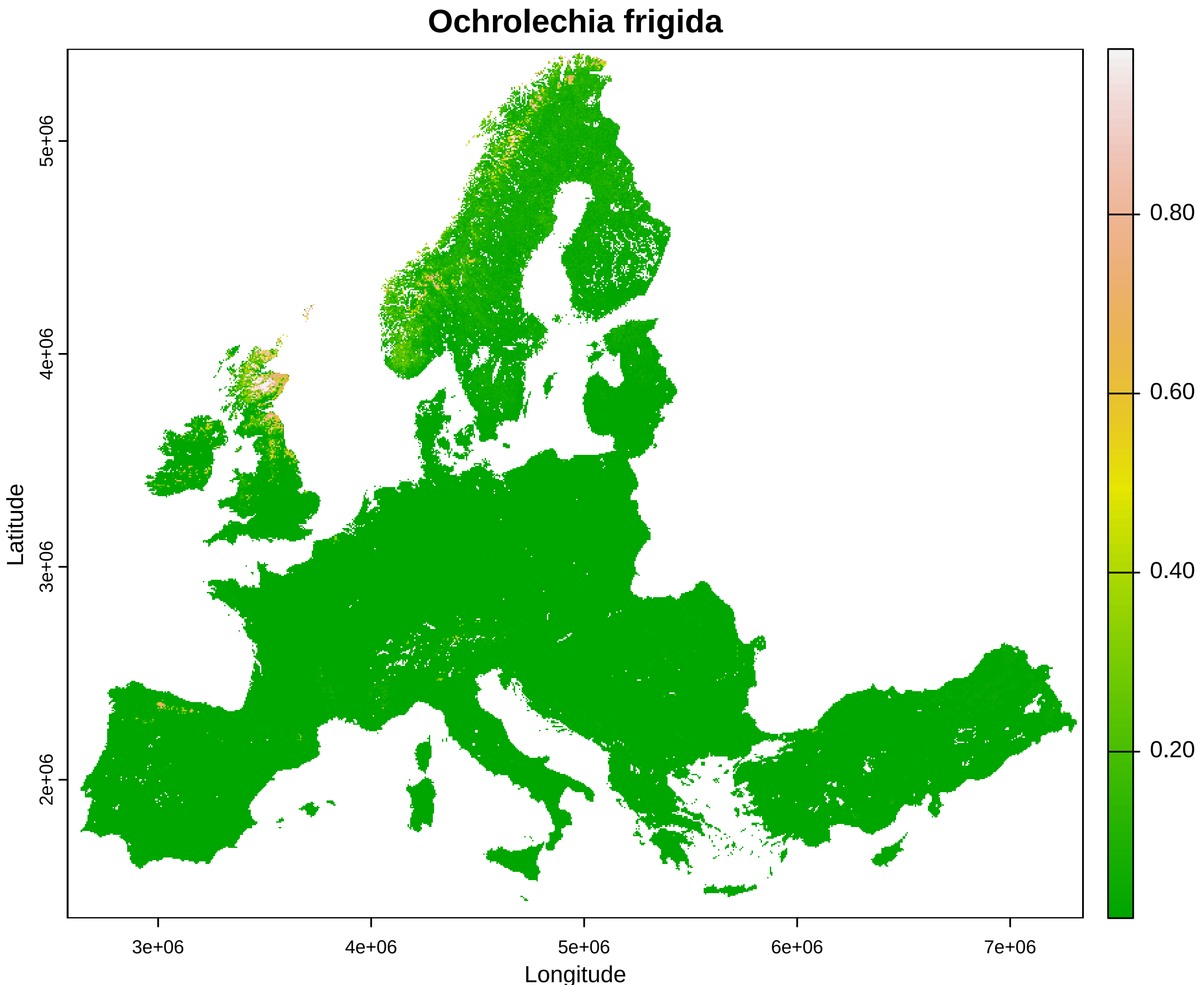

Ochrolechia frigida
Latitude
Longitude
5e+06
4e+06
3e+06
2e+06
3e+06
4e+06
5e+06
6e+06
7e+06
0.80
0.60
0.40
0.20

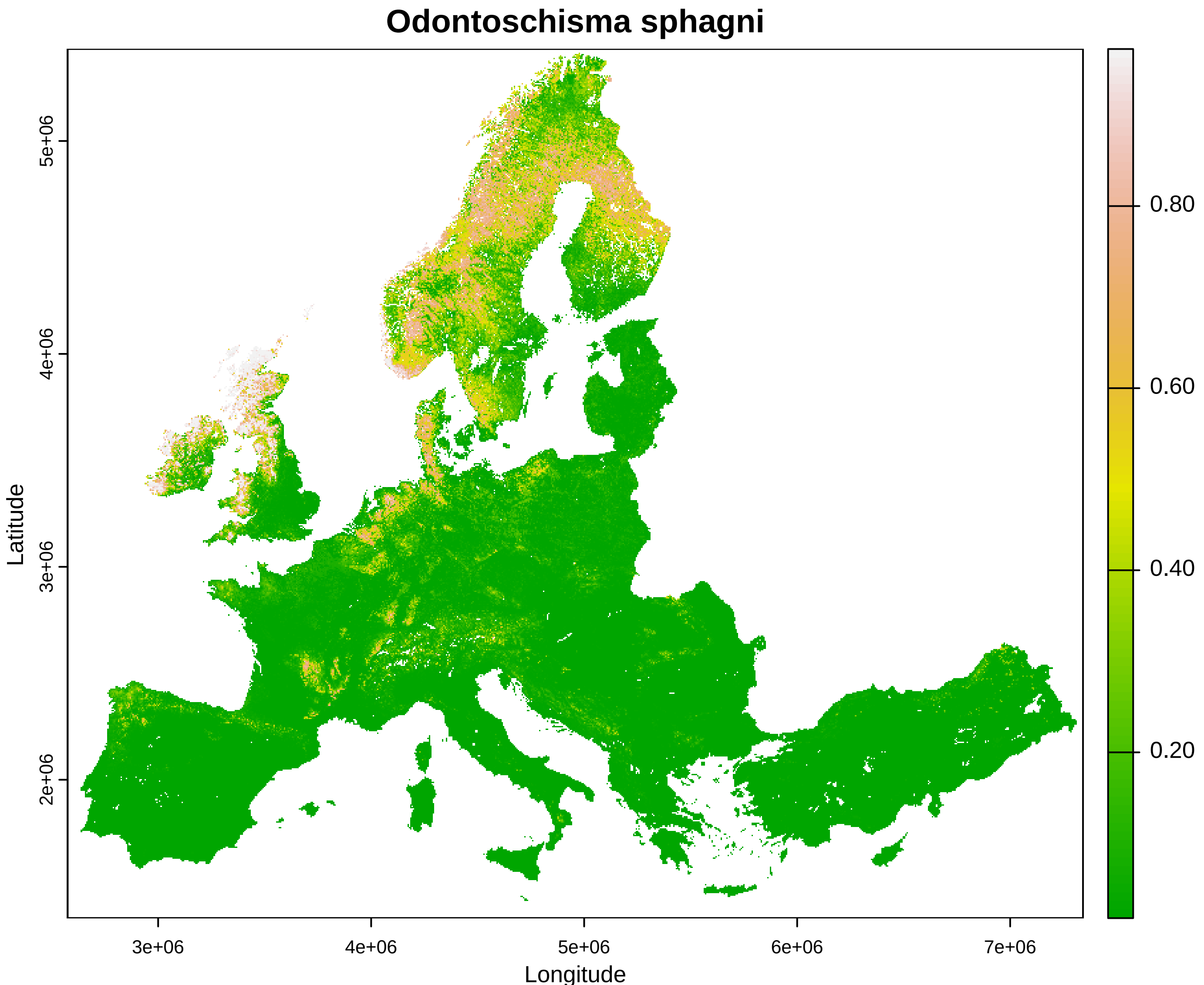

Odontoschisma sphagni
Latitude
Longitude
5e+06
4e+06
3e+06
2e+06
3e+06
4e+06
5e+06
6e+06
7e+06
0.80
0.60
0.40
0.20

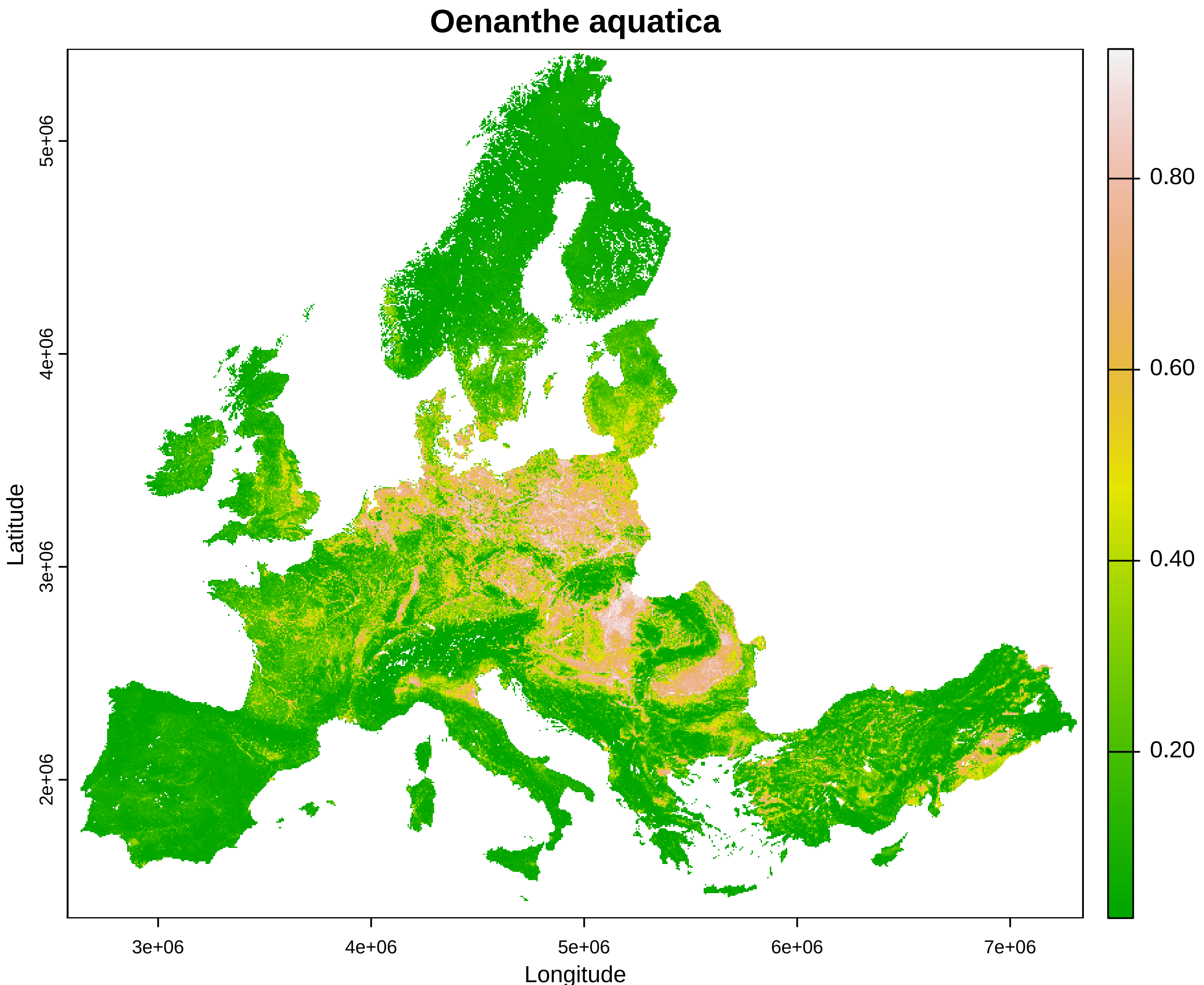
Oenanthe aquatica
Latitude
Longitude
5e+06
4e+06
3e+06
2e+06
3e+06
4e+06
5e+06
6e+06
7e+06
0.80
0.60
0.40
0.20

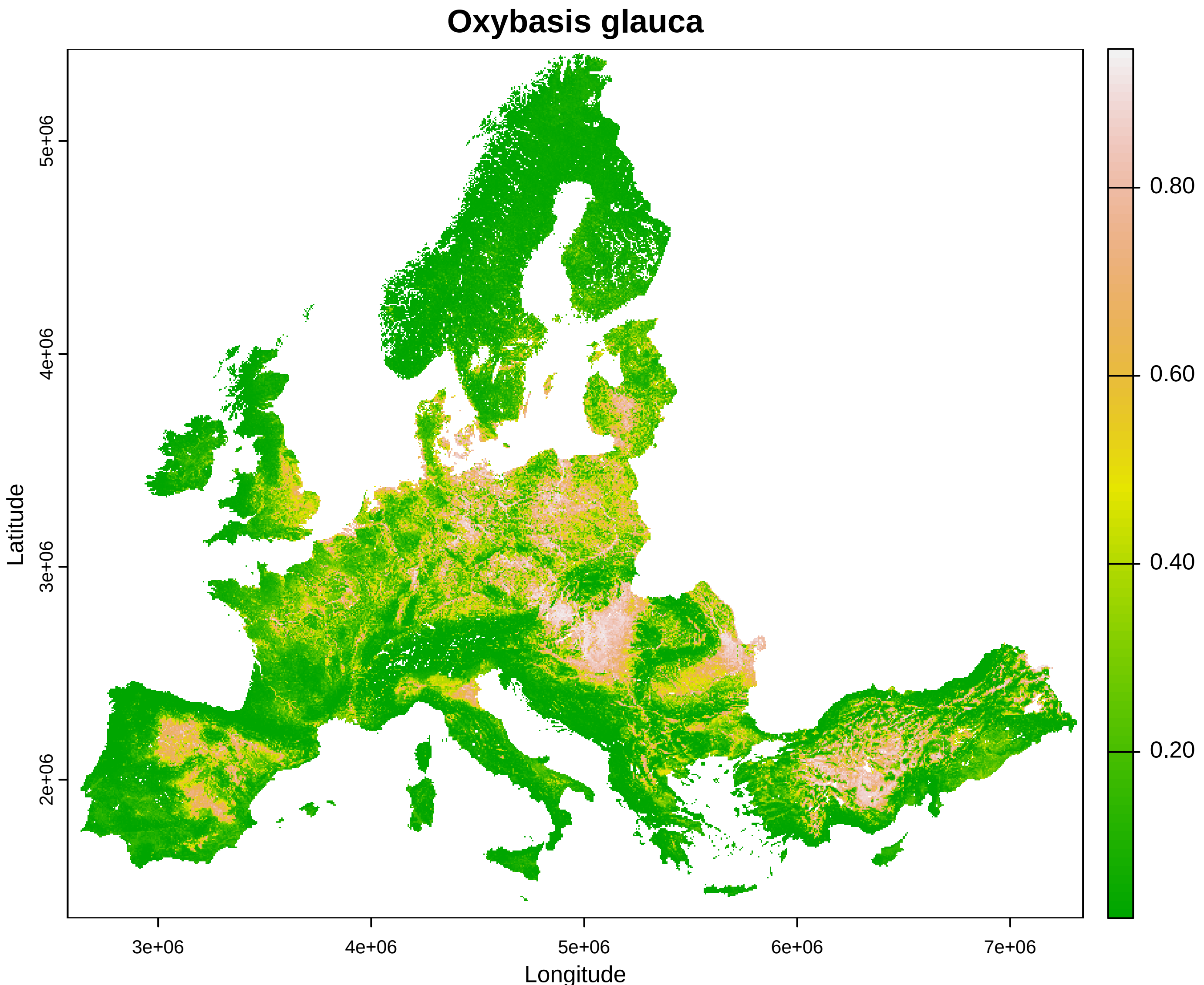
Oxybasis glauca
Latitude
Longitude
5e+06
4e+06
3e+06
2e+06
3e+06
4e+06
5e+06
6e+06
7e+06
0.80
0.60
0.40
0.20

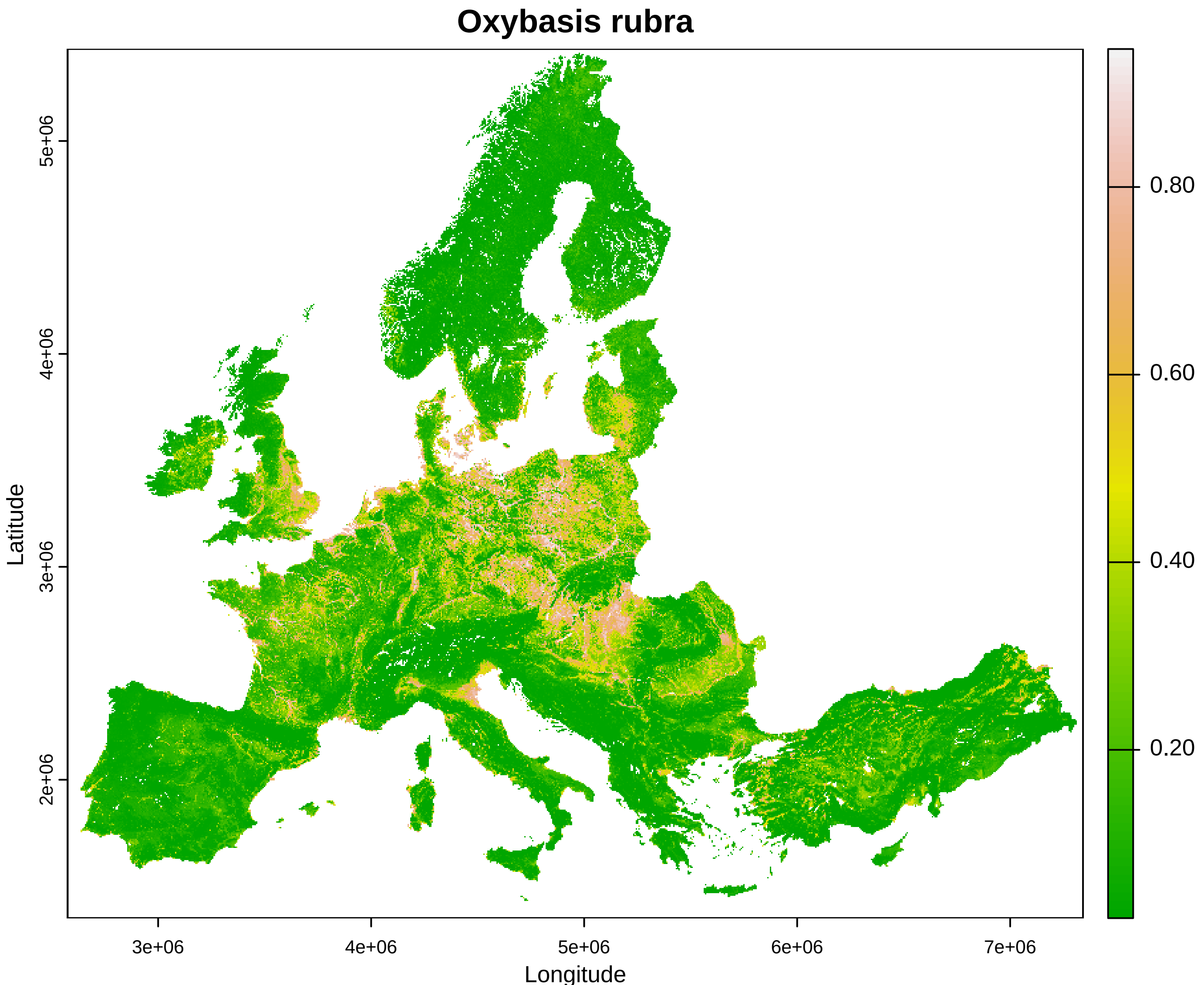

Oxybasis rubra
Latitude
Longitude
5e+06
4e+06
3e+06
2e+06
3e+06
4e+06
5e+06
6e+06
7e+06
0.80
0.60
0.40
0.20

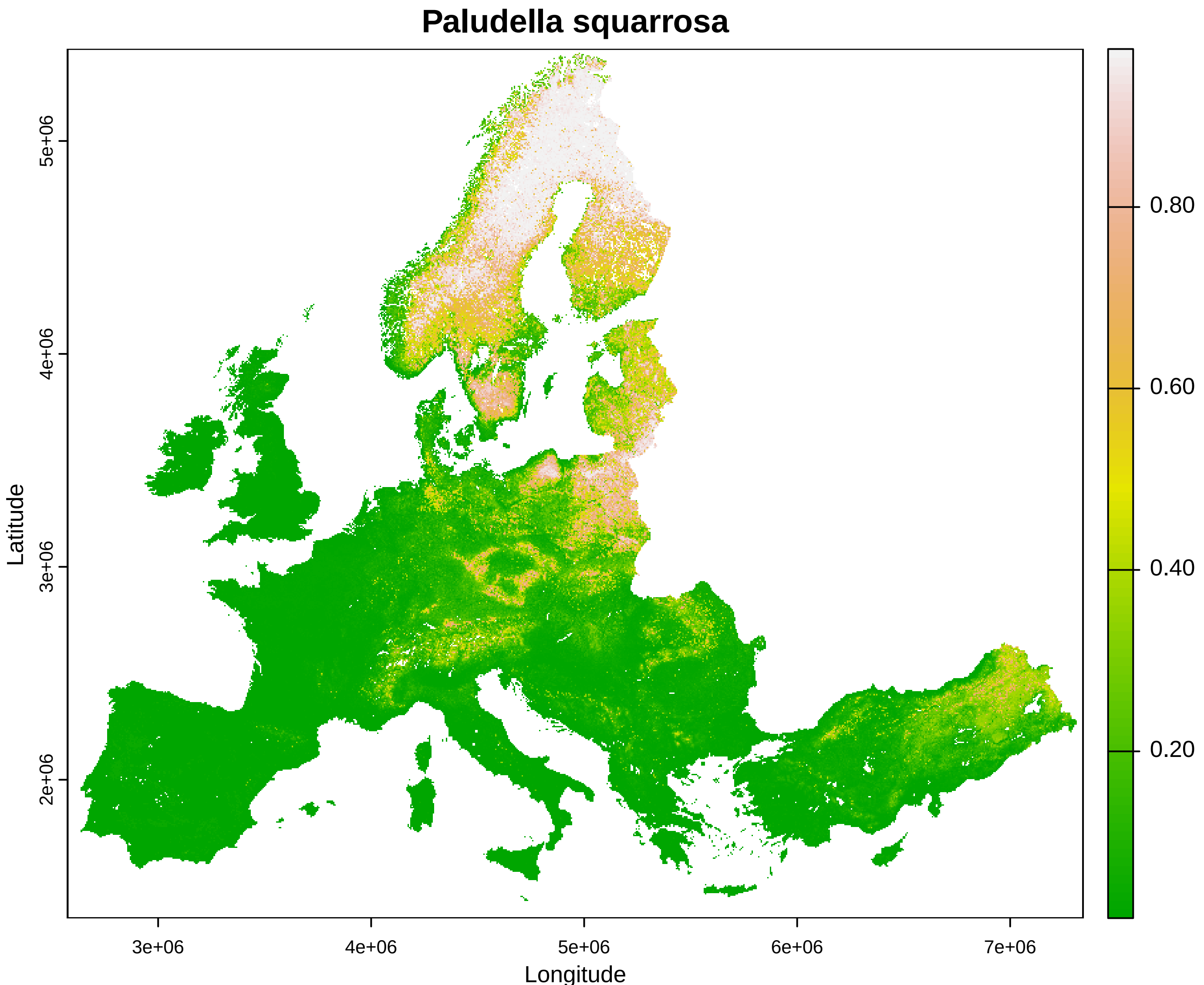
Paludella squarrosa
Latitude
Longitude
5e+06
4e+06
3e+06
2e+06
3e+06
4e+06
5e+06
6e+06
7e+06
0.80
0.60
0.40
0.20

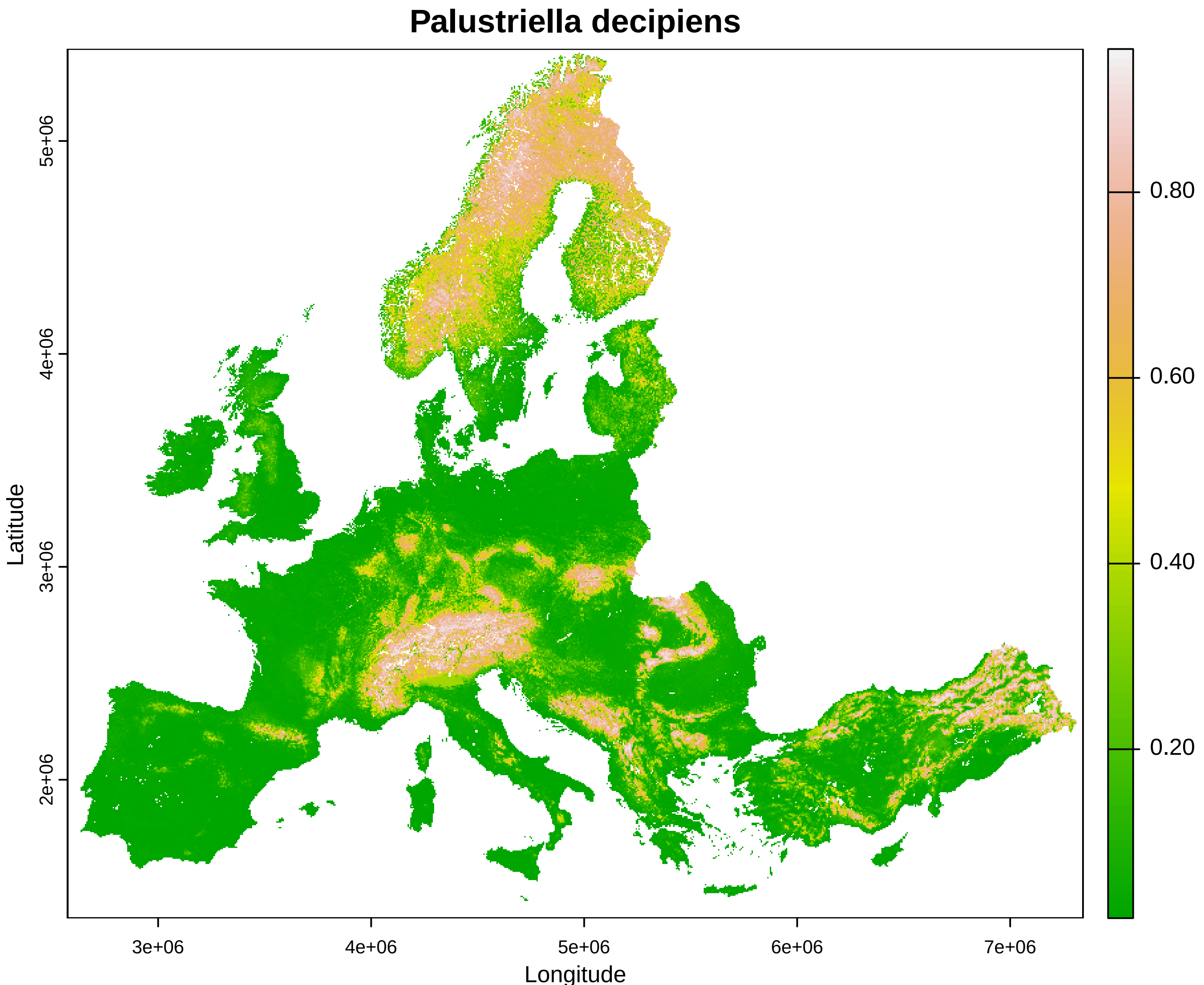
Palustriella decipiens
Latitude
5e+06
4e+06
3e+06
2e+06
Longitude
3e+06
4e+06
5e+06
6e+06
7e+06
0.80
0.60
0.40
0.20

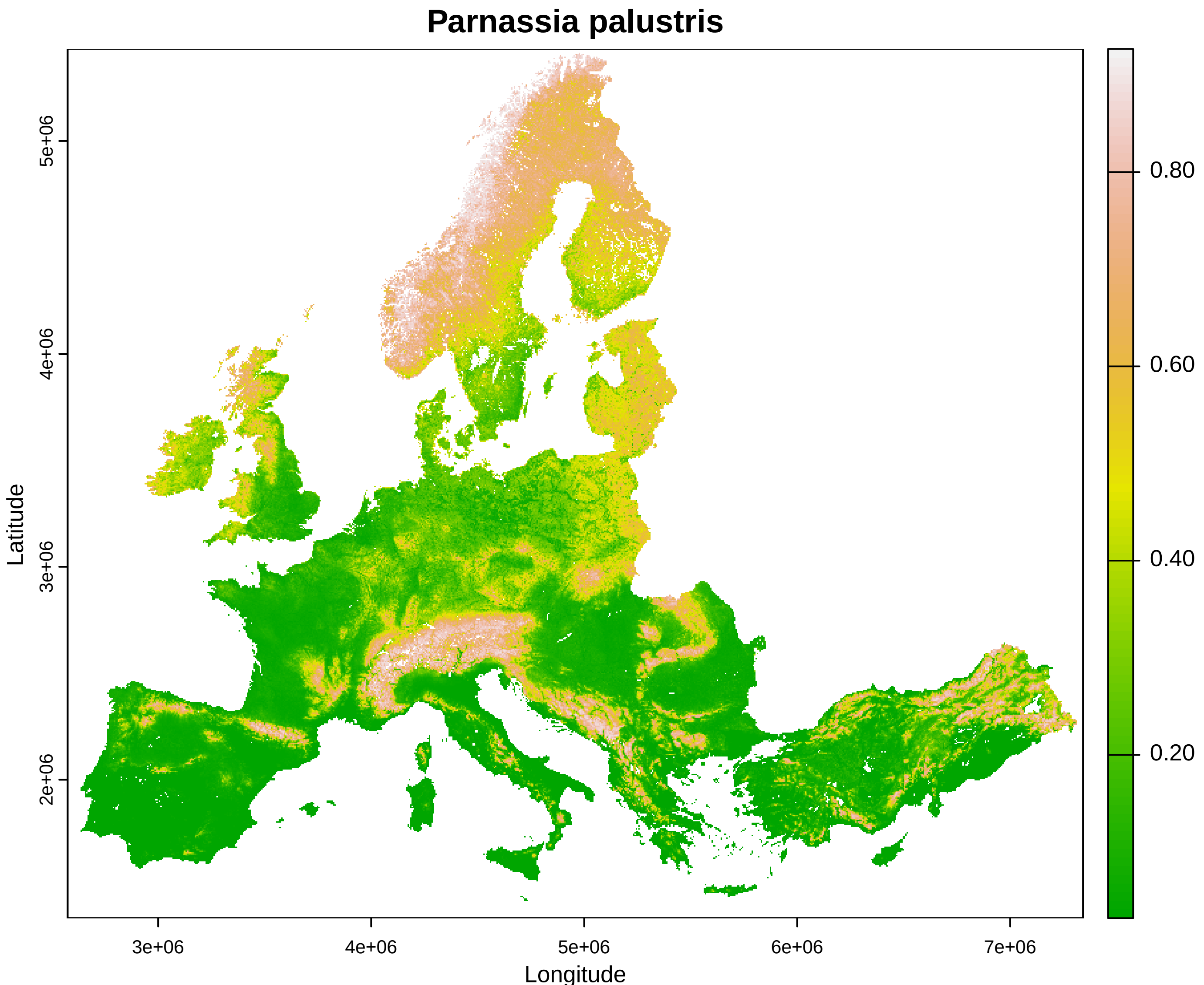
Parnassia palustris
Latitude
Longitude
5e+06
4e+06
3e+06
2e+06
3e+06
4e+06
5e+06
6e+06
7e+06
0.80
0.60
0.40
0.20

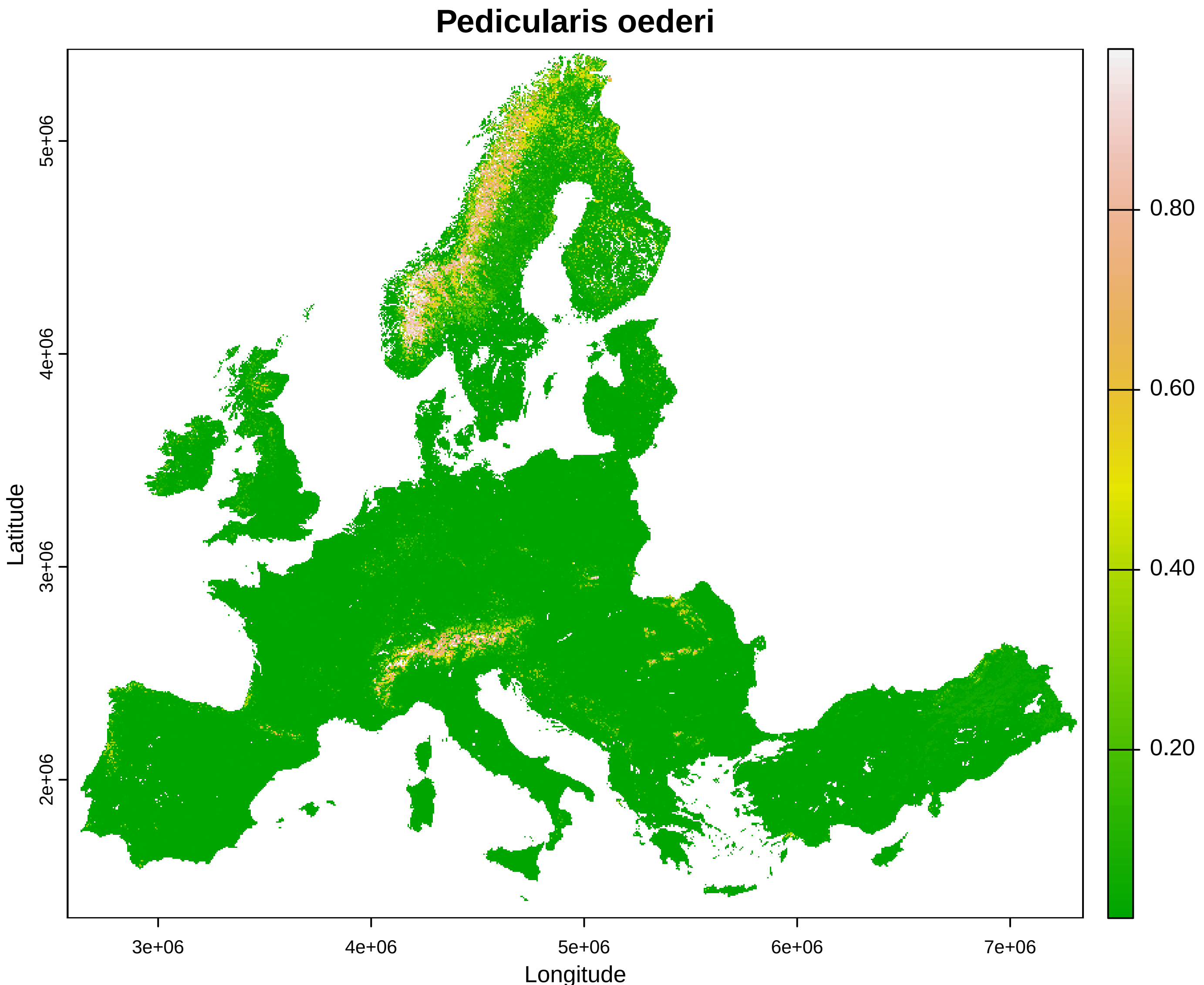
Pedicularis oederi
Latitude
Longitude
5e+06
4e+06
3e+06
2e+06
3e+06
4e+06
5e+06
6e+06
7e+06
0.80
0.60
0.40
0.20

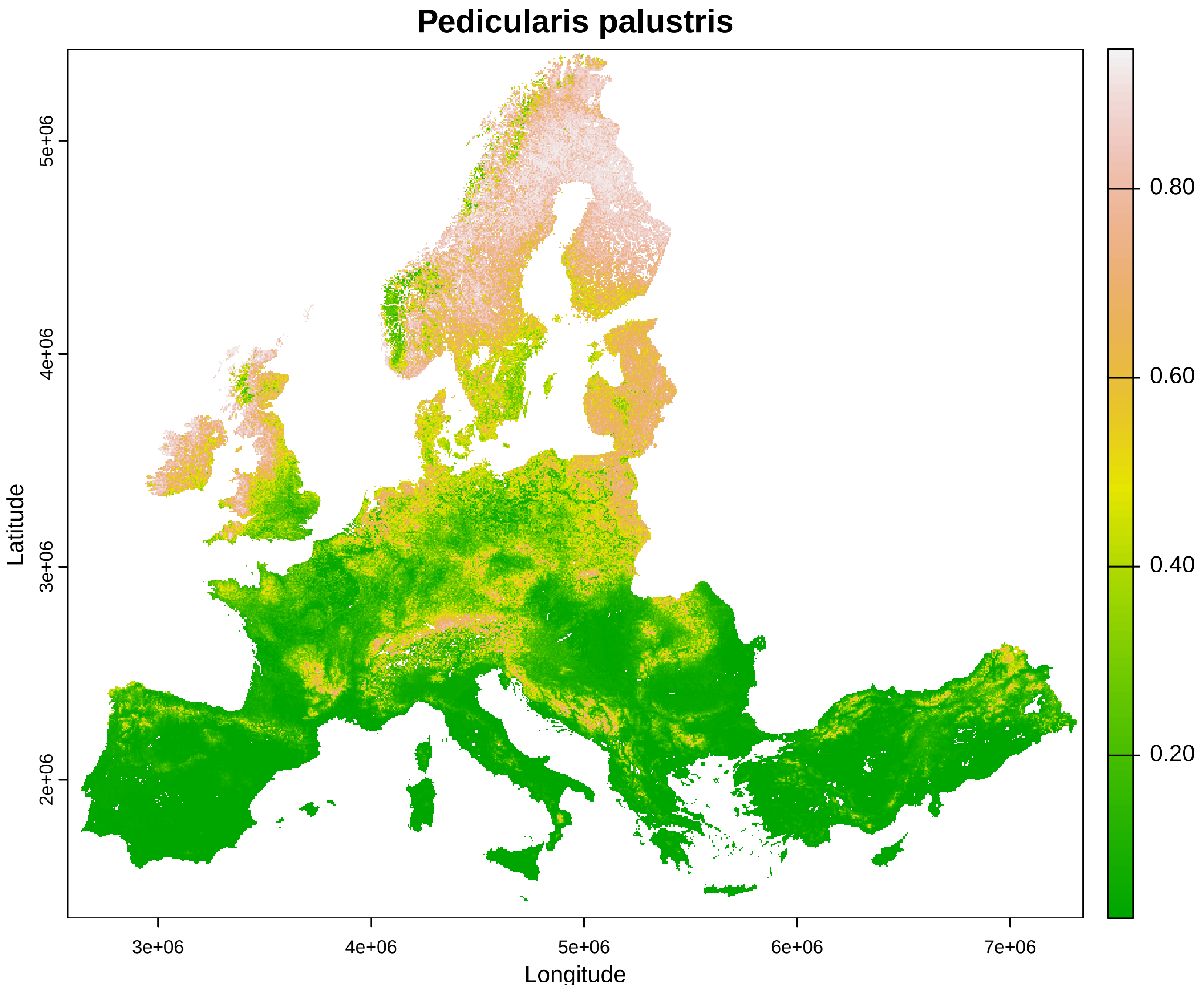
Pedicularis palustris
Latitude
Longitude
5e+06
4e+06
3e+06
2e+06
3e+06
4e+06
5e+06
6e+06
7e+06
0.80
0.60
0.40
0.20

Pedicularis sylvatica

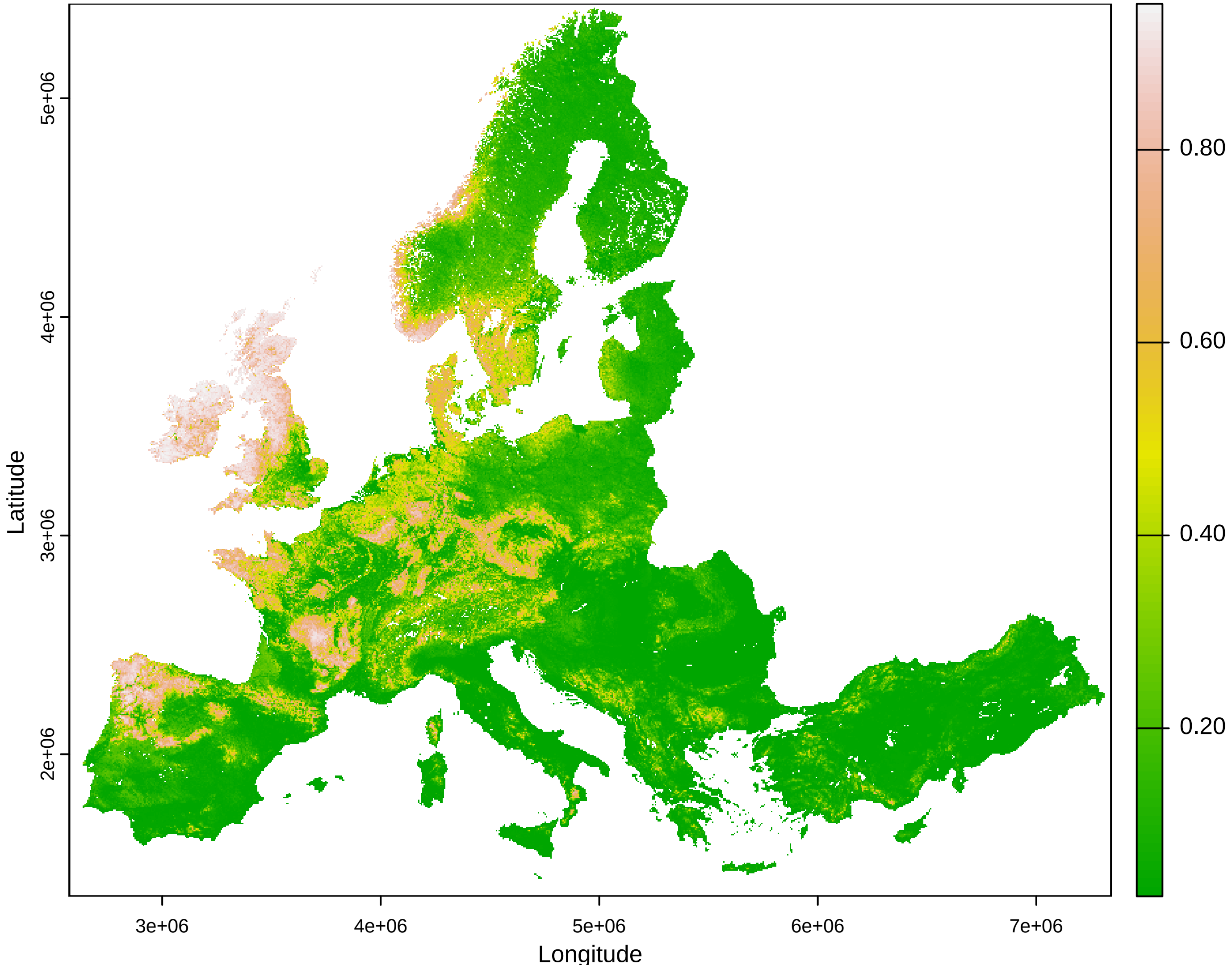

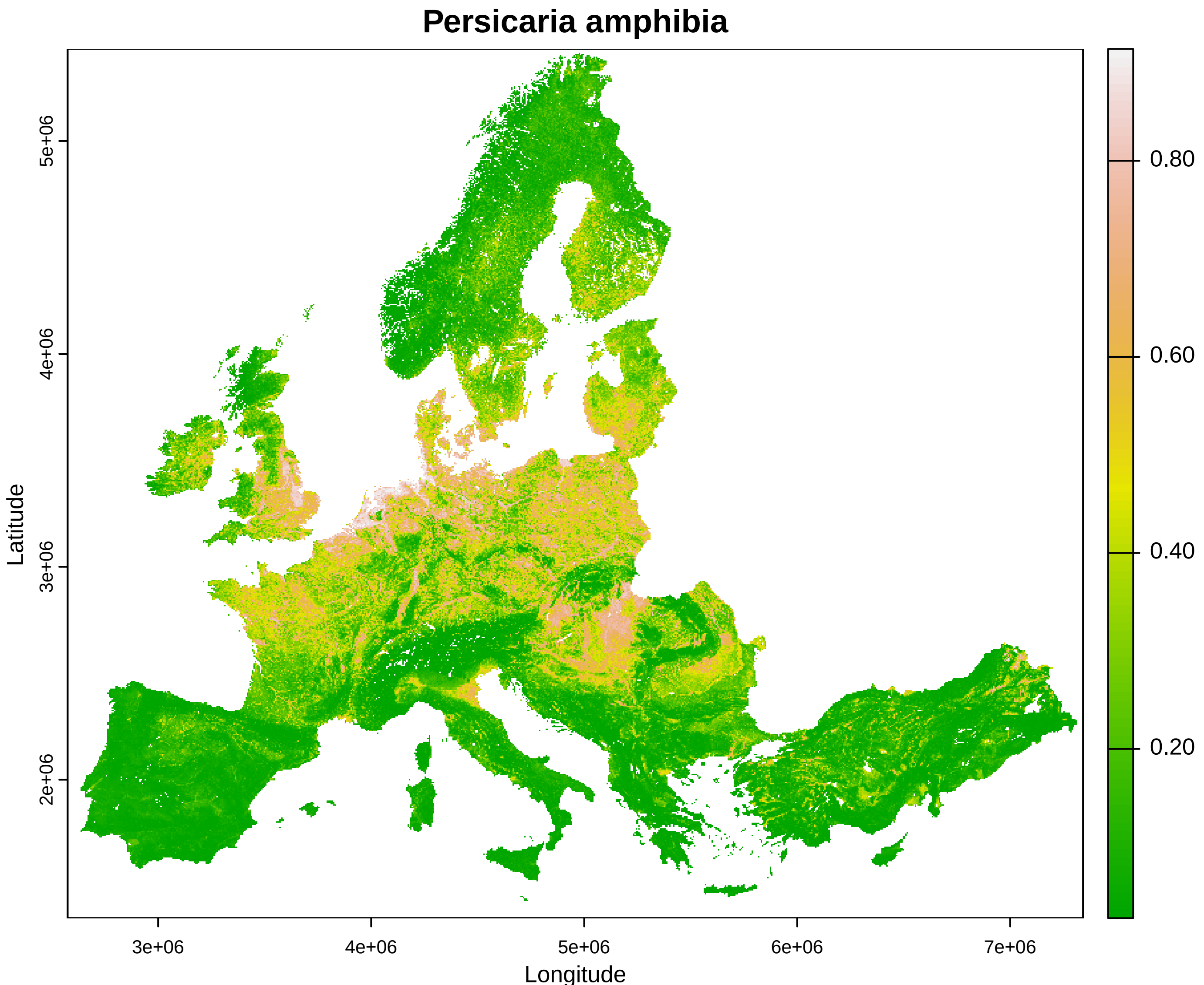
Persicaria amphibia
Latitude
5e+06
4e+06
3e+06
2e+06
3e+06
4e+06
5e+06
6e+06
7e+06
Longitude
0.80
0.60
0.40
0.20

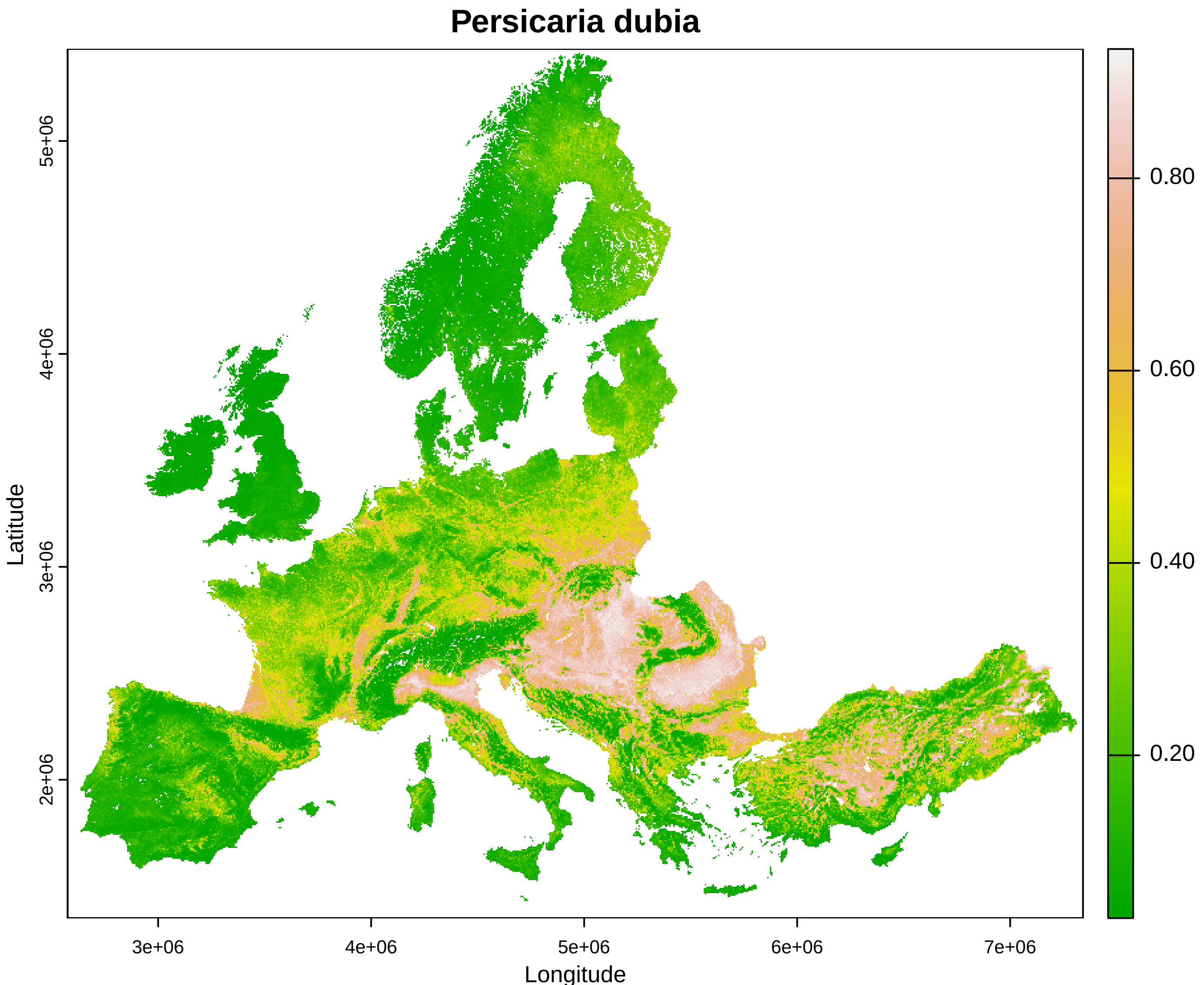
Persicaria dubia
Latitude
Longitude
5e+06
4e+06
3e+06
2e+06
3e+06
4e+06
5e+06
6e+06
7e+06
0.80
0.60
0.40
0.20

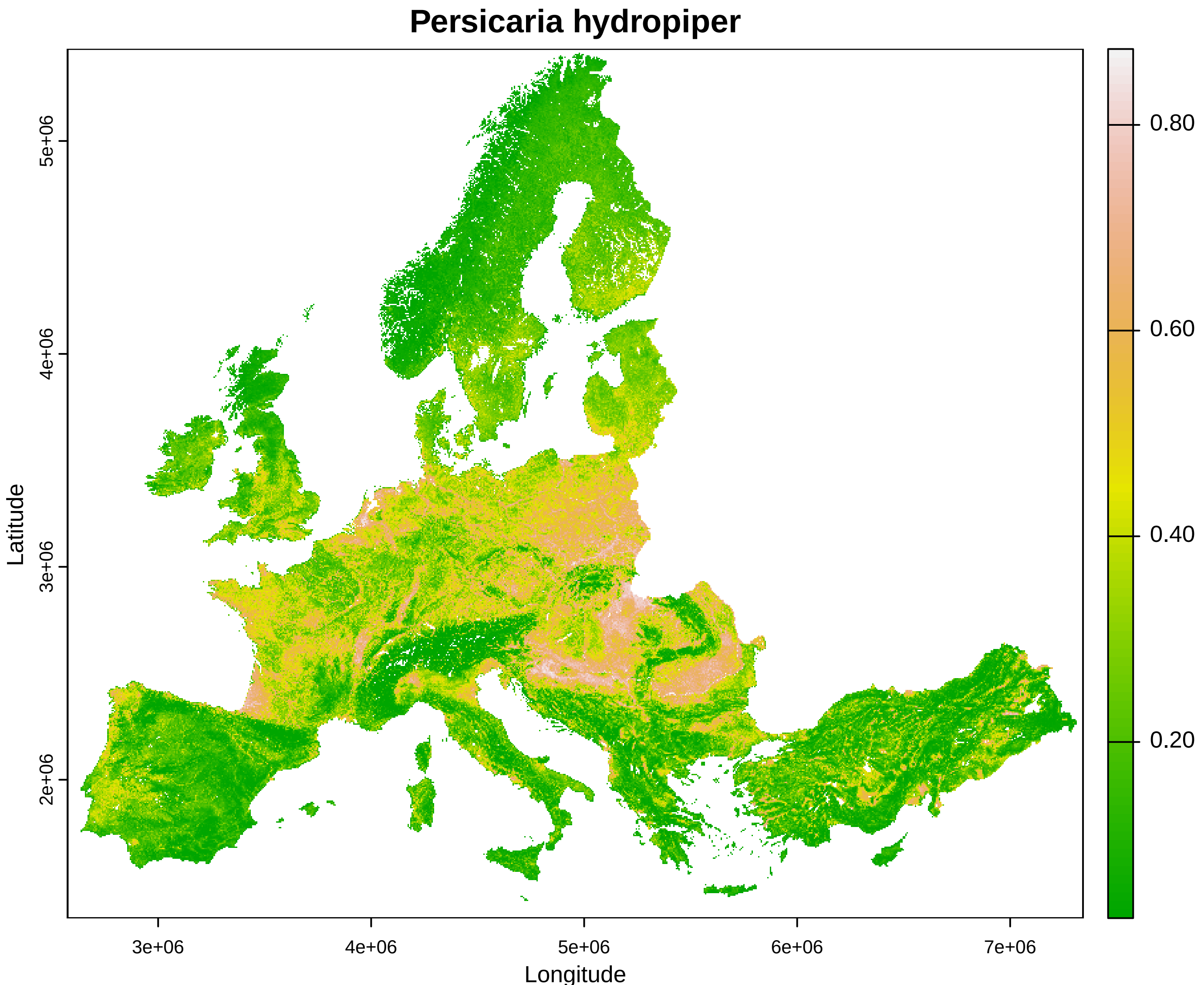

Persicaria hydropiper
Latitude
Longitude
5e+06
4e+06
3e+06
2e+06
3e+06
4e+06
5e+06
6e+06
7e+06
0.80
0.60
0.40
0.20

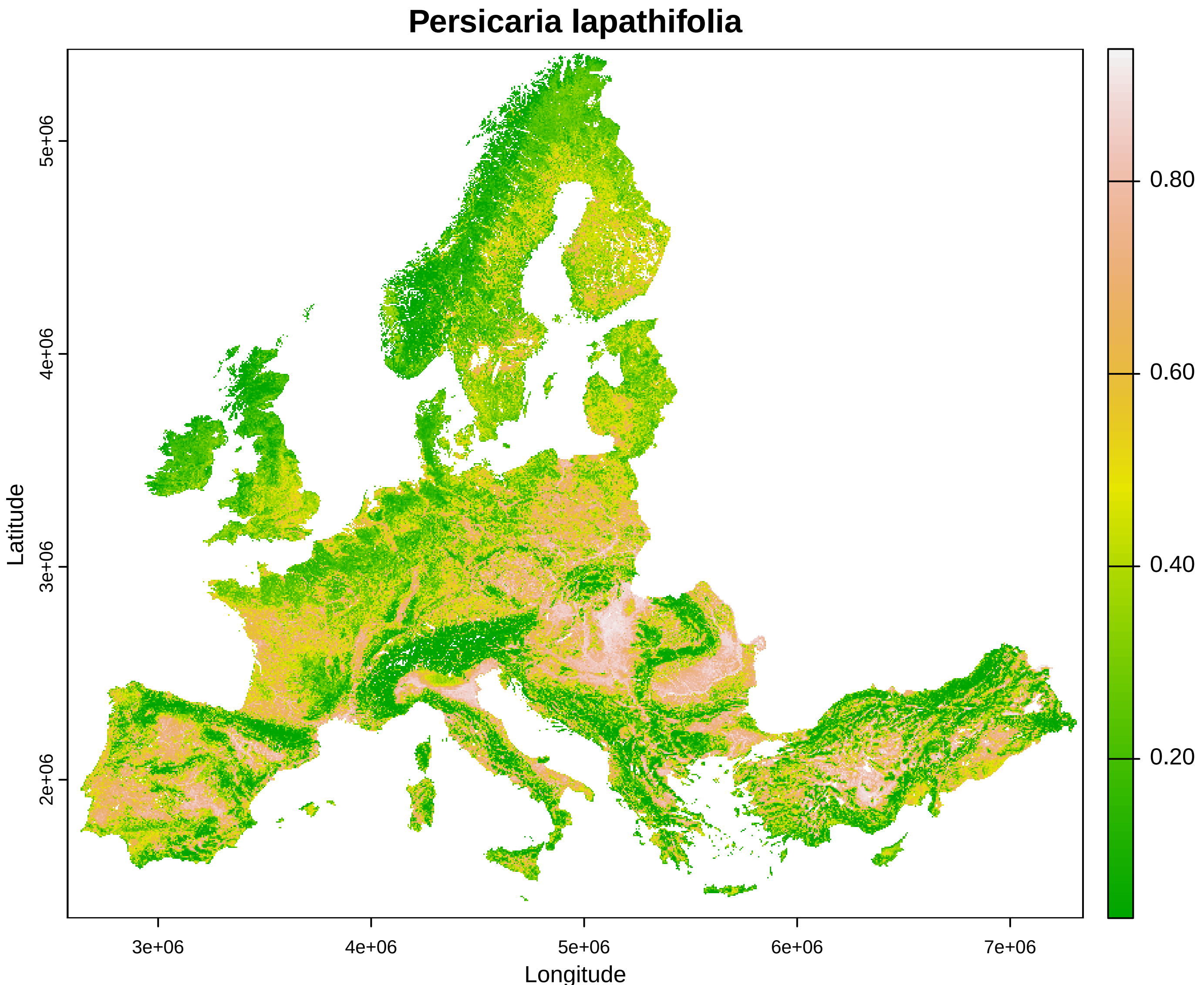

Persicaria lapathifolia
Latitude
5e+06
4e+06
3e+06
2e+06
Longitude
3e+06
4e+06
5e+06
6e+06
7e+06
0.80
0.60
0.40
0.20

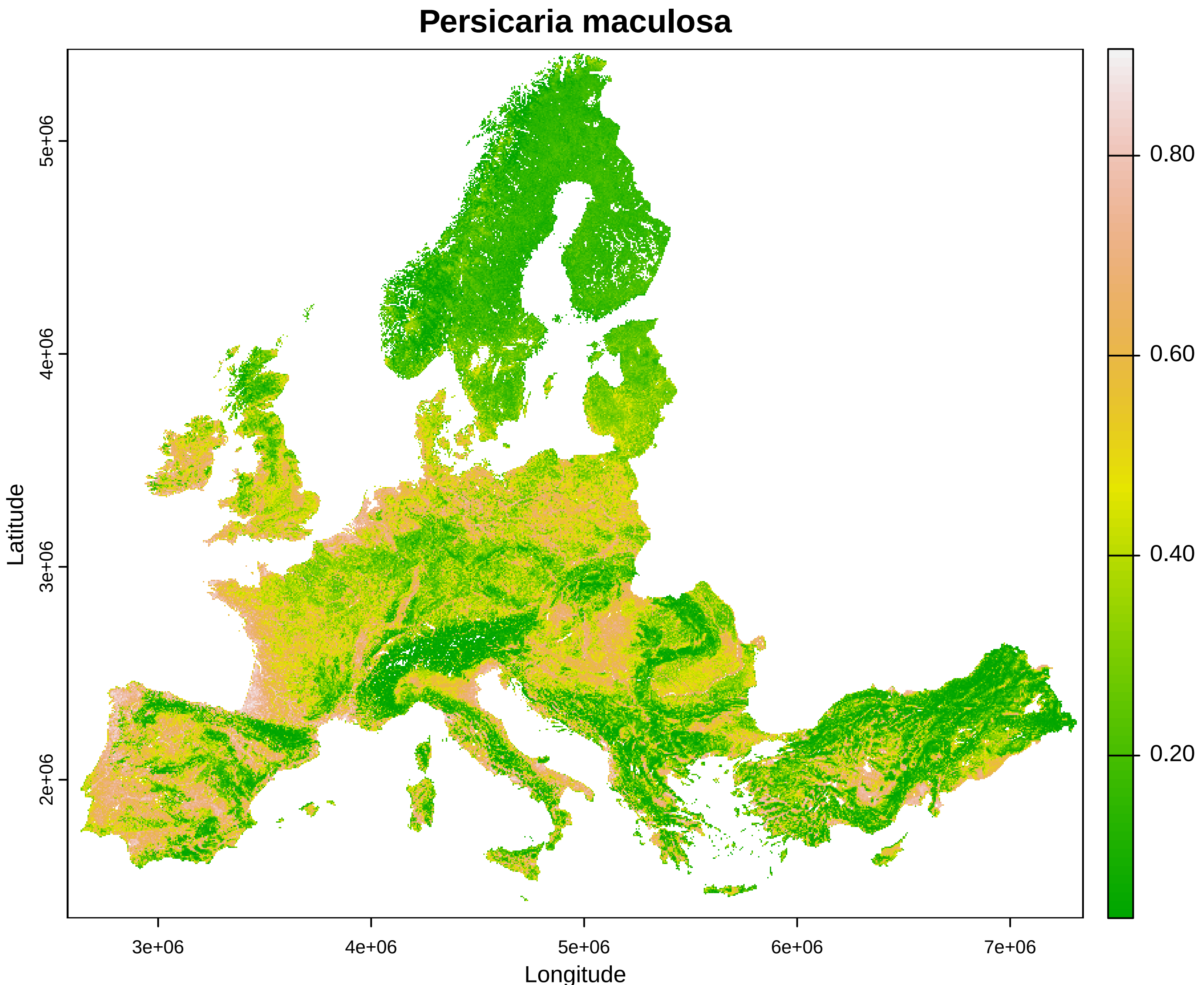

Persicaria maculosa
Latitude
Longitude
5e+06
4e+06
3e+06
2e+06
3e+06
4e+06
5e+06
6e+06
7e+06
0.80
0.60
0.40
0.20

Peucedanum palustre

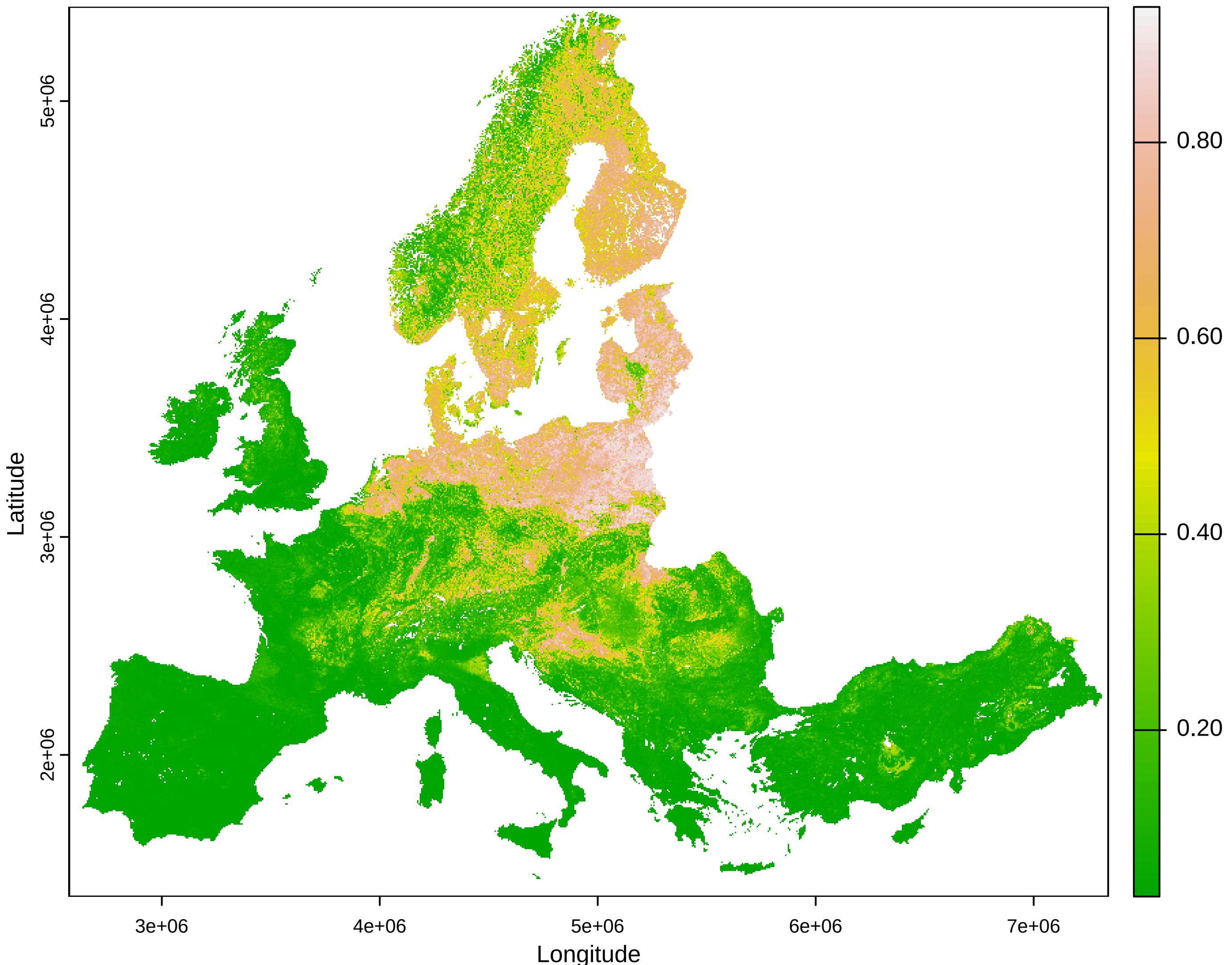

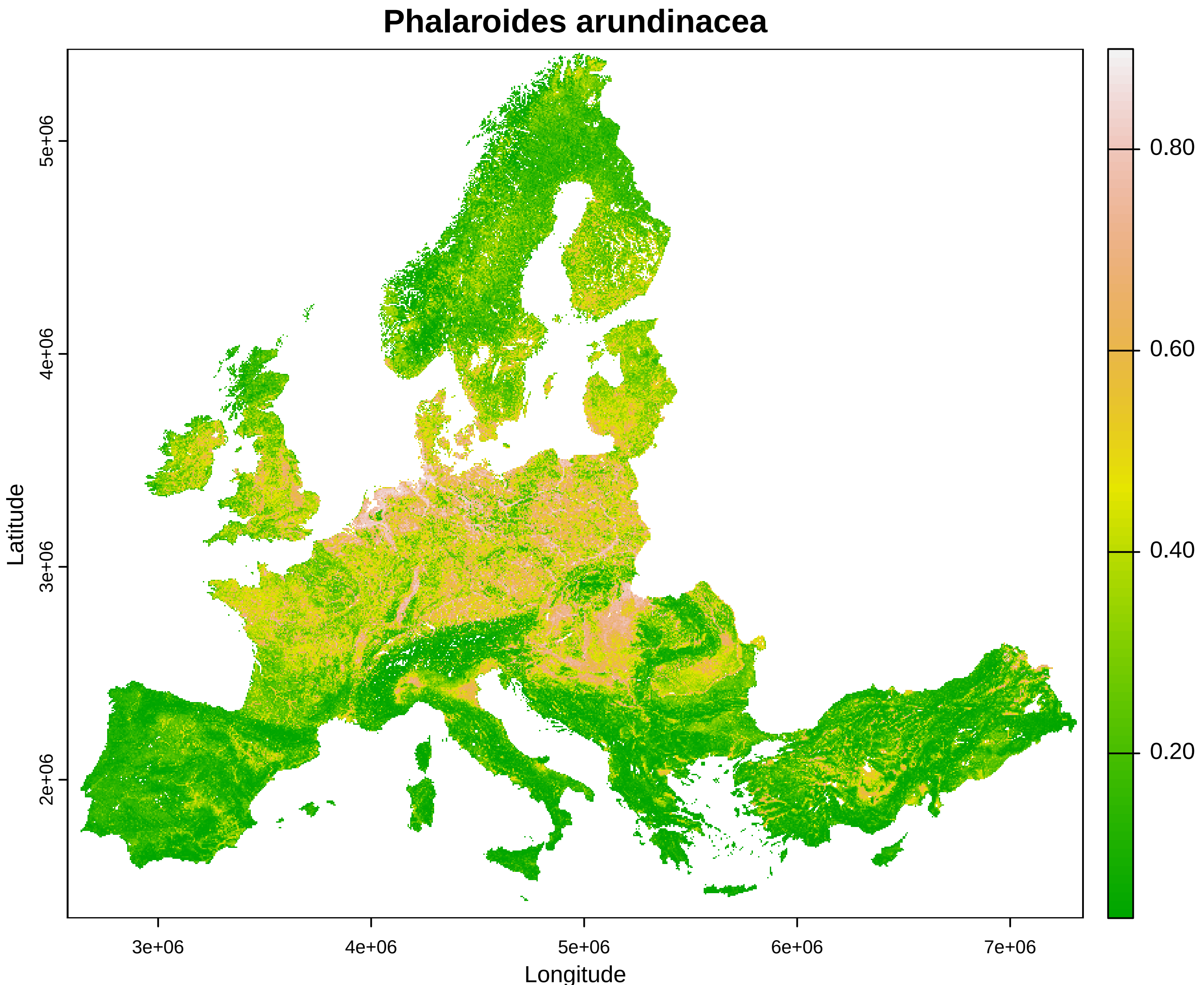

Phalaroides arundinacea
Latitude
Longitude
5e+06
4e+06
3e+06
2e+06
3e+06
4e+06
5e+06
6e+06
7e+06
0.80
0.60
0.40
0.20

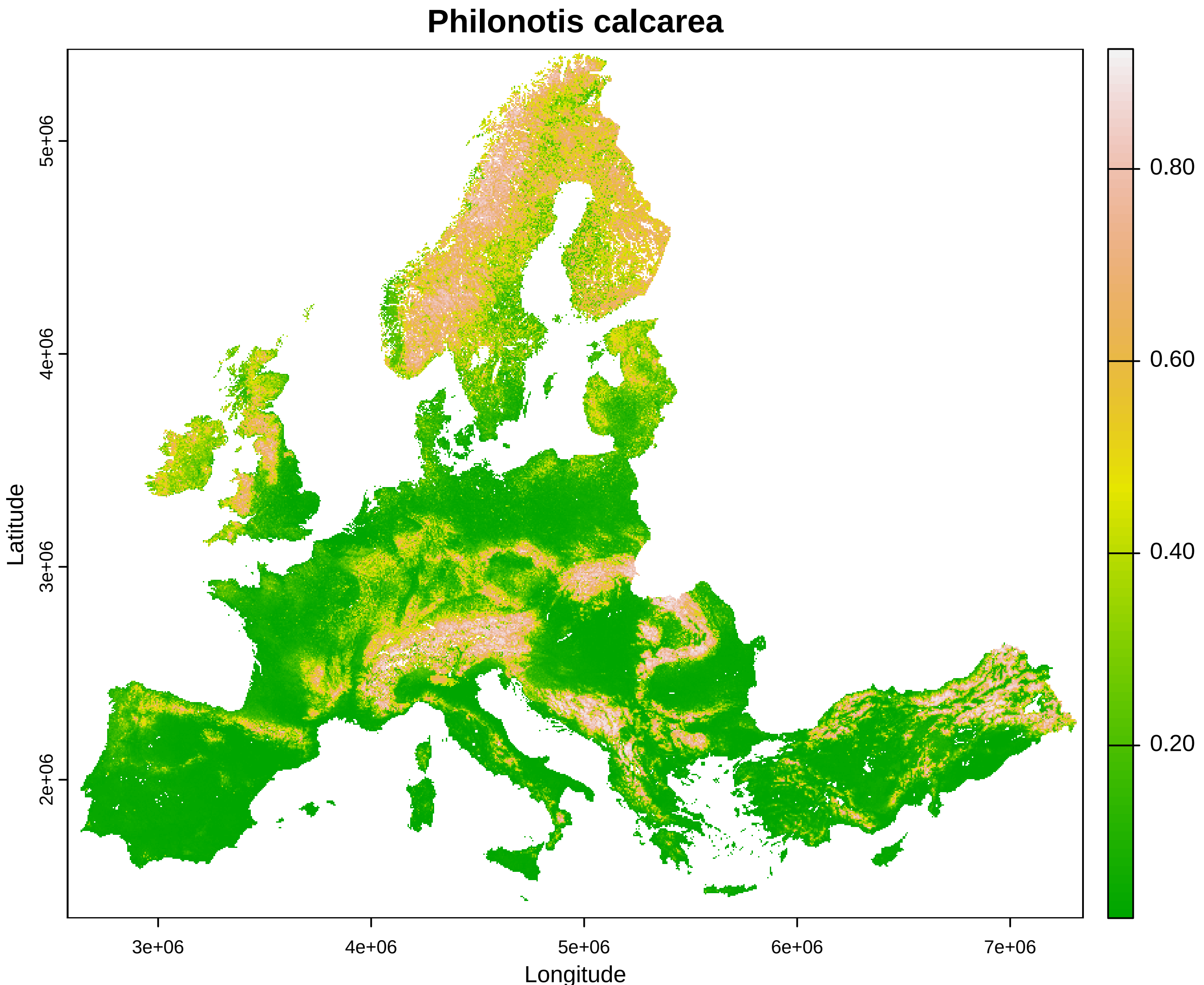

Philonotis calcarea
Latitude
Longitude
5e+06
4e+06
3e+06
2e+06
3e+06
4e+06
5e+06
6e+06
7e+06
0.80
0.60
0.40
0.20

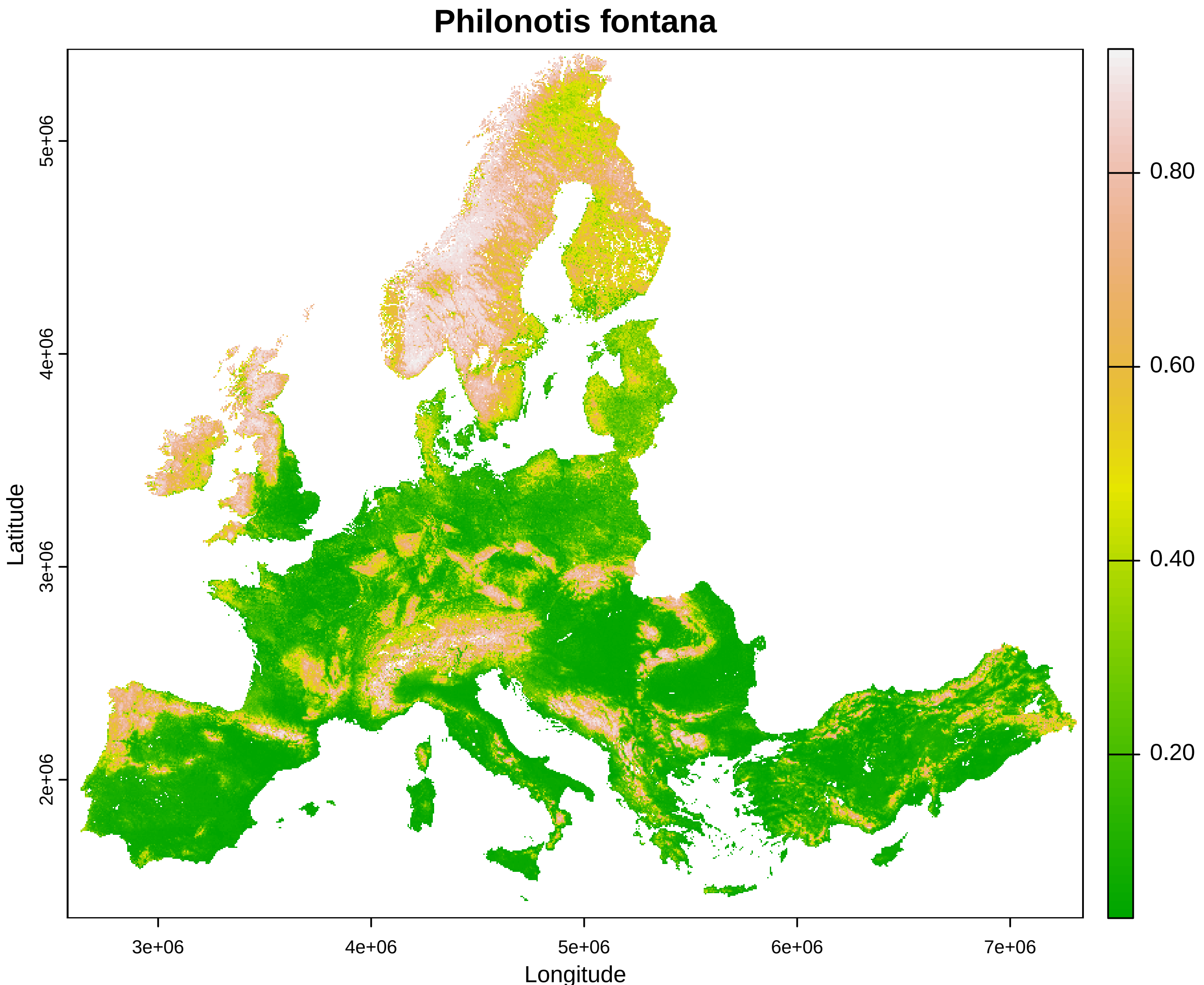

Philonotis fontana
Latitude
Longitude
5e+06
4e+06
3e+06
2e+06
3e+06
4e+06
5e+06
6e+06
7e+06
0.80
0.60
0.40
0.20

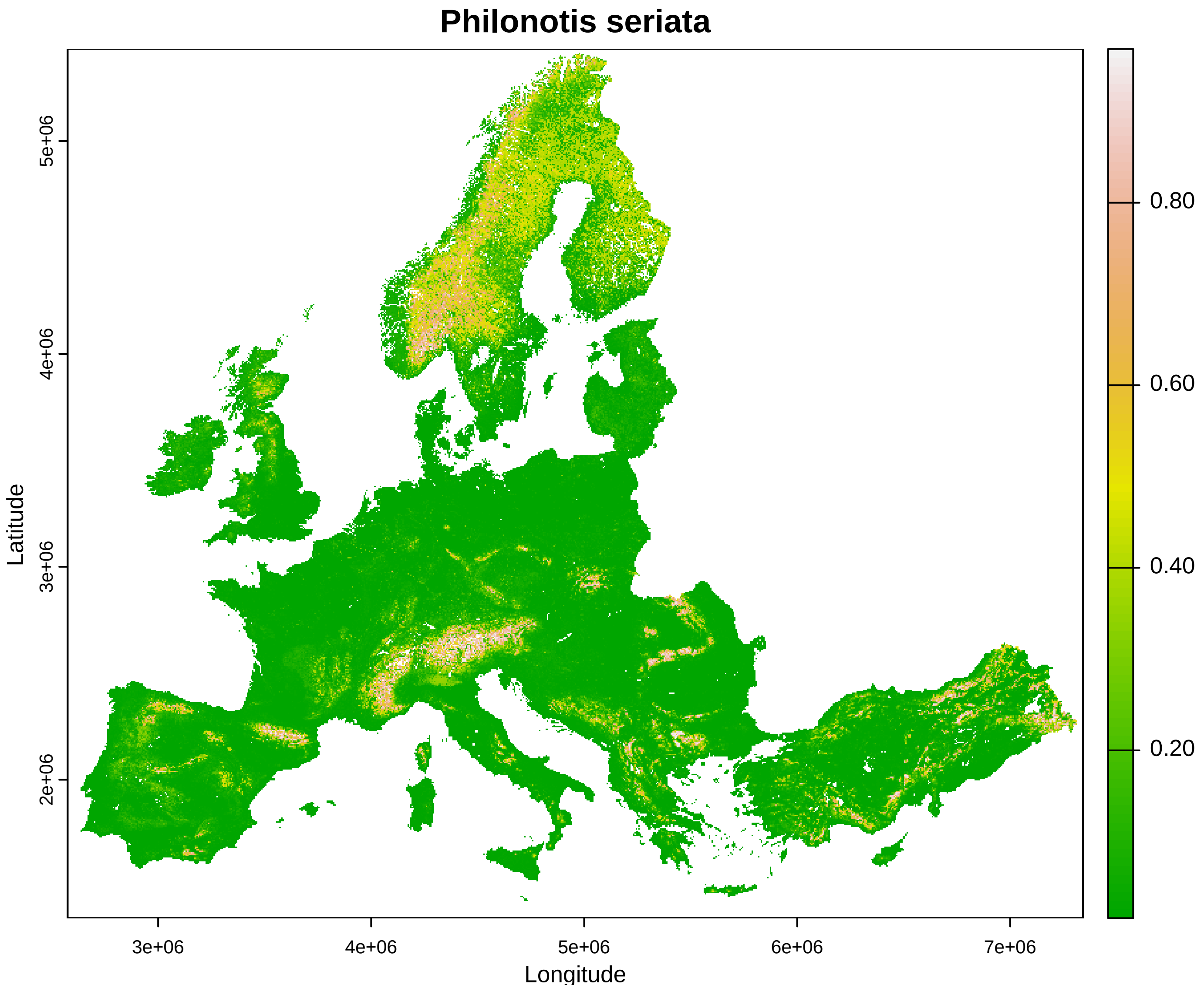
Philonotis seriata
Latitude
5e+06
4e+06
3e+06
2e+06
3e+06
4e+06
5e+06
6e+06
7e+06
Longitude
0.80
0.60
0.40
0.20

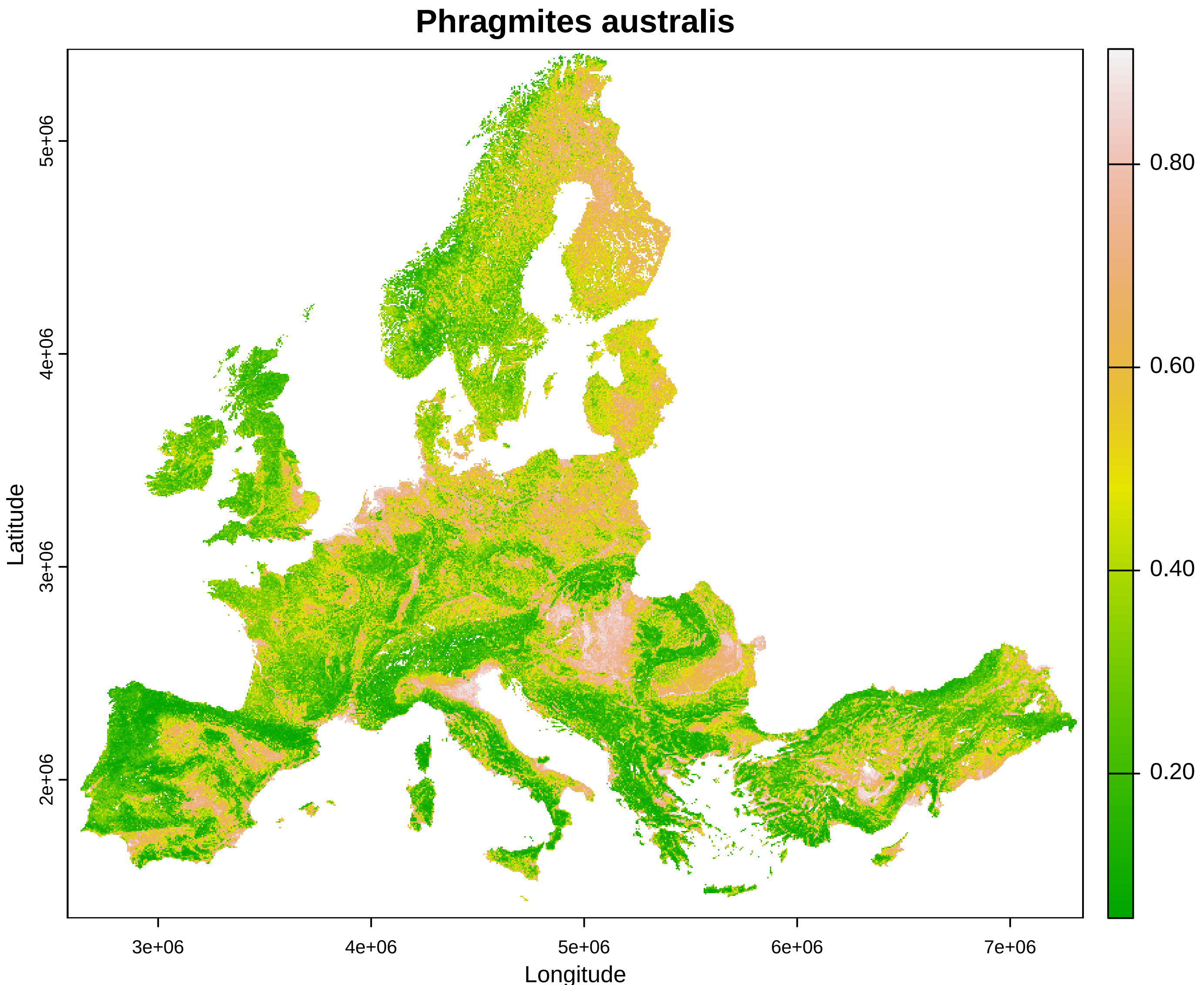

Phragmites australis
Latitude
Longitude
5e+06
4e+06
3e+06
2e+06
3e+06
4e+06
5e+06
6e+06
7e+06
0.80
0.60
0.40
0.20

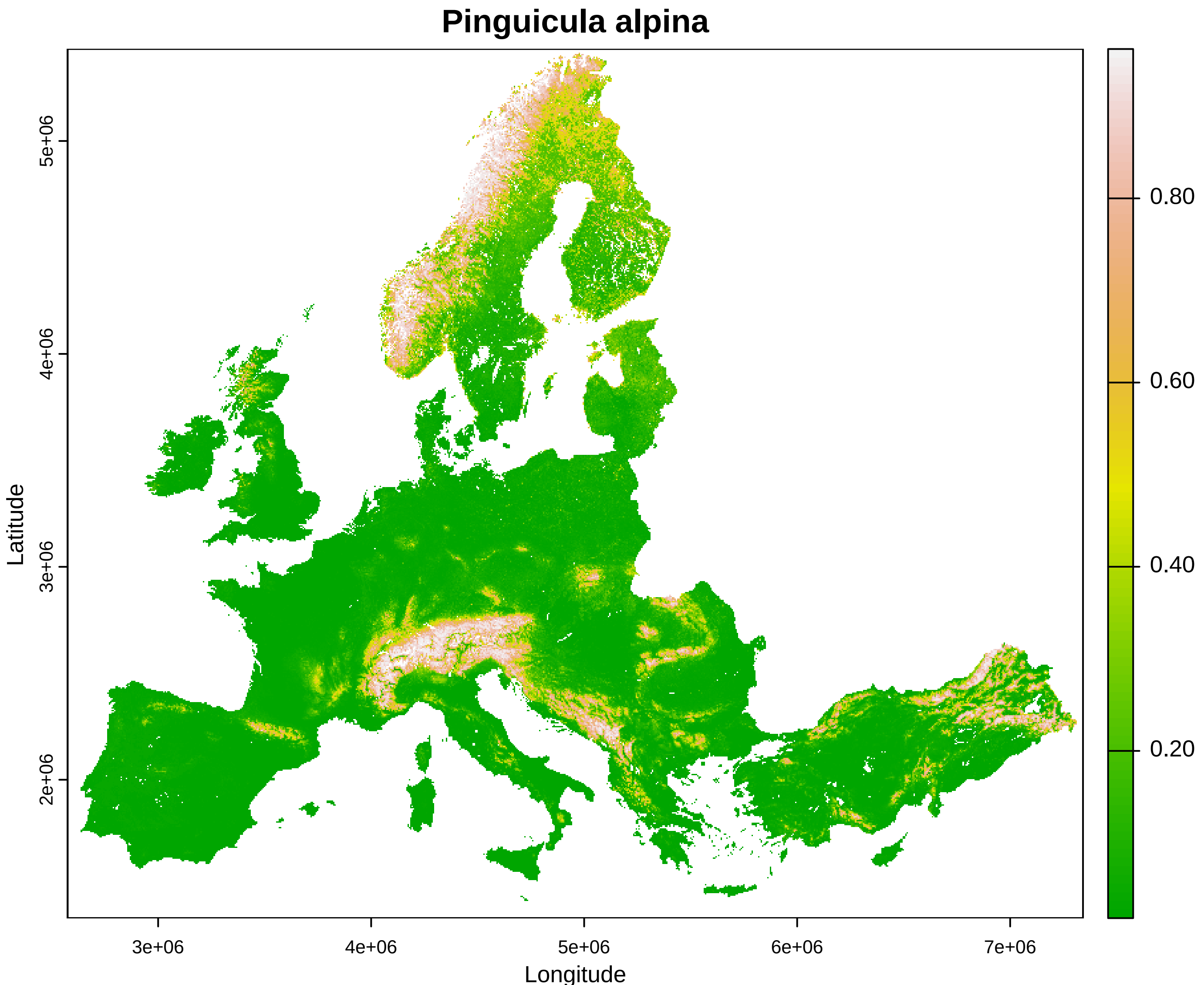

Pinguicula alpina
Latitude
Longitude
5e+06
4e+06
3e+06
2e+06
3e+06
4e+06
5e+06
6e+06
7e+06
0.80
0.60
0.40
0.20

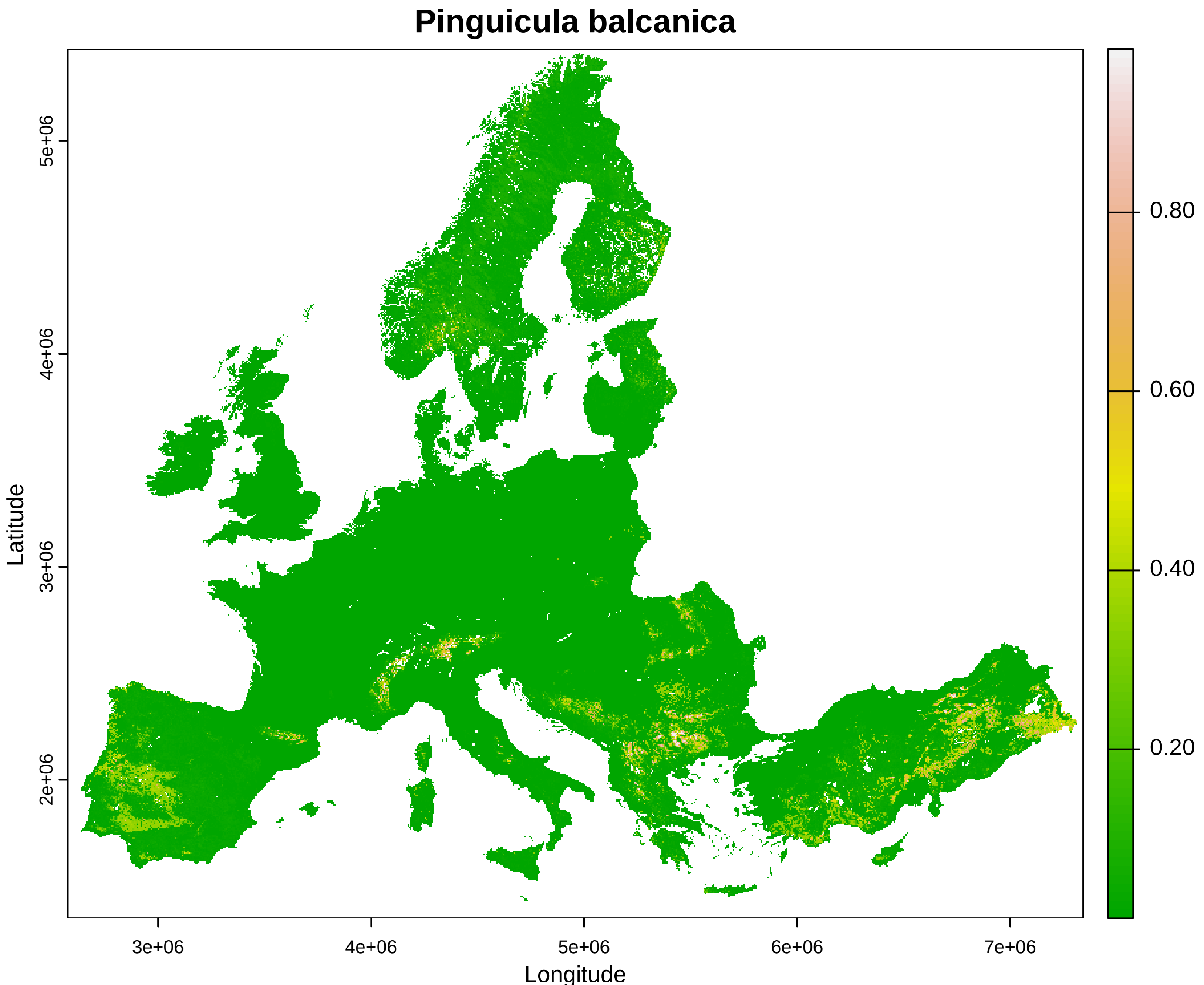
Pinguicula balcanica
Latitude
Longitude
5e+06
4e+06
3e+06
2e+06
3e+06
4e+06
5e+06
6e+06
7e+06
0.80
0.60
0.40
0.20

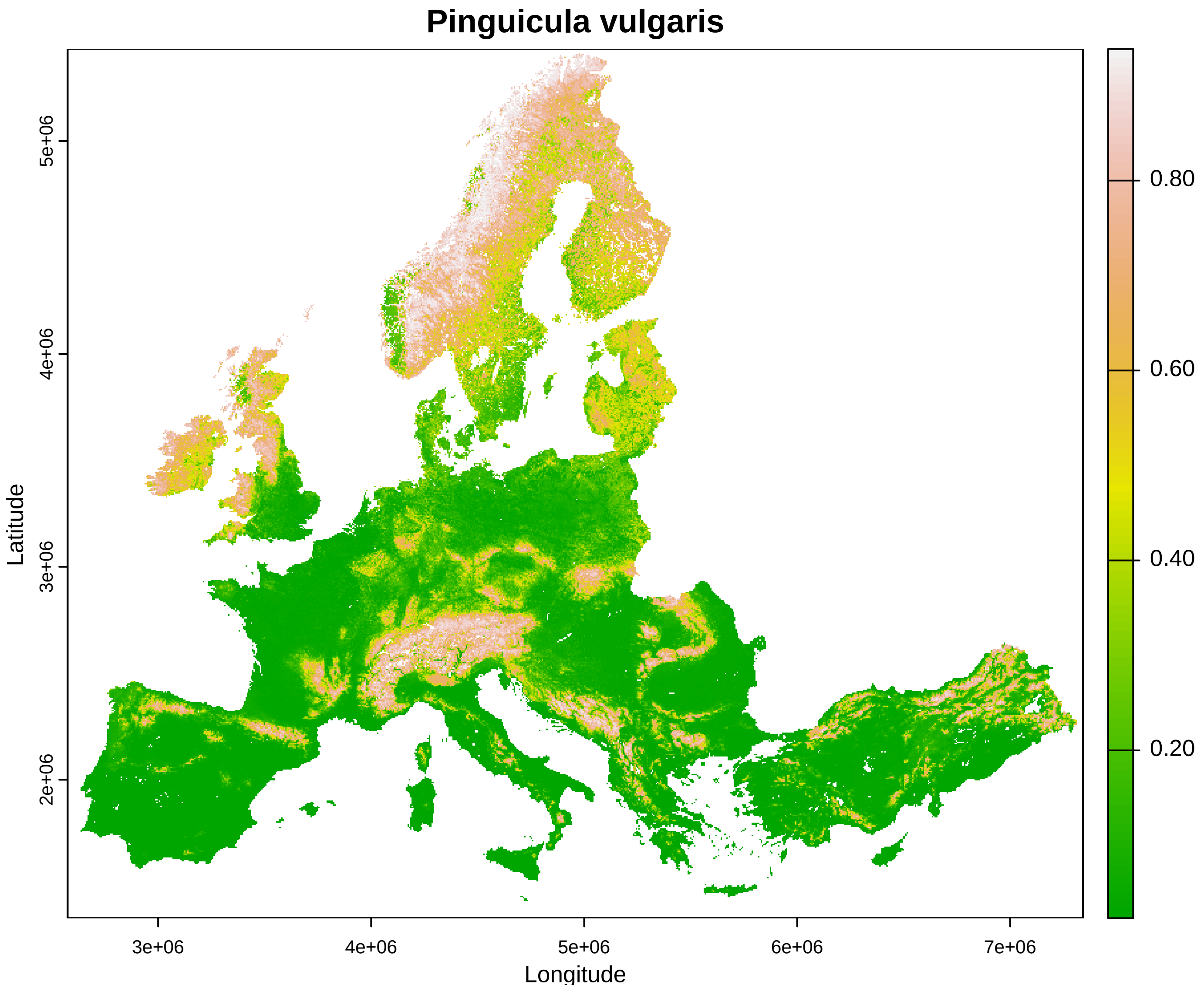

Pinguicula vulgaris
Latitude
Longitude
5e+06
4e+06
3e+06
2e+06
3e+06
4e+06
5e+06
6e+06
7e+06
0.80
0.60
0.40
0.20

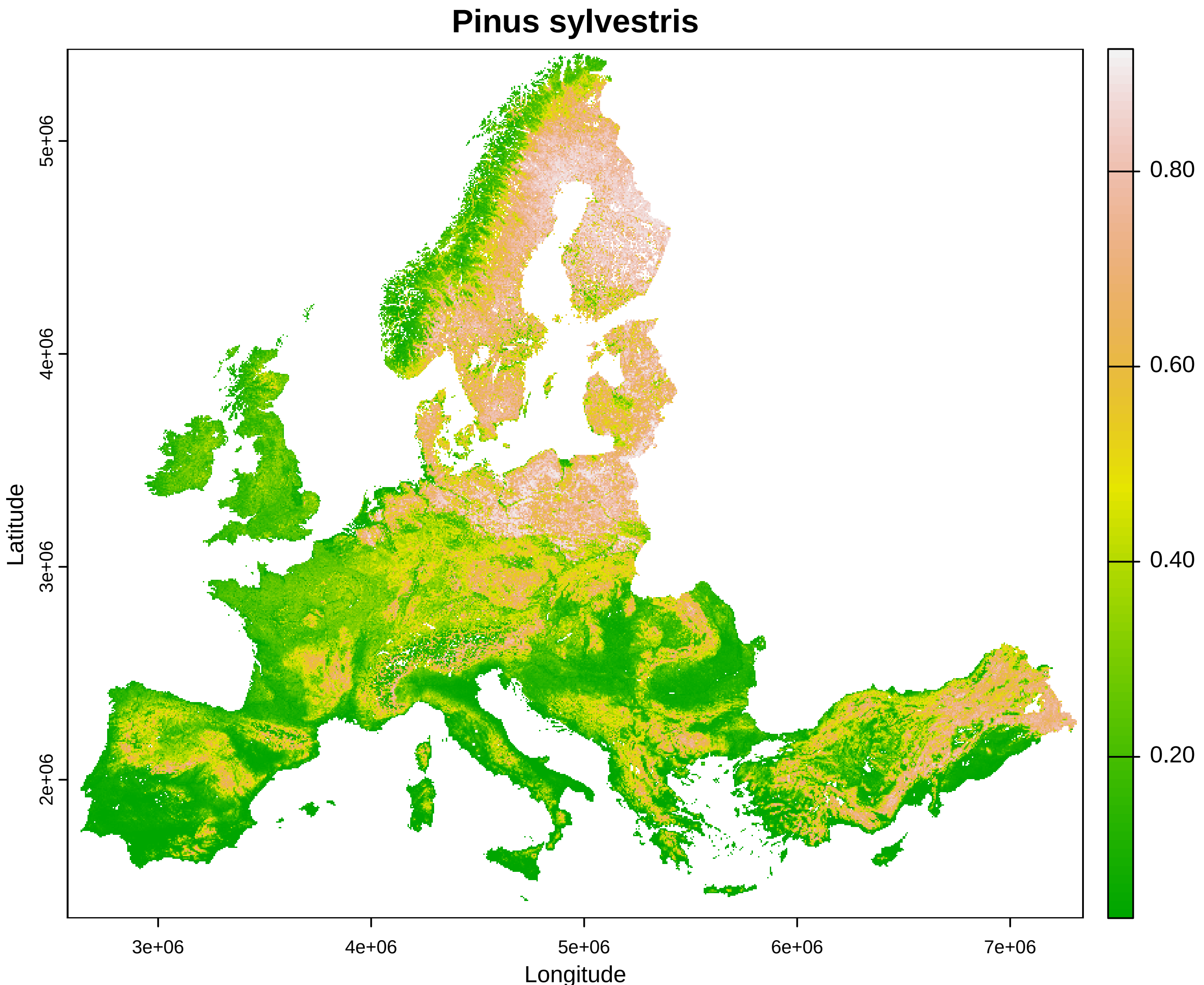
Pinus sylvestris
Latitude
5e+06
4e+06
3e+06
2e+06
3e+06
4e+06
5e+06
6e+06
7e+06
Longitude
0.80
0.60
0.40
0.20

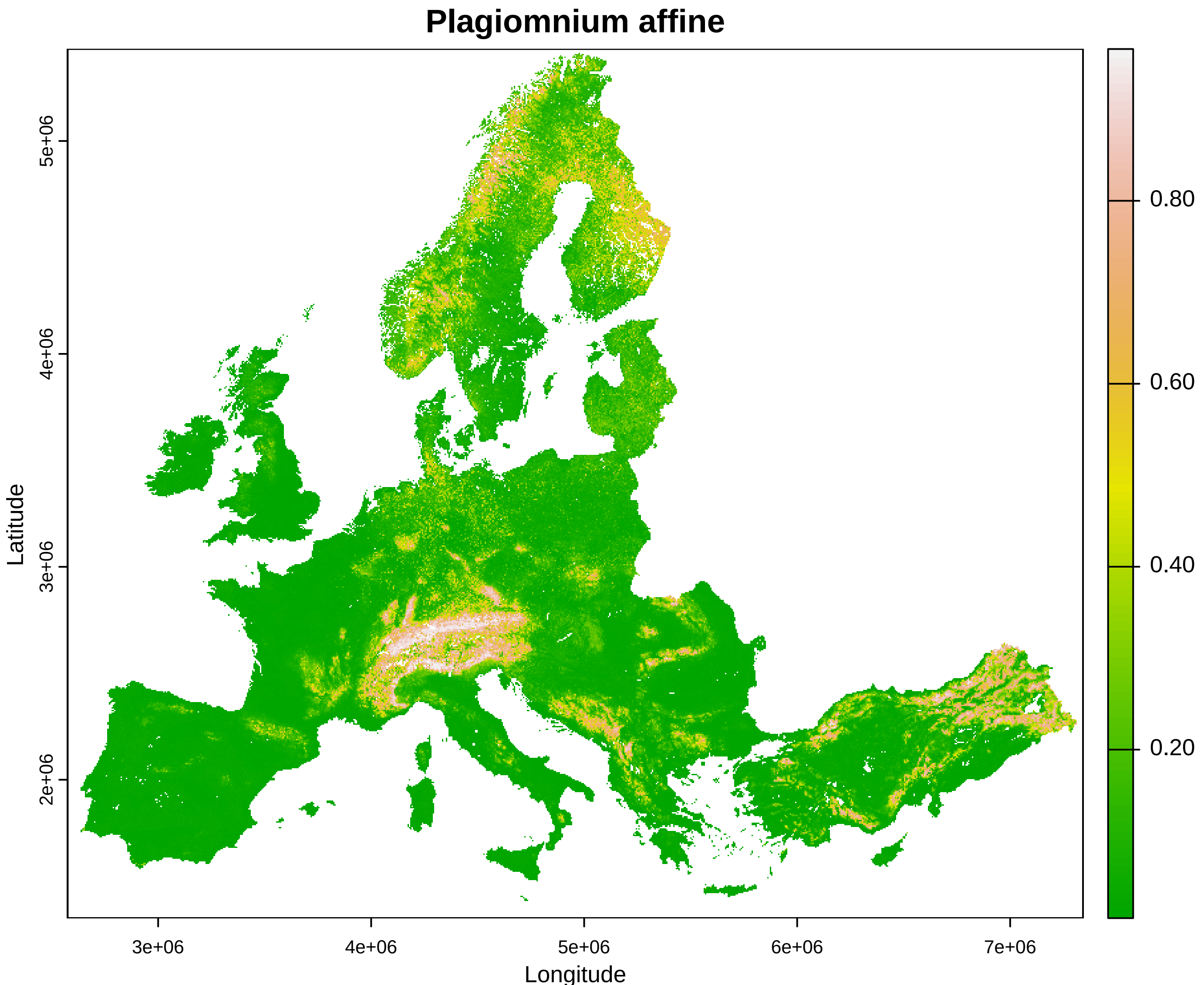

Plagiomnium affine
Latitude
Longitude
5e+06
4e+06
3e+06
2e+06
3e+06
4e+06
5e+06
6e+06
7e+06
0.80
0.60
0.40
0.20

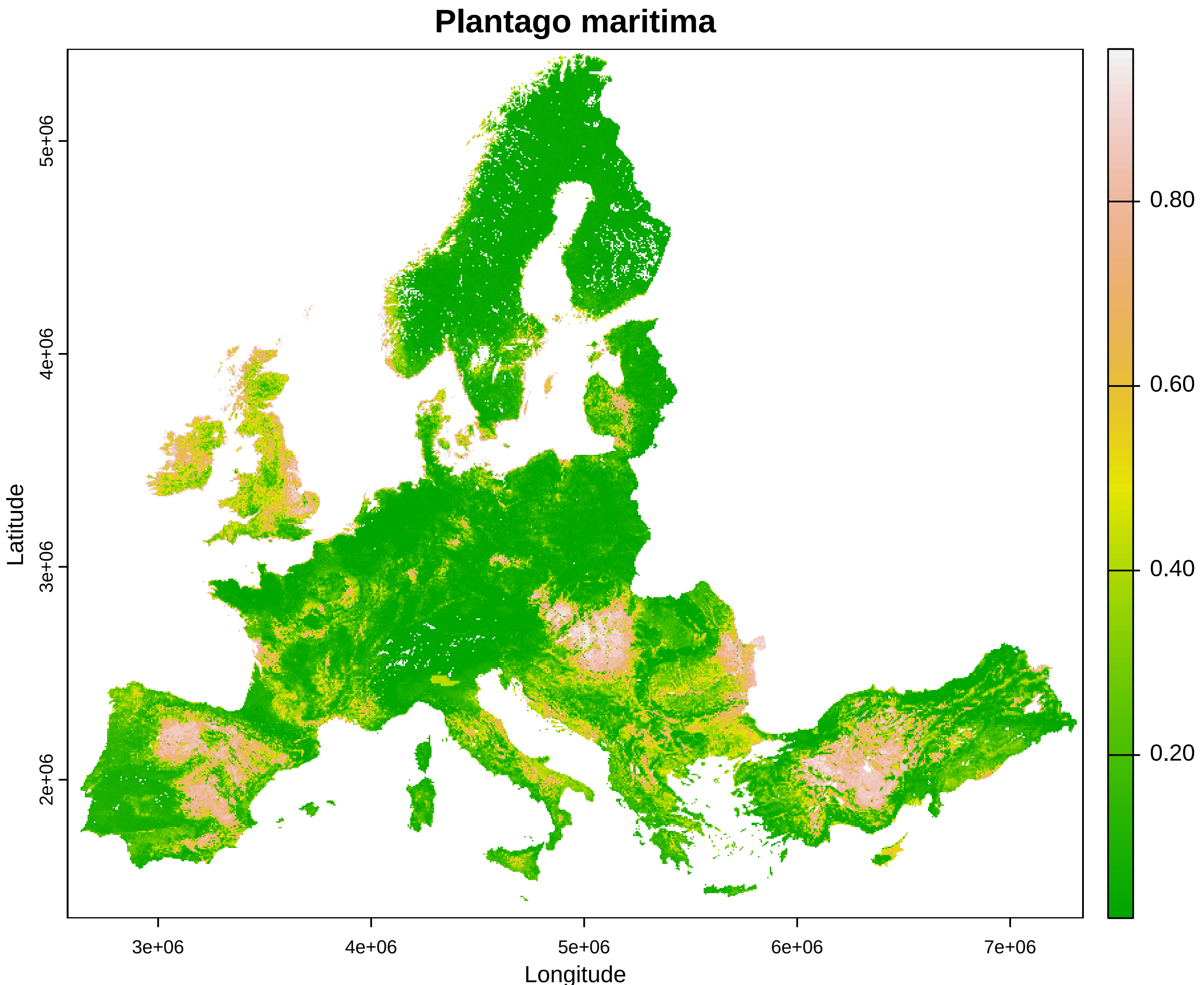
Plantago maritima
Latitude
Longitude
5e+06
4e+06
3e+06
2e+06
3e+06
4e+06
5e+06
6e+06
7e+06
0.80
0.60
0.40
0.20

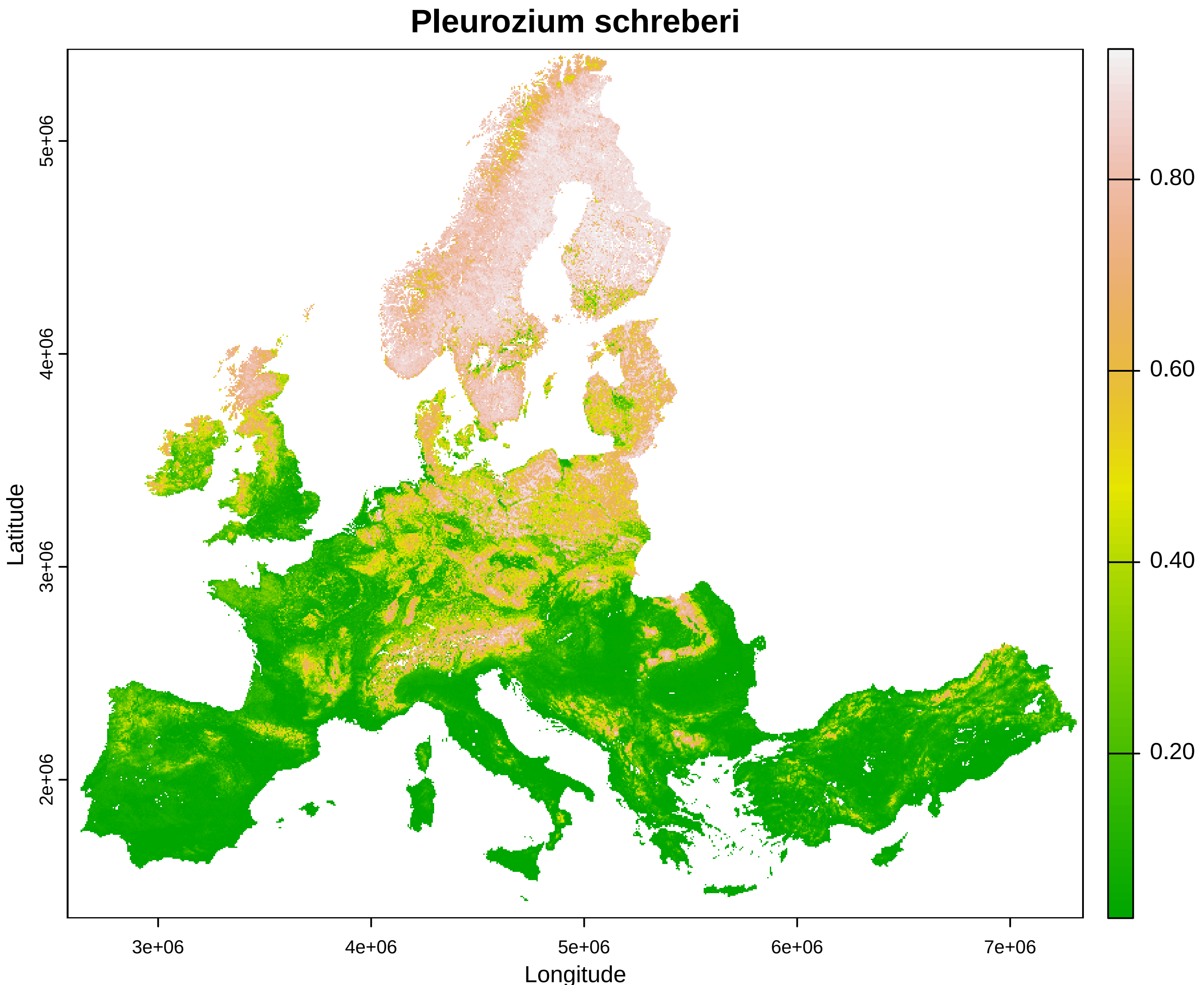
Pleurozium schreberi
Latitude
Longitude
5e+06
4e+06
3e+06
2e+06
3e+06
4e+06
5e+06
6e+06
7e+06
0.80
0.60
0.40
0.20

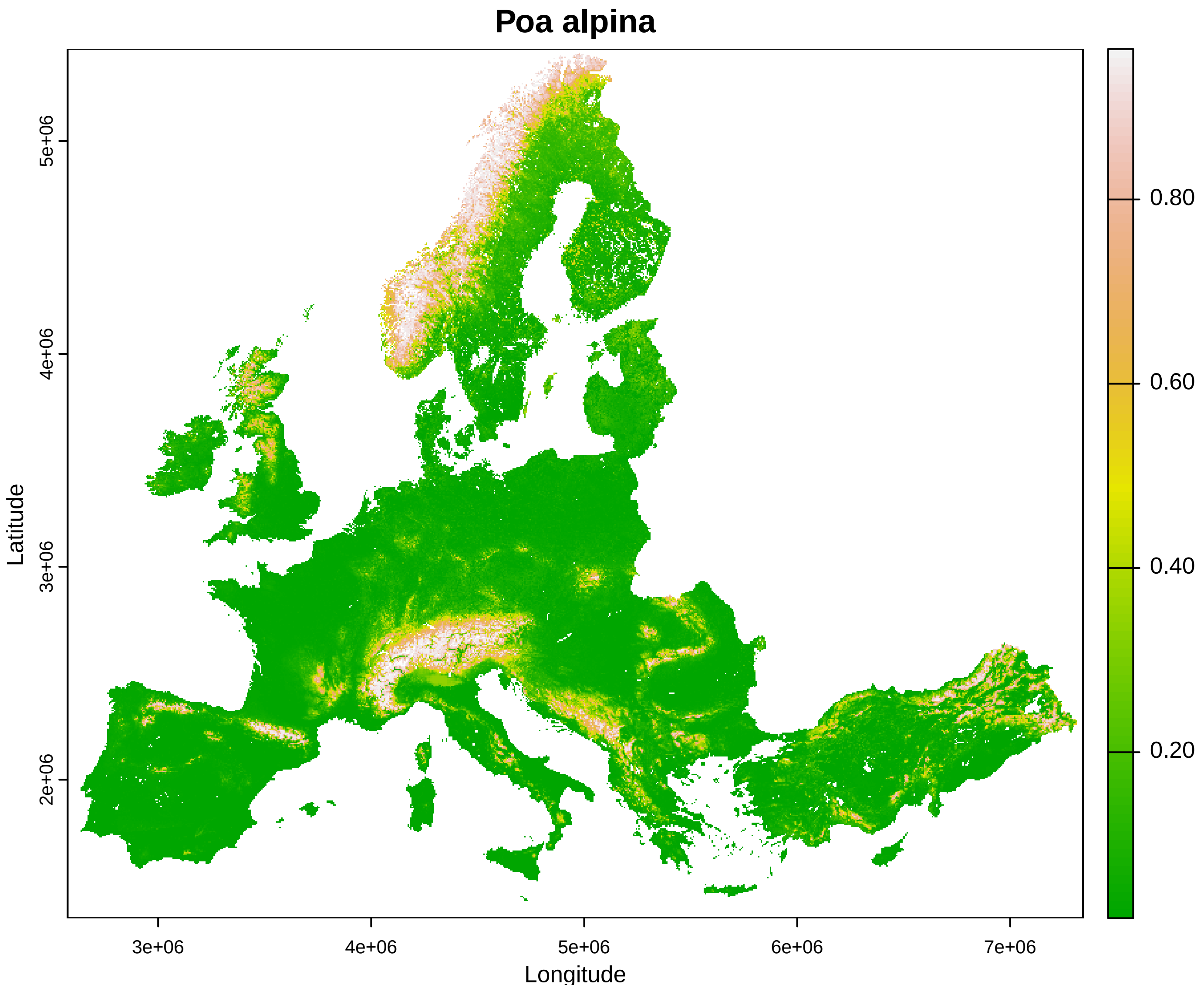
Poa alpina
Latitude
5e+06
4e+06
3e+06
2e+06
3e+06
4e+06
5e+06
6e+06
7e+06
Longitude
0.80
0.60
0.40
0.20

Polygala amarella

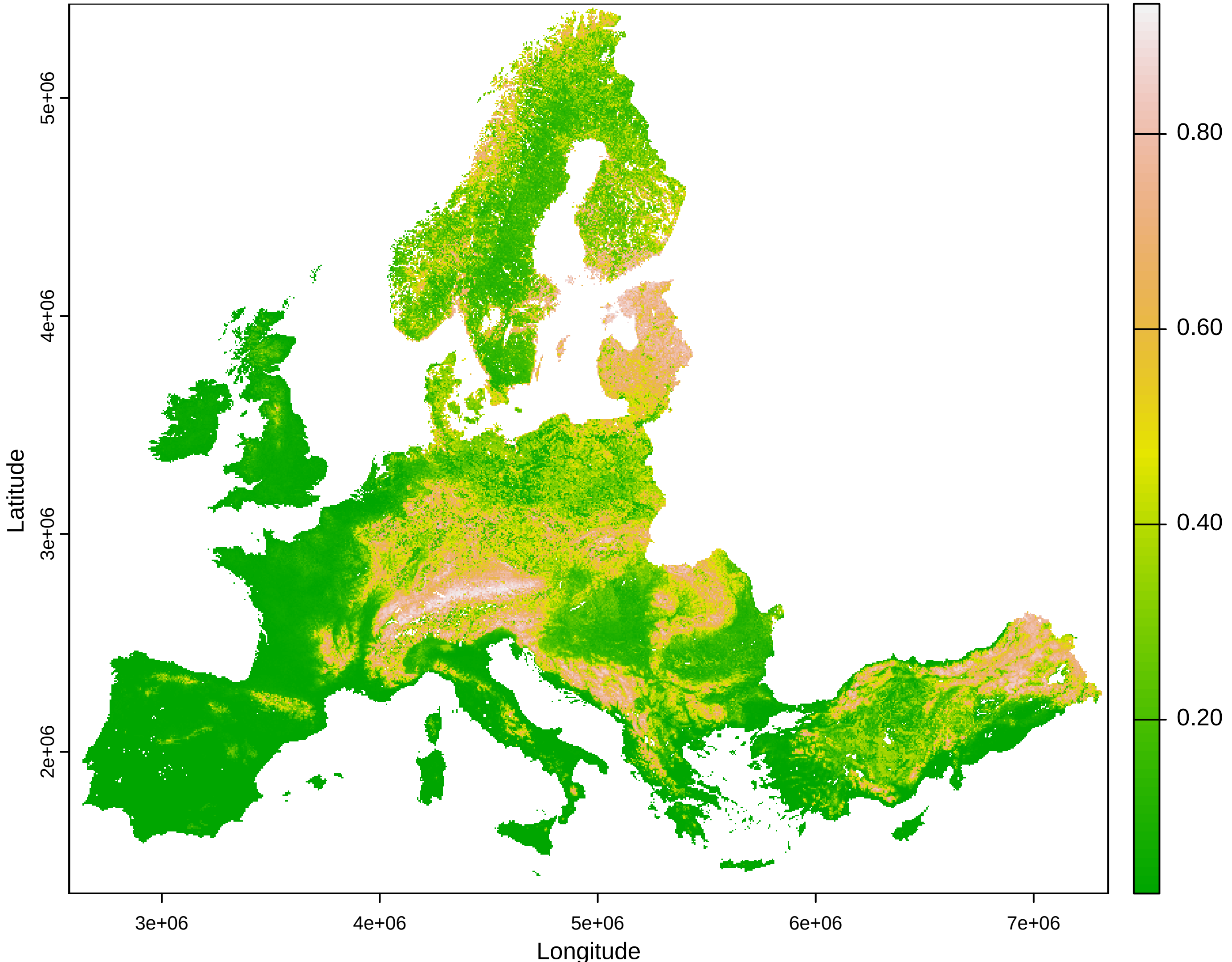

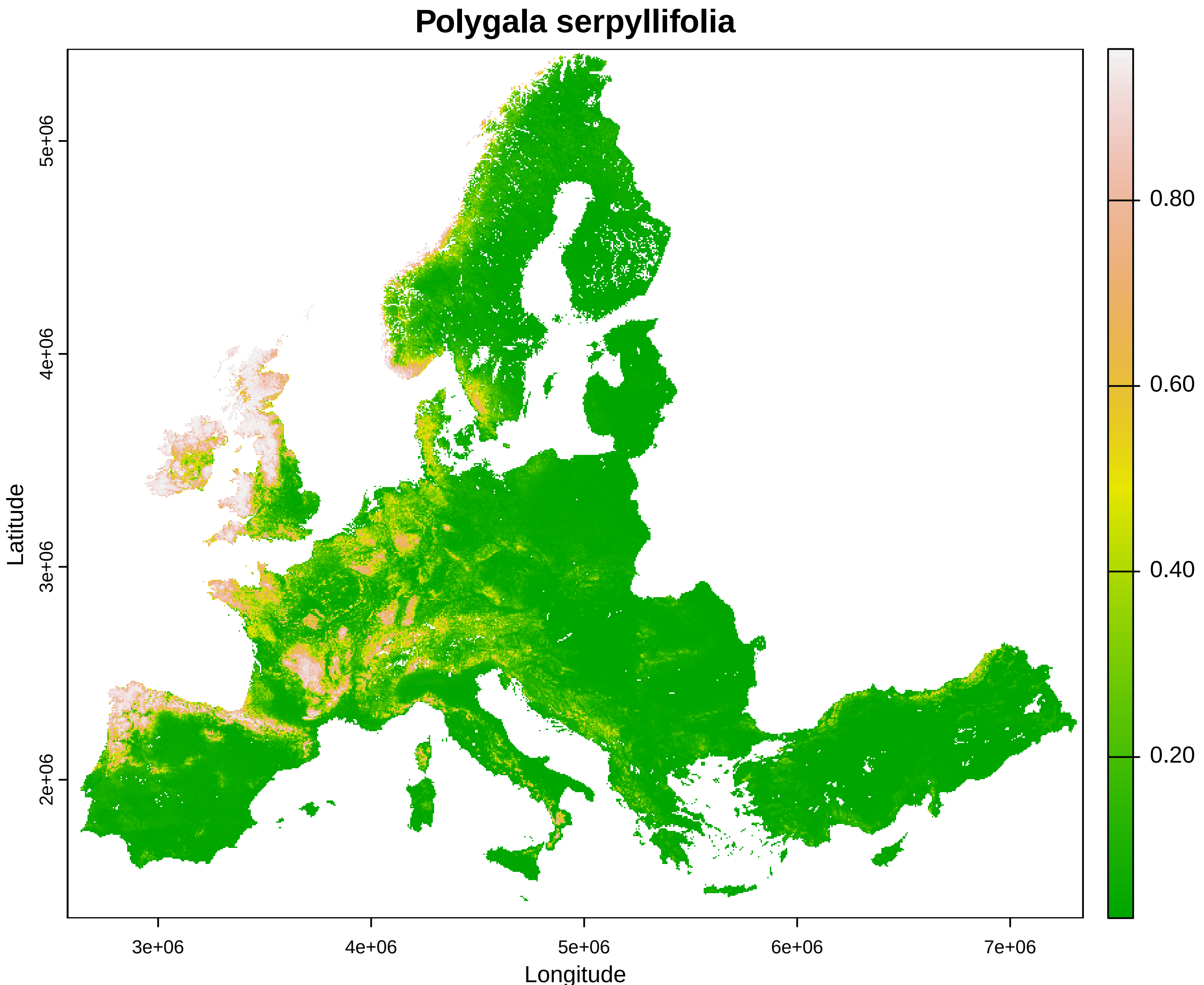
Polygala serpyllifolia
Latitude
Longitude
5e+06
4e+06
3e+06
2e+06
3e+06
4e+06
5e+06
6e+06
7e+06
0.80
0.60
0.40
0.20

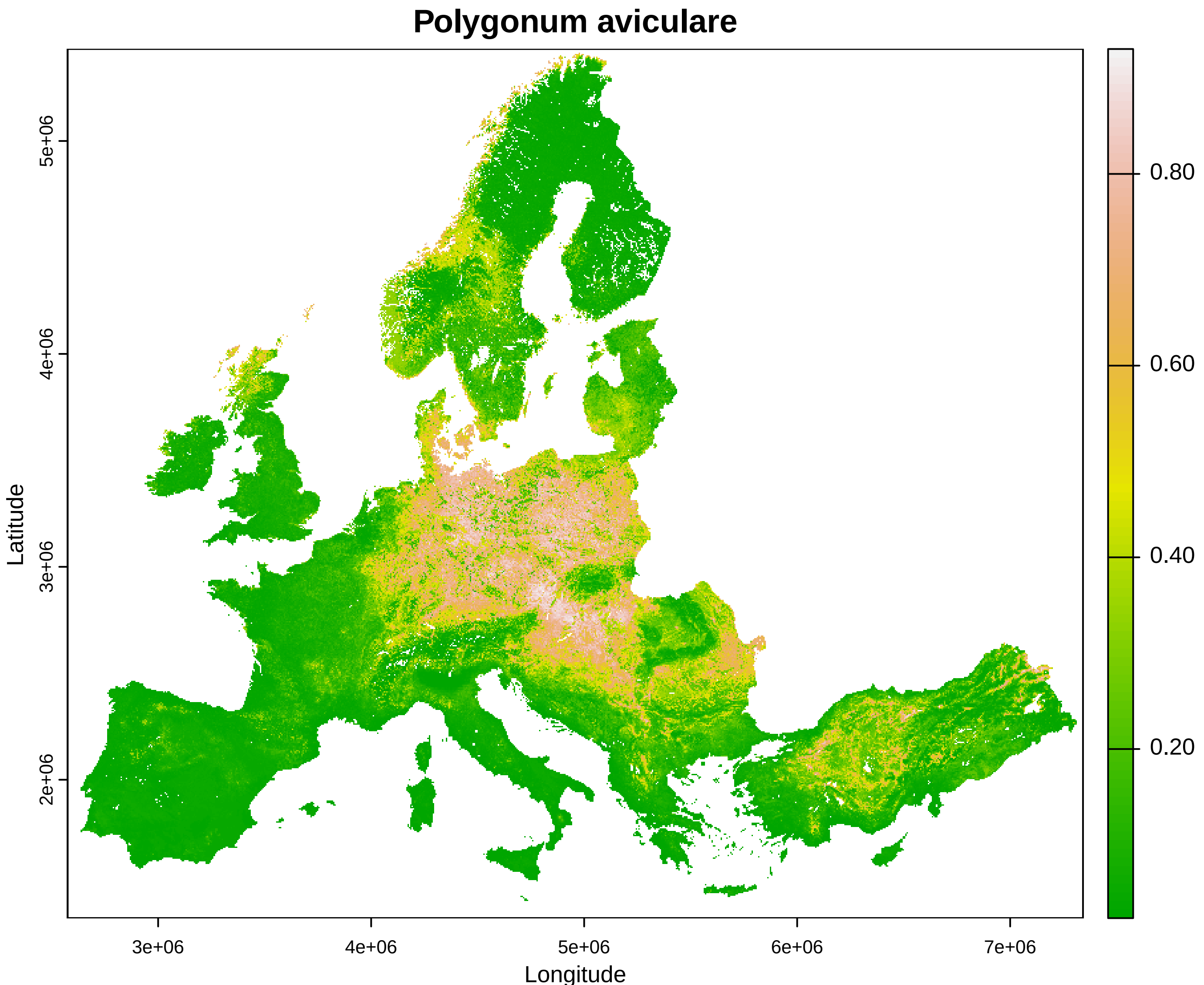

Polygonum aviculare
Latitude
Longitude
5e+06
4e+06
3e+06
2e+06
3e+06
4e+06
5e+06
6e+06
7e+06
0.80
0.60
0.40
0.20

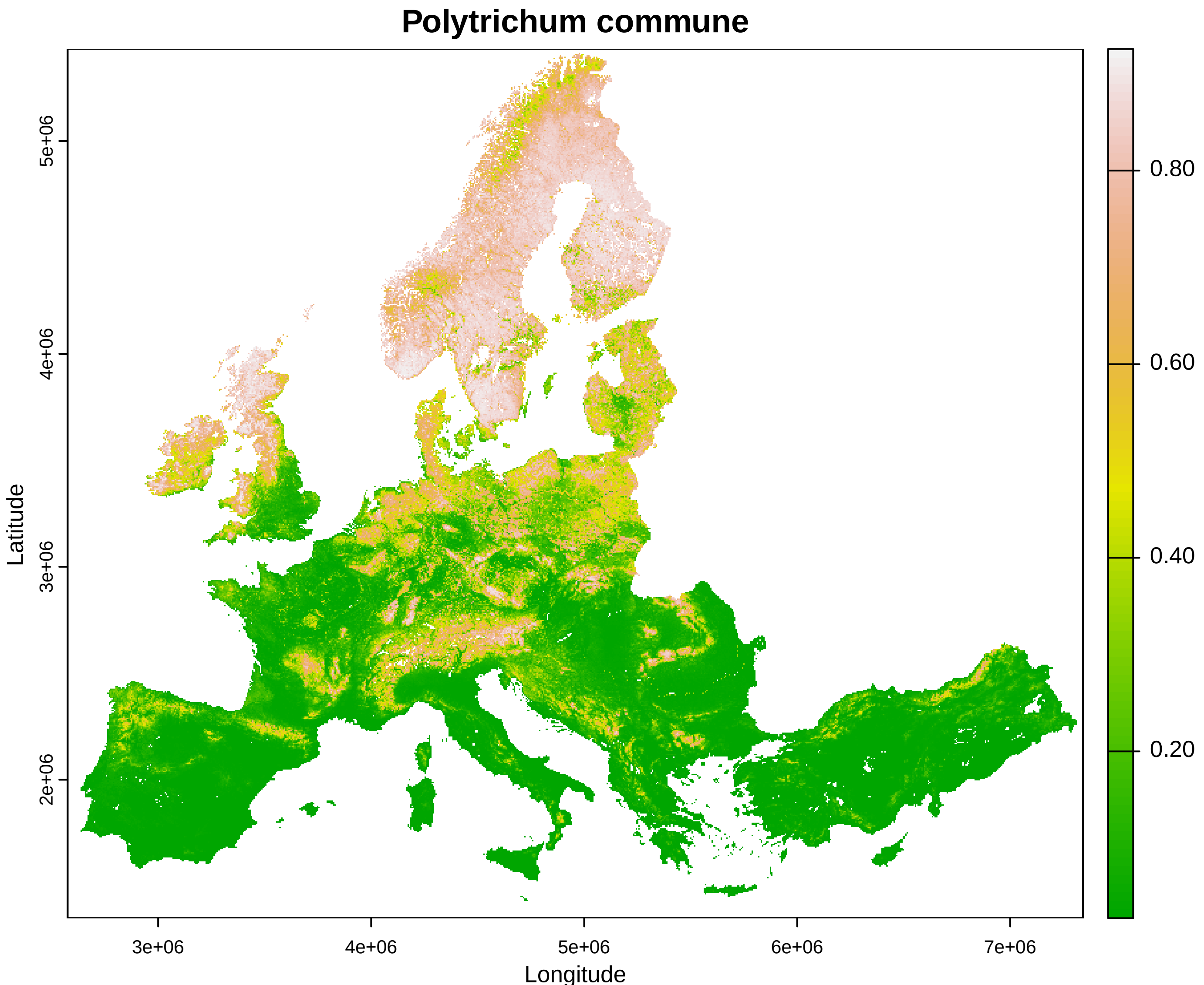
Polytrichum commune
Latitude
Longitude
5e+06
4e+06
3e+06
2e+06
3e+06
4e+06
5e+06
6e+06
7e+06
0.80
0.60
0.40
0.20

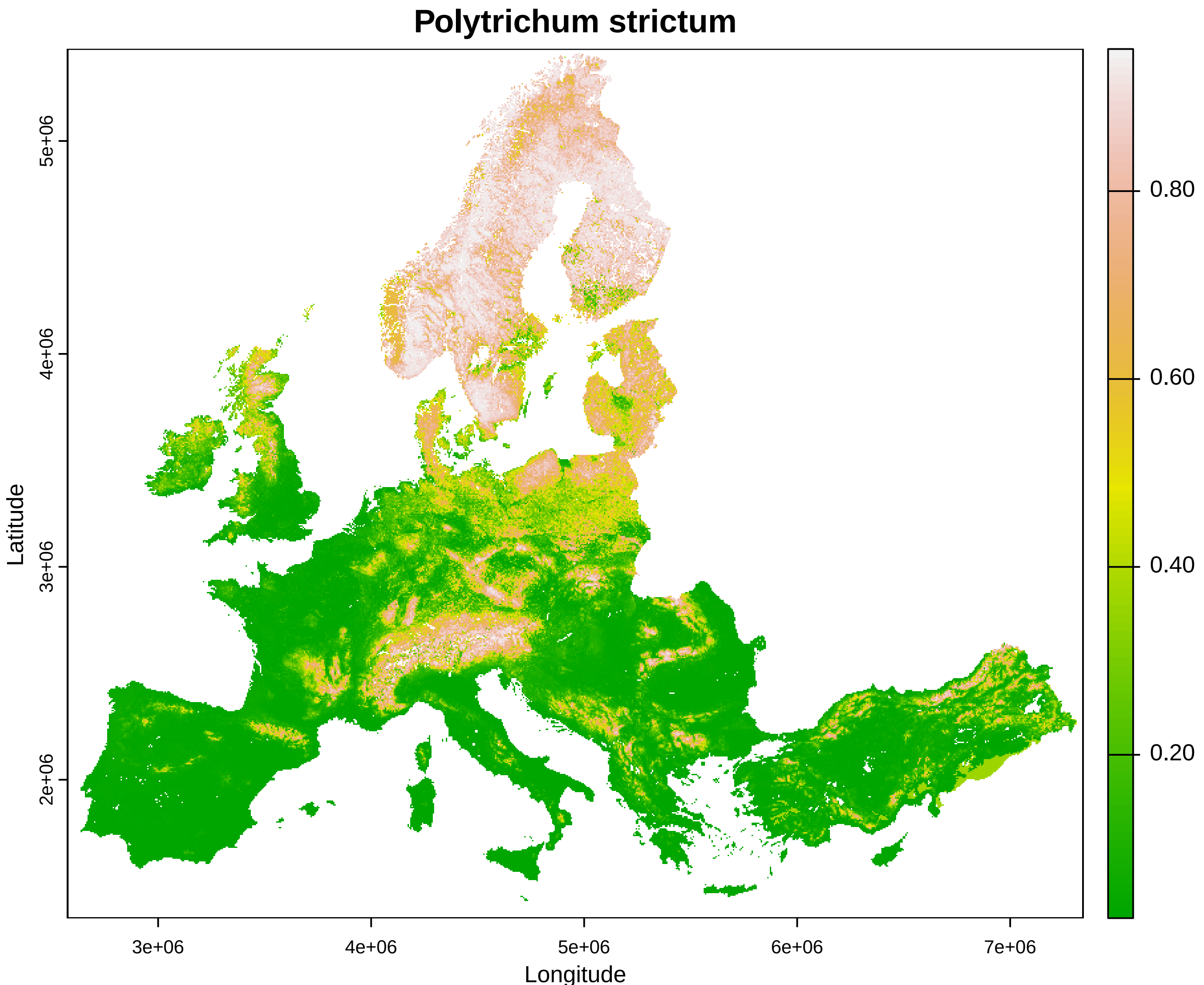
Polytrichum strictum
Latitude
Longitude
5e+06
4e+06
3e+06
2e+06
3e+06
4e+06
5e+06
6e+06
7e+06
0.80
0.60
0.40
0.20

Potamogeton polygonifolius

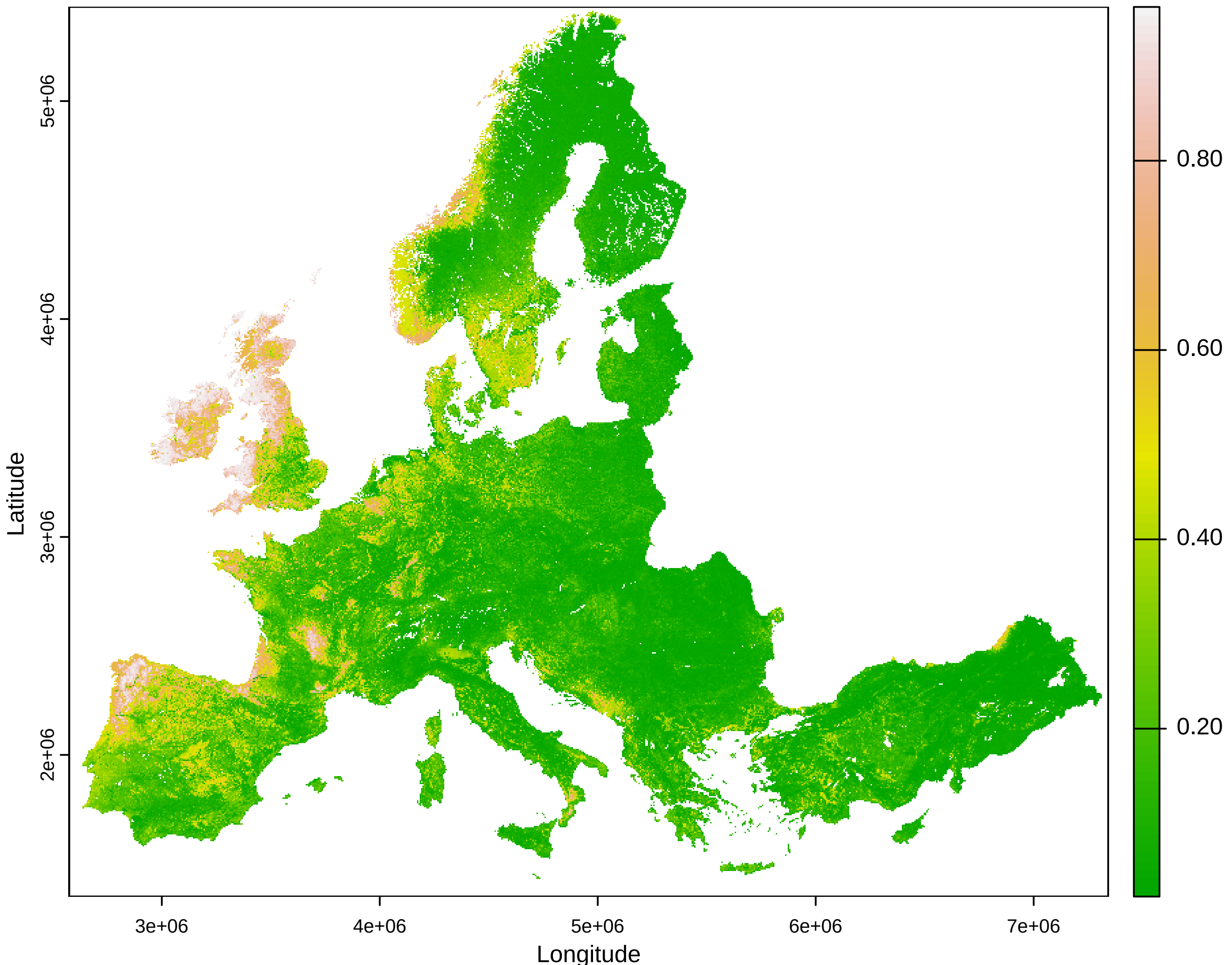

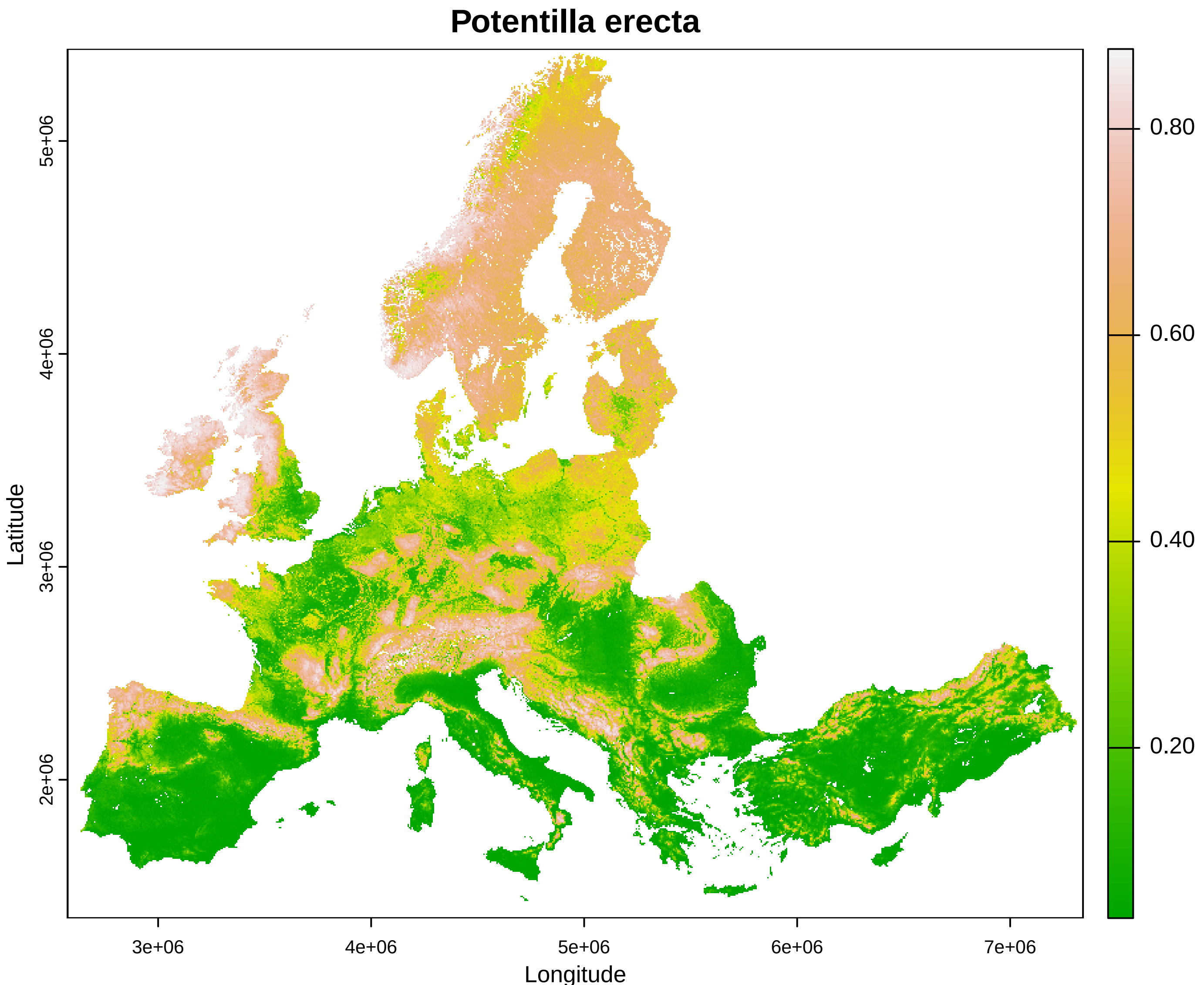

Potentilla erecta
Latitude
Longitude
5e+06
4e+06
3e+06
2e+06
3e+06
4e+06
5e+06
6e+06
7e+06
0.80
0.60
0.40
0.20

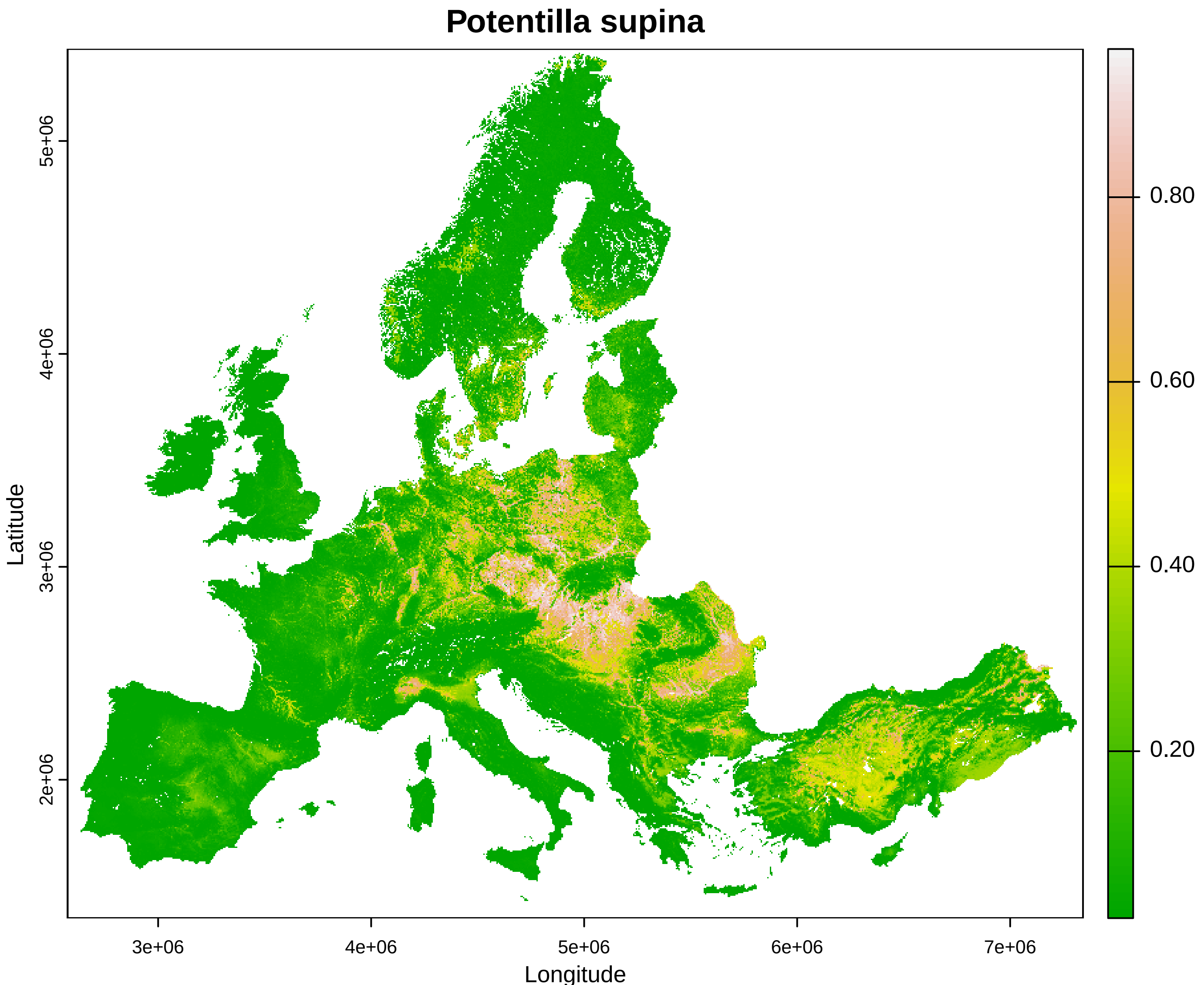
Potentilla supina
Latitude
Longitude
5e+06
4e+06
3e+06
2e+06
3e+06
4e+06
5e+06
6e+06
7e+06
0.80
0.60
0.40
0.20

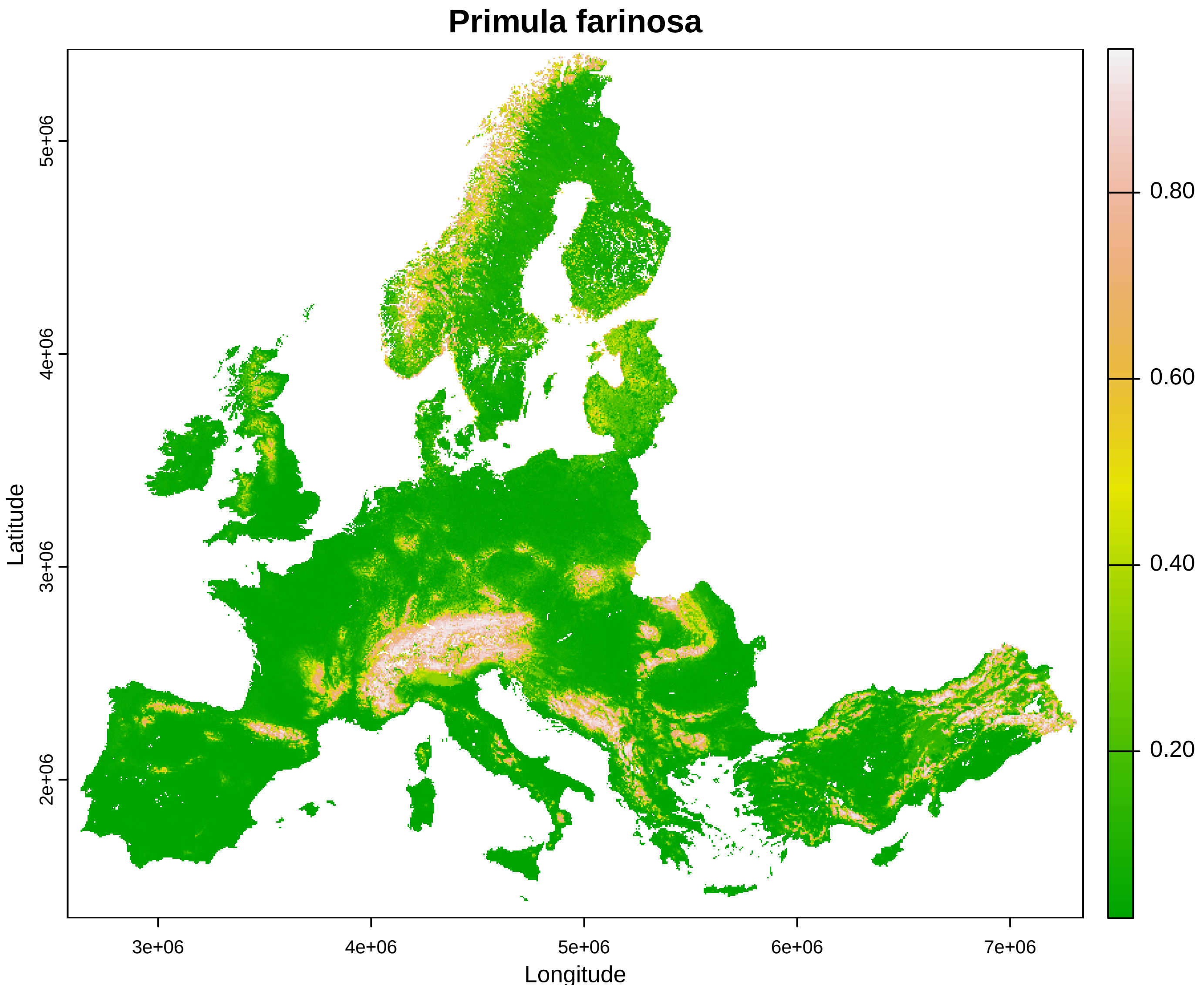
Primula farinosa
Latitude
5e+06
4e+06
3e+06
2e+06
3e+06
4e+06
5e+06
6e+06
7e+06
Longitude
0.80
0.60
0.40
0.20

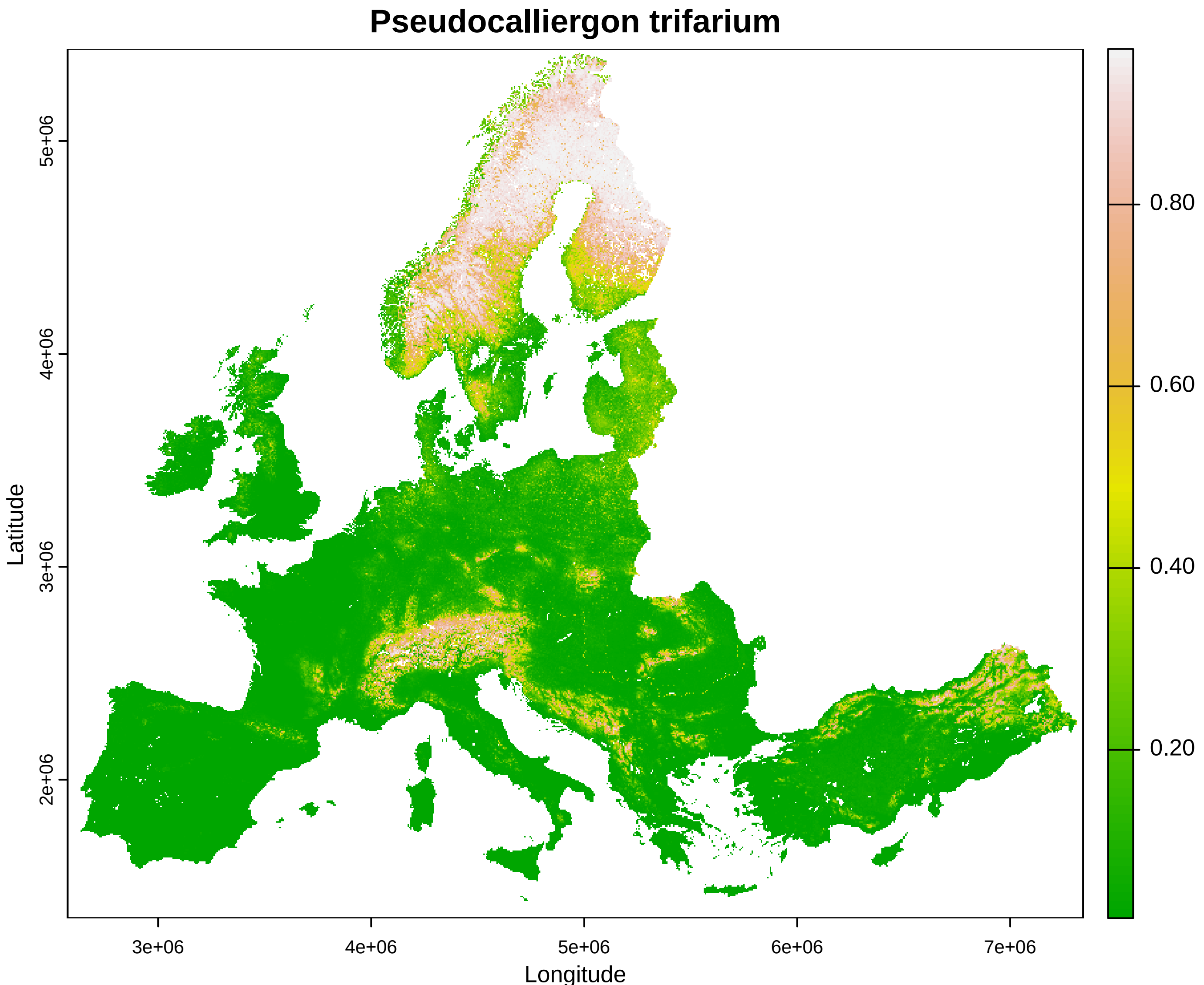

Pseudocalliergon trifarium
Latitude
Longitude
5e+06
4e+06
3e+06
2e+06
3e+06
4e+06
5e+06
6e+06
7e+06
0.80
0.60
0.40
0.20

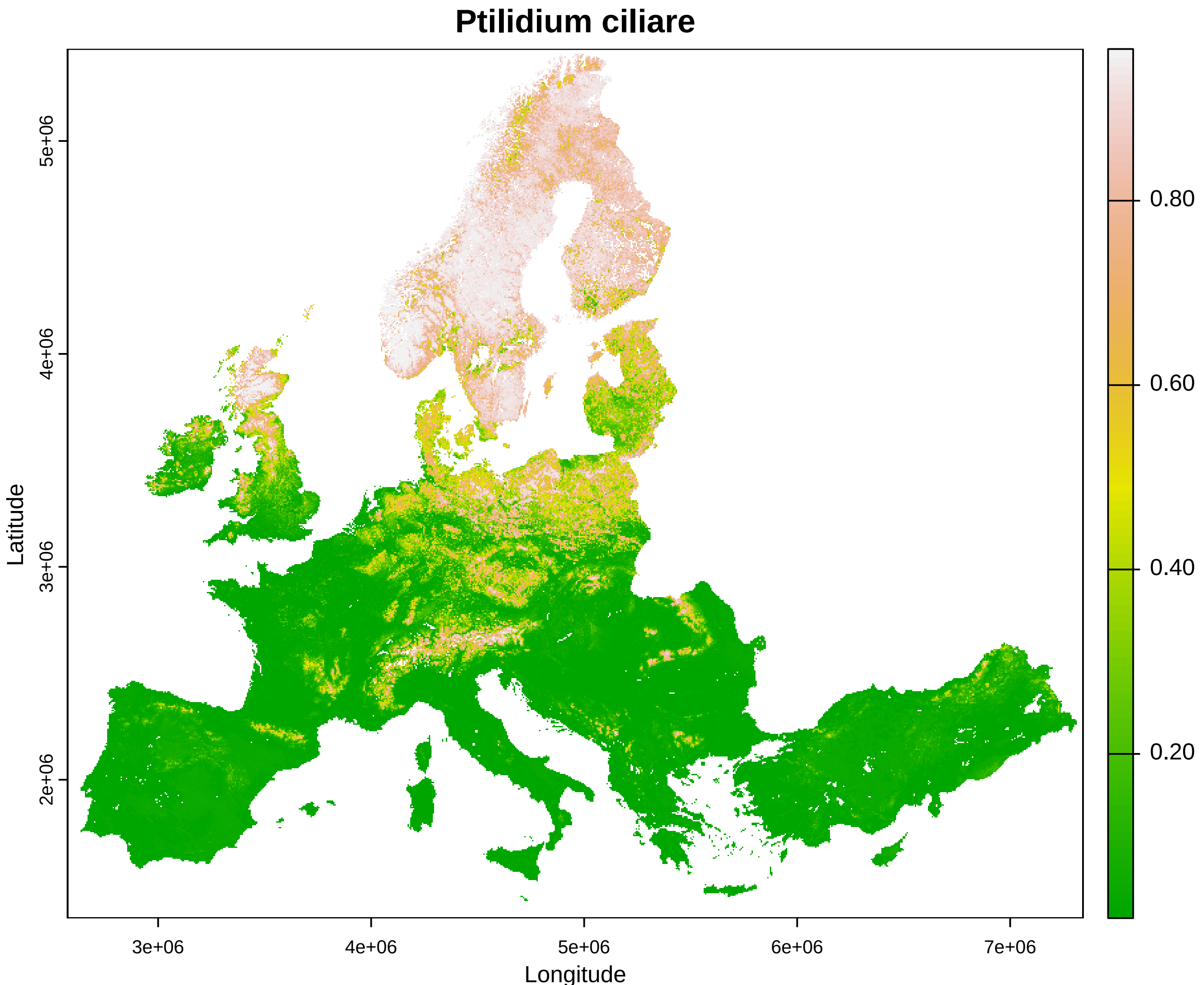

Ptilidium ciliare
Latitude
Longitude
5e+06
4e+06
3e+06
2e+06
3e+06
4e+06
5e+06
6e+06
7e+06
0.80
0.60
0.40
0.20

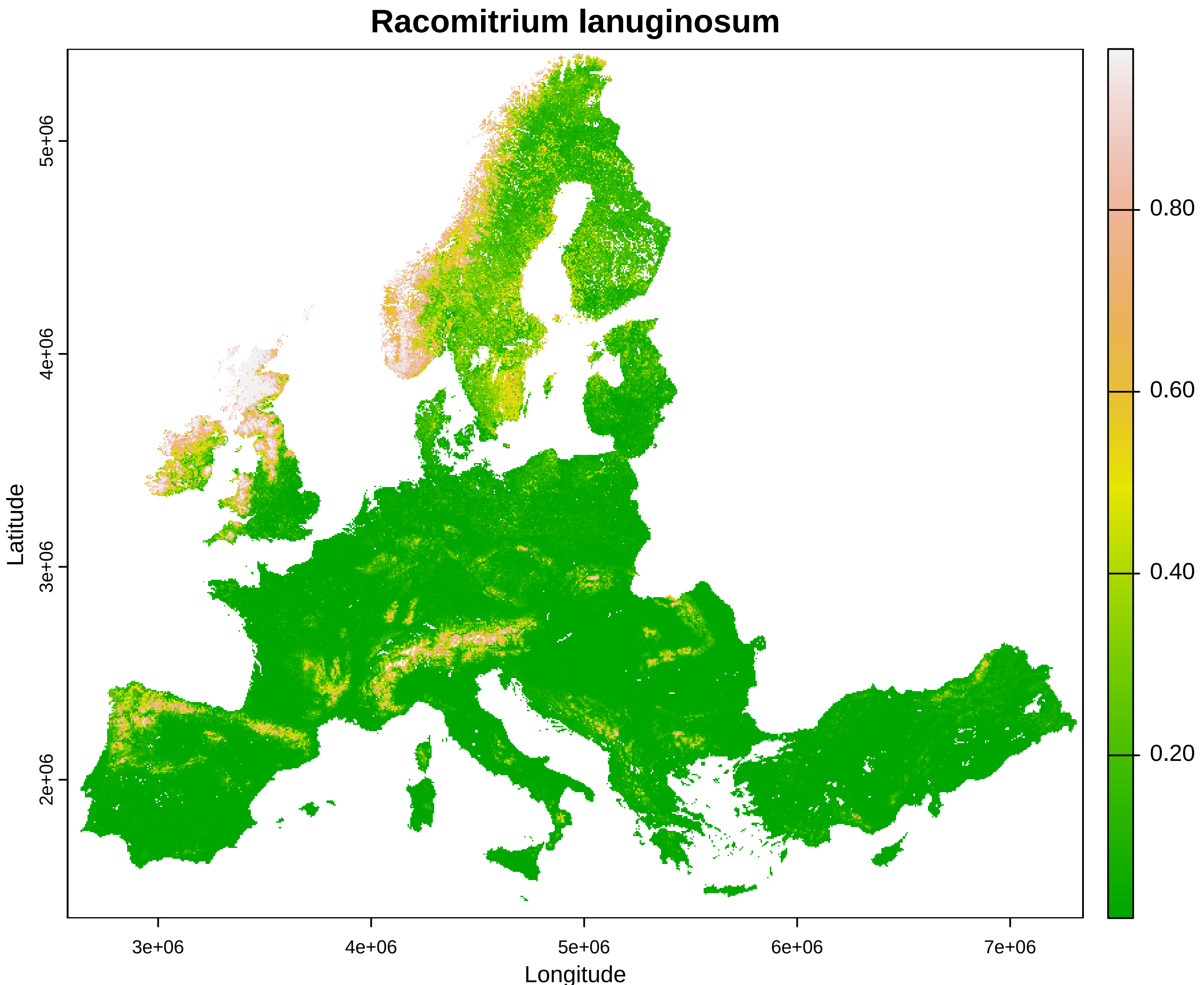

Racomitrium lanuginosum
Latitude
5e+06
4e+06
3e+06
2e+06
3e+06
4e+06
5e+06
6e+06
7e+06
Longitude
0.80
0.60
0.40
0.20

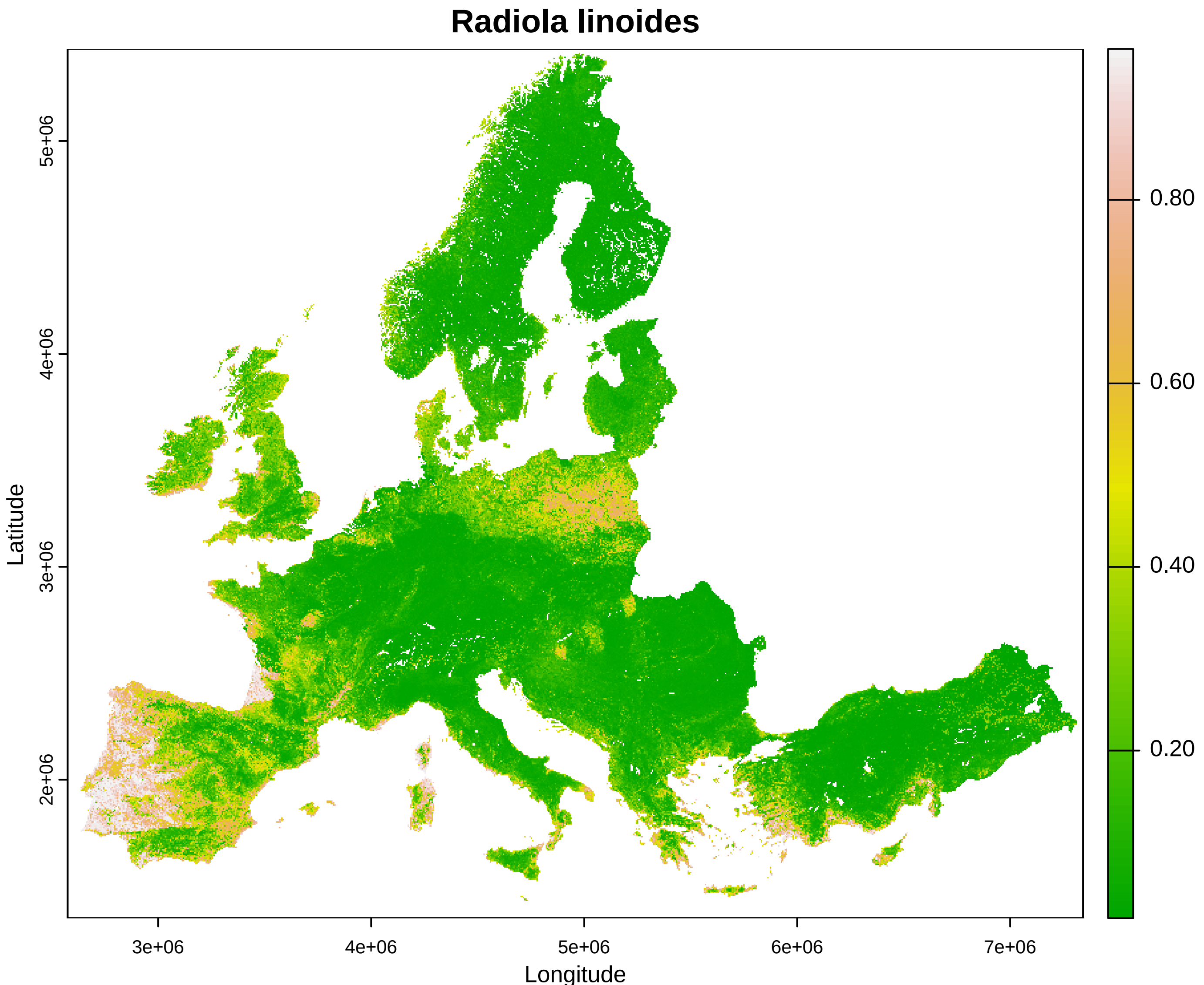
Radiola linoides
Latitude
Longitude
5e+06
4e+06
3e+06
2e+06
3e+06
4e+06
5e+06
6e+06
7e+06
0.80
0.60
0.40
0.20

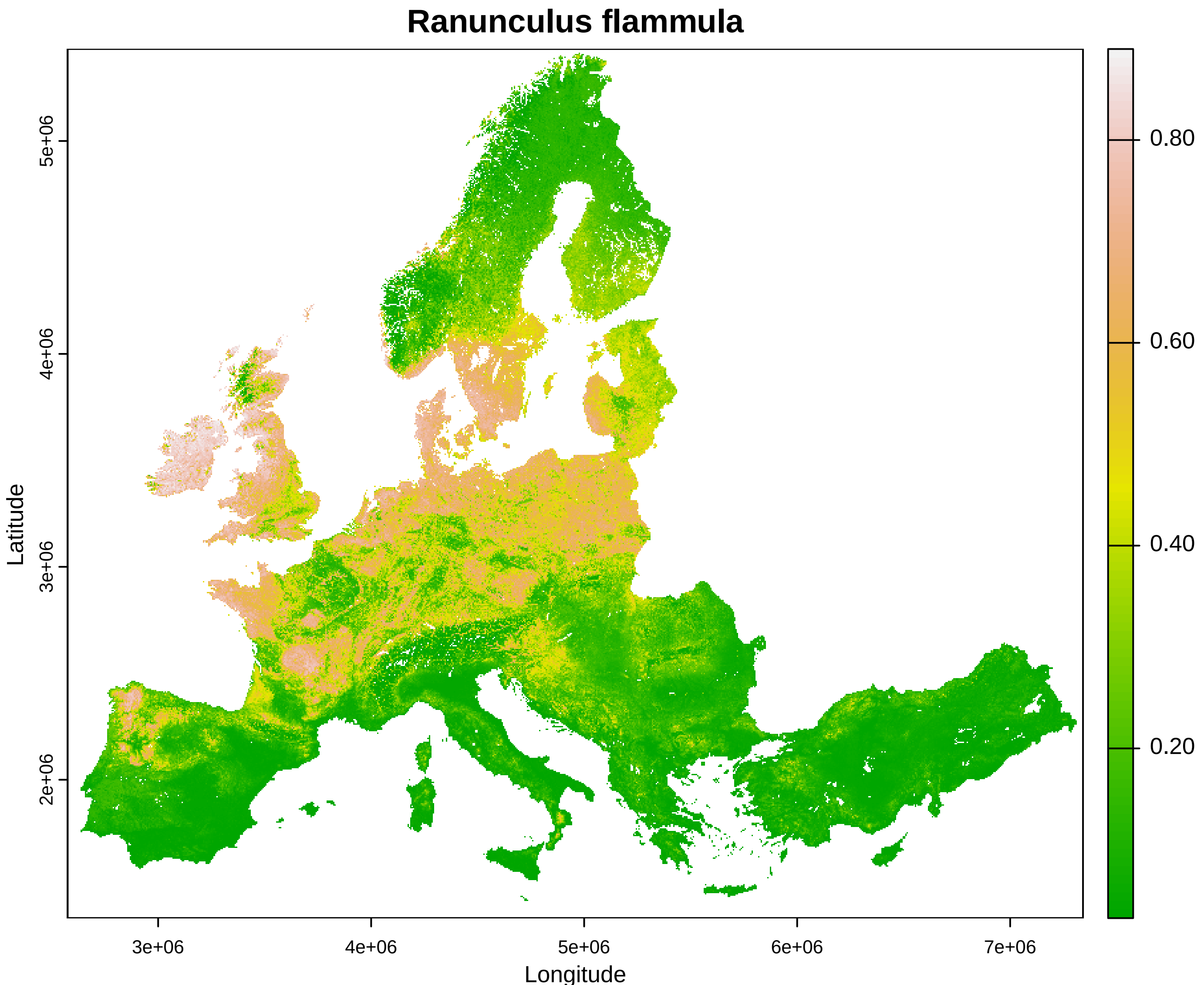

Ranunculus flammula
Latitude
5e+06
4e+06
3e+06
2e+06
3e+06
4e+06
5e+06
6e+06
7e+06
Longitude
0.80
0.60
0.40
0.20

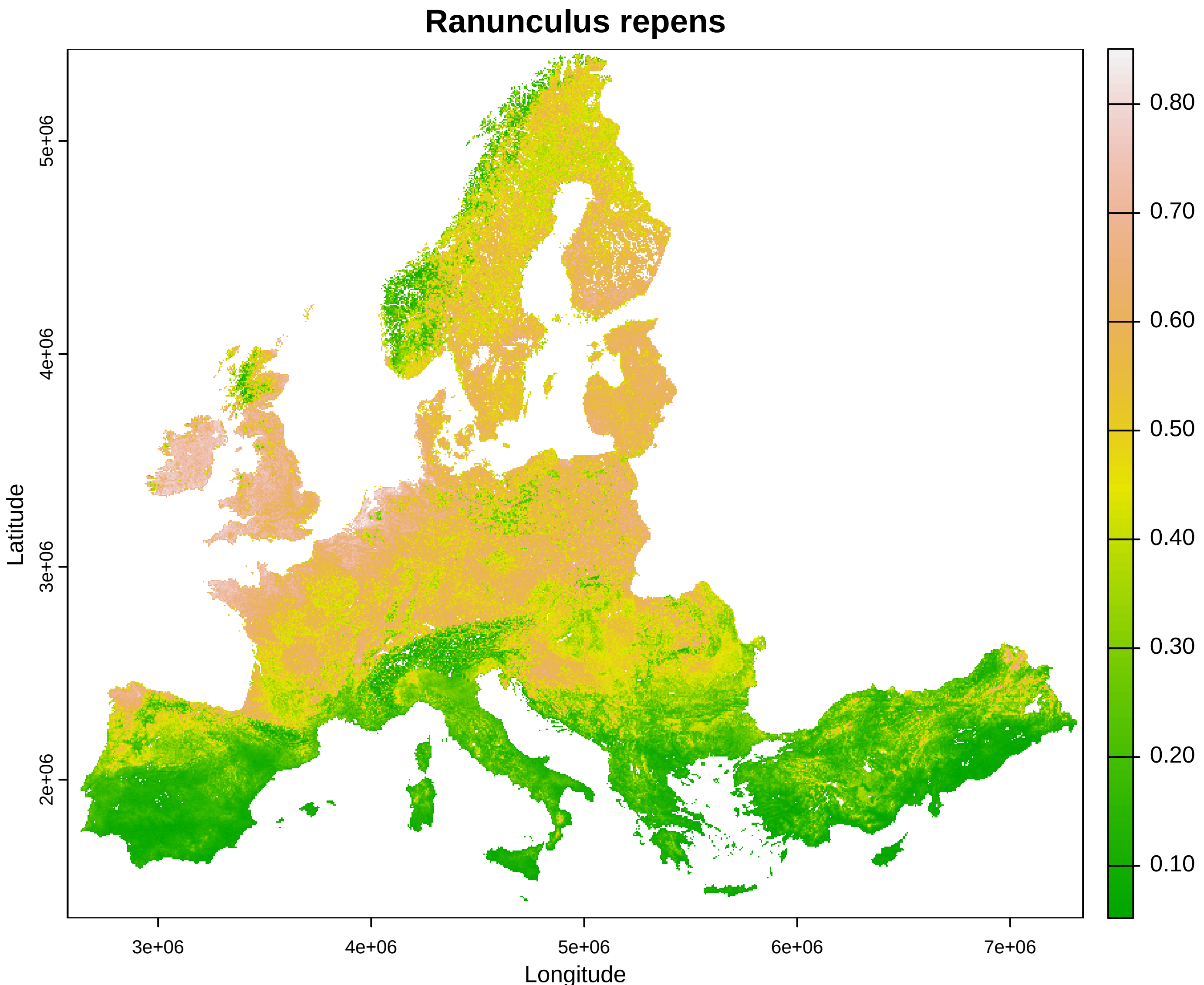
Ranunculus repens
Latitude
5e+06
4e+06
3e+06
2e+06
3e+06
4e+06
5e+06
6e+06
7e+06
Longitude
0.80
0.70
0.60
0.50
0.40
0.30
0.20
0.10

Ranunculus sceleratus

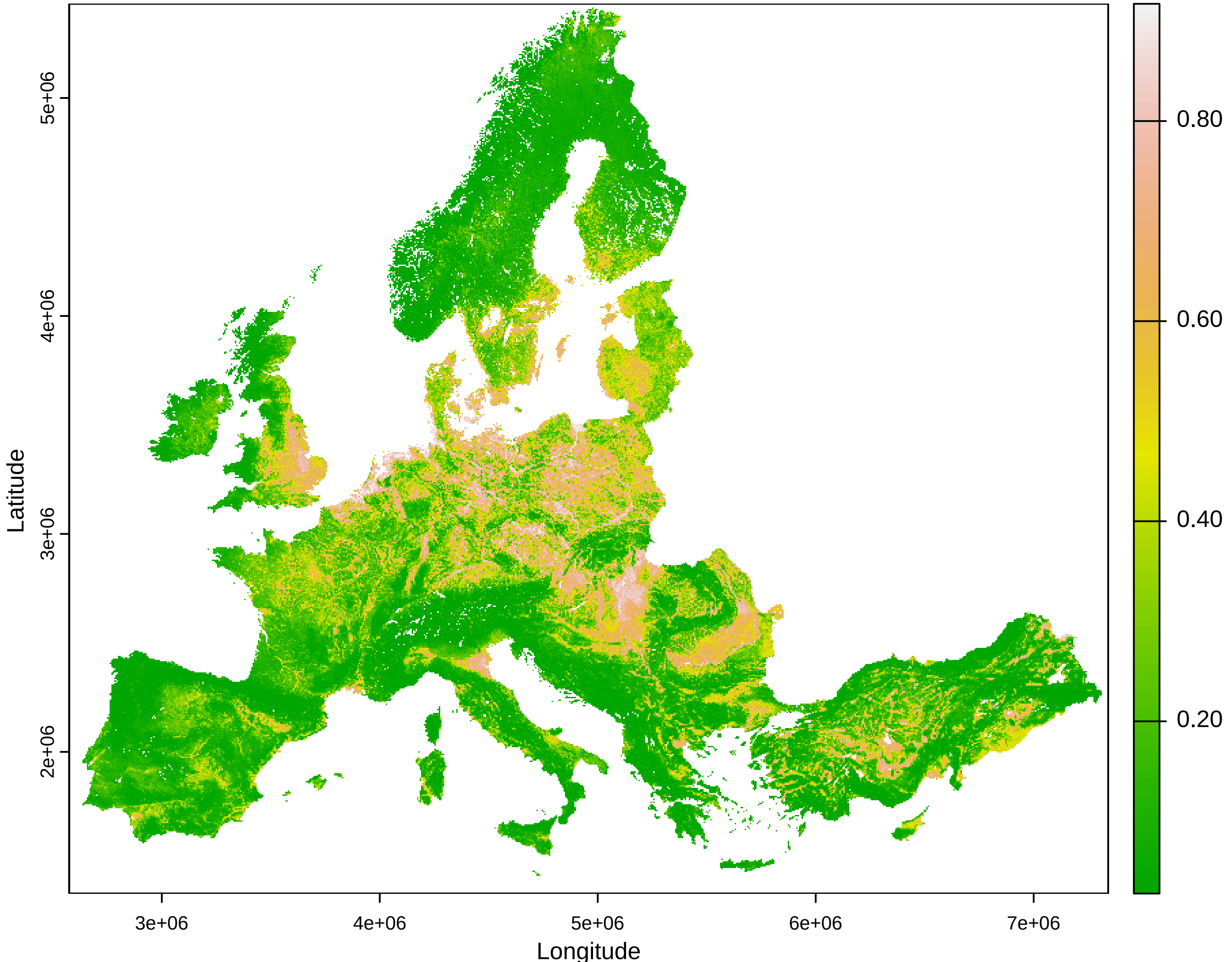

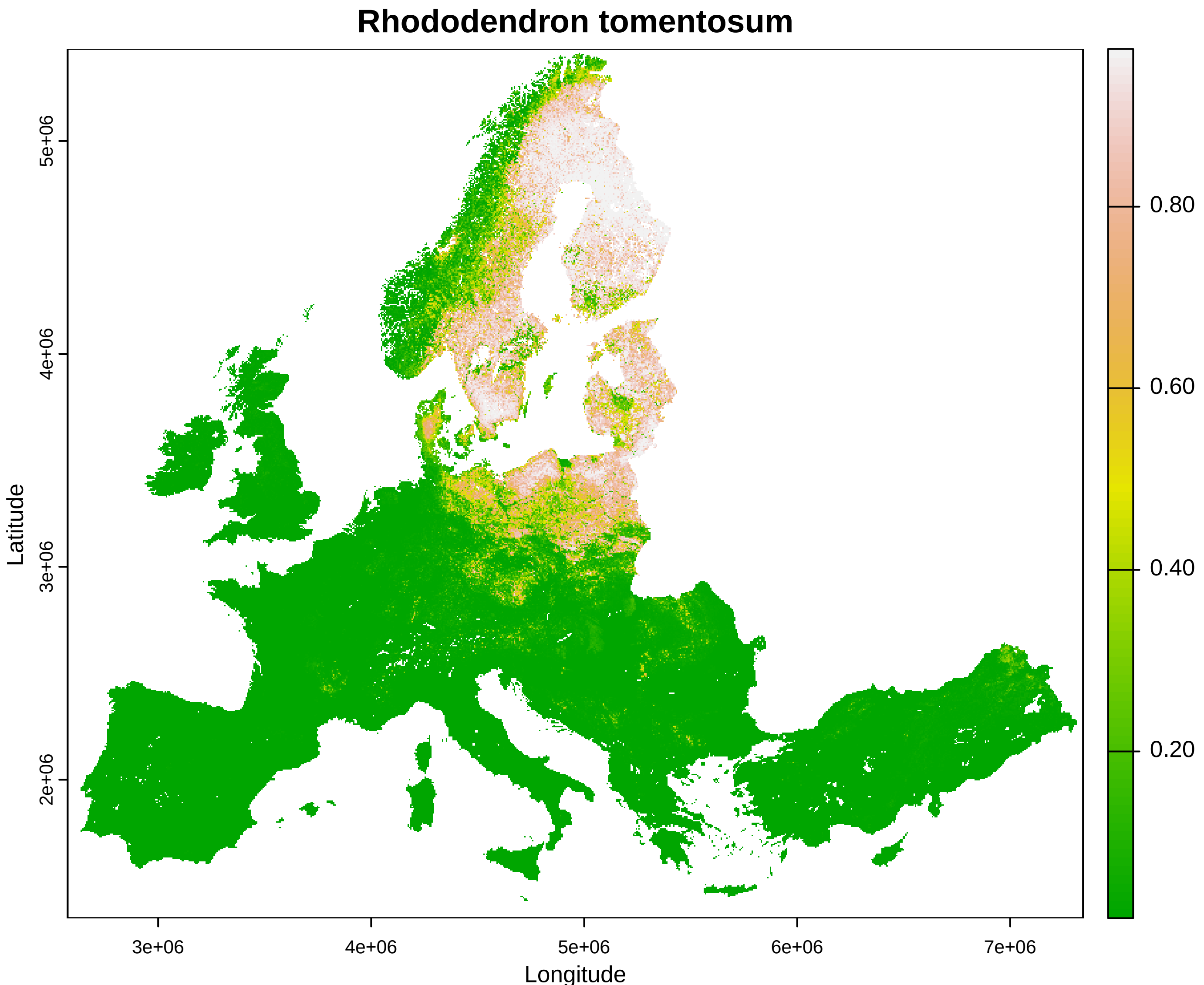

Rhododendron tomentosum
Latitude
Longitude
5e+06
4e+06
3e+06
2e+06
3e+06
4e+06
5e+06
6e+06
7e+06
0.80
0.60
0.40
0.20

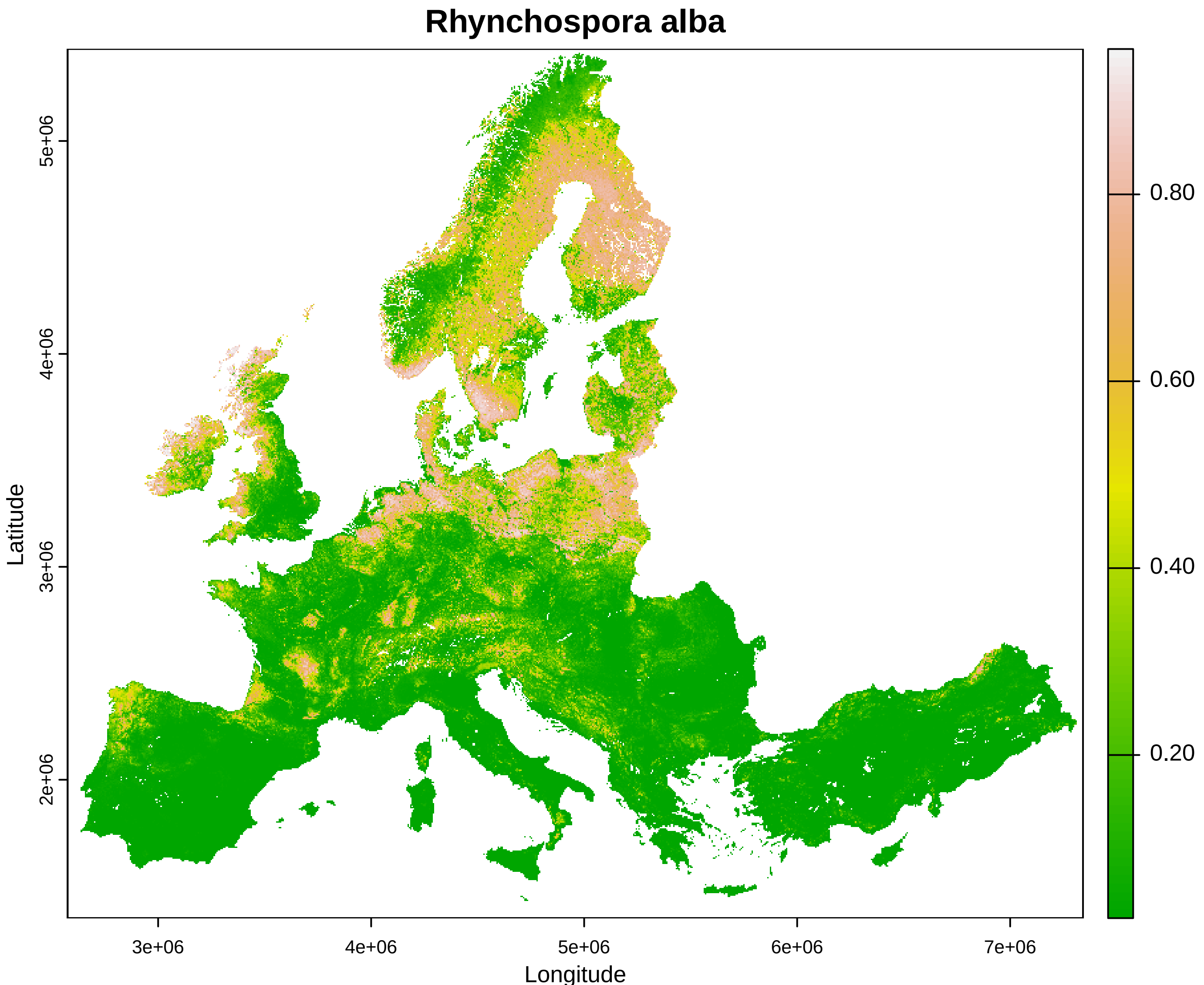
Rhynchospora alba
Latitude
Longitude
5e+06
4e+06
3e+06
2e+06
3e+06
4e+06
5e+06
6e+06
7e+06
0.80
0.60
0.40
0.20

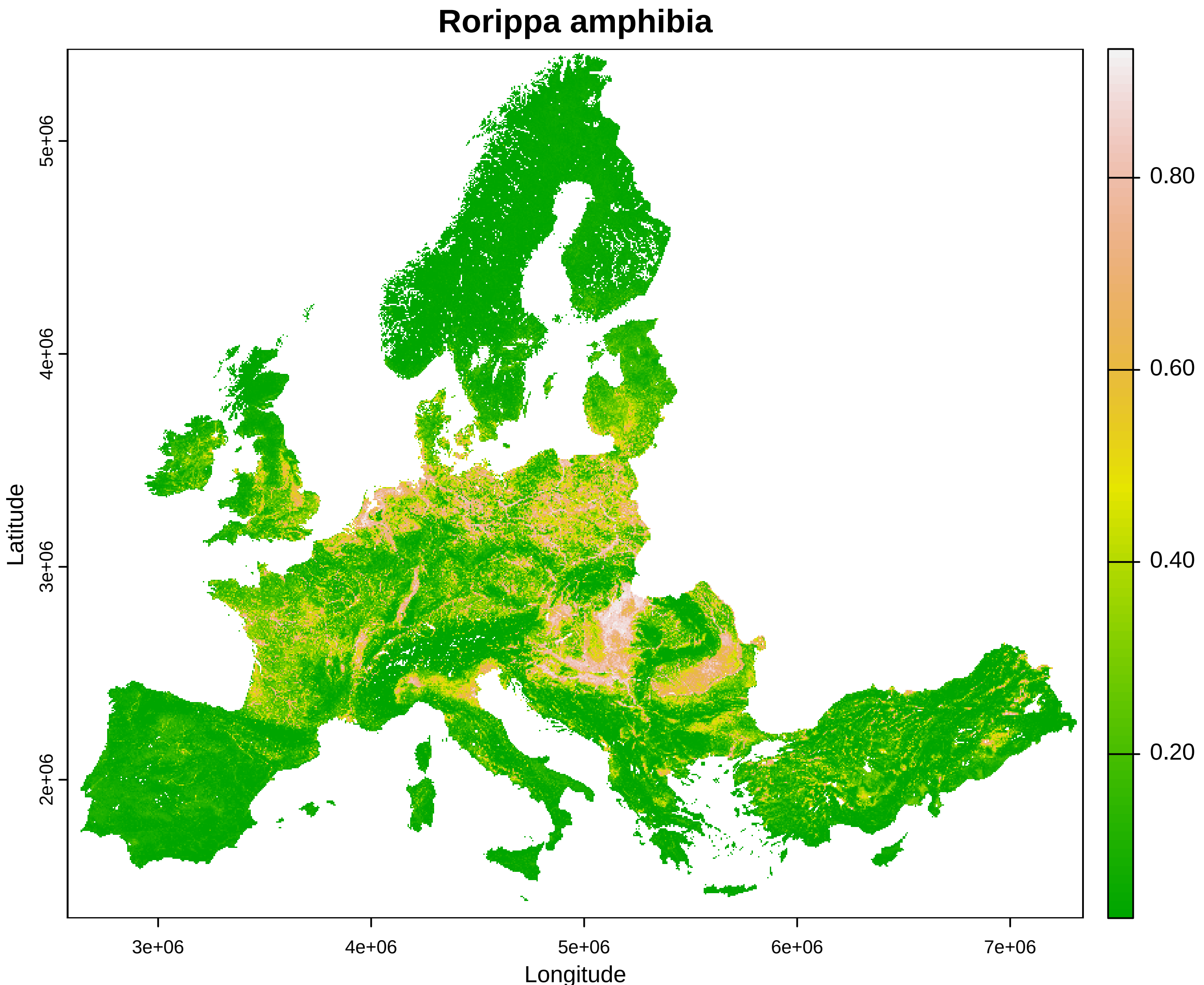
Rorippa amphibia
Latitude
Longitude
5e+06
4e+06
3e+06
2e+06
3e+06
4e+06
5e+06
6e+06
7e+06
0.80
0.60
0.40
0.20

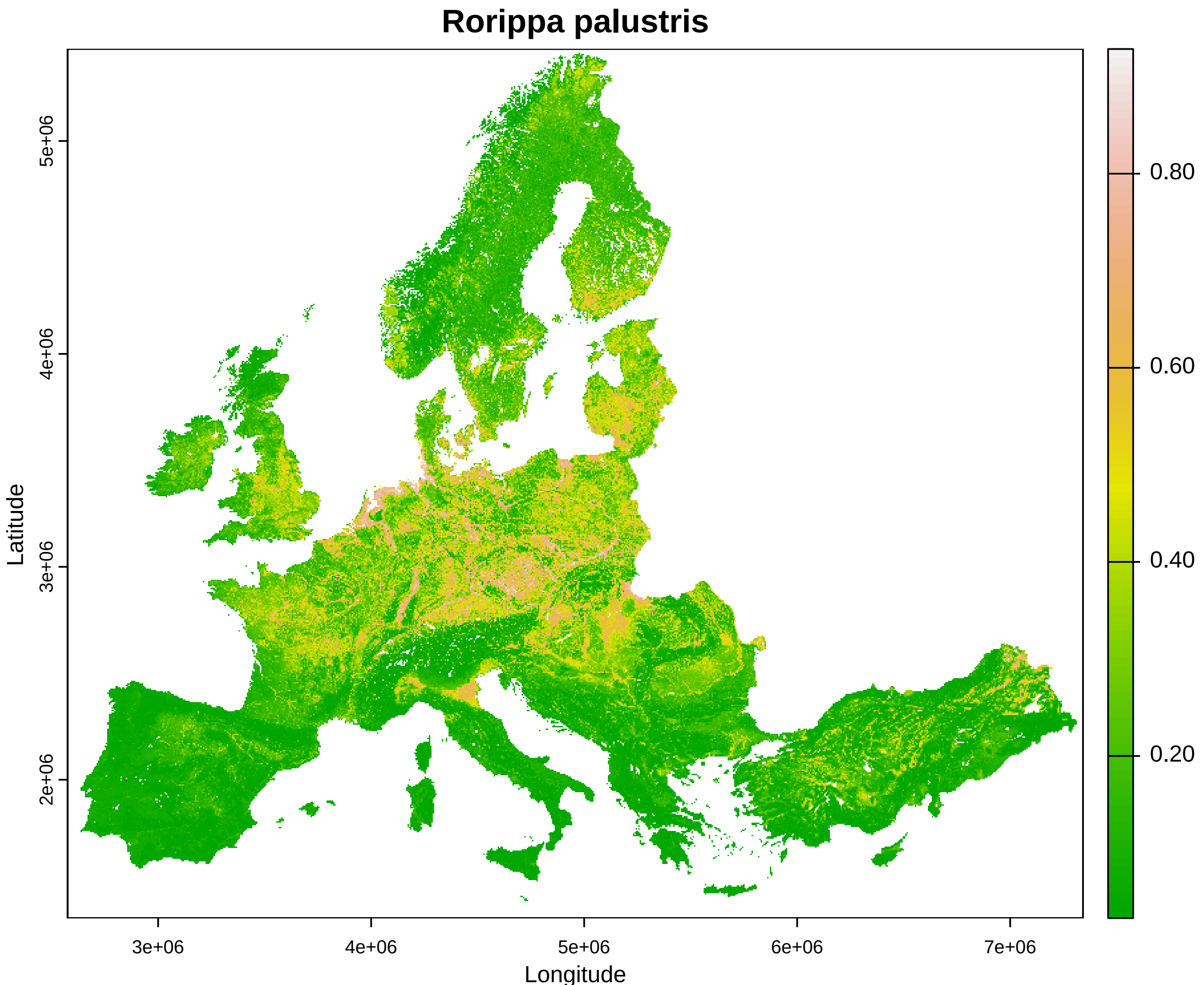
Rorippa palustris
Latitude
Longitude
5e+06
4e+06
3e+06
2e+06
3e+06
4e+06
5e+06
6e+06
7e+06
0.80
0.60
0.40
0.20

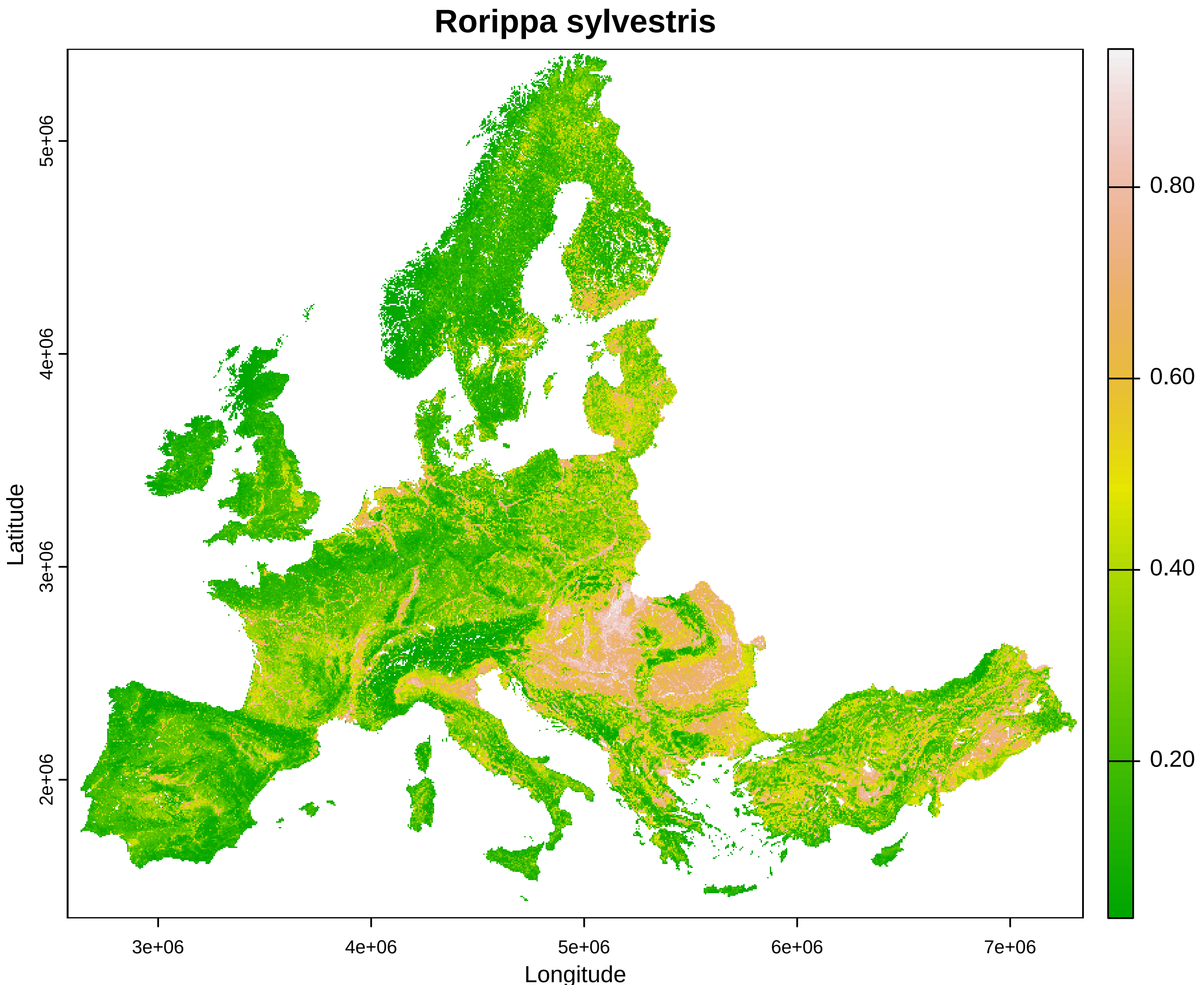

Rorippa sylvestris
Latitude
Longitude
5e+06
4e+06
3e+06
2e+06
3e+06
4e+06
5e+06
6e+06
7e+06
0.80
0.60
0.40
0.20

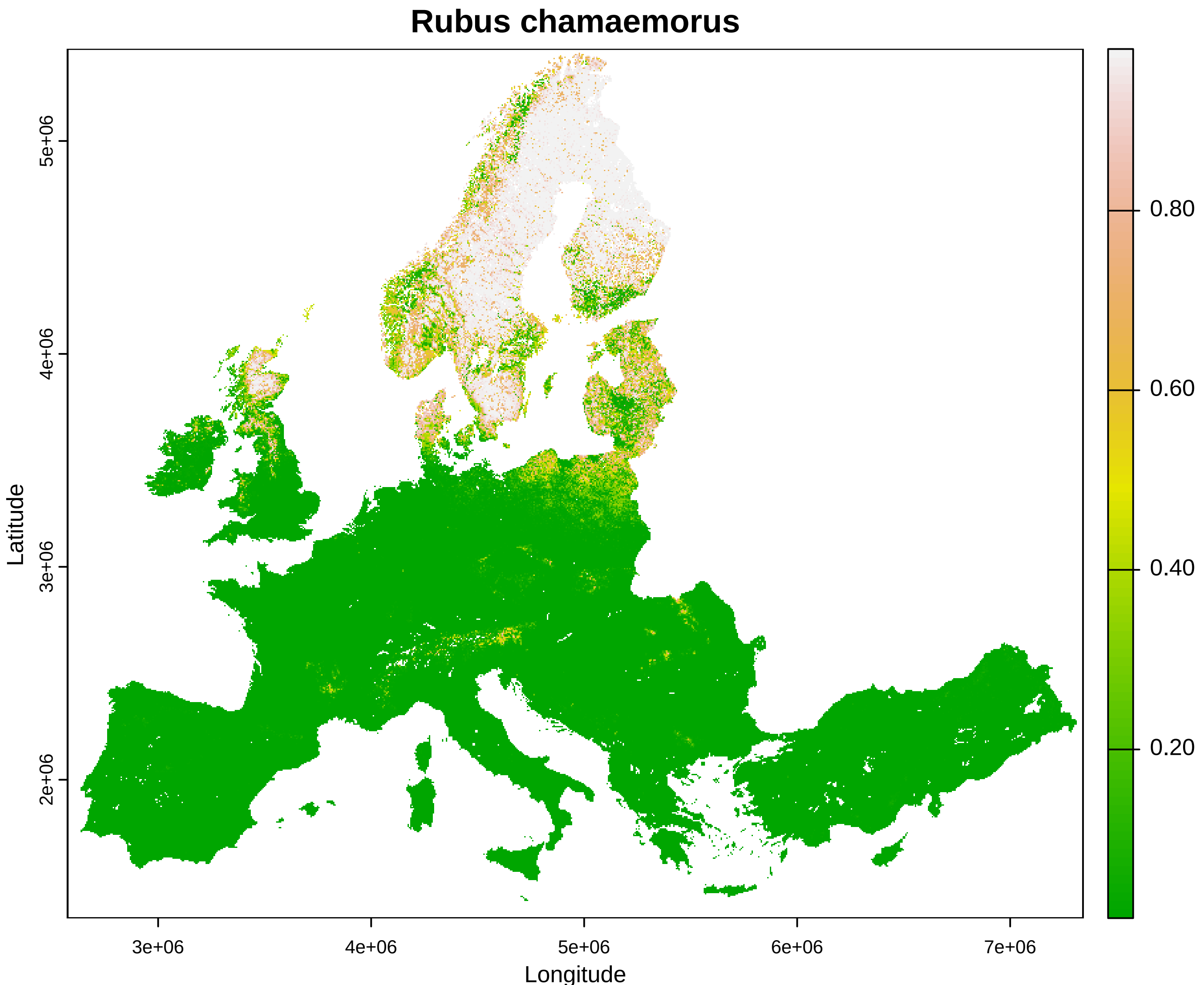

Rubus chamaemorus
Latitude
Longitude
5e+06
4e+06
3e+06
2e+06
3e+06
4e+06
5e+06
6e+06
7e+06
0.80
0.60
0.40
0.20

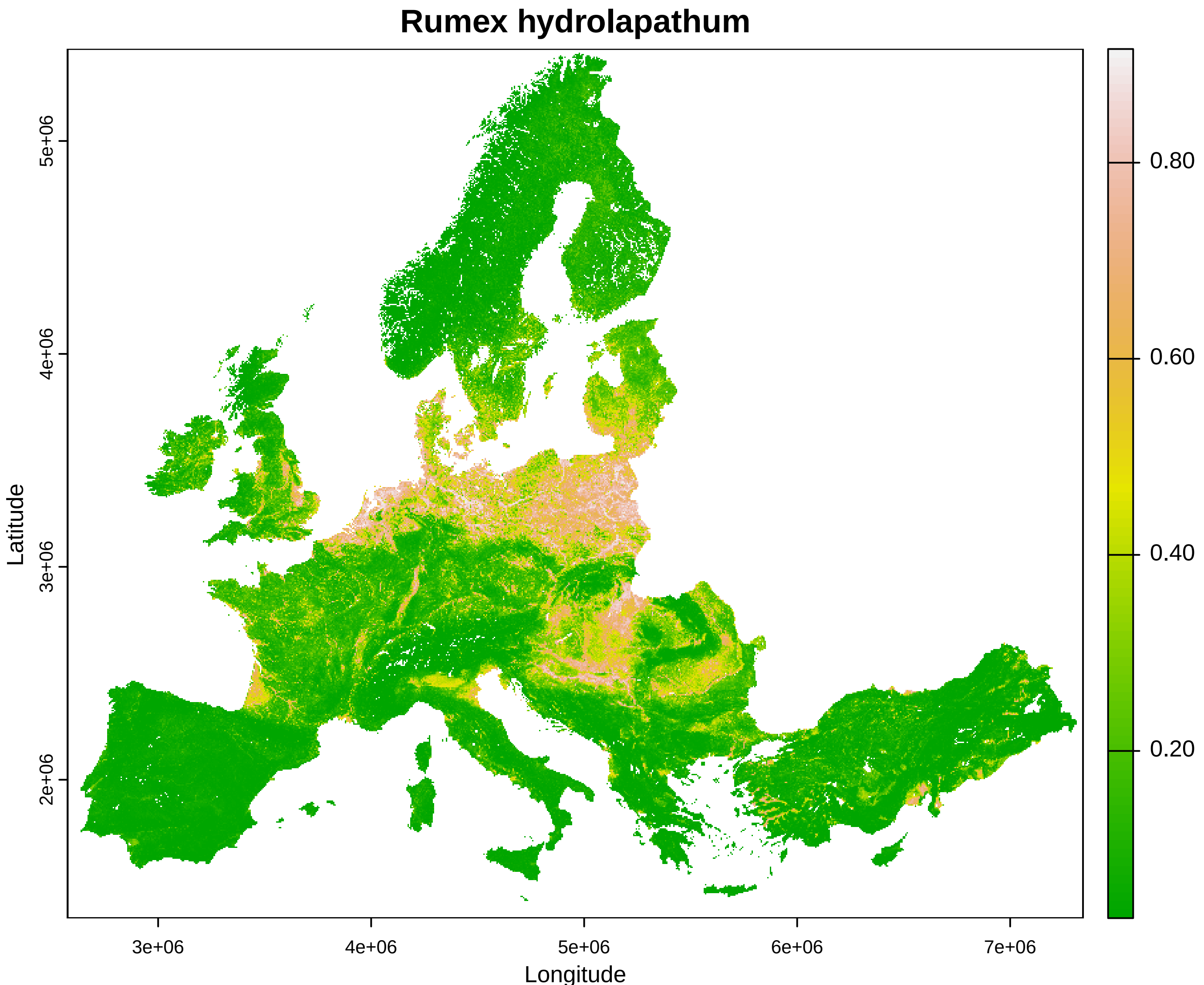

Rumex hydrolapathum
Latitude
5e+06
4e+06
3e+06
2e+06
3e+06
4e+06
5e+06
6e+06
7e+06
Longitude
0.80
0.60
0.40
0.20

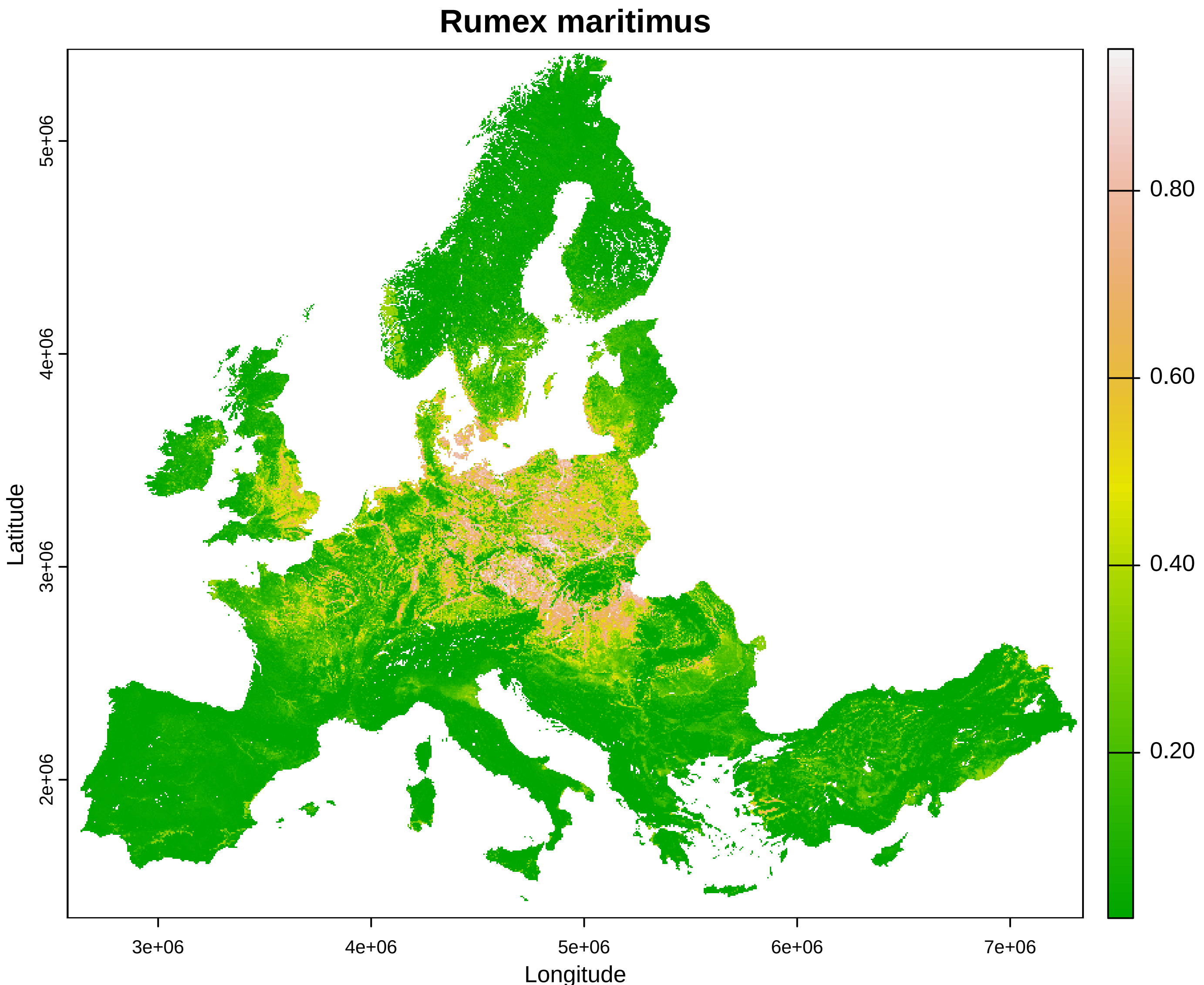
Rumex maritimus
Latitude
Longitude
5e+06
4e+06
3e+06
2e+06
3e+06
4e+06
5e+06
6e+06
7e+06
0.80
0.60
0.40
0.20

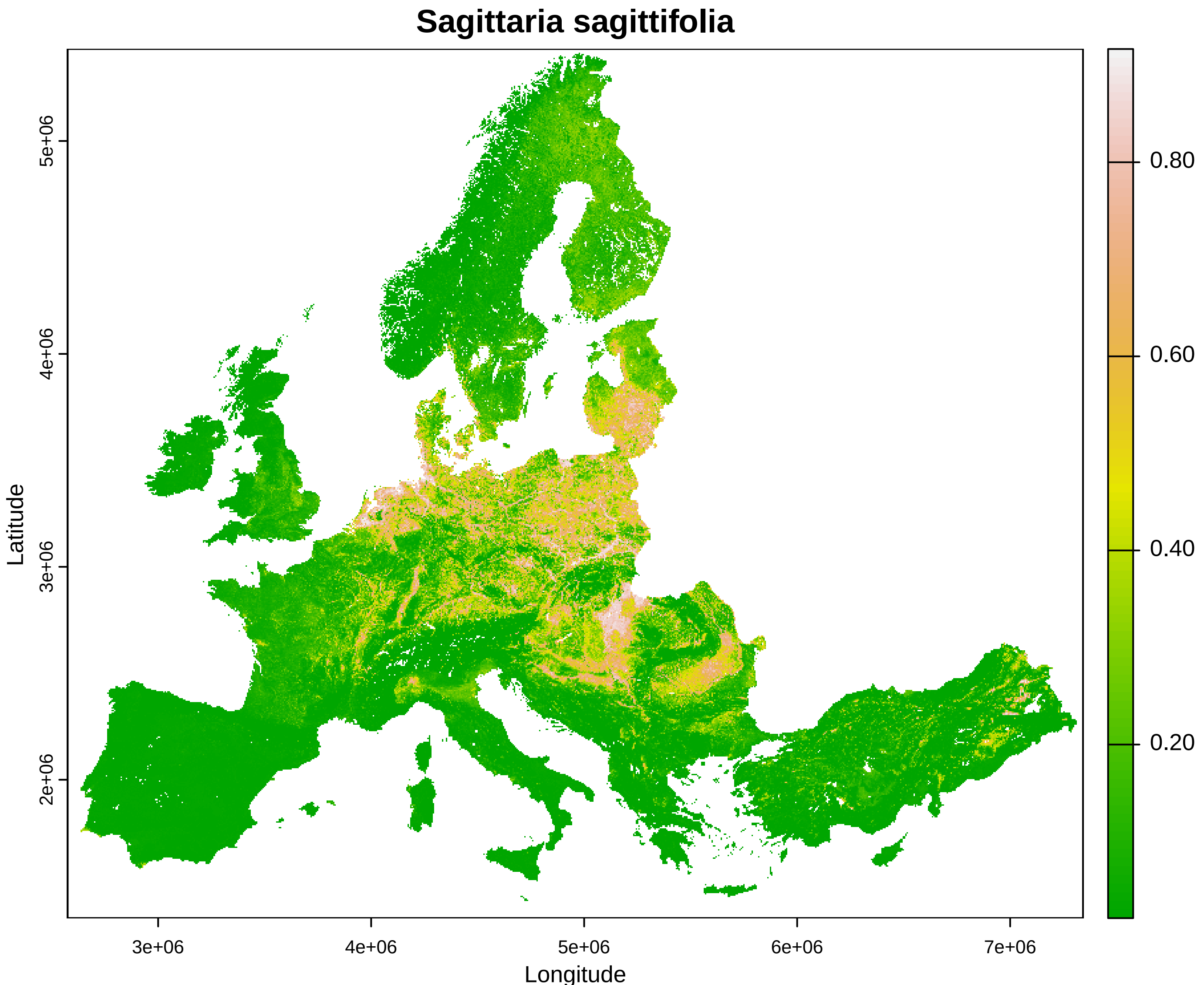

Sagittaria sagittifolia
Latitude
5e+06
4e+06
3e+06
2e+06
Longitude
3e+06
4e+06
5e+06
6e+06
7e+06
0.80
0.60
0.40
0.20

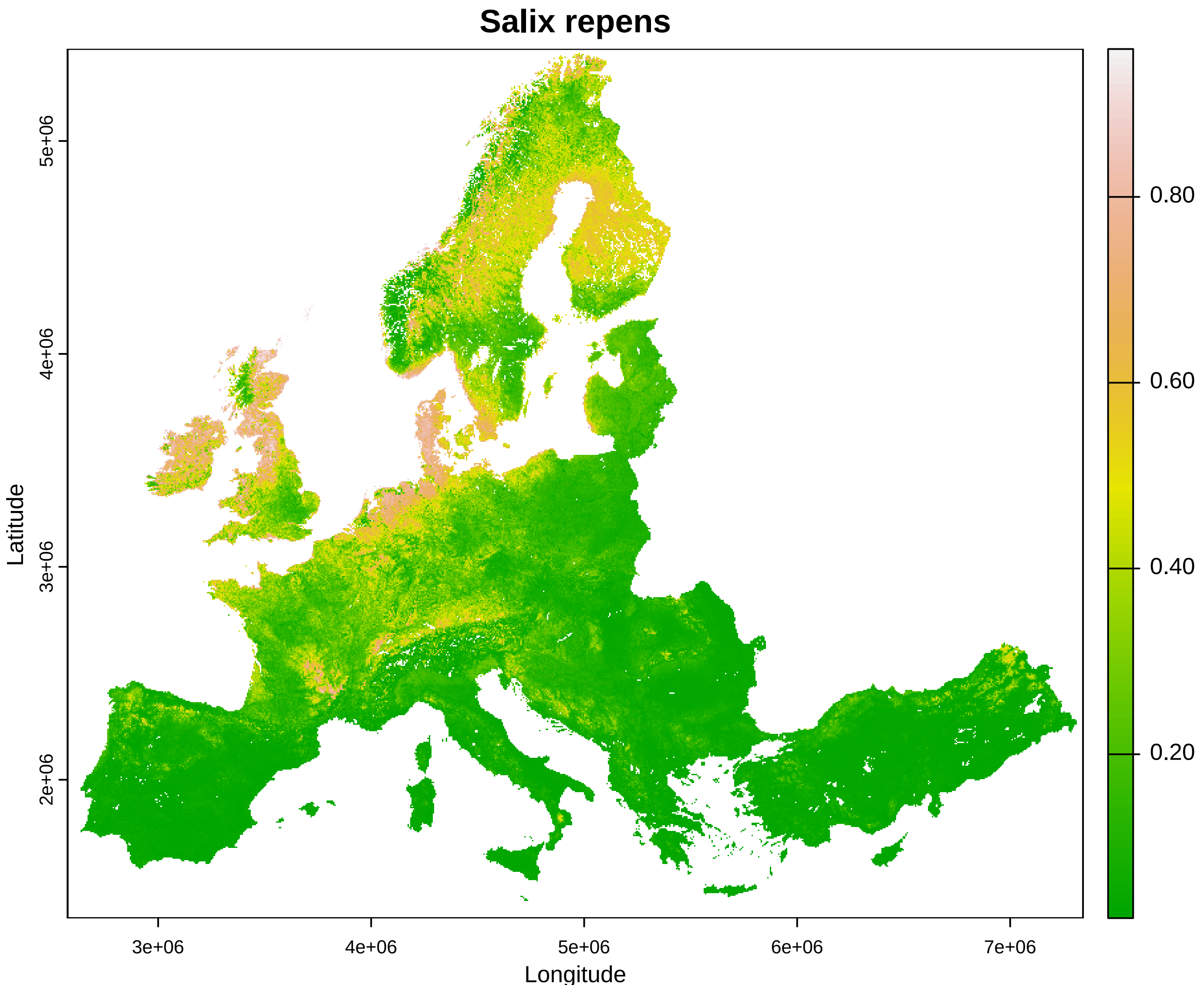

Salix repens
Latitude
5e+06
4e+06
3e+06
2e+06
3e+06
4e+06
5e+06
6e+06
7e+06
Longitude
0.80
0.60
0.40
0.20

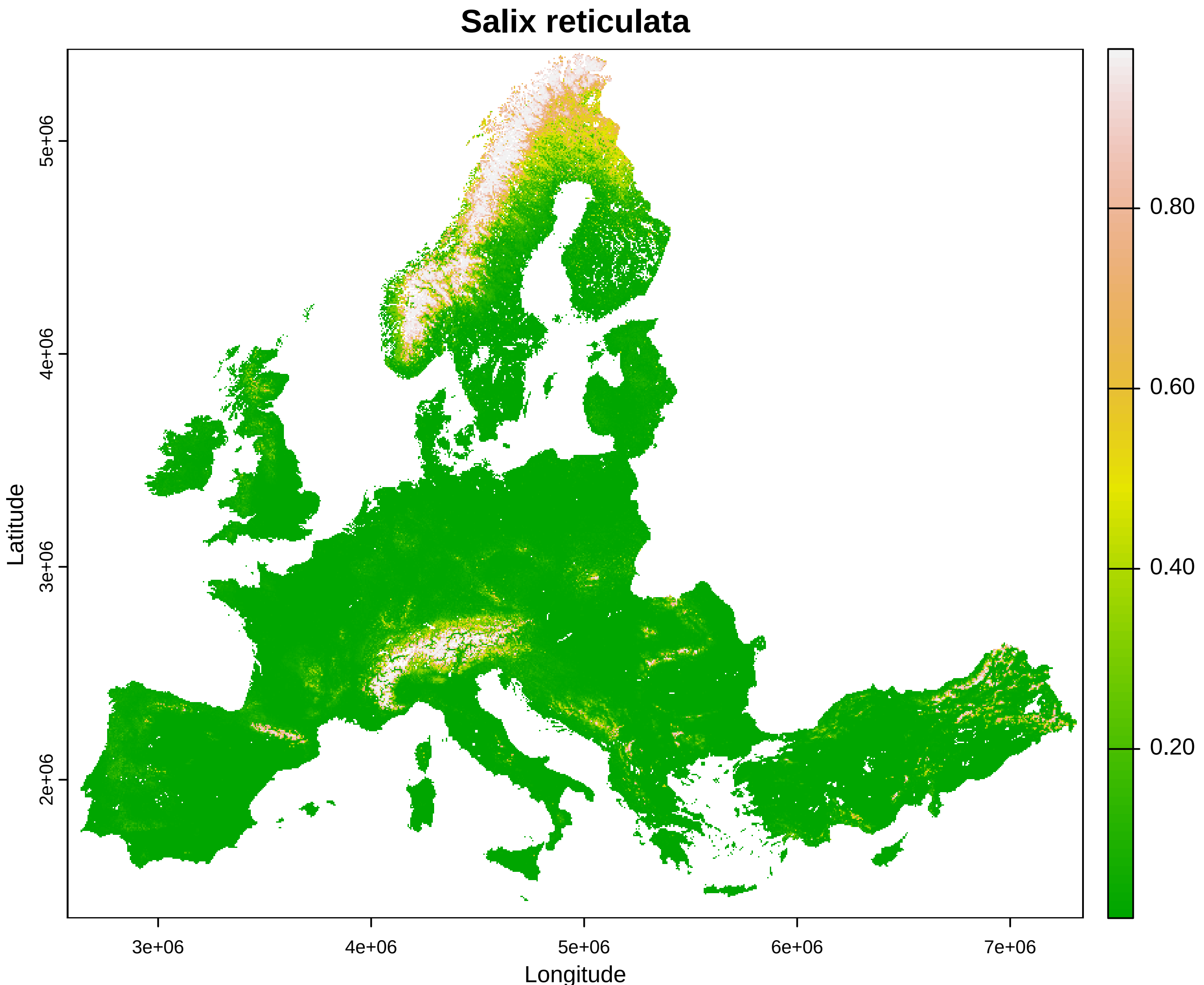
Salix reticulata
Latitude
Longitude
5e+06
4e+06
3e+06
2e+06
3e+06
4e+06
5e+06
6e+06
7e+06
0.80
0.60
0.40
0.20

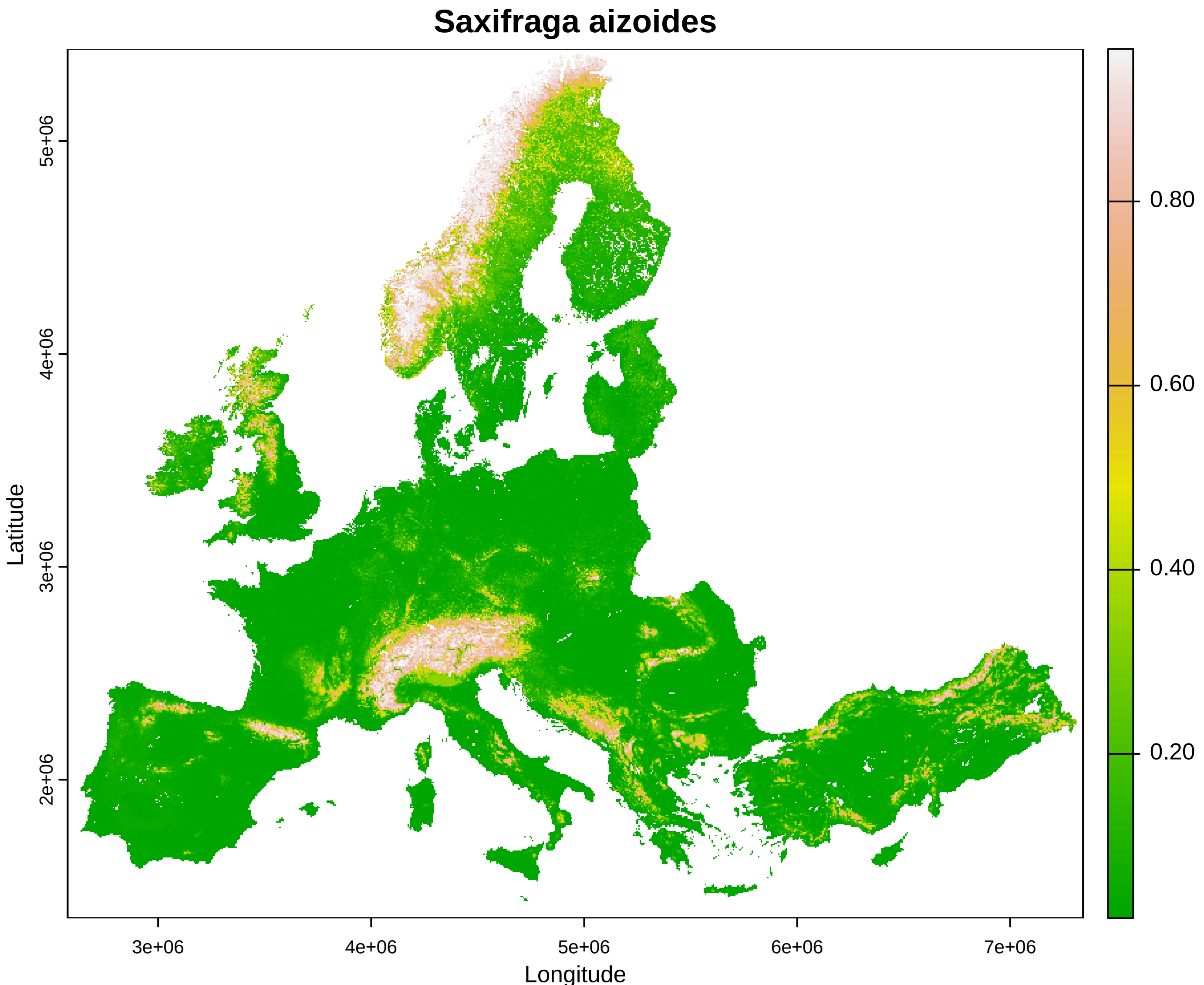
Saxifraga aizoides
Latitude
Longitude
5e+06
4e+06
3e+06
2e+06
3e+06
4e+06
5e+06
6e+06
7e+06
0.80
0.60
0.40
0.20

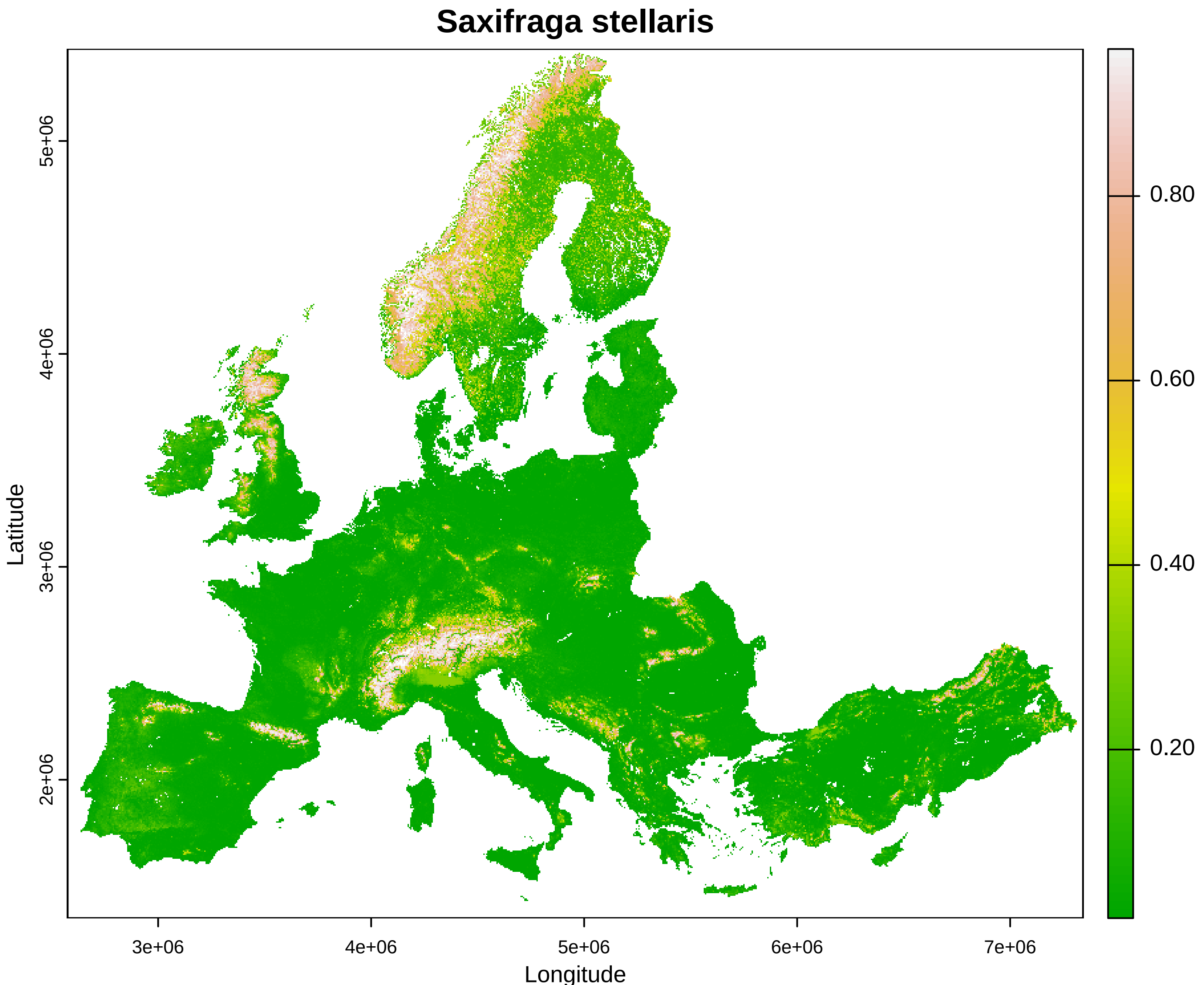
Saxifraga stellaris
Latitude
5e+06
4e+06
3e+06
2e+06
Longitude
3e+06
4e+06
5e+06
6e+06
7e+06
0.80
0.60
0.40
0.20

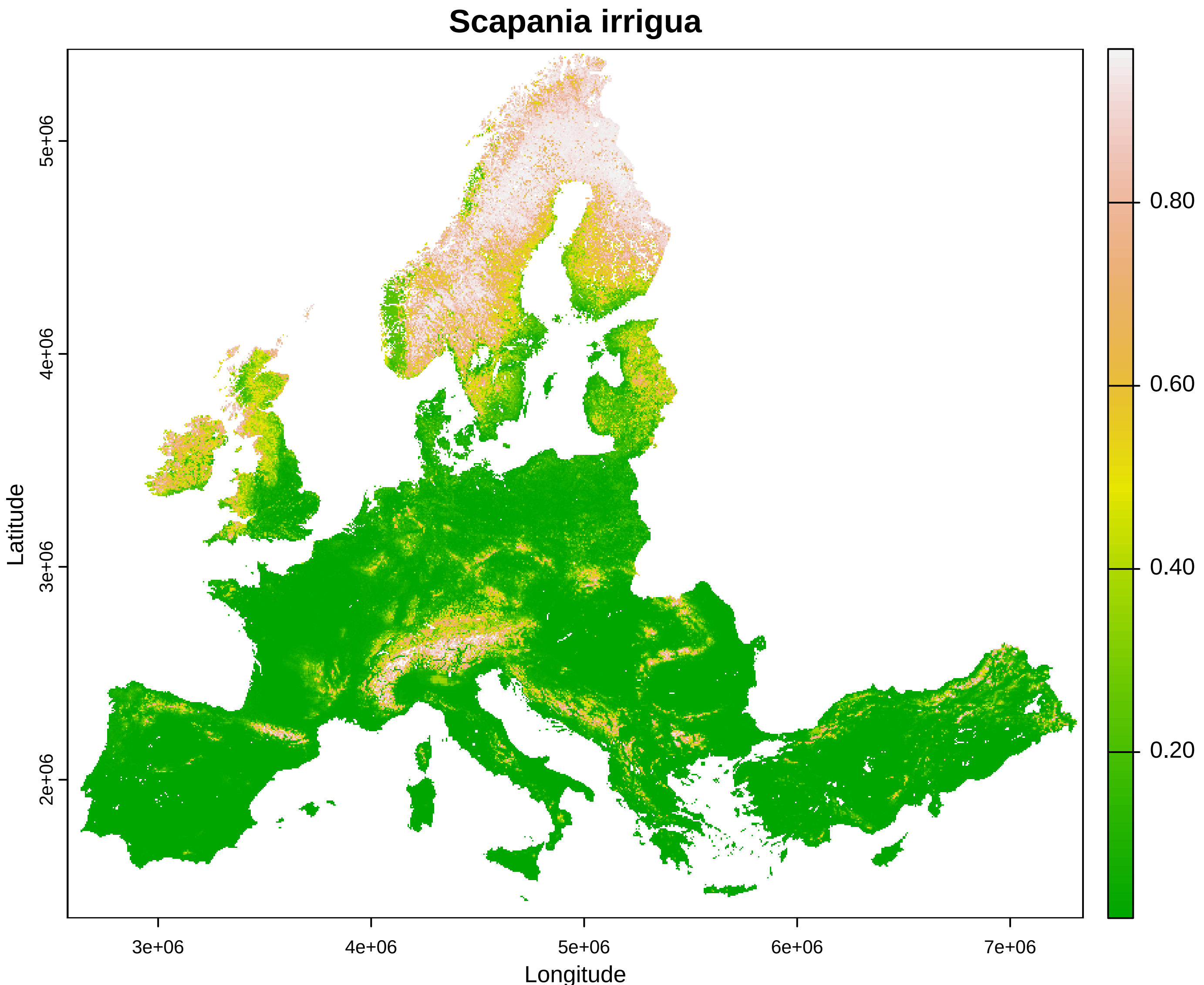

Scapania irrigua
Latitude
Longitude
5e+06
4e+06
3e+06
2e+06
3e+06
4e+06
5e+06
6e+06
7e+06
0.80
0.60
0.40
0.20

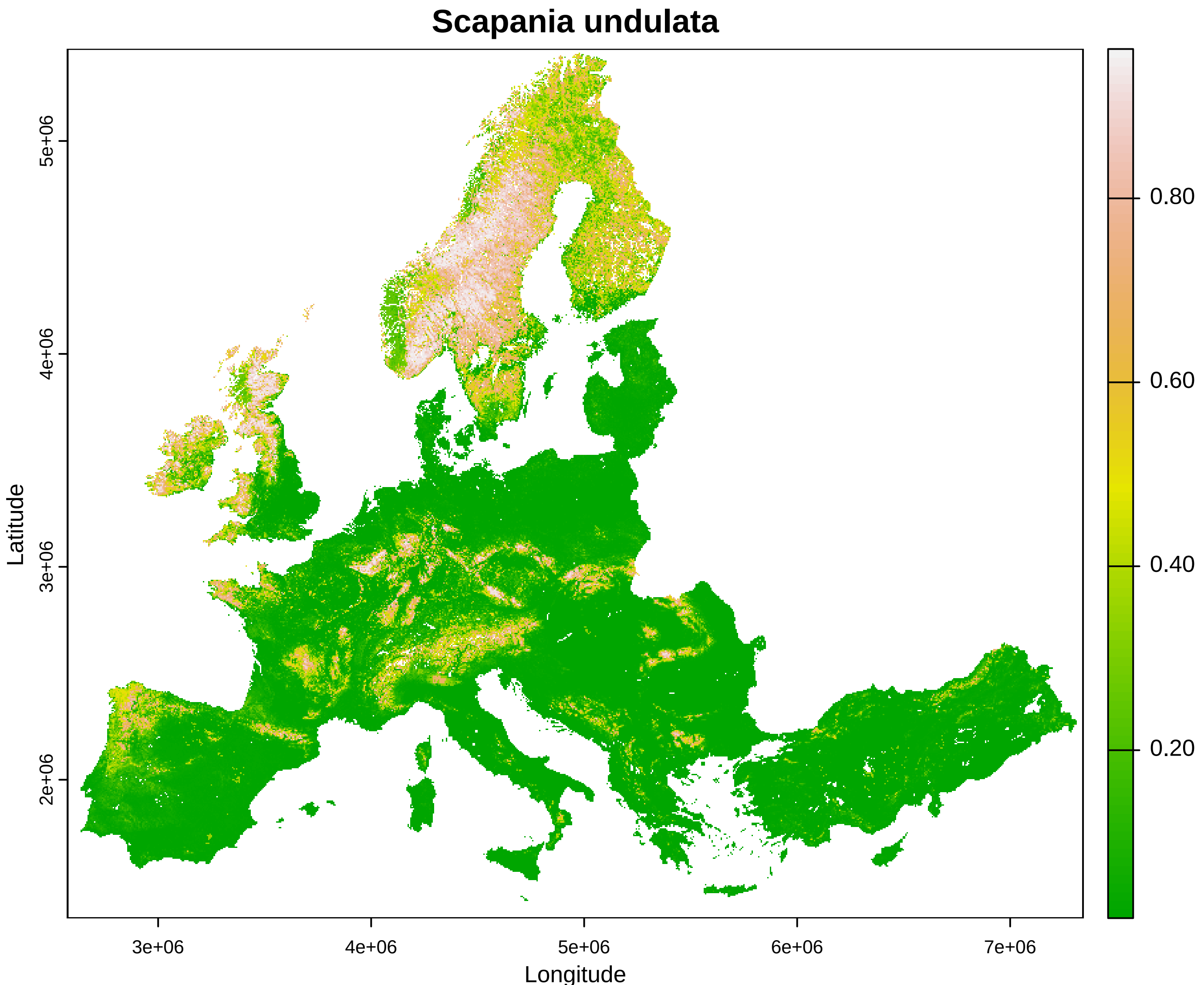

Scapania undulata
Latitude
Longitude
5e+06
4e+06
3e+06
2e+06
3e+06
4e+06
5e+06
6e+06
7e+06
0.80
0.60
0.40
0.20

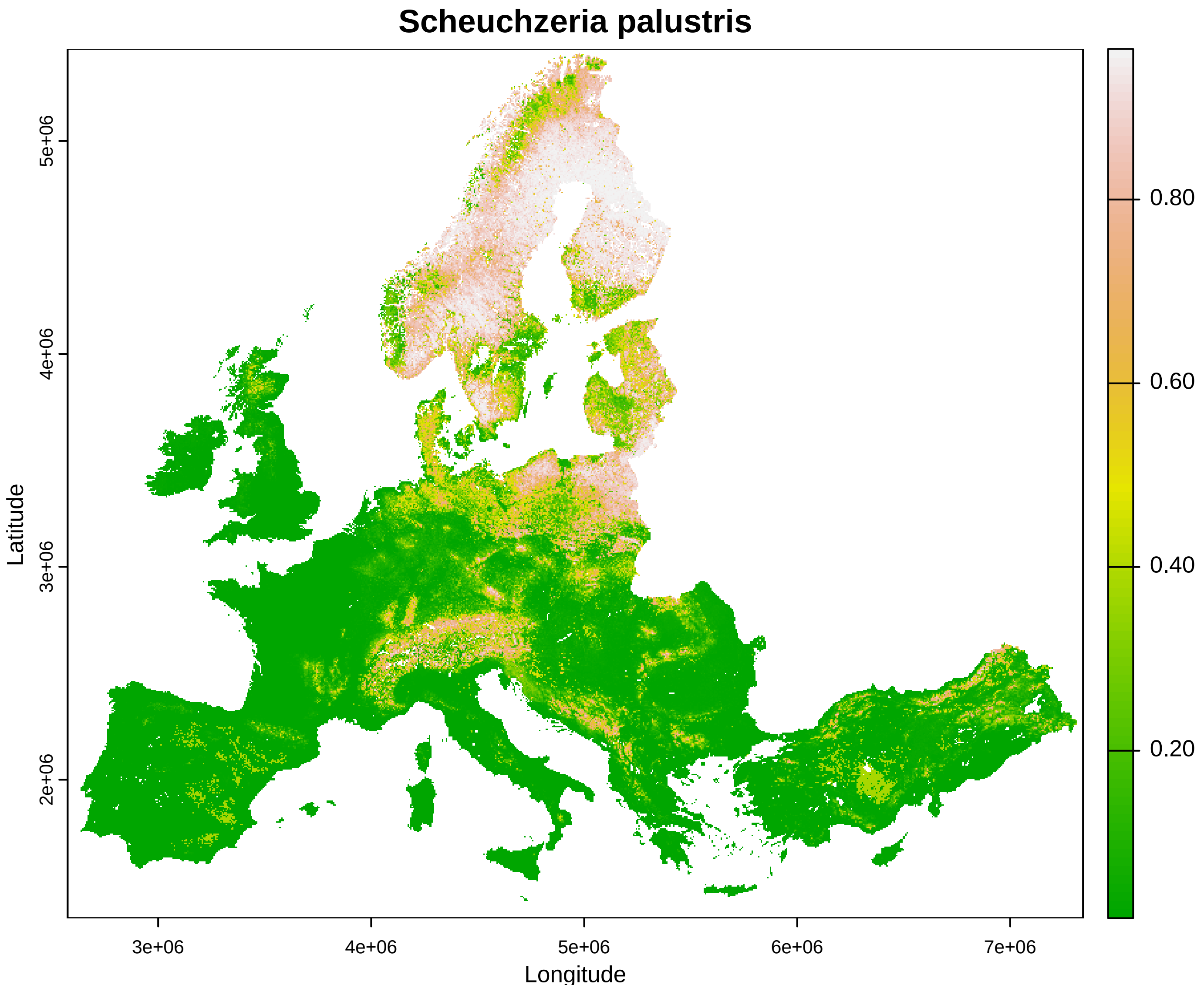
Scheuchzeria palustris
Latitude
Longitude
5e+06
4e+06
3e+06
2e+06
3e+06
4e+06
5e+06
6e+06
7e+06
0.80
0.60
0.40
0.20

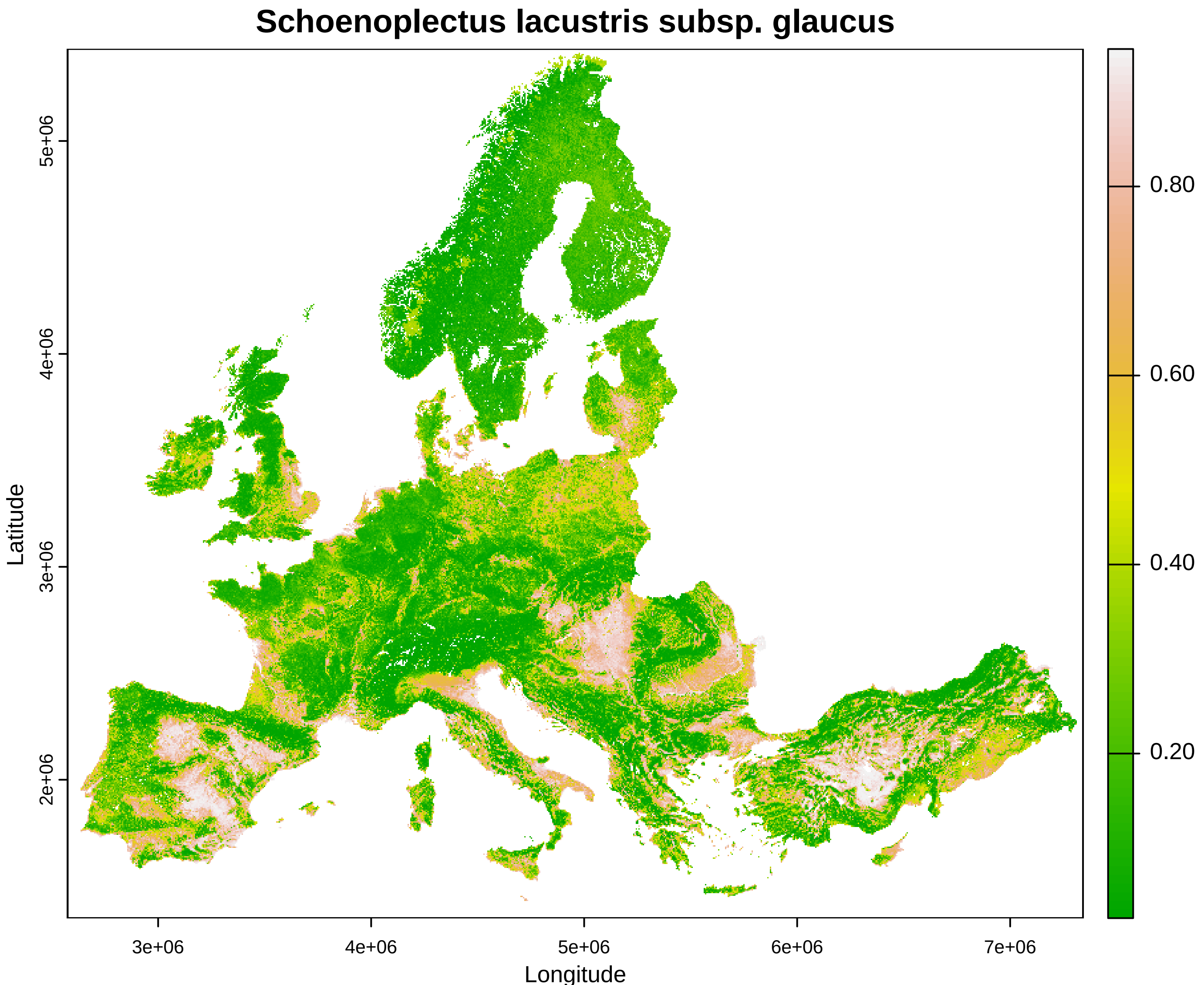
Schoenoplectus lacustris subsp. glaucus
Latitude
5e+06
4e+06
3e+06
2e+06
3e+06
4e+06
5e+06
6e+06
7e+06
Longitude
0.80
0.60
0.40
0.20

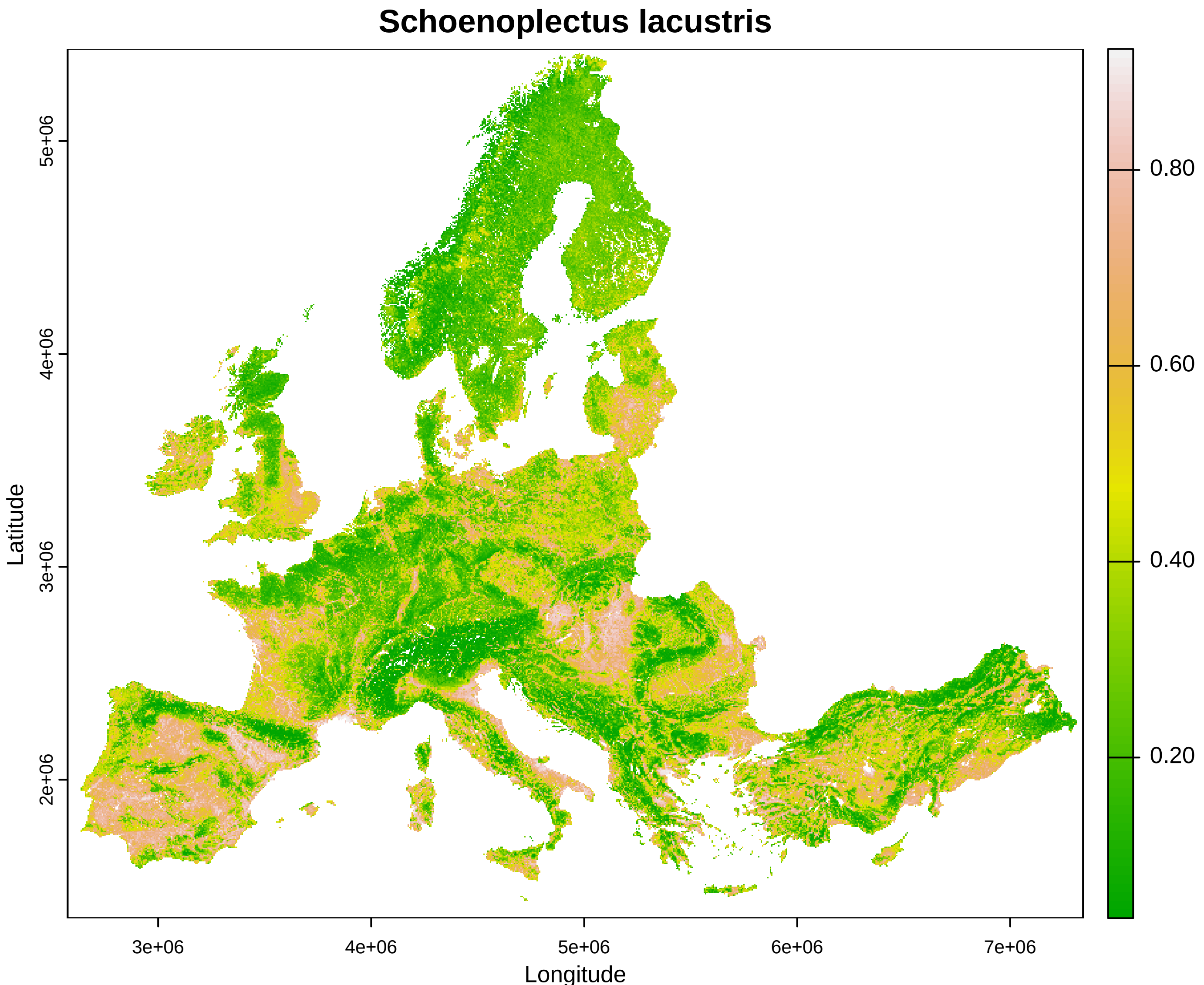

Schoenoplectus lacustris
Latitude
Longitude
5e+06
4e+06
3e+06
2e+06
3e+06
4e+06
5e+06
6e+06
7e+06
0.80
0.60
0.40
0.20

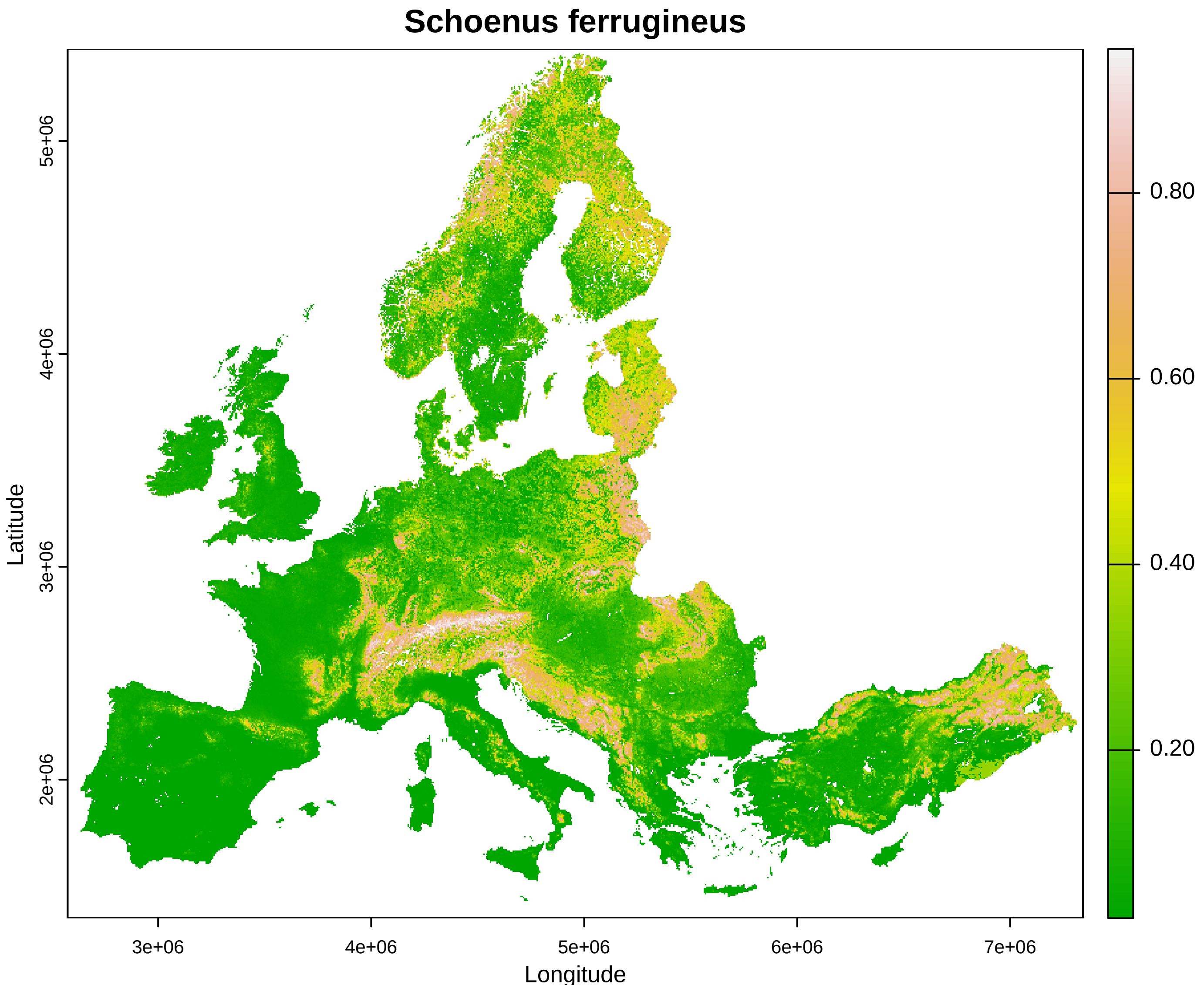
Schoenus ferrugineus
Latitude
Longitude
5e+06
4e+06
3e+06
2e+06
3e+06
4e+06
5e+06
6e+06
7e+06
0.80
0.60
0.40
0.20

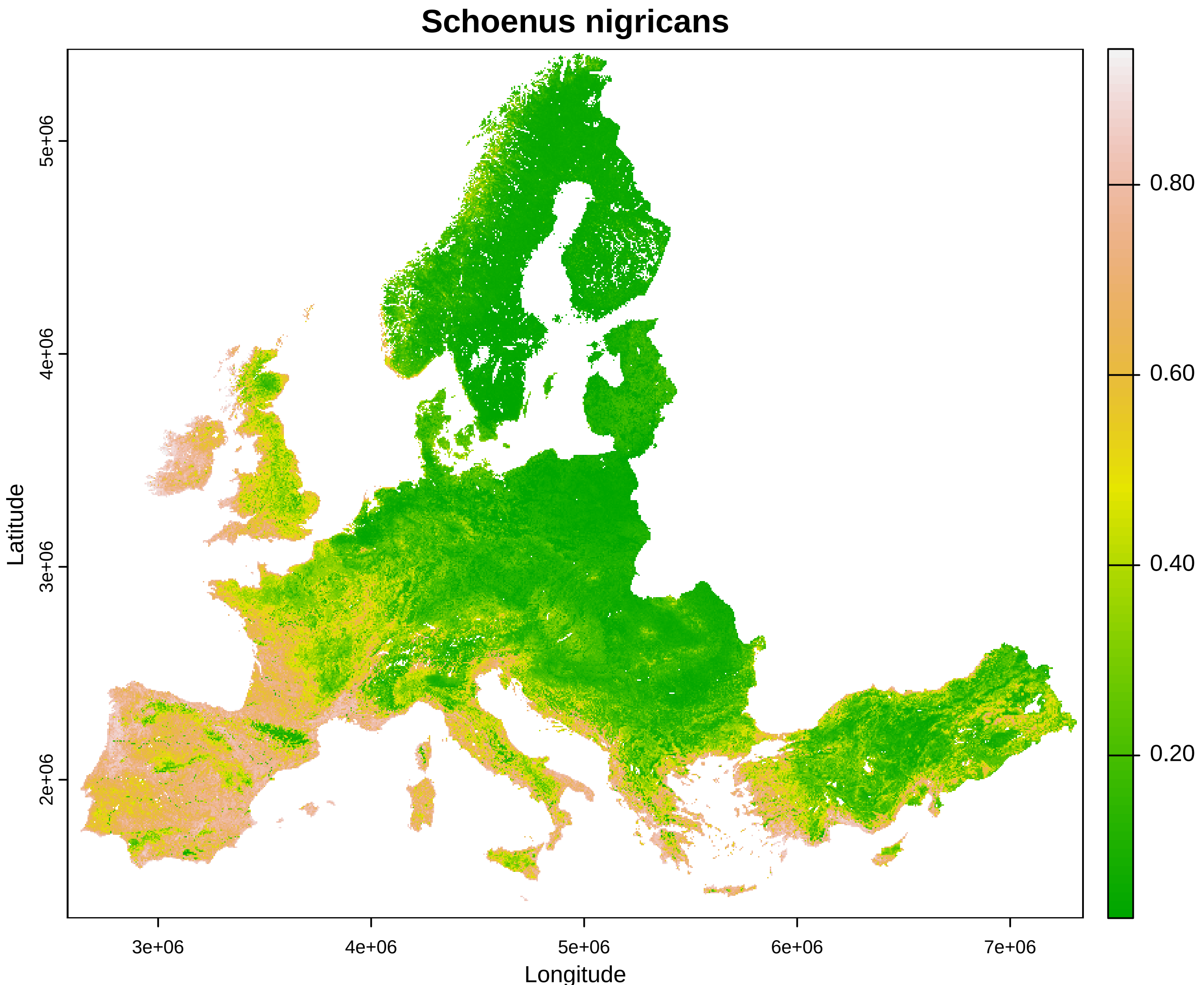
Schoenus nigricans
Latitude
5e+06
4e+06
3e+06
2e+06
Longitude
3e+06
4e+06
5e+06
6e+06
7e+06
0.80
0.60
0.40
0.20

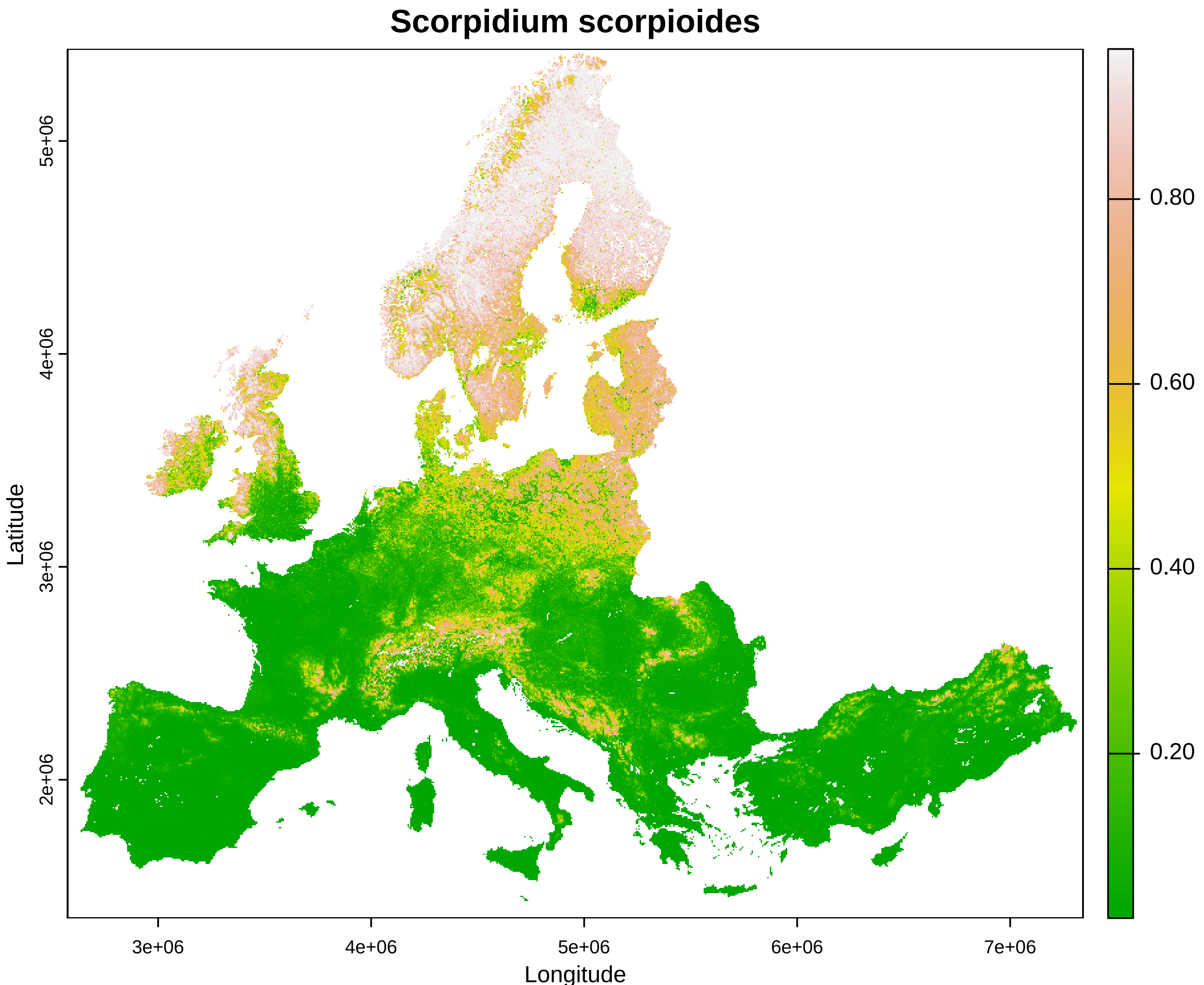
Scorpidium scorpioides
Latitude
Longitude
5e+06
4e+06
3e+06
2e+06
3e+06
4e+06
5e+06
6e+06
7e+06
0.80
0.60
0.40
0.20

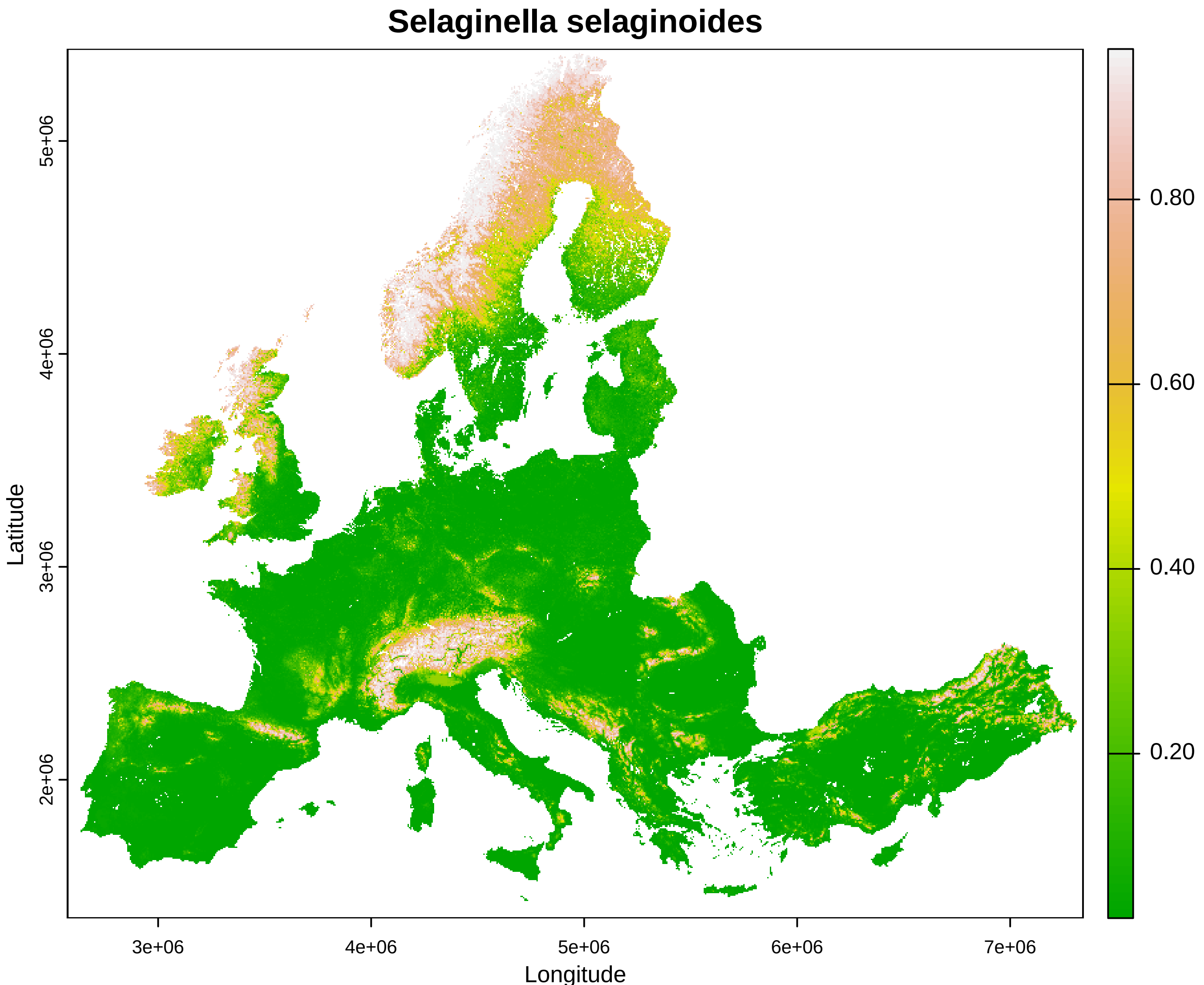
Selaginella selaginoides
Latitude
Longitude
5e+06
4e+06
3e+06
2e+06
3e+06
4e+06
5e+06
6e+06
7e+06
0.80
0.60
0.40
0.20

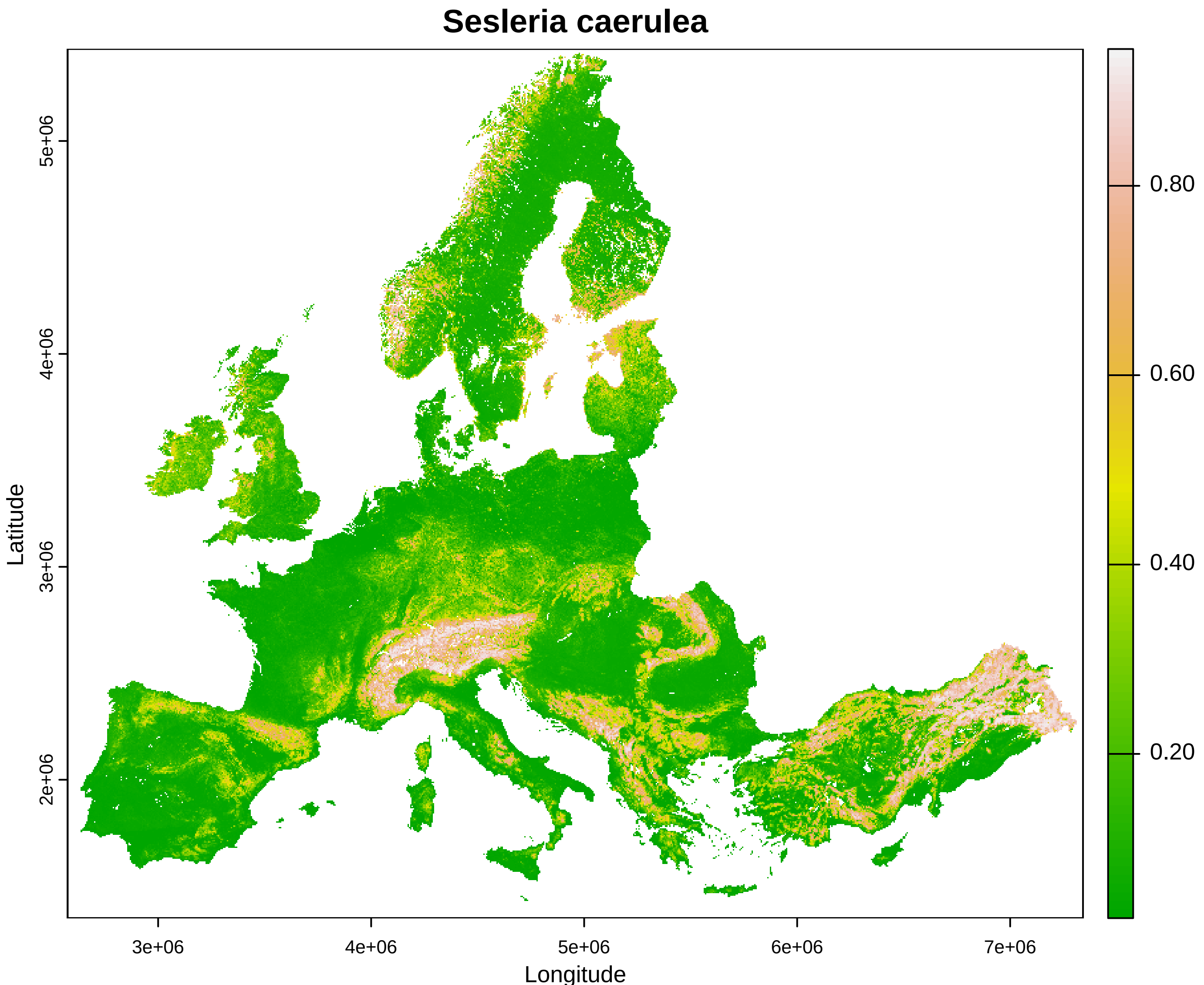

Sesleria caerulea
Latitude
5e+06
4e+06
3e+06
2e+06
3e+06
4e+06
5e+06
6e+06
7e+06
Longitude
0.80
0.60
0.40
0.20

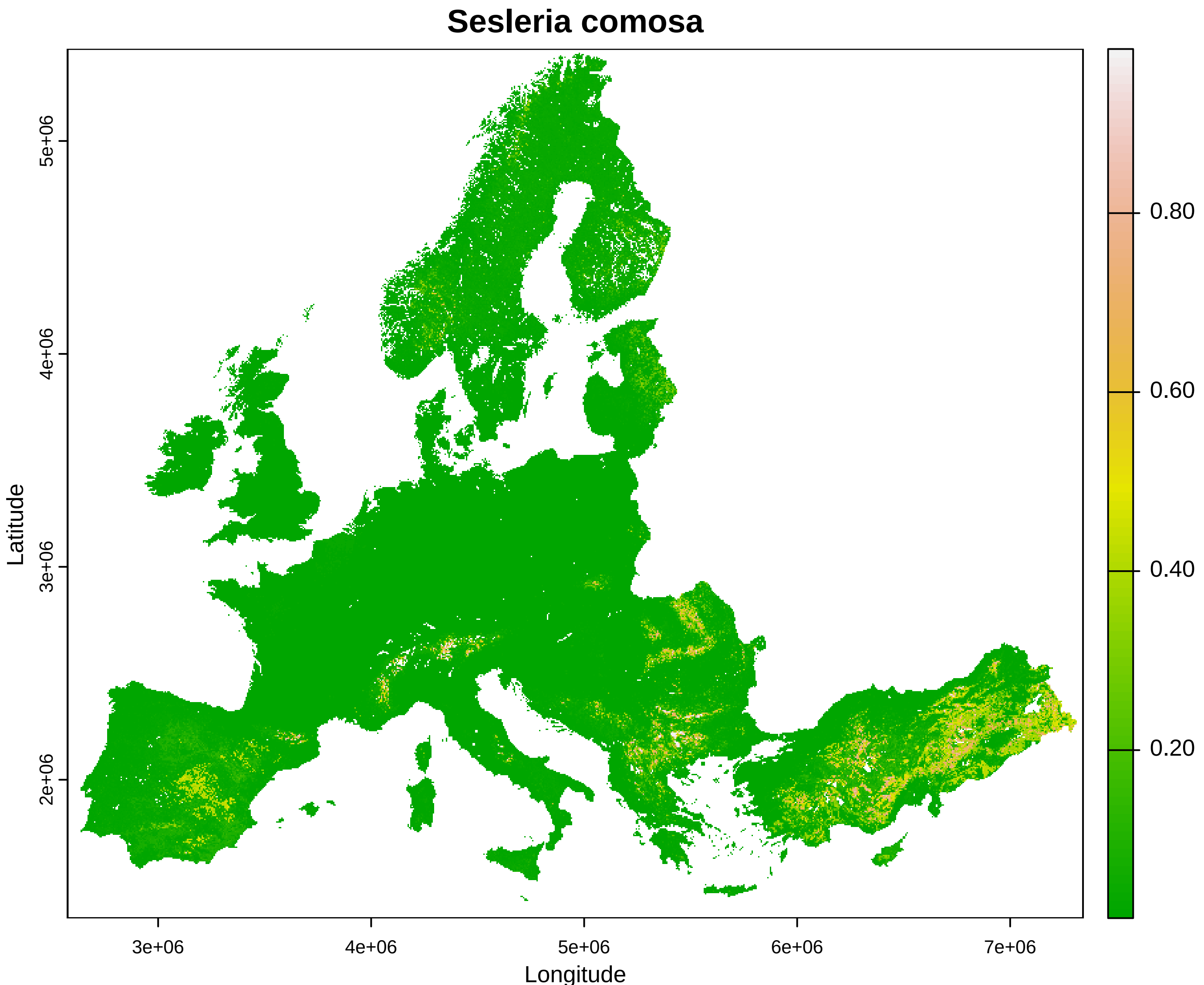

Sesleria comosa
Latitude
Longitude
5e+06
4e+06
3e+06
2e+06
3e+06
4e+06
5e+06
6e+06
7e+06
0.80
0.60
0.40
0.20

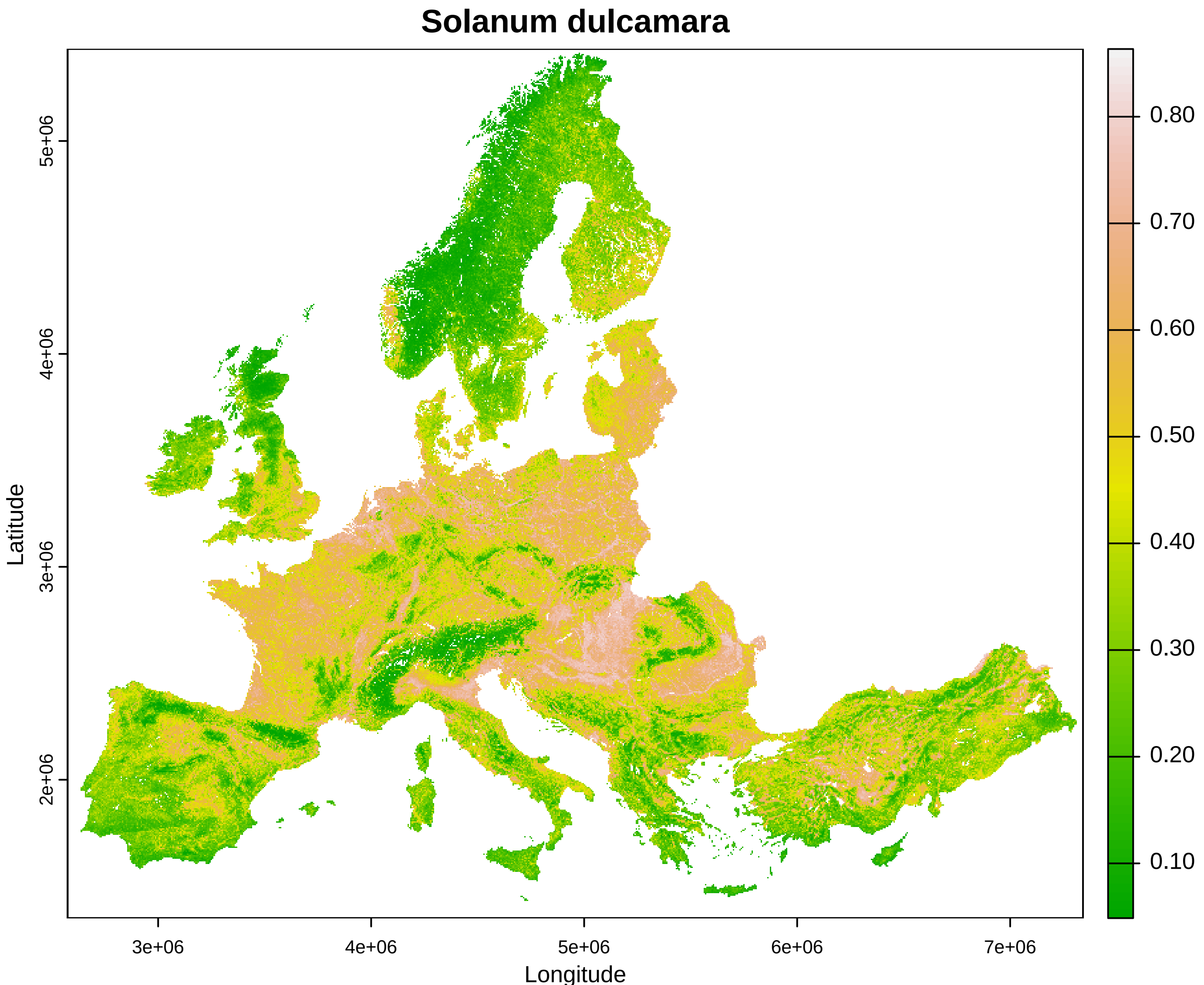

Solanum dulcamara
Latitude
Longitude
5e+06
4e+06
3e+06
2e+06
3e+06
4e+06
5e+06
6e+06
7e+06
0.80
0.70
0.60
0.50
0.40
0.30
0.20
0.10

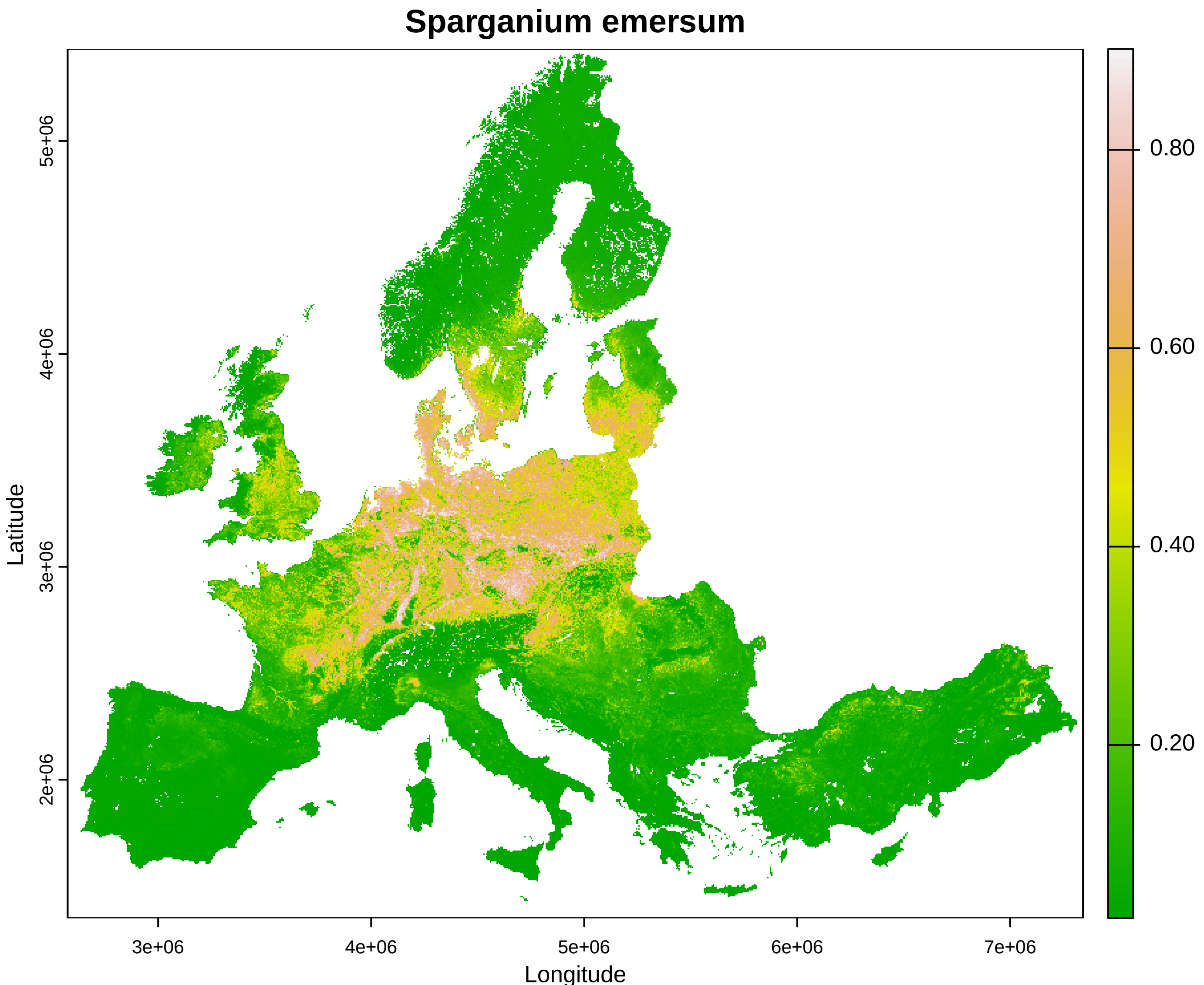

Sparganium emersum
Latitude
Longitude
5e+06
4e+06
3e+06
2e+06
3e+06
4e+06
5e+06
6e+06
7e+06
0.80
0.60
0.40
0.20

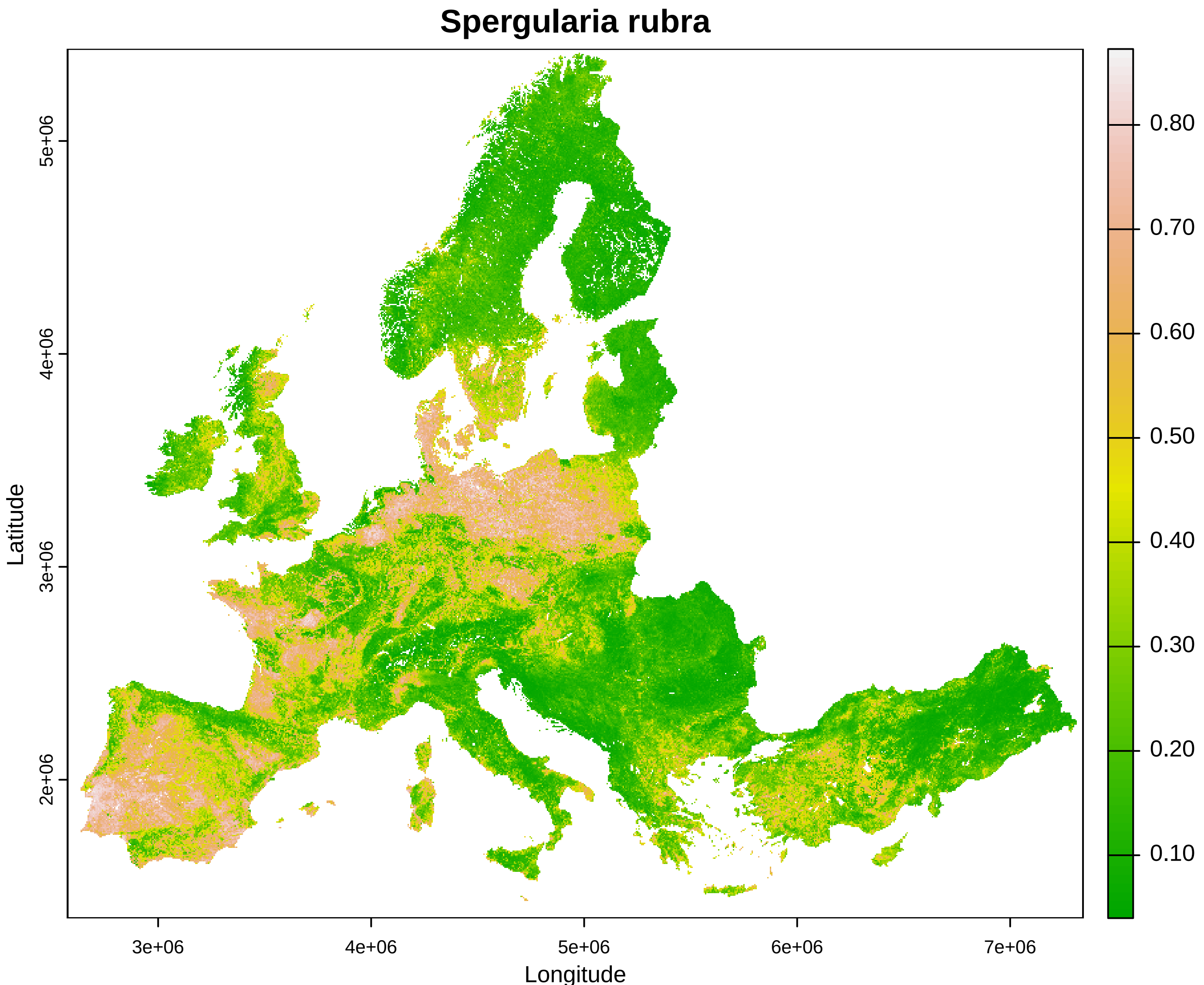
Spergularia rubra
Latitude
Longitude
5e+06
4e+06
3e+06
2e+06
3e+06
4e+06
5e+06
6e+06
7e+06
0.80
0.70
0.60
0.50
0.40
0.30
0.20
0.10

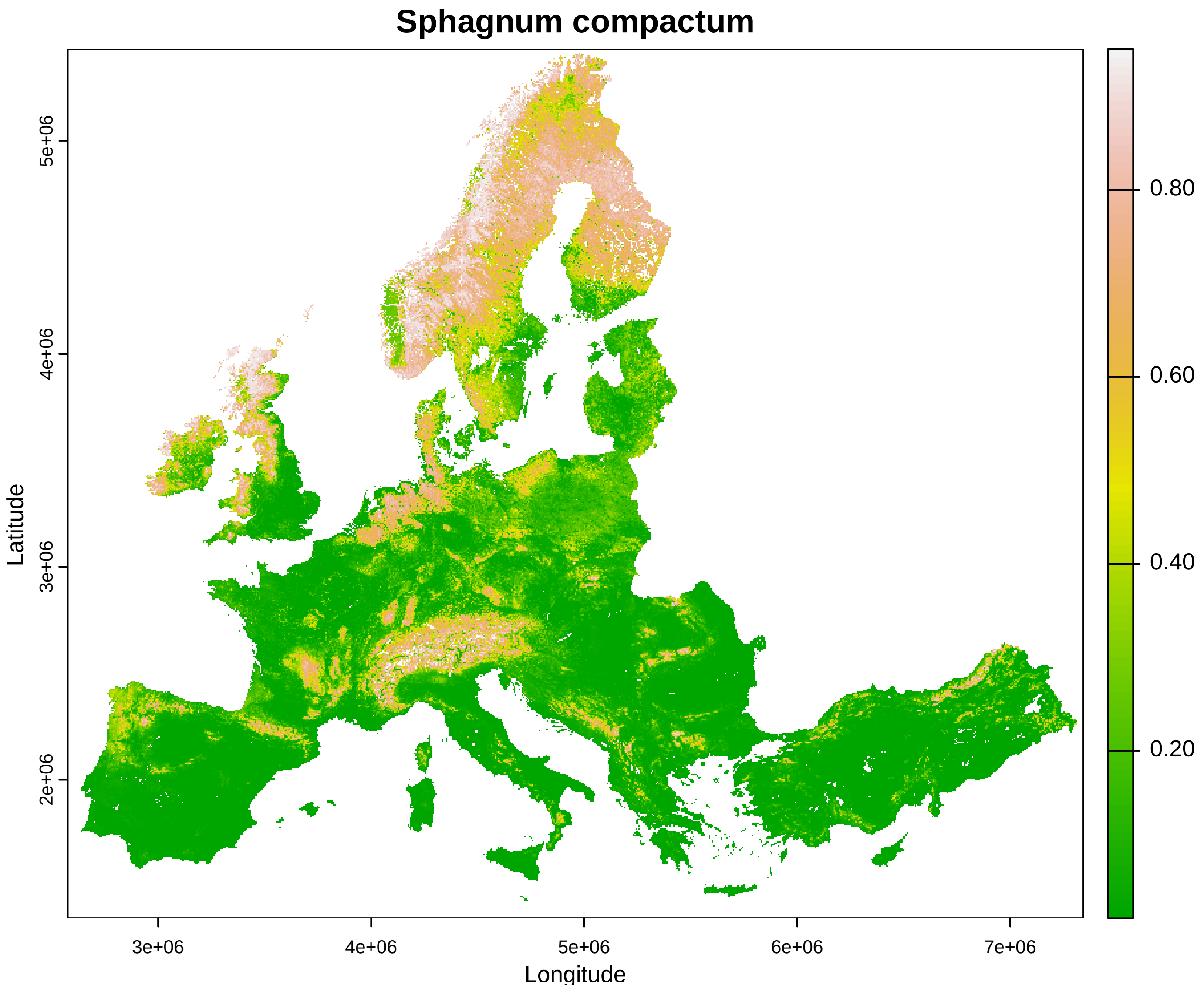
Sphagnum compactum
Latitude
5e+06
4e+06
3e+06
2e+06
3e+06
4e+06
5e+06
6e+06
7e+06
Longitude
0.80
0.60
0.40
0.20

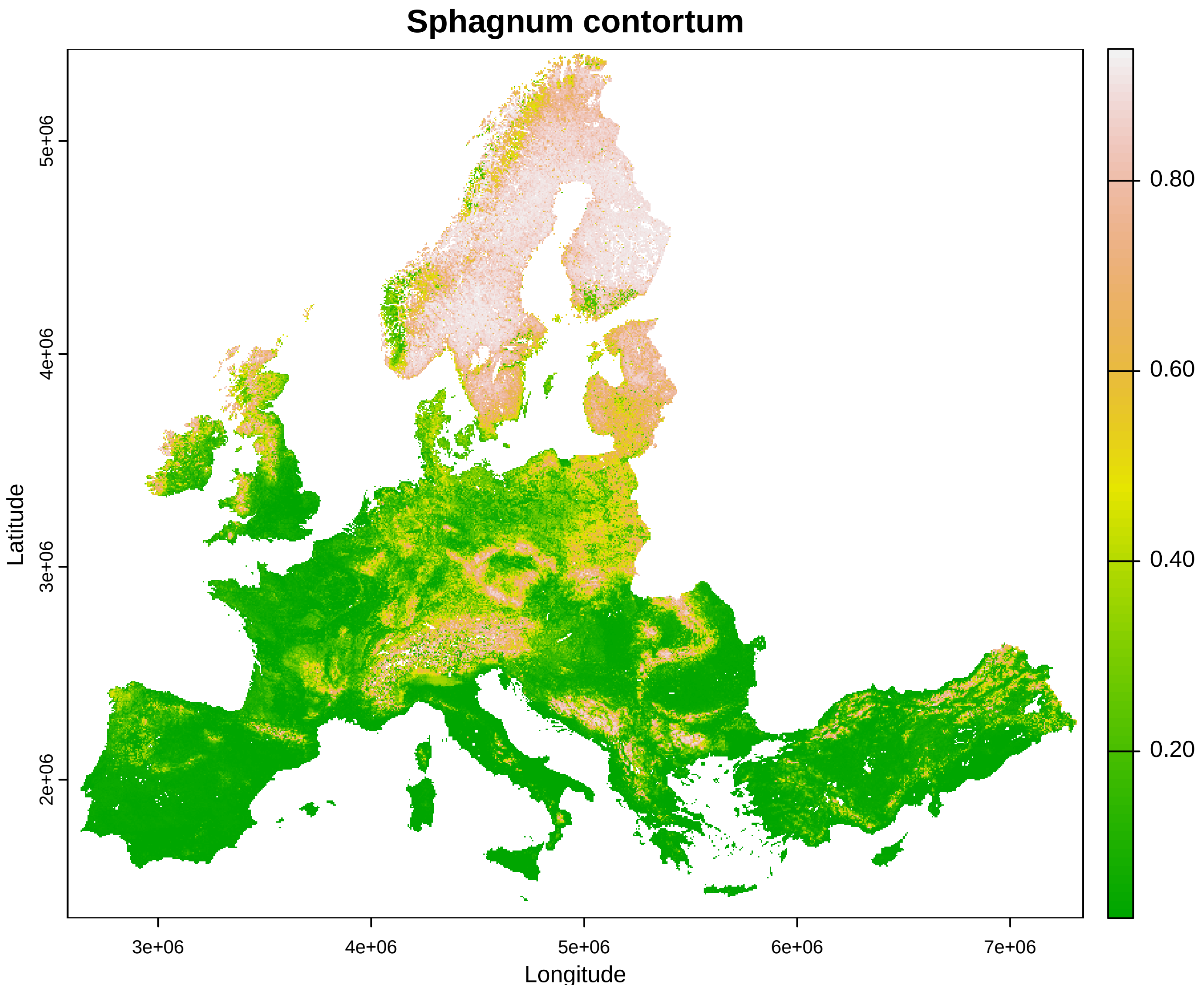
Sphagnum contortum
Latitude
5e+06
4e+06
3e+06
2e+06
Longitude
3e+06
4e+06
5e+06
6e+06
7e+06
0.80
0.60
0.40
0.20

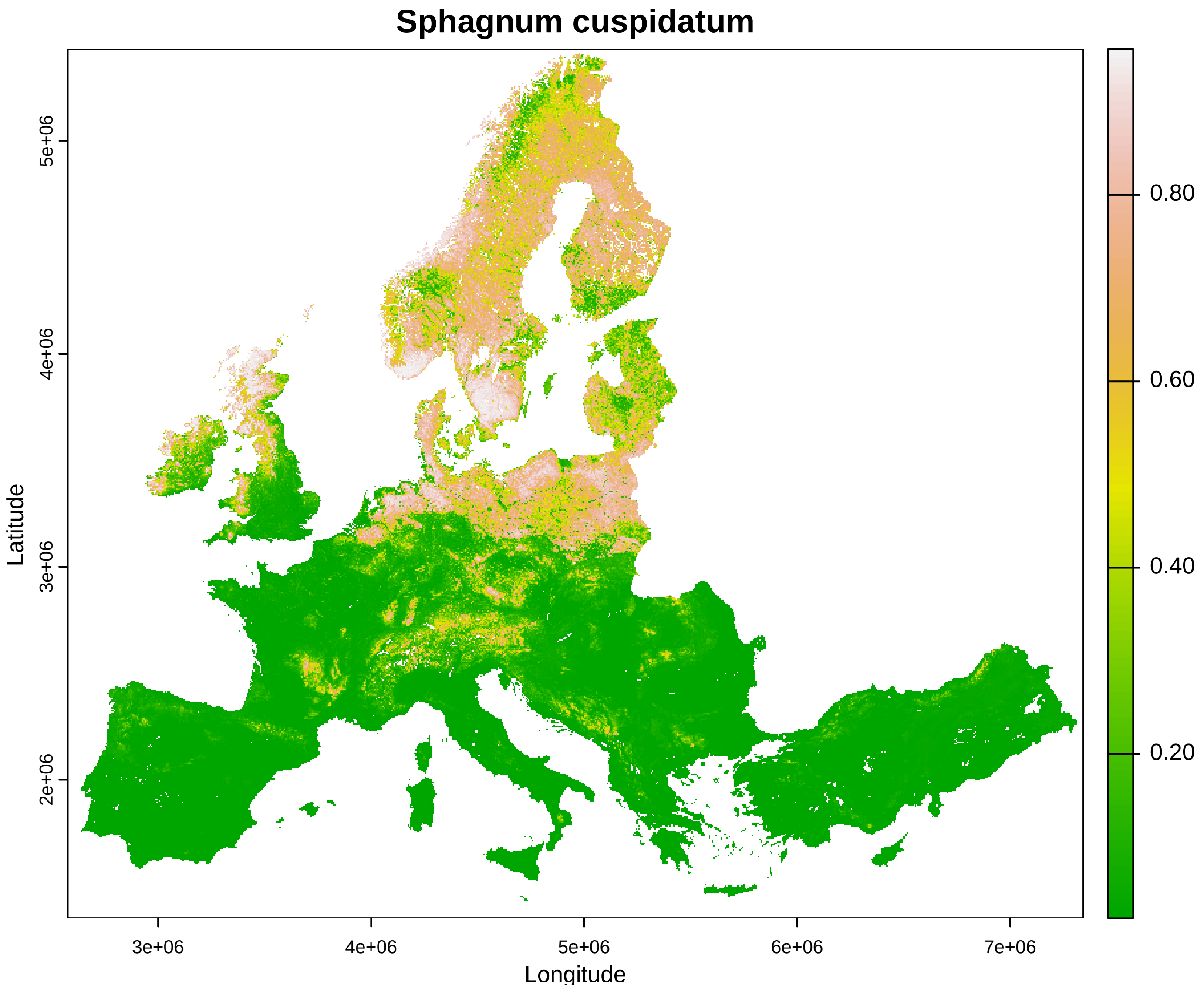
Sphagnum cuspidatum
Latitude
Longitude
5e+06
4e+06
3e+06
2e+06
3e+06
4e+06
5e+06
6e+06
7e+06
0.80
0.60
0.40
0.20

Sphagnum fuscum

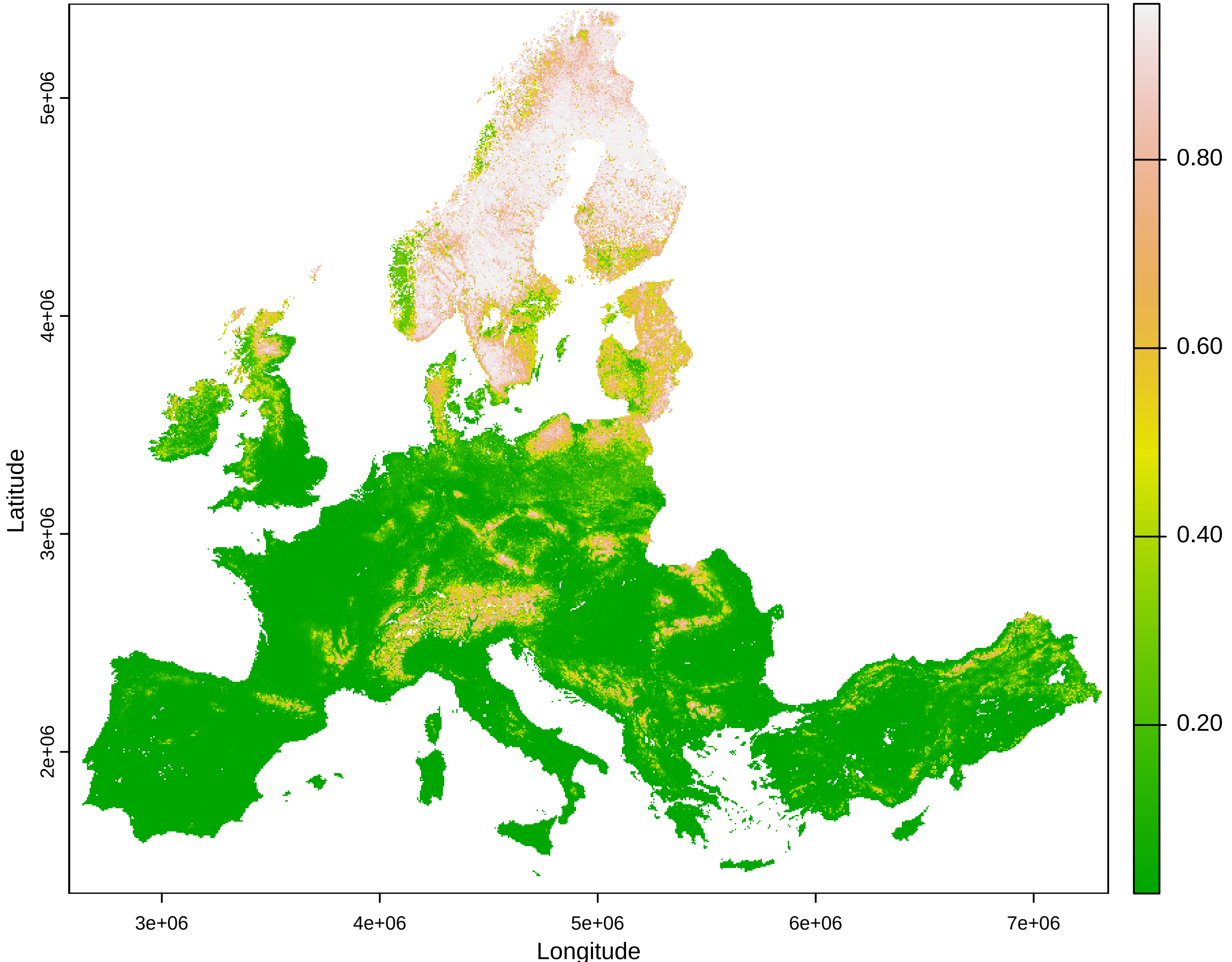

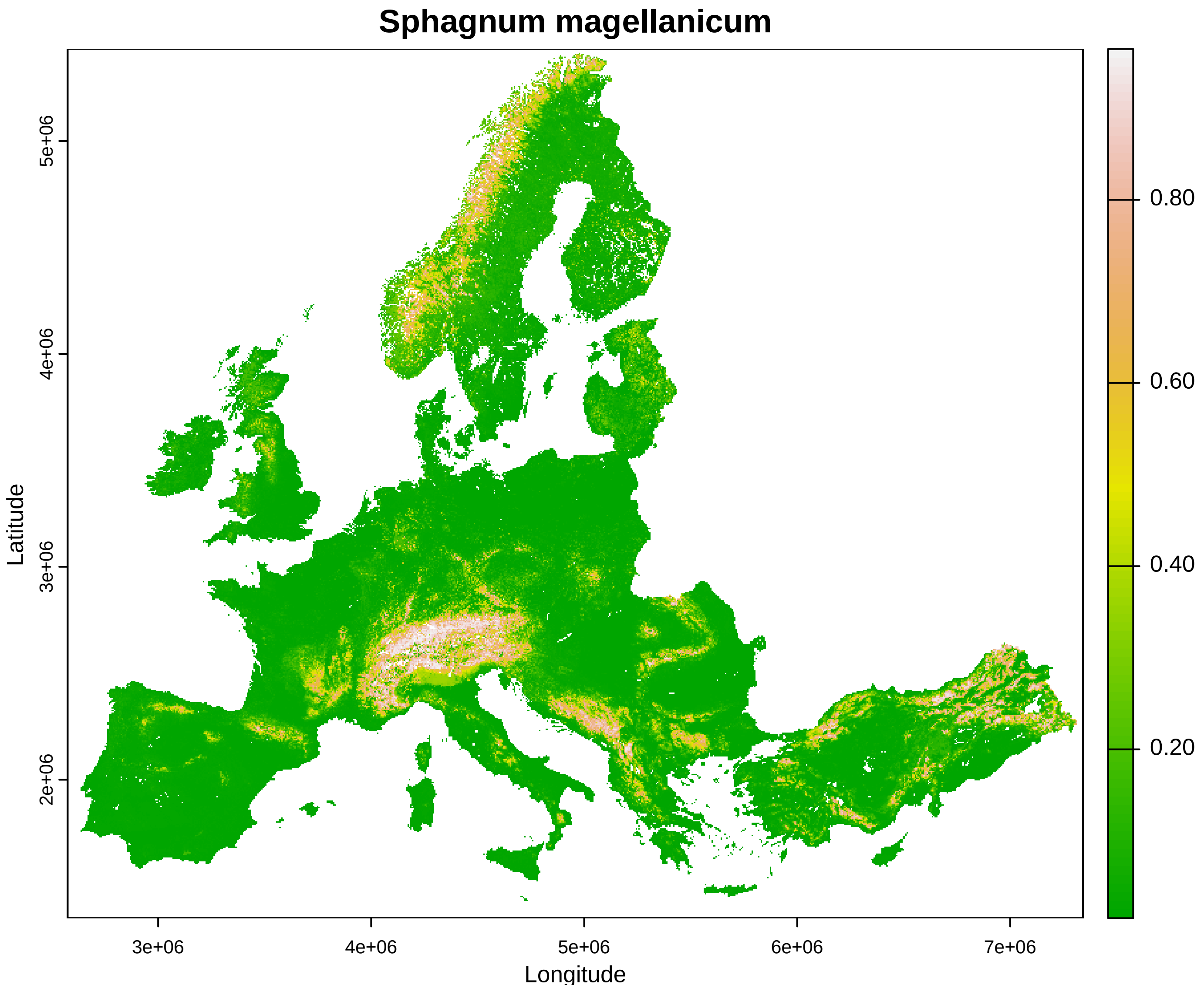
Sphagnum magellanicum
Latitude
5e+06
4e+06
3e+06
2e+06
Longitude
3e+06
4e+06
5e+06
6e+06
7e+06
0.80
0.60
0.40
0.20

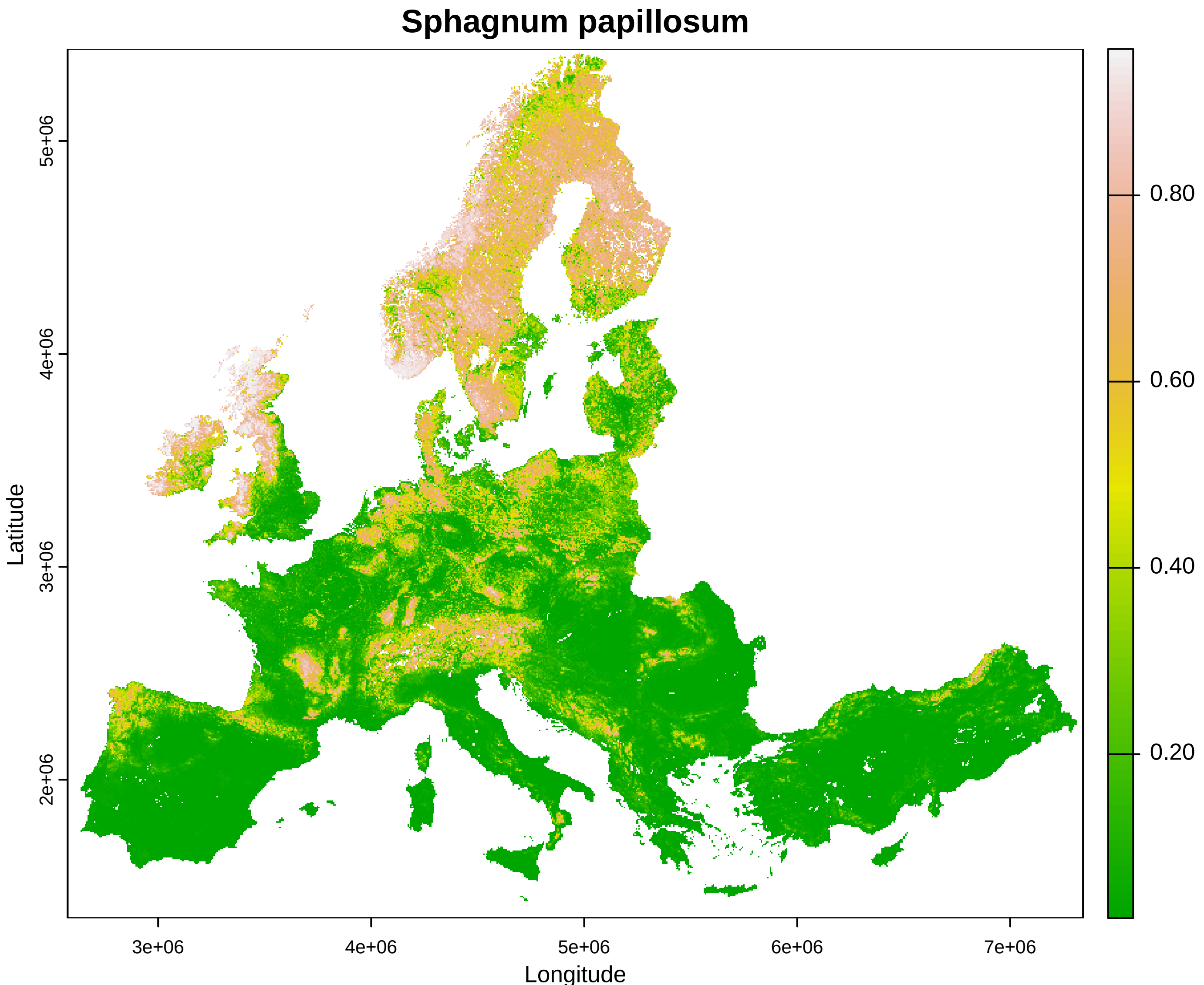

Sphagnum papillosum
Latitude
Longitude
5e+06
4e+06
3e+06
2e+06
3e+06
4e+06
5e+06
6e+06
7e+06
0.80
0.60
0.40
0.20

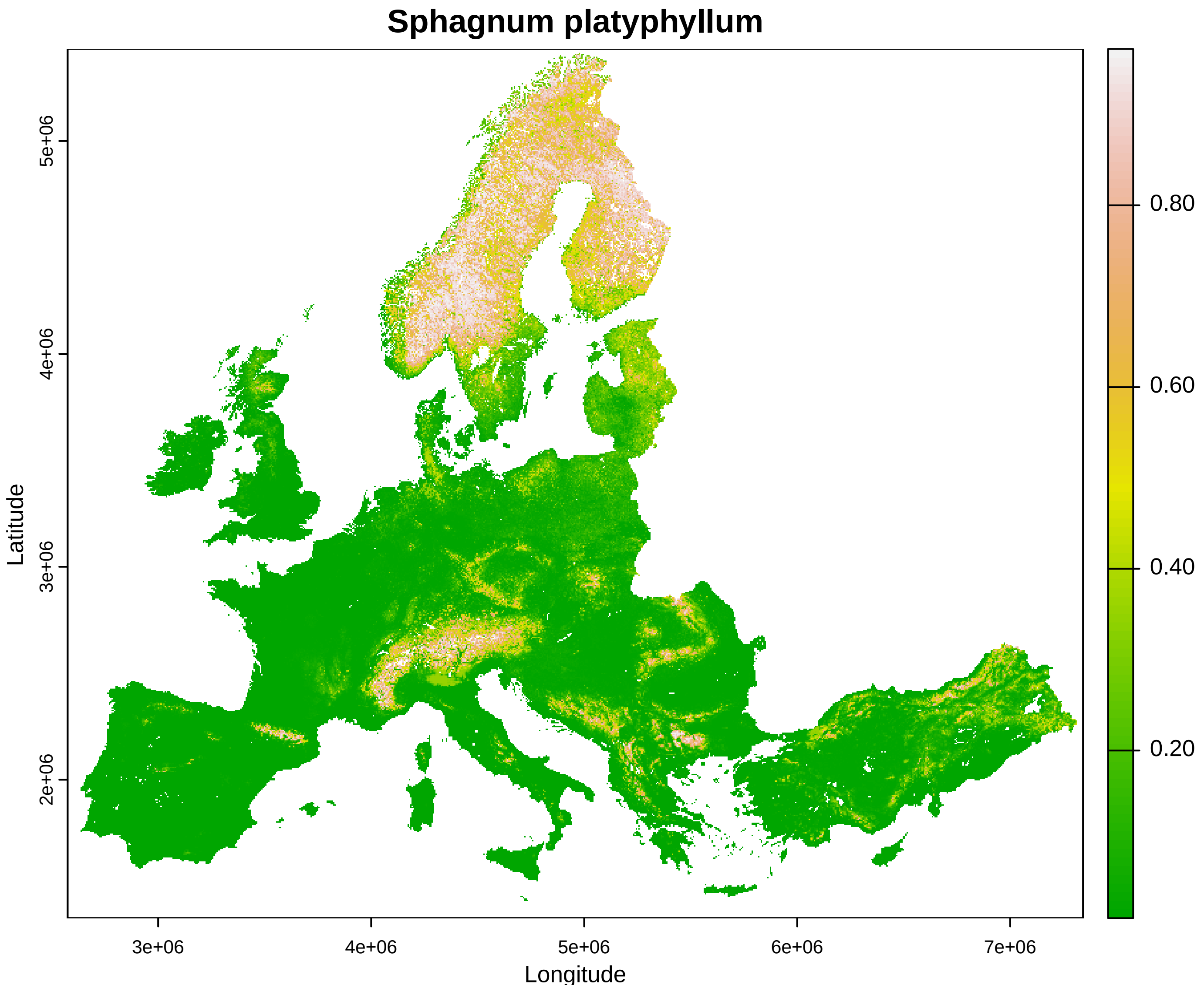

Sphagnum platyphyllum
Latitude
5e+06
4e+06
3e+06
2e+06
Longitude
3e+06
4e+06
5e+06
6e+06
7e+06
0.80
0.60
0.40
0.20

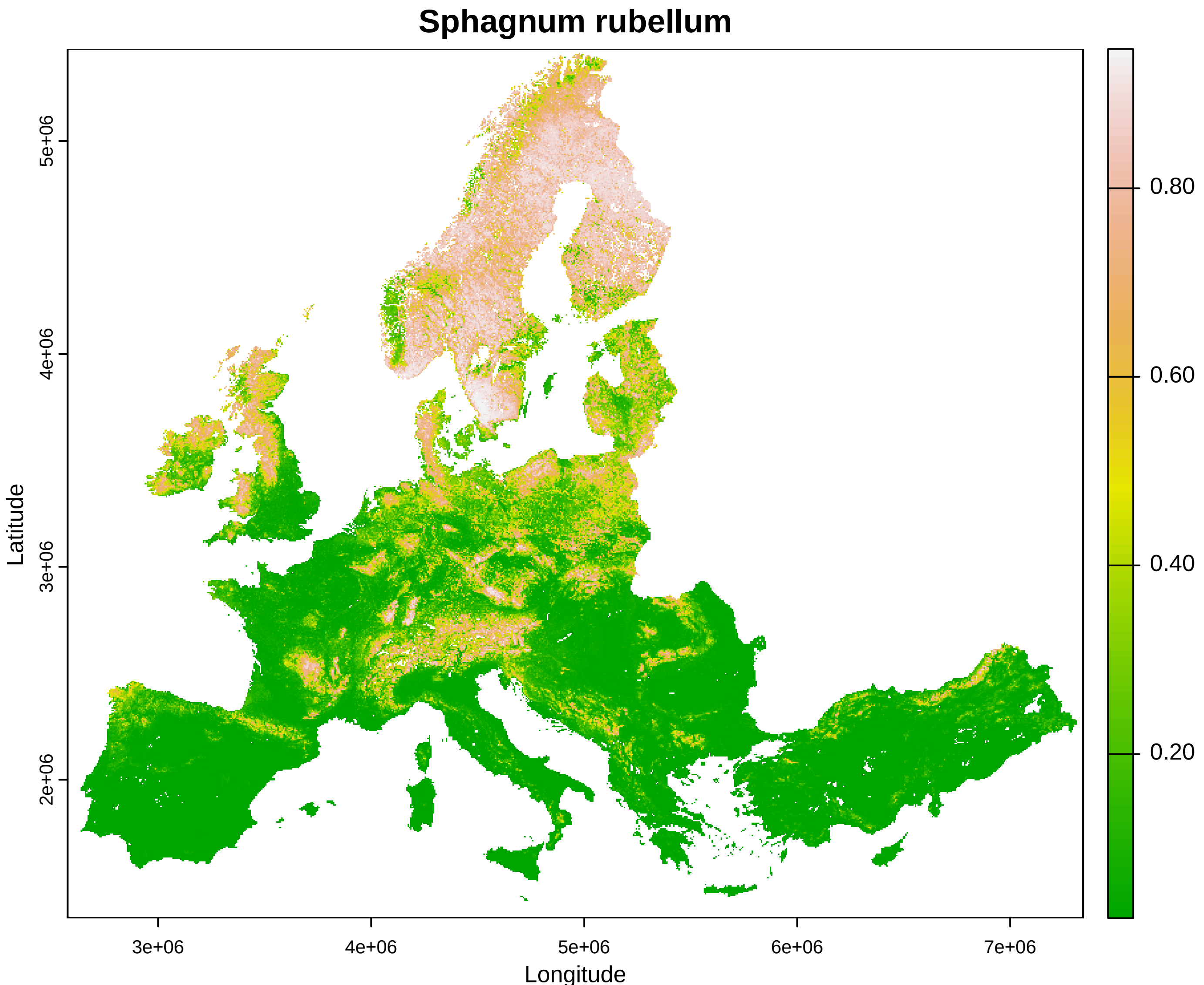

Sphagnum rubellum
Latitude
Longitude
5e+06
4e+06
3e+06
2e+06
3e+06
4e+06
5e+06
6e+06
7e+06
0.80
0.60
0.40
0.20

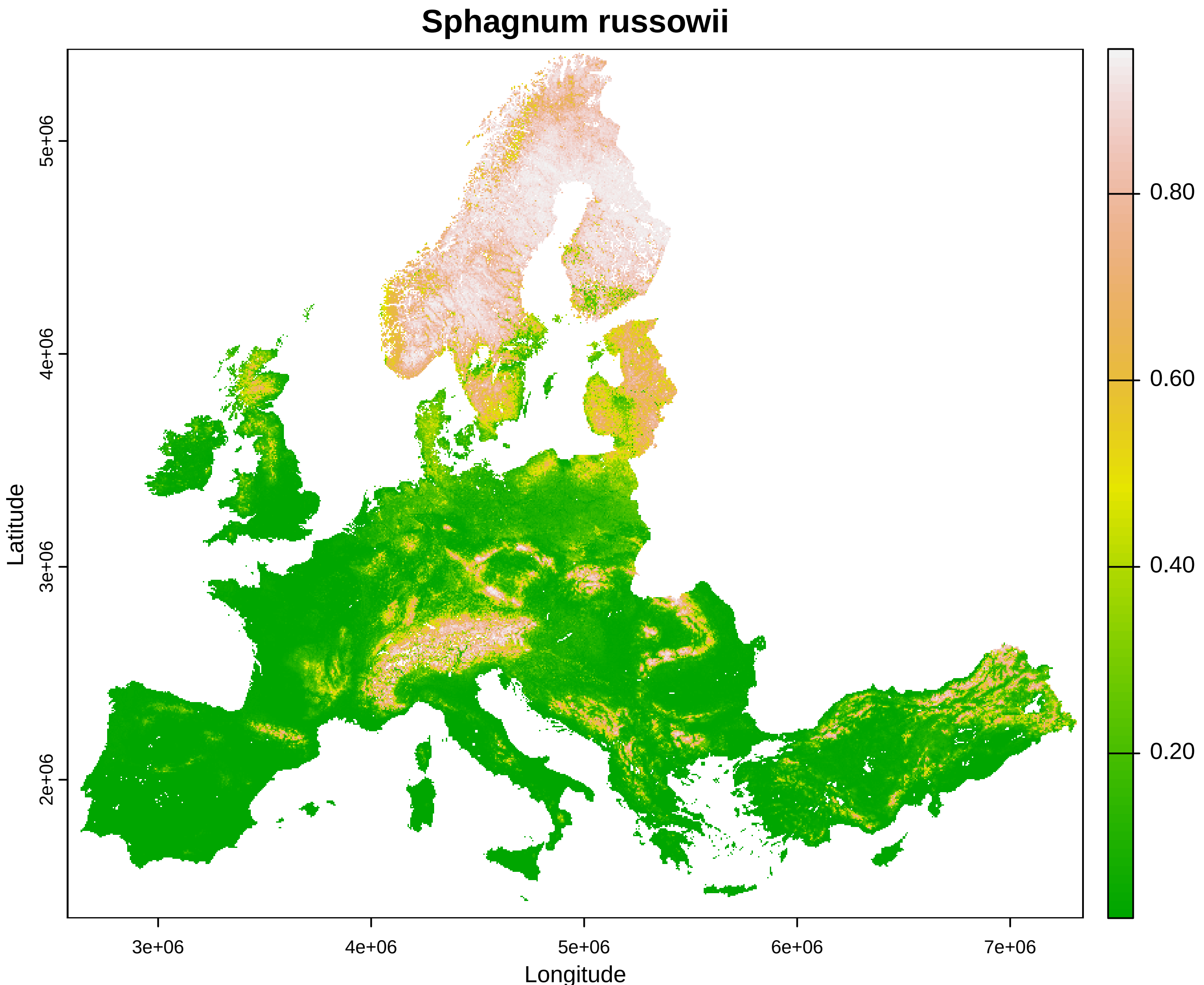
Sphagnum russowii
Latitude
5e+06
4e+06
3e+06
2e+06
3e+06
4e+06
5e+06
6e+06
7e+06
Longitude
0.80
0.60
0.40
0.20

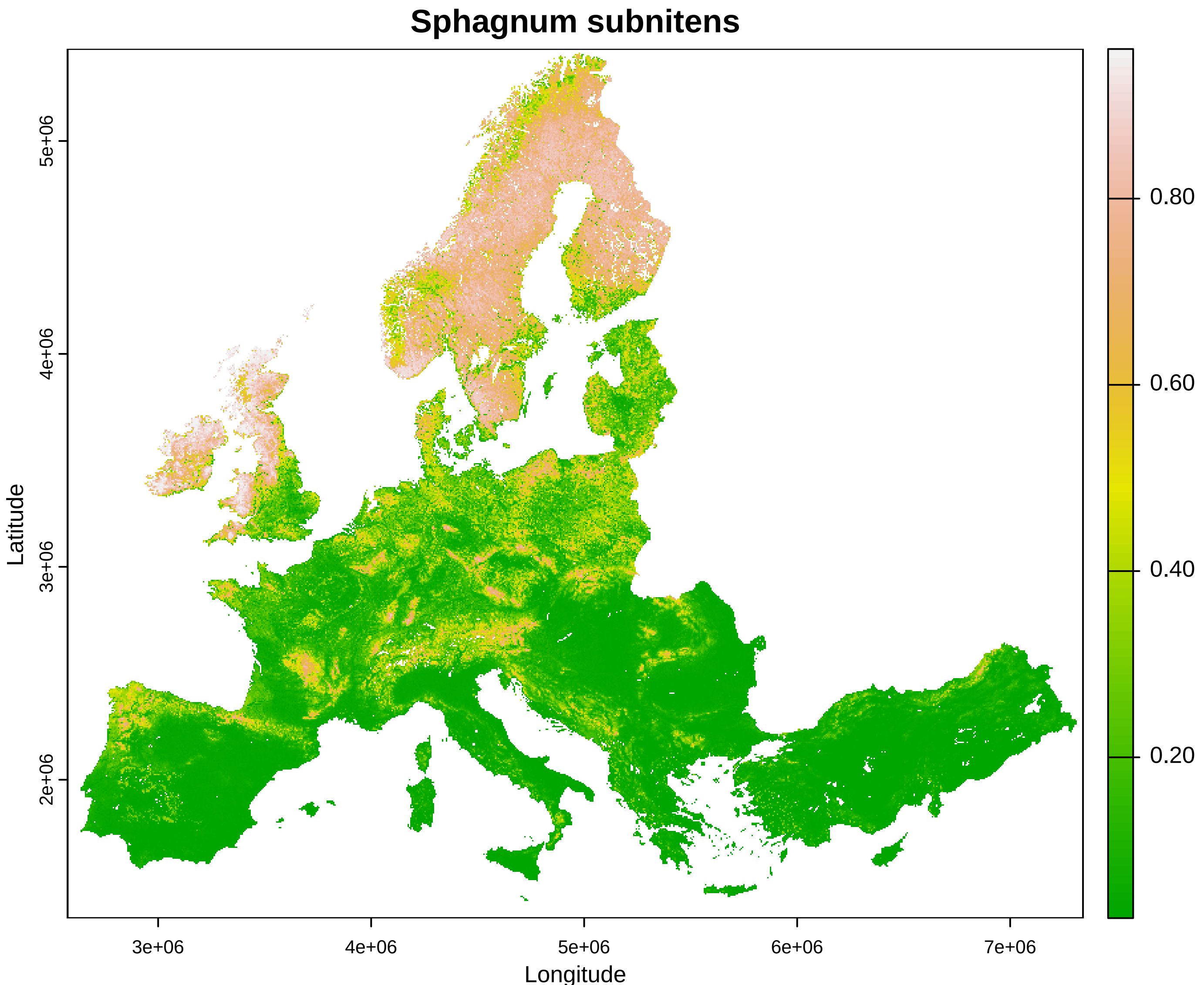
Sphagnum subnitens
Latitude
Longitude
5e+06
4e+06
3e+06
2e+06
3e+06
4e+06
5e+06
6e+06
7e+06
0.80
0.60
0.40
0.20

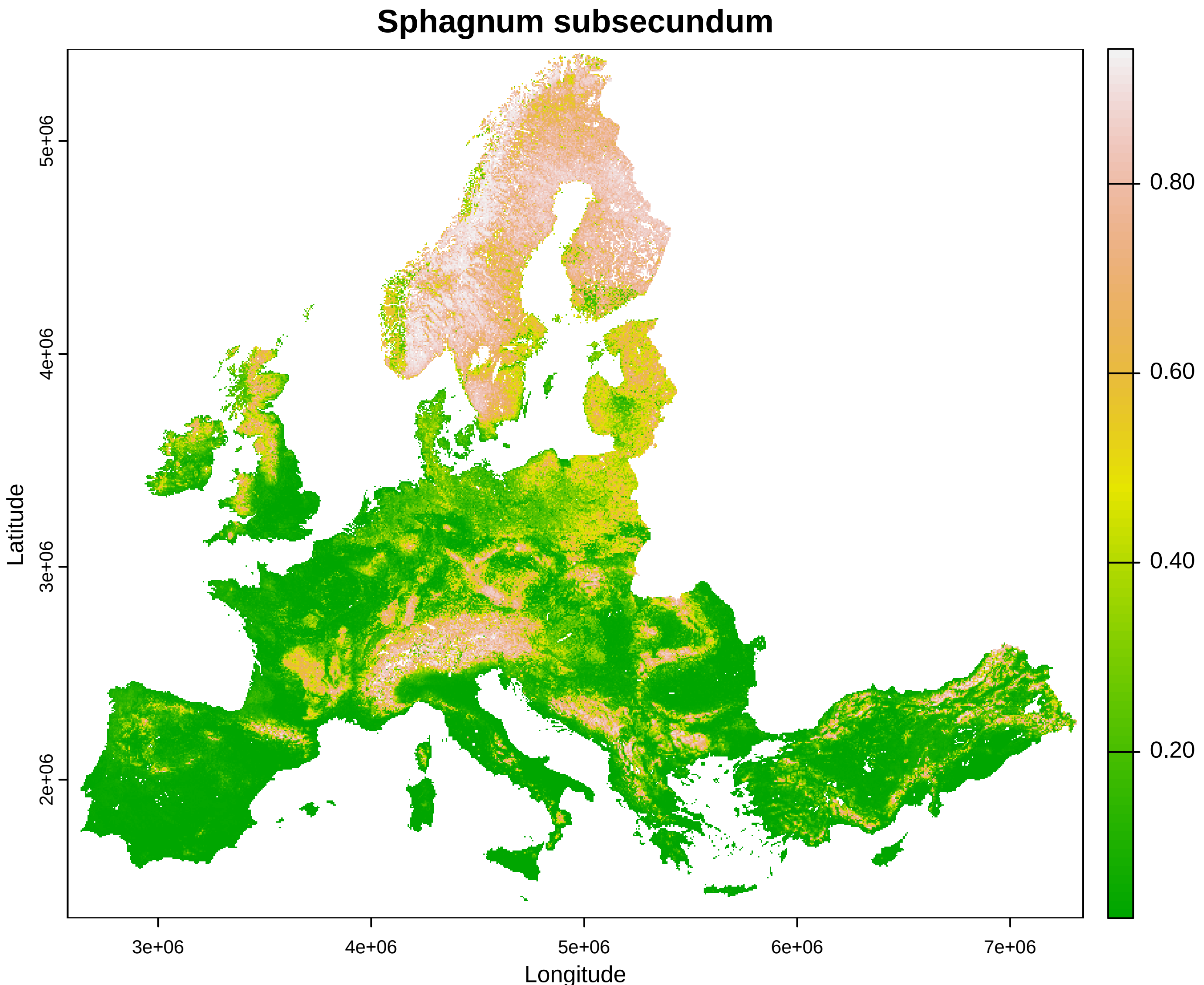
Sphagnum subsecundum
Latitude
Longitude
5e+06
4e+06
3e+06
2e+06
3e+06
4e+06
5e+06
6e+06
7e+06
0.80
0.60
0.40
0.20

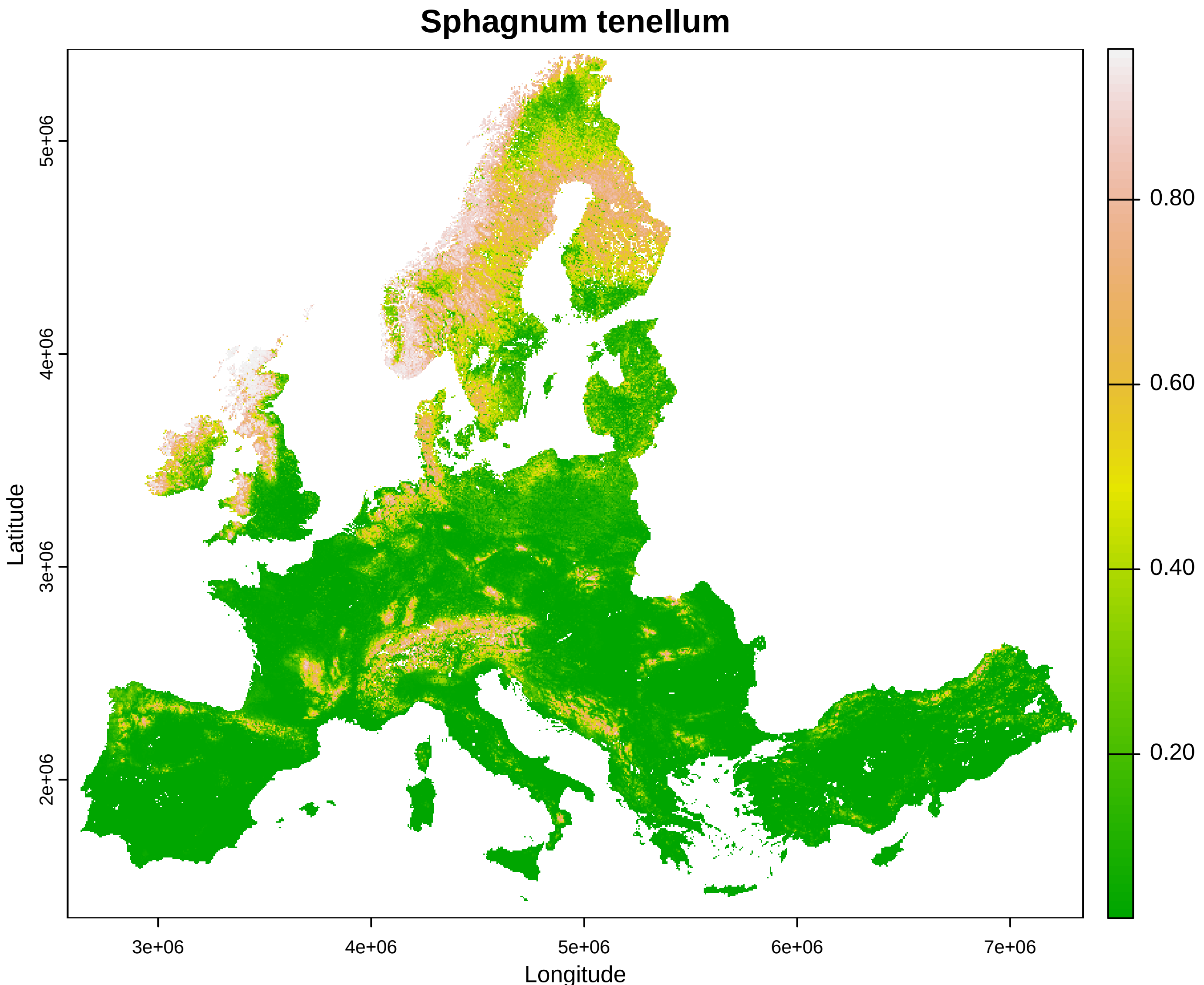

Sphagnum tenellum
Latitude
Longitude
5e+06
4e+06
3e+06
2e+06
3e+06
4e+06
5e+06
6e+06
7e+06
0.80
0.60
0.40
0.20

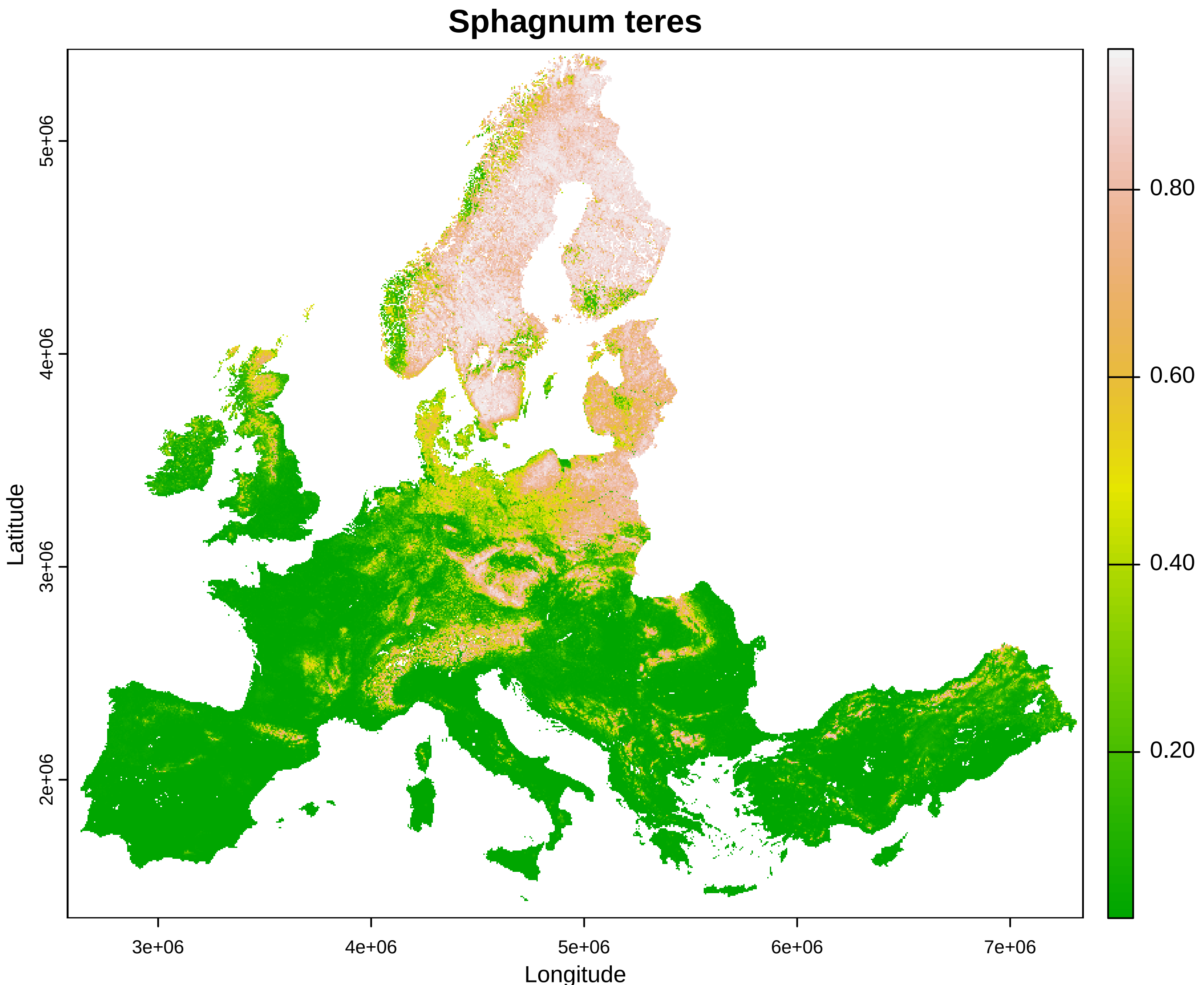
Sphagnum teres
Latitude
5e+06
4e+06
3e+06
2e+06
Longitude
3e+06
4e+06
5e+06
6e+06
7e+06
0.80
0.60
0.40
0.20

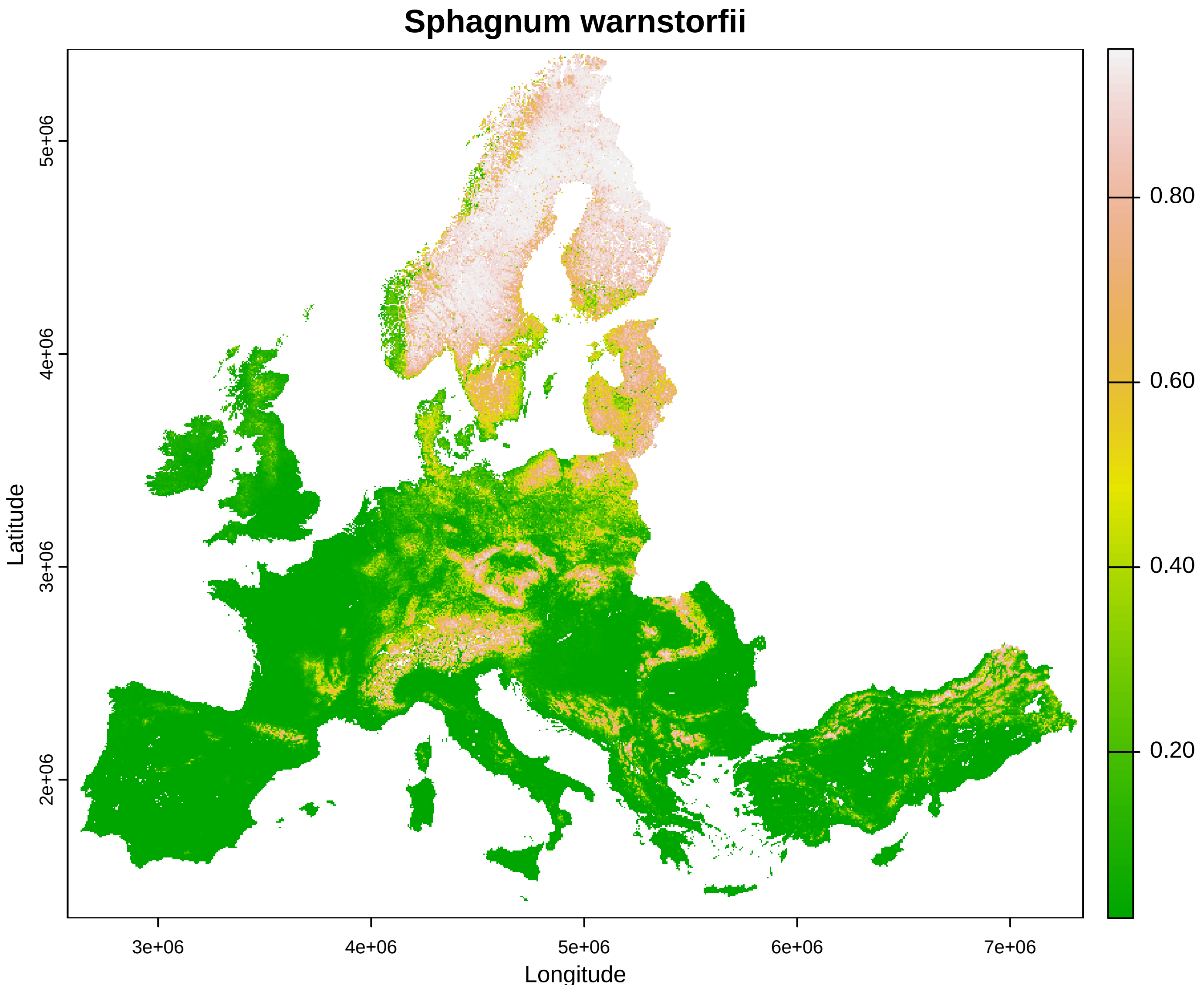

Sphagnum warnstorfii
Latitude
Longitude
5e+06
4e+06
3e+06
2e+06
3e+06
4e+06
5e+06
6e+06
7e+06
0.80
0.60
0.40
0.20

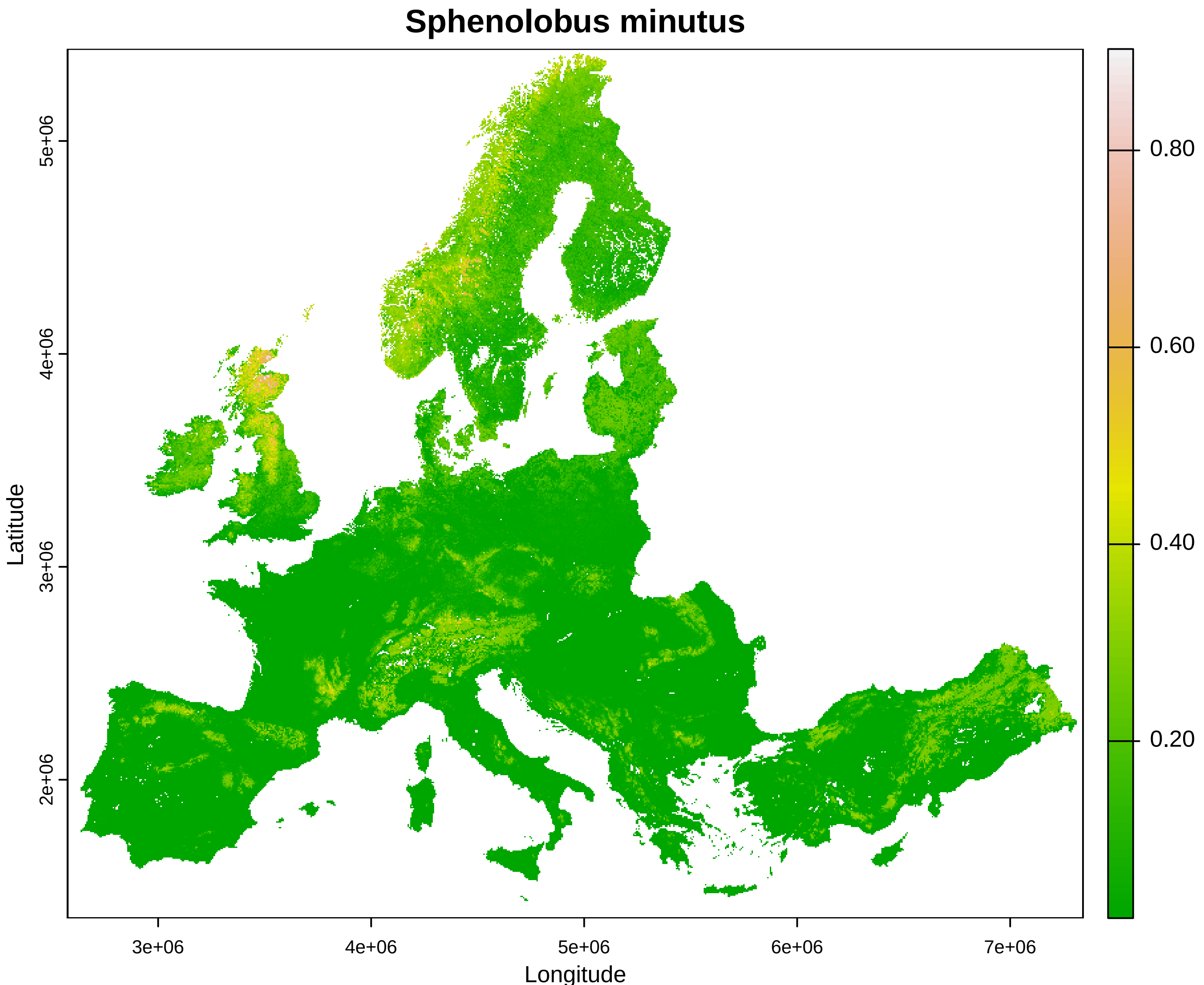

Sphenolobus minutus
Latitude
Longitude
5e+06
4e+06
3e+06
2e+06
3e+06
4e+06
5e+06
6e+06
7e+06
0.80
0.60
0.40
0.20

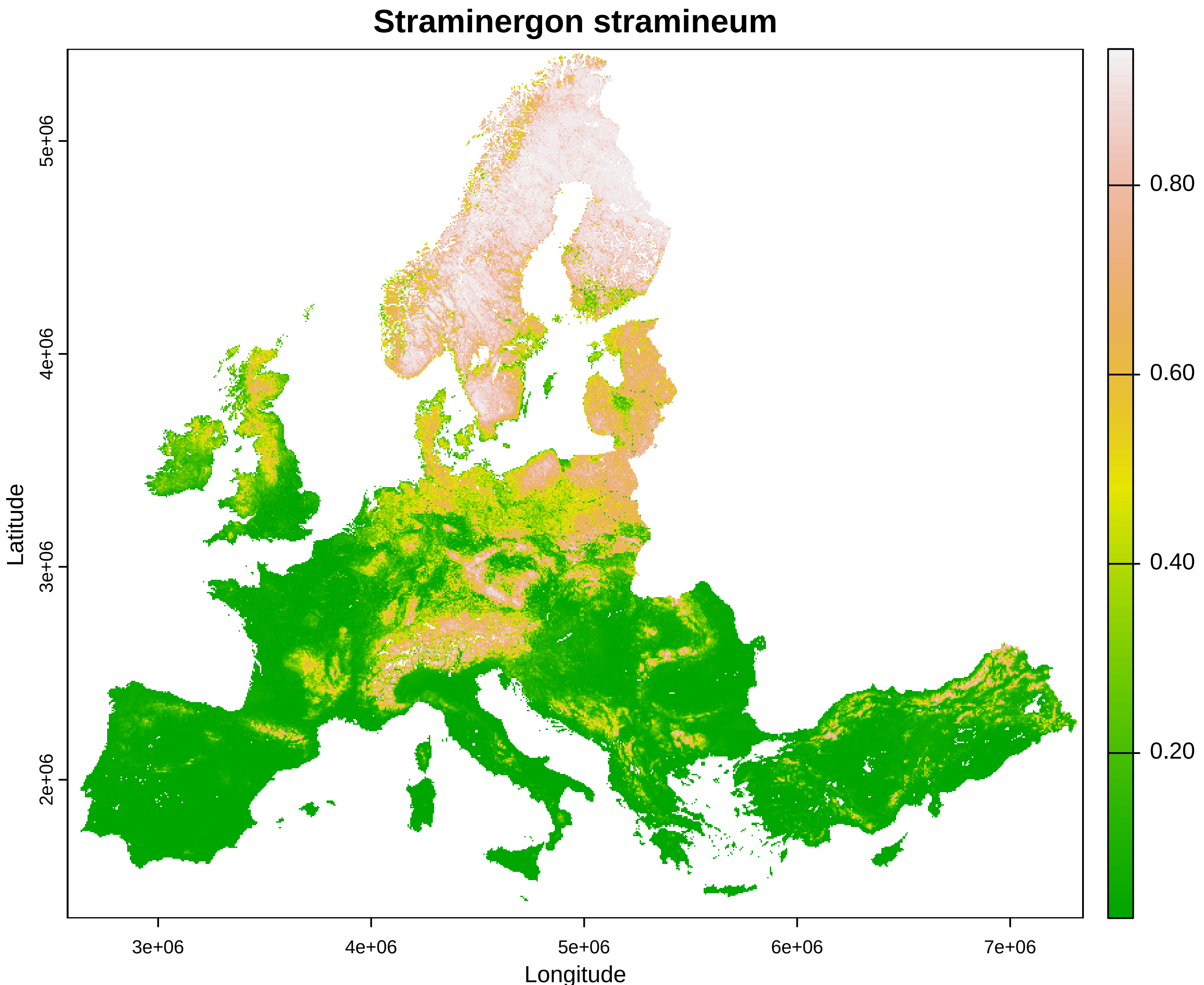

Straminergon stramineum
Latitude
Longitude
5e+06
4e+06
3e+06
2e+06
3e+06
4e+06
5e+06
6e+06
7e+06
0.80
0.60
0.40
0.20

Succisa pratensis

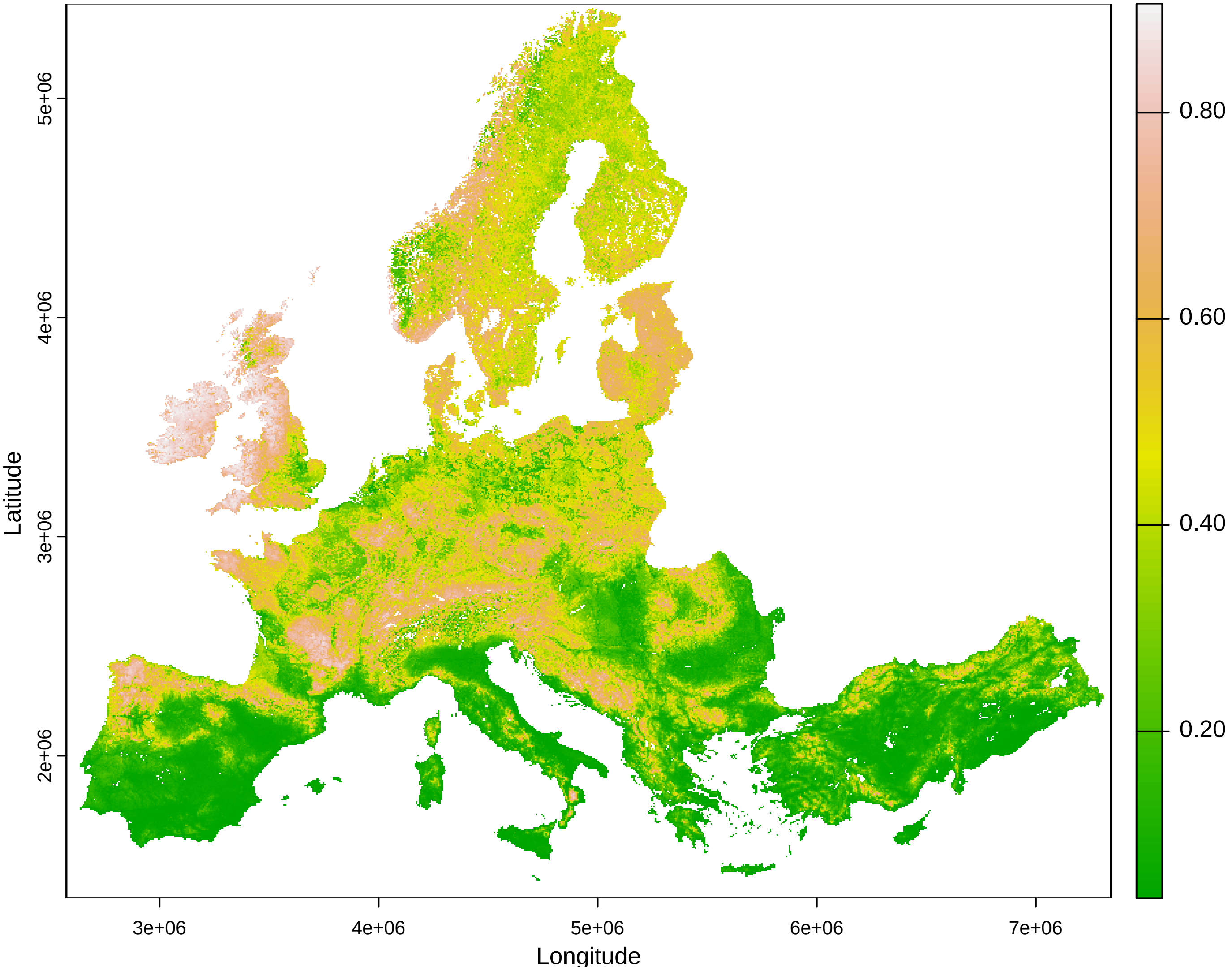

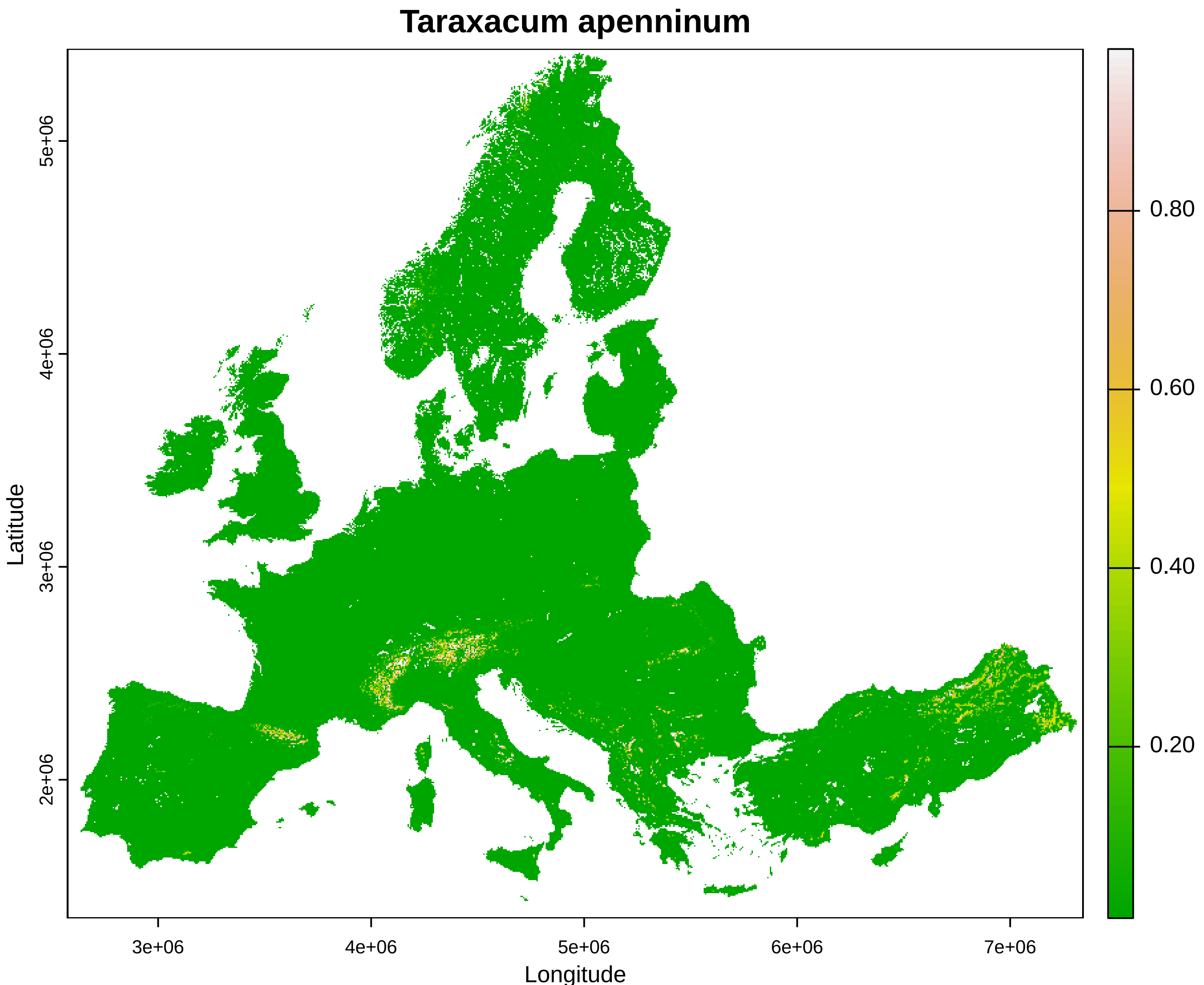
Taraxacum apenninum
Latitude
5e+06
4e+06
3e+06
2e+06
3e+06
4e+06
5e+06
6e+06
7e+06
Longitude
0.80
0.60
0.40
0.20

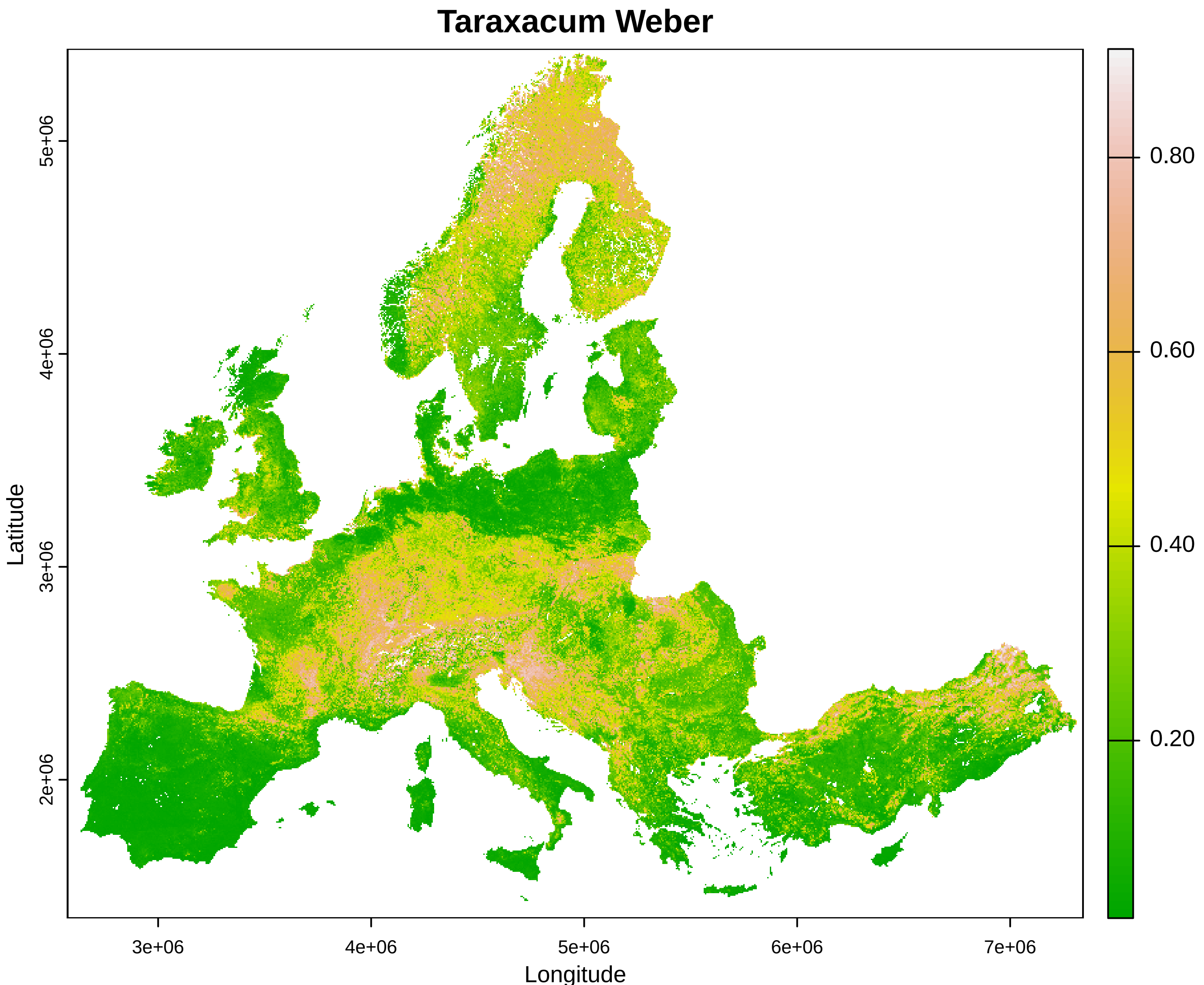
Taraxacum Weber
Latitude
Longitude
5e+06
4e+06
3e+06
2e+06
3e+06
4e+06
5e+06
6e+06
7e+06
0.80
0.60
0.40
0.20

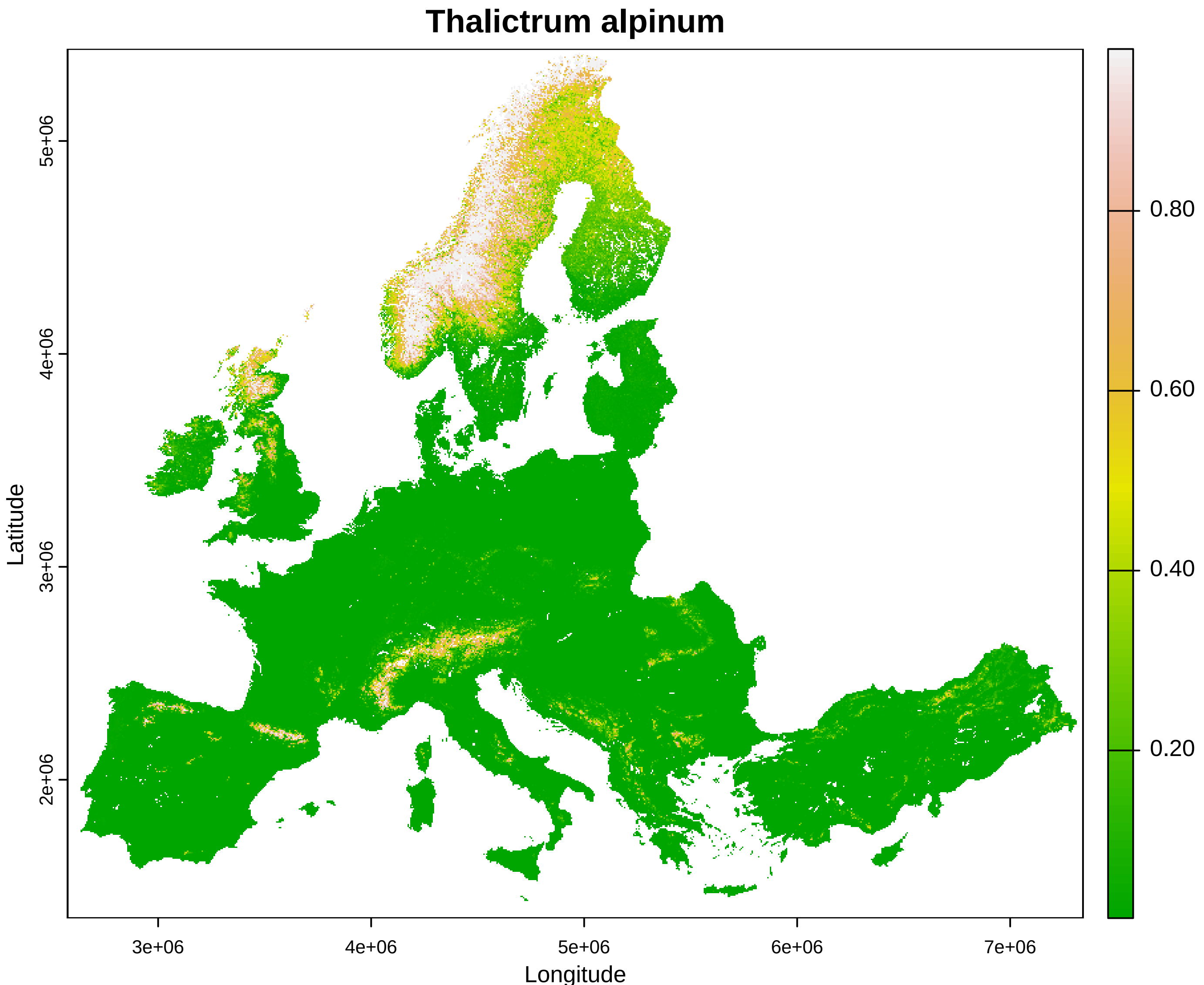
Thalictrum alpinum
Latitude
5e+06
4e+06
3e+06
2e+06
Longitude
3e+06
4e+06
5e+06
6e+06
7e+06
0.80
0.60
0.40
0.20

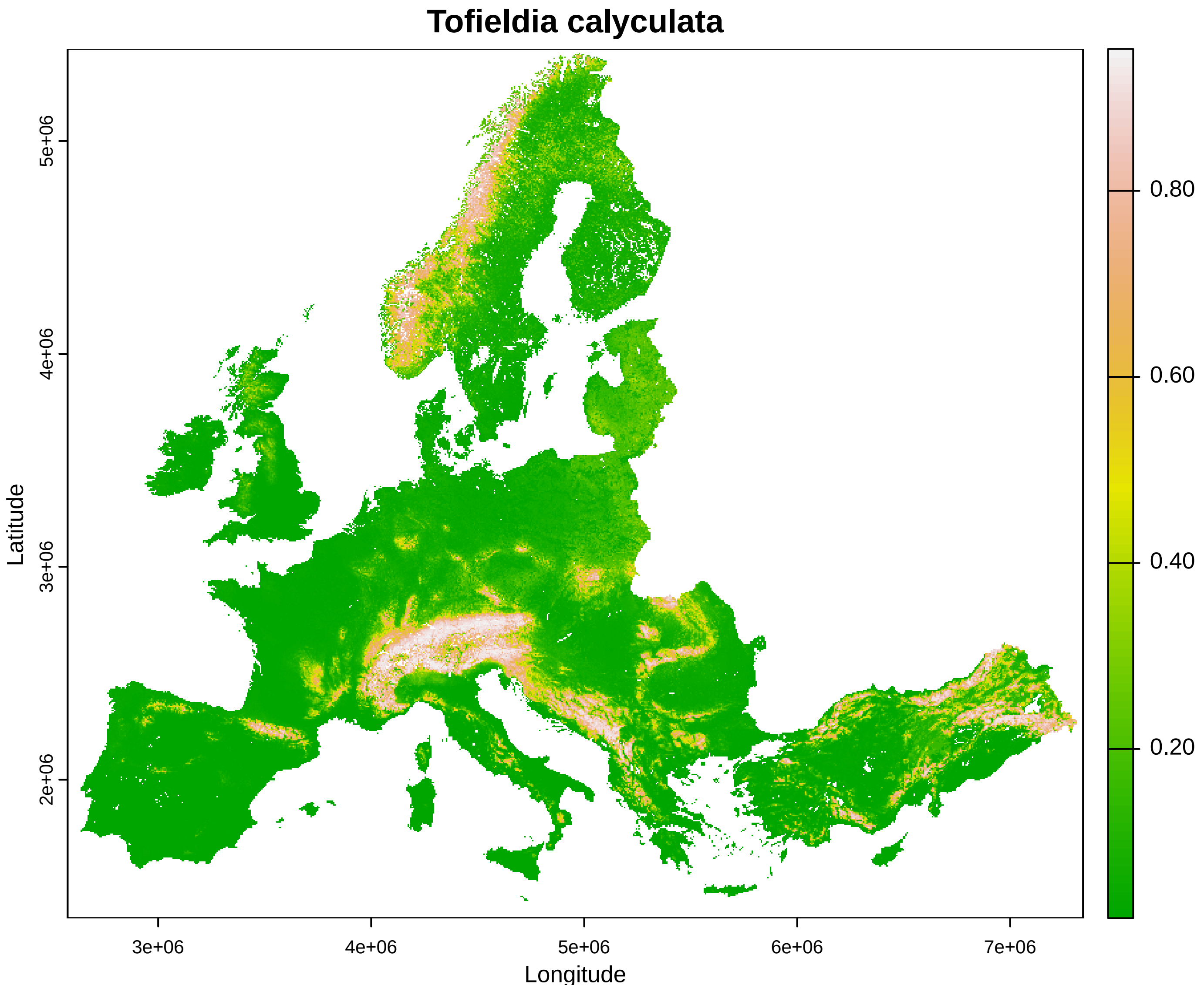

Tofieldia calyculata
Latitude
Longitude
5e+06
4e+06
3e+06
2e+06
3e+06
4e+06
5e+06
6e+06
7e+06
0.80
0.60
0.40
0.20

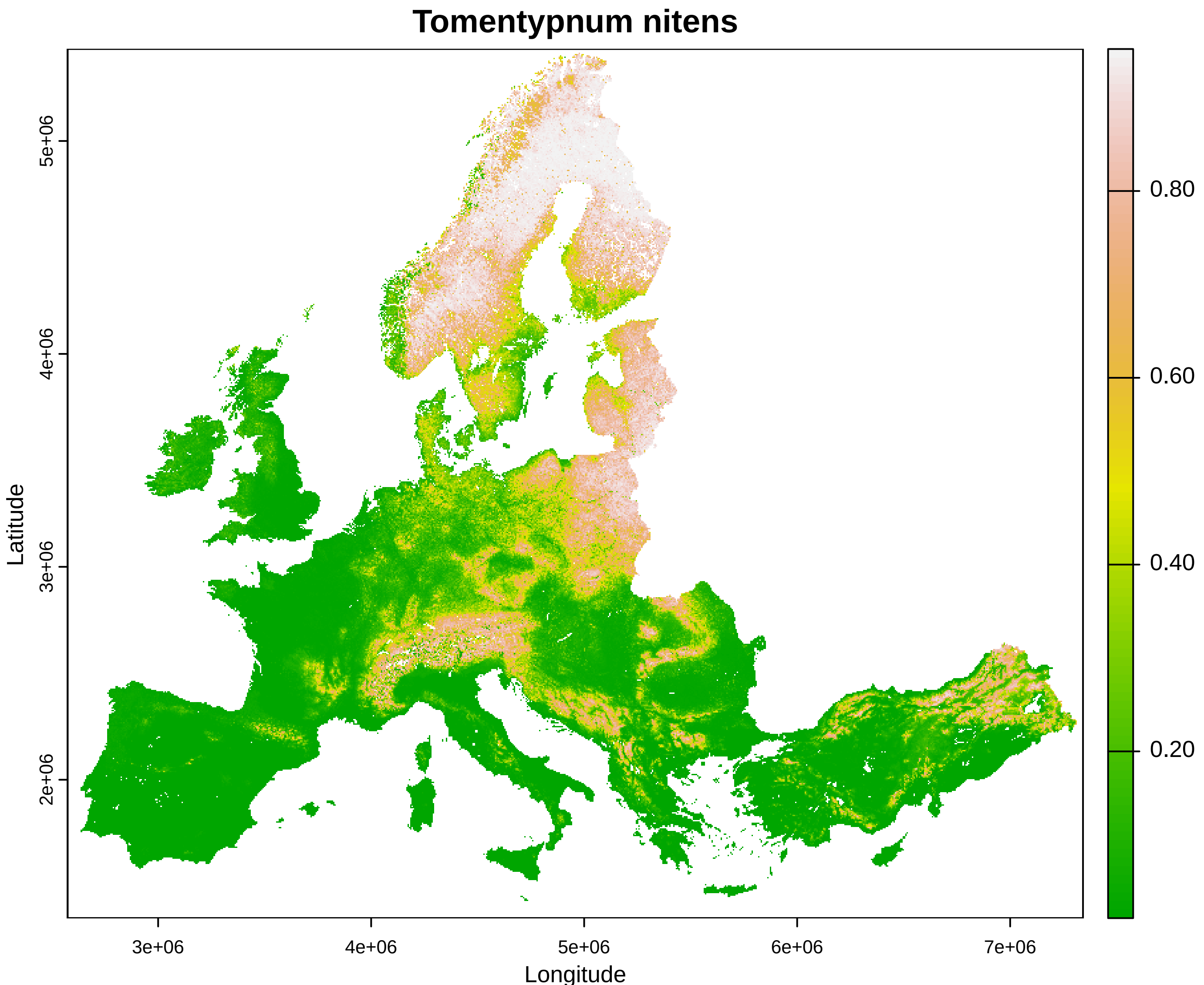
Tomentypnum nitens
Latitude
5e+06
4e+06
3e+06
2e+06
3e+06
4e+06
5e+06
6e+06
7e+06
Longitude
0.80
0.60
0.40
0.20

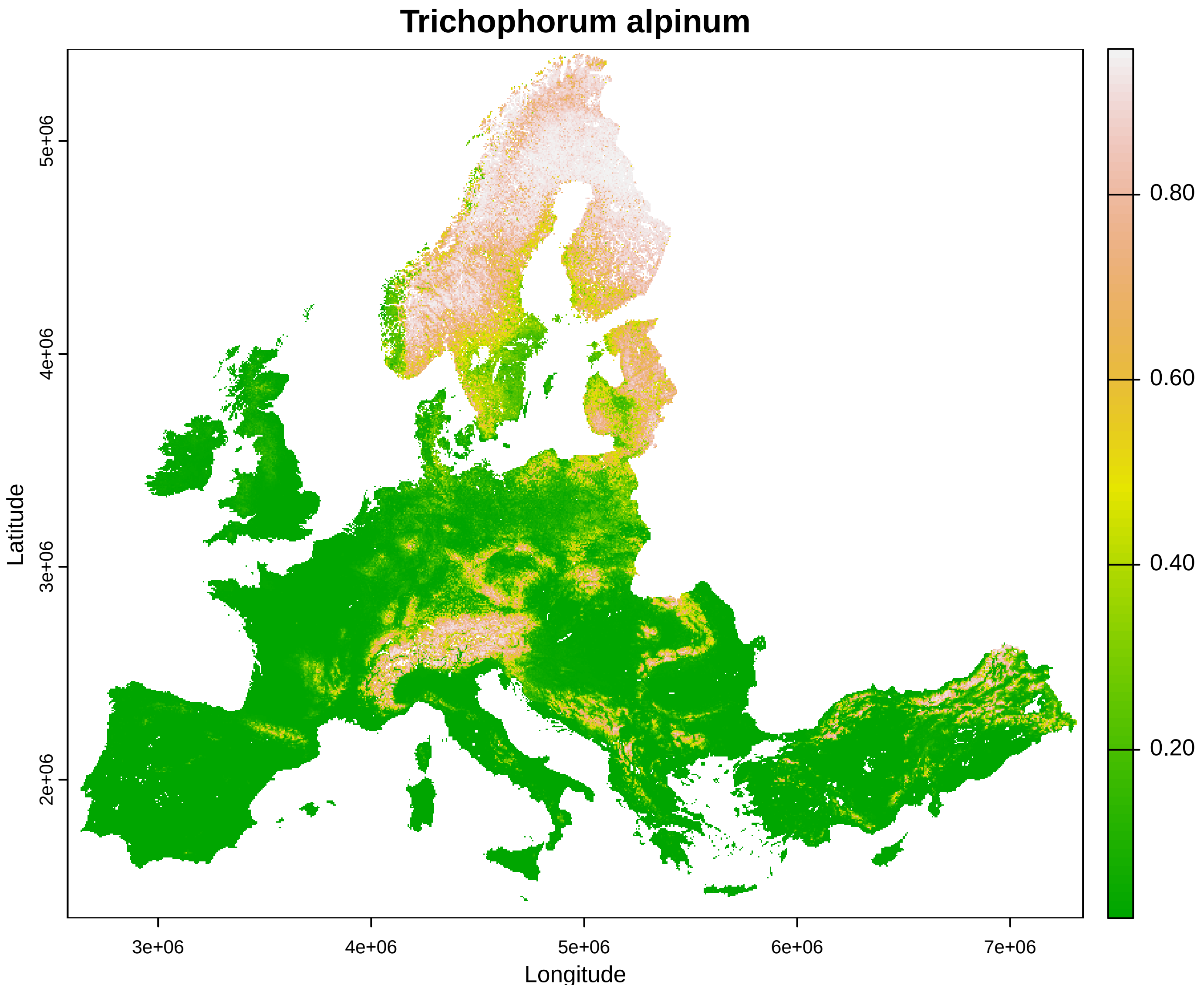

Trichophorum alpinum
Latitude
Longitude
5e+06
4e+06
3e+06
2e+06
3e+06
4e+06
5e+06
6e+06
7e+06
0.80
0.60
0.40
0.20

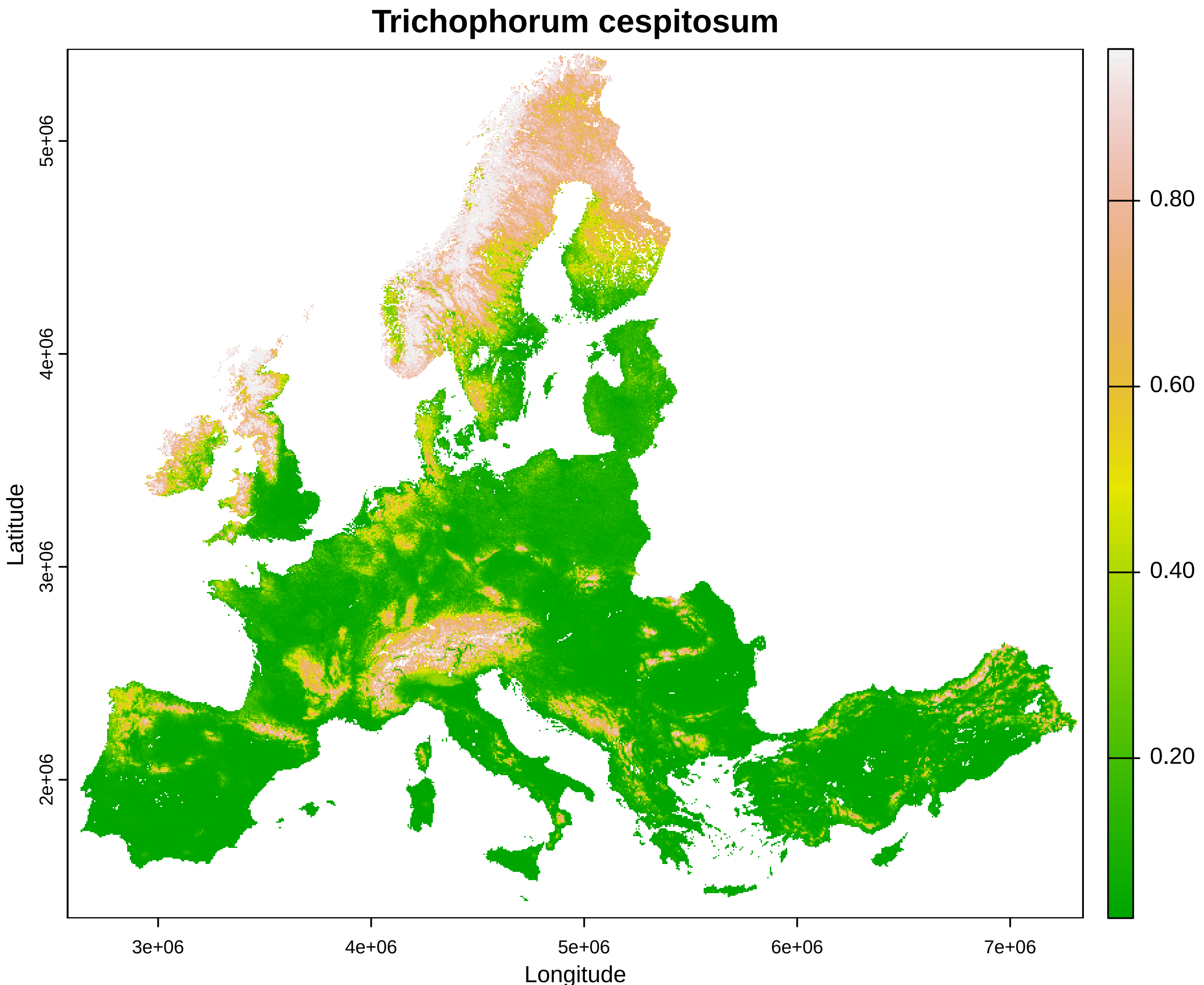
Trichophorum cespitosum
Latitude
Longitude
5e+06
4e+06
3e+06
2e+06
3e+06
4e+06
5e+06
6e+06
7e+06
0.80
0.60
0.40
0.20

Triglochin maritima

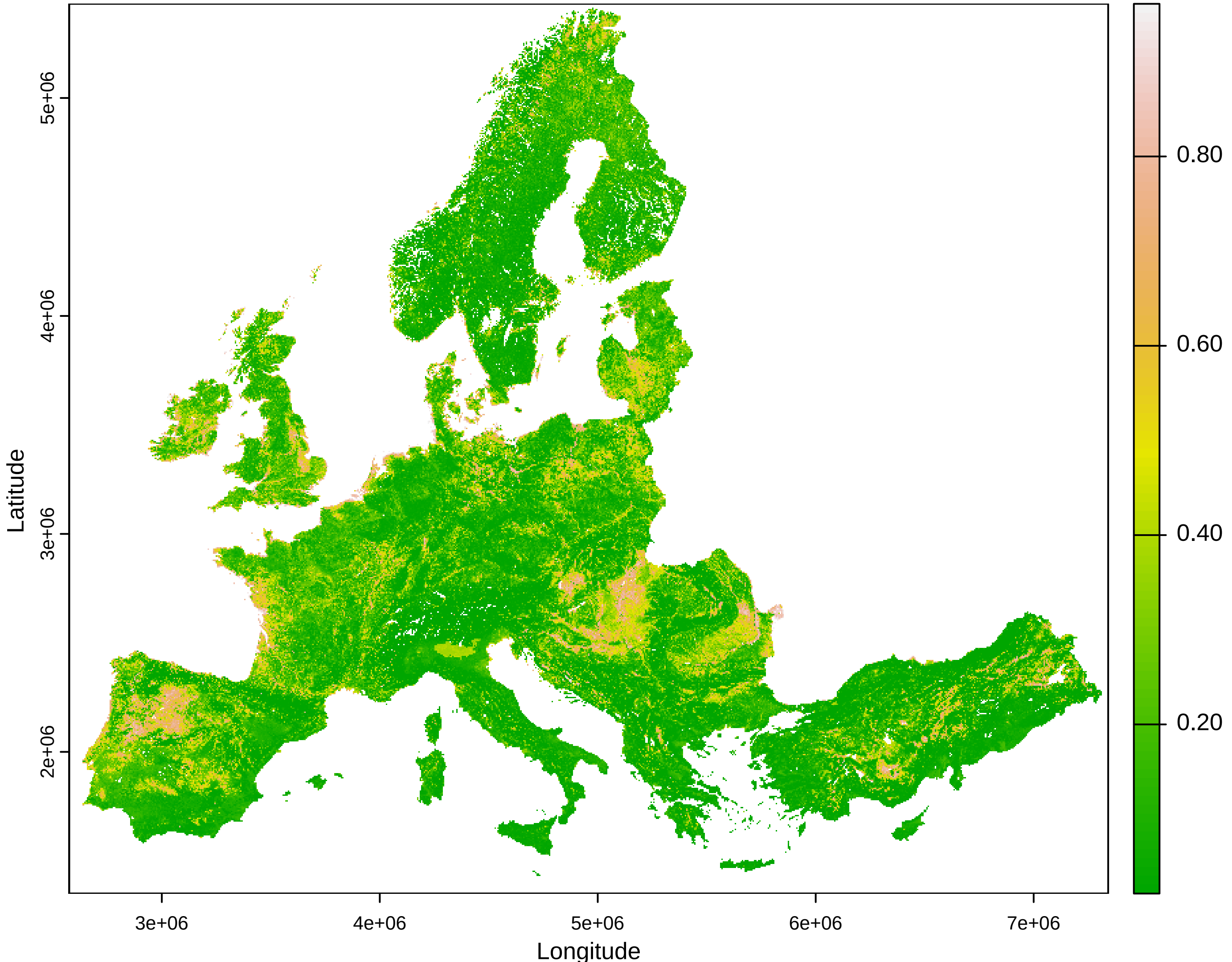

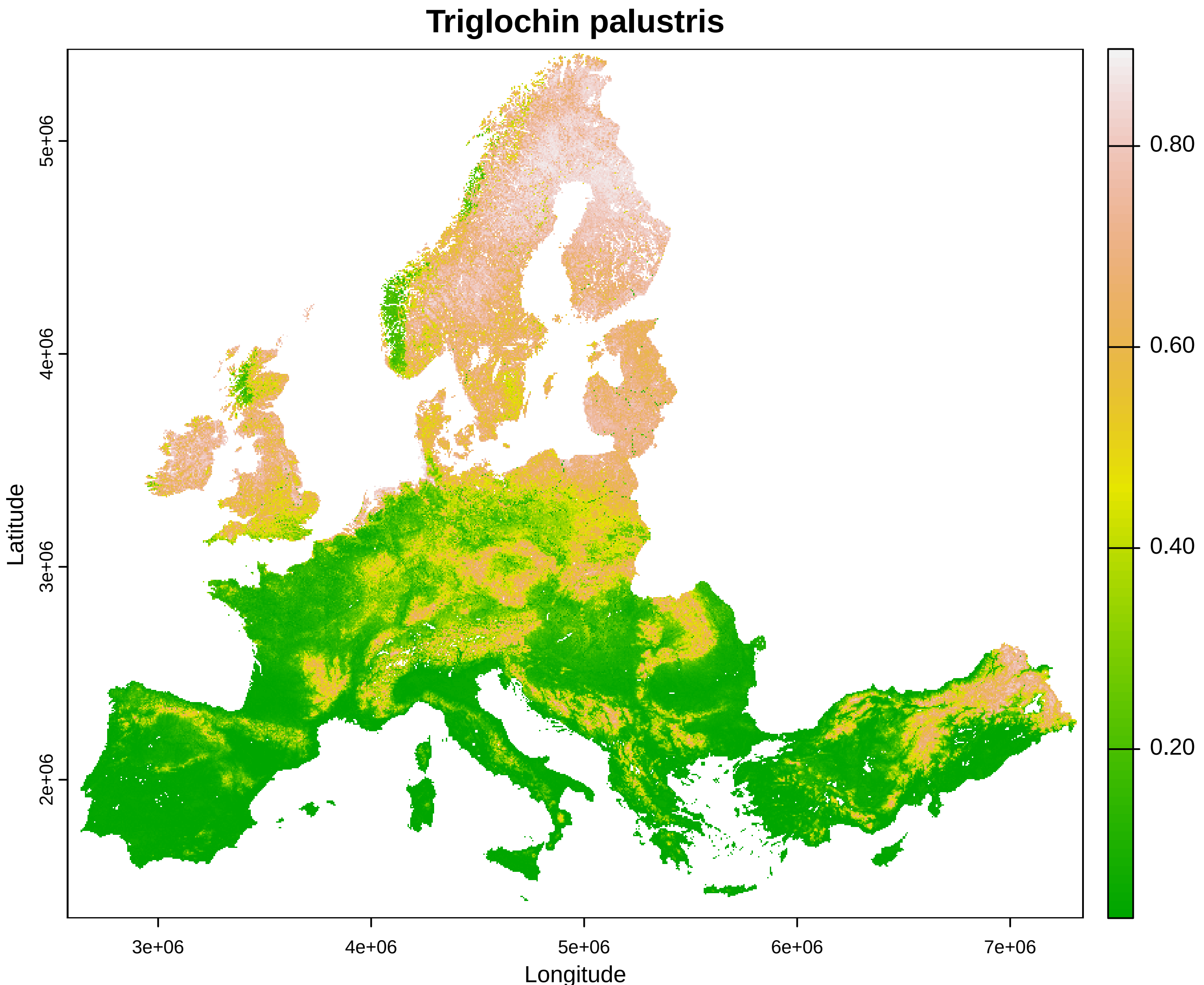

Triglochin palustris
Latitude
5e+06
4e+06
3e+06
2e+06
Longitude
3e+06
4e+06
5e+06
6e+06
7e+06
0.80
0.60
0.40
0.20

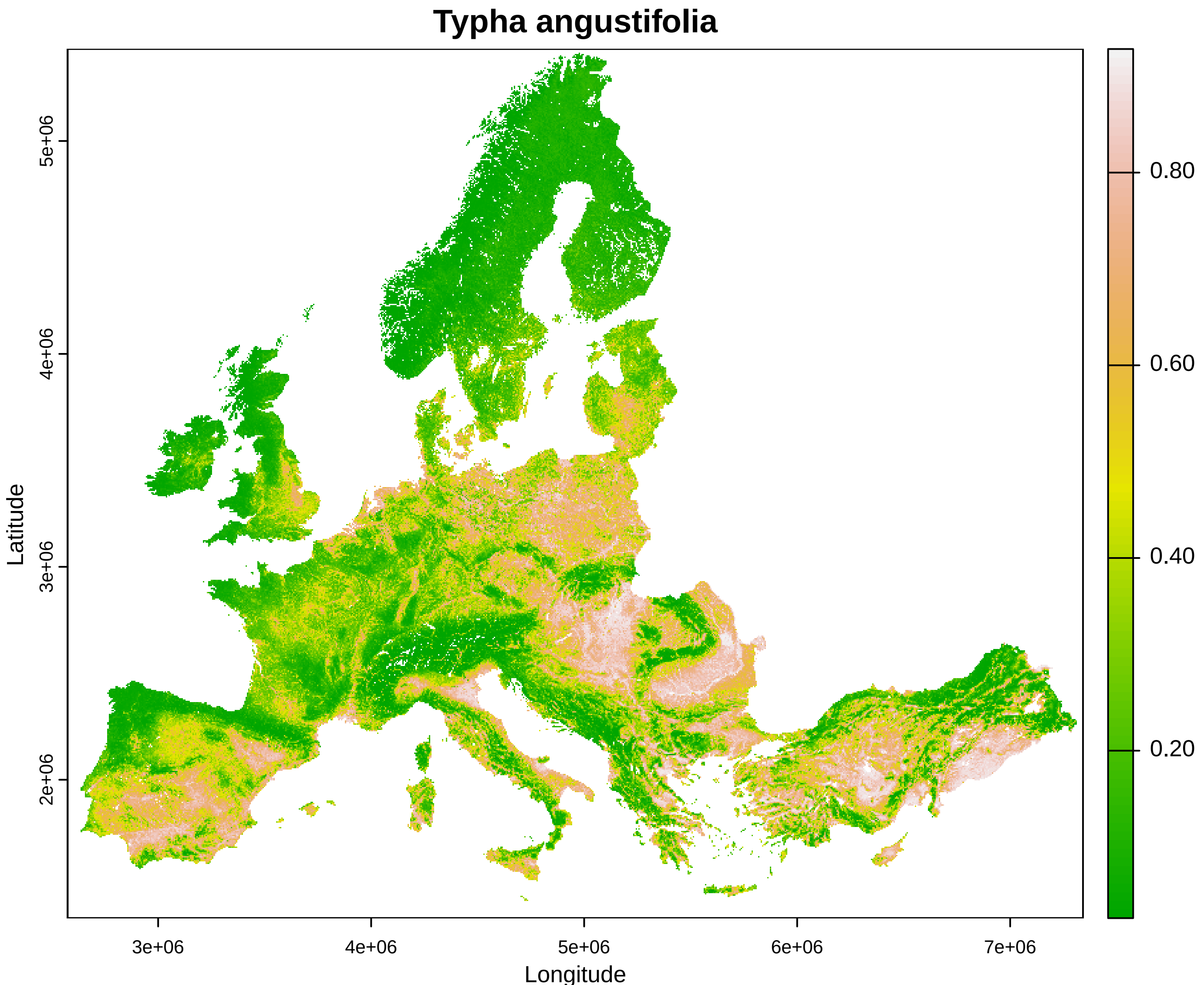
Typha angustifolia
Latitude
5e+06
4e+06
3e+06
2e+06
Longitude
3e+06
4e+06
5e+06
6e+06
7e+06
0.80
0.60
0.40
0.20

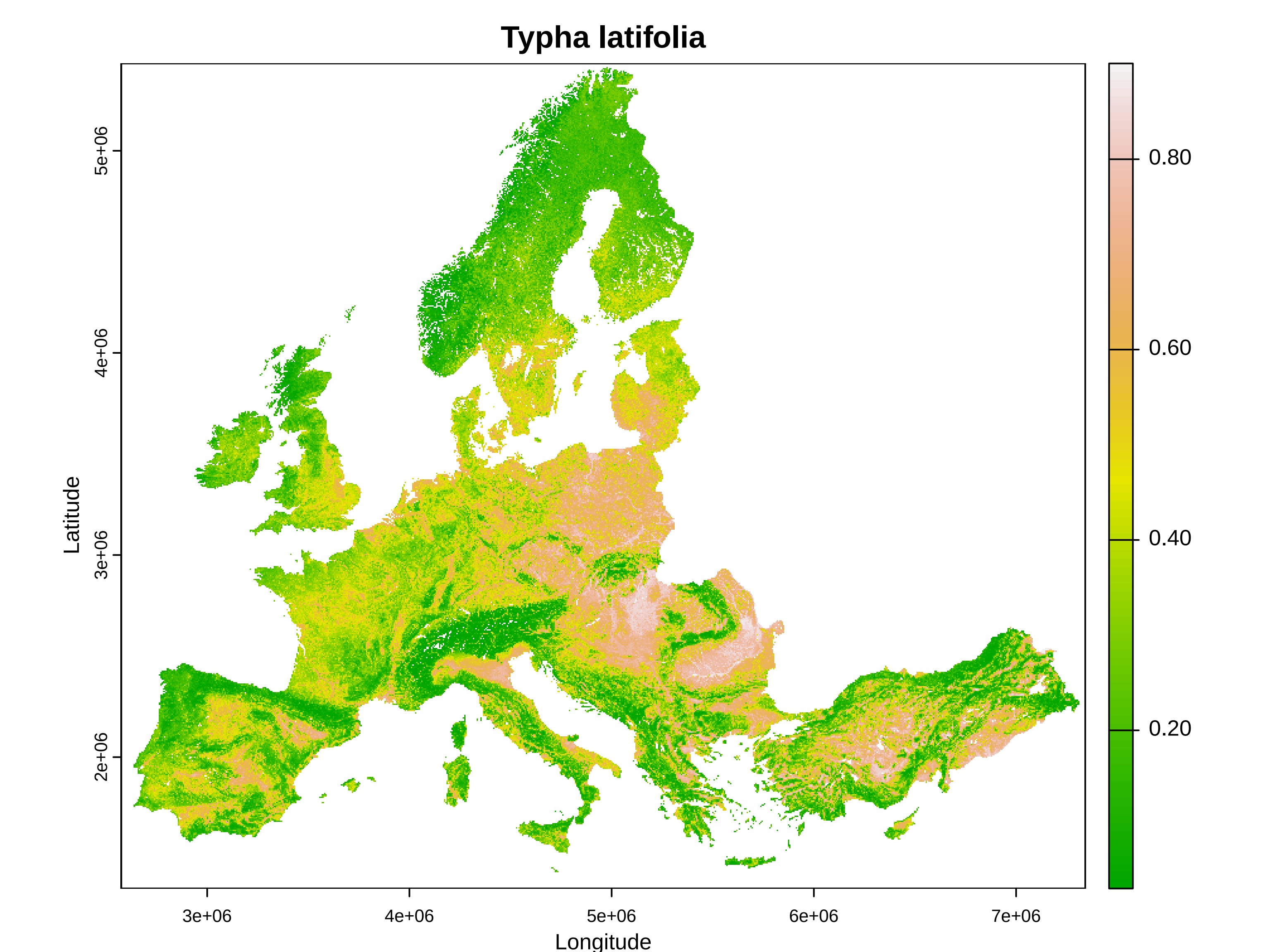
Typha latifolia
Latitude
Longitude
5e+06
4e+06
3e+06
2e+06
3e+06
4e+06
5e+06
6e+06
7e+06
0.80
0.60
0.40
0.20

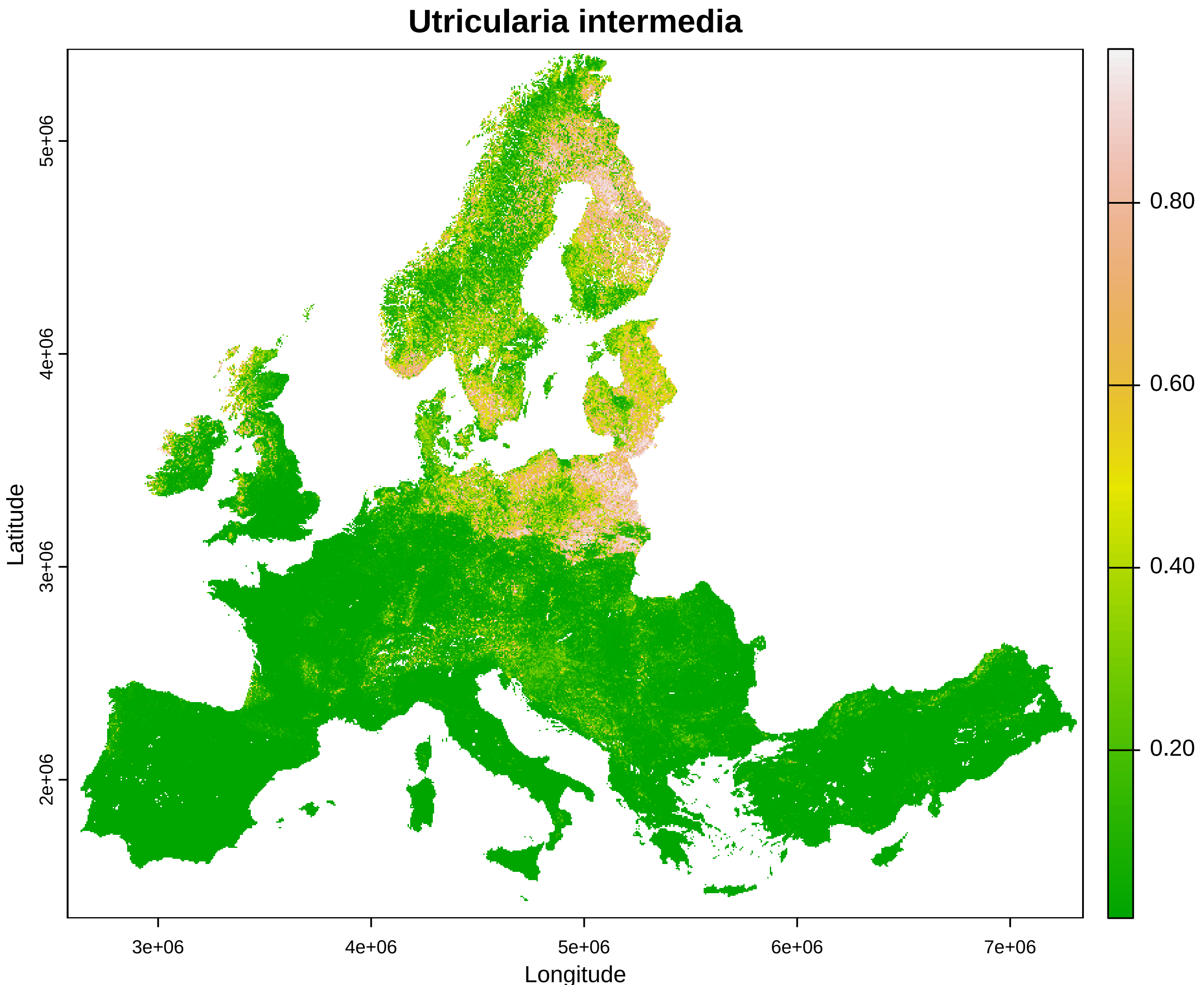
Utricularia intermedia
Latitude
5e+06
4e+06
3e+06
2e+06
Longitude
3e+06
4e+06
5e+06
6e+06
7e+06
0.80
0.60
0.40
0.20

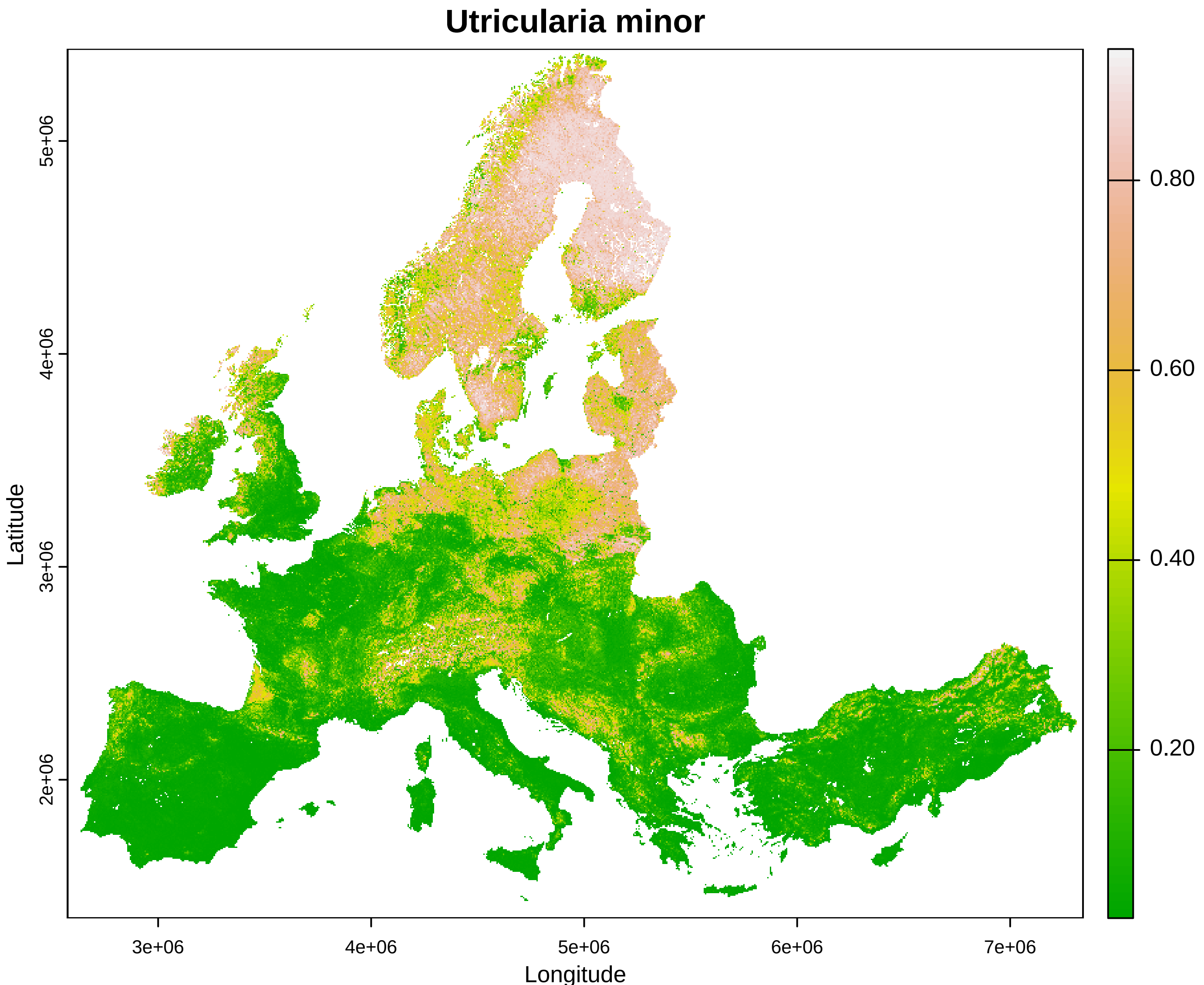
Utricularia minor
Latitude
Longitude
5e+06
4e+06
3e+06
2e+06
3e+06
4e+06
5e+06
6e+06
7e+06
0.80
0.60
0.40
0.20

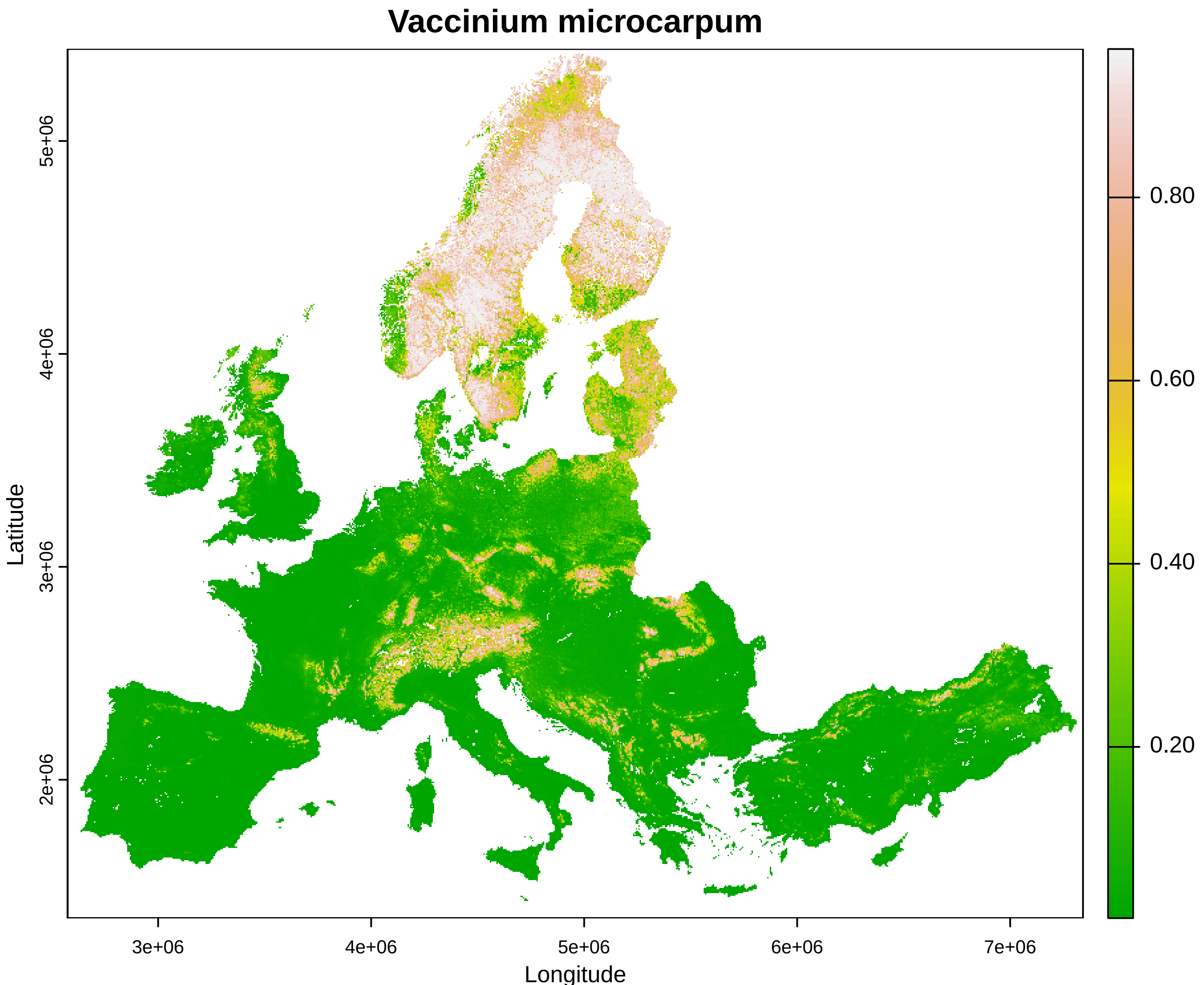
Vaccinium microcarpum
Latitude
Longitude
5e+06
4e+06
3e+06
2e+06
3e+06
4e+06
5e+06
6e+06
7e+06
0.80
0.60
0.40
0.20

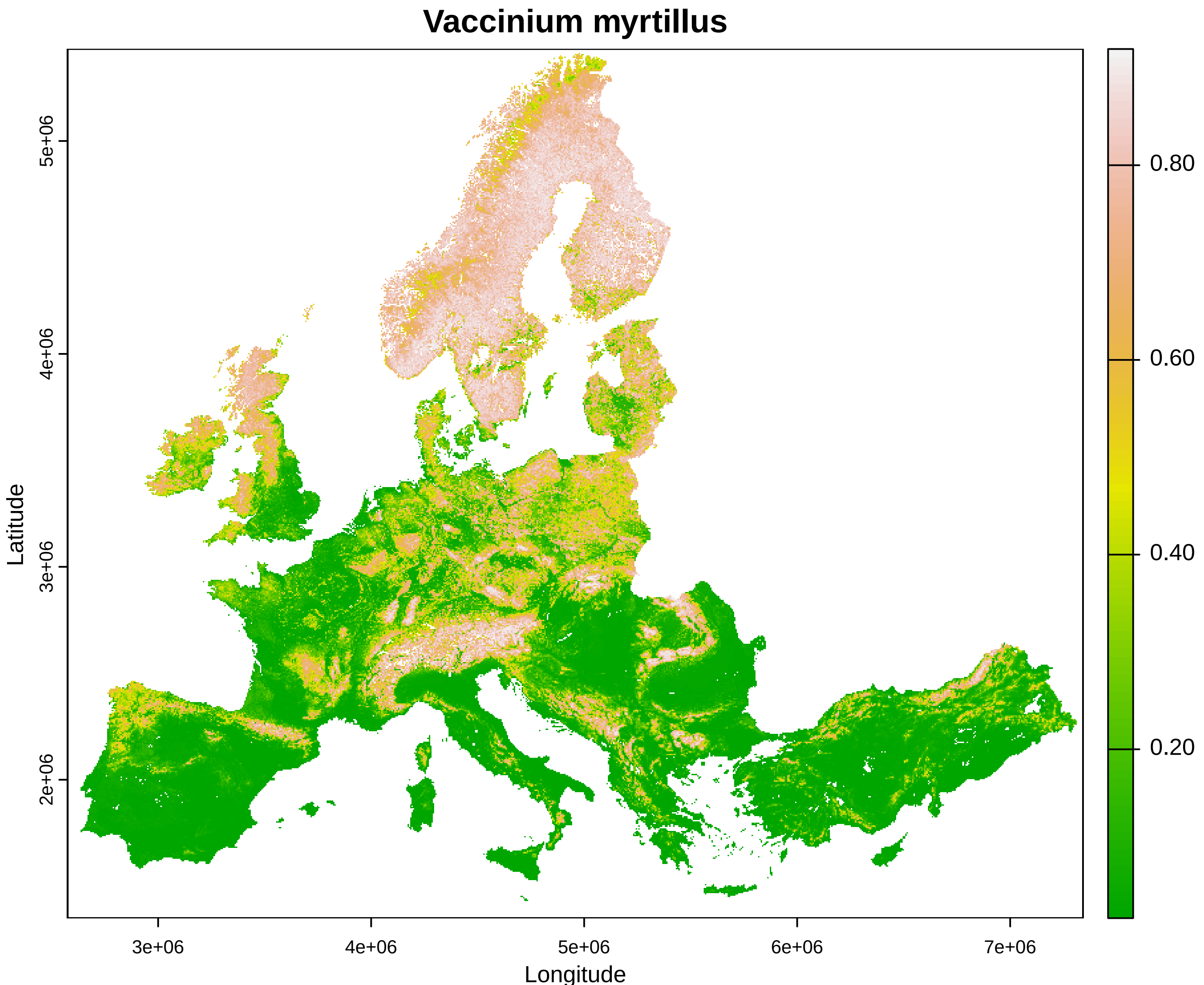
Vaccinium myrtillus
Latitude
Longitude
5e+06
4e+06
3e+06
2e+06
3e+06
4e+06
5e+06
6e+06
7e+06
0.80
0.60
0.40
0.20

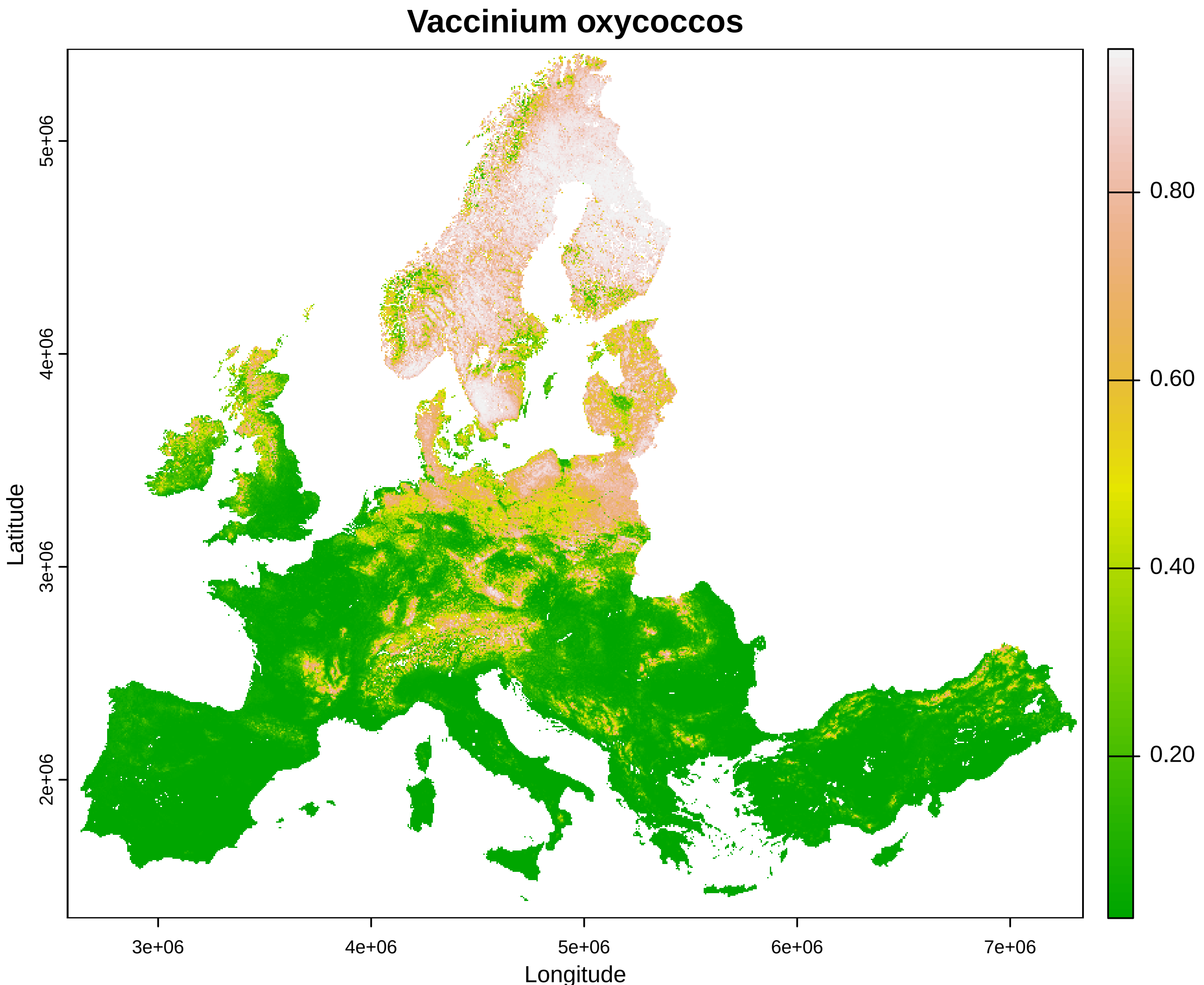

Vaccinium oxycoccos
Latitude
Longitude
5e+06
4e+06
3e+06
2e+06
3e+06
4e+06
5e+06
6e+06
7e+06
0.80
0.60
0.40
0.20

Vaccinium uliginosum

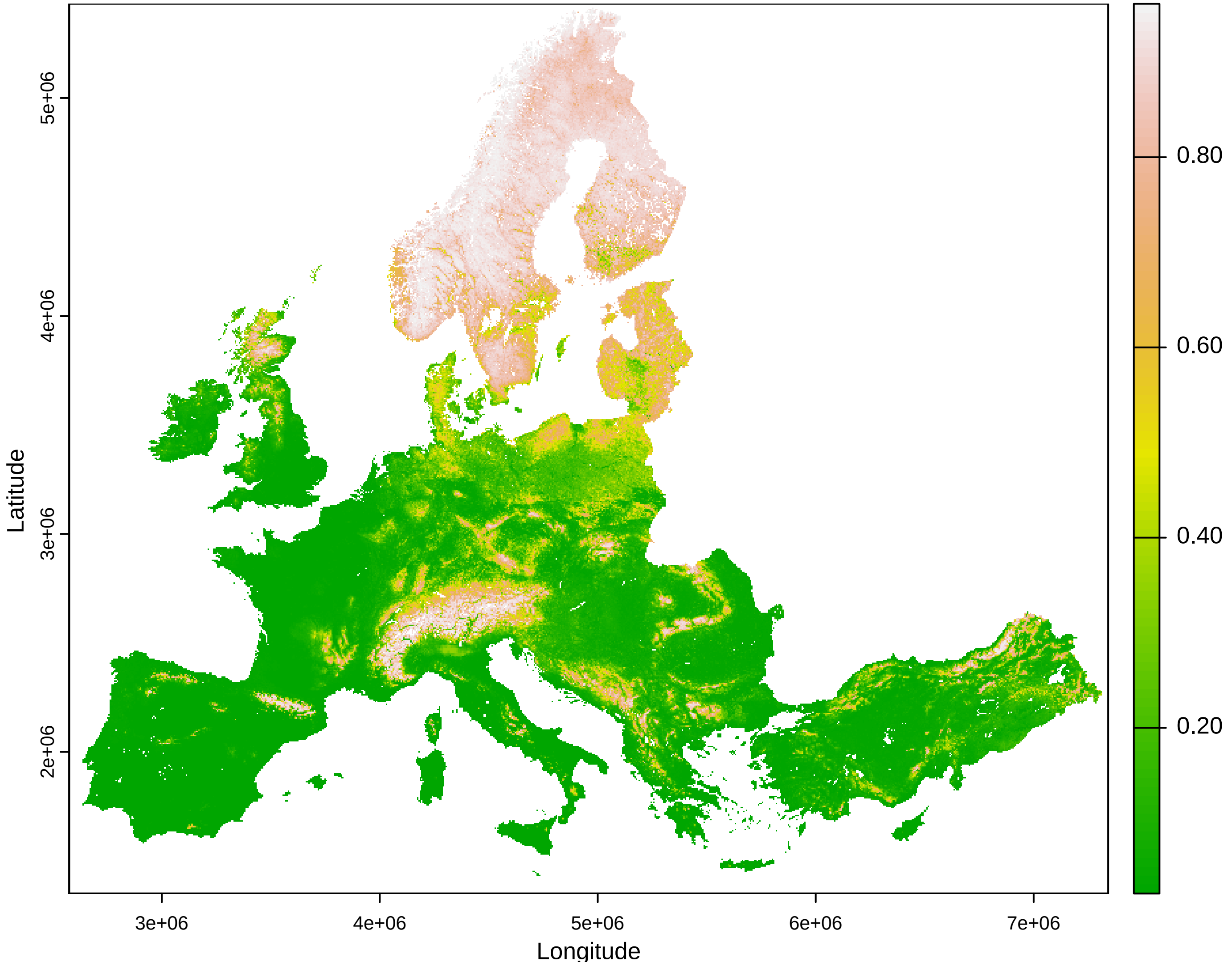

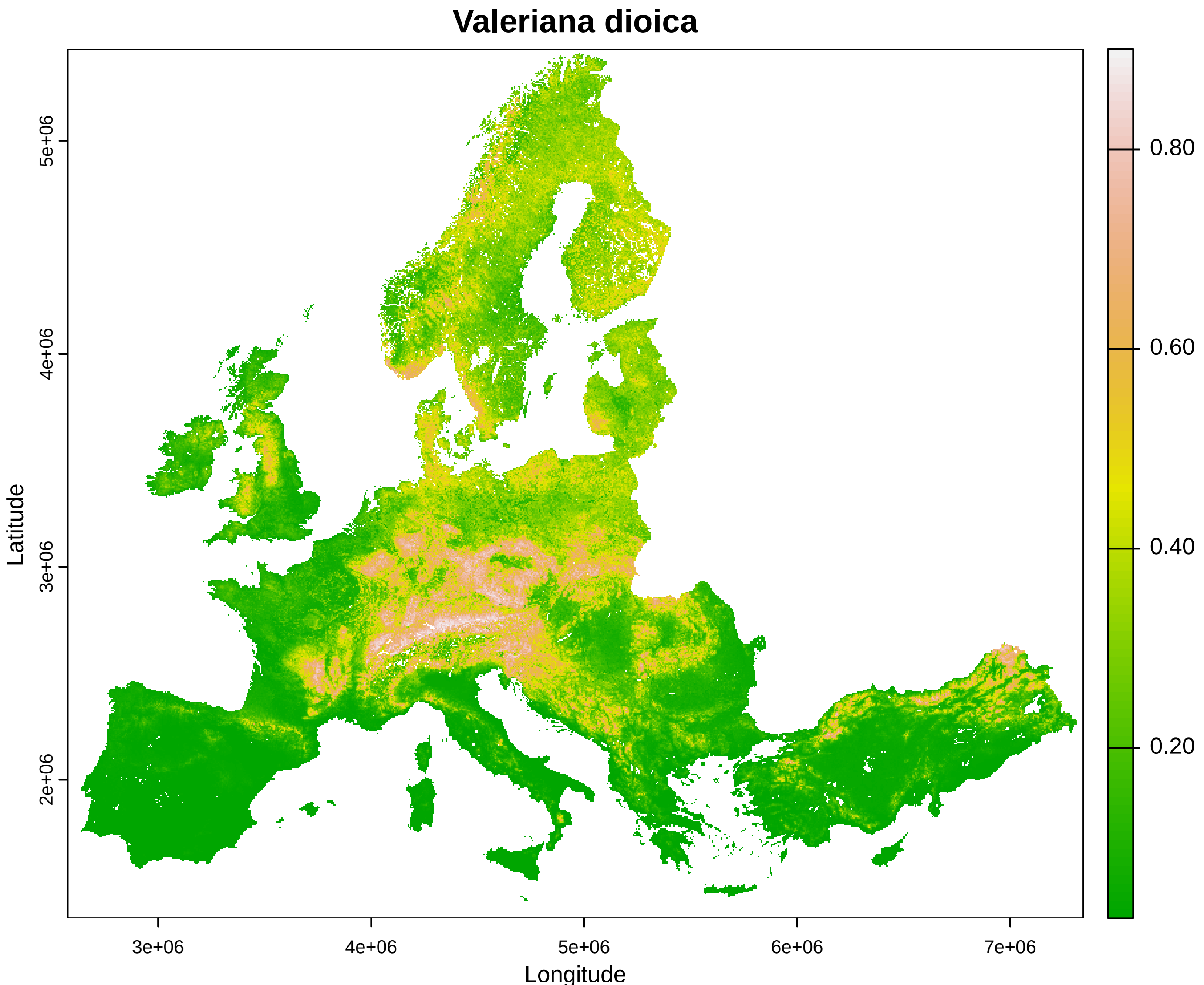

Valeriana dioica
Latitude
5e+06
4e+06
3e+06
2e+06
3e+06
4e+06
5e+06
6e+06
7e+06
Longitude
0.80
0.60
0.40
0.20

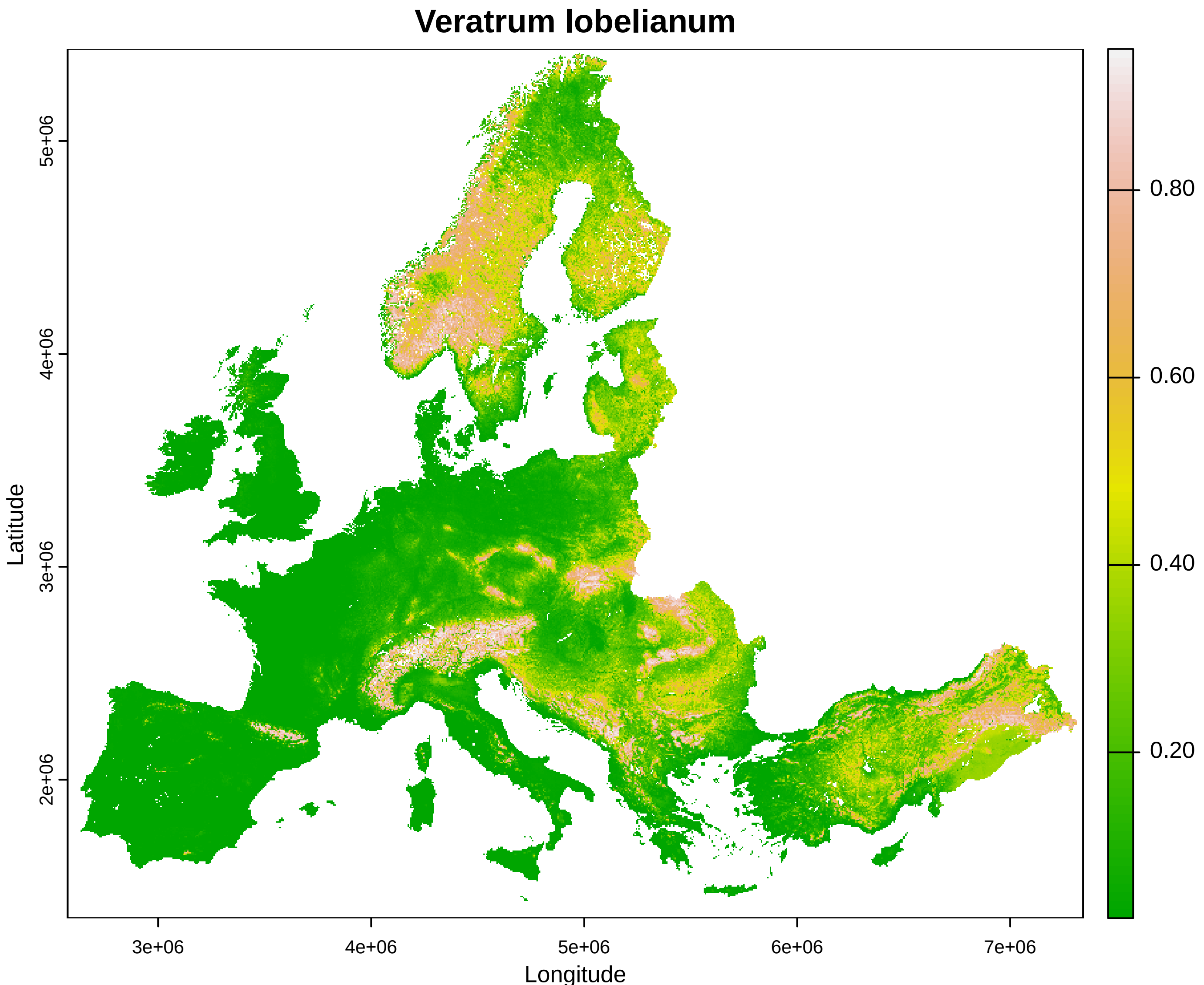
Veratrum lobelianum
Latitude
5e+06
4e+06
3e+06
2e+06
Longitude
3e+06
4e+06
5e+06
6e+06
7e+06
0.80
0.60
0.40
0.20

Viola palustris

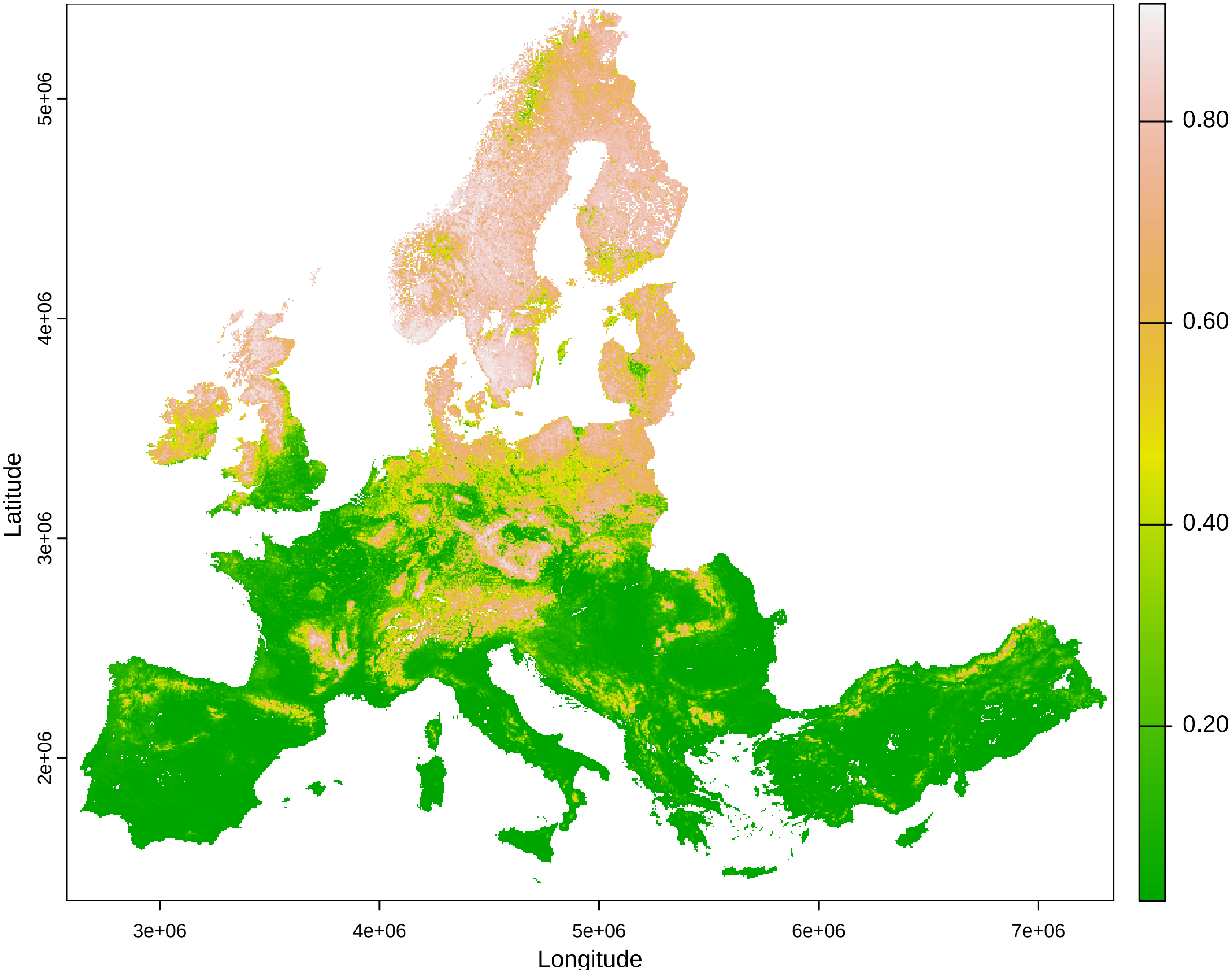

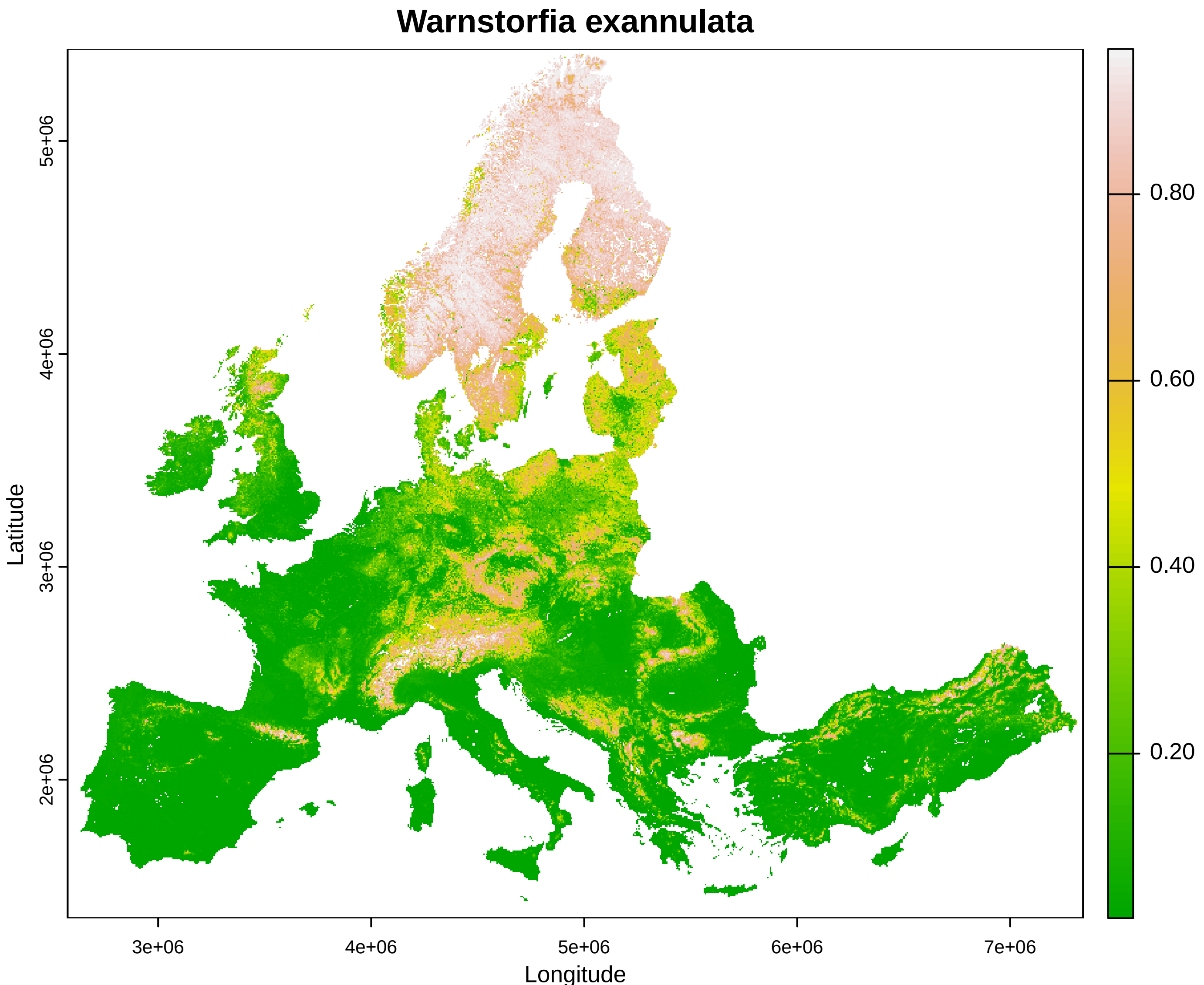
Warnstorfia exannulata
Latitude
Longitude
5e+06
4e+06
3e+06
2e+06
3e+06
4e+06
5e+06
6e+06
7e+06
0.80
0.60
0.40
0.20

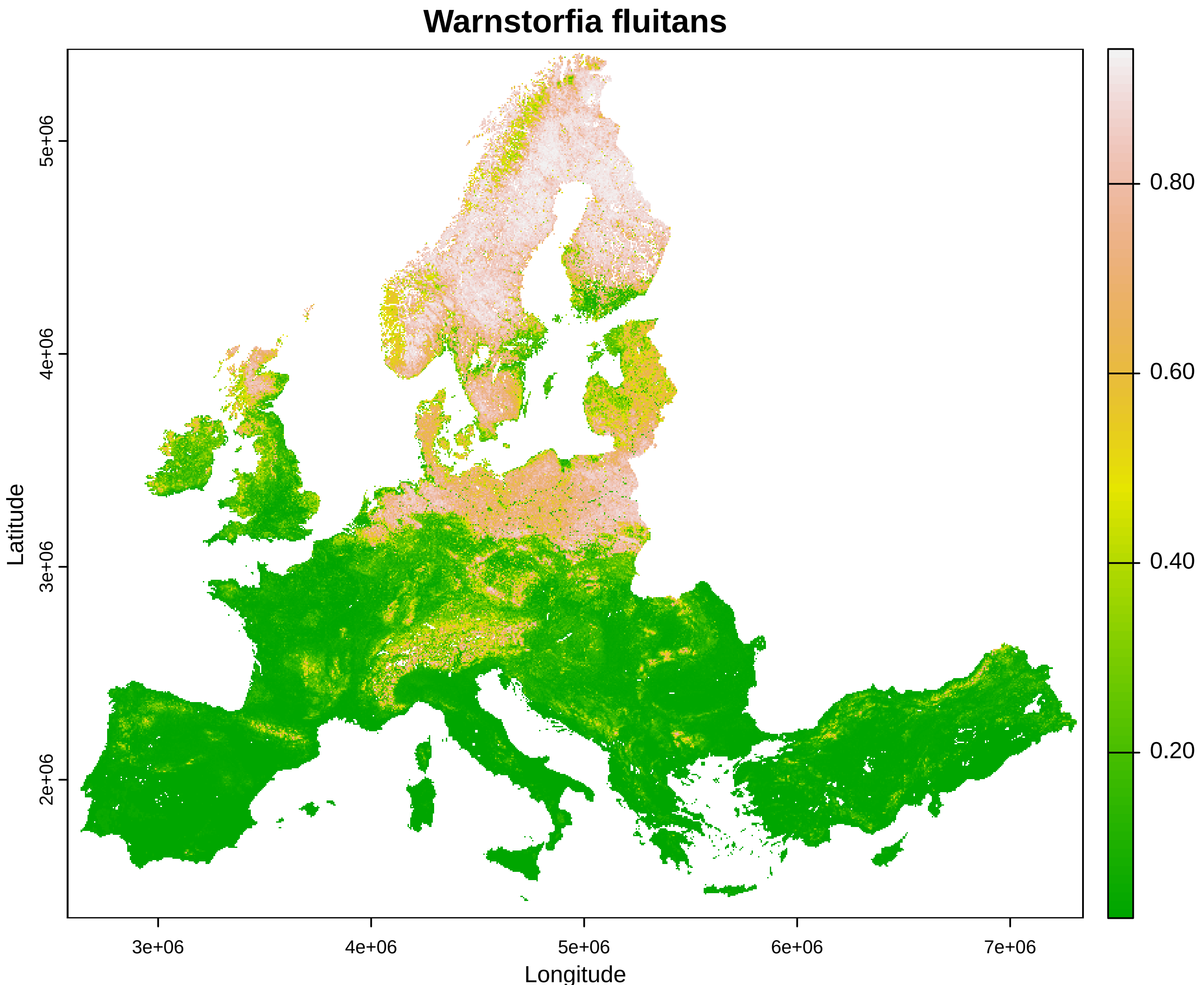

Warnstorfia fluitans
Latitude
Longitude
5e+06
4e+06
3e+06
2e+06
3e+06
4e+06
5e+06
6e+06
7e+06
0.80
0.60
0.40
0.20

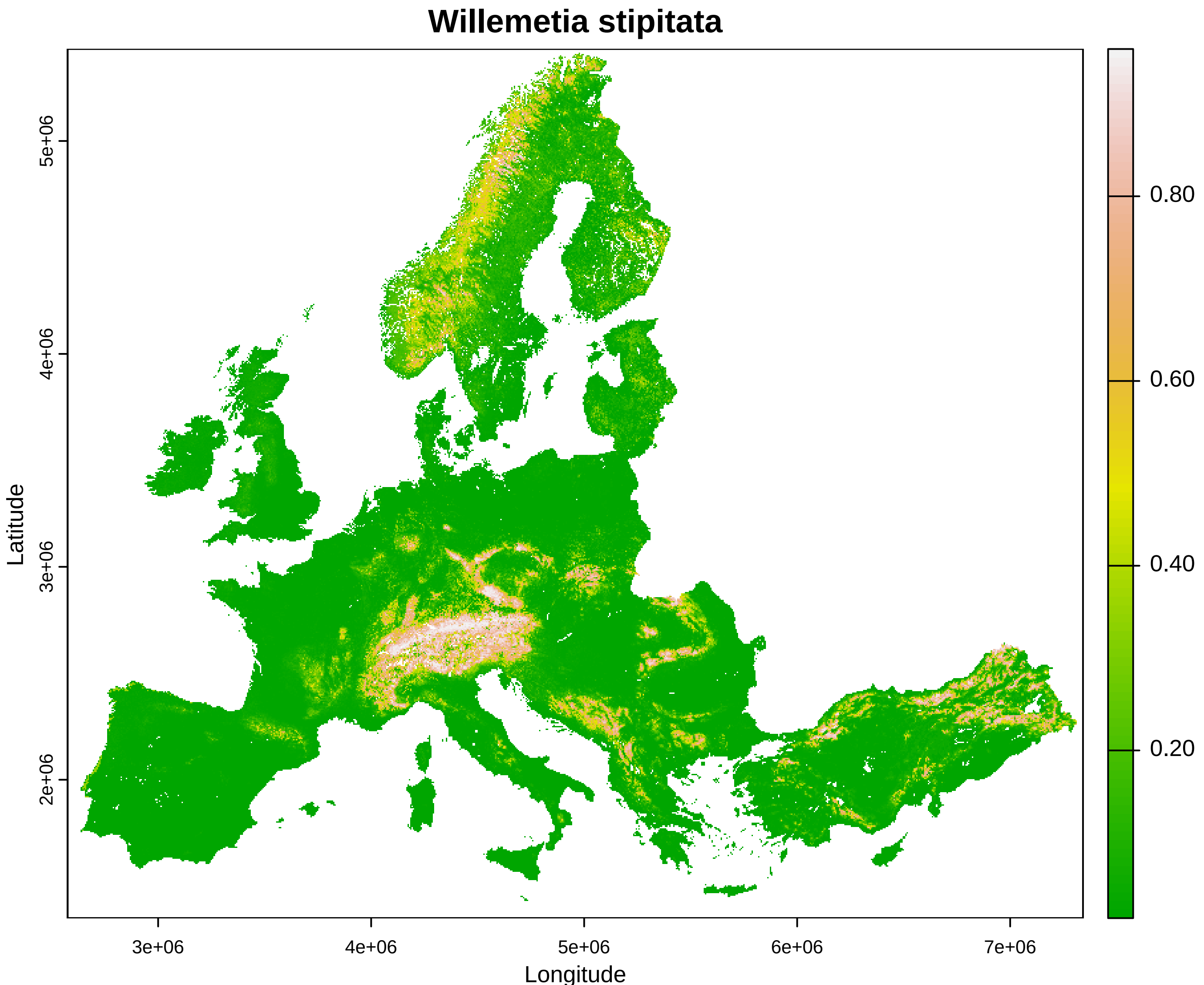
Willemetia stipitata
Latitude
Longitude
5e+06
4e+06
3e+06
2e+06
3e+06
4e+06
5e+06
6e+06
7e+06
0.80
0.60
0.40
0.20
